# Global prevalence of *Giardia* infection in nonhuman mammalian hosts: A systematic review and meta-analysis of five million animals

**DOI:** 10.1371/journal.pntd.0013021

**Published:** 2025-04-24

**Authors:** Kareem Hatam-Nahavandi, Ehsan Ahmadpour, Milad Badri, Aida Vafae Eslahi, Davood Anvari, David Carmena, Lihua Xiao

**Affiliations:** 1 Tropical and Communicable Diseases Research Center, Iranshahr University of Medical Sciences, Iranshahr, Iran; 2 Infectious and Tropical Disease Research Center, Tabriz University of Medical Sciences, Tabriz, Iran; 3 Medical Microbiology Research Center, Qazvin University of Medical Sciences, Qazvin, Iran; 4 Parasitology Reference and Research Laboratory, Spanish National Centre for Microbiology, Health Institute Carlos III, Majadahonda, Spain; 5 CIBERINFEC, ISCIII – CIBER Infectious Diseases, Health Institute Carlos III, Madrid, Spain; 6 Guangdong Laboratory for Lingnan Modern Agriculture, Center for Emerging and Zoonotic Diseases, College of Veterinary Medicine, South China Agricultural University, Guangzhou, China; Cyprus International University: Uluslararasi Kibris Universitesi, CYPRUS

## Abstract

**Background:**

Members of the *Giardia* genus are zoonotic protozoan parasites that cause giardiasis, a diarrheal disease of public and veterinary health concern, in a wide range of mammal hosts, including humans.

**Methodology:**

We conducted a systematic review and meta-analysis to provide evidence-based data on the worldwide prevalence of *Giardia* infection in nonhuman mammals that can be used as scientific foundation for further studies. We searched public databases using specific keywords to identify relevant publications from 1980 to 2023. We computed the pooled prevalence estimates utilizing a random-effects meta-analysis model. Animals were stratified according to their taxonomic hierarchy, as well as ecological and biological factors. We investigated the influence of predetermined variables on prevalence estimates and heterogeneity through subgroup and meta-regression analyses. We conducted phylogenetic analysis to examine the evolutionary relationships among different assemblages of *G. duodenalis*.

**Principal Findings:**

The study included 861 studies (1,632 datasets) involving 4,917,663 animals from 327 species, 203 genera, 67 families, and 14 orders from 89 countries. The global pooled prevalence of *Giardia* infection in nonhuman mammals was estimated at 13.6% (95% CI: 13.4–13.8), with the highest rates observed in Rodentia (28.0%) and Artiodactyla (17.0%). Herbivorous (17.0%), semiaquatic (29.0%), and wild (19.0%) animals showed higher prevalence rates. A decreasing prevalence trend was observed over time (β = -0.1036477, 95% CI -0.1557359 to -0.0515595, *p* < 0.000). Among 16,479 *G. duodenalis* isolates, 15,999 mono-infections belonging to eight (A-H) assemblages were identified. Assemblage E was the predominant genotype (53.7%), followed by assemblages A (18.1%), B (14.1%), D (6.4%), C (5.6%), F (1.4%), G (0.6%), and H (0.1%). The highest *G. duodenalis* genetic diversity was found in cattle (*n* = 7,651, where six assemblages including A (13.6%), B (3.1%), C (0.2%), D (0.1%), E (81.7%), and mixed infections (1.2%) were identified.

**Conclusions/significance:**

Domestic mammals are significant contributors to the environmental contamination with *Giardia* cysts, emphasizing the importance of implementing good management practices and appropriate control measures. The widespread presence of *Giardia* in wildlife suggests that free-living animals can potentially act as sources of the infection to livestock and even humans through overlapping of sylvatic and domestic transmission cycles of the parasite.

## Summary

*Giardia* is a ubiquitous protozoan parasite that causes gastrointestinal illness, known as giardiasis, in both humans and animals. To understand how common this infection is among nonhuman mammals worldwide, we reviewed studies published from 1980 to 2023. We analyzed data from nearly 5 million animals across various species and countries. The findings revealed that approximately 13.6% of these animals were infected with *Giardia*, with the highest rates found in rodents and hoofed animals. Additionally, herbivores, semiaquatic, and wild animals showed elevated infection rates. Interestingly, the overall prevalence of *Giardia* has been decreasing over time. These results highlight that domestic mammals significantly contribute to the spread of *Giardia* in the environment, which can pose risks to humans, emphasizing the importance of developing better management and control strategies. Furthermore, the presence of *Giardia* in wildlife indicates the potential for free-living animals to facilitate the spread of the infection to livestock and humans, underscoring the interconnectedness of sylvatic and domestic transmission cycles.

## Introduction

*Giardia* is a cosmopolitan enteric protozoan parasite that infects various vertebrate hosts, including mammals [1]. An estimated 280 million symptomatic human cases of giardiasis occur worldwide annually [2]. Giardiasis affects approximately 2–5% of the population in developed countries and 20–30% in developing countries [1]. Giardiasis was included in the ‘Neglected Disease Initiative’ launched by the World Health Organization in 2006 due to its burden and close association with poverty [3]. The disease is associated with an estimated loss of 171,100 disability-adjusted life years [4]. Clinical manifestations range from asymptomatic cases to acute diarrhoea and malabsorption [2]. Infected persons are capable of excreting up to 10^10^ cysts daily in their faeces [5]. Similarly, animals can shed large amounts of cysts at the peak of the infection, contributing significantly to environmental contamination [6,7]. Asymptomatic carriers among adult animals are suspected sources of infection for younger animals, highlighting the importance of understanding transmission dynamics [8,9]. Susceptible hosts can become infected either directly via contact with infected individuals/animals or indirectly via accidental ingestion of cysts present in contaminated water or food [10].

For over a century, the taxonomy of *Giardia* has been a subject of controversy, resulting in confusion in naming and understanding the epidemiology of infection, particularly those aspects related to host specificity and zoonotic transmission [11]. Currently, the genus *Giardia* comprises nine valid species, namely *G. agilis*, *G. ardeae*, *G. cricetidarum*, *G. duodenalis* (syn. *G. intestinalis* or *G. lamblia*), *G. microti*, *G. muris*, *G. peramelis*, *G. psittaci,* and *G. varani*. These species have marked differences in morphological characteristics, host range and specificity, and genetic traits [3,12,13]. *Giardia duodenalis* (the only *Giardia* species able to infect humans) is currently regarded as a multispecies complex with eight (A-H) genetic assemblages: assemblages A and B are primarily found in humans and other mammals, C and D in canids, E in wild and domestic ungulates, F in felids, G in rodents, and H in marine pinnipeds [14–16].

Transmission of *G. duodenalis* assemblages is sustained among four major mammalian host groups: humans, pets, livestock, and wildlife [11,17]. Cross-species transmission is common in disrupted habitats where ecological overlap occurs among different species [18–20]. Despite the strong evidence supporting these transmission pathways, the frequency of such events and the specific circumstances under which they occur have yet to be fully resolved [17]. Contamination of surface water with fecal matter of animal origin is an important hypothesized mechanism of zoonotic transmission of *Giardia* [21]. Mammalian wildlife infected with *Giardia* often have easy access to streams and rivers, where they commonly defecate, allowing cysts to be carried over long distances and coming into direct contact with humans through the consumption of drinking or recreational waters. Additionally, wildlife species have been found to harbor (and release) human-infective *G. duodenalis* assemblages, posing a potential threat to water quality and public health [22].

Previous meta-analyses on *G. duodenalis* infection in dogs and cats [23] and cattle [24] revealed large variations in reported prevalence rates across studies, with geography, age of animal, and detection method contributing to these diversities. In this study we aimed to i) investigate the global prevalence of *Giardia* infection in nonhuman mammalian (NHM) species, ii) analyze potential risk factors linked with an increased likelihood of infection, and iii) examine the distribution of *Giardia* species and *G. duodenalis* assemblages among suitable NHM species through a systematic search approach.

## Methods

### Ethics statement

The current study protocol received approval from the Ethics Committee of Iranshahr University of Medical Sciences, Iranshahr, Iran (approval ID: IR.IRSHUMS.REC.1403.007).

### Protocol registration

The systematic review protocol used in this survey has been deposited in the PROSPERO international prospective register of systematic reviews (https://www.crd.york.ac.uk/prospero/) under the registration number CRD42023388395, following the Preferred Reporting Items for Systematic Review and Meta-Analysis Protocol (PRISMA 2020) (S1 Text) [25].

### Research question

The research question was developed based on the CoCoPop framework, which takes into account the condition (parasitisation by *Giardia* spp.), context (global), and population aspects (NHM hosts) of the studies [26].

### Search strategies

Two independent researchers systematically searched for observational studies published between January 1, 1980, and September 1, 2023, across different databases, including CAB Abstracts (https://www.cabi.org/AHPC), Web of Science (https://www.webofknowledge.com/), Scopus (https://www.scopus.com/), and MEDLINE (via PubMed, https://www.ncbi.nlm.nih.gov/pubmed/). Their focus was on studies that reported the prevalence of *Giardia* infections in NHM species as a primary or secondary outcome. The search was last updated on December 31, 2023. The search terms ‘*Giardia*’, ‘giardiasis’, ‘giardiosis’, and a variety of NHM hosts were utilized alone or in combination with the Boolean operators ‘OR’ and/or ‘AND’ (S1 Table). In addition to reviewing the references cited in related systematic reviews, the reference lists of eligible studies were also checked to find additional articles. There was no language limitation for this study. Non-English articles were translated into English utilizing the online tool “Google Translate” (https://translate.google.com/). Occasionally, corresponding authors were personally contacted via e-mail to collect raw data, especially when handling older literature.

### Inclusion and exclusion criteria

Studies were included if they provided details on the prevalence of *Giardia* infection in NHM species (S2 Table). Studies were excluded from the analysis if they i) were assessing the epidemiology of *Giardia* infection in humans, birds, reptiles, or amphibians, ii) included pooled faecal samples, iii) included fecal samples collected from the ground and data for each animal were not independently retrievable, iv) only presented the overall prevalence of gastrointestinal parasites without providing raw data for each parasite, v) conducted molecular characterization of *G. duodenalis* focusing solely on microscopically-positive isolates without primary sample size, and vi) had a sample size < 20 for domestic animals or < 5 for captive wild species [27], or lacked a definite sample size. Abstracts presented in congresses without a clear final result of prevalence, studies comparing diagnostic methods, case-control studies, experimental studies, and clinical trials unable to report a correct estimate of prevalence, longitudinal studies (to address follow-up time bias), case reports or series, letters or commentaries without original data were all removed from analyses. Moreover, we excluded articles if their datasets overlapped with those of other articles included.

### Selection process

**Fig 1:**
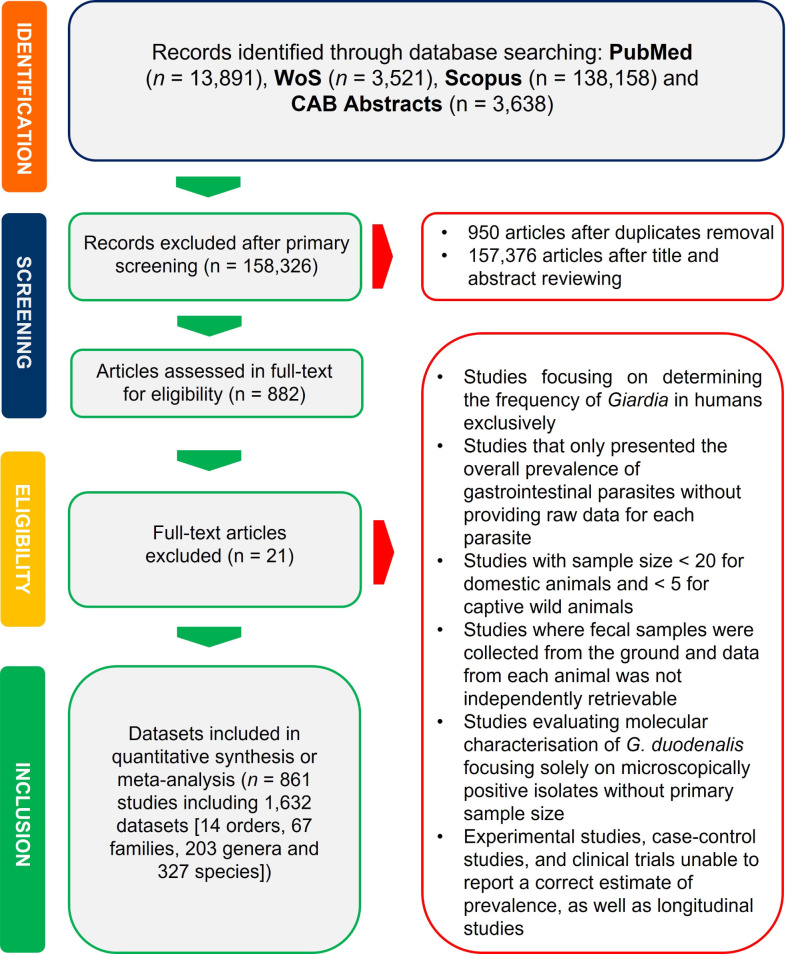
PRISMA flowchart of the search strategy and the selection process of included and excluded studies.

After importing the search results into EndNote X8 software (Thompson Reuters, Philadelphia, USA), duplicated entries were de-duped. The study selection process was conducted in two stages. First, potential eligible studies were identified through screening of the titles and abstracts describing the conducted research. Second, the pre-selected studies underwent a full-text review to determine their compliance with the eligibility criteria. At each stage, two reviewers independently evaluated each article. Discrepancies were resolved through discussion to reach a consensus or, if needed, by arbitration involving a third reviewer. Fig 1 shows a PRISMA flow diagram summarizing the study selection process.

### Data extraction

For each eligible study, the following information was extracted: i) first author’s last name, ii) country, iii) publication year, iv) age range (e.g., pre-weaned, post-weaned, heifer, and adult for cattle), v) sex, vi) study design, vii) detection method(s), viii) gastrointestinal manifestations (diarrhoeic/non-diarrhoeic), ix) animal origin (i.e., wild, domestic, and captive [wild mammals kept in zoos and conservation parks]), x) keeping status (e.g., household pet, stray, breeding kennel, shepherd, and hunting dogs), xi) animal habitat (i.e., terrestrial, semiaquatic, and aquatic), xii) feeding (i.e., herbivorous, carnivorous, and omnivorous), xiii) *Giardia* species (i.e., *G. duodenalis*, *G. peramelis*, *G. microti*, *G. cricetidarum*, and *G. muris*), xiv) *G. duodenalis* assemblages (i.e., A, B, C, D, E, F, G, and H), xv) the number of animals examined, and xvi) number of animals that tested positive for *Giardia* infection. NHM hosts were stratified based on taxonomic classification. If a study presented multiple prevalence rates utilizing various detection methods, only the highest prevalence test result was extracted. That was usually obtained through the most sensitive technique, and it was perceived as the most accurate prevalence data available.

### Risk of bias assessment

The Joanna Briggs Institute prevalence critical appraisal tool [26] was used to assess the methodological quality of each included study. To do so, we examined nine critical aspects of prevalence studies. Each criterion was scored as “yes”, “no”, “unclear” or “not applicable” according to the information provided in the study, with the total score indicating high quality (Q1: low risk of bias) for 7–9 points, moderate quality for 4–6 points (Q2), and low quality for 1–3 points (Q3). The study quality was assessed by two independent reviewers, with any disagreements being resolved through discussion.

### Quantitative analysis

The prevalence of *Giardia* infection in NHM was estimated for each dataset. By utilizing the Freeman-Tukey double arcsine transformation, the variances of prevalence were stabilized, enabling their use in the inverse variance weighting of prevalence. Subsequently, the weighted prevalences were combined individually at the taxonomic ranks of order, family, genus, and species using the DerSimonian and Laird random-effects model [28]. The selection of this model was made based on the expected high heterogeneity deriving from differences in host species and detection methods [23,24]. Heterogeneity was examined using the Cochran *Q* test and *I*^2^ statistic, with an I^2^ value of > 75% defined as high heterogeneity [29]. The sensitivity analyses were carried out to evaluate the strength of pooled estimates after removing each dataset through the jackknife method. Egger’s regression test was utilized to quantitatively evaluate small study effects, with a *p*-value < 0.05 being considered statistically significant. A funnel plot was drawn to qualitatively evaluate publication bias. When dealing with an asymmetric funnel plot, the non-parametric “trim and fill” method was applied to incorporate censored datasets and estimate adjusted effect sizes. Meta-regression and subgroup analyses were conducted to identify the sources of heterogeneity. Corresponding prevalence odds ratios (OR) and 95% CIs were further estimated. Animal host species were stratified based on their taxonomic hierarchy, and pooled prevalence measures were estimated for each rank within the genus, family, and order. The subgroup analyses presented in the included studies were integrated into the initial meta-analysis, with the only exception being when these subgroups were defined by the animals’ country of origin. When a study reported prevalence data for subgroups based on factors such as keeping status, symptoms, and age group, they were treated as separate datasets in subsequent analyses to evaluate the impact of these variables on prevalence rates. Stata software version 18 (Stata Corp., College Station, Texas, USA) was employed for conducting the statistical analyses.

### Retrieving sequences and phylogenetic analyses

A neighbor-joining analysis was used to assess the phylogenetic relationships among assemblages of *G. duodenalis*, with distances calculated by the Jukes-Cantor model. Bootstrapping with 1,000 replicates was used to determine support for the groups generated. Analyses were conducted in MEGA11 [30]. The sequences at three genetic loci, β-giardin (*bg*), glutamate dehydrogenase (*gdh*), and triose phosphate isomerase (*tpi*) from *G. ardeae*, *G. duodenalis*, *G. microti*, and *G. muris* were retrieved from the GenBank database in the FASTA format for this purpose. Initially, these sequences were collected from different mammalian hosts, including beaver, boar, buffalo, cat, cattle, chimpanzee, chinchilla, chipmunk, deer, dog, gorilla, horse, kangaroo, lemur, marmoset, monkey, muskrat, pig, prairie dog, rabbit, raccoon dog, rat, seal, sheep, and wombat. Sequences of human origin were also included in the analyses for comparative purposes. It should be noted that the *bg*, *gdh*, and *tpi* genes are the three markers more commonly used in the literature to assess the genetic diversity of *G. duodenalis*. The *tpi* gene was mapped to chromosome 5, whereas the *bg* and *gdh* genes were mapped to chromosome 4 [31]. Based on the available genome sequence information (corresponding to *G. duodenalis* isolate WB, assemblage A), these three genes are unlinked in the *G. duodenalis* genome, making them suitable for genetic studies [32].

## Results

### Study characteristics and search results

**Fig 2:**
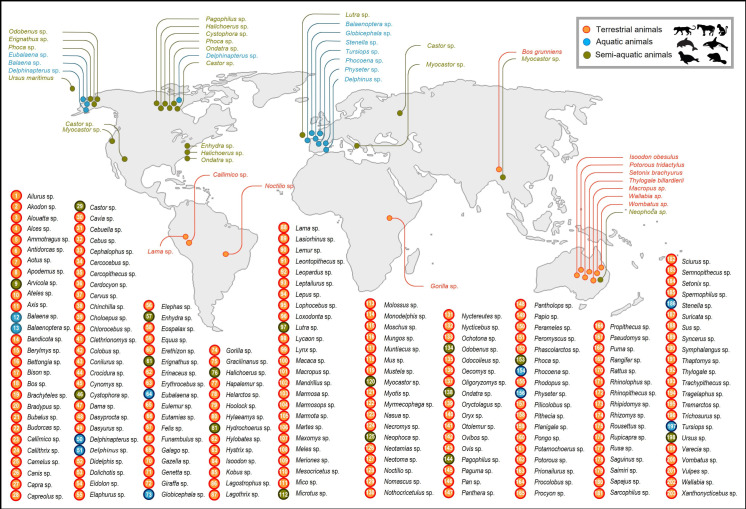
Overview of the nonhuman mammalian hosts (203 genera belonging to 67 families and 14 orders) included in this systematic review and meta-analysis (source of image:https://openclipart.org).

Fig 1 illustrates the process of selecting a study. The initial database search retrieved 159,208 studies from various sources. Subsequently, after the screening process, 158,347 studies were excluded. Eventually, 861 studies (1,632 datasets) met the eligibility criteria for quantitative analysis, covering 4,917,663 animals across 327 species, 203 genera, 67 families, and 14 orders (Fig 2 and Table 1). These studies were conducted in 89 countries across six World Health Organization (WHO) regions (Table 2).

**Table 1:** Pooled *Giardia* infection prevalence rates in nonhuman mammalian hosts. Results from 327 species belonging to 203 genera, 67 families, and 14 orders. The animals were stratified based on taxonomic hierarchy and arranged alphabetically.

Host	No. dataset	No. tested	No. positive	Effect size (95% CI)	Weight (%)	I^2^ (%)	Q	*p*-value
**Total**	**1,632**	**4,917,663**	**161,970**	**0.14 (0.14, 0.14)**	**100**	**99.27**	**176529.61**	**0.00**
**Order Artiodactyla**	**532**	**158,407**	**25,467**	**0.17 (0.16, 0.18)**	**38.70**	**98.61**	**33331.09**	**0.00**
**Family Balaenidae**	**2**	**88**	**48**	**0.55 (0.46, 0.65)**	**0.05**	–	–	–
*Balaena* sp.	1	39	13	0.33 (0.21, 0.49)	0.02	–	–	–
*Eubalaena* sp.	1	49	35	0.71 (0.58, 0.82)	0.03	–	–	–
**Family Balaenopteridae**	**1**	**5**	**2**	**0.40 (0.12, 0.77)**	**0.00**	–	–	–
*Balaenoptera* sp.	1	5	2	0.40 (0.12, 0.77)	0.00	–	–	–
**Family Bovidae**	**379**	**129,593**	**23,309**	**0.20 (0.19, 0.21)**	**29.13**	**98.83**	**29505.08**	**0.00**
*Ammotragus* sp.	1	20	0	0.00 (0.00, 0.16)	0.00	–	–	–
*Antidorcas* sp.	1	5	0	0.00 (0.00, 0.52)	0.00	–	–	–
*Bison* sp.	5	201	31	0.14 (0.06, 0.22)	0.15	60.10	7.52	0.00
*Bos* sp.	228	99,612	19,076	0.21 (0.20, 0.23)	19.26	99.06	22608.66	0.00
*Bubalus* sp.	23	4,845	645	0.12 (0.08, 0.15)	1.77	96.98	636.10	0.00
*Budorcas* sp.	1	191	17	0.09 (0.06, 0.14)	0.10	–	–	–
*Capra* sp.	42	8,791	1,211	0.18 (0.15, 0.21)	3.01	97.63	1642.94	0.00
*Cephalophus* sp.	3	37	0	0.00 (0.00, 0.09)	0.00	–	–	–
*Gazella* sp.	1	7	1	0.14 (0.03, 0.51)	0.01	–	–	–
*Kobus* sp.	1	7	1	0.14 (0.03, 0.51)	0.01	–	–	–
*Oryx* sp.	4	28	4	0.14 (0.00, 0.28)	0.02	–	–	–
*Ovibos* sp.	2	226	22	0.06 (0.03, 0.09)	0.15	–	–	–
*Ovis* sp.	59	15,311	2,285	0.21 (0.18, 0.24)	4.29	98.25	3094.05	0.00
*Pantholops* sp.	1	7	0	0.00 (0.00, 0.40)	0.00	–	–	–
*Rupicapra* sp.	3	219	15	0.06 (0.01, 0.11)	0.24	–	–	–
*Syncerus* sp.	2	75	1	0.02 (0.00, 0.10)	0.11	–	–	–
*Tragelaphus* sp.	2	11	0	0.00 (0.00, 0.28)	0.00	–	–	–
**Family Camelidae**	**24**	**5,174**	**416**	**0.12 (0.08, 0.15)**	**1.52**	**96.44**	**533.84**	**0.00**
*Camelus* sp.	13	2,589	197	0.09 (0.06, 0.13)	0.91	87.51	80.04	0.00
*Lama* sp.	11	2,585	219	0.15 (0.08, 0.23)	0.60	97.68	344.89	0.00
**Family Cervidae**	**63**	**9,790**	**394**	**0.04 (0.03, 0.05)**	**3.99**	**87.56**	**361.82**	**0.00**
*Alces* sp.	6	698	61	0.09 (-0.02, 0.19)	0.27	–	–	–
*Axis* sp.	2	35	1	0.03 (0.01, 0.17)	0.07	–	–	–
*Capreolus* sp.	9	1,000	106	0.10 (0.06, 0.13)	0.60	70.66	27.26	0.00
*Cervus* sp.	24	4,419	85	0.02 (0.01, 0.02)	1.72	71.95	64.18	0.00
*Dama* sp.	4	353	22	0.06 (0.01, 0.11)	0.28	–	–	–
*Elaphurus* sp.	4	85	6	0.25 (0.12, 0.45)	0.02	–	–	–
*Muntiacus* sp.	1	6	0	0.00 (0.00, 0.45)	0.00	–	–	–
*Moschus* sp.	2	425	44	0.04 (0.02, 0.06)	0.21	–	–	–
*Odocoileus* sp.	4	537	7	0.01 (0.00, 0.02)	0.32	–	–	–
*Rangifer* sp.	5	532	23	0.05 (0.01, 0.09)	0.31	–	–	–
*Rusa* sp.	2	1,700	39	0.01 (0.00, 0.01)	0.20	–	–	–
**Family Delphinidae**	**6**	**277**	**15**	**0.05 (0.01, 0.10)**	**0.24**	**50.74**	**6.09**	**0.00**
*Delphinus* sp.	1	133	8	0.06 (0.03, 0.11)	0.10	–	–	–
*Globicephala* sp.	1	7	0	0.00 (0.00, 0.40)	0.00	–	–	–
*Stenella* sp.	2	29	5	0.17 (0.03, 0.31)	0.02	–	–	–
*Tursiops* sp.	2	108	2	0.02 (0.01, 0.07)	0.12	–	–	–
**Family Giraffidae**	**5**	**96**	**16**	**0.20 (0.03, 0.38)**	**0.08**	**70.43**	**10.14**	**0.02**
*Giraffa* sp.	5	96	16	0.20 (0.03, 0.38)	0.08	70.43	10.14	0.02
**Family Monodondidae**	**3**	**45**	**0**	**0.00 (0.00, 0.08)**	**0.00**	–	–	–
*Delphinapterus sp.*	3	45	0	0.00 (0.00, 0.08)	0.00	–	–	–
**Family Phocoenidae**	**1**	**17**	**1**	**0.06 (0.01, 0.27)**	**0.03**	–	–	–
*Phocoena* sp.	1	17	1	0.06 (0.01, 0.27)	0.03	–	–	–
**Family Physeteridae**	**1**	**25**	**4**	**0.16 (0.06, 0.35)**	**0.02**	–	–	–
*Physeter* sp.	1	25	4	0.16 (0.06, 0.35)	0.02	–	–	–
**Family Suidae**	**47**	**13,297**	**1,262**	**0.10 (0.08, 0.12)**	**3.64**	96.67	1111.33	0.00
*Potamochoerus* sp.	1	23	0	0.00 (0.00, 0.14)	0.00	–	–	–
*Sus* sp.	46	13,274	1,262	0.10 (0.08, 0.12)	3.64	96.67	1111.33	0.00
**Order Carnivora**	**523**	**4,683,215**	**126,781**	**0.11 (0.11, 0.11)**	**41.05**	99.55	104857.76	0.00
**Family Ailuridae**	**1**	**10**	**0**	**0.00 (0.00, 0.30)**	**0.00**	–	–	–
*Ailurus* sp.	1	10	0	0.00 (0.00, 0.30)	0.00	–	–	–
**Family Canidae**	**314**	**4,402,487**	**119,320**	**0.13 (0.13, 0.13)**	**26.62**	99.69	97531.66	0.00
*Canis* sp.	289	4,400,219	119,071	0.13 (0.13, 0.14)	25.32	99.72	97230.98	0.00
*Cerdocyon* sp.	1	126	4	0.03 (0.01, 0.08)	0.11	–	–	–
*Lycaon* sp.	4	94	32	0.37 (0.21, 0.53)	0.05	61.18	7.73	0.00
*Nyctereutes* sp.	3	339	25	0.07 (0.05, 0.10)	0.17	–	–	–
*Vulpes* sp.	17	1,709	188	0.12 (0.09, 0.16)	0.97	88.61	97.07	0.00
**Family Felidae**	**165**	**277,348**	**7,113**	**0.08 (0.08, 0.09)**	**13.08**	97.77	6676.65	0.00
*Felis* sp.	147	276,996	7,097	0.08 (0.08, 0.09)	12.65	97.91	6656.24	0.00
*Leopardus* sp.	2	108	2	0.02 (-0.01, 0.04)	0.21	–	–	–
*Leptailurus* sp.	1	7	1	0.14 (0.03, 0.51)	0.01	–	–	–
*Lynx* sp.	3	41	8	0.27 (0.14, 0.44)	0.02	–	–	–
*Panthera* sp.	10	180	6	0.04 (-0.02, 0.10)	0.19	25.83	5.39	0.20
*Prionailurus* sp.	1	5	0	0.00 (0.00, 0.52)	0.00	–	–	–
*Puma* sp.	1	11	2	0.18 (0.05, 0.48)	0.01	–	–	–
**Family Herpestidae**	**2**	**44**	**8**	**0.22 (0.11, 0.37)**	**0.02**	–	–	–
*Mungos* sp.	1	7	0	0.00 (0.00, 0.40)	0.00	–	–	–
*Suricata* sp.	1	37	8	0.22 (0.11, 0.37)	0.02	–	–	–
**Family Mustelidae**	**10**	**1,273**	**90**	**0.08 (0.04, 0.12)**	**0.63**	89.61	76.99	0.00
*Enhydra* sp.	1	103	1	0.01 (0.00, 0.05)	0.13	–	–	–
*Lutra* sp.	1	437	30	0.07 (0.05, 0.10)	0.12	–	–	–
*Martes* sp.	2	27	4	0.15 (0.01, 0.28)	0.03	–	–	–
*Meles* sp.	2	113	21	0.49 (0.35, 0.63)	0.02	–	–	–
*Mustela* sp.	4	593	34	0.06 (-0.00, 0.12)	0.33	86.71	22.58	0.05
**Family Odobenidae**	**1**	**61**	**0**	**0.00 (0.00, 0.06)**	**0.00**	–	–	–
*Odobenus* sp.	1	61	0	0.00 (0.00, 0.06)	0.00	–	–	–
**Family Otariidae**	**1**	**290**	**35**	**0.12 (0.09, 0.16)**	**0.10**	–	–	–
*Neophoca* sp.	1	290	35	0.12 (0.09, 0.16)	0.10	–	–	–
**Family Phocidae**	**13**	**509**	**169**	**0.37 (0.18, 0.55)**	**0.30**	96.17	260.78	0.00
*Cystophora* sp.	1	10	0	0.00 (0.00, 0.30)	0.00	–	–	–
*Erignathus* sp.	1	22	0	0.00 (0.00, 0.15)	0.00	–	–	–
*Halichoerus* sp.	2	46	21	0.42 (0.29, 0.55)	0.03	–	–	–
*Pagophilus* sp.	2	105	31	0.29 (0.21, 0.38)	0.05	–	–	–
*Phoca* sp.	7	326	117	0.38 (0.10, 0.65)	0.21	97.44	234.39	0.01
**Family Procyonidae**	**2**	**89**	**3**	**0.11 (0.04, 0.28)**	**0.03**	–	–	–
*Nasua* sp.	1	27	3	0.11 (0.04, 0.28)	0.03	–	–	–
*Procyon* sp.	1	62	0	0.00 (0.00, 0.06)	0.00	–	–	–
**Family Ursidae**	**12**	**209**	**9**	**0.07 (0.01, 0.13)**	**0.13**	–	–	–
*Helarctos* sp.	1	7	0	0.00 (0.00, 0.40)	0.00	–	–	–
*Tremarctos* sp.	1	28	4	0.14 (0.06, 0.31)	0.02	–	–	–
*Ursus* sp.	10	174	5	0.05 (0.00, 0.09)	0.11	–	–	–
**Family Viverridae**	**2**	**895**	**34**	**0.04 (0.03, 0.05)**	**0.14**	–	–	–
*Genetta* sp.	1	6	0	0.00 (0.00, 0.45)	0.00	–	–	–
*Paguma* sp.	1	889	34	0.04 (0.03, 0.05)	0.14	–	–	–
**Order Chiroptera**	**6**	**223**	**7**	**0.07 (-0.01, 0.15)**	**0.12**	–	–	–
**Family Molossidae**	**1**	**25**	**5**	**0.20 (0.09, 0.39)**	**0.02**	–	–	–
*Molossus* sp.	1	25	5	0.20 (0.09, 0.39)	0.02	–	–	–
**Family Noctilionidae**	**1**	**19**	**1**	**0.05 (0.01, 0.25)**	**0.04**	–	–	–
*Noctilio* sp.	1	19	1	0.05 (0.01, 0.25)	0.04	–	–	–
**Family Pteropodidae**	**2**	**139**	**0**	**0.00 (0.00, 0.02)**	**0.00**	–	–	–
*Eidolon* sp.	1	109	0	0.00 (0.00, 0.03)	0.00	–	–	–
*Rousettus* sp.	1	30	0	0.00 (0.00, 0.11)	0.00	–	–	–
**Family Rhinolophidae**	**1**	**30**	**1**	**0.03 (0.01, 0.17)**	**0.07**	–	–	–
*Rhinolophus* sp.	1	30	1	0.03 (0.01, 0.17)	0.07	–	–	–
**Family Vespertilionidae**	**1**	**10**	**0**	**0.00 (0.00, 0.30)**	**0.00**	–	–	–
*Myotis* sp.	1	10	0	0.00 (0.00, 0.30)	0.00	–	–	–
**Order Dasyuromorphia**	**5**	**236**	**11**	**0.05 (0.02, 0.08)**	**0.16**	–	–	–
**Family Dasyuridae**	**5**	**236**	**11**	**0.05 (0.02, 0.08)**	**0.16**	–	–	–
*Dasyurus* sp.	3	64	2	0.06 (0.02, 0.20)	0.05	–	–	–
*Planigale*	1	5	1	0.20 (0.04, 0.62)	0.00	–	–	–
*Sarcophilus* sp.	1	167	8	0.05 (0.02, 0.09)	0.11	–	–	–
**Order Didelphimorphia**	**9**	**232**	**12**	**0.14 (0.06, 0.21)**	**0.07**	–	–	–
**Family Didelphidae**	**9**	**232**	**12**	**0.14 (0.06, 0.21)**	**0.07**	–	–	–
*Didelphis* sp.	4	172	10	0.15 (0.08, 0.25)	0.05	–	–	–
*Gracilinanus* sp.	1	10	1	0.10 (0.02, 0.40)	0.01	–	–	–
*Marmosa* sp.	2	35	0	0.00 (0.00, 0.10)	0.00	–	–	–
*Marmosops* sp.	1	7	0	0.00 (0.00, 0.40)	0.00	–	–	–
*Monodelphis* sp.	1	8	1	0.13 (0.02, 0.47)	0.01	–	–	–
**Order Diprotodontia**	**38**	**3,824**	**158**	**0.08 (0.06, 0.09)**	**1.15**	87.14	186.68	0.00
**Family Macropodidae**	**22**	**2,534**	**72**	**0.10 (0.05, 0.14)**	**0.63**	84.15	82.00	0.00
*Lagostrophus* sp.	1	6	0	0.00 (0.00, 0.45)	0.00	–	–	–
*Macropus* sp.	17	2,092	66	0.10 (0.04, 0.15)	0.57	86.65	74.92	0.00
*Setonix* sp.	1	15	1	0.07 (0.01, 0.30)	0.03	–	–	–
*Thylogale* sp.	1	13	3	0.23 (0.08, 0.50)	0.01	–	–	–
*Wallabia* sp.	3	425	2	0.10 (0.03, 0.30)	0.02	–	–	–
**Family Potoroidae**	**2**	**90**	**2**	**0.29 (0.08, 0.64)**	**0.00**	–	–	–
*Bettongia* sp.	1	83	0	0.00 (0.00, 0.04)	0.00	–	–	–
*Potorous* sp.	1	7	2	0.29 (0.08, 0.64)	0.00	–	–	–
**Family Vombatidae**	**5**	**597**	**9**	**0.11 (-0.06, 0.27)**	**0.17**	–	–	–
*Lasiorhinus* sp.	1	5	1	0.20 (0.04, 0.62)	0.00	–	–	–
*Vombatus* sp.	4	592	8	0.00 (-0.00, 0.01)	0.17	–	–	–
**Family Phalangeridae**	**7**	**556**	**70**	**16.0 (0.09, 24.0)**	**0.30**	81.37	26.84	0.00
*Trichosurus* sp.	7	556	70	16.0 (0.09, 24.0)	0.30	81.37	26.84	0.00
**Family Phascolarctidae**	**2**	**47**	**5**	**0.13 (0.05, 0.26)**	**0.04**	–	–	–
*Phascolarctos* sp.	2	47	5	0.13 (0.05, 0.26)	0.04	–	–	–
**Order Eulipotyphla**	**9**	**1,730**	**12**	**0.12 (0.05, 0.18)**	**0.07**	–	–	–
**Family Erinaceidae**	**7**	**1,677**	**12**	**0.12 (0.05, 0.18)**	**0.07**	–	–	–
*Erinaceus* sp.	7	1,677	12	0.12 (0.05, 0.18)	0.07	–	–	–
**Family Soricidae**	**2**	**53**	**0**	**0.00 (0.00, 0.07)**	**0.00**	–	–	–
*Crocidura* sp.	2	53	0	0.00 (0.00, 0.07)	0.00	–	–	–
**Order Lagomorpha**	**28**	**6,640**	**631**	**0.14 (0.10, 0.17)**	**1.96**	96.50	628.68	0.00
**Family Leporidae**	**27**	**6,629**	**628**	**0.13 (0.10, 0.17)**	**1.95**	96.64	625.92	0.00
*Lepus* sp.	4	858	76	0.23 (0.18, 0.27)	0.13	–	–	–
*Oryctolagus* sp.	23	5,771	552	0.12 (0.09, 0.16)	1.82	96.63	564.30	0.00
**Family Ochotonidae**	**1**	**11**	**3**	**0.27 (0.10, 0.57)**	**0.01**	–	–	–
*Ochotona* sp.	1	11	3	0.27 (0.10, 0.57)	0.01	–	–	–
**Order Peramelemorphia**	**5**	**225**	**31**	**0.16 (0.05, 0.27)**	**0.28**	94.31	52.72	0.00
**Family Peramelidae**	**5**	**225**	**31**	**0.16 (0.05, 0.27)**	**0.28**	94.31	52.72	0.00
*Perameles* sp.	1	10	0	0.00 (0.00, 0.30)	0.00	–	–	–
*Isoodon* sp.	4	215	31	0.16 (0.05, 0.27)	0.28	94.31	52.72	0.00
**Order Perissodactyla**	**49**	**24,607**	**662**	**0.10 (0.08, 0.12)**	**2.74**	93.63	533.99	0.00
**Family Equidae**	**49**	**24,607**	**662**	**0.10 (0.08, 0.12)**	**2.74**	93.63	533.99	0.00
*Equus* sp.	49	24,607	662	0.10 (0.08, 0.12)	2.74	93.63	533.99	0.00
**Order Pilosa**	**3**	**282**	**3**	**0.00 (-0.00, 0.01)**	**0.15**	–	–	–
**Family Bradypodidae**	**1**	**12**	**2**	**0.17 (0.05, 0.45)**	**0.01**	–	–	–
*Bradypus* sp.	1	12	2	0.17 (0.05, 0.45)	0.01	–	–	–
**Family Choloepodidae**	**1**	**15**	**0**	**0.00 (0.00, 0.21)**	**0.00**	–	–	–
*Choloepus* sp.	1	15	0	0.00 (0.00, 0.21)	0.00	–	–	–
**Family Myrmecophagidae**	**1**	**255**	**1**	**0.00 (0.00, 0.02)**	**0.14**	–	–	–
*Myrmecophaga* sp.	1	255	1	0.00 (0.00, 0.02)	0.14	–	–	–
**Order Primates**	**252**	**12,607**	**1,377**	**0.14 (0.12, 0.16)**	**5.43**	93.17	1785.82	0.00
**Family Aotidae**	**3**	**66**	**4**	**0.08 (0.03, 0.18)**	**0.06**	–	–	–
*Aotus* sp.	3	66	4	0.08 (0.03, 0.18)	0.06			
**Family Atelidae**	**15**	**594**	**171**	**0.30 (0.13, 0.48)**	**0.47**	97.81	457.60	0.00
*Alouatta* sp.	6	395	149	0.41 (0.12, 0.70)	0.29	98.71	386.24	0.01
*Ateles* sp.	6	106	8	0.13 (-0.02, 0.29)	0.09	–	–	–
*Brachyteles* sp.	1	29	1	0.03 (0.01, 0.17)	0.06	–	–	–
*Lagothrix* sp.	2	64	13	0.22 (0.13, 0.34)	0.03	–	–	–
**Family Callitrichidae**	**19**	**542**	**31**	**0.06 (0.03, 0.09)**	**0.47**	37.50	17.60	0.00
*Callimico* sp.	1	6	2	0.33 (0.10, 0.70)	0.00	–	–	–
*Callithrix* sp.	7	121	8	0.06 (-0.01, 0.12)	0.17	44.39	5.39	0.08
*Cebuella* sp.	1	7	2	0.29 (0.08, 0.64)	0.00	–	–	–
*Leontopithecus* sp.	5	302	12	0.06 (-0.01, 0.13)	0.18	–	–	–
*Mico* sp.	1	6	1	0.17 (0.03, 0.56)	0.01	–	–	–
*Saguinus* sp.	4	100	6	0.06 (0.01, 0.10)	0.10	–	–	–
**Family Cebidae**	**22**	**497**	**19**	**0.09 (0.04, 0.14)**	**0.20**	16.68	7.20	0.00
*Cebus* sp.	6	96	4	0.07 (-0.00, 0.14)	0.07	–	–	–
*Saimiri* sp.	9	274	14	0.10 (0.02, 0.18)	0.13	46.84	5.64	0.01
*Sapajus* sp.	7	127	1	0.20 (0.04, 0.62)	0.00	–	–	–
**Family Cercopithecidae**	**128**	**8,721**	**958**	**0.11 (0.09, 0.14)**	**3.29**	93.41	910.78	0.00
*Cercocebus* sp.	7	254	62	0.31 (0.25, 0.37)	0.07	–	–	–
*Cercopithecus* sp.	23	999	111	0.12 (0.07, 0.17)	0.51	75.27	40.44	0.00
*Chlorocebus* sp.	5	56	4	0.20 (0.02, 0.38)	0.02	–	–	–
*Colobus* sp.	7	225	8	0.05 (0.02, 0.08)	0.16	–	–	–
*Erythrocebus* sp.	3	60	5	0.31 (0.14, 0.56)	0.01	–	–	–
*Lophocebus* sp.	1	5	0	0.00 (0.00, 0.52)	0.00	–	–	–
*Macaca* sp.	38	5,640	702	0.10 (0.06, 0.14)	1.83	96.69	693.99	0.00
*Mandrillus* sp.	10	222	5	0.07 (0.01, 0.12)	0.12	0.00	0.21	0.02
*Papio* sp.	14	250	9	0.08 (0.02, 0.13)	0.12	0.00	4.45	0.01
*Piliocolobus* sp.	3	310	27	0.09 (0.03, 0.15)	0.18	–	–	–
*Procolobus* sp.	1	129	12	0.09 (0.05, 0.16)	0.08	–	–	–
*Rhinopithecus* sp.	8	299	10	0.18 (0.07, 0.29)	0.04	–	–	–
*Semnopithecus* sp.	2	10	1	0.20 (0.04, 0.62)	0.00	–	–	–
*Trachypithecus* sp.	6	262	2	0.01 (-0.01, 0.02)	0.15	–	–	–
**Family Galagidae**	**2**	**23**	**0**	**0.00 (0.00, 0.14)**	**0.00**	–	–	–
*Galago* sp.	1	10	0	0.00 (0.00, 0.30)	0.00	–	–	–
*Otolemur* sp.	1	13	0	0.00 (0.00, 0.24)	0.00	–	–	–
**Family Hominidae**	**27**	**1,302**	**96**	**0.15 (0.10, 0.21)**	**0.58**	89.78	107.64	0.00
*Gorilla* sp.	10	871	51	0.09 (0.03, 0.14)	0.47	89.43	47.32	0.00
*Pan* sp.	13	346	36	0.37 (0.22, 0.51)	0.05	47.11	5.67	0.00
*Pongo* sp.	4	85	9	0.14 (0.05, 0.22)	0.05	–	–	–
**Family Hylobatidae**	**13**	**287**	**26**	**0.17 (0.06, 0.28)**	**0.16**	69.69	23.10	0.00
*Hoolock* sp.	1	14	0	0.00 (0.00, 0.23)	0.00	–	–	–
*Hylobates* sp.	7	153	7	0.08 (-0.00, 0.16)	0.12	30.71	4.33	0.06
*Nomascus* sp.	4	114	17	0.24 (0.06, 0.42)	0.04	–	–	–
*Symphalangus* sp.	1	6	2	0.33 (0.10, 0.70)	0.00	–	–	–
**Family Indriidae**	**1**	**43**	**0**	**0.00 (0.00, 0.08)**	**0.00**	–	–	–
*Propithecus* sp.	1	43	0	0.00 (0.00, 0.08)	0.00	–	–	–
**Family Lemuridae**	**18**	**490**	**71**	**0.26 (0.13, 0.40)**	**0.19**	86.94	68.89	0.00
*Eulemur* sp.	1	12	3	0.25 (0.09, 0.53)	0.01	–	–	–
*Hapalemur* sp.	1	44	0	0.00 (0.00, 0.08)	0.00	–	–	*–*
*Lemur* sp.	8	350	65	0.35 (0.14, 0.56)	0.14	92.31	65.00	0.00
*Varecia* sp.	8	84	3	0.09 (-0.01, 0.19)	0.04	–	–	–
**Family Lorisidae**	**3**	**32**	**0**	**0.00 (0.00, 0.11)**	**0.00**	–	–	–
*Nycticebus* sp.	2	20	0	0.00 (0.00, 0.16)	0.00	–	–	–
*Xanthonycticebus* sp.	1	12	0	0.00 (0.00, 0.26)	0.00	–	–	–
**Family Pitheciidae**	**1**	**10**	**1**	**0.10 (0.02, 0.40)**	**0.01**	–	–	–
*Pithecia* sp.	1	10	1	0.10 (0.02, 0.40)	0.01	–	–	–
**Order Proboscidea**	**2**	**58**	**0**	**0.00 (0.00, 0.06)**	**0.00**	–	–	–
**Family Elephantidae**	**2**	**58**	**0**	**0.00 (0.00, 0.06)**	**0.00**	–	–	–
*Elephas* sp.	1	6	0	0.00 (0.00, 0.45)	0.00	–	–	–
*Loxodonta* sp.	1	52	0	0.00 (0.00, 0.07)	0.00	–	–	–
**Order Rodentia**	**171**	**25,377**	**6,818**	**0.28 (0.24, 0.33)**	**8.12**	99.37	21257.18	0.00
**Family Castoridae**	**18**	**3,774**	**1,088**	**0.23 (0.14, 0.31)**	**1.08**	97.31	558.29	0.00
*Castor* sp.	18	3,774	1,088	0.23 (0.14, 0.31)	1.08	97.31	558.29	0.00
**Family Caviidae**	**12**	**1,130**	**57**	**0.04 (0.00, 0.07)**	**0.36**	74.12	11.59	0.04
*Cavia* sp.	7	432	15	0.04 (0.02, 0.06)	0.22	–	–	–
*Dolichotis* sp.	1	15	6	0.40 (0.20, 0.64)	0.01	–	–	–
*Hydrochoerus* sp.	4	683	36	0.02 (0.01, 0.04)	0.14	–	–	–
**Family Chinchillidae**	**11**	**2,438**	**1,086**	**0.41 (0.28, 0.55)**	**0.73**	98.22	561.54	0.00
*Chinchilla* sp.	11	2,438	1,086	0.41 (0.28, 0.55)	0.73	98.22	561.54	0.00
**Family Cricetidae**	**41**	**5,028**	**2,924**	**0.44 (0.30, 0.58)**	**1.64**	99.56	6531.76	0.00
*Akodon* sp.	1	13	1	0.08 (0.01, 0.33)	0.02	–	–	–
*Arvicola* sp.	3	166	85	0.51 (-0.05, 1.06)	0.15	–	–	–
*Clethrionomys* sp.	7	2,375	1,583	0.66 (0.48, 0.85)	0.44	99.11	560.91	0.00
*Hylaeamys* sp.	1	81	1	0.01 (0.00, 0.07)	0.12	–	–	–
*Mesocricetus* sp.	1	11	11	1.00 (0.71, 1.00)	0.00	–	–	–
*Microtus* sp.	12	1,090	804	0.58 (0.28, 0.88)	0.54	99.61	1798.80	0.00
*Necromys* sp.	1	7	1	0.14 (0.03, 0.51)	0.01	–	–	–
*Neotoma* sp.	1	6	1	0.17 (0.03, 0.56)	0.01	–	–	–
*Nothocricetulus* sp.	1	24	3	0.13 (0.04, 0.31)	0.02	–	–	–
*Oecomys* sp.	1	5	1	0.20 (0.04, 0.62)	0.00	–	–	–
*Oligoryzomys* sp.	1	7	1	0.14 (0.03, 0.51)	0.01	–	–	–
*Ondatra* sp.	3	820	314	0.38 (0.35, 0.41)	0.12	–	–	–
*Peromyscus* sp.	4	307	64	0.17 (0.06, 0.27)	0.16	76.87	12.97	0.00
*Phodopus* sp.	2	96	54	0.52 (0.41, 0.62)	0.03	–	–	–
*Rhipidomys* sp.	1	11	0	0.00 (0.00, 0.28)	0.00	–	–	–
*Thaptomys* sp.	1	9	0	0.00 (0.00, 0.33)	0.00	–	–	–
**Family Dasyproctidae**	**1**	**24**	**1**	**0.04 (0.01, 0.20)**	**0.05**	–	–	–
*Dasyprocta* sp.	1	24	1	0.04 (0.01, 0.20)	0.05	–	–	–
**Family Echimyidae**	**4**	**496**	**58**	**0.15 (0.11, 0.18)**	**0.12**	–	–	–
*Myocastor* sp.	4	496	58	0.15 (0.11, 0.18)	0.12	–	–	–
**Family Erethizontidae**	**2**	**16**	**2**	**0.25 (0.07, 0.59)**	**0.01**	–	–	–
*Erethizon* sp.	2	16	2	0.25 (0.07, 0.59)	0.01	–	–	–
**Family Hystricidae**	**1**	**52**	**25**	**0.48 (0.35, 0.61)**	**0.02**	–	–	–
*Hystrix* sp.	1	52	25	0.48 (0.35, 0.61)	0.02	–	–	–
**Family Muridae**	**72**	**11,047**	**1,475**	**0.21 (0.18, 0.24)**	**3.57**	96.22	1639.67	0.00
*Apodemus* sp.	13	1,336	405	0.32 (0.23, 0.42)	0.46	92.54	147.39	0.00
*Bandicota* sp.	1	46	16	0.35 (0.23, 0.49)	0.02	–	–	–
*Berylmys* sp.	1	6	0	0.00 (0.00, 0.45)	0.00	–	–	–
*Conilurus* sp.	1	37	1	0.03 (0.00, 0.14)	0.08	–	–	–
*Maxomys* sp.	1	14	5	0.36 (0.16, 0.61)	0.01	–	–	–
*Meriones* sp.	4	218	20	0.07 (0.02, 0.13)	0.23	49.68	5.96	0.01
*Mus* sp.	16	5,175	410	0.15 (0.10, 0.21)	1.09	97.09	446.11	0.00
*Pseudomys* sp.	1	6	0	0.00 (0.00, 0.45)	0.00	–	–	–
*Rattus* sp.	34	4,209	618	0.22 (0.17, 0.27)	1.68	95.84	696.79	0.00
**Family Sciuridae**	**7**	**884**	**48**	**0.05 (0.01, 0.10)**	**0.42**	87.81	24.60	0.01
*Cynomys* sp.	1	79	11	0.14 (0.08, 0.23)	0.05	–	–	–
*Eutamias* sp.	1	279	24	0.09 (0.06, 0.12)	0.11	–	–	–
*Funambulus* sp.	1	5	5	0.00 (0.47, 1.00)	0.00	–	–	–
*Marmota* sp.	1	399	6	0.02 (0.01, 0.03)	0.14	–	–	–
*Neotamias* sp.	1	8	0	0.00 (0.00, 0.36)	0.00	–	–	–
*Sciurus* sp.	1	15	0	0.00 (0.00, 0.21)	0.00	–	–	–
*Spermophilus* sp.	1	99	2	0.02 (0.01, 0.07)	0.12	–	–	–
**Family Spalacidae**	**2**	**488**	**54**	**0.11 (0.08, 0.14)**	**0.12**	–	–	–
*Eospalax* sp.	1	8	2	0.25 (0.07, 0.59)	0.01	–	–	–
*Rhizomys* sp.	1	480	52	0.11 (0.08, 0.14)	0.12	–	–	–

CI: confidence intervals; I^2^ and Q: heterogeneity measures.

**Table 2:** Global and regional pooled *Giardia* infection prevalence rates in nonhuman mammalian hosts. Results from 1,632 datasets from 89 countries. Countries were grouped according to World Health Organization (WHO) regions and sorted by dataset weight.

WHO region and country	No. dataset	No. animal genera	No. tested	No. positive	Effect size (95% CI)	Weight (%)	*I*^2^ (%)	*Q*
**Global**	**1,632**	**203**	**4,917,663**	**161,970**	**0.14 (0.14, 0.14)**	**100**	**99.27**	**176529.61**
**African**	**97**	**25**	**13,821**	**1,712**	**0.15 (0.13, 0.17)**	**4.97**	**96.69**	**1995.31**
Ethiopia	8	3	2,278	314	0.15 (0.09, 0.22)	0.81	97.37	266.14
Uganda	18	7	1,024	47	0.06 (0.03, 0.08)	0.70	40.26	18.41
Côte d’Ivoire	8	6	1,215	183	0.15 (0.10, 0.21)	0.59	88.62	61.50
Nigeria	8	6	1,215	367	0.32 (0.16, 0.48)	0.48	97.97	294.85
Algeria	6	3	2,557	284	0.16 (0.10, 0.23)	0.44	92.23	51.48
Ghana	4	3	1,150	108	0.09 (0.06, 0.13)	0.43	78.53	13.97
Rwanda	3	3	320	20	0.05 (0.01, 0.09)	0.29	–	–
Mozambique	3	3	696	60	0.08 (0.06, 0.11)	0.26	–	–
Zambia	3	3	637	168	0.25 (0.07, 0.42)	0.20	–	–
Tanzania	3	2	1,002	16	0.13 (–0.04, 0.31)	0.18	–	–
Central African Rep.	10	7	354	3	0.01 (–0.00, 0.02)	0.15	–	–
South Africa	1	1	240	13	0.05 (0.03, 0.09)	0.12	–	–
Kenya	2	1	474	51	0.14 (0.11, 0.18)	0.11	–	–
Cameroon	12	7	287	2	0.05 (–0.02, 0.11)	0.09	–	–
Madagascar	6	3	249	49	0.40 (0.27, 0.54)	0.06	–	–
Gabon	1	1	95	20	0.21 (0.14, 0.30)	0.05	–	–
Namibia	1	1	28	7	0.25 (0.13, 0.43)	0.02	–	–
**Americas**	**374**	**81**	**4,562,828**	**114,969**	**0.13 (0.13, 0.13)**	**25.23**	**99.67**	**95372.30**
USA	134	34	4,486,683	107,060	0.14 (0.13, 0.14)	9.41	99.87	83516.16
Brazil	116	49	22,974	2,495	0.13 (0.11, 0.14)	7.14	95.78	2177.59
Canada	65	21	35,455	2,987	0.14 (0.13, 0.15)	4.72	98.86	4981.21
Argentina	13	7	8,191	797	0.25 (0.20, 0.30)	1.01	98.96	1151.99
Mexico	8	5	1,555	362	0.24 (0.14, 0.34)	0.61	96.69	213.09
Colombia	9	4	2,346	343	0.16 (0.07, 0.25)	0.56	98.04	254.57
Peru	7	3	1,211	263	0.17 (0.06, 0.28)	0.54	97.24	181.37
Ecuador	10	6	428	40	0.10 (0.06, 0.15)	0.37	53.52	17.21
Chile	2	1	1,202	255	0.21 (0.19, 0.23)	0.20	–	–
Cuba	2	1	391	21	0.04 (0.02, 0.06)	0.20	–	–
Costa Rica	2	1	1,194	232	0.19 (0.17, 0.21)	0.18	–	–
Jamaica	1	1	225	44	0.20 (0.15, 0.25)	0.08	–	–
Venezuela	2	1	712	14	0.14 (0.09, 0.23)	0.06	–	–
Grenada	1	1	99	17	0.17 (0.11, 0.26)	0.06	–	–
Trinidad and Tobago	1	1	104	26	0.25 (0.18, 0.34)	0.05	–	–
Nicaragua	1	1	58	13	0.22 (0.14, 0.35)	0.03	–	–
**Eastern Mediterranean**	**108**	**19**	**18,467**	**2,811**	**0.17 (0.15, 0.19)**	**7.39**	**97.03**	**3295.89**
Iran	57	17	7,556	589	0.11 (0.09, 0.13)	3.81	95.43	1051.24
Egypt	18	8	3,659	525	0.18 (0.13, 0.22)	1.41	94.90	333.07
Iraq	21	8	2,974	824	0.26 (0.19, 0.33)	1.17	96.11	513.57
Pakistan	6	3	3,258	731	0.22 (0.13, 0.31)	0.54	97.62	209.76
Israel	2	1	465	93	0.18 (0.15, 0.22)	0.17	–	–
Jordan	2	1	400	30	0.07 (0.04, 0.09)	0.17	–	–
Palestine	1	1	150	5	0.03 (0.01, 0.08)	0.12	–	–
UAE	1	1	5	5	1.00 (0.47, 1.00)	0.00	–	–
**European**	**499**	**83**	**197,495**	**28,724**	**0.19 (0.18, 0.21)**	**30.44**	**99.13**	**47374.90**
Spain	104	56	21,704	3,084	0.16 (0.14, 0.18)	5.61	97.97	4239.60
Italy	59	22	15,264	2,391	0.18 (0.15, 0.21)	4.05	97.40	1846.88
Germany	54	14	88,820	10,714	0.25 (0.22, 0.29)	3.63	99.68	12317.07
Poland	57	22	6,791	2,527	0.23 (0.12, 0.34)	2.71	99.58	10743.78
Portugal	24	8	2,654	415	0.20 (0.09, 0.32)	1.39	98.92	2038.92
Turkey	21	6	2,932	576	0.23 (0.17, 0.29)	1.36	96.25	533.85
Romania	15	6	4,789	873	0.19 (0.14, 0.25)	1.27	98.84	1206.38
UK	14	6	9,933	1,364	0.19 (0.14, 0.25)	1.19	98.28	756.83
Russia	15	10	7,748	649	0.08 (0.05, 0.12)	1.15	96.86	286.79
Belgium	39	25	6,595	1,069	0.23 (0.19, 0.26)	1.03	79.06	114.59
Norway	9	8	3,428	873	0.15 (0.02, 0.28)	0.83	99.29	991.26
Greece	12	7	3,613	571	0.21 (0.11, 0.30)	0.68	98.78	491.54
Croatia	15	10	1,241	118	0.10 (0.05, 0.16)	0.64	92.00	125.07
Denmark	6	4	3,726	879	0.19 (0.07, 0.31)	0.55	98.80	334.67
Czech Republic	5	2	5,227	93	0.03 (0.01, 0.05)	0.55	88.98	36.31
Netherlands	7	4	2,030	454	0.18 (0.11, 0.24)	0.51	90.65	64.14
Austria	6	2	997	129	0.12 (0.06, 0.18)	0.51	90.76	43.27
Ireland	4	2	1,916	278	0.12 (0.02, 0.21)	0.47	98.47	196.02
France	4	3	2,184	555	0.22 (0.08, 0.36)	0.44	98.68	227.67
Serbia	7	2	786	152	0.19 (0.13, 0.24)	0.29	72.13	10.76
Sweden	3	2	677	125	0.24 (0.01, 0.47)	0.25	–	–
Slovakia	3	2	1,034	82	0.18 (0.01, 0.35)	0.25	–	–
Finland	2	1	552	21	0.04 (0.02, 0.05)	0.24	–	–
Switzerland	3	3	1,217	335	0.27 (0.25, 0.30)	0.22	–	–
Scotland	2	2	507	131	0.15 (0.12, 0.18)	0.20	–	–
Greenland	2	2	197	23	0.06 (0.03, 0.09)	0.13	–	–
Hungary	3	2	602	153	0.50 (0.45, 0.56)	0.10	–	–
Bosnia	1	1	123	9	0.07 (0.04, 0.13)	0.09	–	–
Moldavia	1	1	140	63	0.45 (0.37, 0.53)	0.05	–	–
Albania	1	1	58	17	0.29 (0.19, 0.42)	0.03	–	–
Luxembourg	1	1	10	1	0.10 (0.02, 0.40)	0.01	–	–
**South–East Asian**	**61**	**25**	**11,967**	**1,490**	**0.17 (0.15, 0.19)**	**3.34**	**97.77**	**2063.86**
Thailand	17	11	3,120	347	0.18 (0.13, 0.24)	0.97	97.51	522.62
India	12	5	2,676	589	0.26 (0.17, 0.34)	0.83	97.48	436.84
Bangladesh	19	11	3,375	216	0.11 (0.05, 0.17)	0.69	96.24	265.79
Nepal	5	5	556	79	0.15 (0.04, 0.27)	0.30	94.30	52.67
Indonesia	5	2	1,203	26	0.08 (0.05, 0.11)	0.24	–	–
Sri Lanka	2	2	637	143	0.02 (0.01, 0.03)	0.22	–	–
Myanmar	1	1	400	90	0.22 (0.19, 0.27)	0.10	–	–
**Western Pacific**	**493**	**87**	**113,085**	**12,264**	**0.12 (0.12, 0.13)**	**28.64**	**97.57**	**14581.60**
China	332	70	72,550	7,006	0.11 (0.10, 0.12)	18.28	96.92	7045.57
Australia	87	29	17,125	1,755	0.13 (0.12, 0.14)	4.58	97.15	2418.54
South Korea	17	6	7,670	856	0.13 (0.10, 0.15)	1.67	93.72	238.93
Japan	18	6	9,371	1,436	0.15 (0.10, 0.20)	1.66	98.65	1035.70
New Zealand	17	8	3,870	926	0.24 (0.16, 0.31)	1.12	97.32	596.43
Taiwan	7	4	900	100	0.10 (0.06, 0.14)	0.53	74.21	23.26
Malaysia	3	3	684	55	0.07 (0.02, 0.12)	0.33	–	–
Vietnam	5	3	599	112	0.24 (0.12, 0.36)	0.22	88.31	34.23
Philippines	5	4	146	16	0.11 (0.06, 0.16)	0.14	0.00	1.17
Cambodia	2	2	170	2	0.02 (0.01, 0.07)	0.12	–	–

CI: confidence intervals; I^2^ and Q: heterogeneity measures.

*p*-value for heterogeneity across groups was statistically significant except for Cambodia, Cameroon, Central African Republic, Luxembourg, Romania, and Tanzania.

### Pooled prevalence of *Giardia* spp. infection in nonhuman mammals

**Fig 3:**
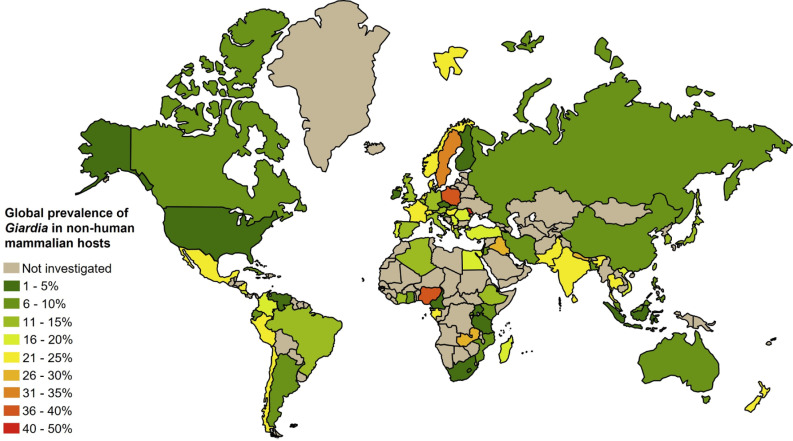
Pooled prevalence of *Giardia* in nonhuman mammals in different countries (source of image:https://commons.wikimedia.org/wiki/File:BlankMap-World.svg).

The pooled prevalence estimates at the global, regional, and national levels are shown in Table 2. Out of 4,917,663 NHM retrieved, 161,970 were found to be positive for *Giardia* infection, yielding an estimated worldwide-pooled prevalence of 14.0% (95% CI: 14.0–14.0). The prevalence estimates for each country are illustrated in Fig 3. High heterogeneity (*I*^2^ = 99.27%) was observed across studies, with notable large sample sizes from four studies in the United States involving around 4.4 million pet dogs and cats [33–36]. By excluding these four studies during sensitivity analyses, the pooled prevalence rates were determined as 16.0% (95% CI: 15.0–16.0) globally, 16.0% (95% CI: 15.0–16.0) for the Americas, and 19.0% (95% CI: 17.0–20.0) for the United States.

S2 Table summarizes the prevalence rates of *Giardia* infection in NHM species (categorized by taxonomic hierarchy) investigated in the 882 studies included in this meta-analysis.

The sensitivity analyses revealed the high stability of our results by demonstrating that there were no significant changes in the overall estimates of the meta-analysis. Egger’s test was significant for the entire dataset (bias: 5.64; 95% CI: 5.06–6.23). The drawn funnel plot (S1A Fig) was also asymmetric, which was highly suggestive of publication bias. Following the application of the trim-and-fill method and the imputation of 734 missing datasets to statistically correct for the bias (S1B Fig), the pooled prevalence rate decreased to 13.6% (95% CI: 13.4–13.8) after correction.

### Stratified prevalence of *Giardia* infection in nonhuman mammals

Based on the information gathered in S2 Table, the pooled prevalence of *Giardia* infection was estimated at 21.0% (30,927/165,356) with copro-antigen detection techniques (CADTs), at 15.0% (15,217/109,392) using PCR, and at 11.0% (115,826/4,642,915) via conventional microscopy (CM). It can be seen that one of the major predictors of prevalence is the detection method employed, with CM performing poorly when compared with CADT (OR = 8.99; 95% CI: 8.86-9.11) or PCR (OR = 6.31; 95% CI: 6.20–6.43). Herbivorous animals had the highest prevalence at 17.0% (26,674/184,845), while carnivorous animals had the lowest at 9.0% (7,555/280,167). Herbivory showed a strong association with increased susceptibility to infection. Animals with terrestrial, semiaquatic, and aquatic habitat types had prevalence estimates of 13.0% (160,060/4,909,836), 29.0% (1,840/7,370), and 22.0% (70/457), respectively. Semiaquatic species had significantly higher odds of being infected (OR = 10.17; 95% CI: 9.64–10.73). Animals of domestic, wild, and captive origins had pooled prevalence estimates of 13.0%, 19.0%, and 10.0%, respectively. A higher likelihood of being infected with *Giardia* was observed in wild animals compared with domestic or captive animals (OR = 6.84; 95% CI: 6.66–7.02) (Table 3).

**Table 3:** Stratified *Giardia* infection prevalence rates in nonhuman mammalian hosts based on ecological and biological characteristics.

Parameters	No. datasets	No. tested	No. positive	Effect size (95% CI)	POR (95% CI)	Weight (%)	*I*^2^ (%)	*Q*
**WHO Region**								
African	97	13,821	1,712	0.15 (0.13, 0.17)	5.46 (5.19, 5.75)	4.97	96.69	1995.31
American	374	4,562,828	114,969	0.13 (0.13, 0.13)	1	25.23	99.67	95372.30
Eastern Mediterranean	108	18,467	2,811	0.17 (0.15, 0.19)	6.94 (6.66, 7.23)	7.39	97.03	3295.89
European	499	197,495	28,724	0.19 (0.18, 0.21)	6.58 (6.49, 6.67)	30.44	99.13	47374.90
South-East Asian	61	11,967	1,490	0.17 (0.15, 0.19)	5.50 (5.20, 5.81)	3.34	97.77	2063.86
Western Pacific	493	113,085	12,264	0.12 (0.12, 0.13)	4.70 (4.61, 4.79)	28.64	97.57	14581.60
**Taxonomic order**								
Artiodactyla	532	158,407	25,467	0.17 (0.16, 0.18)	27.4 (15.6, 53.1)	38.70	98.61	33331.09
Carnivora	523	4,683,215	126,781	0.11 (0.11, 0.11)	3.98 (2.27, 7.72)	41.05	99.55	104857.76
Chiroptera	6	223	7	0.07 (-0.01, 0.15)	4.63 (1.52, 12.9)	0.12	–	–
Dasyuromorphia	5	236	11	0.05 (0.02, 0.08)	6.99 (2.75, 17.5)	0.16	–	–
Didelphimorphia	9	232	12	0.14 (0.06, 0.21)	7.81 (3.16, 19.2)	0.07	–	–
Diprotodontia	38	3,824	158	0.08 (0.06, 0.09)	6.17 (3.42, 12.2)	1.15	87.14	186.68
Eulipotyphla	9	1,730	12	0.12 (0.05, 0.18)	1	0.07	–	–
Lagomorpha	28	6,640	631	0.14 (0.10, 0.17)	15.0 (8.51, 29.3)	1.96	96.50	628.68
Peramelemorphia	5	225	31	0.16 (0.05, 0.27)	22.9 (11.2, 49.6)	0.28	94.31	52.72
Perissodactyla	49	24,607	662	0.10 (0.08, 0.12)	3.95 (2.24, 7.71)	2.74	93.63	533.99
Pilosa	3	282	3	0.00 (-0.00, 0.01)	1.53 (0.27, 5.75)	0.15	–	–
Primates	252	12,607	1,377	0.14 (0.12, 0.16)	17.5 (9.98, 34.1)	5.43	93.17	1785.82
Proboscidea	2	58	0	0.00 (0.00, 0.06)	–	0.00	–	–
Rodentia	171	25,377	6,818	0.28 (0.24, 0.33)	52.6 (30.0, 102.0)	8.12	99.37	21257.18
**Food habits**								
Omnivorous	778	4,452,651	127,741	0.15 (0.15, 0.15)	1.06 (1.04, 1.09)	43.03	99.55	129298.14
Herbivorous	628	184,845	26,674	0.17 (0.16, 0.17)	6.08 (5.92, 6.24)	42.02	98.60	36957.95
Carnivorous	226	280,167	7,555	0.09 (0.08, 0.09)	1	14.96	97.37	7527.76
**Animal habitat**								
Terrestrial	1,567	4,909,836	160,060	0.13 (0.13, 0.14)	1	97.38	99.27	170894.38
Semiaquatic	51	7,370	1,840	0.29 (0.21, 0.37)	10.2 (9.64, 10.7)	2.28	99.00	3801.16
Aquatic	14	457	70	0.22 (0.10, 0.33)	5.53 (4.22, 7.15)	0.34	93.79	128.73
**Animal origin**								
Domestic	945	4,868,444	154,129	0.13 (0.13, 0.14)	1	77.90	99.41	145925.77
Wild	381	39,418	7,207	0.19 (0.18, 0.21)	6.84 (6.66, 7.02)	17.13	99.03	29347.09
Captive	306	9,801	634	0.10 (0.09, 0.12)	2.11 (1.94, 2.29)	4.97	80.42	735.39
**Diagnostic method**								
CADTs	412	165,356	30,927	0.21 (0.20, 0.22)	8.99 (8.86, 9.11)	26.82	98.60	25606.46
CM	642	4,642,915	115,826	0.11 (0.11, 0.11)	1	41.15	99.55	108227.77
PCR	578	109,392	15,217	0.15 (0.15, 0.16)	6.31 (6.20, 6.43)	32.03	98.11	23766.19
**Sample size (*n*)**								
< 100	814	28,320	4,542	0.20 (0.18, 0.21)	6.48 (6.28, 6.70)	19.99	93.67	8264.41
100–500	585	135,420	21,495	0.17 (0.16, 0.18)	6.40 (6.31, 6.51)	51.69	98.52	37415.59
> 500	233	4,753,923	135,933	0.12 (0.12, 0.13)	1	28.32	99.82	119360.02
**Publication period**								
1980–2009	444	1,625,929	73,995	0.16 (0.15, 0.16)	1.73 (1.71, 1.75)	29.43	99.45	68029.24
2010–2023	1,188	3,291,734	87,975	0.15 (0.14, 0.15)	1	70.57	99.02	94365.21
**Risk of bias**								
High	1,367	4,682,443	120,938	0.11 (0.11, 0.12)	1	69.87	99.03	107218.63
Moderate	265	235,220	41,032	0.19 (0.17, 0.20)	7.96 (7.87, 8.06)	30.13	99.56	59457.34

CI: confidence intervals; I^2^ and Q: heterogeneity measures; POR: prevalence odds ratios; CADT: Copro-antigen detection test; CM: Conventional microscopy; PCR: Polymerase chain reaction.

*p*-value for heterogeneity in all sub-groups was statistically significant (*p* = 0.00).

Among cattle (*Bos* sp.), the prevalence estimates were found to be 22.0% (18,705/94,085) in *B. taurus*, 19.0% (199/1,640) in *B. indicus*, and 5.0% (172/3,887) in *B. grunniens* (S3 Table). Pre-weaned calves had significantly higher odds of being infected with the parasite compared to post-weaned calves, heifers, and adult cattle (OR = 4.99; 95% CI: 4.59–5.43). For buffaloes, the prevalences were estimated at 20.0% (25/123) for *Bison bison*, 12.0% (645/4,845) for *Bubalus bubalis*, 8.0% (6/78) for *Bison bonasus*, and 2.0% (1/75) for *Syncerus caffer* (S4 Table). Among small ruminants, *Ovis aries* had a higher pooled prevalence estimate at 21.0% (2,285/15,311) compared to *Capra hircus* at 18.0% (1,211/8,791). Pre-weaned lambs/kids had significantly higher odds of being infected with the parasite compared to other age groups (OR = 2.77; 95% CI: 2.41–3.17) (S5 Table). In camelids, the pooled prevalence estimates were found to be 17.0% (193/2,146) in *Lama pacos*, 12.0% (26/432) in *Lama glama*, 12.0% (107/1,578) in *Camelus dromedarius*, and 6.0% (90/1,011) in *Camelus bactrianus*. There was an association between age and infection, highlighting that animals younger than one year of age had significantly higher odds of being infected (OR = 8.82; 95% CI: 6.57–11.86) (S6 Table). Among omnivorous even-toed ungulates, the prevalence was estimated to be higher in *Sus scrofa domesticus* at 11.0% (1,033/10,141), whereas *Sus scrofa* exhibited a pooled prevalence of 7.0% (229/3,106). The odds of being infected with the parasite were higher in post-weaned pigs compared to fattening pigs (*p* = 0.009) and adult pigs (*p* < 0.0005) (S7 Table). Amongst the odd-toed ungulates, the pooled prevalence rates were found to be 12.0% (105/920) in *Equus asinus* and 9.0% (424/22,895) in *Equus ferus caballus*. An association was observed between age and infection, with young animals under one year of age having significantly higher likelihood of being infected (OR = 1.67; 95% CI: 1.37–2.04) (S8 Table). Regarding canids, the pooled prevalence estimates were found to be 37.0% (32/94) in *Lycaon pictus*, 30.0% (13/44) in *Canis familiaris dingo*, 18.0% (4/20) in *Canis aureus*, 16.0% (58/414) in *Canis lupus*, 15.0% (47/402) in *Canis latrans*, 13.0% (118,949/4,399,339) in *Canis familiaris*, and 12.0% (188/1,709) in *Vulpes* sp. Pet dogs were found to have lower positivity rates compared to stray and breeding dogs. In addition, the pooled prevalence rate was higher in symptomatic pet dogs compared to asymptomatic ones and lower in dogs over one year old compared to those under one year (S9 Table). Among felids, small-sized cats showed a lower pooled prevalence estimate of 8.0% compared to medium/large-sized cats at 12.0%. Pet cats were less likely to test positive than sheltered cats and breeding cats. Additionally, the pooled prevalence was greater in diarrhoeic pet cats compared to non-diarrhoeic ones (S10 Table).

### Meta-regression analyses

Our initial univariate analyses demonstrated significant correlations between the pooled prevalence rate of infection and variables including WHO regions (*p* = 0.003), country (*p* = 0.002), family (*p* = 0.041), genus (*p* = 0.005), species (*p* = 0.002), animal habitat (*p* = 0.001), detection method (*p* < 0.001), publication year (*p* = 0.001), and risk of bias (*p* = 0.001) (Table 4). In the final multivariate analysis, animal habitat (*p* = 0.015), detection method (*p* = 0.000), publication year (*p* = 0.002), and risk of bias (*p* = 0.003) retained statistical significance associated with the pooled prevalence rate of infection. The multivariate analysis also revealed significant correlations between the outcome variable and four study characteristics, including taxonomic order (*p* = 0.002), dietary habits (*p* = 0.002), animal origin (*p* < 0.001), and sample size (*p* < 0.001), that had remained masked following the preliminary univariate analyses (Table 4).

**Table 4:** Study characteristics and estimates of *Giardia* infection prevalence associations. Statistically significant values are bolded.

Variable	Univariate analyses			Multivariate analyses with significant predictors		
	Coefficient (%)	95% CI	*p*-value	Coefficient (%)	95% CI	*p*-value
WHO regions	–0.0214062	–0.0354767 to –0.0073357	**0.003**	–0.0088557	–0.0842535 to 0.0665421	0.818
Country	–0.0014495	–0.0023577 to –0.0005413	**0.002**	–0.0008643	–0.0057381 to 0.0040094	0.728
Order	–0.0016297	–0.0059566 to 0.0026972	0.460	0.0655893	0.0392753 to 0.0919034	**0.000**
Family	–0.0010651	–0.0020844 to –0.0000458	**0.041**	0.0037056	–0.0108709 to 0.0182821	0.618
Genus	–0.0004803	–0.0008139 to –0.0001466	**0.005**	–0.0026778	–0.0124884 to 0.0071328	0.592
Species	–0.0003092	–0.0005093 to –0.0001091	**0.002**	–0.0027486	–0.0077672 to 0.0022710	0.283
Dietary habits	–0.0181263	–0.0523308 to 0.0160782	0.299	0.1077033	0.0407510 to 0.1746556	**0.002**
Animal habitat	0.1580341	0.0619551 to 0.2541131	**0.001**	0.1257504	0.0248568 to 0.2266441	**0.015**
Animal origin	–0.0065572	–0.0348382 to 0.0217238	0.649	–0.0974892	–0.1326343 to –0.0623441	**0.000**
Diagnostic method	–0.0942115	–0.1230812 to –0.0653418	**0.000**	–0.0995677	–0.1304939 to –0.0686416	**0.000**
Sample size	–0.0276875	–0.0599672 to 0.0045921	0.093	–0.0771087	–0.1169384 to –0.0372791	**0.000**
Publication year	–0.1036477	–0.1557359 to –0.0515595	**0.000**	–0.0796326	–0.1307436 to –0.0285216	**0.002**
Risk of bias	–0.1079262	–0.1683888 to –0.0474636	**0.000**	–0.1109589	–0.1852765 to –0.0366413	**0.003**

CI: confidence intervals

### Distribution of *Giardia* species and *G. duodenalis* assemblages

The distribution of *Giardia* species/*G. duodenalis* assemblages by host species and geographical region is summarized in S11 Table. Out of 17,151 *Giardia* isolates (from 410 original papers), the majority were identified as *G. duodenalis* (96.08%), followed by *G. microti* (2.01%), *G. muris* (1.06%), *G. peramelis* (0.50%), and *G. cricetidarum* (0.32%)*. Giardia duodenalis* was detected in almost all mammalian species, while *G. microti* and *G. muris* were found in rodents, *G. peramelis* in marsupials, and *G. cricetidarum* in hamsters. Among 16,479 *G. duodenalis* isolates, 15,999 mono-infections belonging to eight assemblages were identified, with assemblage E being the predominant genotype at 53.65% (8,584/15,999), followed by assemblages A (18.07%), B (14.11%), D (6.44%), C (5.56%), F (1.41%), G (0.61%), and H (0.13%). Furthermore, 415 mixed assemblage infections were identified, with various combinations including A/E (45.30%), C/D (19.76%), A/C (8.43%), A/B (7.95%), A/D (6.51%), A/F (4.82%), B/E (3.37%), A/E/F (1.44%), C/F (0.72%), B/D (0.48%), A/B/D (0.48%), A/B/E (0.24%), D/E (0.24%), and D/F (0.24%). The highest *G. duodenalis* genetic diversity (in terms of number of different assemblages, alone or in combination) was found in cattle (*n* = 7,651, assemblages A-F), followed by dogs (*n* = 2,533, assemblages A-F), sheep (*n* = 1,153, assemblages A, B, D, and E), pigs (*n* = 664, assemblages A-F), and goats (*n* = 529, assemblages A–E) (Table 5).

**Table 5:** The distribution of *Giardia duodenalis* assemblages in domestic and wild populations of nonhuman mammalian species with a larger number of isolates.

Host species	Isolates (*n*)	*G. duodenalis* assemblages (*n*)									References
		A	B	C	D	E	F	G	H	Mixed	
**Family Bovidae**											
*Bos taurus*	7,651	1,042	239	17	6	6,254	0	0	0	93	[37–141]
*Bubalus bubalis*	111	69	4	0	1	37	0	0	0	0	[7,87,114,139,142–148]
*Budorcas taxicolor*	17	0	3	0	0	14	0	0		0	[149]
*Capra hircus*	529	55	12	1	1	443	0	0	0	17	[37,44,80,90,119,141,150–169]
*Ovis aries*	1,153	194	2	0	0	872	0	0	0	85	[37,52,80,82,90,106,119,125,151],[153,157,158,162,164,165,170–186]
*Rupicapra rupicapra*	12	6	0	0	0	5	0	0	0	1	[187,188]
**Family Cervidae**											
*Capreolus capreolus*	21	11	8	0	2	0	0	0	0	0	[189–192]
*Cervus elaphus*	20	6	5	0	1	8	0	0	0	0	[82,106,189–191,193]
*Cervus nippon*	18	4	0	0	0	14	0	0	0	0	[79,82,194–196]
*Dama dama*	8	8	0	0	0	0	0	0	0	0	[197]
*Elaphurus davidianus*	6	0	0	0	0	6	0	0	0	0	[198]
*Moschus berezovskii*	5	2	0	0	0	3	0	0	0	0	[199]
*Moschus chrysogaster*	39	26	0	0	0	7	0	0	0	6	[200]
*Rangifer tarandus*	9	7	0	0	0	2	0	0	0	0	[82]
*Rusa unicolor*	39	28	1	0	0	7	0	0	0	3	[44,201]
**Family Giraffidae**											
*Giraffa camelopardalis*	12	4	0	0	0	8	0	0	0	0	[198,202,203]
**Family Suidae**											
*Sus scrofa*	94	68	8	0	0	18	0	0	0	0	[106,189,191,193,204–208]
*Sus scrofa domesticus*	664	109	39	15	19	475	3	0	0	4	[82,104,116,125,139,209–221]
**Family Camelidae**											
*Camelus bactrianus*	43	16	0	0	0	26	0	0	0	1	[37,222,223]
*Lama pacos*	144	112	0	0	0	31	0	0	0	1	[125,224–227]
**Family Delphinidae**											
*Delphinus delphis*	9	7	2	0	0	0	0	0	0	0	[228,229]
*Stenella coeruleoalba*	5	3	0	0	1	0	1	0	0	0	[230,231]
**Family Canidae**											
*Canis aureus*	4	0	0	0	3	0	0	0	0	1	[189,202]
*Canis familiaris*	2,533	520	109	800	924	32	12	0	0	136	[40,82,99,104,106,134,139,161],[189,202,232–322]
*Canis latrans*	25	8	3	4	8	0	0	0	0	2	[134,244,323,324]
*Canis lupus*	17	7	0	2	4	0	0	0	0	4	[189,190,232,325]
*Canis lupus signatus*	6	0	0	0	4	0	0	0	0	2	[106]
*Nyctereutes procyonoides*	25	0	0	21	2	0	0	0	0	2	[82,326,327]
*Vulpes vulpes*	22	8	7	0	1	1	0	0	0	5	[44,106,189,328–330]
**Family Mustelidae**											
*Meles meles*	10	8	0	0	0	0	0	0	0	2	[331]
**Family Otariidae**											
*Neophoca cinerea*	28	1	27	0	0	0	0	0	0	0	[332]
**Family Phocidae**											
*Halichoerus grypus*	21	6	5	0	0	0	0	0	10	0	[228]
*Phoca vitulina*	22	2	5	0	3	0	0	0	11	1	[228,333]
**Family Felidae**											
*Felis catus*	477	155	59	15	10	6	206	0	0	26	[82,99,134,232,234,236,249,251],[253,255,260,267,269,272,279,281],[289,294,298–300,304,306,307,314],[317,322,334–354]
**Family Viverridae**											
*Paguma larvata*	34	0	33	0	0	0	0	0	0	1	[355]
**Family Dasyuridae**											
*Sarcophilus harrisii*	8 ^a^	0	0	1	0	0	0	0	0	0	[356]
**Family Macropodidae**											
*Macropus fuliginosus*	10	7	3	0	0	0	0	0	0	0	[357,358]
*Macropus giganteus*	19	9	2	4	4	0	0	0	0	0	[44,201]
*Macropus rufus*	6	5	1	0	0	0	0	0	0	0	[357]
**Family Phalangeridae**											
*Trichosurus cunninghami*	7	7	0	0	0	0	0	0	0	0	[357]
*Trichosurus vulpecula*	11	11	0	0	0	0	0	0	0	0	[357,359]
**Family Erinaceidae**											
*Erinaceus europaeus*	10	10	0	0	0	0	0	0	0	0	[360]
**Family Leporidae**											
*Oryctolagus cuniculus*	277	3	249	0	0	19	0	0	0	6	[82,201,361–370]
**Family Peramelidae**											
*Isoodon obesulus*	5	1	0	0	0	1	0	0	0	3	[371]
**Family Equidae**											
*Equus asinus*	230	58	161	0	0	8	0	0	0	3	[372–375]
*Equus ferus caballus*	239	80	103	0	0	53	0	3	0	0	[37,44,104,106,198,373,375–383]
**Family Atelidae**											
*Alouatta caraya*	48	1	47	0	0	0	0	0	0	0	[40,384]
*Alouatta guariba*	16	16	0	0	0	0	0	0	0	0	[385]
**Family Cercopithecidae**											
*Cercopithecus kandti*	17	0	17	0	0	0	0	0	0	0	[386,387]
*Macaca fascicularis*	496	4	492	0	0	0	0	0	0	0	[386,388–392]
*Macaca mulatta*	122	13	106	0	0	3	0	0	0	0	[140,202,386,388,390,393,394]
**Family Hylobatidae**											
*Nomascus leucogenys*	16	0	16	0	0	0	0	0	0	0	[386,390]
**Family Lemuridae**											
*Lemur catta*	46	1	45	0	0	0	0	0	0	0	[198,325,386,387,395,396]
**Family Caviidae**											
*Dolichotis patagonum*	7	3	2	0	0	2	0	0	0	0	[198,325]
**Family Chinchillidae**											
*Chinchilla lanigera*	282	5	232	2	34	6	0	0	0	3	[368,397–399]
**Family Cricetidae**											
*Clethrionomys glareolus*	4	2	2	0	0	0	0	0	0	0	[400]
**Family Echimyidae**											
*Myocastor coypus*	38	2	35	0	0	0	0	0	0	1	[401]
**Family Hystricidae**											
*Hystrix cristata*	15	2	13	0	0	0	0	0	0	0	[402]
**Family Muridae**											
*Bandicota indica*	16	14	2	0	0	0	0	0	0	0	[403]
*Rattus norvegicus*	65	9	0	0	0	0	0	56	0	0	[82,404–407]
*Rattus rattus*	23	0	3	0	0	0	0	20	0	0	[408–410]
*Rattus tanezumi*	39	35	0	2	0	0	0	2	0	0	[403,405]
**Family Sciuridae**											
*Eutamias asiaticus*	24	13	0	0	0	0	0	11	0	0	[411]
*Marmota himalayana*	6	1	4	0	0	1	0	0	0	0	[412]
**Family Spalacidae**											^a^
*Rhizomys sinensis*	52	0	52	0	0	0	0	0	0	0	[413]

^a^TD genotype 1 (*n* = 4); TD genotype 2 (*n* = 3).

**Fig 4:**
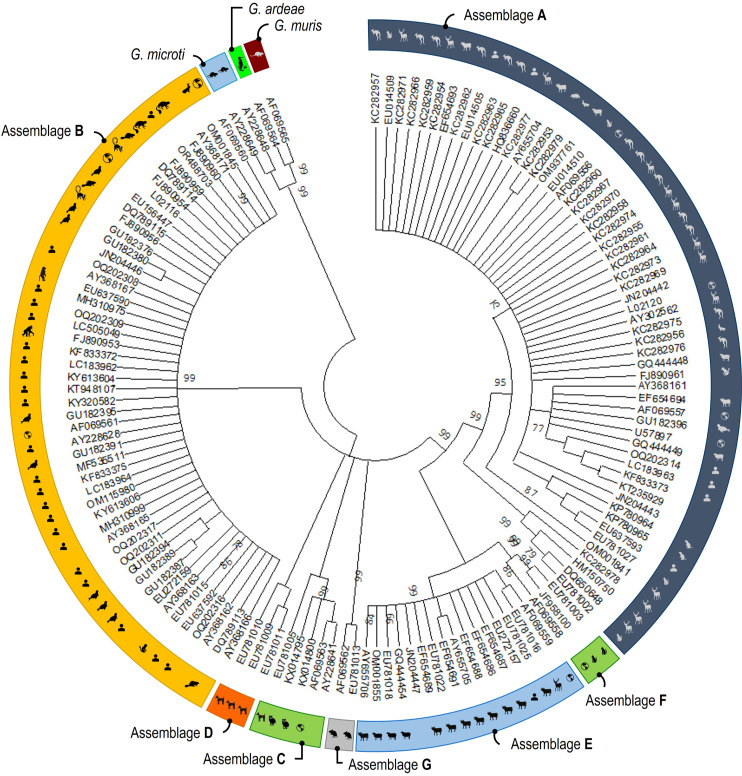
Phylogenetic relationships among *G. duodenalis* assemblages were determined through neighbor-joining analyses, with evolutionary distances calculated using the Jukes-Cantor model at the *tpi* locus based on 141 sequences. The world map logos indicate reference sequences (source of image: https://openclipart.org).

**Fig 5:**
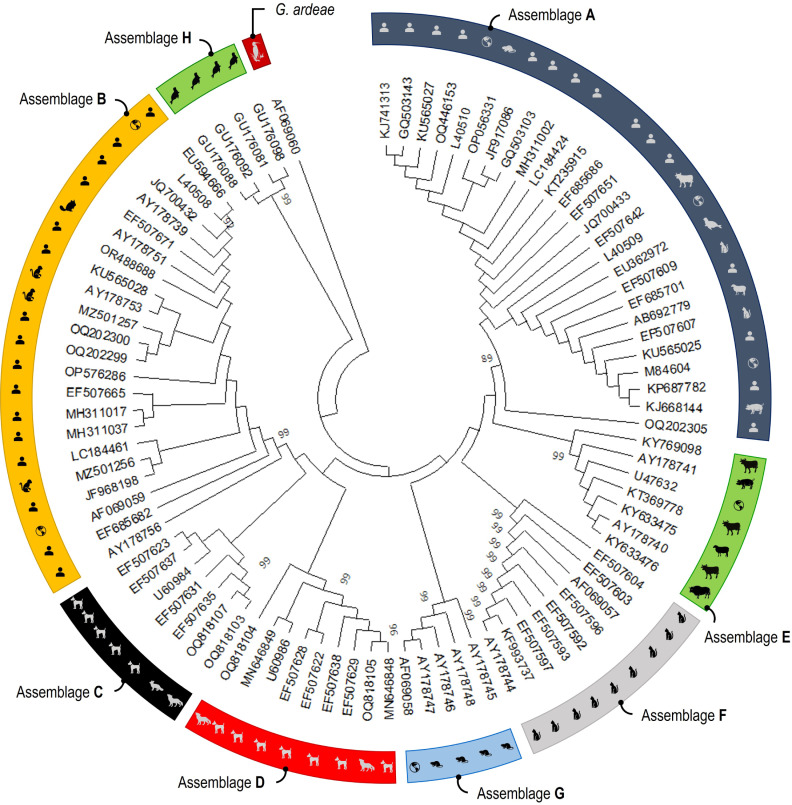
Phylogenetic relationships among *G. duodenalis* assemblages were determined through neighbor-joining analyses, with evolutionary distances calculated using the Jukes-Cantor model at the *gdh* locus using 90 sequences. The world map logos indicate reference sequences (source of image: https://openclipart.org).

**Fig 6:**
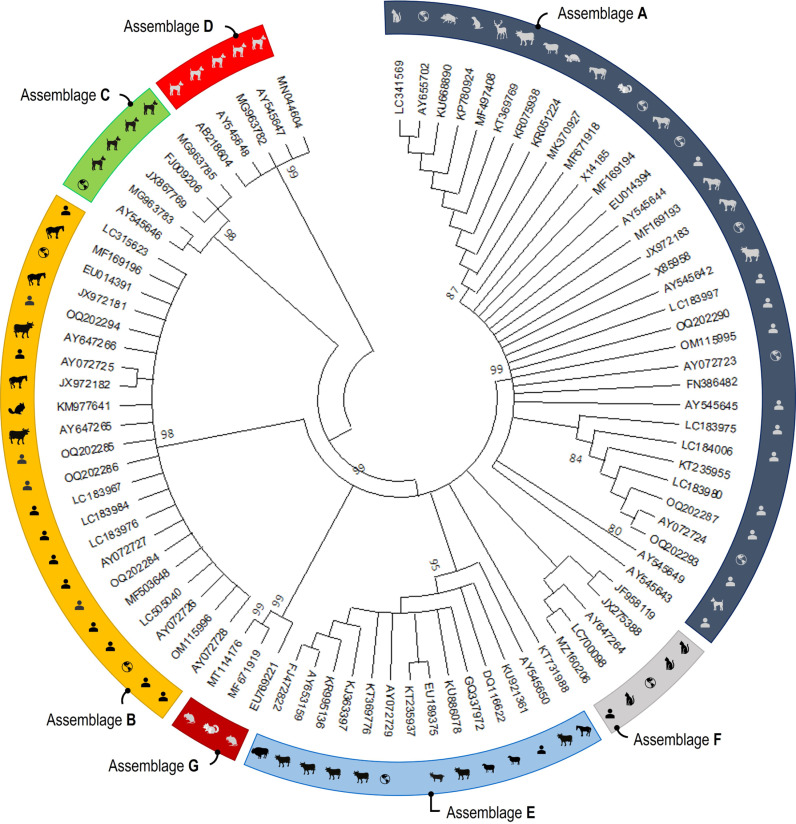
Phylogenetic relationships among *G. duodenalis* assemblages were determined through neighbor-joining analyses, with evolutionary distances calculated using the Jukes-Cantor model at the *bg* locus using 88 sequences. The world map logos indicate reference sequences (source of image: https://openclipart.org).

Phylogenetic analyses consistently grouped the partial nucleotide sequences of the *tpi* (Fig 4), *gdh* (Fig 5) and *bg* (Fig 6) loci into *G. duodenalis* assemblages A to H, as well as *G. ardeae*, *G. microti*, and *G. muris*, with strong support indicated by a posterior probability of 99.

## Discussion

This study utilized data from approximately five million individual NHM to estimate, for the first time, the global prevalence of *Giardia* infection at 14.0%. Substantial heterogeneity was observed across studies, with varying prevalence rates among different animal species. Previous meta-analyses in dogs, cats, and cattle also encountered challenges due to substantial heterogeneity [23,24]. It is crucial to acknowledge that prevalence studies inherently exhibit wide heterogeneity, leading to variability in meta-analyses due to differences in study timing and locations [414].

The study found that the geographical region of origin did have some impact on *Giardia* prevalence heterogeneity, although the effect was not particularly significant. This finding is consistent with that from a previous systematic review in which parasite infections in pet dogs were independent of the sampling region [23]. Evidence of publication bias was also observed, potentially leading to an overestimation of the pooled prevalence. Following the adjustment for publication bias, the pooled prevalence decreased to 13.6%.

Multivariate meta-regression analysis identified animal habitat as a major source of heterogeneity, with semiaquatic mammals showing higher prevalence compared to aquatic and terrestrial mammals. Beavers (*Castor* sp.) and muskrats (*Ondatra* sp.) exhibited prevalence rates approximately 2.5 times greater than the overall pooled prevalence, possibly due to their dual habitation in aquatic and terrestrial environments, increasing exposure to *Giardia* cysts. This finding might pose public health significance if these animals are infected with zoonotic *G. duodenalis* assemblages and inhabit surface waters intended for human consumption. A report from East Texas in USA found that 30.0% of beavers (30/100) and 66.7% of nutria (*Myocastor* sp.) (20/30) were positive for *Giardia* cysts, with infections occurring in all surveyed habitats (pond, creek, and marsh) and a correlation between season and habitat for beavers [415]. The semiaquatic habitat and coprophagy were highlighted as significant factors in *Giardia* epidemiology, contributing to the high susceptibility of these species to infection [415]. Infected beavers and nutria can be constantly re-exposed by ingesting their own faeces directly from the rectum.

The detection method used at initial screening was highlighted as another significant source of heterogeneity, with CADTs and PCR being more sensitive than CM. This result was highly expected as the diagnostic performance of CM is compromised by limited sensitivity and discontinuous cyst shedding in the fecal matter of infected animals. Previous systematic reviews [23,24] also emphasized the influence of diagnostic methods on reported prevalence rates in canine and bovine populations.

A decreasing trend in the prevalence rate of *Giardia* infection over time was observed, possibly due to the implementation of effective control regulations in managing gastrointestinal pathogens in domestic animals. This finding aligns with a prior large-scale study (*n* = 2,468,359) conducted on pet dogs in the USA, demonstrating a decrease in the prevalence of infection from a peak in 2003 (0.61%) to a low in 2009 (0.27%) [36]

The variables of taxonomic order, sample size, food habits, and animal origin were initially masked by confounding factors, but ultimately emerged as significant contributors of prevalence heterogeneity. Feeding habit was identified as another significant source of heterogeneity, with herbivorous mammals showing higher prevalence compared to omnivorous and carnivorous mammals. This finding is in line with earlier global meta-analyses [23,24], which indicated a higher prevalence of *Giardia* infection in cattle (20.0%) compared to dogs (15.2%) and cats (12.0%). The heightened susceptibility of mammalian herbivores to infection may be attributed to their frequent exposure to *Giardia* cysts in pastures and meadows contaminated with fecal material from other animal hosts. Additionally, the origin of the animals emerged as another significant source of prevalence heterogeneity, with higher infection rates observed in wild mammals compared to captive or domestic mammals. The prevalence rate was higher in wild canids (dingo, coyote, wolf, and jackal) (15.0%) and wild felids (serval, lynx, fishing cat, cougar, jaguar, leopard, tiger, and lion) (20.0%) compared to domestic canids (dogs) (13.0%) and domestic felids (cats) (8.0%), respectively. Similarly, Figueiredo et al. [106] found that the prevalence of infection was slightly higher in wild mammalian species (16.6%) than in domestic mammals (15.0%). The lower infection prevalence in domestic and captive animals could be attributed to various factors, including improved hygiene practices, controlled living environments, regular veterinary care, and reduced exposure to contaminated water sources in zoo and domestic settings. Despite this general trend, it should be noted that animals confined in restricted areas (i.e., sheltered dogs or captive nonhuman primates) can harbor relatively high *Giardia* infection rates that are presumably perpetuated through animal-animal contact (see below). Limited studies have been conducted to date on the epidemiology of *Giardia* in captive mammalian species (S2 Table), while conservation parks, zoos, and wildlife rehabilitation centres provide ideal environment to study this parasite given the diverse range of mammalian species available for research over a prolonged period.

The parameters of symptoms, keeping status and age groups, which were only extractable from a limited number of studies focused on domestic mammalian species (including buffaloes, camels, cattle, donkeys, horses, goats, sheep, pigs, dogs, and cats) also affected the prevalence of *Giardia* infections. Similarly, various excellent meta-analyses on *Giardia* in domestic mammals demonstrated that reported prevalence rates were linked to factors such as the age of the animal, whether or not symptomatic and where the animal was housed [23,24]. It has been proposed that the decline in prevalence rates and severity of the infection with animal age is attributed to the development host-mediated immune protective responses [218], although this hypothesis still remains controversial [416].

This study showed that the prevalence rates of *Giardia* infections among domestic ruminants (cattle, sheep, and goats) were higher in farmed populations than in free-ranging populations. Furthermore, the prevalence rate was higher in dogs and cats from shelters and breeding establishments compared to pet dogs and cats in households, showing a high risk of infection acquisition in these populations. Similarly, Swan and Thompson [417] reported a higher prevalence of infection in dogs from refuges (30.0%) and breeding kennels (22.0%) than in those kept as household pets (9.0%). Cats in catteries exhibited a higher prevalence of infection (31.0%) than those from refugees (11.0%) or kept as household pets (8.0%), with breeding cattery cats being group-housed and refuge cats being individually accommodated. This suggests that confinement of a large number of animals in a limited area facilitates transmission of the parasite. The presence of infected animals in such environments would readily lead to contamination, consequently establishing a perpetual source of infection for susceptible animals.

Five *Giardia* species, including *G. cricetidarum*, *G. duodenalis*, *G. microti*, *G. muris*, and *G. peramelis*, were identified circulating in NHM species with different host specificities. Additionally, all known eight *G. duodenalis* assemblages were distributed among NHM species (Table 5). Assemblages A and B have been found in humans and animals, suggesting zoonotic potential, whereas the remaining assemblages are considered host-specific and pose minimal risk of causing human giardiasis. Nonetheless, some studies have indicated the sporadic presence of canine-adapted assemblages C/D, ungulate-adapted assemblage E, and feline-adapted assemblage F in humans [418–423].

Many molecular-based studies in cattle have shown that ungulate-adapted assemblage E is the predominant *G. duodenalis* genetic variant circulating in this host, followed by zoonotic assemblages A and (less commonly) B (Table 5).

Assemblages C, D, and F were reported in only a small number of cattle in a few studies [125,136,424], suggesting that the assemblages may have less strict host-specificity; however, the interpretation of data must still be made cautiously. On the one hand, it is well known that inconsistent genotyping results are often generated among different loci when more markers are utilized [1]. On the other hand, in the studies based only on the detection of DNA in fecal samples, the possibility that cattle could merely be shedding ingested cysts or even DNA through their faeces (mechanical passage) without being truly infected cannot be ruled out [16]. In a study on cattle in Scotland, assemblage B was found to be the second most prevalent (18.2%) following assemblage E (77.2%) at the *bg* locus [108]. Remarkably, some assemblage B isolates of bovine origin had 100% sequence identity with a human isolate (KX960128) from Spain, suggesting that cattle could serve as a reservoir for this assemblage, with potential public health consequences [108]. Some studies have shown substantial genetic variation within assemblage E among cattle [70], with intra-assemblage genetic recombination proposed as a potential explanation [16]. In other bovines, assemblages A, B, D, and E have been identified in yaks (*Bos grunnien*) [170,425], assemblages A and E in American buffaloes (*Bison bison*) [426], assemblage C in European buffaloes (*Bison bonasus*) [325], assemblage D in African buffaloes (*Syncerus caffer*) [325], and assemblages A and E in water buffaloes (*Bubalus bubalis*) [7,142,143,145].

Similar to cattle, goats and sheep are predominantly infected with assemblage E, with zoonotic assemblages A and B being infrequently detected (Table 5). An Italian study found that assemblage B was the cause of a giardiasis outbreak in lambs, leading to severe weight loss [427]. In an Australia study on sheep, assemblage E was identified as the most common genotype (33 isolates) followed by assemblage A (11 isolates) at the 18S rRNA locus, indicating that sheep may not play a significant role as a zoonotic reservoir for the parasite [171]. Two reports have documented assemblages C and D in goats, which were suspected to acquire the infection through ingestion of water or grass contaminated with canine faeces [44,163]. Assemblage E has been reported to exhibit high levels of genetic diversity in sheep, similar to what is observed in cattle [170]. In a recent study conducted in Iran and comprising 200 domestic animals, *Giardia* cysts were found in 4.3% of goats (1/23) and 4.0% of sheep (2/50) by microscopy [10], while 19.3% of cattle (17/88) and 6.7% of camels (2/30) were positive by qPCR [10]. A cattle isolate was successfully genotyped as *G. duodenalis* assemblage B (PQ139658) [428], displaying 99.46% sequence identity with a human isolate (LC184469).

Assemblages A to F have been sporadically documented in domestic pigs, where ungulate-adapted assemblage E was also the most prevalent *G. duodenalis* genetic variant reported [1,2,209]. In a study on pigs in Western Australia, assemblage E was found to be significantly associated with diarrheic stool, while assemblage A showed no such association [209].

Assemblages A to F have been reported in both dogs and cats (Table 5) with assemblages C and D most frequently identified in dogs [429,430] and assemblage F most commonly detected in cats [322]. To date, few studies have been conducted on genotyping *Giardia* in pets and their owners, with the majority of studies indicating that molecular data does not support the household transmission of zoonotic assemblages from pets to humans [306,429–431]. In a pilot study on humans and their pets in Germany, it was observed that a human and a dog living in one household tested positive for assemblage B at the *tpi* locus, with their sequences sharing an identity of only 98% [432].

In contrast to domestic animals, the molecular epidemiology and transmission dynamics *Giardia* infections in wild animals have only been opportunistically studied, resulting in a restricted knowledge of its distribution, genetic variation and zoonotic potential across terrestrial, semiaquatic and aquatic wildlife populations [16].

Assemblages of A, B, E, and F have hitherto been documented in both captive and wild nonhuman primates including lemurs, monkeys and apes worldwide [12,20,198], with A and B being the most prevalent genotypes identified (S11 Table). This suggests that nonhuman primates could potentially act as wildlife reservoirs for zoonotic assemblages [2].

Wildlife species can be infected by human-derived assemblages, indicating their susceptibility to spillover events [17]. A study in the Bwindi Impenetrable National Park in Uganda found infections with assemblage A in both free-ranging human-habituated gorillas (*Gorilla gorilla beringei*) and humans with varying degrees of contact, suggesting a potential introduction of this genotype through habituation activities and its maintenance through anthropozoonotic transmission in their habitats [122]. Another study in muskoxen (*Ovibos moschatus*) in Banks Island of the Canadian Arctic identified *Giardia* isolates belonging to assemblage A, indicating that these herbivorous animals could potentially act as wildlife reservoirs for the parasite, as well as be susceptible to spillover events from human sources [19]. Several authors have suggested that beavers (*Castor canadensis*) could acquire *Giardia* infections (including zoonotic assemblage B) through contaminated water, thereby increasing the quantities of the original contaminating isolate and acting as a potential source of human waterborne outbreaks [22,433]. In addition, studies conducted in live and death sea otters along the California coast in the USA have substantiated the existence of *Giardia* infections, suggesting that sea otters may be contributing to the contamination of surface waters. Previous studies have shown that the parasite can be transported from land to sea through discharge sources such as wastewater effluents and agricultural runoff [134,434,435].

Assemblages A, B, C, D, F, and H of *G. duodenalis* have been previously reported in a variety of aquatic and semiaquatic mammals, including dolphins, seals, sea lions, and whales, with infection rates reaching up to 80% in certain studies [230,436,437] (S11 Table). Exposure to biological pollutants of human fecal origin could be a potential source of infection in these mammals. The identification of assemblages A and B and well as C, D, and F in aquatic mammals supports this hypothesis and suggests a complex epidemiological scenario in which different transmission pathways involving both spurious (mechanical carriage) and true infections coexist in a yet unknown proportion.

Studies on native Australian wildlife have reported the presence of assemblages A, B, C, D and E in 31 marsupial species from nine different families [356,438–440]. Assemblages A and B are frequently found in marsupials, while other assemblages including C, D and E are sporadically reported [44,439], suggesting potential transmission pathways of the parasite assemblages among multiple hosts [440]. Recently, two novel *Giardia* genotypes have been found in the Tasmanian devil (*Sarcophilus harrisii*): TD genotype 1, sharing 94.4% nucleotide identity with assemblage C (GenBank: U60982), and TD genotype 2, sharing 86.9% nucleotide identity with assemblage A from humans in South India (GenBank: JN616252). Remarkably, these genotypes were phylogenetically grouped together within the main *G. duodenalis* clade, distinct from other *Giardia* assemblages, at the *gdh* locus [356].

*Giardia peramelis* (formerly known as the ‘quenda genotype’) was initially found in quendas (*Isoodon obesulus*) [439,441]. A recent study has identified *G. peramelis* at low infection rates (< 4%) in common brushtail possums (*Trichosurus vulpecula*), a brushtail rabbit-rat (*Conilurus penicillatus*), and a northern brown bandicoot (*Isoodon macrourus*) [359], suggesting that it may not be a species specific to quendas. *Giardia peramelis* has also been reported in a calf in Australia [43].

Rodents are commonly infected by *G. duodenalis* assemblage G. Assemblages A to F have also been reported in rodents [400,442]. Some studies indicate a higher occurrence of assemblages A and B in rodents compared to assemblage G, potentially posing a risk for zoonotic transmission from rodents [411,413]. The existence of assemblages A to F in wild rodents could be attributed to the sharing of habitats between wildlife and domestic animals [1,2,443,444]. Synanthropic rodents, such as *Rattus norvegicus*, *Rattus rattus*, and *Mus musculus*, are known to carry and spread *G. duodenalis* assemblages. These rodents have successfully expanded into peri-urban areas, posing a threat to native wildlife [13]. Rodents have also been found to host rodent-specific species of *Giardia*, including *G. microti* and *G. muris* [400,445]. *Giardia microti* has been reported in various rodent genera, such as *Apodemus*, *Arvicola*, *Clethrionomys*, *Eothenomys*, *Microtus*, *Mus*, *Ondatra*, and *Peromyscus* [2,400,404,446,447]. Besides rodents, *G. microti* has also been identified in other species such as *Acinonyx jubatus* and *Panthera pardus japonensis* [325], *Canis familiaris* [448], *Canis lupus*, and *Capreolus capreolus* [189], suggesting that *G. microti* may not be as exclusive to rodents as previously believed.

*Giardia cricetidarum* is the most recently identified *Giardia* species in the Russian dwarf hamster (*Phodopus sungorus*), exhibiting the closest resemblance to *G. muris* based on morphological and molecular characteristics [404]. The absence of reports of *G. cricetidarum* in other host species implies its specificity to hamsters; however, further evidence is necessary to substantiate this assertion.

Limited studies have been conducted on *Giardia* in bats, with sporadic detections and no genetic characterization at the species or assemblage level, making it difficult to ascertain the extent to which bats serve as reservoirs for zoonotic *Giardia* spp. [449].

Wildlife infections with the zoonotic assemblages of *G. duodenalis* can put other free-ranging species at risk [22]. In a paleoparasitological study in Brazil, it was observed that two coprolites from extinct animals, *Nothrotherium maquinense* and *Palaeolama maior*, tested positive for *G. duodenalis* [450], highlighting the susceptibility of wildlife to *Giardia* and underscoring the importance of understanding and mitigating the risks posed by environmental pollution on wildlife health. However, directly attributing giardiasis to the wildlife extinctions seems exaggerated [451]. A recent study at the Bangladesh National Zoo identified assemblage B (GenBank: MK982529) in a *Manis palaeojavanica*, an extinct species of pangolin [202]. However, it seems that they were actually referring to *Manis javanica* [202].

Anthroponotic transmission plays a major role in the epidemiology of human giardiasis, a hypothesis supported by many studies [452,453]. Nevertheless, the risk of human infection with *Giardia* transmitted from various animal species remains a significant public health concern, as evidenced by the diverse range of hosts identified as potential reservoirs for the parasite.

Numerous *G. duodenalis* isolates from various host species across different geographical regions have been genotyped, demonstrating the presence of identical assemblages in both humans and other animals [1]. Such data suggest that *G. duodenalis* assemblages are potentially zoonotic. The broad host range of *G. duodenalis* increases the risk of cross-species transmission, especially in areas where humans and animals live in close proximity. The genetic diversity of *G. duodenalis*, with different genotypes and subtypes exhibiting varying degrees of host adaptation, poses additional challenges in understanding zoonotic transmission. While some studies have indicated that host adaptation may reduce the likelihood of zoonotic transmission, the frequent occurrence of mixed infections and the apparent heterozygosity at certain genetic loci complicate the epidemiological landscape. This genetic variability can influence the virulence and infectivity of different genotypes, potentially leading to outbreaks of giardiasis in human populations [454–457]. The presence of assemblage B in semiaquatic wildlife in investigations of waterborne giardiasis outbreaks has provided the single most important evidence linking *G. duodenalis* to zoonotic transmission [433,458]. However, it has been suggested that beavers and muskrats are more likely to become infected through water contaminated with fecal material of human or domestic animal origin, subsequently amplifying the quantity of the original contaminating isolate [459]. Domestic animals such as cattle, sheep, goats, and pigs may not be important zoonotic reservoirs for *G. duodenalis*. The public health risk of giardiasis from these animals is minimal, as the human-pathogenic assemblages A and B likely compete with the more prevalent assemblage E in these livestock (Table 5). Similar conclusions can probably be drawn for domestic pets, such as dogs and cats, which predominantly carry host-adapted assemblages C, D, and F as their dominant genotypes (Table 5). Assemblage A is the most prevalent non-host-specific genotype found in NHM species (S11 Table). Animals are most commonly infected by subassemblage AI, while humans are predominantly infected with subassemblage AII (S12 Table). To achieve a more accurate evaluation of zoonotic transmission, studies must investigate the transmission dynamics of *G. duodenalis* between animals and humans cohabiting in the same household or localized area of endemicity. Carefully designed epidemiological studies involving cats, dogs, and their owners, using subtyping tools, are essential for accurately quantifying the spillover and spillback of *G. duodenalis* between pets and their owners [460]. In Italy, in a socially deprived community where dogs roamed freely, only subassemblage AI was detected in both children and dogs [278]. Likewise, a Brazilian study found that both children and dogs living in the same household were infected with subassemblage AI [234]. Other molecular-based studies investigating zoonotic transmission between humans and domestic pets sharing the household have not found evidence of such events. For instance, studies conducted in Spain and Germany [306,432] support the notion that dogs and cats are not relevant reservoirs for human giardiasis. In contrast, an Indian study found subassemblage AII in two isolates from humans and one isolate from a dog in the same household [461]. Additionaly, subassemblage AII was found to be the dominant subtype in both humans and a dog living on a tea estate [461].

Moreover, the presence of *Giardia* in asymptomatic animals further complicates the risk assessment, as these animals can shed infectious cysts into the environment without displaying any signs of illness [10,428,462]. One of the significant risks associated with zoonotic giardiasis is the environmental resilience of *Giardia* cysts. They can remain infectious for extended periods in soil and water, accumulate in the environment and increase the likelihood of human exposure through contaminated water sources or food.

Understanding these risks is crucial for developing effective public health strategies to mitigate the transmission of *G. duodenalis* and reduce the burden of giardiasis in humans. Continued research into the molecular epidemiology of *Giardia*, alongside improved sanitation and hygiene practices, will be essential in addressing the challenges posed by this pathogen.

The strengths of the present meta-analysis lie in our commitment to transparency and reproducibility, exemplified by the registration of our study protocol on PROSPERO to ensure the thorough documentation and accessibility of our methodology. Through the strategic use of four databases, we provide a comprehensive depiction of *Giardia* infection prevalence in animals. In a departure from the conventional focus on livestock, our study delved into the global prevalence of *Giardia* infection in NHM species, furnishing a more holistic view of the parasite’s epidemiology and transmission dynamics. Additionally, we defined new predictors such as taxonomic hierarchy, animal origin, food habits, and habitat that likely influence infection prevalence. Moreover, we detailed the distribution of *G. duodenalis* assemblages in these animals, a critical aspect in understanding the potential zoonotic risks associated with infection and pinpointing common transmission sources among diverse host species. One of the limitations of our study was that the majority of existing studies on wildlife were conducted with small sample sizes, hindering the generalizability of results to larger populations and limiting the ability to draw robust conclusions regarding risk factors.

The results of the meta-analysis highlight the common occurrence of *Giardia* infection in NHM. Wild mammals exhibit the highest prevalence compared to domestic or captive mammals. Herbivorous animals are notably more affected compared to omnivorous and carnivorous species. Semiaquatic animals display a higher prevalence than aquatic and terrestrial animals. Rodentia and Artiodactyla stand out for having the highest prevalences of infection in comparison to other mammalian orders. These results emphasize the importance of monitoring and addressing *Giardia* infections in wildlife populations to safeguard animal health and potentially reduce transmission risks to other species, including humans. It is evident that studies relying on direct microscopy will underestimate prevalence significantly compared to immune-based or PCR detection methods. Major domestic animal hosts such as cattle, buffaloes, camels, llamas, sheep, goats, pigs, horses, donkeys, dogs and cats are potent reservoirs for six assemblages of *G. duodenalis* (A to F). Cross-species transmission of *G. duodenalis* is affected by interspecies contact and infection pressure in intensive settings (e.g., refuge shelters for cats and dogs), allowing for the propagation of both zoonotic and non-zoonotic assemblages. Future investigations focusing on identifying specific *G. duodenalis* assemblages in wildlife could provide valuable details about transmission routes and the potential role of NHM species as zoonotic reservoirs, or their susceptibility to spillover events. Understanding these dynamics is crucial for effective management strategies and public health interventions aimed at reducing the transmission of *Giardia* infections among animal populations and potentially to humans.

## Supporting information

S1 Fig Funnel plots with pseudo 95% Confidence Intervals (95% CI). A) Showing publication bias. B) Showing imputed missing datasets to correct for publication bias (represented by filled squares).
(DOC)

S1 Table Full search strategies utilized for the databases of Medline/PubMed, Web of Sciences, Scopus and CAB Abstracts. (DOC)

S2 Table Worldwide prevalence of *Giardia* infection in nonhuman mammals. The animals were sorted according to the taxonomic hierarchy, and then by the year of publication of the studies (*n* = 882).
(DOC)

S3 Table Stratified prevalence of *Giardia duodenalis* infection in cattle according to *a priori* defined sub-groups. (DOC)

S4 Table Stratified prevalence for *Giardia duodenalis* infection in buffaloes according to *a priori* defined sub-groups. (DOC)

S5 Table Stratified prevalence of *Giardia duodenalis* infection in sheep and goat according to *a priori* defined sub-groups. (DOC)

S6 Table Stratified prevalence of *Giardia duodenalis* infection in camels and alpacas according to *a priori* defined sub-groups. (DOC)

S7 Table Stratified prevalence of *Giardia duodenalis* infection in domestic and feral pigs according to *a priori* defined sub-groups. (DOC)

S8 Table Stratified prevalence of *Giardia duodenalis* infection in horses and donkeys according to *a priori* defined sub-groups. (DOC)

S9 Table Stratified prevalence of *Giardia duodenalis* infection in wild and domestic canids according to *a priori* defined sub-groups. (DOC)

S10 Table Stratified prevalence of *Giardia duodenalis* infection in domestic, captive and wild felids according to *a priori* defined sub-groups. (DOC)

S11 Table Worldwide occurrence of *Giardia* species or genotypes in domestic and wild populations of nonhuman mammalian species. (DOC)

S12 Table *Giardia duodenalis* subassemblages AI and AII in humans and animals. (DOC)

S1 Text PRISMA checklist. (DOC)
