# Supplementary material for: Global prevalence of Giardia infection in nonhuman mammalian hosts: A systematic review and meta-analysis of five million animals
Source: PLoS Negl Trop Dis. 2025 Apr 24;19(4):e0013021. doi: 10.1371/journal.pntd.0013021 (PMC12052165; doi:10.1371/journal.pntd.0013021)

**S1 Fig.** Funnel plots with pseudo 95% Confidence Intervals (95% CI). A) Showing publication bias. B) Showing imputed missing datasets to correct for publication bias (represented by filled squares).


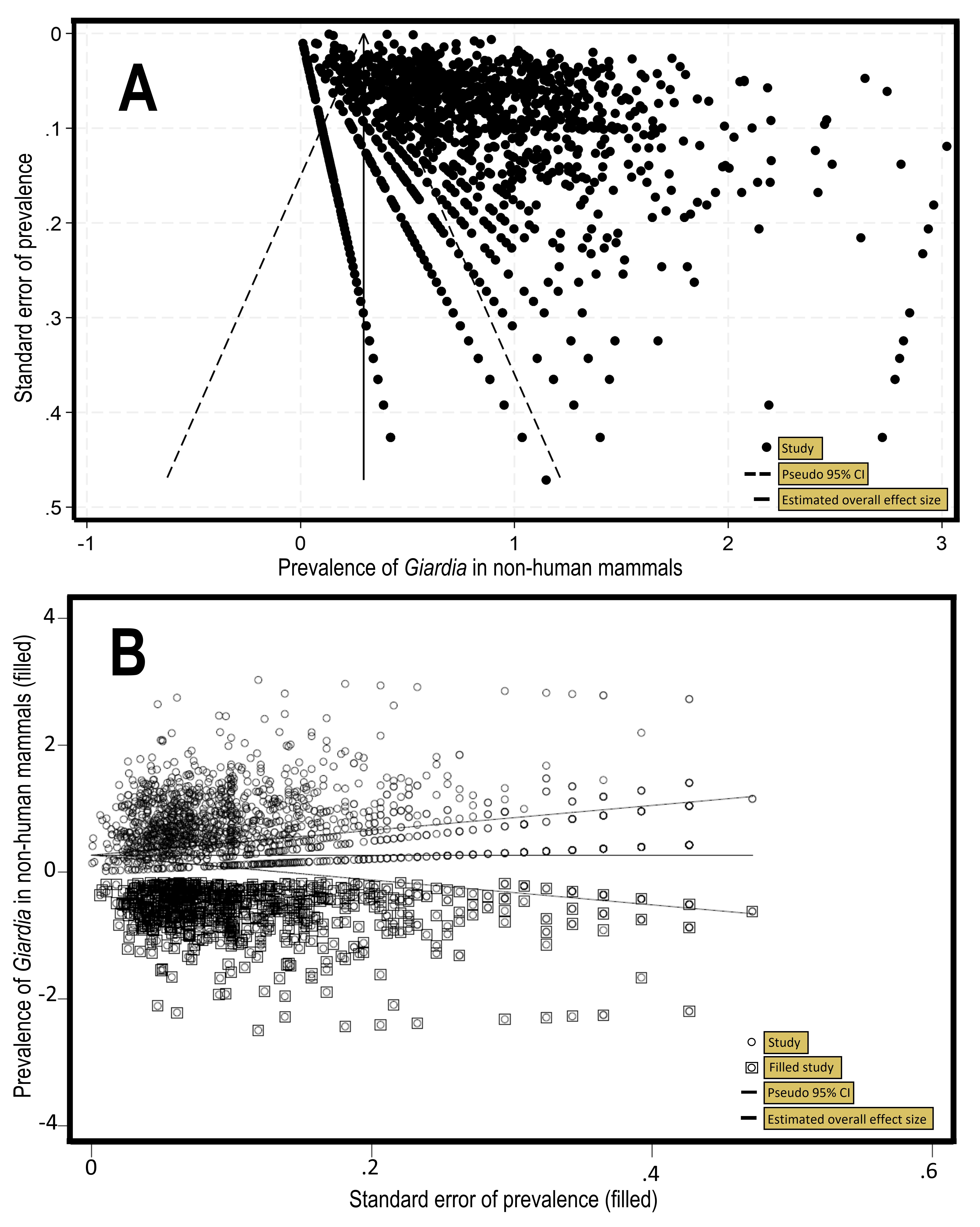

Supplement: S1 Fig — A) Showing publication bias. B) Showing imputed missing datasets to correct for publication bias (represented by filled squares). (DOC) [file pntd.0013021.s001.doc]
