# Supplementary material for: Global prevalence of Giardia infection in nonhuman mammalian hosts: A systematic review and meta-analysis of five million animals
Source: PLoS Negl Trop Dis. 2025 Apr 24;19(4):e0013021. doi: 10.1371/journal.pntd.0013021 (PMC12052165; doi:10.1371/journal.pntd.0013021)
Supplement: S1 Table — (DOC) [file pntd.0013021.s002.doc]

**S1 Table.** Full search strategies utilized for the databases of Medline/PubMed, Web of Sciences, Scopus and CAB Abstracts.

| **Database: Medline/PubMed** |
| --- |
| (Giard*** OR *Giardia* OR "*Giardia lamblia*" OR "*Giardia duodenalis*" OR "*Giardia intestinalis*" OR "*Lamblia intestinalis*" OR giardiasis OR giardiosis OR lambliasis OR "*Giardia* infection" OR (*Giardia* AND infection) OR "*Giardia duodenalis* infection" OR (infection AND "*Giardia duodenalis*") OR "*Giardia lamblia* infection" OR (infection AND "*Giardia lamblia*") OR "*Giardia intestinalis* infection" OR (infection AND "*Giardia intestinalis*")) AND (dog OR canids OR dingo OR wolf OR coyote OR fox OR jackal OR cat OR felids OR caracal OR leopard OR tiger OR cheetah OR cougar OR puma OR ocelot OR lion OR jaguar OR bobcat OR "fishing cat" OR polecat OR oncilla OR margay OR ruminant OR livestock OR cattle OR cow OR yak OR bison OR gaur OR gayal OR buffalo OR sheep OR mouflon OR lamb OR ewe OR goat OR camel OR alpaca OR vicuna OR llama OR guanaco OR cria OR pig OR boar OR peccary OR donkey OR mule OR ass OR horse OR mustang OR pony OR zebra OR foal OR angulates OR herbivore OR wildlife OR antelope OR hippopotamus OR blackbuck OR bongo OR rhinoceros OR eland OR gazelle OR gemsbok OR oryx OR ibex OR impala OR kob OR kudu OR markhor OR marshbuck OR muskox OR nilgai OR nyala OR saiga OR springbok OR takin OR waterbuck OR wildebeest OR caribou OR cheetal OR elk OR moose OR deer OR sambar OR serow OR wapiti OR fawn OR giraffe OR okapi OR elephant OR beaver OR muskrat OR rat OR mouse OR porcupine OR chinchilla OR "guinea pig" OR cavy OR mara OR agouti OR paca OR degu OR "kangaroo rat" OR nutria OR hamster OR vole OR gerbile OR zokor OR dormouse OR squirrel OR chipmunk OR marmot OR grounhod OR "prairie dog" OR raccoon OR coati OR meerkat OR mongoose OR mink OR weasel OR marten OR badger OR otter OR ferret OR tayra OR bear OR civet OR genet OR bat OR quoll OR "tasmanian devil" OR monkey OR loris OR lemur OR galago OR baboon OR chimpanzee OR gorilla OR orangutan OR bandicoot OR sloth OR "giant anteater" OR walrus OR seal OR whale OR dolphin) AND 1970/01/01:2023/12/31[dp] |

**S1 Table.** (Continued)

| **Database: CAB Abstracts** |
| --- |
| (*Giardia* OR giardiasis OR giardiosis) AND (mammal OR dog OR canids OR dingo OR wolf OR coyote OR fox OR jackal OR cat OR felids OR caracal OR leopard OR tiger OR cheetah OR cougar OR puma OR ocelot OR lion OR jaguar OR bobcat OR polecat OR oncilla OR margay OR ruminant OR livestock OR cattle OR cow OR yak OR bison OR gaur OR gayal OR buffalo OR sheep OR mouflon OR lamb OR ewe OR goat OR camel OR alpaca OR vicuna OR llama OR guanaco OR cria OR pig OR boar OR peccary OR donkey OR mule OR ass OR horse OR mustang OR pony OR zebra OR foal OR angulates OR herbivore OR wildlife OR antelope OR hippopotamus OR blackbuck OR bongo OR rhinoceros OR eland OR gazelle OR gemsbok OR oryx OR ibex OR impala OR kob OR kudu OR markhor OR marshbuck OR muskox OR nilgai OR nyala OR saiga OR springbok OR takin OR waterbuck OR wildebeest OR deer OR caribou OR cheetal OR elk OR moose OR sambar OR serow OR wapiti OR fawn OR giraffe OR okapi OR elephant OR beaver OR muskrat OR rat OR mouse OR porcupine OR chinchilla OR cavy OR mara OR agouti OR paca OR degu OR nutria OR hamster OR vole OR gerbile OR zokor OR dormouse OR squirrel OR chipmunk OR marmot OR grounhod OR raccoon OR coati OR meerkat OR mongoose OR mink OR weasel OR marten OR badger OR otter OR ferret OR tayra OR bear OR civet OR genet OR bat OR quoll OR devil OR monkey OR loris OR lemur OR galago OR baboon OR chimpanzee OR gorilla OR orangutan OR bandicoot OR sloth OR walrus OR seal OR whale OR dolphin) AND yr:[1973 TO 2023] |

**S1 Table.** (Continued)

| **Database: Scopus** |
| --- |
| (ALL(Giard***) OR ALL(*Giardia*) OR ALL(giardiasis) OR ALL(giardiosis) OR ALL("*Giardia* infection")) AND (ALL(mammal) OR ALL(dog) OR ALL(canids) OR ALL(dingo) OR ALL(wolf) OR ALL(coyote) OR ALL(fox) OR ALL(jackal) OR ALL(cat) OR ALL(felids) OR ALL(caracal) OR ALL(leopard) OR ALL(tiger) OR ALL(cheetah) OR ALL(cougar) OR ALL(puma) OR ALL(ocelot) OR ALL(lion) OR ALL(jaguar) OR ALL(bobcat) OR ALL("fishing cat") OR ALL(polecat) OR ALL(oncilla) OR ALL(margay) OR ALL(ruminant) OR ALL(livestock) OR ALL(cattle) OR ALL(cow) OR ALL(yak) OR ALL(bison) OR ALL(gaur) OR ALL(gayal) OR ALL(buffalo) OR ALL(sheep) OR ALL(mouflon) OR ALL(lamb) OR ALL(ewe) OR ALL(goat) OR ALL(camel) OR ALL(alpaca) OR ALL(vicuna) OR ALL(llama) OR ALL(guanaco) OR ALL(cria) OR ALL(pig) OR ALL(boar) OR ALL(peccary) OR ALL(donkey) OR ALL(mule) OR ALL(ass) OR ALL(horse) OR ALL(mustang) OR ALL(pony) OR ALL(zebra) OR ALL(foal) OR ALL(angulates) OR ALL(herbivore) OR ALL(wildlife) OR ALL(antelope) OR ALL(hippopotamus) OR ALL(blackbuck) OR ALL(bongo) OR ALL(rhinoceros) OR ALL(eland) OR ALL(gazelle) OR ALL(gemsbok) OR ALL(oryx) OR ALL(ibex) OR ALL(impala) OR ALL(kob) OR ALL(kudu) OR ALL(markhor) OR ALL(marshbuck) OR ALL(muskox) OR ALL(nilgai) OR ALL(nyala) OR ALL(saiga) OR ALL(springbok) OR ALL(takin) OR ALL(waterbuck) OR ALL(wildebeest) OR ALL(caribou) OR ALL(cheetal) OR ALL(elk) OR ALL(moose) OR ALL(deer) OR ALL(sambar) OR ALL(serow) OR ALL(wapiti) OR ALL(fawn) OR ALL(giraffe) OR ALL(okapi) OR ALL(elephant) OR ALL(beaver) OR ALL(muskrat) OR ALL(rat) OR ALL(mouse) OR ALL(porcupine) OR ALL(chinchilla) OR ALL("guinea pig") OR ALL(cavy) OR ALL(mara) OR ALL(agouti) OR ALL(paca) OR ALL(degu) OR ALL("kangaroo rat") OR ALL(nutria) OR ALL(hamster) OR ALL(vole) OR ALL(gerbile) OR ALL(zokor) OR ALL(dormouse) OR ALL(squirrel) OR ALL(chipmunk) OR ALL(marmot) OR ALL(grounhod) OR ALL("prairie dog") OR ALL(raccoon) OR ALL(coati) OR ALL(meerkat) OR ALL(mongoose) OR ALL(mink) OR ALL(weasel) OR ALL(marten) OR ALL(badger) OR ALL(otter) OR ALL(ferret) OR ALL(tayra) OR ALL(bear) OR ALL(civet) OR ALL(genet) OR ALL(bat) OR ALL(quoll) OR ALL("tasmanian devil") OR ALL(monkey) OR ALL(loris) OR ALL(lemur) OR ALL(galago) OR ALL(baboon) OR ALL(chimpanzee) OR ALL(gorilla) OR ALL(orangutan) OR ALL(bandicoot) OR ALL(sloth) OR ALL("giant anteater") OR ALL(walrus) OR ALL(seal) OR ALL(whale) OR ALL(dolphin)) AND (PUBYEAR > 1969 AND PUBYEAR < 2023) |

**S1 Table.** (Continued)

| **Database: Web of Science** |
| --- |
| (ALL=(Giard***) OR ALL=(*Giardia*) OR ALL=(giardiasis) OR ALL=(giardiosis) OR ALL=("*Giardia* infection")) AND (ALL=(mammal) OR ALL=(dog) OR ALL=(canids) OR ALL=(dingo) OR ALL=(wolf) OR ALL=(coyote) OR ALL=(fox) OR ALL=(jackal) OR ALL=(cat) OR ALL=(felids) OR ALL=(caracal) OR ALL=(leopard) OR ALL=(tiger) OR ALL=(cheetah) OR ALL=(cougar) OR ALL=(puma) OR ALL=(ocelot) OR ALL=(lion) OR ALL=(jaguar) OR ALL=(bobcat) OR ALL=("fishing cat") OR ALL=(polecat) OR ALL=(oncilla) OR ALL=(margay) OR ALL=(ruminant) OR ALL=(livestock) OR ALL=(cattle) OR ALL=(cow) OR ALL=(yak) OR ALL=(bison) OR ALL=(gaur) OR ALL=(gayal) OR ALL=(buffalo) OR ALL=(sheep) OR ALL=(mouflon) OR ALL=(lamb) OR ALL=(ewe) OR ALL=(goat) OR ALL=(camel) OR ALL=(alpaca) OR ALL=(vicuna) OR ALL=(llama) OR ALL=(pig) OR ALL=(boar) OR ALL=(peccary) OR ALL=(donkey) OR ALL=(mule) OR ALL=(ass) OR ALL=(horse) OR ALL=(mustang) OR ALL=(pony) OR ALL=(zebra) OR ALL=(foal) OR ALL=(angulates) OR ALL=(herbivore) OR ALL=(wildlife) OR ALL=(antelope) OR ALL=(hippopotamus) OR ALL=(blackbuck) OR ALL=(bongo) OR ALL=(rhinoceros) OR ALL=(eland) OR ALL=(gazelle) OR ALL=(gemsbok) OR ALL=(oryx) OR ALL=(ibex) OR ALL=(impala) OR ALL=(kob) OR ALL=(kudu) OR ALL=(markhor) OR ALL=(marshbuck) OR ALL=(muskox) OR ALL=(nilgai) OR ALL=(nyala) OR ALL=(saiga) OR ALL=(springbok) OR ALL=(takin) OR ALL=(waterbuck) OR ALL=(wildebeest) OR ALL=(caribou) OR ALL=(cheetal) OR ALL=(elk) OR ALL=(moose) OR ALL=(deer) OR ALL=(sambar) OR ALL=(serow) OR ALL=(wapiti) OR ALL=(fawn) OR ALL=(giraffe) OR ALL=(okapi) OR ALL=(elephant) OR ALL=(beaver) OR ALL=(muskrat) OR ALL=(rat) OR ALL=(mouse) OR ALL=(porcupine) OR ALL=(chinchilla) OR ALL=("guinea pig") OR ALL=(cavy) OR ALL=(mara) OR ALL=(agouti) OR ALL=(paca) OR ALL=(degu) OR ALL=("kangaroo rat") OR ALL=(nutria) OR ALL=(hamster) OR ALL=(vole) OR ALL=(gerbile) OR ALL=(zokor) OR ALL=(dormouse) OR ALL=(squirrel) OR ALL=(chipmunk) OR ALL=(marmot) OR ALL=(grounhod) OR ALL=("prairie dog") OR ALL=(raccoon) OR ALL=(coati) OR ALL=(meerkat) OR ALL=(mongoose) OR ALL=(mink) OR ALL=(weasel) OR ALL=(marten) OR ALL=(badger) OR ALL=(otter) OR ALL=(ferret) OR ALL=(tayra) OR ALL=(bear) OR ALL=(civet) OR ALL=(genet) OR ALL=(bat) OR ALL=(quoll) OR ALL=("tasmanian devil") OR ALL=(monkey) OR ALL=(loris) OR ALL=(lemur) OR ALL=(galago) OR ALL=(baboon) OR ALL=(chimpanzee) OR ALL=(gorilla) OR ALL=(orangutan) OR ALL=(bandicoot) OR ALL=(sloth) OR ALL=("giant anteater") OR ALL=(walrus) OR ALL=(seal) OR ALL=(whale) OR ALL=(dolphin)) AND PY=1985-2023 |
