# Supplementary material for: Global prevalence of Giardia infection in nonhuman mammalian hosts: A systematic review and meta-analysis of five million animals
Source: PLoS Negl Trop Dis. 2025 Apr 24;19(4):e0013021. doi: 10.1371/journal.pntd.0013021 (PMC12052165; doi:10.1371/journal.pntd.0013021)
Supplement: S2 Table — The animals were sorted according to the taxonomic hierarchy, and then by the year of publication of the studies (n = 882). (DOC) [file pntd.0013021.s003.doc]

**S2 Table.** Worldwide prevalence of *Giardia* infection in non-human mammals. The animals were sorted according to the taxonomic hierarchy, and then by the year of publication of the studies (*n* = 882).

| **Host's scientific name (common name)** | **Country** | **Total (*n*)** | **Pos. (*n*)** | **Age group** | **Status** | **Detection method** | **Ref.** | **E** |
| --- | --- | --- | --- | --- | --- | --- | --- | --- |
| **Order Afrosoricida** |  |  |  |  |  |  |  |  |
| **Family Tenrecidae** |  |  |  |  |  |  |  |  |
| *Echinops telfairi* (lesser hedgehog tenrec) | Croatia | 1 | 0 | None | Captive | CADT | [1] | ❸ |
| **Order Artiodactyla** |  |  |  |  |  |  |  |  |
| **Family Bovidae** |  |  |  |  |  |  |  |  |
| **Subfamily Aepycerotinae** |  |  |  |  |  |  |  |  |
| *Aepyceros melampus* (impala or rooibok) | Poland | 3 | 0 | None | Captive | CM | [2] | ❸ |
| *Aepyceros melampus* (impala or rooibok) | Bangladesh | 2 | 0 | None | Captive | PCR | [3] | ❸ |
| **Subfamily Alcelaphinae** |  |  |  |  |  |  |  |  |
| *Connochaetes taurinus* (blue wildebeest) | Poland | 4 | 0 | None | Captive | CM | [2] | ❸ |
| *Connochaetes taurinus* (blue wildebeest) | Belgium | 2 | 0 | None | Captive | CADT | [4] | ❸ |
| *Connochaetes taurinus* (blue wildebeest) | Croatia | 1 | 0 | None | Captive | CADT | [1] | ❸ |
| *Connochaetes taurinus* (blue wildebeest) | Bangladesh | 2 | 0 | None | Captive | PCR | [3] | ❸ |
| *Connochaetes taurinus* (blue wildebeest) | China | 2 | 0 | None | Captive | PCR | [5] | ❸ |
| *Damaliscus pygargus* (bontebok) | China | 2 | 0 | None | Captive | PCR | [6] | ❸ |
| **Subfamily Antilopinae** |  |  |  |  |  |  |  |  |
| *Antidorcas marsupialis* (springbok) | Poland | 5 | 0 | None | Captive | CM | [2] |  |
| *Antilope cervicapra* (blackbuck) | Poland | 4 | 0 | None | Captive | CM | [2] | ❸ |
| *Gazella leptoceros* (slender-horned gazelle) | Belgium | 7 | 1 | None | Captive | CADT | [4] |  |
| **Subfamily Bovinae** |  |  |  |  |  |  |  |  |
| **Tribe Boselaphini** |  |  |  |  |  |  |  |  |
| *Boselaphus* *tragocamelus* (nilgai) | Poland | 2 | 0 | None | Captive | CM | [2] | ❸ |
| *Boselaphus* *tragocamelus* (nilgai) | Croatia | 1 | 0 | None | Captive | CADT | [1] | ❸ |
| **Tribe Bovini** |  |  |  |  |  |  |  |  |
| *Bison bison* (American buffalo) | Poland | 2 | 0 | None | Captive | CM | [2] | ❸ |
| *Bison bison* (American buffalo) | Canada | 41 | 6 | None | Free range | CADT | [7] |  |
| *Bison bison* (American buffalo) | Belgium | 82 | 19 | None | Farmed | CADT/PCR | [4] |  |
| *Bison bison* (American buffalo) | Belgium | 3 | 0 | None | Captive | CADT | [4] | ❸ |
| *Bison bison* (American buffalo) | Serbia | 4 | 0 | None | Captive | PCR | [8] | ❸ |
| *Bison bonasus* (European buffalo) | Poland | 16 | 2 | None | Captive | CADT | [9] |  |
| *Bison bonasus* (European buffalo) | Poland | 55 | 4 | None | Wild | CADT | [10] |  |
| *Bison bonasus* (European buffalo) | Belgium | 7 | 0 | None | Captive | CADT | [4] |  |
| *Bison bonasus* (European buffalo) | Croatia | 3 | 1 | None | Captive | CADT/PCR | [1] | ❸ |
| *Bos frontalis* (gayal) | Bangladesh | 4 | 0 | None | Captive | PCR | [3] | ❸ |
| *Bos grunniens* (domestic yak) | Poland | 2 | 0 | None | Captive | CM | [2] | ❸ |
| *Bos grunniens* (domestic yak) | China | 57 | 10 | None | Free range | CADT | [11] |  |
| *Bos grunniens* (domestic yak) | China | 545 | 16 | 2 groups | Free range | PCR | [12] |  |
| *Bos grunniens* (domestic yak) | China | 208 | 4 | None | Free range | PCR | [13] |  |
| *Bos grunniens* (domestic yak) | China | 605 | 63 | 3 groups | Free range | PCR | [14] |  |
| *Bos grunniens* (domestic yak) | China | 5 | 1 | None | Captive | PCR | [15] |  |
| *Bos grunniens* (domestic yak) | China | 297 | 18 | 4–7 m | Free range | CADT/PCR | [16] |  |
| *Bos grunniens* (domestic yak) | China | 344 | 20 | 1–2 m | Farmed | CADT/PCR | [17] |  |
| *Bos grunniens* (domestic yak) | China | 577 | 10 | None | Free range | PCR | [18] |  |
| *Bos grunniens* (domestic yak) | China | 101 | 6 | None | Free range | PCR | [19] |  |
| *Bos grunniens* (domestic yak) | China | 71 | 0 | None | Free range | PCR | [20] |  |
| *Bos grunniens* (domestic yak) | China | 1,027 | 21 | 2 groups | Free range | PCR | [21] |  |
| *Bos grunniens* (domestic yak) | China | 40 | 3 | None | Free range | PCR | [22] |  |
| *Bos grunniens* (domestic yak) | Serbia | 5 | 0 | None | Captive | PCR | [8] |  |
| *Bos grunniens* *mutus* (wild yak) | Belgium | 5 | 0 | None | Captive | CADT | [4] |  |
| *Bos grunniens* *mutus* (wild yak) | China | 3 | 0 | None | Captive | PCR | [5] | ❸ |
| *Bos javanicus* (banteng) | Poland | 1 | 0 | None | Captive | CM | [2] | ❸ |
| *Bos taurus* (domestic cattle) | India | 157 | 81 | < 1 yrs | Farmed | CM | [23] |  |
| *Bos taurus* (domestic cattle) | Switzerland | 815 | 217 | < 6 m | Farmed | CM | [24] |  |
| *Bos taurus* (domestic cattle) | Canada | 49 | 5 | 2 groups | Farmed | CM | [25] |  |
| *Bos taurus* (domestic cattle) | USA | 14 | 14 | < 6 m | Farmed | CADT | [26] | ❷ |
| *Bos taurus* (domestic cattle) | Spain | 554 | 65 | 4 groups | Farmed | CM | [27] |  |
| *Bos taurus* (domestic cattle) | Germany | 40 | 31 | None | Farmed | CADT | [28] |  |
| *Bos taurus* (domestic cattle) | Spain | 592 | 1 | None | Farmed | CM | [29] |  |
| *Bos taurus* (domestic cattle) | Brazil | 206 | 27 | None | Farmed | CM | [30] |  |
| *Bos taurus* (domestic cattle) | Denmark | 92 | 7 | None | Farmed | CM | [31] |  |
| *Bos taurus* (domestic cattle) | Canada | 104 | 30 | 2 groups | Farmed | CADT | [32] |  |
| *Bos taurus* (domestic cattle) | Canada | 386 | 283 | < 5 m | Farmed | CADT | [33] |  |
| *Bos taurus* (domestic cattle) | Greece | 100 | 0 | 2–5 yrs | Farmed | CM | [34] |  |
| *Bos taurus* (domestic cattle) | Poland | 35 | 5 | < 1 m | Farmed | CM | [35] |  |
| *Bos taurus* (domestic cattle) | Canada | 550 | 231 | 2–16 wk | Farmed | CM | [36] | ❺ |
| *Bos taurus* (domestic cattle) | USA | 557 | 36 | None | Farmed | CM | [37] |  |
| *Bos taurus* (domestic cattle) | Taiwan | 5 | 1 | None | Farmed | CADT | [38] | ❸ |
| *Bos taurus* (domestic cattle) | USA | 290 | 54 | < 6 m | Farmed | CM | [39] |  |
| *Bos taurus* (domestic cattle) | Canada | 20 | 20 | < 3 m | Farmed | CM | [40] | ❷ |
| *Bos taurus* (domestic cattle) | USA | 2,943 | 262 | 3 groups | Farmed | CM | [41] |  |
| *Bos taurus* (domestic cattle) | USA | 184 | 50 | 3 groups | Farmed | CM | [42] |  |
| *Bos taurus* (domestic cattle) | New Zeal. | 715 | 290 | < 8 wks | Farmed | CADT | [43] |  |
| *Bos taurus* (domestic cattle) | Australia | 36 | 21 | 2–10 wks | Farmed | CADT/PCR | [44] |  |
| *Bos taurus* (domestic cattle) | Canada | 28 | 16 | 2–10 wks | Farmed | CADT/PCR | [44] |  |
| *Bos taurus* (domestic cattle) | Uganda | 50 | 6 | 2 groups | Farmed | CADT | [45] |  |
| *Bos taurus* (domestic cattle) | Uganda | 50 | 5 | 2 groups | Farmed | CADT/PCR | [46] |  |
| *Bos taurus* (domestic cattle) | USA | 22 | 10 | 2 groups | Farmed | CADT | [47] |  |
| *Bos taurus* (domestic cattle) | Sweden | 270 | 71 | < 3 m | Farmed | CADT | [48] |  |
| *Bos taurus* (domestic cattle) | USA | 782 | 29 | None | Farmed | CM | [49] | ❺ |
| *Bos taurus* (domestic cattle) | New Zealand | 658 | 48 | 2 groups | Farmed | CADT/PCR | [50] |  |
| *Bos taurus* (domestic cattle) | Canada | 495 | 170 | 2–10 wks | Farmed | CADT/PCR | [51] |  |
| *Bos taurus* (domestic cattle) | Germany | 998 | 0 | None | Farmed | CM | [52] |  |
| *Bos taurus* (domestic cattle) | USA | 83 | 26 | < 1 yr | Farmed | CADT | [53] |  |
| *Bos taurus* (domestic cattle) | Belgium | 235 | 58 | < 2 m | Farmed | CADT | [54] |  |
| *Bos taurus* (domestic cattle) | USA | 513 | 224 | < 2 m | Farmed | CADT/PCR | [55] |  |
| *Bos taurus* (domestic cattle) | Italy | 10 | 3 | < 1 yr | Farmed | CM | [56] | ❸ |
| *Bos taurus* (domestic cattle) | Canada | 382 | 45 | 2–14 yrs | Farmed | CADT | [57] |  |
| *Bos taurus* (domestic cattle) | Canada | 192 | 69 | 2–70 d | Farmed | CADT | [57] |  |
| *Bos taurus* (domestic cattle) | Canada | 287 | 13 | 2–14 yrs | Farmed | CADT | [57] |  |
| *Bos taurus* (domestic cattle) | Turkey | 457 | 17 | 6 groups | Farmed | CM | [58] |  |
| *Bos taurus* (domestic cattle) | USA | 456 | 237 | 3–11 m | Farmed | PCR | [59] |  |
| *Bos taurus* (domestic cattle) | Tanzania | 942 | 3 | None | Farmed | CM | [60] |  |
| *Bos taurus* (domestic cattle) | Turkey | 231 | 34 | < 8 m | Farmed | CM | [61] |  |
| *Bos taurus* (domestic cattle) | Norway | 1,386 | 679 | < 6 m | Farmed | CADT | [62] |  |
| *Bos taurus* (domestic cattle) | USA | 571 | 204 | 1–2 yrs | Farmed | PCR | [63] |  |
| *Bos taurus* (domestic cattle) | Denmark | 1,150 | 501 | 3 groups | Farmed | CADT | [64] |  |
| *Bos taurus* (domestic cattle) | Canada | 30 | 11 | > 3 yrs | Farmed | CADT/PCR | [65] |  |
| *Bos taurus* (domestic cattle) | Canada | 1,165 | 232 | 2 groups | Farmed | CADT | [66] |  |
| *Bos taurus* (domestic cattle) | Austria | 230 | 14 | < 2 m | Farmed | PCR | [67] |  |
| *Bos taurus* (domestic cattle) | Iraq | 110 | 24 | None | Farmed | CM | [68] |  |
| *Bos taurus* (domestic cattle) | Spain | 734 | 221 | 12 groups | Farmed | CADT | [69] |  |
| *Bos taurus* (domestic cattle) | USA | 541 | 144 | > 2 yrs | Farmed | PCR | [70] |  |
| *Bos taurus* (domestic cattle) | Portugal | 467 | 42 | 2 groups | Farmed | CADT/PCR | [71] |  |
| *Bos taurus* (domestic cattle) | Spain | 379 | 101 | 3–13 yrs | Farmed | CADT/PCR | [72] |  |
| *Bos taurus* (domestic cattle) | Taiwan | 75 | 7 | None | Farmed | CADT/PCR | [73] |  |
| *Bos taurus* (domestic cattle) | Canada | 143 | 60 | 4 groups | Farmed | PCR | [74] |  |
| *Bos taurus* (domestic cattle) | Belgium | 832 | 260 | < 10 wks | Farmed | CADT/PCR | [75] |  |
| *Bos taurus* (domestic cattle) | New Zealand | 1,190 | 370 | 1–7 wks | Farmed | CADT/PCR | [76] |  |
| *Bos taurus* (domestic cattle) | Turkey | 182 | 17 | < 6 m | Farmed | CM | [77] |  |
| *Bos taurus* (domestic cattle) | New Zealand | 155 | 7 | None | Farmed | CADT | [78] |  |
| *Bos taurus* (domestic cattle) | Canada | 208 | 100 | < 6 m | Farmed | CADT | [79] |  |
| *Bos taurus* (domestic cattle) | USA | 189 | 22 | < 2 m | Farmed | CADT/PCR | [80] |  |
| *Bos taurus* (domestic cattle) | Vietnam | 68 | 34 | < 3 m | Farmed | CADT/PCR | [81] |  |
| *Bos taurus* (domestic cattle) | India | 187 | 24 | 5 groups | Farmed | CM | [82] |  |
| *Bos taurus* (domestic cattle) | USA | 990 | 312 | < 2 yrs | Farmed | PCR | [83] | ❷ |
| *Bos taurus* (domestic cattle) | Egypt | 182 | 56 | 2 groups | Farmed | CM | [84] |  |
| *Bos taurus* (domestic cattle) | USA | 5,260 | 1,006 | None | Farmed | CADT | [85] | ❺ |
| *Bos taurus* (domestic cattle) | Spain | 1,316 | 123 | 3 groups | Farmed | CADT | [86] |  |
| *Bos taurus* (domestic cattle) | Australia | 35 | 4 | None | Farmed | PCR | [87] |  |
| *Bos taurus* (domestic cattle) | Pakistan | 1,440 | 362 | 2 groups | Farmed | CM | [88] |  |
| *Bos taurus* (domestic cattle) | Belgium | 421 | 146 | < 9 m | Farmed | CADT | [89] |  |
| *Bos taurus* (domestic cattle) | Iran | 300 | 68 | 2 groups | Farmed | CM | [90] |  |
| *Bos taurus* (domestic cattle) | Kenya | 364 | 51 | None | Farmed | CM | [91] |  |
| *Bos taurus* (domestic cattle) | Uganda | 25 | 2 | None | Farmed | PCR | [92] |  |
| *Bos taurus* (domestic cattle) | Australia | 70 | 10 | None | Farmed | PCR | [93] |  |
| *Bos taurus* (domestic cattle) | Australia | 364 | 98 | < 4 m | Farmed | PCR | [94] |  |
| *Bos taurus* (domestic cattle) | Canada | 1,075 | 428 | 2 groups | Farmed | CADT/PCR | [95] |  |
| *Bos taurus* (domestic cattle) | Mexico | 174 | 9 | None | Farmed | CM/PCR | [96] |  |
| *Bos taurus* (domestic cattle) | Thailand | 247 | 0 | 2 groups | Farmed | CM | [97] |  |
| *Bos taurus* (domestic cattle) | Argentina | 620 | 214 | < 2 m | Farmed | CM | [98] |  |
| *Bos taurus* (domestic cattle) | Brazil | 254 | 96 | 3–10 m | Farmed | CM | [99] |  |
| *Bos taurus* (domestic cattle) | India | 180 | 22 | 3 groups | Farmed | CADT/PCR | [100] |  |
| *Bos taurus* (domestic cattle) | Spain | 227 | 17 | 4–88 m | Farmed | CADT | [101] |  |
| *Bos taurus* (domestic cattle) | Romania | 288 | 77 | 4–7 m | Farmed | CADT | [102] |  |
| *Bos taurus* (domestic cattle) | Brazil | 200 | 15 | 2 groups | Farmed | PCR | [103] |  |
| *Bos taurus* (domestic cattle) | Canada | 675 | 228 | 3 groups | Farmed | CADT/PCR | [104] |  |
| *Bos taurus* (domestic cattle) | Canada | 739 | 310 | 2 groups | Farmed | CADT/PCR | [105] |  |
| *Bos taurus* (domestic cattle) | Algeria | 634 | 88 | 4 groups | Farmed | CM | [106] |  |
| *Bos taurus* (domestic cattle) | USA | 2,109 | 504 | < 6 m | Farmed | CM/PCR | [107] |  |
| *Bos taurus* (domestic cattle) | USA | 819 | 274 | 6– m | Farmed | PCR | [108] |  |
| *Bos taurus* (domestic cattle) | USA | 201 | 69 | None | Farmed | CADT/PCR | [109] |  |
| *Bos taurus* (domestic cattle) | Malaysia | 240 | 30 | 2 groups | Farmed | PCR | [110] |  |
| *Bos taurus* (domestic cattle) | New Zealand | 180 | 11 | 2 groups | Farmed | PCR | [111] |  |
| *Bos taurus* (domestic cattle) | Colombia | 308 | 115 | < 2 m | Farmed | CM | [112] |  |
| *Bos taurus* (domestic cattle) | China | 814 | 42 | 4 groups | Farmed | CM/PCR | [113] |  |
| *Bos taurus* (domestic cattle) | Italy | 503 | 162 | 2–16 wks | Farmed | CADT/PCR | [114] |  |
| *Bos taurus* (domestic cattle) | France | 477 | 190 | 2–16 wks | Farmed | CADT/PCR | [114] |  |
| *Bos taurus* (domestic cattle) | Germany | 536 | 274 | 2–16 wks | Farmed | CADT/PCR | [114] |  |
| *Bos taurus* (domestic cattle) | UK | 556 | 305 | 2–16 wks | Farmed | CADT/PCR | [114] |  |
| *Bos taurus* (domestic cattle) | Romania | 204 | 69 | < 6 m | Farmed | CADT | [115] |  |
| *Bos taurus* (domestic cattle) | USA | 47 | 24 | 10 groups | Farmed | PCR | [116] |  |
| *Bos taurus* (domestic cattle) | China | 249 | 19 | None | Farmed | PCR | [117] |  |
| *Bos taurus* (domestic cattle) | Ethiopia | 384 | 9 | None | Farmed | CM | [118] |  |
| *Bos taurus* (domestic cattle) | Brazil | 6 | 1 | < 6 m | Farmed | CM | [119] | ❸ |
| *Bos taurus* (domestic cattle) | Nigeria | 224 | 56 | None | Farmed | CM | [120] |  |
| *Bos taurus* (domestic cattle) | Romania | 621 | 239 | < 6 m | Farmed | CADT | [121] |  |
| *Bos taurus* (domestic cattle) | Brazil | 256 | 30 | < 10 m | Farmed | CM/PCR | [122] |  |
| *Bos taurus* (domestic cattle) | Brazil | 100 | 9 | 2 groups | Farmed | CADT | [123] |  |
| *Bos taurus* (domestic cattle) | Brazil | 118 | 94 | < 3 m | Farmed | CM/PCR | [124] |  |
| *Bos taurus* (domestic cattle) | USA | 16 | 0 | None | Fair | CADT | [125] |  |
| *Bos taurus* (domestic cattle) | UK | 283 | 93 | None | Farmed | PCR | [126] |  |
| *Bos taurus* (domestic cattle) | China | 1,777 | 128 | 6 groups | Farmed | CM/PCR | [127] |  |
| *Bos taurus* (domestic cattle) | Algeria | 690 | 92 | 4 groups | Farmed | CM | [128] |  |
| *Bos taurus* (domestic cattle) | Rwanda | 135 | 8 | 2 groups | Farmed | CADT/PCR | [129] |  |
| *Bos taurus* (domestic cattle) | Egypt | 593 | 40 | 4 groups | Farmed | CADT/PCR | [130] |  |
| *Bos taurus* (domestic cattle) | China | 1,366 | 29 | 4 groups | Farmed | CM/PCR | [131] |  |
| *Bos taurus* (domestic cattle) | China | 47 | 5 | None | Farmed | CADT | [11] |  |
| *Bos taurus* (domestic cattle) | Indonesia | 394 | 0 | None | Farmed | CM | [132] |  |
| *Bos taurus* (domestic cattle) | China | 622 | 21 | None | Farmed | CM/PCR | [133] |  |
| *Bos taurus* (domestic cattle) | Germany | 1,564 | 112 | < 1 yr | Farmed | CM/PCR | [134] |  |
| *Bos taurus* (domestic cattle) | Sri Lanka | 340 | 141 | < 3 m | Farmed | PCR | [135] |  |
| *Bos taurus* (domestic cattle) | Iraq | 1,049 | 322 | None | Farmed | CM | [136] |  |
| *Bos taurus* (domestic cattle) | Moldavia | 140 | 63 | < 5 m | Farmed | CADT | [137] |  |
| *Bos taurus* (domestic cattle) | India | 120 | 39 | < 1 yr | Farmed | CADT | [138] |  |
| *Bos taurus* (domestic cattle) | Bangladesh | 625 | 136 | < 6 m | Farmed | CADT/PCR | [139] |  |
| *Bos taurus* (domestic cattle) | Spain | 362 | 68 | 2–11 yrs | Farmed | PCR | [140] |  |
| *Bos taurus* (domestic cattle) | China | 655 | 52 | 3 groups | Farmed | CM/PCR | [141] |  |
| *Bos taurus* (domestic cattle) | India | 1,176 | 211 | 2 groups | Farmed | CM | [142] |  |
| *Bos taurus* (domestic cattle) | Kenya | 110 | 0 | 6 wks | Farmed | CM | [143] |  |
| *Bos taurus* (domestic cattle) | Thailand | 900 | 54 | None | Farmed | PCR | [144] |  |
| *Bos taurus* (domestic cattle) | India | 20 | 8 | None | Farmed | CADT | [145] | ❺ |
| *Bos taurus* (domestic cattle) | Poland | 86 | 16 | None | Farmed | CADT/PCR | [146] |  |
| *Bos taurus* (domestic cattle) | Romania | 28 | 3 | < 8 m | Farmed | CM/PCR | [147] |  |
| *Bos taurus* (domestic cattle) | China | 514 | 69 | 2 groups | Farmed | PCR | [148] |  |
| *Bos taurus* (domestic cattle) | Egypt | 62 | 18 | 1–3 m | Farmed | CM | [149] |  |
| *Bos taurus* (domestic cattle) | Iraq | 50 | 35 | None | Farmed | CM/PCR | [150] |  |
| *Bos taurus* (domestic cattle) | Zambia | 377 | 130 | 3 groups | Farmed | CADT | [151] |  |
| *Bos taurus* (domestic cattle) | South Korea | 590 | 78 | < 2 m | Farmed | CADT/PCR | [152] |  |
| *Bos taurus* (domestic cattle) | China | 507 | 48 | None | Farmed | PCR | [153] |  |
| *Bos taurus* (domestic cattle) | China | 622 | 21 | None | Farmed | PCR | [154] |  |
| *Bos taurus* (domestic cattle) | Ethiopia | 220 | 18 | 3 groups | Farmed | PCR | [155] |  |
| *Bos taurus* (domestic cattle) | China | 822 | 14 | 3 groups | Farmed | CM/PCR | [156] |  |
| *Bos taurus* (domestic cattle) | Iran | 61 | 8 | < 3 m | Farmed | CM | [157] |  |
| *Bos taurus* (domestic cattle) | Vietnam | 412 | 57 | 2 groups | Farmed | CM | [158] |  |
| *Bos taurus* (domestic cattle) | Australia | 175 | 64 | None | Farmed | PCR | [159] |  |
| *Bos taurus* (domestic cattle) | China | 371 | 70 | 2 groups | Farmed | PCR | [160] |  |
| *Bos taurus* (domestic cattle) | China | 2,945 | 107 | 6 groups | Farmed | PCR | [161] |  |
| *Bos taurus* (domestic cattle) | Germany | 1,416 | 0 | None | Farmed | CM | [162] |  |
| *Bos taurus* (domestic cattle) | Algeria | 102 | 28 | < 3 m | Farmed | PCR | [163] |  |
| *Bos taurus* (domestic cattle) | Ghana | 328 | 28 | None | Farmed | PCR | [164] |  |
| *Bos taurus* (domestic cattle) | Brazil | 243 | 65 | < 2 m | Farmed | CM | [165] |  |
| *Bos taurus* (domestic cattle) | Uganda | 45 | 2 | None | Farmed | PCR | [166] |  |
| *Bos taurus* (domestic cattle) | China | 818 | 492 | < 2 m | Farmed | PCR | [167] |  |
| *Bos taurus* (domestic cattle) | China | 339 | 77 | 2 groups | Farmed | PCR | [168] |  |
| *Bos taurus* (domestic cattle) | China | 1,040 | 49 | 3 groups | Farmed | PCR | [169] |  |
| *Bos taurus* (domestic cattle) | Turkey | 198 | 35 | < 3 m | Farmed | CM | [170] |  |
| *Bos taurus* (domestic cattle) | Nepal | 96 | 43 | 4 groups | Farmed | CM | [171] |  |
| *Bos taurus* (domestic cattle) | USA | 2,249 | 686 | < 2 m | Farmed | CADT | [172] |  |
| *Bos taurus* (domestic cattle) | Iran | 246 | 23 | 2 groups | Farmed | CM/PCR | [173] |  |
| *Bos taurus* (domestic cattle) | China | 389 | 39 | < 1 yr | Free range | PCR | [174] |  |
| *Bos taurus* (domestic cattle) | Egypt | 248 | 33 | 6 groups | Farmed | PCR | [175] |  |
| *Bos taurus* (domestic cattle) | China | 1,440 | 31 | 4 groups | Farmed | PCR | [176] |  |
| *Bos taurus* (domestic cattle) | China | 278 | 26 | < 2 m | Farmed | PCR | [177] |  |
| *Bos taurus* (domestic cattle) | Brazil | 197 | 4 | None | Farmed | CM | [178] |  |
| *Bos taurus* (domestic cattle) | South Korea | 90 | 9 | < 2 m | Farmed | PCR | [179] |  |
| *Bos taurus* (domestic cattle) | Turkey | 100 | 4 | < 6 m | Farmed | CADT | [180] |  |
| *Bos taurus* (domestic cattle) | Ghana | 320 | 17 | 3 groups | Farmed | CM | [181] |  |
| *Bos taurus* (domestic cattle) | USA | 1,412 | 454 | 2 groups | Farmed | PCR | [182] |  |
| *Bos taurus* (domestic cattle) | Mozambique | 480 | 39 | < 7 m | Farmed | CADT | [183] |  |
| *Bos taurus* (domestic cattle) | South Korea | 207 | 23 | < 2 m | Farmed | PCR | [184] |  |
| *Bos taurus* (domestic cattle) | China | 306 | 126 | 3–11 m | Farmed | PCR | [185] |  |
| *Bos taurus* (domestic cattle) | Scotland | 388 | 126 | 3 groups | Farmed | PCR | [186] |  |
| *Bos taurus* (domestic cattle) | Indonesia | 500 | 0 | None | Farmed | CM | [187] |  |
| *Bos taurus* (domestic cattle) | Iran | 192 | 8 | 3 groups | Farmed | PCR | [188] |  |
| *Bos taurus* (domestic cattle) | Indonesia | 109 | 9 | > 2 yrs | Farmed | CM/PCR | [189] |  |
| *Bos taurus* (domestic cattle) | China | 1,366 | 281 | 3 groups | Farmed | PCR | [190] |  |
| *Bos taurus* (domestic cattle) | China | 388 | 288 | < 8 wks | Farmed | PCR | [191] |  |
| *Bos taurus* (domestic cattle) | Austria | 177 | 48 | < 6 m | Farmed | CADT | [192] |  |
| *Bos taurus* (domestic cattle) | South Korea | 100 | 13 | 3 groups | Farmed | PCR | [193] |  |
| *Bos taurus* (domestic cattle) | Indonesia | 100 | 7 | 4 groups | Farmed | CM | [194] |  |
| *Bos taurus* (domestic cattle) | Philippines | 47 | 5 | None | Farmed | CM | [195] |  |
| *Bos taurus* (domestic cattle) | South Korea | 315 | 40 | < 8 wks | Farmed | PCR | [196] |  |
| *Bos taurus* (domestic cattle) | Turkey | 71 | 46 | < 1 yr | Farmed | PCR | [197] |  |
| *Bos taurus* (domestic cattle) | China | 57 | 3 | None | Farmed | PCR | [19] |  |
| *Bos taurus* (domestic cattle) | Ethiopia | 330 | 32 | 3 groups | Farmed | CADT | [198] |  |
| *Bos taurus* (domestic cattle) | Turkey | 450 | 136 | 3 groups | Farmed | PCR | [199] |  |
| *Bos taurus* (domestic cattle) | Iraq | 270 | 62 | 3 groups | Farmed | CM | [200] |  |
| *Bos taurus* (domestic cattle) | China | 448 | 51 | None | Farmed | PCR | [201] |  |
| *Bos taurus* (domestic cattle) | Iraq | 50 | 14 | 3 groups | Farmed | CM | [202] |  |
| *Bos taurus* (domestic cattle) | Pakistan | 614 | 43 | 3 groups | Farmed | CM | [203] |  |
| *Bos taurus* (domestic cattle) | Iraq | 100 | 44 | 3 groups | Farmed | CM/PCR | [204] |  |
| *Bos taurus* (domestic cattle) | Colombia | 59 | 0 | None | Farmed | CM | [205] |  |
| *Bos taurus* (domestic cattle) | Australia | 811 | 225 | None | Farmed | PCR | [206] |  |
| *Bos taurus* (domestic cattle) | China | 1,414 | 14 | 4 groups | Farmed | PCR | [207] |  |
| *Bos taurus* (domestic cattle) | China | 442 | 17 | None | Free range | PCR | [20] |  |
| *Bos taurus* (domestic cattle) | Iraq | 100 | 56 | None | Farmed | CM | [208] |  |
| *Bos taurus* (domestic cattle) | Vietnam | 74 | 11 | None | Farmed | PCR | [209] |  |
| *Bos taurus* (domestic cattle) | Uganda | 11 | 1 | None | Farmed | CADT/PCR | [210] | ❸ |
| *Bos taurus* (domestic cattle) | China | 321 | 16 | < 1 yr | Farmed | PCR | [211] |  |
| *Bos taurus* (domestic cattle) | Argentina | 43 | 18 | None | Farmed | PCR | [212] |  |
| *Bos taurus* (domestic cattle) | Taiwan | 156 | 31 | None | Free range | CM/PCR | [213] |  |
| *Bos taurus* (domestic cattle) | Bangladesh | 870 | 0 | None | Farmed | CM | [214] |  |
| *Bos taurus* (domestic cattle) | China | 391 | 41 | 2 groups | Farmed | PCR | [215] |  |
| *Bos taurus* (domestic cattle) | Egypt | 51 | 27 | None | Farmed | CM | [216] |  |
| *Bos taurus* (domestic cattle) | Ecuador | 45 | 9 | None | Farmed | CM | [217] |  |
| *Bos taurus* (domestic cattle) | UAE | 11 | 3 | None | Farmed | PCR | [218] | ❸ |
| *Bos taurus* (domestic cattle) | South Korea | 792 | 44 | 2 groups | Farmed | PCR | [219] |  |
| *Bos taurus* (domestic cattle) | China | 69 | 13 | < 8 wks | Farmed | CADT | [220] |  |
| *Bos taurus* (domestic cattle) | USA | 2,539 | 1,013 | < 8 wks | Farmed | PCR | [221] |  |
| *Bos taurus* (domestic cattle) | Ethiopia | 208 | 81 | < 8 wks | Farmed | CADT | [222] |  |
| *Bos taurus* (domestic cattle) | China | 108 | 10 | 2 groups | Farmed | PCR | [223] |  |
| *Bos taurus* (domestic cattle) | China | 524 | 144 | 4 groups | Farmed | PCR | [224] |  |
| *Bos taurus* (domestic cattle) | China | 1 | 0 | None | Captive | PCR | [6] | ❸ |
| *Bos taurus* (domestic cattle) | China | 138 | 47 | None | Farmed | PCR | [225] |  |
| *Bos taurus* (domestic cattle) | Pakistan | 92 | 22 | 4 groups | Farmed | CADT | [226] |  |
| *Bos taurus* (domestic cattle) | Turkey | 100 | 9 | 2 groups | Farmed | CM/PCR | [227] |  |
| *Bos taurus* (domestic cattle) | Bangladesh | 699 | 40 | 3 groups | Farmed | PCR | [228] |  |
| *Bos taurus* (domestic cattle) | Iran | 88 | 0 | 2 groups | Farmed | CM | [229] |  |
| *Bos taurus* (domestic cattle) | China | 505 | 149 | 4 groups | Farmed | PCR | [230] |  |
| *Bos taurus* (domestic cattle) | South Korea | 455 | 20 | < 1 yr | Farmed | PCR | [231] |  |
| *Bos taurus* (domestic cattle) | China | 438 | 74 | None | Farmed | PCR | [232] |  |
| *Bos taurus* (domestic cattle) | Iraq | 100 | 34 | < 1 yr | Farmed | PCR | [233] |  |
| *Bos taurus* (domestic cattle) | Turkey | 50 | 3 | None | Farmed | CADT | [234] |  |
| *Bos taurus* (domestic cattle) | Portugal | 87 | 13 | None | Farmed | PCR | [235] |  |
| *Bos taurus indicus* (zebu cattle) | Poland | 2 | 0 | None | Captive | CM | [2] | ❸ |
| *Bos taurus indicus* (zebu cattle) | Tanzania | 19 | 4 | None | Farmed | PCR | [236] |  |
| *Bos taurus indicus* (zebu cattle) | Myanmar | 400 | 90 | 3 groups | Free range | CM | [237] |  |
| *Bos taurus indicus* (zebu cattle) | Ethiopia | 229 | 25 | 3 groups | Farmed | PCR | [155] |  |
| *Bos taurus indicus* (zebu cattle) | Brazil | 937 | 71 | 6 groups | Farmed | CM | [238] |  |
| *Bos taurus indicus* (zebu cattle) | India | 14 | 9 | None | Farmed | CADT/PCR | [239] |  |
| *Bos taurus indicus* (zebu cattle) | Madagascar | 41 | 0 | None | Farmed | CADT | [240] |  |
| *Bubalus bubalis* (water buffalo) | Greece | 70 | 70 | 2–5 yrs | Farmed | CM | [34] |  |
| *Bubalus bubalis* (water buffalo) | Brazil | 106 | 5 | 3–45 d | Farmed | CM | [241] |  |
| *Bubalus bubalis* (water buffalo) | Pakistan | 300 | 99 | 3 groups | Farmed | CM | [242] |  |
| *Bubalus bubalis* (water buffalo) | Italy | 57 | 15 | < 2 m | Farmed | CADT/PCR | [243] |  |
| *Bubalus bubalis* (water buffalo) | Italy | 347 | 63 | 1–9 wks | Farmed | CADT | [244] |  |
| *Bubalus bubalis* (water buffalo) | Pakistan | 720 | 188 | > 2 yrs | Farmed | PCR | [88] |  |
| *Bubalus bubalis* (water buffalo) | Australia | 476 | 62 | 3 groups | Farmed | PCR | [245] |  |
| *Bubalus bubalis* (water buffalo) | Egypt | 211 | 10 | 2 groups | Farmed | CADT/PCR | [130] |  |
| *Bubalus bubalis* (water buffalo) | Sri Lanka | 297 | 2 | 2 groups | Farmed | PCR | [135] |  |
| *Bubalus bubalis* (water buffalo) | Thailand | 567 | 2 | 3 groups | Farmed | PCR | [246] |  |
| *Bubalus bubalis* (water buffalo) | India | 22 | 2 | None | Farmed | CADT | [145] | ❺ |
| *Bubalus bubalis* (water buffalo) | Egypt | 100 | 25 | 1–4 m | Farmed | PCR | [247] |  |
| *Bubalus bubalis* (water buffalo) | Iran | 317 | 0 | < 9 m | Farmed | CM | [248] |  |
| *Bubalus bubalis* (water buffalo) | India | 83 | 9 | None | Farmed | CADT/PCR | [249] |  |
| *Bubalus bubalis* (water buffalo) | Brazil | 183 | 12 | 2 groups | Farmed | PCR | [250] |  |
| *Bubalus bubalis* (water buffalo) | Philippines | 12 | 1 | None | Farmed | CM | [195] |  |
| *Bubalus bubalis* (water buffalo) | Australia | 313 | 14 | None | Free range | PCR | [251] |  |
| *Bubalus bubalis* (water buffalo) | China | 108 | 1 | None | Farmed | PCR | [201] |  |
| *Bubalus bubalis* (water buffalo) | Vietnam | 17 | 3 | None | Farmed | PCR | [209] |  |
| *Bubalus bubalis* (water buffalo) | Romania | 38 | 1 | 2–11 wks | Farmed | PCR | [252] |  |
| *Bubalus bubalis* (water buffalo) | Nepal | 300 | 28 | 2 groups | Captive | CM | [253] |  |
| *Bubalus bubalis* (water buffalo) | Turkey | 100 | 11 | 4 groups | Farmed | PCR | [254] |  |
| *Bubalus bubalis* (water buffalo) | Iraq | 60 | 15 | None | Farmed | PCR | [255] |  |
| *Bubalus bubalis* (water buffalo) | Romania | 63 | 9 | 3–20 wks | Farmed | CM | [256] |  |
| *Syncerus caffer* (African buffalo) | Belgium | 1 | 0 | None | Captive | CADT | [4] | ❸ |
| *Syncerus caffer* (African buffalo) | Croatia | 4 | 1 | None | Captive | CADT/PCR | [1] | ❸ |
| *Syncerus caffer* (African buffalo) | Cen. Af. Rep. | 20 | 0 | None | Wild | PCR | [257] |  |
| *Syncerus caffer* (African buffalo) | Rwanda | 55 | 1 | None | Captive | PCR | [129] |  |
| **Tribe Tragelaphini** |  |  |  |  |  |  |  |  |
| *Taurotragus oryx* (common eland) | Japan | 1 | 0 | None | Captive | CADT | [258] | ❸ |
| *Taurotragus oryx* (common eland) | Belgium | 3 | 0 | None | Captive | CADT | [4] | ❸ |
| *Taurotragus oryx* (common eland) | Bangladesh | 2 | 0 | None | Captive | PCR | [3] | ❸ |
| *Tragelaphus euryceros* (bongo) | Belgium | 1 | 0 | None | Captive | CADT | [4] | ❸ |
| *Tragelaphus euryceros* (bongo) | Cen. Af. Rep. | 6 | 0 | None | Wild | PCR | [257] |  |
| *Tragelaphus spekii* (sitatunga) | Poland | 2 | 0 | None | Captive | CM | [2] | ❸ |
| *Tragelaphus spekii* (sitatunga) | Belgium | 5 | 0 | None | Captive | CADT | [4] | ❸ |
| *Tragelaphus strepsiceros* (greater kudu) | Poland | 3 | 0 | None | Captive | CM | [2] | ❸ |
| *Tragelaphus strepsiceros* (greater kudu) | Bangladesh | 1 | 0 | None | Captive | PCR | [3] | ❸ |
| **Subfalimy Caprinae** |  |  |  |  |  |  |  |  |
| **Tribe Caprini** |  |  |  |  |  |  |  |  |
| *Ammotragus lervia* (barbary sheep) | Japan | 2 | 0 | None | Captive | CADT | [258] | ❸ |
| *Ammotragus lervia* (barbary sheep) | Spain | 20 | 0 | None | Wild | PCR | [259] |  |
| *Budorcas taxicolor bedfordi* (golden takin) | China | 191 | 17 | None | Captive | PCR | [260] |  |
| *Budorcas taxicolor bedfordi* (golden takin) | China | 1 | 0 | None | Captive | PCR | [15] | ❸ |
| *Budorcas taxicolor* (takin) | China | 2 | 0 | None | Captive | PCR | [6] | ❸ |
| *Capra aegagrus hircus* (wild goat) | Cen. Af. Rep. | 9 | 1 | None | Wild | PCR | [257] |  |
| *Capra hircus* (domestic goat) | Switzerland | 20 | 4 | ≤ 6 m | Farmed | CM | [24] |  |
| *Capra hircus* (domestic goat) | Spain | 574 | 23 | None | Farmed | CM | [29] |  |
| *Capra hircus* (domestic goat) | Greece | 147 | 73 | 1–5 yrs | Farmed | CM | [34] |  |
| *Capra hircus* (domestic goat) | Japan | 6 | 0 | None | Captive | CADT | [258] |  |
| *Capra hircus* (domestic goat) | France | 100 | 13 | > 2 yrs | Farmed | CADT | [261] |  |
| *Capra hircus* (domestic goat) | Spain | 116 | 23 | None | Farmed | CADT/PCR | [72] |  |
| *Capra hircus* (domestic goat) | Spain | 315 | 134 | < 6 m | Farmed | CADT/PCR | [262] |  |
| *Capra hircus* (domestic goat) | Belgium | 148 | 53 | < 3 m | Farmed | CADT | [263] |  |
| *Capra hircus* (domestic goat) | Uganda | 57 | 8 | None | Farmed | PCR | [92] |  |
| *Capra hircus* (domestic goat) | Australia | 4 | 1 | None | Wild | PCR | [93] | ❸ |
| *Capra hircus* (domestic goat) | Côte d’Ivoire | 2 | 1 | None | Farmed | PCR | [264] | ❸ |
| *Capra hircus* (domestic goat) | China | 139 | 4 | 4 groups | Farmed | CM/PCR | [265] |  |
| *Capra hircus* (domestic goat) | Brazil | 5 | 1 | None | Farmed | CM | [119] |  |
| *Capra hircus* (domestic goat) | Malaysia | 310 | 21 | None | Farmed | PCR | [266] |  |
| *Capra hircus* (domestic goat) | USA | 11 | 0 | None | Fair | CADT | [125] |  |
| *Capra hircus* (domestic goat) | UK | 9 | 1 | None | Farmed | PCR | [126] |  |
| *Capra hircus* (domestic goat) | Iran | 94 | 15 | < 1 yr | Farmed | CM/PCR | [267] |  |
| *Capra hircus* (domestic goat) | China | 51 | 11 | None | Farmed | CADT | [11] |  |
| *Capra hircus* (domestic goat) | Tanzania | 41 | 9 | None | Farmed | PCR | [236] |  |
| *Capra hircus* (domestic goat) | Brazil | 58 | 17 | < 1 yr | Farmed | CM/PCR | [268] |  |
| *Capra hircus* (domestic goat) | China | 506 | 32 | None | Farmed | CM/PCR | [269] |  |
| *Capra hircus* (domestic goat) | India | 20 | 3 | None | Farmed | CADT | [145] | ❺ |
| *Capra hircus* (domestic goat) | Bangladesh | 100 | 3 | < 6 m | Farmed | PCR | [270] |  |
| *Capra hircus* (domestic goat) | Nigeria | 98 | 46 | 3 groups | Farmed | CM | [271] |  |
| *Capra hircus* (domestic goat) | India | 207 | 70 | None | Farmed | CADT/PCR | [272] |  |
| *Capra hircus* (domestic goat) | Ghana | 285 | 35 | None | Farmed | PCR | [164] |  |
| *Capra hircus* (domestic goat) | Germany | 98 | 0 | None | Farmed | CM | [162] |  |
| *Capra hircus* (domestic goat) | China | 907 | 38 | 2 groups | Farmed | PCR | [273] |  |
| *Capra hircus* (domestic goat) | China | 342 | 51 | > 2 yrs | Farmed | PCR | [274] |  |
| *Capra hircus* (domestic goat) | China | 336 | 16 | 4 groups | Free range | PCR | [275] |  |
| *Capra hircus* (domestic goat) | Turkey | 66 | 24 | 2 groups | Farmed | PCR | [197] |  |
| *Capra hircus* (domestic goat) | Iran | 100 | 5 | 3 groups | Farmed | PCR | [188] |  |
| *Capra hircus* (domestic goat) | China | 260 | 0 | None | Free range | PCR | [276] |  |
| *Capra hircus* (domestic goat) | Nigeria | 302 | 138 | 2 groups | Farmed | CM/PCR | [277] |  |
| *Capra hircus* (domestic goat) | Mozambique | 60 | 8 | < 6 m | Farmed | CADT | [183] |  |
| *Capra hircus* (domestic goat) | China | 59 | 1 | None | Free range | PCR | [19] |  |
| *Capra hircus* (domestic goat) | Iran | 150 | 15 | None | Farmed | CM | [278] |  |
| *Capra hircus* (domestic goat) | Bangladesh | 751 | 1 | None | Farmed | CM | [214] |  |
| *Capra hircus* (domestic goat) | Egypt | 32 | 12 | None | Farmed | CM | [216] |  |
| *Capra hircus* (domestic goat) | UAE | 6 | 0 | None | Captive | PCR | [218] | ❸ |
| *Capra hircus* (domestic goat) | Uganda | 11 | 0 | None | Farmed | CADT | [210] | ❸ |
| *Capra hircus* (domestic goat) | Iraq | 100 | 54 | None | Farmed | CM | [208] |  |
| *Capra hircus* (domestic goat) | China | 4 | 0 | None | Captive | PCR | [5] | ❸ |
| *Capra hircus* (domestic goat) | China | 561 | 21 | 2 groups | Free range | PCR | [223] |  |
| *Capra hircus* (domestic goat) | Ethiopia | 250 | 53 | 2 groups | Farmed | PCR | [222] |  |
| *Capra hircus* (domestic goat) | China | 352 | 68 | None | Farmed | PCR | [279] |  |
| *Capra hircus* (domestic goat) | Nigeria | 150 | 13 | None | Farmed | CM | [280] |  |
| *Capra hircus* (domestic goat) | Iran | 23 | 1 | 2 groups | Farmed | CM | [229] |  |
| *Capra hircus* (domestic goat) | China | 202 | 33 | < 3 m | Farmed | PCR | [281] |  |
| *Capra hircus* (domestic goat) | China | 226 | 56 | None | Farmed | PCR | [282] |  |
| *Capra ibex* (alpine ibex) | Belgium | 3 | 0 | ≤ 6 m | Captive | CADT | [4] | ❸ |
| *Capra pyrenaica* (Spanish ibex) | Spain | 89 | 8 | None | Wild | PCR | [259] |  |
| *Ovibos muschatus* (muskox) | Canada | 72 | 15 | None | Wild | CADT/PCR | [283] |  |
| *Ovibos muschatus* (muskox) | Greenland | 154 | 7 | 5 groups | Wild | PCR | [284] |  |
| *Ovis ammon* (argali) | Cen. Af. Rep. | 2 | 0 | None | Wild | PCR | [257] | ❸ |
| *Ovis ammon* (argali) | China | 2 | 0 | None | Captive | PCR | [5] | ❸ |
| *Ovis aries* (domestic sheep) | Switzerland | 382 | 114 | ≤ 6 m | Farmed | CM | [24] |  |
| *Ovis aries* (domestic sheep) | Canada | 205 | 36 | 2 groups | Farmed | CM | [25] |  |
| *Ovis aries* (domestic sheep) | Spain | 1,165 | 73 | None | Farmed | CM | [29] |  |
| *Ovis aries* (domestic sheep) | Canada | 62 | 25 | 2 groups | Farmed | CADT | [32] |  |
| *Ovis aries* (domestic sheep) | Greece | 273 | 91 | 2–5 yrs | Farmed | CM | [34] |  |
| *Ovis aries* (domestic sheep) | Taiwan | 2 | 0 | None | Watershed | CADT | [38] | ❸ |
| *Ovis aries* (domestic sheep) | Japan | 2 | 0 | None | Captive | CADT | [258] | ❸ |
| *Ovis aries* (domestic sheep) | Australia | 500 | 220 | > 8 wks | Farmed | PCR | [285] |  |
| *Ovis aries* (domestic sheep) | Italy | 325 | 5 | 2 groups | Farmed | CM/PCR | [286] |  |
| *Ovis aries* (domestic sheep) | Taiwan | 6 | 1 | None | Farmed | CADT | [73] | ❸ |
| *Ovis aries* (domestic sheep) | Spain | 446 | 86 | None | Farmed | CADT/PCR | [72] |  |
| *Ovis aries* (domestic sheep) | USA | 63 | 16 | 2 groups | Farmed | PCR | [287] |  |
| *Ovis aries* (domestic sheep) | Belgium | 137 | 36 | < 3 m | Farmed | CADT/PCR | [263] |  |
| *Ovis aries* (soay sheep) | Belgium | 5 | 1 | ≤ 6 m | Captive | CADT | [4] | ❸ |
| *Ovis aries* (domestic sheep) | Turkey | 132 | 64 | < 2 m | Farmed | CM | [288] |  |
| *Ovis aries* (domestic sheep) | Australia | 477 | 53 | < 8 wks | Farmed | PCR | [289] |  |
| *Ovis aries* (domestic sheep) | Australia | 284 | 43 | < 2 m | Farmed | PCR | [290] |  |
| *Ovis aries* (domestic sheep) | Uganda | 7 | 1 | None | Farmed | PCR | [92] | ❸ |
| *Ovis aries* (domestic sheep) | Australia | 2 | 0 | None | Wild | PCR | [93] | ❸ |
| *Ovis aries* (domestic sheep) | Mexico | 265 | 30 | None | Farmed | CM | [96] |  |
| *Ovis aries* (domestic sheep) | Spain | 120 | 107 | 1–3 m | Farmed | PCR | [291] |  |
| *Ovis aries* (domestic sheep) | China | 539 | 30 | 4 groups | Farmed | CM/PCR | [265] |  |
| *Ovis aries* (domestic sheep) | USA | 35 | 0 | None | Fair | CADT | [125] |  |
| *Ovis aries* (domestic sheep) | Brazil | 20 | 6 | None | Farmed | CM | [119] |  |
| *Ovis aries* (domestic sheep) | Brazil | 105 | 26 | None | Farmed | PCR | [122] |  |
| *Ovis aries* (domestic sheep) | Iran | 89 | 17 | < 1 yr | Farmed | CM/PCR | [267] |  |
| *Ovis aries* (domestic sheep) | UK | 64 | 28 | None | Farmed | PCR | [126] |  |
| *Ovis aries* (domestic sheep) | China | 61 | 13 | None | Farmed | CADT | [11] |  |
| *Ovis aries* (domestic sheep) | Brazil | 100 | 34 | 2 groups | Farmed | PCR | [292] |  |
| *Ovis aries* (domestic sheep) | India | 20 | 9 | None | Farmed | CADT | [145] | ❺ |
| *Ovis aries* (domestic sheep) | Poland | 81 | 18 | None | Farmed | PCR | [146] |  |
| *Ovis aries* (domestic sheep) | China | 375 | 16 | 4 groups | Farmed | PCR | [293] |  |
| *Ovis aries* (domestic sheep) | Ecuador | 2 | 1 | None | Farmed | PCR | [294] | ❸ |
| *Ovis aries* (domestic sheep) | China | 716 | 47 | 2 groups | Farmed | CM/PCR | [295] |  |
| *Ovis aries* (domestic sheep) | Ghana | 217 | 28 | None | Farmed | PCR | [164] |  |
| *Ovis aries* (domestic sheep) | Germany | 374 | 0 | None | Farmed | CM | [162] |  |
| *Ovis aries* (Tibetan sheep) | China | 495 | 65 | 3 groups | Free range | PCR | [14] |  |
| *Ovis aries* (domestic sheep) | Ethiopia | 389 | 10 | 3 groups | Farmed | CM/PCR | [296] |  |
| *Ovis aries* (Tibetan sheep) | China | 177 | 3 | None | Free range | PCR | [297] |  |
| *Ovis aries* (domestic sheep) | China | 65 | 0 | None | Farmed | PCR | [174] |  |
| *Ovis aries* (domestic sheep) | Brazil | 11 | 0 | None | Farmed | CM | [178] | ❸ |
| *Ovis aries* (domestic sheep) | China | 318 | 24 | > 2 yrs | Free range | PCR | [298] |  |
| *Ovis aries* (domestic sheep) | Turkey | 50 | 21 | 2 groups | Farmed | PCR | [197] |  |
| *Ovis aries* (domestic sheep) | Iran | 192 | 12 | 3 groups | Farmed | PCR | [188] |  |
| *Ovis aries* (black-boned sheep) | China | 325 | 71 | 4 groups | Free range | PCR | [275] |  |
| *Ovis aries* (Mongolian sheep) | China | 38 | 6 | None | Free range | PCR | [19] |  |
| *Ovis aries* (Tibetan sheep) | China | 620 | 5 | None | Free range | PCR | [276] |  |
| *Ovis aries* (Tibetan sheep) | China | 78 | 1 | None | Free range | PCR | [19] |  |
| *Ovis aries* (domestic sheep) | Algeria | 346 | 24 | 2 groups | Farmed | CM | [299] |  |
| *Ovis aries* (domestic sheep) | China | 209 | 134 | None | Farmed | PCR | [300] |  |
| *Ovis aries* (domestic sheep) | China | 1,014 | 147 | 3 groups | Farmed | PCR | [301] |  |
| *Ovis aries* (domestic sheep) | Iran | 150 | 38 | None | Farmed | CM | [278] |  |
| *Ovis aries* (domestic sheep) | Bangladesh | 112 | 0 | None | Farmed | CM | [214] |  |
| *Ovis aries* (domestic sheep) | Egypt | 112 | 39 | None | Farmed | CM | [216] |  |
| *Ovis aries* (domestic sheep) | UAE | 5 | 0 | None | Captive | PCR | [218] | ❸ |
| *Ovis aries* (domestic sheep) | Iraq | 100 | 44 | None | Farmed | CM | [208] |  |
| *Ovis aries* (domestic sheep) | Greenland | 43 | 16 | None | Farmed | PCR | [284] |  |
| *Ovis aries* (domestic sheep) | China | 797 | 27 | 2 groups | Fa/Fr | PCR | [223] |  |
| *Ovis aries* (domestic sheep) | Ethiopia | 268 | 86 | 2 groups | Farmed | PCR | [222] |  |
| *Ovis aries* (domestic sheep) | China | 120 | 29 | None | Farmed | PCR | [279] |  |
| *Ovis aries* (domestic sheep) | China | 117 | 63 | None | Farmed | PCR | [225] |  |
| *Ovis aries* (Tibetan sheep) | China | 761 | 12 | None | Free range | PCR | [302] |  |
| *Ovis aries* (domestic sheep) | Pakistan | 92 | 17 | 3 groups | Farmed | CADT | [226] |  |
| *Ovis aries* (domestic sheep) | Iran | 50 | 2 | 2 groups | Farmed | CM | [229] |  |
| *Ovis aries* (domestic sheep) | China | 474 | 81 | None | Farmed | PCR | [303] |  |
| *Ovis aries* (domestic sheep) | Portugal | 46 | 8 | None | Farmed | PCR | [235] |  |
| *Ovis aries* (domestic sheep) | Turkey | 120 | 35 | < 2 m | Farmed | PCR | [304] |  |
| *Ovis aries musimon* (European mouflon) | Poland | 2 | 0 | None | Captive | CM | [2] | ❸ |
| *Ovis gmelini* (mouflon) | Spain | 10 | 1 | None | Wild | PCR | [259] |  |
| *Ovis* sp. | China | 11 | 2 | None | Captive | PCR | [15] | ❸ |
| *Pantholops hodgsonii* (Tibetan antelope) | China | 7 | 0 | None | Wild | CADT | [11] |  |
| *Rupicapra pyrenaica* *ornata* (Apennine chamois) | Italy | 54 | 1 | None | Wild | CADT/PCR | [305] |  |
| *Rupicapra pyrenaica* (Pyrenean chamois) | Spain | 62 | 8 | None | Wild | PCR | [259] |  |
| *Rupicapra rupicapra* *rupicapra* (Alpine chamois) | Italy | 103 | 6 | None | Wild | CADT/PCR | [305] |  |
| **Subfamily Cephalophinae** |  |  |  |  |  |  |  |  |
| *Cephalophus callipygus* (Peter’s duiker) | Cen. Af. Rep. | 15 | 0 | None | Wild | PCR | [257] |  |
| *Cephalophus dorsalis* (bay duiker) | Cen. Af. Rep. | 12 | 0 | None | Wild | PCR | [257] |  |
| *Cephalophus monticola* (blue duiker) | Cen. Af. Rep. | 10 | 0 | None | Wild | PCR | [257] |  |
| *Cephalophus silvicultor* (yellow-backed duiker) | Cen. Af. Rep. | 2 | 0 | None | Wild | PCR | [257] | ❸ |
| **Subfamily Hippotraginae** |  |  |  |  |  |  |  |  |
| *Addax nasomaculatus* (addax) | Croatia | 4 | 1 | None | Captive | CADT | [1] | ❸ |
| *Oryx dammah* (scimitar-horned oryx) | Croatia | 5 | 2 | None | Captive | CADT/PCR | [1] |  |
| *Oryx dammah* (scimitar-horned oryx) | Belgium | 2 | 0 | None | Captive | CADT | [4] | ❸ |
| *Oryx dammah* (scimitar-horned oryx) | China | 6 | 0 | None | Captive | PCR | [6] |  |
| *Oryx gazella* (gemsbok) | China | 11 | 1 | None | Captive | PCR | [6] |  |
| *Oryx leucoryx* (Arabian oryx) | Belgium | 6 | 1 | None | Captive | CADT/PCR | [4] |  |
| **Subfamily Reduncinae** |  |  |  |  |  |  |  |  |
| *Kobus ellipsiprymnus* (waterbuck) | Bangladesh | 7 | 1 | None | Captive | PCR | [3] |  |
| **Family Cervidae [deer]** |  |  |  |  |  |  |  |  |
| *Alces* *alces* (moose) | Poland | 2 | 0 | None | Captive | CM | [2] | ❸ |
| *Alces* *alces* (moose) | USA | 6 | 0 | None | Wild | CM | [306] |  |
| *Alces* *alces* (moose) | Canada | 177 | 1 | None | Wild | CADT | [7] |  |
| *Alces* *alces* (moose) | Alaska | 32 | 0 | None | Farmed | CADT | [307] |  |
| *Alces* *alces* (moose) | Norway | 455 | 56 | 3 groups | Wild | CADT | [308] |  |
| *Alces* *alces* (moose) | Belgium | 4 | 1 | None | Captive | CADT | [4] | ❸ |
| *Alces* *alces* (moose) | Poland | 5 | 0 | None | Wild | PCR | [309] |  |
| *Alces* *alces* (moose) | Poland | 23 | 4 | None | Wild | PCR | [310] |  |
| *Axis axis* (cheetal) | Poland | 5 | 0 | None | Captive | CM | [2] |  |
| *Axis axis* (cheetal) | Croatia | 2 | 0 | None | Captive | CADT | [1] | ❸ |
| *Axis axis* (cheetal) | Bangladesh | 30 | 1 | None | Captive | PCR | [3] |  |
| *Capreolus capreolus* (roe deer) | Norway | 291 | 45 | 3 groups | Wild | CADT | [308] |  |
| *Capreolus capreolus* (roe deer) | Poland | 22 | 1 | None | Wild | CADT | [10] |  |
| *Capreolus capreolus* (roe deer) | Croatia | 21 | 5 | None | Captive | CADT/PCR | [311] |  |
| *Capreolus capreolus* (roe deer) | Spain | 224 | 12 | 2 groups | Wild | CADT | [312] |  |
| *Capreolus capreolus* (roe deer) | Poland | 50 | 2 | None | Wild | PCR | [309] |  |
| *Capreolus capreolus* (roe deer) | Poland | 48 | 11 | None | Wild | PCR | [310] |  |
| *Capreolus capreolus* (roe deer) | Spain | 93 | 7 | None | Wild | PCR | [259] |  |
| *Capreolus capreolus* (roe deer) | Spain | 212 | 19 | None | Wild | CADT/PCR | [313] |  |
| *Capreolus capreolus* (roe deer) | Portugal | 39 | 4 | None | Wild | PCR | [235] |  |
| *Cervus albirostris* (white-lipped deer) | China | 5 | 0 | None | Captive | PCR | [5] |  |
| *Cervus canadensis* (elk or wapiti) | Poland | 3 | 0 | None | Captive | CM | [2] | ❸ |
| *Cervus canadensis* (elk or wapiti) | Canada | 38 | 6 | None | Free range | CADT | [7] |  |
| *Cervus canadensis* (elk or wapiti) | Canada | 34 | 0 | None | Wild | CADT | [7] |  |
| *Cervus canadensis* (elk or wapiti) | Belgium | 4 | 1 | None | Captive | CADT/PCR | [4] | ❸ |
| *Cervus canadensis nelsoni* (wapiti) | USA | 1 | 0 | None | Wild | CM | [306] | ❸ |
| *Cervus elaphus* (red deer) | Poland | 2 | 0 | None | Captive | CM | [2] | ❸ |
| *Cervus elaphus* (red deer) | USA | 115 | 2 | None | Wild | CM | [314] |  |
| *Cervus elaphus* (red deer) | USA | 40 | 3 | 1–5 yrs | Wild | CADT | [315] |  |
| *Cervus elaphus* (red deer) | Norway | 289 | 5 | 3 groups | Wild | CADT | [308] |  |
| *Cervus elaphus* (red deer) | Poland | 118 | 2 | None | Fa/Wi | CADT | [10] |  |
| *Cervus elaphus* (red deer) | Belgium | 1 | 0 | None | Captive | CADT | [4] | ❸ |
| *Cervus elaphus* (red deer) | Croatia | 374 | 4 | None | Captive | CADT | [311] |  |
| *Cervus elaphus* (red deer) | Poland | 61 | 1 | None | Wild | PCR | [309] |  |
| *Cervus elaphus* (red deer) | Poland | 28 | 5 | None | Wild | PCR | [310] |  |
| *Cervus elaphus* (red deer) | China | 16 | 0 | None | Farmed | CM | [316] |  |
| *Cervus elaphus* (red deer) | China | 83 | 7 | None | Wild | PCR | [225] |  |
| *Cervus elaphus* (red deer) | China | 3 | 0 | None | Captive | PCR | [6] | ❸ |
| *Cervus elaphus* (red deer) | Spain | 653 | 25 | None | Fa/Wi | PCR | [259] |  |
| *Cervus elaphus* (red deer) | Portugal | 96 | 4 | None | Wild | PCR | [235] |  |
| *Cervus eldii* (*eld's deer*) | China | 7 | 1 | None | Wild | PCR | [317] |  |
| *Cervus nippon* (sika deer) | Japan | 1 | 0 | None | Captive | CADT | [258] | ❸ |
| *Cervus nippon* (sika deer) | China | 599 | 5 | None | Farmed | CM/PCR | [316] |  |
| *Cervus nippon* (sika deer) | Japan | 271 | 2 | None | Wild | PCR | [318] |  |
| *Cervus nippon* (sika deer) | China | 818 | 5 | None | Farmed | PCR | [211] |  |
| *Cervus nippon* (sika deer) | China | 32 | 0 | None | Captive | PCR | [5] |  |
| *Cervus nippon* (sika deer) | China | 63 | 5 | None | Wild | PCR | [225] |  |
| *Cervus nippon* (sika deer) | China | 10 | 0 | None | Captive | PCR | [6] |  |
| *Cervus nippon* (sika deer) | China | 7 | 1 | None | Wild | PCR | [317] |  |
| *Cervus nippon dybowskii* (Manchurian sika deer) | Poland | 2 | 0 | None | Captive | CM | [2] | ❸ |
| *Cervus nippon dybowskii* (Manchurian sika deer) | Belgium | 1 | 0 | None | Captive | CADT | [4] | ❸ |
| *Cervus* sp. | Canada | 649 | 1 | None | Wild | CADT | [7] |  |
| *Cervus* sp. | China | 9 | 0 | None | Captive | PCR | [15] |  |
| *Dama* *dama* (fallow deer) | Poland | 1 | 0 | None | Captive | CM | [2] | ❸ |
| *Dama* *dama* (fallow deer) | USA | 4 | 0 | 1–5 yrs | Wild | CADT | [315] | ❸ |
| *Dama* *dama* (fallow deer) | Japan | 3 | 0 | None | Captive | CADT | [258] | ❸ |
| *Dama* *dama* (fallow deer) | Italy | 139 | 16 | None | Wild | CM/PCR | [319] |  |
| *Dama* *dama* (fallow deer) | Poland | 65 | 0 | None | Wild | PCR | [309] |  |
| *Dama* *dama* (fallow deer) | Brazil | 53 | 1 | None | Captive | CM | [320] |  |
| *Dama* *dama* (fallow deer) | China | 2 | 0 | None | Captive | PCR | [5] | ❸ |
| *Dama* *dama* (fallow deer) | Spain | 96 | 5 | None | Wild | PCR | [259] |  |
| *Elaphurus davidianus* (Père david's deer) | Poland | 5 | 0 | None | Captive | CM | [2] |  |
| *Elaphurus davidianus* (Père david's deer) | Belgium | 1 | 0 | None | Captive | CADT | [4] | ❸ |
| *Elaphurus davidianus* (Père david's deer) | China | 47 | 0 | None | Farmed | CM | [316] |  |
| *Elaphurus davidianus* (Père david's deer) | China | 2 | 0 | None | Captive | PCR | [5] | ❸ |
| *Elaphurus davidianus* (Père david's deer) | China | 24 | 6 | None | Captive | PCR | [6] |  |
| *Elaphurus davidianus* (Père david's deer) | China | 9 | 0 | None | Wild | PCR | [317] |  |
| *Moschus* *berezovskii* (forest musk deer) | China | 223 | 5 | None | Farmed | PCR | [321] |  |
| *Moschus* *chrysogaster* (Alpine musk deer) | China | 202 | 39 | 2 groups | Farmed | PCR | [322] |  |
| *Muntiacus muntjak* (barking deer) | Bangladesh | 6 | 0 | None | Captive | PCR | [3] |  |
| *Muntiacus reevesi* (Chinese muntjac) | Poland | 1 | 0 | None | Captive | CM | [2] | ❸ |
| *Odocoileus hemionus* (black-tailed deer) | USA | 38 | 0 | 1–5 yrs | Wild | CADT | [315] |  |
| *Odocoileus virginianus* (white-tailed deer) | Poland | 2 | 0 | None | Captive | CM | [2] | ❸ |
| *Odocoileus virginianus* (white-tailed deer) | USA | 3 | 0 | None | Wild | CM | [306] | ❸ |
| *Odocoileus virginianus* (white-tailed deer) | USA | 394 | 5 | 2 groups | Pastoral | CADT | [323] |  |
| *Odocoileus virginianus* (white-tailed deer) | USA | 26 | 1 | None | Wild | CADT/PCR | [324] |  |
| *Odocoileus virginianus* (white-tailed deer) | USA | 79 | 1 | 3 groups | Wild | PCR | [325] |  |
| *Rangifer tarandus* (reindeer or caribou) | Poland | 3 | 0 | None | Captive | CM | [2] | ❸ |
| *Rangifer tarandus* (reindeer or caribou) | Alaska | 49 | 0 | None | Farmed | CADT | [307] |  |
| *Rangifer tarandus* (reindeer or caribou) | Norway | 155 | 11 | 3 groups | Wild | CADT | [308] |  |
| *Rangifer tarandus* (reindeer or caribou) | Belgium | 2 | 0 | None | Captive | CADT | [4] | ❸ |
| *Rangifer tarandus* (reindeer or caribou) | Canada | 149 | 3 | None | Wild | CM | [326] |  |
| *Rangifer tarandus* (reindeer or caribou) | China | 124 | 9 | None | Wild | PCR | [225] |  |
| *Rangifer tarandus* (reindeer or caribou) | Alaska | 55 | 0 | None | Wild | PCR | [327] |  |
| *Rusa unicolor* (sambar) | Ausralia | 137 | 29 | None | Wild | PCR | [93] |  |
| *Rusa unicolor* (sambar) | Australia | 1,563 | 10 | None | Wild | PCR | [328] |  |
| **Family Giraffidae** |  |  |  |  |  |  |  |  |
| *Giraffa camelopardalis* (masai giraffe) | Poland | 2 | 0 | None | Captive | CM | [2] | ❸ |
| *Giraffa camelopardalis* (masai giraffe) | Japan | 3 | 0 | None | Captive | CADT | [258] | ❸ |
| *Giraffa camelopardalis* (masai giraffe) | Belgium | 1 | 0 | None | Captive | CADT | [4] | ❸ |
| *Giraffa camelopardalis* (masai giraffe) | Brazil | 53 | 4 | None | Captive | CM | [320] |  |
| *Giraffa camelopardalis* (masai giraffe) | Bangladesh | 4 | 1 | None | Captive | PCR | [3] | ❸ |
| *Giraffa camelopardalis* (masai giraffe) | China | 5 | 0 | None | Captive | PCR | [5] |  |
| *Giraffa camelopardalis* (masai giraffe) | China | 21 | 9 | None | Captive | PCR | [329] |  |
| *Giraffa camelopardalis* (masai giraffe) | China | 13 | 2 | None | Captive | PCR | [6] |  |
| *Giraffa camelopardalis* (masai giraffe) | Serbia | 1 | 0 | None | Captive | PCR | [8] |  |
| **Family Suidae** |  |  |  |  |  |  |  |  |
| *Hylochoerus meinertzhageni* (giant forest hog) | Cen. Af. Rep. | 2 | 0 | None | Wild | PCR | [257] | ❸ |
| *Potamochoerus porcus* (red river hog) | Cen. Af. Rep. | 23 | 0 | None | Wild | PCR | [257] |  |
| *Sus scrofa* (wild boar) | USA | 221 | 17 | 2 groups | Wild | CADT | [330] |  |
| *Sus scrofa* (wild boar) | Poland | 5 | 0 | None | Captive | CM | [10] |  |
| *Sus scrofa* (wild boar) | Croatia | 144 | 2 | None | Captive | CADT/PCR | [311] |  |
| *Sus scrofa* (wild boar) | Spain | 381 | 5 | 2 groups | Wild | CADT | [312] |  |
| *Sus scrofa* (wild boar) | Poland | 27 | 11 | None | Wild | PCR | [310] |  |
| *Sus scrofa* (wild boar) | Iran | 25 | 0 | None | Wild | CM | [331] |  |
| *Sus scrofa* (wild boar) | USA | 370 | 16 | 2 groups | Wild | CADT/PCR | [332] |  |
| *Sus scrofa* (wild boar) | China | 357 | 11 | None | Captive | PCR | [333] |  |
| *Sus scrofa* (wild boar) | Spain | 359 | 20 | None | Wild | PCR | [259] |  |
| *Sus scrofa* (wild boar) | South Korea | 612 | 125 | None | Wild | PCR | [334] |  |
| *Sus scrofa* (wild boar) | Spain | 498 | 6 | 4 groups | Wild | PCR | [335] |  |
| *Sus scrofa* (wild boar) | Portugal | 107 | 16 | None | Wild | PCR | [235] |  |
| *Sus scrofa domesticus* (domestic pig) | USA | 325 | 24 | 4 groups | Farmed | CADT | [336] |  |
| *Sus scrofa domesticus* (domestic pig) | Canada | 218 | 17 | 2 groups | Farmed | CADT | [32] |  |
| *Sus scrofa domesticus* (domestic pig) | Greece | 141 | 0 | 2–48 m | Farmed | CM | [34] |  |
| *Sus scrofa domesticus* (domestic pig) | Taiwan | 17 | 3 | None | Watershed | CADT | [38] |  |
| *Sus scrofa domesticus* (domestic pig) | Australia | 646 | 5 | None | Farmed | CM | [337] |  |
| *Sus scrofa domesticus* (domestic pig) | Denmark | 1,237 | 215 | 3 groups | Farmed | CADT/PCR | [64] |  |
| *Sus scrofa domesticus* (domestic pig) | Norway | 686 | 10 | None | Farmed | CADT | [338] | ❺ |
| *Sus scrofa domesticus* (domestic pig) | Taiwan | 2 | 0 | None | Farmed | CADT | [73] | ❸ |
| *Sus scrofa domesticus* (domestic pig) | Australia | 289 | 90 | 3 groups | Farmed | PCR | [339] |  |
| *Sus scrofa domesticus* (domestic pig) | Turkey | 238 | 9 | 2 groups | Farmed | CM | [340] |  |
| *Sus scrofa domesticus* (domestic pig) | Canada | 122 | 81 | None | Farmed | PCR | [341] | ❺ |
| *Sus scrofa domesticus* (domestic pig) | Canada | 633 | 6 | 2 groups | Farmed | CADT | [342] |  |
| *Sus scrofa domesticus* (domestic pig) | Zambia | 217 | 26 | 4 groups | Farmed | CADT | [343] |  |
| *Sus scrofa domesticus* (domestic pig) | Brazil | 90 | 3 | None | Farmed | CM | [122] | ❺ |
| *Sus scrofa domesticus* (domestic pig) | USA | 31 | 0 | None | Fair | CADT | [125] |  |
| *Sus scrofa domesticus* (domestic pig) | UK | 7 | 4 | None | Farmed | PCR | [126] | ❸ |
| *Sus scrofa domesticus* (domestic pig) | Cambodia | 76 | 0 | None | Farmed | CM | [344] |  |
| *Sus scrofa domesticus* (domestic pig) | Denmark | 856 | 120 | 4 groups | Farmed | CADT/PCR | [345] |  |
| *Sus scrofa domesticus* (domestic pig) | Poland | 84 | 25 | None | Farmed | CADT/PCR | [146] |  |
| *Sus scrofa domesticus* (domestic pig) | Ecuador | 36 | 2 | None | Farmed | PCR | [294] |  |
| *Sus scrofa domesticus* (domestic pig) | China | 560 | 45 | 4 groups | Farmed | PCR | [346] |  |
| *Sus scrofa domesticus* (domestic pig) | Brazil | 25 | 0 | None | Farmed | CM | [178] |  |
| *Sus scrofa domesticus* (domestic pig) | Philippines | 44 | 7 | None | Farmed | CM | [195] |  |
| *Sus scrofa domesticus* (domestic pig) | China | 801 | 21 | 4 groups | Farmed | PCR | [347] |  |
| *Sus scrofa domesticus* (domestic pig) | China | 93 | 25 | None | Farmed | PCR | [348] |  |
| *Sus scrofa domesticus* (domestic pig) | Nigeria | 209 | 53 | 3 groups | Farmed | CADT | [349] |  |
| *Sus scrofa domesticus* (domestic pig) | China | 450 | 28 | 4 groups | Farmed | PCR | [350] |  |
| *Sus scrofa domesticus* (domestic pig) | Madagascar | 40 | 16 | None | Farmed | CADT | [240] |  |
| *Sus scrofa domesticus* (domestic pig) | Uganda | 12 | 0 | None | Farmed | CADT | [210] | ❸ |
| *Sus scrofa domesticus* (domestic pig) | Ecuador | 26 | 5 | None | Farmed | CM | [351] |  |
| *Sus scrofa domesticus* (domestic pig) | Vietnam | 28 | 7 | None | Farmed | PCR | [209] |  |
| *Sus scrofa domesticus* (domestic pig) | Taiwan | 141 | 6 | None | Farmed | PCR | [213] |  |
| *Sus scrofa domesticus* (domestic pig) | China | 396 | 21 | 3 groups | Farmed | PCR | [352] |  |
| *Sus scrofa domesticus* (domestic pig) | Nepal | 100 | 7 | 3 groups | Farmed | CM | [353] |  |
| *Sus scrofa domesticus* (domestic pig) | China | 59 | 1 | None | Farmed | PCR | [225] |  |
| *Sus scrofa domesticus* (domestic pig) | China | 725 | 195 | 5 groups | Farmed | PCR | [354] |  |
| *Sus scrofa domesticus* (domestic pig) | China | 826 | 8 | None | Farmed | PCR | [355] |  |
| *Sus scrofa domesticus* (domestic pig) | China | 514 | 46 | 4 groups | Farmed | PCR | [356] |  |
| *Sus scrofa domesticus* (Tibetan pig) | China | 60 | 0 | None | Free range | PCR | [22] |  |
| *Sus* sp. | Australia | 27 | 0 | None | Farmed | PCR | [93] |  |
| **Family Tayassuidae** |  |  |  |  |  |  |  |  |
| *Dicotyles tajacu* (collared peccary) | Poland | 4 | 0 | None | Captive | CM | [2] | ❸ |
| *Dicotyles tajacu* (collared peccary) | Croatia | 2 | 2 | None | Captive | CADT/PCR | [1] | ❸ |
| **Family Camelidae** |  |  |  |  |  |  |  |  |
| *Camelus bactrianus* (Bactrian camel) | Poland | 3 | 0 | None | Captive | CM | [2] | ❸ |
| *Camelus bactrianus* (Bactrian camel) | Japan | 3 | 0 | None | Captive | CADT | [258] | ❸ |
| *Camelus bactrianus* (Bactrian camel) | Croatia | 3 | 0 | > 2 yrs | Captive | CADT | [1] | ❸ |
| *Camelus bactrianus* (Bactrian camel) | China | 40 | 3 | 3 groups | Free range | PCR | [19] |  |
| *Camelus bactrianus* (Bactrian camel) | China | 852 | 84 | 3 groups | Free range | PCR | [357] |  |
| *Camelus bactrianus* (Bactrian camel) | China | 42 | 2 | None | Captive | PCR | [5] |  |
| *Camelus bactrianus* (Bactrian camel) | Serbia | 2 | 0 | None | Captive | PCR | [8] | ❸ |
| *C. bactrianus*/*C. dromedarious* | USA | 77 | 1 | None | Not stated | CM | [358] |  |
| *Camelus dromedarious* (dromedary camel) | Poland | 1 | 0 | None | Not stated | CM | [2] | ❸ |
| *Camelus dromedarious* (dromedary camel) | Saudi Arabia | 7 | 7 | None | Farmed | CM | [359] |  |
| *Camelus dromedarious* (dromedary camel) | Egypt | 120 | 6 | None | Free range | CM | [360] |  |
| *Camelus dromedarious* (dromedary camel) | Iraq | 100 | 24 | 3 groups | Free range | CM | [361] |  |
| *Camelus dromedarious* (dromedary camel) | Iraq | 200 | 40 | None | Free range | CM | [362] |  |
| *Camelus dromedarious* (dromedary camel) | China | 2 | 0 | None | Captive | PCR | [15] | ❸ |
| *Camelus dromedarious* (dromedary camel) | Algeria | 717 | 0 | 4 groups | Free range | CM | [363] |  |
| *Camelus dromedarious* (dromedary camel) | Iraq | 120 | 5 | 3 groups | Free range | CM | [364] |  |
| *Camelus dromedarious* (dromedary camel) | Bangladesh | 2 | 0 | None | Captive | PCR | [3] | ❸ |
| *Camelus dromedarious* (dromedary camel) | Algeria | 68 | 12 | None | Free range | PCR | [365] |  |
| *Camelus dromedarious* (dromedary camel) | Egypt | 102 | 4 | 3 groups | Free range | PCR | [366] |  |
| *Camelus dromedarious* (dromedary camel) | Iran | 30 | 0 | 2 groups | Free range | CM | [229] |  |
| *Camelus dromedarious* (dromedary camel) | Egypt | 121 | 16 | 2 groups | Free range | CM | [367] |  |
| *Camelus* sp. | China | 4 | 1 | None | Captive | PCR | [6] | ❸ |
| *Lama glama* (llama) | Poland | 4 | 0 | None | Captive | CM | [2] | ❸ |
| *Lama glama* (llama) | USA | 354 | 12 | 8 groups | Farmed | CADT | [368] |  |
| *Lama glama* (llama) | USA | 45 | 8 | < 7 m | Farmed | CADT | [369] |  |
| *Lama glama* (llama) | Japan | 1 | 0 | None | Captive | CADT | [258] | ❸ |
| *Lama glama* (llama) | UK | 1 | 0 | None | Farmed | PCR | [126] | ❸ |
| *Lama glama* (llama) | Ecuador | 9 | 4 | None | Farmed | CM | [351] |  |
| *Lama glama* (llama) | China | 24 | 2 | None | Captive | PCR | [6] |  |
| *Lama guanicoe* (guanaco) | Poland | 2 | 0 | None | Captive | CM | [2] | ❸ |
| *Lama pacos* (alpaca) | USA | 61 | 3 | 3 groups | Farmed | PCR | [370] |  |
| *Lama pacos* (alpaca) | Peru | 274 | 137 | 2 groups | Farmed | CADT/PCR | [371] |  |
| *Lama pacos* (alpaca) | UK | 6 | 1 | None | Farmed | PCR | [126] |  |
| *Lama pacos* (alpaca) | Peru | 352 | 46 | 2 groups | Farmed | PCR | [372] |  |
| *Lama pacos* (alpaca) | Australia | 1,421 | 6 | 2 groups | Farmed | PCR | [373] |  |
| *Lama pacos* (alpaca) | China | 13 | 0 | None | Captive | PCR | [5] |  |
| *Lama vicugna* (vicuna) | China | 26 | 0 | None | Captive | PCR | [6] |  |
| **Family Balaenidae** |  |  |  |  |  |  |  |  |
| *Delphinapterus leucas* (beluga whale) | Canada | 16 | 0 | None | Wild | CADT | [374] |  |
| *Delphinapterus leucas* (beluga whale) | Canada | 11 | 0 | None | Wild | CADT | [375] |  |
| *Delphinapterus leucas* (beluga whale) | Alaska | 18 | 0 | None | Wild | CADT | [376] |  |
| *Balaena mysticetus* (bowhead whale) | Alaska | 39 | 13 | None | Wild | CADT | [376] |  |
| *Eubalaena glacialis* (North Atlantic whale) | Alaska | 49 | 35 | None | Wild | CADT | [376] |  |
| **Family Balaenopteridae** |  |  |  |  |  |  |  |  |
| *Balaenoptera acutorostrata* (minke whale) | Spain | 5 | 2 | None | Wild | CADT/PCR | [377] |  |
| *Balaenoptera physalus* (fin whale) | Spain | 1 | 0 | None | Wild | CADT | [377] | ❸ |
| **Family Delphinidae** |  |  |  |  |  |  |  |  |
| *Delphinus delphis* (common dolphin) | USA | 1 | 1 | None | Wild | PCR | [378] | ❸ |
| *Delphinus delphis* (common dolphin) | Spain | 133 | 8 | None | Wild | CADT/PCR | [379] |  |
| *Globicephala melas* (long-finned pilot whale) | Spain | 7 | 0 | None | Wild | CADT | [377] |  |
| *Grampus griseus* (Risso's dolphin) | Spain | 2 | 0 | None | Wild | CADT | [377] | ❸ |
| *Stenella coeruleoalba* (striped dolphin) | Spain | 18 | 3 | None | Wild | CADT/PCR | [377] |  |
| *Stenella coeruleoalba* (striped dolphin) | Italy | 11 | 2 | None | Wild | PCR | [380] |  |
| *Tursiops aduncus* (Indo-Pacific bottlenose dolphin) | Egypt | 94 | 2 | None | Wild | CM | [381] |  |
| *Tursiops truncatus* (Atlantic bottlenose dolphin) | Spain | 14 | 0 | None | Wild | CADT | [377] |  |
| **Family Kogiidae** |  |  |  |  |  |  |  |  |
| *Kogia breviceps* (pygmy sperm whale) | Spain | 1 | 1 | None | Wild | CADT/PCR | [377] | ❸ |
| **Family Phocoenidae** |  |  |  |  |  |  |  |  |
| *Phocoena phocoena* (harbour porpoise) | Spain | 17 | 1 | None | Wild | CADT/PCR | [377] |  |
| **Family Physeteridae** |  |  |  |  |  |  |  |  |
| *Physeter macrocephalus* (sperm whale) | Spain | 25 | 4 | None | Wild | CADT | [382] |  |
| **Family Ziphiidae** |  |  |  |  |  |  |  |  |
| *Hyperoodon ampullatus* (northern bottlenose whale) | Canada | 1 | 0 | None | Wild | CADT | [375] | ❸ |
| **Family Hippopotamidae** |  |  |  |  |  |  |  |  |
| *Choeropsis liberiensis* (pygmy hippopotamus) | Poland | 1 | 0 | None | Captive | CM | [2] | ❸ |
| *Choeropsis liberiensis* (pygmy hippopotamus) | Croatia | 1 | 0 | None | Wild | CADT | [1] | ❸ |
| *Hippopotamus amphibius* (common hippopotamus) | Bangladesh | 2 | 0 | None | Captive | PCR | [3] | ❸ |
| *Hippopotamus amphibius* (common hippopotamus) | China | 4 | 0 | None | Captive | PCR | [5] | ❸ |
| **Order Carnivora** |  |  |  |  |  |  |  |  |
| **Family Ailuridae** |  |  |  |  |  |  |  |  |
| *Ailurus fulgens* (red panda) | Japan | 2 | 0 | None | Captive | CADT | [258] | ❸ |
| *Ailurus fulgens* (red panda) | Croatia | 2 | 0 | None | Captive | CADT | [1] | ❸ |
| *Ailurus fulgens* (red panda) | China | 3 | 0 | None | Captive | PCR | [5] | ❸ |
| *Ailurus fulgens* (red panda) | China | 10 | 0 | None | Captive | PCR | [6] |  |
| **Family Canidae** |  |  |  |  |  |  |  |  |
| *Canis aureus* (golden jackal) | Croatia | 8 | 1 | None | Wild | CADT/PCR | [311] |  |
| *Canis aureus* (golden jackal) | Bangladesh | 12 | 3 | None | Captive | PCR | [3] |  |
| *Canis familiaris* (domestic dog) | USA | 4,058 | 33 | None | Pet | CM | [383] |  |
| *Canis familiaris* (domestic dog) | Scotland | 119 | 5 | None | Stray | CM | [384] |  |
| *Canis familiaris* (domestic dog) | USA | 22 | 1 | None | Shelter | CM | [306] |  |
| *Canis familiaris* (domestic dog) | Australia | 333 | 68 | 3 groups | Pe/Sh/BS | CM | [385] |  |
| *Canis familiaris* (domestic dog) | Australia | 100 | 21 | None | Stray | CM | [386] |  |
| *Canis familiaris* (domestic dog) | USA | 2,294 | 166 | 2 groups | Pet | CM | [387] |  |
| *Canis familiaris* (domestic dog) | USA | 117 | 42 | < 6 m | Pet | CM | [388] |  |
| *Canis familiaris* (domestic dog) | New Zealand | 223 | 31 | None | Pet | CM | [389] |  |
| *Canis familiaris* (domestic dog) | Belgium | 2,324 | 0 | None | Stray | CM | [390] |  |
| *Canis familiaris* (domestic dog) | Germany | 3,329 | 20 | None | Pet | CM | [391] |  |
| *Canis familiaris* (domestic dog) | Australia | 61 | 22 | None | Pet | CM | [392] |  |
| *Canis familiaris* (domestic dog) | Australia | 132 | 14 | 2 groups | Sh/FD/Pe | CM | [393] |  |
| *Canis familiaris* (domestic dog) | Australia | 182 | 31 | None | Stray | CM | [394] |  |
| *Canis familiaris* (domestic dog) | Australia | 55 | 8 | None | Ur/Pet | CADT | [395] |  |
| *Canis familiaris* (domestic dog) | USA | 8,077 | 380 | < 15 yrs | Pet | CM | [396] |  |
| *Canis familiaris* (domestic dog) | USA | 6,458 | 40 | 5 groups | Shelter | CM | [397] |  |
| *Canis familiaris* (domestic dog) | Spain | 912 | 111 | None | Kennel | CM | [29] |  |
| *Canis familiaris* (domestic dog) | USA | 309 | 14 | 6 groups | Shelter | CM | [398] |  |
| *Canis familiaris* (domestic dog) | Australia | 421 | 93 | None | Re/PS/Pe | CM | [399] |  |
| *Canis familiaris* (domestic dog) | Brazil | 140 | 4 | None | Stray | CM | [400] |  |
| *Canis familiaris* (domestic dog) | Japan | 1,035 | 151 | 4 groups | Ho | CM | [401] |  |
| *Canis familiaris* (domestic dog) | Canada | 1,216 | 88 | 4 groups | Pet | CADT | [402] |  |
| *Canis familiaris* (domestic dog) | Japan | 81 | 39 | None | Pet | CADT | [403] |  |
| *Canis familiaris* (domestic dog) | Serbia | 167 | 24 | > 1 yr | Pe/St/FD | CM | [404] |  |
| *Canis familiaris* (domestic dog) | Brazil | 271 | 33 | 2 groups | Pe/St | CM | [405] |  |
| *Canis familiaris* (domestic dog) | Brazil | 250 | 49 | None | Pet | CM | [406] |  |
| *Canis familiaris* (domestic dog) | USA | 50 | 16 | None | Sh/RD | CM | [407] |  |
| *Canis familiaris* (domestic dog) | Germany | 8,438 | 1,401 | None | Pet | CADT | [408] |  |
| *Canis familiaris* (domestic dog) | USA | 130 | 7 | None | Pet | CADT | [409] |  |
| *Canis familiaris* (domestic dog) | Brazil | 100 | 41 | 2 groups | Kennel | CM | [410] |  |
| *Canis familiaris* (domestic dog) | Czech Rep. | 23 | 9 | None | Shelter | CM | [411] |  |
| *Canis familiaris* (domestic dog) | India | 101 | 20 | None | Stray | PCR | [412] |  |
| *Canis familiaris* (domestic dog) | Italy | 616 | 131 | None | Pe/Ke/St | CM | [413] |  |
| *Canis familiaris* (domestic dog) | Canada | 107 | 12 | None | Research | CADT | [414] |  |
| *Canis familiaris* (domestic dog) | Japan | 772 | 22 | None | Pet | CM | [415] |  |
| *Canis familiaris* (domestic dog) | Italy | 113 | 17 | None | Ke/Co | CM/PCR | [56] |  |
| *Canis familiaris* (domestic dog) | Italy | 105 | 20 | 2 groups | Pe/St | CADT | [416] |  |
| *Canis familiaris* (domestic dog) | Germany | 264 | 78 | None | Shelter | CADT | [417] |  |
| *Canis familiaris* (domestic dog) | Germany | 1,281 | 30 | None | Pet | CADT | [52] |  |
| *Canis familiaris* (domestic dog) | Venezuela | 614 | 0 | 5 groups | Pet | CM | [418] |  |
| *Canis familiaris* (domestic dog) | Brazil | 437 | 6 | None | Pet | CM | [419] |  |
| *Canis familiaris* (domestic dog) | Poland | 86 | 46 | < 8 yrs | Pet | CADT | [420] |  |
| *Canis familiaris* (domestic dog) | Brazil | 166 | 52 | 2 groups | Pe/Sh | CM | [421] |  |
| *Canis familiaris* (domestic dog) | Japan | 361 | 135 | 2 groups | Kennel | CADT | [422] |  |
| *Canis familiaris* (domestic dog) | Japan | 513 | 65 | None | Pet | CADT | [423] |  |
| *Canis familiaris* (domestic dog) | Italy | 183 | 101 | 2 groups | Shelter | CADT | [424] |  |
| *Canis familiaris* (domestic dog) | Mexico | 200 | 93 | > 2 years | Stray | CM | [425] |  |
| *Canis familiaris* (domestic dog) | USA | 98 | 36 | None | Shelter | CADT | [426] |  |
| *Canis familiaris* (domestic dog) | Costa Rica | 1,136 | 227 | None | Pet | CM | [427] |  |
| *Canis familiaris* (domestic dog) | Italy | 307 | 53 | 4 groups | Pe/Ke/St | CM | [428] |  |
| *Canis familiaris* (domestic dog) | USA | 16,064 | 2,506 | None | Pet | CADT | [429] |  |
| *Canis familiaris* (domestic dog) | Argentina | 2,193 | 195 | 4 groups | Pet | CM | [430] |  |
| *Canis familiaris* (domestic dog) | Brazil | 95 | 8 | None | Ur/Pet | CM | [431] |  |
| *Canis familiaris* (domestic dog) | Canada | 102 | 7 | None | Pet | CADT | [432] |  |
| *Canis familiaris* (domestic dog) | Chile | 972 | 211 | 2 groups | Pet | CM | [433] |  |
| *Canis familiaris* (domestic dog) | Canada | 9,486 | 11 | 2 groups | Pet | CM | [434] |  |
| *Canis familiaris* (domestic dog) | Poland | 350 | 32 | None | Pet | PCR | [435] |  |
| *Canis familiaris* (domestic dog) | Czech Rep. | 3,780 | 37 | None | U/Pe | CM | [436] |  |
| *Canis familiaris* (domestic dog) | Czech Rep. | 540 | 12 | None | R/FD | CM | [436] |  |
| *Canis familiaris* (domestic dog) | Czech Rep. | 524 | 17 | None | Ur/Sh | CM | [436] |  |
| *Canis familiaris* (domestic dog) | UK | 608 | 52 | 4–11 yrs | Pet | CM | [437] |  |
| *Canis familiaris* (domestic dog) | Norway | 290 | 60 | 4 groups | Pet | CADT | [438] |  |
| *Canis familiaris* (domestic dog) | Thailand | 229 | 18 | 4 groups | Pet | CM/PCR | [439] |  |
| *Canis familiaris* (domestic dog) | Brazil | 1,473 | 66 | 2 groups | Pet | CM | [440] |  |
| *Canis familiaris* (domestic dog) | Spain | 1,800 | 18 | 4 groups | Pe/St | CM | [441] |  |
| *Canis familiaris* (domestic dog) | Spain | 251 | 1 | 3 groups | Pet | CM | [442] |  |
| *Canis familiaris* (domestic dog) | Spain | 1,161 | 82 | None | Stray | CM | [443] |  |
| *Canis familiaris* (domestic dog) | Brazil | 410 | 119 | 2 groups | Pe/St | CM | [444] |  |
| *Canis familiaris* (domestic dog) | Greece | 281 | 12 | 3 groups | Hu/Sh | CM | [445] |  |
| *Canis familiaris* (domestic dog) | Brazil | 53 | 13 | 2 groups | Pet | CM | [446] |  |
| *Canis familiaris* (domestic dog) | Finland | 150 | 8 | 2 groups | Pet | CADT/PCR | [447] |  |
| *Canis familiaris* (domestic dog) | Brazil | 200 | 17 | 4 groups | Pet | CM | [448] |  |
| *Canis familiaris* (domestic dog) | Slovakia | 727 | 12 | 3 groups | Pe/Se/Gu | CM | [449] |  |
| *Canis familiaris* (domestic dog) | Hungary | 187 | 110 | None | Kennel | CADT/PCR | [450] |  |
| *Canis familiaris* (domestic dog) | Brazil | 19 | 7 | None | Pet | PCR | [451] |  |
| *Canis familiaris* (domestic dog) | Brazil | 1,755 | 150 | None | Pet | CM | [452] |  |
| *Canis familiaris* (domestic dog) | UK | 4,526 | 380 | 2 groups | Pet | CM | [453] |  |
| *Canis familiaris* (domestic dog) | Belgium | 413 | 76 | None | Pet | CADT | [454] |  |
| *Canis familiaris* (domestic dog) | Iran | 147 | 1 | None | Pet | CM | [455] |  |
| *Canis familiaris* (domestic dog) | Brazil | 254 | 43 | 2 groups | Pe/St | CM | [456] |  |
| *Canis familiaris* (domestic dog) | Brazil | 1,837 | 179 | 4 groups | Pet | CADT | [457] |  |
| *Canis familiaris* (domestic dog) | South Korea | 472 | 53 | 3 groups | Pe/Ke | CADT | [458] |  |
| *Canis familiaris* (domestic dog) | Brazil | 200 | 33 | 3 groups | Sh/Pe | CM | [459] |  |
| *Canis familiaris* (domestic dog) | Serbia | 151 | 22 | > 1 yr | Pe/St | CM | [460] |  |
| *Canis familiaris* (domestic dog) | Australia | 1,400 | 130 | 2 groups | Re/Cl | CM | [461] |  |
| *Canis familiaris* (domestic dog) | Italy | 240 | 64 | 2 groups | Pet | CM/PCR | [462] |  |
| *Canis familiaris* (domestic dog) | Italy | 415 | 32 | None | U/Gr | CADT | [463] |  |
| *Canis familiaris* (domestic dog) | Belgium | 1,159 | 263 | None | Ke/Pe | CADT/PCR | [464] |  |
| *Canis familiaris* (domestic dog) | Romania | 153 | 24 | 2 groups | Pet | CM | [465] |  |
| *Canis familiaris* (domestic dog) | USA | 6,555 | 216 | None | Pet | CM | [466] |  |
| *Canis familiaris* (domestic dog) | Spain | 505 | 31 | 2 groups | U/Pe/St | CM | [467] |  |
| *Canis familiaris* (domestic dog) | Japan | 420 | 60 | 2 groups | Pet | CM | [468] |  |
| *Canis familiaris* (domestic dog) | Japan | 350 | 38 | 2 groups | Pet | CM | [468] |  |
| *Canis familiaris* (domestic dog) | Japan | 335 | 39 | 2 groups | Pet | CM | [468] |  |
| *Canis familiaris* (domestic dog) | USA | 1,199,293 | 48,353 | 6 groups | Pet | CM | [469] |  |
| *Canis familiaris* (domestic dog) | Netherlands | 92 | 14 | None | Pet | CM/PCR | [470] |  |
| *Canis familiaris* (domestic dog) | Italy | 143 | 44 | None | Pet | PCR | [471] |  |
| *Canis familiaris* (domestic dog) | Brazil | 81 | 9 | None | Pet | CM | [472] |  |
| *Canis familiaris* (domestic dog) | Iran | 174 | 1 | None | Pe/FD | CM | [473] |  |
| *Canis familiaris* (domestic dog) | Italy | 127 | 26 | 2 groups | Kennel | PCR | [474] |  |
| *Canis familiaris* (domestic dog) | Thailand | 104 | 73 | None | Pet | PCR | [475] |  |
| *Canis familiaris* (domestic dog) | Belgium | 281 | 80 | 4 groups | Pet | CADT | [476] |  |
| *Canis familiaris* (domestic dog) | Germany | 3,175 | 754 | 4 groups | Pet | CADT | [476] |  |
| *Canis familiaris* (domestic dog) | Spain | 1,757 | 441 | 4 groups | Pet | CADT | [476] |  |
| *Canis familiaris* (domestic dog) | France | 1,017 | 280 | 4 groups | Pet | CADT | [476] |  |
| *Canis familiaris* (domestic dog) | Italy | 1,468 | 380 | 4 groups | Pet | CADT | [476] |  |
| *Canis familiaris* (domestic dog) | Netherlands | 727 | 179 | 4 groups | Pet | CADT | [476] |  |
| *Canis familiaris* (domestic dog) | UK | 260 | 38 | 4 groups | Pet | CADT | [476] |  |
| *Canis familiaris* (domestic dog) | Ecuador | 97 | 5 | < 7 yrs | Pet | CADT/PCR | [477] |  |
| *Canis familiaris* (domestic dog) | Canada | 155 | 95 | None | Ground | CM/PCR | [478] | ❶ |
| *Canis familiaris* (domestic dog) | Mexico | 147 | 10 | 6 groups | Kennel | CM/PCR | [479] |  |
| *Canis familiaris* (domestic dog) | Brazil | 46 | 1 | None | Stray | CM | [480] |  |
| *Canis familiaris* (domestic dog) | Italy | 14 | 9 | None | Stray | PCR | [481] |  |
| *Canis familiaris* (domestic dog) | USA | 120 | 10 | None | Sled racing | CADT | [482] |  |
| *Canis familiaris* (domestic dog) | Iran | 98 | 7 | 2 groups | Stray | CM | [483] |  |
| *Canis familiaris* (domestic dog) | Iran | 150 | 6 | 3 groups | Pet | CADT | [484] |  |
| *Canis familiaris* (domestic dog) | South Africa | 240 | 13 | > 5 m | Stray | CM | [485] |  |
| *Canis familiaris* (domestic dog) | Canada | 1,866 | 299 | None | Clinic | CADT | [486] |  |
| *Canis familiaris* (domestic dog) | Poland | 148 | 3 | None | Pe/Sh | CM/PCR | [487] |  |
| *Canis familiaris* (domestic dog) | Argentina | 1,944 | 25 | None | U/R/Pe/St | CM | [488] |  |
| *Canis familiaris* (domestic dog) | UK | 878 | 87 | 2 groups | Shelter | CADT/PCR | [489] |  |
| *Canis familiaris* (domestic dog) | Japan | 77 | 2 | < 15 yrs | Pet | CM/PCR | [490] |  |
| *Canis familiaris* (domestic dog) | Romania | 1,500 | 45 | 6 groups | Pet | CM | [491] |  |
| *Canis familiaris* (domestic dog) | Iran | 112 | 21 | 2 groups | Stray | CM | [492] |  |
| *Canis familiaris* (domestic dog) | Poland | 108 | 30 | 3 groups | Kennel | CADT/PCR | [493] |  |
| *Canis familiaris* (domestic dog) | Germany | 24,677 | 4,591 | None | Pet | CADT | [494] |  |
| *Canis familiaris* (domestic dog) | Canada | 75 | 10 | 3 m–15 yrs | Pet | CADT | [495] |  |
| *Canis familiaris* (domestic dog) | USA | 519,585 | 35,172 | None | Pet | CM | [496] |  |
| *Canis familiaris* (domestic dog) | Portugal | 126 | 23 | None | Pe/Ke | CM/PCR | [497] |  |
| *Canis familiaris* (domestic dog) | Japan | 1,794 | 420 | ≤ 3 m | Pet shop | CADT/PCR | [498] |  |
| *Canis familiaris* (domestic dog) | Japan | 2,365 | 196 | 2 groups | Pet | CADT | [499] |  |
| *Canis familiaris* (domestic dog) | Canada | 619 | 50 | 3 groups | Pe/Sh | CM | [500] |  |
| *Canis familiaris* (domestic dog) | Argentina | 46 | 5 | 2 groups | Pe/St | CM | [501] |  |
| *Canis familiaris* (domestic dog) | Spain | 544 | 221 | None | Shelter | CM | [502] |  |
| *Canis familiaris* (domestic dog) | Costa Rica | 58 | 5 | 2 groups | Pet | CADT/PCR | [503] |  |
| *Canis familiaris* (domestic dog) | Côte d’Ivoire | 11 | 6 | None | R/St | PCR | [264] |  |
| *Canis familiaris* (domestic dog) | Germany | 341 | 39 | 2 groups | Pe/St/Sh | CADT | [504] |  |
| *Canis familiaris* (domestic dog) | Spain | 604 | 96 | None | Shelter | CM/PCR | [505] |  |
| *Canis familiaris* (domestic dog) | China | 209 | 23 | 3 groups | Pet | PCR | [506] |  |
| *Canis familiaris* (domestic dog) | Nigeria | 40 | 1 | None | Slaughtered | CM | [507] |  |
| *Canis familiaris* (domestic dog) | Romania | 416 | 144 | 2 groups | U/R/Sh/Ke | CADT | [508] |  |
| *Canis familiaris* (domestic dog) | USA | 182 | 16 | None | U/R | CADT/PCR | [109] |  |
| *Canis familiaris* (domestic dog) | Peru | 130 | 19 | 4 groups | R/St | CM | [509] |  |
| *Canis familiaris* (domestic dog) | Brazil | 300 | 52 | None | Ho/St/Ke | CM/PCR | [510] |  |
| *Canis familiaris* (domestic dog) | Canada | 231 | 40 | None | Pet | PCR | [511] |  |
| *Canis familiaris* (domestic dog) | USA | 100 | 18 | 2 groups | Shelter | PCR | [512] |  |
| *Canis familiaris* (domestic dog) | USA | 129 | 5 | < 14 yrs | Pet | CM/PCR | [513] |  |
| *Canis familiaris* (domestic dog) | Brazil | 20 | 4 | None | Pet | CM/PCR | [119] |  |
| *Canis familiaris* (domestic dog) | Brazil | 195 | 33 | None | Pet | CM | [514] |  |
| *Canis familiaris* (domestic dog) | China | 205 | 27 | 2 groups | FD/Po | PCR | [515] |  |
| *Canis familiaris* (domestic dog) | Trinidad | 104 | 26 | 2 groups | Pe/Sh/St | PCR | [516] |  |
| *Canis familiaris* (domestic dog) | USA | 2,468,359 | 10,843 | 4 groups | Pet | CM | [517] |  |
| *Canis familiaris* (domestic dog) | Australia | 32 | 1 | None | Catchment | PCR | [518] | ❶ |
| *Canis familiaris* (domestic dog) | Venezuela | 98 | 14 | None | Pet | CM | [519] |  |
| *Canis familiaris* (domestic dog) | Brazil | 357 | 19 | 6 groups | Stray | CM | [520] |  |
| *Canis familiaris* (domestic dog) | Italy | 239 | 9 | None | Pet | CADT/PCR | [521] |  |
| *Canis familiaris* (domestic dog) | India | 120 | 49 | 4 groups | Pet | CADT | [522] |  |
| *Canis familiaris* (domestic dog) | Peru | 300 | 50 | < 12 yrs | Pet | CM | [523] |  |
| *Canis familiaris* (domestic dog) | Canada | 209 | 61 | < 1 yr | Sh/PS/Pe | CADT/PCR | [524] |  |
| *Canis familiaris* (domestic dog) | Egypt | 180 | 21 | 3 groups | Pe/Po | CM | [525] |  |
| *Canis familiaris* (domestic dog) | Brazil | 147 | 16 | None | Pet | PCR | [526] |  |
| *Canis familiaris* (domestic dog) | Iran | 210 | 2 | 2 groups | Pet | CM | [527] |  |
| *Canis familiaris* (domestic dog) | Iran | 100 | 9 | 2 groups | Stray | CM | [528] |  |
| *Canis familiaris* (domestic dog) | USA | 672 | 196 | None | Pet | CADT | [529] |  |
| *Canis familiaris* (domestic dog) | China | 31 | 4 | None | FD | CADT | [11] |  |
| *Canis familiaris* (domestic dog) | Bangladesh | 61 | 26 | 2 groups | Pet | CM | [530] |  |
| *Canis familiaris* (domestic dog) | Brazil | 80 | 34 | 2 groups | Pet | CM | [531] |  |
| *Canis familiaris* (domestic dog) | Portugal | 368 | 43 | 6 groups | Pet | CM | [532] |  |
| *Canis familiaris* (domestic dog) | Spain | 169 | 64 | None | Sh/Hu | CM/PCR | [533] |  |
| *Canis familiaris* (domestic dog) | Italy | 655 | 172 | 6 groups | Pe/Ke | CM/PCR | [534] |  |
| *Canis familiaris* (domestic dog) | Canada | 251 | 16 | 2 groups | Pet | CADT | [535] |  |
| *Canis familiaris* (domestic dog) | Cambodia | 94 | 2 | 3 groups | Pet | CM | [536] |  |
| *Canis familiaris* (domestic dog) | Canada | 251 | 62 | 2 groups | Pet | CADT | [537] |  |
| *Canis familiaris* (domestic dog) | Taiwan | 118 | 11 | None | Stray | PCR | [538] |  |
| *Canis familiaris* (domestic dog) | China | 318 | 51 | 3 groups | Pe/St | CADT | [539] |  |
| *Canis familiaris* (domestic dog) | Italy | 208 | 42 | 2 groups | Pet | CADT/PCR | [540] |  |
| *Canis familiaris* (domestic dog) | China | 216 | 21 | None | Shelter | PCR | [541] |  |
| *Canis familiaris* (domestic dog) | Mexico | 101 | 46 | 3 groups | Shelter | PCR | [542] |  |
| *Canis familiaris* (domestic dog) | India | 18 | 12 | None | Stray | CADT | [145] | ❺ |
| *Canis familiaris* (domestic dog) | China | 315 | 10 | 2 groups | Pet | PCR | [543] |  |
| *Canis familiaris* (domestic dog) | China | 267 | 12 | 3 groups | Pe/St/PS | PCR | [544] |  |
| *Canis familiaris* (domestic dog) | Russia | 97 | 0 | None | Pet | CM | [545] |  |
| *Canis familiaris* (domestic dog) | Italy | 502 | 41 | None | Pe/St | CM/PCR | [546] |  |
| *Canis familiaris* (domestic dog) | Iran | 1,500 | 20 | 3 groups | Ho/St | CM | [547] |  |
| *Canis familiaris* (domestic dog) | South Korea | 202 | 67 | 2 groups | Shelter | PCR | [548] |  |
| *Canis familiaris* (domestic dog) | Italy | 285 | 165 | 3 groups | Kennel | PCR | [549] |  |
| *Canis familiaris* (domestic dog) | Poland | 36 | 7 | None | Ho | CADT/PCR | [146] |  |
| *Canis familiaris* (domestic dog) | Canada | 1,085 | 38 | 2 groups | Shelter | CM | [550] |  |
| *Canis familiaris* (domestic dog) | Nigeria | 203 | 0 | None | Ground | CM | [551] | ❶ |
| *Canis familiaris* (domestic dog) | Brazil | 3,099 | 169 | 2 groups | Pet | CM | [552] |  |
| *Canis familiaris* (domestic dog) | USA | 300 | 27 | < 17 yrs | Pet | CADT | [553] |  |
| *Canis familiaris* (domestic dog) | Australia | 18 | 0 | None | Catchment | PCR | [328] |  |
| *Canis familiaris* (domestic dog) | Poland | 128 | 27 | < 10 yrs | Pet | CADT/PCR | [554] |  |
| *Canis familiaris* (domestic dog) | Brazil | 20 | 0 | None | Pet | PCR | [555] |  |
| *Canis familiaris* (domestic dog) | China | 940 | 134 | 3 groups | Pe/St | CM/PCR | [556] |  |
| *Canis familiaris* (domestic dog) | Brazil | 108 | 10 | 3 groups | Pet | CM/PCR | [557] |  |
| *Canis familiaris* (domestic dog) | Ecuador | 40 | 5 | None | Farm dog | PCR | [294] |  |
| *Canis familiaris* (domestic dog) | China | 485 | 127 | 2 groups | Pe/PS | PCR | [558] |  |
| *Canis familiaris* (domestic dog) | Jamaica | 225 | 44 | None | Pet | PCR | [559] |  |
| *Canis familiaris* (domestic dog) | Iraq | 93 | 4 | 2 groups | Pet | CM | [560] |  |
| *Canis familiaris* (domestic dog) | Poland | 207 | 37 | None | R/Ho | CM | [561] |  |
| *Canis familiaris* (domestic dog) | Spain | 55 | 16 | None | U/R/Pet | PCR | [562] |  |
| *Canis familiaris* (domestic dog) | Spain | 194 | 63 | 3 groups | Shelter | PCR | [563] |  |
| *Canis familiaris* (domestic dog) | Australia | 300 | 9 | None | Parks | CM | [564] | ❶ |
| *Canis familiaris* (domestic dog) | Iran | 450 | 6 | None | Parks | CM | [565] | ❶ |
| *Canis familiaris* (domestic dog) | Greece | 879 | 222 | 7 groups | Sh/Ho/SP | CADT/PCR | [566] |  |
| *Canis familiaris* (domestic dog) | China | 19 | 6 | None | Pet | PCR | [15] |  |
| *Canis familiaris* (domestic dog) | Cuba | 98 | 11 | None | Pe/Sh | PCR | [567] |  |
| *Canis familiaris* (domestic dog) | Germany | 2,731 | 16 | None | Pet | CM | [162] |  |
| *Canis familiaris* (domestic dog) | Italy | 705 | 204 | None | Parks | PCR | [568] | ❶ |
| *Canis familiaris* (domestic dog) | Poland | 95 | 0 | 2 groups | Shelter | CM | [569] |  |
| *Canis familiaris* (domestic dog) | Portugal | 369 | 42 | None | Pet | CM | [570] |  |
| *Canis familiaris* (domestic dog) | Thailand | 109 | 21 | 2 groups | Pe/Sh | PCR | [571] |  |
| *Canis familiaris* (domestic dog) | China | 159 | 18 | None | Stray | PCR | [572] |  |
| *Canis familiaris* (domestic dog) | Spain | 348 | 127 | None | Pe/Sh/Hu/SP | CADT/PCR | [573] |  |
| *Canis familiaris* (domestic dog) | Italy | 262 | 56 | 3 groups | St/Sh | CADT/PCR | [574] |  |
| *Canis familiaris* (domestic dog) | Cuba | 293 | 10 | 2 groups | Pe/St | CM | [575] |  |
| *Canis familiaris* (domestic dog) | Iran | 301 | 31 | None | R/St/Ho | CM | [576] |  |
| *Canis familiaris* (domestic dog) | China | 527 | 57 | 2 groups | Stray | CM/PCR | [577] |  |
| *Canis familiaris* (domestic dog) | Italy | 639 | 31 | None | Kennel | CADT/PCR | [578] |  |
| *Canis familiaris* (domestic dog) | Brazil | 22 | 0 | None | Pet | CM | [178] |  |
| *Canis familiaris* (domestic dog) | Italy | 2,775 | 194 | None | Sh/Pe | CM | [579] |  |
| *Canis familiaris* (domestic dog) | Germany | 376 | 115 | 5 groups | Pe/Sh | CADT/PCR | [580] |  |
| *Canis familiaris* (domestic dog) | Netherlands | 646 | 189 | None | Pe/Sh/Hu | PCR | [581] |  |
| *Canis familiaris* (domestic dog) | India | 212 | 49 | None | Parks | CADT/PCR | [582] | ❸ |
| *Canis familiaris* (domestic dog) | China | 485 | 54 | 3 groups | Pe/PS/FD | PCR | [583] |  |
| *Canis familiaris* (domestic dog) | Ireland | 615 | 37 | 3 groups | Stray | CM | [584] |  |
| *Canis familiaris* (domestic dog) | Poland | 257 | 18 | 2 groups | Pet | CM | [585] |  |
| *Canis familiaris* (domestic dog) | Mexico | 402 | 103 | 2 groups | Sh/St | CM/PCR | [586] |  |
| *Canis familiaris* (domestic dog) | Iran | 315 | 6 | 2 groups | Pet | CM/PCR | [587] |  |
| *Canis familiaris* (domestic dog) | Canada | 294 | 13 | None | Sl/Sh/Co | CADT/PCR | [588] |  |
| *Canis familiaris* (domestic dog) | South Korea | 640 | 99 | 2 groups | Sh/Pe/Hu | PCR | [589] |  |
| *Canis familiaris* (domestic dog) | Russia | 1,788 | 172 | None | Pet | CM | [590] |  |
| *Canis familiaris* (domestic dog) | China | 641 | 60 | 2 groups | Pe/Sh/PS | PCR | [591] |  |
| *Canis familiaris* (domestic dog) | Colombia | 1,111 | 145 | 2 groups | Pet | CM | [592] |  |
| *Canis familiaris* (domestic dog) | Mozambique | 156 | 13 | < 6 m | Pet | CADT | [183] |  |
| *Canis familiaris* (domestic dog) | Nicaragua | 58 | 13 | 3 groups | Hunting | PCR | [593] |  |
| *Canis familiaris* (domestic dog) | USA | 261 | 118 | None | Pe/Sh | CADT | [594] |  |
| *Canis familiaris* (domestic dog) | Thailand | 301 | 76 | 3 groups | Sh/BS | CADT/PCR | [595] |  |
| *Canis familiaris* (domestic dog) | Israel | 163 | 19 | 2 groups | Pe/Ke | CADT | [596] |  |
| *Canis familiaris* (domestic dog) | China | 651 | 20 | 2 groups | Pe/Sh/PS/BS | PCR | [597] |  |
| *Canis familiaris* (domestic dog) | Italy | 47 | 16 | None | Pet | CADT/PCR | [598] |  |
| *Canis familiaris* (domestic dog) | Colombia | 18 | 0 | None | Pet | CM | [205] |  |
| *Canis familiaris* (domestic dog) | Canada | 860 | 64 | None | Ground | CADT/PCR | [599] | ❸ |
| *Canis familiaris* (domestic dog) | Israel | 302 | 74 | 3 groups | Shelter | PCR | [600] |  |
| *Canis familiaris* (domestic dog) | Madagascar | 41 | 12 | None | Free roam | CADT | [240] |  |
| *Canis familiaris* (domestic dog) | Brazil | 147 | 10 | 2 groups | Pet | CM/PCR | [601] |  |
| *Canis familiaris* (domestic dog) | Spain | 233 | 7 | None | FD/St | CM | [602] |  |
| *Canis familiaris* (domestic dog) | Poland | 217 | 13 | None | Pet | PCR | [603] |  |
| *Canis familiaris* (domestic dog) | Uganda | 2 | 0 | None | Free roam | CADT | [210] | ❸ |
| *Canis familiaris* (domestic dog) | Ecuador | 83 | 4 | None | Free roam | CM | [351] |  |
| *Canis familiaris* (domestic dog) | Vietnam | 3 | 2 | None | Ho | PCR | [209] | ❸ |
| *Canis familiaris* (domestic dog) | Bangladesh | 2 | 0 | None | Captive | PCR | [3] | ❸ |
| *Canis familiaris* (domestic dog) | Argentina | 36 | 16 | None | Ho | PCR | [212] |  |
| *Canis familiaris* (domestic dog) | Slovakia | 257 | 52 | 2 groups | Ho | CM | [604] |  |
| *Canis familiaris* (domestic dog) | Egypt | 986 | 86 | 3 groups | Stray | CM/PCR | [605] |  |
| *Canis familiaris* (domestic dog) | Serbia | 282 | 45 | None | Parks | CM | [606] | ❸ |
| *Canis familiaris* (domestic dog) | Palestine | 150 | 5 | 2 groups | Pe/St | CM | [607] |  |
| *Canis familiaris* (domestic dog) | Portugal | 80 | 27 | 3 groups | Pe/Sh | CM/PCR | [608] |  |
| *Canis familiaris* (domestic dog) | USA | 4,692 | 391 | None | Pe/Sh | CM | [609] |  |
| *Canis familiaris* (domestic dog) | Brazil | 230 | 13 | None | Pet | CM | [610] |  |
| *Canis familiaris* (domestic dog) | Sweden | 303 | 8 | None | Pet | CM | [611] |  |
| *Canis familiaris* (domestic dog) | Italy | 168 | 69 | < 17 yrs | Shelter | CADT | [612] |  |
| *Canis familiaris* (domestic dog) | China | 105 | 4 | None | Pet | PCR | [225] |  |
| *Canis familiaris* (domestic dog) | China | 682 | 8 | None | Ground | PCR | [613] | ❸ |
| *Canis familiaris* (domestic dog) | Serbia | 367 | 88 | None | Pet | CM | [614] |  |
| *Canis familiaris* (domestic dog) | China | 448 | 32 | 3 groups | Pet | PCR | [615] |  |
| *Canis familiaris* (domestic dog) | China | 604 | 22 | None | Pet | PCR | [616] |  |
| *Canis familiaris* (domestic dog) | UK | 52 | 19 | None | Pet | PCR | [617] | ❹ |
| *Canis familiaris* (domestic dog) | Germany | 171 | 87 | < 1 yr | Pe/Sh | PCR | [618] |  |
| *Canis familiaris* (domestic dog) | Croatia | 285 | 78 | 2 groups | Pe/Sh | CADT | [619] |  |
| *Canis familiaris* (domestic dog) | Brazil | 139 | 24 | None | Ho | CM/PCR | [620] |  |
| *Canis familiaris* (domestic dog) | Iran | 246 | 7 | 2 groups | Pe/St/Sh | CM/PCR | [621] |  |
| *Canis familiaris* (domestic dog) | Iran | 3 | 0 | None | Stray | CM | [229] | ❸ |
| *Canis familiaris* (domestic dog) | Russia | 2,208 | 226 | 2 groups | Pet | CM | [622] |  |
| *Canis familiaris* (domestic dog) | USA | 170 | 51 | < 13 yrs | Research | PCR | [623] |  |
| *Canis familiaris* (domestic dog) | Egypt | 218 | 84 | None | Ho | PCR | [624] |  |
| *Canis familiaris* (domestic dog) | Ireland | 789 | 205 | 4 groups | Shelter | CADT | [625] |  |
| *Canis familiaris* (domestic dog) | China | 99 | 18 | None | Pet | PCR | [626] |  |
| *Canis familiaris* (domestic dog) | Spain | 252 | 106 | None | Pet | CADT/PCR | [627] |  |
| *Canis familiaris* (domestic dog) | Brazil | 359 | 12 | None | Pet | CM | [628] |  |
| *Canis familiaris* (domestic dog) | Austria | 70 | 7 | None | Shelter | CADT | [629] |  |
| *Canis familiaris* (domestic dog) | Portugal | 46 | 11 | None | Pet | PCR | [235] |  |
| *Canis familiaris* (domestic dog) | Iraq | 75 | 11 | None | Pet | CM/PCR | [630] |  |
| *Canis familiaris* (domestic dog) | Taiwan | 250 | 31 | None | Pet | PCR | [631] |  |
| *Canis familiaris* (domestic dog) | Turkey | 33 | 18 | None | Stray | CADT/PCR | [632] |  |
| *Canis familiaris dingo* (dingo) | Australia | 44 | 13 | None | Wild | PCR | [93] |  |
| *Canis latrans* (coyote) | USA | 3 | 0 | None | Wild | CM | [306] | ❸ |
| *Canis latrans* (coyote) | Canada | 99 | 5 | None | Wild | CADT | [7] |  |
| *Canis latrans* (coyote) | Japan | 2 | 0 | None | Captive | CADT | [258] | ❸ |
| *Canis latrans* (coyote) | USA | 22 | 7 | None | Wild | PCR | [633] |  |
| *Canis latrans (coyote)* | Canada | 70 | 13 | None | Wild | CADT/PCR | [634] |  |
| *Canis latrans* (coyote) | USA | 18 | 7 | None | Wild | CADT/PCR | [109] |  |
| *Canis latrans* (coyote) | Australia | 1 | 0 | None | Wild | PCR | [518] | ❸ |
| *Canis latrans* (coyote) | Canada | 193 | 15 | None | Wild | CADT/PCR | [599] |  |
| *Canis lupus* (grey wolf) | USA | 9 | 0 | None | Wild | CM | [306] |  |
| *Canis lupus* (grey wolf) | Canada | 2 | 0 | None | Wild | CADT | [7] | ❸ |
| *Canis lupus* (grey wolf) | Poland | 30 | 14 | None | Wild | CM | [635] |  |
| *Canis lupus* (grey wolf) | Japan | 3 | 0 | None | Captive | CADT | [258] | ❸ |
| *Canis lupus* (grey wolf) | Poland | 5 | 1 | None | Wild | CADT | [9] |  |
| *Canis lupus* (grey wolf) | Poland | 14 | 0 | None | Wild | CADT | [10] |  |
| *Canis lupus* (grey wolf) | Croatia | 9 | 4 | None | Captive | CADT/PCR | [1] |  |
| *Canis lupus* (grey wolf) | Croatia | 127 | 13 | None | Wild | CADT/PCR | [311] |  |
| *Canis lupus* (grey wolf) | Poland | 7 | 2 | None | Wild | PCR | [310] |  |
| *Canis lupus* (grey wolf) | China | 2 | 1 | None | Captive | PCR | [15] | ❸ |
| *Canis lupus* (grey wolf) | China | 4 | 0 | None | Captive | PCR | [5] | ❸ |
| *Canis lupus* (grey wolf) | China | 7 | 0 | None | Captive | PCR | [6] |  |
| *Canis lupus* (grey wolf) | Italy | 79 | 2 | None | Wild | CADT | [636] |  |
| *Canis lupus* *arctos* (arctic wolf) | Serbia | 4 | 0 | None | Captive | PCR | [8] | ❸ |
| *Canis lupus* *signatus* (Iberian wolf) | Spain | 6 | 1 | None | Wild | PCR | [637] |  |
| *Canis lupus* *signatus* (Iberian wolf) | Portugal | 121 | 31 | None | Wild | PCR | [235] |  |
| *Chrysocyon brachyurus* (maned wolf) | Croatia | 2 | 1 | None | Captive | CADT/PCR | [1] | ❸ |
| *Chrysocyon brachyurus* (maned wolf) | Brazil | 3 | 0 | None | Captive | CADT | [638] | ❸ |
| *Lupulella mesomelas* (black-backed jackal) | Japan | 2 | 0 | None | Captive | CADT | [258] | ❸ |
| *Lycaon pictus* (African wild dog) | Zambia | 43 | 12 | None | Wild | PCR | [639] |  |
| *Lycaon pictus* (African wild dog) | Namibia | 28 | 7 | None | Wild | PCR | [639] |  |
| *Lycaon pictus* (African wild dog) | Australia | 16 | 10 | None | Captive | PCR | [639] |  |
| *Lycaon pictus* (African wild dog) | China | 7 | 3 | None | Captive | PCR | [6] |  |
| *Nyctereutes procyonoides* (raccoon dog) | Poland | 2 | 0 | None | Captive | CM | [2] | ❸ |
| *Nyctereutes procyonoides* (raccoon dog) | Japan | 1 | 0 | None | Captive | CADT | [258] | ❸ |
| *Nyctereutes procyonoides* (raccoon dog) | Poland | 18 | 2 | None | Farmed | CM/PCR | [640] |  |
| *Nyctereutes procyonoides* (raccoon dog) | China | 305 | 22 | 2 groups | Farmed | PCR | [641] |  |
| *Nyctereutes procyonoides* (raccoon dog) | China | 16 | 1 | None | Pet | PCR | [225] |  |
| *Cerdocyon thous* (crab-eating fox) | Brazil | 126 | 4 | None | Captive | CM | [320] |  |
| *Vulpes lagopus* (Arctic fox) | Poland | 2 | 0 | None | Captive | CM | [2] | ❸ |
| *Vulpes lagopus* (Arctic fox) | Canada | 95 | 15 | None | Wild | CADT | [642] |  |
| *Vulpes lagopus* (Arctic fox) | USA | 109 | 20 | None | Wild | CADT | [327] |  |
| *Vulpes corsac* (*corsac fox*) | Poland | 1 | 0 | None | Captive | CM | [2] | ❸ |
| *Vulpes vulpes* (red fox) | Poland | 3 | 0 | None | Captive | CM | [2] | ❸ |
| *Vulpes vulpes* (red fox) | Norway | 269 | 13 | 2 groups | Wild | CADT/PCR | [643] |  |
| *Vulpes vulpes* (red fox) | Australia | 19 | 6 | None | Wild | PCR | [93] |  |
| *Vulpes vulpes* (red fox) | Croatia | 66 | 3 | None | Wild | CADT/PCR | [311] |  |
| *Vulpes vulpes* (red fox) | Australia | 23 | 0 | None | Wild | PCR | [518] |  |
| *Vulpes vulpes* (red fox) | Bosnia | 123 | 9 | None | Wild | CADT | [644] |  |
| *Vulpes vulpes* (red fox) | Poland | 21 | 4 | None | Wild | CADT | [310] |  |
| *Vulpes vulpes* (red fox) | Romania | 217 | 10 | 2 groups | Wild | PCR | [645] |  |
| *Vulpes vulpes* (red fox) | Australia | 9 | 0 | None | Wild | PCR | [328] |  |
| *Vulpes vulpes* (red fox) | Poland | 80 | 0 | None | Farmed | CM | [640] |  |
| *Vulpes vulpes* (red fox) | China | 1 | 0 | None | Captive | PCR | [15] | ❸ |
| *Vulpes vulpes* (red fox) | Spain | 87 | 7 | None | Wild | PCR | [637] |  |
| *Vulpes vulpes* (red fox) | Sweden | 104 | 46 | None | Wild | CADT/PCR | [646] |  |
| *Vulpes vulpes* (red fox) | Italy | 71 | 5 | 2 groups | Wild | CADT | [647] |  |
| *Vulpes vulpes* (red fox) | Spain | 197 | 19 | None | Wild | PCR | [648] |  |
| *Vulpes vulpes* (red fox) | China | 1 | 0 | None | Captive | PCR | [5] | ❸ |
| *Vulpes vulpes* (red fox) | Portugal | 118 | 22 | None | Wild | PCR | [235] |  |
| *Vulpes vulpes* (red fox) | Italy | 101 | 9 | None | Wild | CADT | [636] |  |
| *Vulpes zerda* (fennec fox) | Croatia | 1 | 0 | None | Captive | CADT | [1] | ❸ |
| *Vulpes zerda* (fennec fox) | China | 1 | 0 | None | Captive | PCR | [5] | ❸ |
| *Vulpes zerda* (fennec fox) | China | 2 | 0 | None | Captive | PCR | [6] | ❸ |
| *Vulpes zerda* (fennec fox) | Serbia | 2 | 0 | None | Captive | PCR | [8] | ❸ |
| **Family Mephitidae** |  |  |  |  |  |  |  |  |
| *Mephitis mephitis* (striped skunk) | Poland | 2 | 0 | None | Captive | CM | [2] | ❸ |
| **Family Mustelidae** |  |  |  |  |  |  |  |  |
| *Aonyx cinereus* (Asian small-clawed otter) | Croatia | 1 | 0 | None | Captive | CADT | [1] | ❸ |
| *Enhydra lutris* (sea otter) | USA | 103 | 1 | None | Wild | CADT | [109] |  |
| *Lutra lutra* (Eurasian otter) | Poland | 1 | 0 | None | Captive | CM | [2] | ❸ |
| *Lutra lutra* (Eurasian otter) | Spain | 437 | 30 | None | Wild | CADT | [649] |  |
| *Lutra lutra* (Eurasian otter) | Poland | 1 | 0 | None | Wild | PCR | [310] | ❸ |
| *Lutra lutra* (Eurasian otter) | Spain | 2 | 0 | None | Wild | PCR | [637] | ❸ |
| *Martes foina* (beech marten) | Spain | 8 | 1 | None | Wild | PCR | [637] |  |
| *Martes foina* (beech marten) | Portugal | 19 | 3 | None | Wild | PCR | [235] |  |
| *Martes* sp. (marten) | Canada | 4 | 0 | None | Wild | CADT | [7] | ❸ |
| *Martes* sp. (marten) | Poland | 1 | 0 | None | Wild | PCR | [310] | ❸ |
| *Meles meles* (Eurasian badger) | Poland | 1 | 0 | None | Captive | CM | [2] | ❸ |
| *Meles meles* (Eurasian badger) | Poland | 1 | 0 | None | Wild | PCR | [310] | ❸ |
| *Meles meles* (Eurasian badger) | Spain | 70 | 0 | None | Wild | PCR | [637] |  |
| *Meles meles* (Eurasian badger) | Italy | 43 | 21 | None | Wild | CADT/PCR | [650] |  |
| *Mustela furo* (ferret) | Spain | 2 | 0 | None | Wild | PCR | [637] | ❸ |
| *Mustela nigripes* (black-footed ferret) | USA | 6 | 1 | None | Captive | CM | [651] |  |
| *Mustela putorius* (European polecat) | Spain | 2 | 0 | None | Wild | PCR | [637] | ❸ |
| *Mustela putorius* *furo* (domestic ferret) | New Zealand | 3 | 1 | None | Wild | CM | [652] | ❸ |
| *Mustela putorius* *furo* (domestic ferret) | Germany | 68 | 2 | None | Pet | CADT | [653] |  |
| *Mustela putorius* *furo* (domestic ferret) | Germany | 196 | 26 | None | Pet | CADT | [654] |  |
| *Mustela putorius* *furo* (domestic ferret) | Russia | 323 | 5 | None | Pet | CM | [590] |  |
| *Mustela putorius* *furo* (domestic ferret) | UAE | 1 | 0 | None | Captive | PCR | [218] | ❸ |
| *Mustela* sp. (weasel) | Poland | 1 | 0 | None | Wild | PCR | [310] | ❸ |
| *Pekania pennanti* (fisher) | Canada | 3 | 0 | None | Wild | CADT | [7] | ❸ |
| **Family Odobenidae** |  |  |  |  |  |  |  |  |
| Odobenus rosmarus (walrus) | USA | 61 | 0 | None | Wild | CADT | [327] |  |
| **Family Otariidae** |  |  |  |  |  |  |  |  |
| *Neophoca cinerea* (Australian sea lion) | Australia | 290 | 35 | None | Ca/Wi | PCR | [655] |  |
| *Zalophus californianus* (California sea lion) | Japan | 1 | 0 | None | Captive | CADT | [258] | ❸ |
| *Zalophus californianus* (California sea lion) | Croatia | 4 | 0 | None | Captive | CADT | [1] | ❸ |
| **Family Phocidae** |  |  |  |  |  |  |  |  |
| *Cystophora cristata* (hooded seal) | Canada | 10 | 0 | None | Wild | CADT | [656] |  |
| *Erignathus barbatus* (bearded seal) | Alaska | 22 | 0 | None | Wild | CADT | [376] |  |
| *Erignathus barbatus* (bearded seal) | Canada | 4 | 3 | 2 groups | Wild | CADT | [657] | ❸ |
| *Halichoerus grypus* *atlantica* (grey seal) | Canada | 19 | 4 | None | Wild | CADT | [375] |  |
| *Halichoerus grypus* *atlantica* (grey seal) | USA | 27 | 17 | None | Wild | PCR | [378] |  |
| *Halichoerus grypus* *atlantica* (grey seal) | Croatia | 2 | 1 | None | Captive | CADT | [1] | ❸ |
| *Pagophilus groenlandicus* (harp seal) | Canada | 47 | 15 | None | Wild | CADT | [375] |  |
| *Pagophilus groenlandicus* (harp seal) | Canada | 58 | 16 | None | Wild | CADT | [656] |  |
| *Phoca hispida* (ringed seal) | Canada | 15 | 3 | None | Wild | CADT | [374] |  |
| *Phoca hispida* (ringed seal) | Alaska | 31 | 20 | None | Wild | CADT | [376] |  |
| *Phoca hispida* (ringed seal) | Canada | 55 | 44 | 3 groups | Wild | CADT/PCR | [657] |  |
| *Phoca vitulina* *richardsi* (pacific harbor seal) | USA | 97 | 41 | None | Wild | CADT/PCR | [658] |  |
| *Phoca vitulina* *richardsi* (pacific harbor seal) | USA | 112 | 5 | None | Wild | PCR | [378] |  |
| *Phoca vitulina vitulina* (Atlantic harbour seal) | Canada | 8 | 1 | None | Wild | CADT | [375] |  |
| *Phoca vitulina vitulina* (Atlantic harbour seal) | USA | 8 | 3 | None | Wild | PCR | [378] |  |
| **Family Procyonidae** |  |  |  |  |  |  |  |  |
| *Nasua nasua* (South American coati) | Poland | 2 | 0 | None | Captive | CM | [2] | ❸ |
| *Nasua nasua* (South American coati) | Brazil | 2 | 0 | None | Wild | CM | [659] | ❸ |
| *Nasua nasua* (South American coati) | Croatia | 4 | 1 | None | Captive | CADT/PCR | [1] | ❸ |
| *Nasua nasua* (South American coati) | Brazil | 1 | 0 | None | Captive | CADT | [638] | ❸ |
| *Nasua nasua* (South American coati) | Brazil | 27 | 3 | None | Captive | CADT | [660] |  |
| *Nasuella olivacea* (western dwarf coati) | China | 1 | 0 | None | Captive | PCR | [5] | ❸ |
| *Potos flavus* (Kinkajou) | Poland | 2 | 0 | None | Captive | CM | [2] | ❸ |
| *Potos flavus* (Kinkajou) | Brazil | 1 | 0 | None | Captive | CADT | [638] | ❸ |
| *Procyon cancrivorus* (South American raccoon) | Brazil | 1 | 0 | None | Wild | CM | [659] | ❸ |
| *Procyon cancrivorus* (South American raccoon) | Brazil | 2 | 0 | None | Captive | CADT | [638] | ❸ |
| *Procyon lotor* (raccoon) | Poland | 2 | 0 | None | Captive | CM | [2] | ❸ |
| *Procyon lotor* (raccoon) | China | 3 | 0 | None | Captive | PCR | [5] | ❸ |
| *Procyon lotor* (raccoon) | China | 1 | 0 | None | Captive | PCR | [6] | ❸ |
| *Procyon lotor* (raccoon) | Italy | 62 | 0 | None | Wild | CADT | [661] |  |
| **Family Ursidae** |  |  |  |  |  |  |  |  |
| *Ailuropoda melanoleuca* (panda bear) | China | 1 | 0 | None | Captive | PCR | [15] | ❸ |
| *Helarctos malayanus* (Malayan sun bear) | Japan | 2 | 0 | None | Captive | CADT | [258] | ❸ |
| *Helarctos malayanus* (Malayan sun bear) | Croatia | 2 | 1 | None | Captive | CADT/PCR | [1] | ❸ |
| *Helarctos malayanus* (Malayan sun bear) | China | 7 | 0 | None | Captive | PCR | [6] |  |
| *Tremarctos ornatus* (spectacled bear) | Peru | 28 | 4 | None | Wild | CM | [662] |  |
| *Ursus americanus* (American black bear) | USA | 10 | 0 | None | Wild | CM | [306] |  |
| *Ursus americanus* (American black bear) | USA | 3 | 0 | None | Wild | CM | [314] | ❸ |
| *Ursus americanus* (American black bear) | Croatia | 2 | 0 | None | Captive | CADT | [1] | ❸ |
| *Ursus arctos* (brown bear) | Poland | 2 | 0 | None | Captive | CM | [2] | ❸ |
| *Ursus arctos* (brown bear) | Greece | 8 | 0 | 2–30 yrs | Captive | CM | [34] |  |
| *Ursus arctos* (brown bear) | Croatia | 19 | 0 | None | Wild | CADT | [311] |  |
| *Ursus arctos* (brown bear) | Croatia | 2 | 0 | None | Captive | CADT | [1] | ❸ |
| *Ursus arctos arctos* (Eurasian brown bear) | Croatia | 94 | 4 | None | Wild | CM | [663] |  |
| *Ursus arctos* (brown bear) | China | 3 | 0 | None | Captive | PCR | [5] | ❸ |
| *Ursus arctos* (brown bear) | China | 6 | 0 | None | Captive | PCR | [6] |  |
| *Ursus arctos horribilis* (grizzly bear) | USA | 9 | 0 | None | Wild | CM | [306] |  |
| *Ursus maritimus* (polar bear) | Poland | 1 | 0 | None | Captive | CM | [2] | ❸ |
| *Ursus maritimus* (polar bear) | Japan | 3 | 0 | None | Captive | CADT | [258] | ❸ |
| *Ursus maritimus* (polar bear) | China | 2 | 0 | None | Captive | PCR | [6] | ❸ |
| *Ursus maritimus* (polar bear) | USA | 8 | 1 | None | Wild | CADT | [327] |  |
| *Ursus thibetanus* (Asian black bear) | Bangladesh | 4 | 0 | None | Captive | PCR | [3] | ❸ |
| *Ursus thibetanus* (Asian black bear) | China | 9 | 0 | None | Captive | PCR | [5] |  |
| *Ursus thibetanus* (Asian black bear) | China | 5 | 0 | None | Captive | PCR | [6] |  |
| *Ursus thibetanus* (Asian black bear) | China | 6 | 0 | None | Wild | PCR | [317] |  |
| **Family Felidae** |  |  |  |  |  |  |  |  |
| *Acinonyx jubatus* (cheetah) | Croatia | 2 | 2 | None | Captive | CADT/PCR | [1] | ❸ |
| *Acinonyx jubatus* (cheetah) | Serbia | 2 | 0 | None | Captive | PCR | [8] | ❸ |
| *Caracal caracal* (caracal) | Poland | 2 | 0 | None | Captive | CM | [2] | ❸ |
| *Felis catus* (domestic cat) | USA | 1 | 0 | None | Shelter | CM | [306] | ❸ |
| *Felis catus* (domestic cat) | Ausralia | 226 | 32 | 3 groups | Sh/Pe/BS | CM | [385] |  |
| *Felis catus* (domestic cat) | Australia | 50 | 8 | None | Shelter | CM | [386] |  |
| *Felis catus* (domestic cat) | USA | 452 | 16 | 2 groups | Pet | CM | [387] |  |
| *Felis catus* (domestic cat) | New Zealand | 192 | 10 | None | Pet | CM | [389] |  |
| *Felis catus* (domestic cat) | Belgium | 30 | 0 | None | Stray | CM | [390] |  |
| *Felis catus* (domestic cat) | Germany | 1,147 | 28 | None | Pet | CADT | [391] |  |
| *Felis catus* (domestic cat) | USA | 2,000 | 48 | < 20 yrs | Pet | CM | [396] |  |
| *Felis catus* (domestic cat) | USA | 206 | 5 | 5 groups | Pe/Sh | CM | [664] |  |
| *Felis catus* (domestic cat) | USA | 263 | 19 | < 1 yr | Pe/Sh | CM | [665] |  |
| *Felis catus* (domestic cat) | Serbia | 81 | 18 | 2 groups | Pet | CM | [404] |  |
| *Felis catus* (domestic cat) | Germany | 3,167 | 400 | None | Pet | CADT | [408] |  |
| *Felis catus* (domestic cat) | Australia | 40 | 32 | 2 groups | Pe/PS/Re/BS | PCR | [666] |  |
| *Felis catus* (domestic cat) | Brazil | 131 | 8 | None | Pe/St | CM | [667] |  |
| *Felis catus* (domestic cat) | Italy | 48 | 2 | 2 groups | Pe/St | CADT | [416] |  |
| *Felis catus* (domestic cat) | Italy | 1 | 1 | None | Kennel | CM/PCR | [56] | ❸ |
| *Felis catus* (domestic cat) | Germany | 98 | 22 | None | Shelter | CADT | [417] |  |
| *Felis catus* (domestic cat) | USA | 117 | 36 | 2 groups | Cattery | CADT | [668] |  |
| *Felis catus* (domestic cat) | Germany | 441 | 5 | None | Pet | CADT | [52] |  |
| *Felis catus* (domestic cat) | Iran | 166 | 10 | None | Stray | CM/PCR | [669] |  |
| *Felis catus* (domestic cat) | USA | 4,977 | 538 | None | Pet | CADT | [429] |  |
| *Felis catus* (domestic cat) | USA | 211,105 | 1,223 | 5 groups | Pet | CM | [670] |  |
| *Felis catus* (domestic cat) | Japan | 600 | 240 | 4 groups | U/R/Pe/PS | CADT | [671] |  |
| *Felis catus* (domestic cat) | Chile | 230 | 44 | 2 groups | Pet | CM | [433] |  |
| *Felis catus* (domestic cat) | Colombia | 46 | 3 | 3 groups | Stray | PCR | [672] |  |
| *Felis catus* (domestic cat) | Canada | 8,160 | 12 | 2 groups | Pet | CM | [434] |  |
| *Felis catus* (domestic cat) | Canada | 41 | 1 | 2 groups | Pet | CM | [434] |  |
| *Felis catus* (domestic cat) | Brazil | 288 | 10 | 2 groups | Pet | CM | [440] |  |
| *Felis catus* (domestic cat) | USA | 354 | 34 | None | Shelter | CADT | [673] |  |
| *Felis catus* (domestic cat) | Italy | 76 | 2 | 4 groups | Stray | CM | [674] |  |
| *Felis catus* (domestic cat) | Italy | 266 | 42 | 3 groups | Pe/St | CADT | [675] |  |
| *Felis catus* (domestic cat) | USA | 250 | 34 | 5 groups | Pet | CADT/PCR | [676] |  |
| *Felis catus* (domestic cat) | Brazil | 1 | 1 | None | Pet | PCR | [451] | ❸ |
| *Felis catus* (domestic cat) | Brazil | 327 | 27 | None | Not stated | CM | [452] |  |
| *Felis catus* (domestic cat) | Iran | 112 | 0 | None | Stray | CM | [455] |  |
| *Felis catus* (domestic cat) | Brazil | 462 | 72 | 4 groups | Pet | CADT | [457] |  |
| *Felis catus* (domestic cat) | Australia | 1,063 | 21 | 2 groups | Pe/St | CM | [461] |  |
| *Felis catus* (domestic cat) | UK | 1,355 | 74 | None | Pet | CM | [677] |  |
| *Felis catus* (domestic cat) | UK | 93 | 9 | None | Pet | CADT | [677] |  |
| *Felis catus* (domestic cat) | Iran | 113 | 1 | 2 groups | Stray | CM | [678] |  |
| *Felis catus* (domestic cat) | USA | 250 | 34 | None | Pet | CADT | [679] |  |
| *Felis catus* (domestic cat) | Australia | 120 | 11 | 2 groups | Ca/Sh | CADT | [680] |  |
| *Felis catus* (domestic cat) | Brazil | 51 | 3 | None | Euthanasia | CM | [681] |  |
| *Felis catus* (domestic cat) | Romania | 23 | 6 | 2 groups | Pet | CM | [465] |  |
| *Felis catus* (domestic cat) | USA | 1,566 | 36 | 5 groups | Pet | CM | [466] |  |
| *Felis catus* (domestic cat) | UK | 55 | 3 | 9–20 wks | Pet | CADT | [682] |  |
| *Felis catus* (domestic cat) | Spain | 50 | 3 | 2 groups | Shelter | CM | [467] |  |
| *Felis catus* (domestic cat) | Netherlands | 22 | 3 | None | Pet | CM/PCR | [470] |  |
| *Felis catus* (domestic cat) | Brazil | 116 | 40 | None | Pet | CM | [683] |  |
| *Felis catus* (domestic cat) | Belgium | 114 | 30 | 4 groups | Pet | CADT | [476] |  |
| *Felis catus* (domestic cat) | Germany | 2,111 | 519 | 4 groups | Pet | CADT | [476] |  |
| *Felis catus* (domestic cat) | Spain | 370 | 54 | 4 groups | Pet | CADT | [476] |  |
| *Felis catus* (domestic cat) | France | 503 | 77 | 4 groups | Pet | CADT | [476] |  |
| *Felis catus* (domestic cat) | Italy | 655 | 116 | 4 groups | Pet | CADT | [476] |  |
| *Felis catus* (domestic cat) | Netherlands | 409 | 54 | 4 groups | Pet | CADT | [476] |  |
| *Felis catus* (domestic cat) | UK | 52 | 6 | 4 groups | Pet | CADT | [476] |  |
| *Felis catus* (domestic cat) | New Zealand | 22 | 7 | None | Cat show | CADT | [684] |  |
| *Felis catus* (domestic cat) | Romania | 414 | 3 | 2 groups | Pet | CM | [685] |  |
| *Felis catus* (domestic cat) | Iran | 150 | 5 | 3 groups | Pet | CADT | [484] |  |
| *Felis catus* (domestic cat) | Canada | 389 | 16 | 4 groups | Pet | CADT | [486] |  |
| *Felis catus* (domestic cat) | Japan | 55 | 1 | < 15 yrs | Pet | CM/PCR | [490] |  |
| *Felis catus* (domestic cat) | Germany | 8,560 | 1,082 | None | Pet | CADT | [494] |  |
| *Felis catus* (domestic cat) | Iran | 37 | 7 | 2 groups | Stray | CM | [492] |  |
| *Felis catus* (domestic cat) | Iran | 52 | 0 | 2 groups | Stray | CM | [686] |  |
| *Felis catus* (domestic cat) | Portugal | 22 | 2 | None | Pe/Sh | CM/PCR | [497] |  |
| *Felis catus* (domestic cat) | USA | 1,322 | 118 | None | Shelter | CM | [687] |  |
| *Felis catus* (domestic cat) | Poland | 160 | 6 | None | Pet | CADT/PCR | [688] |  |
| *Felis catus* (domestic cat) | Canada | 153 | 0 | 3 groups | Pe/Sh | CM | [500] |  |
| *Felis catus* (domestic cat) | Egypt | 113 | 3 | None | Stray | CM | [689] |  |
| *Felis catus* (domestic cat) | Romania | 183 | 51 | 3 groups | Pet | CADT | [690] |  |
| *Felis catus* (domestic cat) | Italy | 181 | 11 | None | Pe/St | PCR | [691] |  |
| *Felis catus* (domestic cat) | Costa Rica | 7 | 4 | None | Pet | CADT/PCR | [503] | ❸ |
| *Felis catus* (domestic cat) | Japan | 321 | 26 | 3 groups | PS | CM/PCR | [692] |  |
| *Felis catus* (domestic cat) | Norway | 52 | 4 | None | Cat show | CADT | [693] |  |
| *Felis catus* (domestic cat) | Germany | 584 | 40 | 2 groups | Stray | CADT | [504] |  |
| *Felis catus* (domestic cat) | Spain | 144 | 6 | None | Shelter | CM/PCR | [505] |  |
| *Felis catus* (domestic cat) | Finland | 402 | 13 | 2 groups | Pet | CADT | [694] |  |
| *Felis catus* (domestic cat) | USA | 74 | 11 | None | Shelter | CADT/PCR | [109] |  |
| *Felis catus* (domestic cat) | USA | 273 | 19 | None | Pet | CADT | [695] |  |
| *Felis catus* (domestic cat) | Brazil | 10 | 2 | None | Pet | CM/PCR | [119] | ❸ |
| *Felis catus* (domestic cat) | Hungary | 115 | 43 | None | Pet | CADT | [696] |  |
| *Felis catus* (domestic cat) | Canada | 283 | 28 | 4 groups | Pet | CADT | [697] |  |
| *Felis catus* (domestic cat) | Australia | 4 | 0 | None | Catchment | PCR | [518] | ❸ |
| *Felis catus* (domestic cat) | Brazil | 191 | 8 | 5 groups | Pet | CM | [698] |  |
| *Felis catus* (domestic cat) | Italy | 81 | 1 | 2 groups | Pet | CADT/PCR | [521] |  |
| *Felis catus* (domestic cat) | Italy | 139 | 4 | 2 groups | Stray | CADT | [699] |  |
| *Felis catus* (domestic cat) | Austria | 92 | 4 | 2 groups | Pet | CM | [700] |  |
| *Felis catus* (domestic cat) | Belgium | 55 | 0 | 2 groups | Pet | CM | [700] |  |
| *Felis catus* (domestic cat) | France | 187 | 8 | 2 groups | Pet | CM | [700] |  |
| *Felis catus* (domestic cat) | Hungary | 300 | 0 | 2 groups | Pet | CM | [700] |  |
| *Felis catus* (domestic cat) | Italy | 515 | 10 | 2 groups | Pet | CM | [700] |  |
| *Felis catus* (domestic cat) | Romania | 300 | 2 | 2 groups | Pet | CM | [700] |  |
| *Felis catus* (domestic cat) | Spain | 70 | 24 | 2 groups | Pet | CM | [700] |  |
| *Felis catus* (domestic cat) | Iran | 140 | 15 | None | Stray | CM | [701] |  |
| *Felis catus* (domestic cat) | Albania | 58 | 17 | None | Pet | CADT | [702] |  |
| *Felis catus* (domestic cat) | UK | 1,088 | 255 | 3 groups | Clinic | PCR | [703] |  |
| *Felis catus* (domestic cat) | USA | 68 | 38 | None | Shelter | PCR | [704] |  |
| *Felis catus* (domestic cat) | Italy | 156 | 35 | 2 groups | Pet | CADT/PCR | [540] |  |
| *Felis catus* (domestic cat) | Iraq | 80 | 2 | None | Pe/St | CM | [705] |  |
| *Felis catus* (domestic cat) | Italy | 146 | 11 | None | Pet | PCR | [706] |  |
| *Felis catus* (domestic cat) | India | 1 | 1 | None | Stray | CADT | [145] | ❺ |
| *Felis catus* (domestic cat) | China | 52 | 1 | 3 groups | Pet shop | PCR | [544] |  |
| *Felis catus* (domestic cat) | Russia | 51 | 0 | 3 groups | Pet | CM | [545] |  |
| *Felis catus* (domestic cat) | Canada | 634 | 9 | 2 groups | Shelter | CM | [550] |  |
| *Felis catus* (domestic cat) | Australia | 345 | 35 | < 12 yrs | Re/Pe/PS/BS | PCR | [707] |  |
| *Felis catus* (domestic cat) | China | 102 | 10 | None | Shelter | CM/PCR | [708] |  |
| *Felis catus* (domestic cat) | Austria | 298 | 37 | None | Pe/Sh/Ca | CADT | [709] |  |
| *Felis catus* (domestic cat) | Australia | 1 | 0 | None | Catchment | PCR | [328] | ❸ |
| *Felis catus* (domestic cat) | Poland | 33 | 5 | None | Pet | CADT/PCR | [554] |  |
| *Felis catus* (domestic cat) | Ecuador | 6 | 0 | None | Pet | PCR | [294] | ❸ |
| *Felis catus* (domestic cat) | China | 160 | 21 | 2 groups | Ur/Sh/PS | PCR | [558] |  |
| *Felis catus* (domestic cat) | Spain | 34 | 2 | None | Pet | PCR | [562] |  |
| *Felis catus* (domestic cat) | Spain | 65 | 6 | 3 groups | Shelter | PCR | [563] |  |
| *Felis catus* (domestic cat) | Greece | 264 | 55 | 6 groups | Pe/Sh | CADT/PCR | [566] |  |
| *Felis catus* (domestic cat) | China | 11 | 2 | None | Pet | PCR | [15] | ❸ |
| *Felis catus* (domestic cat) | Germany | 903 | 5 | None | Pet | CM | [162] |  |
| *Felis catus* (domestic cat) | Poland | 68 | 2 | 2 groups | Shelter | CM | [569] |  |
| *Felis catus* (domestic cat) | Japan | Pos. isolate | 41 | None | Cattery | PCR | [710] | ❹ |
| *Felis catus* (domestic cat) | USA | 846 | 10 | 3 groups | Free roam | CM | [711] |  |
| *Felis catus* (domestic cat) | China | 104 | 6 | 2 groups | Stray | CM/PCR | [577] |  |
| *Felis catus* (domestic cat) | Brazil | 1 | 0 | None | Pet | CM | [178] | ❸ |
| *Felis catus* (domestic cat) | Germany | 145 | 26 | 5 groups | Pe/Sh/St | CADT/PCR | [580] |  |
| *Felis catus* (domestic cat) | Greece | 1,150 | 26 | 2 groups | Pe/St | CM | [712] |  |
| *Felis catus* (domestic cat) | Ireland | 271 | 5 | 3 groups | Stray | CM | [584] |  |
| *Felis catus* (domestic cat) | Poland | 96 | 5 | 2 groups | Pet | CM | [585] |  |
| *Felis catus* (domestic cat) | Brazil | 30 | 6 | None | Captive | CM | [320] |  |
| *Felis catus* (domestic cat) | Iran | 300 | 4 | 2 groups | Ho | CM/PCR | [587] |  |
| *Felis catus* (domestic cat) | Russia | 1,261 | 59 | None | Pet | CM | [590] |  |
| *Felis catus* (domestic cat) | China | 346 | 5 | 2 groups | Pet | PCR | [713] |  |
| *Felis catus* (domestic cat) | China | 418 | 15 | 2 groups | Pe/Cl/Sh/St | PCR | [591] |  |
| *Felis catus* (domestic cat) | Colombia | 203 | 40 | None | Pet | CM | [592] |  |
| *Felis catus* (domestic cat) | USA | 127 | 24 | None | Pet | CADT | [594] |  |
| *Felis catus* (domestic cat) | Thailand | 66 | 18 | 3 groups | Pe/Br | CADT/PCR | [595] |  |
| *Felis catus* (domestic cat) | Denmark | 317 | 36 | 2 groups | U/Sh/Cl | CADT/PCR | [714] |  |
| *Felis catus* (domestic cat) | Colombia | 7 | 0 | None | Stray | CM | [205] | ❸ |
| *Felis catus* (domestic cat) | South Korea | 158 | 6 | 2 groups | Shelter | PCR | [715] |  |
| *Felis catus* (domestic cat) | Turkey | 102 | 51 | None | Pet | CADT | [716] |  |
| *Felis catus* (domestic cat) | Poland | 76 | 3 | None | Pet | PCR | [603] |  |
| *Felis catus* (domestic cat) | South Korea | 1,620 | 126 | 3 groups | Pet | PCR | [717] |  |
| *Felis catus* (domestic cat) | Italy | 133 | 47 | 2 groups | Stray | CADT/PCR | [718] |  |
| *Felis catus* (domestic cat) | Slovakia | 50 | 18 | 2 groups | Ho | CM | [604] |  |
| *Felis catus* (domestic cat) | China | 17 | 1 | None | Pet | PCR | [225] |  |
| *Felis catus* (domestic cat) | UK | 122 | 46 | None | Pet | CADT | [617] | ❹ |
| *Felis catus* (domestic cat) | South Korea | 150 | 46 | 2 groups | Stray | CADT | [719] |  |
| *Felis catus* (domestic cat) | Russia | 1,350 | 71 | 2 groups | Pet | CM | [622] |  |
| *Felis catus* (domestic cat) | Mexico | 200 | 50 | 2 groups | Pe/St | CM | [720] |  |
| *Felis catus* (domestic cat) | Egypt | 134 | 43 | None | Ho | PCR | [624] |  |
| *Felis catus* (domestic cat) | Iran | 165 | 2 | 2 groups | Pe/St | PCR | [721] |  |
| *Felis catus* (domestic cat) | China | 46 | 10 | None | Pet | PCR | [626] |  |
| *Felis catus* (domestic cat) | Spain | 35 | 7 | None | Pet | CADT | [627] |  |
| *Felis catus* (domestic cat) | Ireland | 241 | 31 | 4 groups | Shelter | CADT | [625] |  |
| *Felis catus* (domestic cat) | Brazil | 55 | 2 | None | Pet | CM | [628] |  |
| *Felis catus* (domestic cat) | Turkey | 40 | 1 | None | Pet | CM/PCR | [722] |  |
| *Felis catus* (domestic cat) | South Korea | 290 | 23 | None | Pe/St/Sh | PCR | [723] |  |
| *Felis catus* (domestic cat) | Austria | 130 | 19 | None | Shelter | CADT | [629] |  |
| *Felis catus* (domestic cat) | Iraq | 75 | 9 | None | Pet | CM/PCR | [630] |  |
| *Felis catus* (domestic cat) | Taiwan | 143 | 11 | None | Pet | PCR | [631] |  |
| *Felis catus* (domestic cat) | Turkey | 42 | 10 | None | Stray | CADT/PCR | [632] |  |
| *Felis chaus* (jungle cat) | Poland | 4 | 0 | None | Captive | CM | [2] | ❸ |
| *Felis silvestris* (wildcat) | Australia | 39 | 8 | None | Feral | CADT | [724] |  |
| *Felis silvestris* (wildcat) | Poland | 3 | 1 | None | Captive | CM | [2] | ❸ |
| *Felis silvestris* (wildcat) | Spain | 2 | 0 | None | Wild | PCR | [637] | ❸ |
| *Felis silvestris* (wildcat) | Luxembourg | 10 | 1 | None | Wild | PCR | [725] |  |
| *Leopardus colocola* (pampas cat) | Brazil | 46 | 1 | None | Captive | CM | [320] |  |
| *Leopardus geoffroyi* (Geoffroy's cat) | Brazil | 62 | 1 | None | Captive | CM | [320] |  |
| *Leptailurus serval* (serval) | Poland | 7 | 1 | None | Captive | CM | [2] |  |
| *Leptailurus serval* (serval) | Croatia | 4 | 1 | None | Captive | CADT/PCR | [1] | ❸ |
| *Lynx canadensis* (Canada lynx) | Poland | 2 | 0 | None | Captive | CM | [2] | ❸ |
| *Lynx lynx* (Eurasian lynx) | Poland | 1 | 0 | None | Captive | CM | [2] | ❸ |
| *Lynx lynx* (Eurasian lynx) | Canada | 1 | 0 | None | Wild | CADT | [7] | ❸ |
| *Lynx lynx* (Eurasian lynx) | Croatia | 2 | 1 | None | Captive | CADT/PCR | [1] | ❸ |
| *Lynx lynx* (Eurasian lynx) | China | 5 | 0 | None | Captive | PCR | [6] |  |
| *Lynx pardinus* (Iberian lynx) | Spain | 6 | 0 | None | Wild | PCR | [637] |  |
| *Lynx pardinus* (Iberian lynx) | Portugal | 30 | 8 | None | Wild | PCR | [235] |  |
| *Otocolobus manul* (Pallas's cat) | Poland | 2 | 0 | None | Captive | CM | [2] | ❸ |
| *Panthera leo* (lion) | Japan | 3 | 0 | None | Captive | CADT | [258] | ❸ |
| *Panthera leo* (lion) | Croatia | 2 | 1 | None | Captive | CADT | [1] | ❸ |
| *Panthera leo* (lion) | Poland | 2 | 0 | None | Captive | CM | [2] | ❸ |
| *Panthera leo* (lion) | China | 1 | 0 | None | Captive | PCR | [15] | ❸ |
| *Panthera leo* (lion) | Bangladesh | 6 | 0 | None | Captive | PCR | [3] |  |
| *Panthera leo bleyenberghi* (lion) | China | 7 | 2 | None | Captive | PCR | [6] |  |
| *Panthera onca* (jaguar) | Japan | 2 | 0 | None | Captive | CADT | [258] | ❸ |
| *Panthera onca* (jaguar) | China | 6 | 0 | None | Captive | PCR | [6] |  |
| *Panthera pardus* (leopard) | Poland | 3 | 0 | None | Captive | CM | [2] | ❸ |
| *Panthera pardus* (leopard) | Japan | 2 | 0 | None | Captive | CADT | [258] | ❸ |
| *Panthera pardus* (leopard) | China | 5 | 1 | None | Captive | PCR | [15] |  |
| *Panthera pardus fusca* (Indian leopard) | China | 6 | 1 | None | Captive | PCR | [6] |  |
| *Panthera pardus* *orientalis* (Amur leopard) | Croatia | 2 | 1 | None | Captive | CADT/PCR | [1] | ❸ |
| *Panthera tigris* (tiger) | China | 9 | 0 | None | Captive | PCR | [15] |  |
| *Panthera tigris* (tiger) | Philippines | 8 | 0 | None | Captive | CM | [726] |  |
| *Panthera tigris altaica* (Siberian tiger) | Poland | 3 | 0 | None | Captive | CM | [2] | ❸ |
| *Panthera tigris altaica* (Siberian tiger) | Croatia | 2 | 0 | None | Captive | CADT | [1] | ❸ |
| *Panthera tigris altaica* (Siberian tiger) | Brazil | 107 | 1 | None | Captive | CM | [320] |  |
| *Panthera tigris altaica* (Siberian tiger) | China | 20 | 1 | None | Captive | PCR | [6] |  |
| *Panthera tigris tigris* (Bengal tiger) | Poland | 3 | 0 | None | Captive | CM | [2] | ❸ |
| *Panthera tigris tigris* (Bengal tiger) | Bangladesh | 1 | 0 | None | Captive | PCR | [3] | ❸ |
| *Panthera tigris tigris* (Bengal tiger) | China | 4 | 0 | None | Captive | PCR | [6] | ❸ |
| *Panthera tigris tigris* (Bengal white tiger) | China | 6 | 0 | None | Captive | PCR | [6] |  |
| *Panthera uncia* (snow leopard) | Croatia | 2 | 1 | None | Captive | CADT/PCR | [1] | ❸ |
| *Prionailurus bengalensis* (leopard cat) | Poland | 1 | 0 | None | Captive | CM | [2] | ❸ |
| *Prionailurus viverrinus* (fishing cat) | Bangladesh | 5 | 0 | None | Captive | PCR | [3] |  |
| *Puma concolor* (mountain lion) | Japan | 2 | 0 | None | Captive | CADT | [258] | ❸ |
| *Puma concolor* (mountain lion) | USA | 11 | 2 | None | Wild | CADT/PCR | [109] |  |
| **Family Herpestidae** |  |  |  |  |  |  |  |  |
| *Herpestes ichneumon* (Egyptian mongoose) | Spain | 2 | 0 | None | Wild | PCR | [637] | ❸ |
| *Mungos mungo* (banded mongoose) | Japan | 7 | 0 | None | Captive | CADT | [258] |  |
| *Suricata suricatta* (meerkat) | Brazil | 37 | 8 | None | Captive | CM | [320] |  |
| *Suricata suricatta* (meerkat) | China | 2 | 0 | None | Captive | PCR | [6] | ❸ |
| *Urva auropunctata* (small Indian mongoose) | Poland | 2 | 0 | None | Captive | CM | [2] |  |
| **Family Hyaenidae** |  |  |  |  |  |  |  |  |
| *Crocuta crocuta* (spotted hyena) | Bangladesh | 1 | 0 | None | Captive | PCR | [3] | ❸ |
| *Crocuta crocuta* (spotted hyena) | China | 2 | 0 | None | Captive | PCR | [6] | ❸ |
| *Hyaena hyaena* (striped hyena) | Japan | 2 | 0 | None | Captive | CADT | [258] | ❸ |
| *Hyaena hyaena* (striped hyena) | Bangladesh | 2 | 0 | None | Captive | PCR | [3] | ❸ |
| **Family Viverridae** |  |  |  |  |  |  |  |  |
| *Arctictis binturong* (binturong or bearcat) | Poland | 2 | 0 | None | Captive | CM | [2] | ❸ |
| *Arctictis binturong whitei* (Palawan bearcat) | Philippines | 2 | 0 | None | Captive | CM | [726] | ❸ |
| *Genetta genetta* (common genet) | Spain | 6 | 0 | None | Wild | PCR | [637] |  |
| *Paguma larvata* (masked palm civet) | Poland | 4 | 0 | None | Captive | CM | [2] | ❸ |
| *Paguma larvata* (masked palm civet) | China | 889 | 34 | 3 groups | Farmed | PCR | [727] |  |
| *Paradoxurus hermaphroditus* (Asian palm civet) | Poland | 2 | 0 | None | Captive | CM | [2] | ❸ |
| *Paradoxurus hermaphroditus* (Asian palm civet) | Croatia | 3 | 1 | None | Captive | CADT | [1] | ❸ |
| *Paradoxurus hermaphroditus* (Asian palm civet) | Philippines | 1 | 0 | None | Captive | CM | [726] | ❸ |
| **Order Chiroptera** |  |  |  |  |  |  |  |  |
| **Family Molossidae** |  |  |  |  |  |  |  |  |
| *Molossus molossus* (Pallas's mastiff bat) | Brazil | 25 | 5 | None | Wild | CM | [728] |  |
| **Family Noctilionidae** |  |  |  |  |  |  |  |  |
| *Noctilio albiventris* (lesser bulldog bat) | Brazil | 19 | 1 | None | Wild | CM | [728] |  |
| **Family Phyllostomidae** |  |  |  |  |  |  |  |  |
| *Trachops cirrhosus* (fringe-lipped ed bat) | Costa-Rica | 1 | 1 | None | Wild | CM | [729] | ❸ |
| **Family Pteropodidae** |  |  |  |  |  |  |  |  |
| *Eidolon helvum* (straw-coloured fruit bat) | Nigeria | 109 | 0 | None | Wild | PCR | [730] |  |
| *Pteropus poliocephalus* (fruit bat) | Australia | 1 | 0 | None | Wild | PCR | [93] | ❸ |
| *Rousettus leschenaulti* (fruit bat) | Nepal | 30 | 0 | None | Wild | CM | [731] |  |
| **Family Rhinolophidae** |  |  |  |  |  |  |  |  |
| *Rhinolophus hipposideros* (horseshoe bat) | Croatia | Pooled FS | 0 | None | Captive | CADT | [1] | ❸ |
| *Rhinolophus macrotis* (horseshoe bat) | Nepal | 30 | 1 | None | Wild | CM | [731] |  |
| **Family Vespertilionidae** |  |  |  |  |  |  |  |  |
| *Myotis lavali* (LaVal's myotis) | Brazil | 10 | 0 | None | Wild | CM | [728] |  |
| **Order Cingulata** |  |  |  |  |  |  |  |  |
| **Family Chlamyphoridae** |  |  |  |  |  |  |  |  |
| *Euphractus sexcinctus* (six-banded armadillo) | Brazil | 3 | 0 | None | Wild | CM | [659] | ❸ |
| *Tolypeutes matacus* (South three-banded armadillo) | Serbia | 1 | 0 | None | Captive | PCR | [8] | ❸ |
| **Order Dasyuromorphia** |  |  |  |  |  |  |  |  |
| **Family Dasyuridae** |  |  |  |  |  |  |  |  |
| *Dasyurus geoffroii* (western quoll) | Australia | 2 | 0 | None | Captive | PCR | [732] | ❸ |
| *Dasyurus geoffroii* (western quoll) | Australia | 22 | 0 | None | Wild | CM | [733] |  |
| *Dasyurus* *maculatus* (spotted quoll) | Australia | 32 | 2 | None | Wild | CM | [734] |  |
| *Dasyurus* *maculatus* (spotted quoll) | Australia | 3 | 1 | None | Captive | PCR | [732] | ❸ |
| *Dasyurus hallucatus* (northern quoll) | Australia | 10 | 0 | None | Wild | PCR | [735] |  |
| *Parantechinus apicalis* (dibbler) | Australia | 1 | 0 | None | Wild | CM | [733] | ❸ |
| *Phascogale calura* (red tailed phascogale) | Australia | 1 | 0 | None | Wild | CM | [733] | ❸ |
| *Planigale maculata* (common planigale) | Australia | 5 | 1 | None | Wild | PCR | [733] |  |
| *Pseudantechinus* sp. | Australia | 2 | 0 | None | Wild | CM | [733] | ❸ |
| *Sarcophilus harrisii* (Tasmanian devil) | Australia | 4 | 0 | None | Captive | PCR | [732] | ❸ |
| *Sarcophilus harrisii* (Tasmanian devil) | Australia | 167 | 8 | None | Wild | PCR | [736] |  |
| **Family Myemecobiidae** |  |  |  |  |  |  |  |  |
| *Myrmecobius fasciatus* (numbat) | Australia | 1 | 0 | None | Wild | CM | [733] | ❸ |
| **Order Didelphimorphia** |  |  |  |  |  |  |  |  |
| **Family Didelphidae** |  |  |  |  |  |  |  |  |
| *Didelphis albiventris* (white-eared opossum) | Brazil | 60 | 0 | None | Wild | CM | [659] |  |
| *Didelphis marsupialis* (common opossum) | Brazil | 36 | 0 | None | Wild | CM | [659] |  |
| *Didelphis* sp. | USA | 68 | 10 | None | Wild | CADT | [109] |  |
| *Didelphis* sp. | Brazil | 8 | 0 | None | Wild | PCR | [737] |  |
| *Gracilinanus agilis* (agile gracile opossum) | Brazil | 10 | 1 | None | Wild | PCR | [737] |  |
| *Lutreolina crassicaudata* (big lutrine opossum) | Brazil | 1 | 0 | None | Wild | CM | [659] | ❸ |
| *Marmosa demerarae* (woolly mouse opossum) | Brazil | 9 | 0 | None | Wild | PCR | [737] |  |
| *Marmosa murina* (Linnaeus’s mouse opossum) | Brazil | 26 | 0 | None | Wild | PCR | [737] |  |
| *Marmosops incanus* (gray slender opossum) | Brazil | 7 | 0 | None | Wild | PCR | [737] |  |
| *Monodelphis americana* (northern three-striped oposs.) | Brazil | 8 | 1 | None | Wild | PCR | [737] |  |
| **Order Diprotodontia** |  |  |  |  |  |  |  |  |
| **Family Macropodidae** |  |  |  |  |  |  |  |  |
| *Lagorchestes conspicillatus* (spectacled hare-wallaby) | Australia | 2 | 0 | None | Wild | CM | [733] | ❸ |
| *Lagostrophus* *fasciatus* (banded hare-wallaby) | Australia | 6 | 0 | None | Wild | CM | [733] |  |
| *Macropus agilis* (agile wallaby) | Australia | 6 | 0 | None | Captive | PCR | [732] |  |
| *Macropus eugenii* (tammar wallaby) | Australia | 24 | 3 | None | Captive | PCR | [732] |  |
| *Macropus fuliginosus* (western grey kangaroo) | Australia | 136 | 10 | None | Wild | PCR | [732] |  |
| *Macropus fuliginosus* (western grey kangaroo) | Australia | 72 | 3 | None | Wild | PCR | [738] |  |
| *Macropus giganteus* (eastern grey kangaroo) | Australia | 2 | 0 | None | Captive | PCR | [732] | ❸ |
| *Macropus giganteus* (eastern grey kangaroo) | Australia | 160 | 18 | None | Wild | PCR | [93] |  |
| *Macropus giganteus* (eastern grey kangaroo) | Australia | 1,455 | 1 | None | Wild | PCR | [328] |  |
| *Macropus giganteus* (eastern gray kangaroo) | China | 13 | 0 | None | Captive | PCR | [5] |  |
| *Macropus giganteus* (albino eastern gray kangaroo) | China | 10 | 0 | None | Captive | PCR | [5] |  |
| *Macropus parma* (parma wallaby) | Poland | 1 | 0 | None | Captive | CM | [2] | ❸ |
| *Macropus parma* (parma wallaby) | Australia | 5 | 2 | None | Captive | PCR | [732] |  |
| *Macropus parma* (parma wallaby) | Serbia | 10 | 0 | None | Captive | PCR | [8] |  |
| *Macropus rufogriseus* (Bennett's wallaby) | Australia | 93 | 18 | None | Wild | CM | [734] |  |
| *Macropus rufogriseus* (Bennett's wallaby) | China | 15 | 1 | None | Captive | PCR | [6] |  |
| *Macropus rufogriseus* (Bennett's wallaby) | Australia | 17 | 0 | None | Wild | PCR | [93] |  |
| *Macropus rufus* (red kangaroo) | Australia | 55 | 8 | None | Captive | PCR | [732] |  |
| *Macropus rufus* (red kangaroo) | Serbia | 5 | 0 | None | Captive | PCR | [8] |  |
| *Macropus* sp. | China | 8 | 1 | None | Captive | PCR | [329] |  |
| *Macropus* sp. | China | 8 | 1 | None | Captive | PCR | [6] |  |
| *Petrogale lateralis* (black*-*flanked rock*-*wallaby) | Australia | 2 | 0 | None | Captive | PCR | [732] | ❸ |
| *Petrogale xanthopus* (yellow*-*footed rock*-*wallaby) | Australia | 1 | 1 | None | Captive | PCR | [732] | ❸ |
| *Setonix brachyurus* (quokka) | Australia | 15 | 1 | None | Captive | PCR | [732] |  |
| *Thylogale billardierii* (Tasmanian pademelon) | Australia | 13 | 3 | None | Wild | CM | [734] |  |
| *Wallabia bicolor* (swamp wallaby) | Australia | 20 | 2 | None | Wild | PCR | [732] |  |
| *Wallabia bicolor* (swamp wallaby) | Australia | 388 | 0 | None | Wild | PCR | [328] |  |
| **Family Petauridae** |  |  |  |  |  |  |  |  |
| *Petaurus breviceps* (sugar glider possum) | Australia | 2 | 0 | None | Captive | PCR | [732] | ❸ |
| **Family Phalangeridae** |  |  |  |  |  |  |  |  |
| *Trichosurus cunninghami* (mountain brushtail possum) | Australia | 32 | 7 | None | Wild | PCR | [732] | ❸ |
| *Trichosurus vulpecula* (common brushtail possum) | New Zealand | 124 | 16 | None | Wild | CM | [739] |  |
| *Trichosurus vulpecula* (common brushtail possum) | Australia | 89 | 14 | None | Wild | CM | [734] |  |
| *Trichosurus vulpecula* (common brushtail possum) | New Zealand | 76 | 18 | None | Wild | CM | [652] |  |
| *Trichosurus vulpecula* (common brushtail possum) | Japan | 1 | 0 | None | Captive | CADT | [258] | ❸ |
| *Trichosurus vulpecula* (common brushtail possum) | Australia | 48 | 13 | None | Wild | PCR | [732] |  |
| *Trichosurus vulpecula* (common brushtail possum) | Australia | 129 | 0 | None | Wild | CM | [733] |  |
| *Trichosurus vulpecula* (common brushtail possum) | Croatia | 2 | 1 | None | Captive | CADT | [1] | ❸ |
| *Trichosurus vulpecula* (common brushtail possum) | Australia | 3 | 0 | None | Wild | PCR | [328] | ❸ |
| *Trichosurus vulpecula* (common brushtail possum) | Australia | 58 | 2 | None | Wild | PCR | [735] |  |
| **Family Phascolarctidae** |  |  |  |  |  |  |  |  |
| *Phascolarctos cinereus* (koala bear) | Japan | 7 | 0 | None | Captive | CADT | [258] |  |
| *Phascolarctos cinereus* (koala bear) | Australia | 40 | 5 | None | Wild | PCR | [732] |  |
| **Family Potoroidae** |  |  |  |  |  |  |  |  |
| *Aepyprymnus rufescens* (rufous bettong) | Australia | 2 | 1 | None | Captive | PCR | [732] | ❸ |
| *Bettongia lesueur* (boodie) | Australia | 3 | 0 | None | Wild | CM | [733] | ❸ |
| *Bettongia* *penicillata* (woylie) | Australia | 3 | 0 | None | Captive | PCR | [732] | ❸ |
| *Bettongia* *penicillata* (woylie) | Australia | 83 | 0 | None | Wild | CM | [733] |  |
| *Potorous tridactylus* (long*-*nosed potoroo) | Australia | 7 | 2 | None | Wild | CM | [734] |  |
| *Potorous tridactylus* (long*-*nosed potoroo) | Australia | 3 | 1 | None | Captive | PCR | [732] | ❸ |
| **Family Pseudocheiridae** |  |  |  |  |  |  |  |  |
| *Pseudocheirus occidentalis* (western ringtail possum) | Australia | 1 | 0 | None | Captive | PCR | [732] | ❸ |
| *Pseudocheirus peregrinus* (common ringtail possum) | Australia | 2 | 1 | None | Captive | PCR | [732] | ❸ |
| **Family Vombatidae** |  |  |  |  |  |  |  |  |
| *Lasiorhinus* *latifrons* (southern hairy*-*nosed wombat) | Australia | 5 | 1 | None | Captive | PCR | [732] |  |
| *Lasiorhinus* *krefftii* (northern hairy*-*nosed wombat) | Australia | 1 | 0 | None | Captive | PCR | [732] | ❸ |
| *Vombatus ursinus* (common wombat) | Australia | 35 | 7 | None | Wild | CM | [734] |  |
| *Vombatus ursinus* (common wombat) | Australia | 2 | 0 | None | Captive | PCR | [732] | ❸ |
| *Vombatus ursinus* (common wombat) | Australia | 55 | 0 | None | Wild | PCR | [87] |  |
| *Vombatus ursinus* (common wombat) | Australia | 67 | 0 | None | Wild | PCR | [93] |  |
| *Vombatus ursinus* (common wombat) | Australia | 435 | 1 | None | Wild | PCR | [328] |  |
| **Order Eulipotyphla** |  |  |  |  |  |  |  |  |
| **Family Soricidae** |  |  |  |  |  |  |  |  |
| *Crocidura russula* (greater white-toothed shrew) | Spain | 6 | 0 | None | Wild | PCR | [740] |  |
| *Crocidura russula* (greater white-toothed shrew) | Portugal | 47 | 0 | None | Wild | PCR | [741] |  |
| *Sorex* *coronatus* (Millet's shrew) | Spain | 1 | 0 | None | Wild | PCR | [740] | ❸ |
| *Sorex vagrans* (vagrant shrew) | USA | 1 | 0 | None | Wild | CM | [314] | ❸ |
| **Family Erinaceidae** |  |  |  |  |  |  |  |  |
| *Erinaceus europaeus* (European hedgehog) | Germany | 1,175 | 0 | None | Pet | CM | [391] |  |
| *Erinaceus europaeus* (European hedgehog) | New Zealand | 6 | 2 | None | Wild | CM | [652] |  |
| *Erinaceus europaeus* (European hedgehog) | Germany | 106 | 0 | None | Pet | CM | [52] |  |
| *Erinaceus europaeus* (European hedgehog) | Germany | 2 | 0 | None | Pet | CADT | [653] | ❸ |
| *Erinaceus europaeus* (European hedgehog) | Netherland | 90 | 10 | None | Wild | PCR | [742] |  |
| *Erinaceus europaeus* (European hedgehog) | Germany | 205 | 0 | None | Pet | CM | [162] |  |
| *Erinaceus europaeus* (European hedgehog) | Russia | 21 | 0 | None | Pet | CM | [590] |  |
| *Erinaceus europaeus* (European hedgehog) | Denmark | 74 | 0 | None | Wild | CADT | [743] |  |
| **Order Hyracoidea** |  |  |  |  |  |  |  |  |
| **Family Procaviidae** |  |  |  |  |  |  |  |  |
| *Procavia capensis* (rock hyrax) | Croatia | 2 | 2 | None | Captive | CADT/PCR | [1] | ❸ |
| **Order Lagomorpha** |  |  |  |  |  |  |  |  |
| **Family Leporidae** |  |  |  |  |  |  |  |  |
| *Lepus americanus* (snowshoe hare) | USA | 2 | 0 | None | Wild | CM | [306] | ❸ |
| *Lepus arcticus* (arctic hare) | Canada | 453 | 0 | None | Wild | CADT | [7] |  |
| *Lepus europaeus* (European hare) | Croatia | 73 | 0 | None | Captive | CADT | [311] |  |
| *Lepus europaeus* (European hare) | Poland | 4 | 0 | None | Wild | PCR | [310] | ❸ |
| *Lepus granatensis* (Iberian hare) | Spain | 111 | 25 | 3 groups | Wild | PCR | [744] |  |
| *Lepus tolai* (tolai hare) | China | 221 | 51 | None | Pet | PCR | [745] |  |
| *Oryctolagus cuniculus* (rabbit) | USA | 15 | 0 | None | Wild | CM | [314] |  |
| *Oryctolagus cuniculus* (rabbit) | New Zealand | 5 | 1 | None | Wild | CM | [652] |  |
| *Oryctolagus cuniculus* (rabbit) | Germany | 232 | 0 | None | Pet | CM | [52] |  |
| *Oryctolagus cuniculus* (rabbit) | Japan | 4 | 0 | None | Captive | CADT | [258] | ❸ |
| *Oryctolagus cuniculus* (rabbit) | Germany | 84 | 5 | None | Pet | CADT | [653] |  |
| *Oryctolagus cuniculus* (rabbit) | China | 378 | 28 | None | Farmed | CM/PCR | [746] |  |
| *Oryctolagus cuniculus* (rabbit) | Ausralia | 263 | 3 | None | Wild | PCR | [518] |  |
| *Oryctolagus cuniculus* (rabbit) | USA | 4 | 0 | None | Fair | CADT | [125] | ❸ |
| *Oryctolagus cuniculus* (rabbit) | Germany | 528 | 40 | None | Pet | CADT/PCR | [747] |  |
| *Oryctolagus cuniculus* (rabbit) | China | 955 | 80 | 4 groups | Farmed | CM/PCR | [748] |  |
| *Oryctolagus cuniculus* (rabbit) | Ausralia | 97 | 1 | None | Wild | PCR | [328] |  |
| *Oryctolagus cuniculus* (rabbit) | Iran | 58 | 5 | 2 groups | Pet | CADT | [749] |  |
| *Oryctolagus cuniculus* (rabbit) | Ecuador | 20 | 4 | None | Pet | PCR | [294] |  |
| *Oryctolagus cuniculus* (rabbit) | Germany | 434 | 0 | None | Pet | CM | [162] |  |
| *Oryctolagus cuniculus* (rabbit) | China | 426 | 42 | None | Farmed | CM/PCR | [750] |  |
| *Oryctolagus cuniculus* (rabbit) | China | 321 | 6 | None | Farmed | PCR | [751] |  |
| *Oryctolagus cuniculus* (chinchilla rabbit) | Nigeria | 83 | 60 | < 6 m | Farmed | PCR | [752] |  |
| *Oryctolagus cuniculus* (rabbit) | Russia | 165 | 3 | None | Pet | CM | [590] |  |
| *Oryctolagus cuniculus* (long-haired rabbit) | China | 188 | 3 | None | Pet | PCR | [745] |  |
| *Oryctolagus cuniculus* (New Zealand white rabbit) | China | 207 | 15 | None | Pet | PCR | [745] |  |
| *Oryctolagus cuniculus* (rabbit) | Ecuador | 24 | 2 | None | Captive | CM | [351] |  |
| *Oryctolagus cuniculus* (rabbit) | China | 537 | 19 | 4 groups | Farmed | PCR | [753] |  |
| *Oryctolagus cuniculus* (rabbit) | China | 212 | 67 | None | Farmed | PCR | [225] |  |
| *Oryctolagus cuniculus* (rabbit) | Spain | 439 | 128 | 3 groups | Wild | PCR | [744] |  |
| *Oryctolagus cuniculus* (rabbit) | Brazil | 100 | 40 | None | Pet | CM/PCR | [754] |  |
| *Oryctolagus* sp. | UAE | 2 | 2 | None | Captive | PCR | [218] | ❸ |
| *Oryctolagus* sp. | Australia | 2 | 0 | None | Farmed | PCR | [93] | ❸ |
| **Family Ochotonidae** |  |  |  |  |  |  |  |  |
| *Ochotona daurica* (pika) | China | 11 | 3 | None | Wild | CADT | [11] |  |
| **Order Peramelemorphia** |  |  |  |  |  |  |  |  |
| **Family Peramelidae** |  |  |  |  |  |  |  |  |
| Isoodon macrourus (northern brown bandicoot) | Australia | 62 | 1 | None | Wild | PCR | [735] |  |
| *Isoodon obesulus* (southern brown bandicoot) | Australia | 26 | 16 | None | Wild | CM | [734] |  |
| *Isoodon obesulus* (southern brown bandicoot) | Australia | 72 | 1 | None | Wild | CM | [755] |  |
| *Isoodon obesulus* (southern brown bandicoot) | Australia | 3 | 0 | None | Captive | PCR | [732] |  |
| *Isoodon obesulus* (southern brown bandicoot) | Australia | 55 | 13 | None | Wild | CM/PCR | [733] |  |
| *Isoodon obesulus* (southern brown bandicoot) | Australia | Pos. isolate | 75 | None | Wild | PCR | [756] | ❹ |
| *Perameles bougainville* (western barred bandicoot) | Australia | 10 | 0 | None | Wild | CM | [733] |  |
| *Perameles gunnii* (eastern barred bandicoot) | Australia | 2 | 0 | None | Captive | PCR | [732] |  |
| **Family Thylacomyidae** |  |  |  |  |  |  |  |  |
| *Macrotis lagotis* (bilby) | Australia | 3 | 0 | None | Wild | CM | [733] | ❸ |
| **Order Perissodactyla** |  |  |  |  |  |  |  |  |
| **Family Rhinocerotidae** |  |  |  |  |  |  |  |  |
| *Rhinoceros unicornis* (Indian rhinoceros) | China | 1 | 0 | None | Captive | PCR | [15] | ❸ |
| *Rhinoceros unicornis* (Indian rhinoceros) | Bangladesh | 1 | 0 | None | Captive | PCR | [3] | ❸ |
| *Dicerorhinus sumatrensis* (Sumatran rhinoceros) | China | 1 | 0 | None | Captive | PCR | [5] | ❸ |
| **Family Equidae** |  |  |  |  |  |  |  |  |
| *Equus asinus* (domestic donkey) | Poland | 3 | 0 | None | Captive | CM | [2] | ❸ |
| *Equus asinus* (domestic donkey) | Germany | 46 | 0 | None | Not stated | CM | [391] |  |
| *Equus asinus* (domestic donkey) | China | 181 | 28 | 2 groups | Not stated | PCR | [757] |  |
| *Equus asinus* (domestic donkey) | Jordan | 74 | 11 | None | Not stated | CADT/PCR | [758] |  |
| *Equus asinus* (domestic donkey) | China | 540 | 62 | 3 groups | Farmed | PCR | [759] |  |
| *Equus asinus* (domestic donkey) | Bangladesh | 8 | 0 | None | Captive | PCR | [3] |  |
| *Equus asinus* (domestic donkey) | Iran | 5 | 0 | None | Pastoral | CM | [229] |  |
| *Equus asinus* (domestic donkey) | China | 758 | 129 | 2 groups | Farmed | PCR | [760] |  |
| *Equus asinus × Equus caballus* (mule) | USA | 66 | 4 | None | Not stated | CADT | [761] |  |
| *Equus ferus caballus* (domestic horse) | Germany | 9,192 | 0 | None | Not stated | CM | [391] |  |
| *Equus ferus caballus* (domestic horse) | USA | 222 | 29 | 4 groups | Farmed | CADT | [762] |  |
| *Equus ferus caballus* (domestic horse) | Czech | 360 | 18 | 5 m to 14 yrs | Racing | CM | [763] |  |
| *Equus ferus caballus* (domestic horse) | Germany | 37 | 2 | None | Farmed | CM | [764] |  |
| *Equus ferus caballus* (domestic horse) | USA | 91 | 0 | 4–24 yrs | Not stated | CADT | [765] |  |
| *Equus ferus caballus* (domestic horse) | Canada | 34 | 7 | 2 groups | Farmed | CADT | [32] |  |
| *Equus ferus caballus* (domestic horse) | USA | 300 | 2 | None | Trial | CADT | [766] |  |
| *Equus ferus caballus* (domestic horse) | USA | 56 | 0 | None | Pastoral | CM | [767] |  |
| *Equus ferus caballus* (domestic horse) | Greece | 110 | 0 | 5–25 yrs | Farmed | CM | [34] |  |
| *Equus ferus caballus* (domestic horse) | USA | 223 | 9 | None | Not stated | CADT | [761] |  |
| *Equus ferus caballus* (feral horse) | Canada | 1 | 0 | None | Wild | CADT | [7] | ❸ |
| *Equus ferus caballus* (domestic horse) | Germany | 4,399 | 0 | None | Not stated | CM | [52] |  |
| *Equus ferus caballus* (polish konik) | Poland | 5 | 0 | None | Wild | CADT | [10] | ❸ |
| *Equus ferus caballus* (domestic horse) | Poland | 5 | 0 | None | Farmed | CADT | [10] | ❸ |
| *Equus ferus caballus* (domestic horse) | Taiwan | 1 | 0 | None | Farmed | CADT | [73] | ❸ |
| *Equus ferus caballus* (domestic horse) | Brazil | 64 | 0 | None | Club | CM | [768] |  |
| *Equus ferus caballus* (domestic horse) | USA | 223 | 3 | < 10 m | Working | CM | [769] |  |
| *Equus ferus caballus* (domestic horse) | USA | 396 | 2 | < 25 yrs | Farmed | CM | [770] |  |
| *Equus ferus caballus* (domestic horse) | Italy | 150 | 20 | 5 groups | Farmed | CADT/PCR | [771] |  |
| *Equus ferus caballus* (domestic horse) | Australia | 11 | 1 | None | Farmed | PCR | [93] |  |
| *Equus ferus caballus* (domestic horse) | Iraq | 107 | 21 | 4 groups | Stable | CM | [772] |  |
| *Equus ferus caballus* (domestic horse) | Italy | 431 | 37 | None | Farmed | PCR | [773] |  |
| *Equus ferus caballus* (domestic horse) | Iran | 100 | 40 | < 27 yrs | Racing | CM | [774] |  |
| *Equus ferus caballus* (domestic horse) | USA | 2 | 0 | None | Fair | CADT | [125] | ❸ |
| *Equus ferus caballus* (domestic horse) | Colombia | 195 | 34 | 2 groups | Pastoral | PCR | [775] |  |
| *Equus ferus caballus* (domestic horse) | China | 15 | 2 | None | Pastoral | CADT | [11] |  |
| *Equus ferus caballus* (domestic horse) | China | 262 | 4 | None | Pastoral | PCR | [776] |  |
| *Equus ferus caballus* (domestic horse) | Poland | 10 | 1 | None | Farmed | CADT/PCR | [146] |  |
| *Equus ferus caballus* (domestic horse) | Belgium | 134 | 19 | 2–37 d | Farmed | CADT | [777] |  |
| *Equus ferus caballus* (domestic horse) | Germany | 30 | 3 | 17–62 d | Farmed | CADT | [777] |  |
| *Equus ferus caballus* (domestic horse) | Greece | 190 | 22 | 5–180 d | Farmed | CADT | [777] |  |
| *Equus ferus caballus* (domestic horse) | Netherlands | 44 | 5 | 1–28 d | Farmed | CADT | [777] |  |
| *Equus ferus caballus* (domestic horse) | Iran | 42 | 15 | None | Farmed | CM/PCR | [778] |  |
| *Equus ferus caballus* (domestic horse) | Ecuador | 1 | 0 | None | Farmed | PCR | [294] | ❸ |
| *Equus ferus caballus* (domestic horse) | Jordan | 326 | 19 | None | Stable | CADT/PCR | [758] |  |
| *Equus ferus caballus* (domestic horse) | China | 264 | 22 | None | Racing | PCR | [779] |  |
| *Equus ferus caballus* (domestic horse) | Germany | 3,475 | 0 | None | Pet | CM | [162] |  |
| *Equus ferus caballus* (domestic horse) | Brazil | 37 | 0 | None | Farmed | CM | [178] |  |
| *Equus ferus caballus* (domestic horse) | Turkey | 150 | 25 | 3 groups | Racing | PCR | [780] |  |
| *Equus ferus caballus* (domestic horse) | China | 32 | 3 | None | Farmed | PCR | [19] |  |
| *Equus ferus caballus* (domestic horse) | China | 323 | 9 | 3 groups | Pastoral | PCR | [759] |  |
| *Equus ferus caballus* (domestic horse) | China | 621 | 48 | 2 groups | Racing | PCR | [781] |  |
| *Equus ferus caballus* (domestic horse) | Colombia | 6 | 0 | None | Working | CM | [205] |  |
| *Equus ferus caballus* (domestic horse) | Bangladesh | 5 | 0 | None | Captive | PCR | [3] | ❸ |
| *Equus ferus caballus* (domestic horse) | China | 3 | 0 | None | Captive | PCR | [5] | ❸ |
| *Equus ferus caballus* (domestic horse) | China | 3 | 2 | None | Captive | PCR | [6] | ❸ |
| *Equus ferus caballus* (domestic horse) | Portugal | 26 | 2 | None | Farmed | PCR | [235] |  |
| *Equus ferus caballus* (pony) | China | 6 | 0 | None | Captive | PCR | [5] |  |
| *Equus ferus caballus* (pony) | China | 5 | 0 | None | Wild | PCR | [317] |  |
| *Equus ferus caballus* (domestic horse) | Brazil | 208 | 0 | None | Farmed | CM | [782] |  |
| *Equus ferus caballus* (domestic horse) | Iran | 1 | 0 | None | Working | CM | [229] | ❸ |
| *Equus ferus przewalski* (Przewalski’s horse) | Croatia | 3 | 0 | None | Captive | CADT | [1] | ❸ |
| *Equus hemonius* (Asian wild ass) | China | 2 | 0 | None | Captive | PCR | [6] | ❸ |
| *Equus hemonius kulan* (Asian wild ass) | Croatia | 2 | 0 | None | Captive | CADT | [1] | ❸ |
| *Equus quagga* (plains zebra) | Bangladesh | 3 | 0 | None | Captive | PCR | [3] | ❸ |
| *Equus zebra* (mountain zebra) | China | 4 | 0 | None | Captive | PCR | [15] | ❸ |
| *Equus zebra* (mountain zebra) | China | 10 | 0 | None | Captive | PCR | [5] |  |
| *Equus zebra* (mountain zebra) | China | 24 | 4 | None | Captive | PCR | [6] |  |
| *Equus zebra* (mountain zebra) | Serbia | 4 | 0 | None | Captive | PCR | [8] |  |
| **Family Tapiridae** |  |  |  |  |  |  |  |  |
| *Tapirus terrestris* (Brazilian tapir) | Poland | 2 | 0 | None | Captive | CM | [2] | ❸ |
| **Order Pholidota** |  |  |  |  |  |  |  |  |
| **Family Manidae** |  |  |  |  |  |  |  |  |
| *Manis palaeojavanica* (Malayan pangolin) | Bangladesh | 2 | 1 | None | Captive | PCR | [3] | ❸ |
| **Order Pilosa** |  |  |  |  |  |  |  |  |
| **Family Bradypodidae** |  |  |  |  |  |  |  |  |
| *Bradypus tridactylus* (pale-throated sloth) | Brazil | 12 | 2 | None | Wild | CM/PCR | [783] |  |
| **Family Choloepodidae** |  |  |  |  |  |  |  |  |
| *Choloepus didactylus* (Linnaeus's two-toed sloth) | Croatia | 1 | 0 | None | Captive | CADT | [1] | ❸ |
| *Choloepus didactylus* (Linnaeus's two-toed sloth) | Brazil | 15 | 0 | None | Wild | PCR | [783] |  |
| **Family Myrmecophagidae** |  |  |  |  |  |  |  |  |
| *Myrmecophaga tridactyla* (giant anteater) | Brazil | 255 | 1 | None | Captive | CM | [320] |  |
| **Order Primates** |  |  |  |  |  |  |  |  |
| **New world monkey species** |  |  |  |  |  |  |  |  |
| **Family Atelidae** |  |  |  |  |  |  |  |  |
| *Alouatta belzebul* (red-handed howler monkey) | Brazil | 1 | 0 | None | Captive | CADT | [638] | ❸ |
| *Alouatta caraya* (black howler monkey) | Argentina | 90 | 49 | None | Wild | CADT | [784] |  |
| *Alouatta caraya* (black howler monkey) | Brazil | 4 | 1 | None | Captive | CM/PCR | [785] | ❸ |
| *Alouatta caraya* (black howler monkey) | Brazil | 1 | 0 | None | Captive | CADT | [638] | ❸ |
| *Alouatta caraya* (black howler monkey) | Brazil | 37 | 1 | None | Captive | CM | [320] |  |
| *Alouatta caraya* (black howler monkey) | Argentina | 52 | 47 | None | Wild | PCR | [212] |  |
| *Alouatta fusca* (brown howler monkey) | Brazil | 3 | 1 | None | Captive | CM/PCR | [785] | ❸ |
| *Alouatta guariba* (brown howler monkey) | Brazil | 28 | 16 | None | Captive | PCR | [786] |  |
| *Alouatta guariba* (brown howler monkey) | Brazil | 1 | 0 | None | Captive | CADT | [638] | ❸ |
| *Alouatta guariba clamitans* (southern brown howler) | Brazil | 122 | 15 | None | Captive | CM | [320] |  |
| *Alouatta pigra* (Guatemalan black howler) | Mexico | 66 | 21 | None | Wild | CADT/PCR | [787] |  |
| *Alouatta seniculus* (Venezuelan red howler monkey) | Brazil | 4 | 2 | None | Captive | CM | [785] | ❸ |
| *Alouatta seniculus* (Venezuelan red howler monkey) | Brazil | 1 | 1 | None | Captive | CADT | [638] | ❸ |
| *Alouatta seniculus* (Venezuelan red howler monkey) | China | 1 | 0 | None | Captive | PCR | [5] | ❸ |
| *Alouatta* sp. (captive hybrid howler monkeys) | Spain | 4 | 1 | None | Captive | PCR | [788] | ❸ |
| *Ateles belzebuth* (white-fronted spider monkey) | Brazil | 2 | 1 | None | Captive | CM/PCR | [785] | ❸ |
| *Ateles chamek* (Peruvian spider monkey) | Brazil | 2 | 0 | None | Captive | CADT | [638] | ❸ |
| *Ateles fusciceps* (black-headed spider monkey) | Belgium | 5 | 2 | None | Captive | CM | [789] |  |
| *Ateles fusciceps* (black-headed spider monkey) | Brazil | 2 | 0 | None | Captive | CM | [785] | ❸ |
| *Ateles marginatus* (white-cheeked spider monkey) | Brazil | 1 | 0 | None | Captive | CADT | [638] | ❸ |
| *Ateles* *paniscus* (red-faced spider monkey) | Brazil | 5 | 0 | None | Captive | CM | [785] |  |
| *Ateles* *paniscus* (red-faced spider monkey) | China | 2 | 0 | None | Captive | PCR | [790] | ❸ |
| *Ateles* *paniscus* (red-faced spider monkey) | China | 5 | 0 | None | Captive | PCR | [791] |  |
| *Ateles* *paniscus* (red-faced spider monkey) | China | 13 | 0 | None | Captive | CM | [792] |  |
| *Ateles* *paniscus* (red-faced spider monkey) | Brazil | 1 | 0 | None | Captive | CADT | [638] | ❸ |
| *Ateles* sp. | Brazil | 68 | 4 | None | Captive | CM | [320] |  |
| *Ateles* sp. | Spain | 10 | 2 | None | Captive | PCR | [788] |  |
| *Brachyteles arachnoides* (southern muriqui) | Brazil | 29 | 1 | None | Captive | CM | [320] |  |
| *Lagothrix lagotricha* (brown woolly monkey) | Brazil | 5 | 0 | None | Captive | CM | [785] |  |
| *Lagothrix lagotricha* (brown woolly monkey) | Brazil | 59 | 13 | None | Captive | CM | [320] |  |
| *Lagothrix* sp. | Spain | 2 | 0 | None | Captive | PCR | [788] | ❸ |
| **Family Aotidae** |  |  |  |  |  |  |  |  |
| *Aotus azarae* (azara's night monkey) | Argentina | 53 | 4 | None | Wild | CM | [793] |  |
| *Aotus nigriceps* (back-headed night monkey) | Brazil | 2 | 0 | None | Captive | CADT | [638] | ❸ |
| *Aotus trivirgatus* (three-striped night monkey) | China | 7 | 0 | None | Captive | CM | [792] |  |
| *Aotus* sp. | China | 4 | 0 | None | Captive | PCR | [791] | ❸ |
| *Aotus* sp. | Spain | 6 | 0 | None | Captive | PCR | [788] |  |
| **Family Callitrichidae** |  |  |  |  |  |  |  |  |
| *Callithrix aurita* (buffy-tufted marmoset) | Brazil | 1 | 0 | None | Captive | CADT | [638] | ❸ |
| *Callithrix geoffroyi* (Geoffroy's tufted-ear marmoset) | Brazil | 1 | 0 | None | Captive | CM | [785] | ❸ |
| *Callithrix jacchus* (common marmoset) | Belgium | 5 | 0 | None | Captive | CM | [789] |  |
| *Callithrix jacchus* (common marmoset) | China | 8 | 0 | None | Captive | CM | [792] |  |
| *Callithrix jacchus* (common marmoset) | Brazil | 1 | 0 | None | Captive | CADT | [638] | ❸ |
| *Callithrix jacchus* (common marmoset) | Russia | 50 | 2 | None | Pet | CM | [590] |  |
| *Callithrix penicillata* (black-tufted marmoset) | Brazil | 32 | 1 | None | Captive | CM | [320] |  |
| *Callithrix pygmaea* (pygmy marmoset) | Belgium | 10 | 1 | None | Captive | CM | [789] |  |
| *Callithrix pygmaea* (pygmy marmoset) | Croatia | 1 | 0 | None | Captive | CADT | [1] | ❸ |
| *Callithrix* sp. (captive hybrid marmosets) | China | 5 | 0 | None | Captive | PCR | [791] |  |
| *Callithrix* sp. (captive hybrid marmosets) | Spain | 11 | 4 | None | Captive | PCR | [788] |  |
| *Callimico goeldii* (Goeldi's marmoset) | Belgium | 2 | 0 | None | Captive | CM | [789] | ❸ |
| *Callimico* sp. | Spain | 6 | 2 | None | Captive | PCR | [788] |  |
| *Cebuella pygmaea* (western pygmy marmoset) | Spain | 3 | 1 | None | Captive | PCR | [794] | ❸ |
| *Cebuella* sp. | Spain | 7 | 2 | None | Captive | PCR | [788] |  |
| *Leontopithecus* sp. | Spain | 14 | 1 | None | Captive | PCR | [788] |  |
| *Leontopithecus chrysomelas* (golden headed lion tamarin) | Brazil | 7 | 0 | None | Captive | CM | [785] |  |
| *Leontopithecus chrysomelas* (golden headed lion tamarin) | China | 7 | 0 | None | Captive | CM | [792] |  |
| *Leontopithecus chrysomelas* (golden headed lion tamarin) | Brazil | 1 | 0 | None | Captive | CADT | [638] | ❸ |
| *Leontopithecus chrysopygus* (black lion tamarin) | Brazil | 237 | 6 | None | Captive | CM | [320] |  |
| *Leontopithecus rosalia* (golden lion tamarin) | Brazil | 2 | 0 | None | Captive | CM | [785] | ❸ |
| *Leontopithecus rosalia* (golden lion tamarin) | Brazil | 37 | 5 | None | Captive | CM | [320] |  |
| *Mico argentatus* (Silvery marmoset) | Poland | 4 | 4 | None | Captive | CM | [2] | ❸ |
| *Mico argentatus* (Silvery marmoset) | Brazil | 2 | 0 | None | Captive | CM | [785] | ❸ |
| *Mico argentatus* (Silvery marmoset) | Serbia | 1 | 0 | None | Captive | PCR | [8] | ❸ |
| *Mico* sp. | Spain | 6 | 1 | None | Captive | PCR | [788] |  |
| *Saguinus bicolor* (pied tamarin) | Brazil | 1 | 0 | None | Captive | CM | [785] | ❸ |
| *Saguinus fuscicollis* (Spix's saddle-back tamarin) | Brazil | 1 | 0 | None | Captive | CM | [785] | ❸ |
| *Saguinus geoffroyi* (Geoffroy's tamarin) | Poland | 3 | 0 | None | Captive | CM | [2] | ❸ |
| *Saguinus imperator* (Emperor tamarin) | Belgium | 2 | 0 | None | Captive | CM | [789] | ❸ |
| *Saguinus melanoleucus* (White-mantled tamarin) | Brazil | 1 | 0 | None | Captive | CM | [785] | ❸ |
| *Saguinus midas* (red-handed tamarin) | China | 9 | 0 | None | Captive | CM | [792] |  |
| *Saguinus mystax* (Moustached tamarin) | Brazil | 1 | 0 | None | Captive | CADT | [638] | ❸ |
| *Saguinus oedipus* (Cottontop tamarin) | China | 7 | 0 | None | Captive | CM | [792] |  |
| *Saguinus oedipus* (Cottontop tamarin) | Russia | 65 | 3 | None | Pet | CM | [590] |  |
| *Saguinus* sp. | Spain | 19 | 3 | None | Captive | PCR | [788] |  |
| **Family Cebidae** |  |  |  |  |  |  |  |  |
| *Cebus albifrons* (Humboldt's white-fronted capuchin) | China | 5 | 0 | None | Captive | PCR | [791] |  |
| *Cebus albifrons* (Humboldt's white-fronted capuchin) | China | 26 | 0 | None | Captive | CM | [792] |  |
| *Cebus albifrons* (Humboldt's white-fronted capuchin) | Brazil | 2 | 0 | None | Captive | CADT | [638] | ❸ |
| *Cebus capucinus* (Colombian white-faced capuchin) | China | 5 | 0 | None | Captive | CM | [792] |  |
| *Cebus capucinus* (Colombian white-faced capuchin) | China | 1 | 0 | None | Captive | PCR | [5] | ❸ |
| *Cebus capucinus* (Colombian white-faced capuchin) | China | 4 | 0 | None | Captive | PCR | [6] | ❸ |
| *Cebus kaapori* (kaapori capuchin) | China | 11 | 0 | None | Captive | CM | [792] |  |
| *Cebus kaapori* (kaapori capuchin) | Brazil | 22 | 1 | None | Captive | CM | [320] |  |
| *Cebus olivaceus* (Guianan weeper capuchin) | China | 1 | 0 | None | Captive | CM | [792] | ❸ |
| *Cebus olivaceus* (Guianan weeper capuchin) | China | 4 | 0 | None | Captive | PCR | [791] |  |
| *Cebus olivaceus* (Guianan weeper capuchin) | China | 1 | 0 | None | Captive | PCR | [6] | ❸ |
| *Cebus* sp. | Spain | 27 | 3 | None | Captive | PCR | [788] |  |
| *Sapajus apella* (tufted capuchin) | Japan | 2 | 0 | None | Captive | CADT | [258] | ❸ |
| *Sapajus apella* (tufted capuchin) | Belgium | 5 | 1 | None | Captive | CM | [789] |  |
| *Sapajus apella* (tufted capuchin) | Croatia | 5 | 0 | None | Captive | CADT | [1] |  |
| *Sapajus apella* (tufted capuchin) | China | 48 | 0 | None | Captive | PCR | [790] |  |
| *Sapajus apella* (tufted capuchin) | China | 2 | 0 | None | Captive | PCR | [791] | ❸ |
| *Sapajus apella* (tufted capuchin) | China | 50 | 0 | None | Captive | CM | [792] |  |
| *Sapajus apella* (tufted capuchin) | Brazil | 2 | 0 | None | Captive | CADT | [638] | ❸ |
| *Sapajus apella* (tufted capuchin) | China | 8 | 0 | None | Captive | PCR | [5] |  |
| *Sapajus apella* (tufted capuchin) | China | 6 | 0 | None | Captive | PCR | [6] |  |
| *Sapajus flavius* (blond capuchin) | Brazil | 2 | 0 | None | Captive | CADT | [638] | ❸ |
| *Sapajus robustus* (crested capuchin) | Brazil | 1 | 0 | None | Captive | CADT | [638] | ❸ |
| *Sapajus xanthosternos* (golden-bellied capuchin) | Brazil | 3 | 0 | None | Captive | CADT | [638] | ❸ |
| *Sapajus* sp. | Brazil | 3 | 0 | None | Captive | CADT | [638] | ❸ |
| *Sapajus* sp. | Spain | 5 | 0 | None | Captive | PCR | [788] |  |
| *Saimiri* *sciureus* (Guianan squirrel monkey) | Poland | 3 | 0 | None | Captive | CM | [2] | ❸ |
| *Saimiri* *sciureus* (Guianan squirrel monkey) | Belgium | 26 | 2 | None | Captive | CM | [789] |  |
| *Saimiri* *sciureus* (Guianan squirrel monkey) | China | 20 | 1 | None | Captive | PCR | [795] |  |
| *Saimiri* *sciureus* (Guianan squirrel monkey) | China | 103 | 0 | None | Captive | CM | [792] |  |
| *Saimiri* *sciureus* (Guianan squirrel monkey) | Spain | 5 | 0 | None | Captive | PCR | [794] |  |
| *Saimiri* *sciureus* (Guianan squirrel monkey) | China | 11 | 0 | None | Captive | PCR | [5] |  |
| *Saimiri* *sciureus* (Guianan squirrel monkey) | China | 11 | 0 | None | Captive | PCR | [6] |  |
| *Saimiri* sp. | China | 41 | 0 | None | Captive | PCR | [790] |  |
| *Saimiri* sp. | China | 43 | 10 | None | Captive | PCR | [791] |  |
| *Saimiri* sp. | Spain | 14 | 1 | None | Captive | PCR | [788] |  |
| **Family Pitheciidae** |  |  |  |  |  |  |  |  |
| *Callicebus* *nigrifrons* (black-fronted titi) | Brazil | 2 | 0 | None | Captive | CM | [785] | ❸ |
| *Callicebus* sp. | Spain | 3 | 0 | None | Captive | PCR | [788] | ❸ |
| *Plecturocebus* sp. | Spain | 3 | 0 | None | Captive | PCR | [788] | ❸ |
| *Chiropotes satanas* (black bearded saki) | China | 2 | 0 | None | Captive | PCR | [791] | ❸ |
| *Chiropotes satanas* (black bearded saki) | China | 1 | 0 | None | Captive | CM | [792] | ❸ |
| *Pithecia* sp. | Spain | 10 | 1 | None | Captive | PCR | [788] |  |
| **Old world monkey species** |  |  |  |  |  |  |  |  |
| **Family Cercopithecidae** |  |  |  |  |  |  |  |  |
| *Allochrocebus lhoesti* (L'Hoest's monkey) | China | 1 | 0 | None | Captive | CM | [792] | ❸ |
| *Cercopithecus ascanius* (red-tailed monkey) | Uganda | 234 | 10 | None | Captive | CM | [796] |  |
| *Cercopithecus ascanius* (red-tailed monkey) | Uganda | 20 | 1 | None | Wild | CADT | [797] |  |
| *Cercopithecus ascanius* (red-tailed monkey) | Uganda | 21 | 1 | None | Captive | PCR | [92] |  |
| *Cercopithecus campbelli* (Campbell's mona monkey) | Côte d’Ivoire | 178 | 28 | None | Wild | CM | [798] |  |
| *Cercopithecus cephus* (moustached monkey) | Poland | 2 | 0 | None | Captive | CM | [2] | ❸ |
| *Cercopithecus diana* (diana monkey) | Croatia | 4 | 0 | None | Captive | CADT | [1] | ❸ |
| *Cercopithecus diana* (diana monkey) | China | 1 | 0 | None | Captive | PCR | [791] | ❸ |
| *Cercopithecus diana* (diana monkey) | Côte d’Ivoire | 171 | 25 | None | Wild | CM | [798] |  |
| *Cercopithecus diana* (diana monkey) | China | 6 | 0 | None | Captive | CM | [792] |  |
| *Cercopithecus diana* (diana monkey) | China | 3 | 0 | None | Captive | PCR | [6] | ❸ |
| *Cercopithecus* *hamlyni* (Hamlyn's monkey) | Belgium | 8 | 0 | None | Captive | CM | [789] |  |
| *Cercopithecus* *hamlyni* (Hamlyn's monkey) | Spain | 1 | 1 | None | Captive | PCR | [799] | ❸ |
| *Cercopithecus kandti* (golden monkey) | China | 9 | 0 | None | Captive | PCR | [790] |  |
| *Cercopithecus kandti* (golden monkey) | China | 29 | 5 | None | Captive | PCR | [791] |  |
| *Cercopithecus kandti* (golden monkey) | China | 47 | 6 | None | Captive | PCR | [800] |  |
| *Cercopithecus mitis* (blue monkey) | China | 8 | 3 | None | Captive | CM | [792] |  |
| *Cercopithecus mona* (mona monkey) | China | 6 | 1 | None | Captive | PCR | [791] |  |
| *Cercopithecus mona* (mona monkey) | Cameroon | 11 | 0 | None | Captive | CM | [801] |  |
| *Cercopithecus neglectus* (de Brazza's monkey) | Japan | 2 | 0 | None | Captive | CADT | [258] | ❸ |
| *Cercopithecus neglectus* (de Brazza's monkey) | Spain | 1 | 1 | None | Captive | PCR | [799] | ❸ |
| *Cercopithecus neglectus* (de Brazza's monkey) | China | 4 | 0 | None | Captive | PCR | [795] | ❸ |
| *Cercopithecus neglectus* (de Brazza's monkey) | China | 3 | 0 | None | Captive | PCR | [791] | ❸ |
| *Cercopithecus neglectus* (de Brazza's monkey) | China | 18 | 0 | None | Captive | CM | [792] |  |
| *Cercopithecus neglectus* (de Brazza's monkey) | Cameroon | 5 | 0 | None | Captive | CM | [801] |  |
| *Cercopithecus neglectus* (de Brazza's monkey) | Vietnam | 1 | 0 | None | Captive | CADT | [209] | ❸ |
| *Cercopithecus neglectus* (de Brazza's monkey) | Spain | 5 | 1 | None | Captive | PCR | [794] |  |
| *Cercopithecus neglectus* (de Brazza's monkey) | China | 7 | 0 | None | Captive | PCR | [6] |  |
| *Cercopithecus nictitans* (greater spot-nosed monkey) | China | 5 | 0 | None | Captive | CM | [792] |  |
| *Cercopithecus nictitans* (greater spot-nosed monkey) | Cameroon | 21 | 0 | None | Captive | CM | [801] |  |
| *Cercopithecus nictitans* (greater spot-nosed monkey) | China | 6 | 0 | None | Captive | PCR | [6] |  |
| *Cercopithecus petaurista* (lesser spot-nosed monkey) | Côte d’Ivoire | 157 | 30 | None | Wild | CM | [798] |  |
| *Cercopithecus preussi* (Preuss's monkey) | Cameroon | 1 | 0 | None | Captive | CM | [801] | ❸ |
| *Cercopithecus roloway* (roloway monkey) | China | 1 | 1 | None | Captive | PCR | [791] | ❸ |
| *Cercopithecus tantalus* (tantalus monkey) | Cameroon | 20 | 0 | None | Captive | CM | [801] |  |
| *Cercopithecus* sp. | China | 4 | 0 | None | Captive | PCR | [790] | ❸ |
| *Cercopithecus* sp. | China | 1 | 0 | None | Captive | PCR | [5] | ❸ |
| *Cercopithecus* sp. | Spain | 7 | 0 | None | Captive | PCR | [788] |  |
| *Chlorocebus aethiops* (grivet) | Japan | 2 | 0 | None | Captive | CADT | [258] | ❸ |
| *Chlorocebus aethiops* (grivet) | Italy | 5 | 0 | None | Captive | CADT | [802] |  |
| *Chlorocebus aethiops* (grivet) | China | 24 | 0 | None | Captive | CM | [792] |  |
| *Chlorocebus pygerythrus* (Vervet monkey) | Bangladesh | 7 | 0 | None | Captive | PCR | [3] |  |
| *Chlorocebus pygerythrus* (Vervet monkey) | Brazil | 3 | 0 | None | Captive | CADT | [638] | ❸ |
| *Chlorocebus sabaeus* (green monkey) | China | 2 | 0 | None | Captive | PCR | [790] | ❸ |
| *Chlorocebus sabaeus* (green monkey) | China | 15 | 3 | None | Captive | PCR | [791] |  |
| *Chlorocebus sabaeus* (green monkey) | China | 3 | 0 | None | Captive | PCR | [5] | ❸ |
| *Chlorocebus sabaeus* (green monkey) | China | 5 | 1 | None | Captive | PCR | [6] |  |
| *Erythrocebus patas* (common patas monkey) | China | 16 | 5 | None | Captive | PCR | [791] |  |
| *Erythrocebus patas* (common patas monkey) | China | 26 | 0 | None | Captive | CM | [792] |  |
| *Erythrocebus patas* (common patas monkey) | Cameroon | 18 | 0 | None | Captive | CM | [801] |  |
| *Erythrocebus patas* (common patas monkey) | China | 4 | 0 | None | Captive | PCR | [5] | ❸ |
| *Erythrocebus patas* (common patas monkey) | China | 4 | 0 | None | Captive | PCR | [6] | ❸ |
| *Miopithecus talapoin* (Angolan talapoin) | Spain | 1 | 0 | None | Captive | PCR | [799] | ❸ |
| **Tribe Papionini** |  |  |  |  |  |  |  |  |
| *Cercocebus agilis* (agile mangabey) | Cen. Af. Rep. | 6 | 0 | None | Wild | PCR | [257] |  |
| *Cercocebus agilis* (agile mangabey) | Cameroon | 13 | 0 | None | Captive | CM | [801] |  |
| *Cercocebus atys* (sooty mangabey) | Spain | 2 | 1 | None | Captive | PCR | [799] | ❸ |
| *Cercocebus atys* (sooty mangabey) | Côte d’Ivoire | 199 | 57 | None | Wild | CM | [798] |  |
| *Cercocebus* *torquatus* (collared mangabey) | Spain | 6 | 5 | None | Captive | PCR | [794] |  |
| *Cercocebus* *torquatus* (collared mangabey) | Croatia | 1 | 0 | None | Captive | CADT | [1] | ❸ |
| *Cercocebus* *torquatus* (collared mangabey) | Italy | 10 | 0 | None | Captive | CADT | [802] |  |
| *Cercocebus* *torquatus* (collared mangabey) | Cameroon | 15 | 0 | None | Captive | CM | [801] |  |
| *Cercocebus* sp. | China | 3 | 0 | None | Captive | PCR | [5] | ❸ |
| *Cercocebus* sp. | Spain | 5 | 0 | None | Captive | PCR | [788] |  |
| *Lophocebus albigina* (grey-cheeked mangabey) | Cameroon | 4 | 0 | None | Captive | CM | [801] | ❸ |
| *Lophocebus* *aterrimus* (black crested mangabey) | Poland | 1 | 0 | None | Captive | CM | [2] | ❸ |
| *Lophocebus* *aterrimus* (black crested mangabey) | Belgium | 5 | 0 | None | Captive | CM | [789] |  |
| *Macaca arctoides* (stump-tailed macaque) | China | 5 | 0 | None | Captive | PCR | [795] |  |
| *Macaca arctoides* (stump-tailed macaque) | China | 13 | 0 | None | Captive | CM | [792] |  |
| *Macaca arctoides* (stump-tailed macaque) | China | 1 | 0 | None | Captive | PCR | [5] | ❸ |
| *Macaca assamensis* (Assam macaque) | China | 6 | 1 | None | Captive | PCR | [790] |  |
| *Macaca assamensis* (Assam macaque) | China | 3 | 0 | None | Captive | PCR | [791] | ❸ |
| *Macaca assamensis* (Assam macaque) | China | 11 | 0 | None | Captive | CM | [792] |  |
| *Macaca assamensis* (Assam macaque) | China | 8 | 0 | None | Wild | PCR | [803] |  |
| *Macaca fascicularis* (crab-eating macaque) | Poland | 1 | 0 | None | Captive | CM | [2] | ❸ |
| *Macaca fascicularis* (crab-eating macaque) | Japan | 1 | 0 | None | Captive | CADT | [258] | ❸ |
| *Macaca fascicularis* (crab-eating macaque) | China | 62 | 1 | None | Captive | PCR | [790] |  |
| *Macaca fascicularis* (crab-eating macaque) | China | 205 | 5 | 2 groups | Laboratory | PCR | [804] |  |
| *Macaca fascicularis* (crab-eating macaque) | China | 18 | 7 | None | Captive | PCR | [791] |  |
| *Macaca fascicularis* (crab-eating macaque) | China | 27 | 0 | None | Captive | CM | [792] |  |
| *Macaca fascicularis* (crab-eating macaque) | Italy | 443 | 0 | None | Captive | CADT | [805] |  |
| *Macaca fascicularis* (crab-eating macaque) | Thailand | 200 | 14 | None | Wild | PCR | [806] |  |
| *Macaca fascicularis* (crab-eating macaque) | China | 60 | 1 | None | Wild | PCR | [807] |  |
| *Macaca fascicularis* (crab-eating macaque) | Brazil | 3 | 0 | None | Captive | CADT | [638] | ❸ |
| *Macaca fascicularis* (crab-eating macaque) | China | 1,452 | 469 | 2 groups | Farmed | PCR | [808] |  |
| *Macaca fascicularis* (crab-eating macaque) | Indonesia | 100 | 10 | None | Wild | CM | [809] |  |
| *Macaca fascicularis* (crab-eating macaque) | Philippines | 35 | 3 | None | Wild | CM | [810] |  |
| *Macaca fascicularis* (crab-eating macaque) | China | 1 | 0 | None | Captive | PCR | [5] | ❸ |
| *Macaca fuscata* (Japanese macaque) | Italy | 22 | 0 | None | Captive | CADT | [802] |  |
| *Macaca fuscata* (Japanese macaque) | China | 9 | 3 | None | Captive | PCR | [790] |  |
| *Macaca fuscata* (Japanese macaque) | China | 3 | 0 | None | Captive | CM | [792] | ❸ |
| *Macaca fuscata* (Japanese macaque) | Brazil | 2 | 0 | None | Captive | CADT | [638] | ❸ |
| *Macaca fuscata* (Japanese macaque) | China | 1 | 0 | None | Captive | PCR | [5] | ❸ |
| *Macaca leonina* (northern pig-tailed macaque) | Bangladesh | 2 | 0 | None | Captive | PCR | [3] | ❸ |
| *Macaca leonina* (northern pig-tailed macaque) | China | 1 | 0 | None | Captive | PCR | [5] | ❸ |
| *Macaca maura* (moor macaque) | China | 34 | 0 | None | Captive | PCR | [790] |  |
| *Macaca mulatta* (rhesus macaque) | China | 411 | 35 | None | Wild | PCR | [811] |  |
| *Macaca mulatta* (rhesus macaque) | China | 1,048 | 22 | None | Captive | PCR | [790] |  |
| *Macaca mulatta* (rhesus macaque) | China | 86 | 3 | None | Captive | PCR | [795] |  |
| *Macaca mulatta* (rhesus macaque) | China | 106 | 9 | None | Captive | PCR | [791] |  |
| *Macaca mulatta* (rhesus macaque) | China | 36 | 0 | None | Captive | CM | [792] |  |
| *Macaca mulatta* (rhesus macaque) | India | 170 | 53 | None | Wild | CADT/PCR | [239] |  |
| *Macaca mulatta* (rhesus macaque) | China | 101 | 1 | None | Wild | PCR | [807] |  |
| *Macaca mulatta* (rhesus macaque) | Brazil | 2 | 0 | None | Captive | CADT | [638] | ❸ |
| *Macaca mulatta* (rhesus macaque) | Bangladesh | 62 | 3 | None | Captive | PCR | [3] |  |
| *Macaca mulatta* (rhesus macaque) | China | 310 | 22 | None | Laboratory | PCR | [812] |  |
| *Macaca mulatta* (rhesus macaque) | China | 320 | 23 | None | Wild | PCR | [803] |  |
| *Macaca mulatta* (rhesus macaque) | China | 7 | 0 | None | Captive | PCR | [6] |  |
| *Macaca nemestrina* (southern pig-tailed macaque) | Japan | 2 | 0 | None | Captive | CADT | [258] | ❸ |
| *Macaca nemestrina* (southern pig-tailed macaque) | China | 1 | 0 | None | Captive | PCR | [790] | ❸ |
| *Macaca nemestrina* (southern pig-tailed macaque) | China | 16 | 9 | None | Captive | PCR | [791] |  |
| *Macaca nemestrina* (southern pig-tailed macaque) | China | 32 | 0 | None | Captive | CM | [792] |  |
| *Macaca nemestrina* (southern pig-tailed macaque) | Brazil | 1 | 0 | None | Captive | CADT | [638] | ❸ |
| *Macaca* *nigra* (Celebes crested macaque) | Belgium | 2 | 0 | None | Captive | CM | [789] | ❸ |
| *Macaca* *nigra* (Celebes crested macaque) | China | 2 | 0 | None | Captive | PCR | [791] | ❸ |
| *Macaca* *nigra* (Celebes crested macaque) | China | 3 | 0 | None | Captive | CM | [792] | ❸ |
| *Macaca radiata* (bonnet macaque) | India | 161 | 2 | None | Wild | CM | [813] |  |
| *Macaca silenus* (lion-tailed macaque) | Japan | 2 | 0 | None | Captive | CADT | [258] | ❸ |
| *Macaca silenus* (lion-tailed macaque) | China | 7 | 0 | None | Captive | CM | [792] |  |
| *Macaca sylvanus* (barbary macaque) | Spain | 8 | 3 | None | Captive | PCR | [794] |  |
| *Macaca thibetana* (Tibetan macaque) | China | 1 | 0 | None | Captive | PCR | [790] | ❸ |
| *Macaca thibetana* (Tibetan macaque) | China | 2 | 0 | None | Captive | PCR | [791] | ❸ |
| *Macaca thibetana* (Tibetan macaque) | China | 14 | 0 | None | Captive | CM | [792] |  |
| *Macaca thibetana* (Tibetan macaque) | China | 7 | 2 | None | Captive | PCR | [6] |  |
| *Macaca* sp. | China | 15 | 0 | None | Captive | PCR | [5] |  |
| *Macaca* sp. | Spain | 13 | 1 | None | Captive | PCR | [788] |  |
| *Mandrillus leucophaeus* (drill) | Japan | 3 | 0 | None | Captive | CADT | [258] | ❸ |
| *Mandrillus leucophaeus* (drill) | Spain | 1 | 1 | None | Captive | PCR | [799] | ❸ |
| *Mandrillus leucophaeus* (drill) | China | 2 | 0 | None | Captive | CM | [792] | ❸ |
| *Mandrillus leucophaeus* (drill) | Cameroon | 24 | 0 | None | Captive | CM | [801] |  |
| *Mandrillus leucophaeus* (drill) | Spain | 8 | 0 | None | Captive | PCR | [794] |  |
| *Mandrillus* *sphinx* (mandrill) | Belgium | 14 | 1 | None | Captive | CM | [789] |  |
| *Mandrillus* *sphinx* (mandrill) | Italy | 16 | 0 | None | Captive | CADT | [802] |  |
| *Mandrillus* *sphinx* (mandrill) | Brazil | 2 | 0 | None | Captive | CM | [785] | ❸ |
| *Mandrillus* *sphinx* (mandrill) | China | 49 | 0 | None | Captive | PCR | [790] |  |
| *Mandrillus* *sphinx* (mandrill) | China | 5 | 0 | None | Captive | PCR | [795] |  |
| *Mandrillus* *sphinx* (mandrill) | China | 23 | 2 | None | Captive | PCR | [791] |  |
| *Mandrillus* *sphinx* (mandrill) | China | 47 | 0 | None | Captive | CM | [792] |  |
| *Mandrillus* *sphinx* (mandrill) | Brazil | 2 | 0 | None | Captive | CADT | [638] | ❸ |
| *Mandrillus* *sphinx* (mandrill) | Cameroon | 18 | 1 | None | Captive | CM | [801] |  |
| *Mandrillus* *sphinx* (mandrill) | China | 4 | 0 | None | Captive | PCR | [6] | ❸ |
| *Mandrillus* sp. | Spain | 18 | 1 | None | Captive | PCR | [788] |  |
| *Papio anubis* (olive baboon) | China | 12 | 2 | None | Captive | PCR | [790] |  |
| *Papio anubis* (olive baboon) | China | 5 | 0 | None | Captive | PCR | [791] |  |
| *Papio anubis* (olive baboon) | China | 22 | 0 | None | Captive | CM | [792] |  |
| *Papio anubis* (olive baboon) | Brazil | 1 | 0 | None | Captive | CADT | [638] | ❸ |
| *Papio anubis* (olive baboon) | Cameroon | 45 | 0 | None | Captive | CM | [801] |  |
| *Papio anubis* (olive baboon) | China | 10 | 1 | None | Captive | PCR | [6] |  |
| *Papio cynocephalus* (yellow baboon) | China | 5 | 2 | None | Captive | PCR | [791] |  |
| *Papio cynocephalus* (yellow baboon) | China | 7 | 0 | None | Captive | CM | [792] |  |
| *Papio cynocephalus* (yellow baboon) | Brazil | 2 | 0 | None | Captive | CADT | [638] | ❸ |
| *Papio cynocephalus* (yellow baboon) | China | 1 | 0 | None | Captive | PCR | [6] | ❸ |
| *Papio hamadryas* (hamadryas baboon) | Belgium | 20 | 1 | None | Captive | CM | [789] |  |
| *Papio hamadryas* (hamadryas baboon) | Brazil | 1 | 0 | None | Captive | CM | [785] | ❸ |
| *Papio hamadryas* (hamadryas baboon) | China | 6 | 0 | None | Captive | PCR | [790] |  |
| *Papio hamadryas* (hamadryas baboon) | China | 12 | 0 | None | Captive | PCR | [795] |  |
| *Papio hamadryas* (hamadryas baboon) | China | 21 | 1 | None | Captive | PCR | [791] |  |
| *Papio hamadryas* (hamadryas baboon) | China | 69 | 0 | None | Captive | CM | [792] |  |
| *Papio hamadryas* (hamadryas baboon) | Brazil | 1 | 0 | None | Captive | CADT | [638] | ❸ |
| *Papio hamadryas* (hamadryas baboon) | Bangladesh | 5 | 0 | None | Captive | PCR | [3] |  |
| *Papio hamadryas* (hamadryas baboon) | China | 4 | 0 | None | Captive | PCR | [6] | ❸ |
| *Papio papio* (Guinea baboon) | Brazil | 2 | 0 | None | Captive | CM | [785] | ❸ |
| *Papio papio* (Guinea baboon) | China | 4 | 0 | None | Captive | CM | [792] | ❸ |
| *Papio papio* (Guinea baboon) | Brazil | 1 | 0 | None | Captive | CADT | [638] | ❸ |
| *Papio* sp. | Spain | 11 | 2 | None | Captive | PCR | [788] |  |
| *Theropithecus* *gelada* (Gelada) | Spain | 3 | 0 | None | Captive | PCR | [788] | ❸ |
| **Subfamily Colobinae** |  |  |  |  |  |  |  |  |
| **African group** |  |  |  |  |  |  |  |  |
| *Colobus guereza* (mantled guereza) | Poland | 1 | 0 | None | Captive | CM | [2] | ❸ |
| *Colobus guereza* (mantled guereza) | Belgium | 7 | 0 | None | Captive | CM | [789] |  |
| *Colobus guereza* (mantled guereza) | Uganda | 25 | 0 | None | Wild | CADT | [797] |  |
| *Colobus guereza* (mantled guereza) | Uganda | 29 | 1 | None | Captive | PCR | [92] |  |
| *Colobus guereza* (mantled guereza) | Croatia | 4 | 1 | None | Captive | CADT/PCR | [1] | ❸ |
| *Colobus guereza* (mantled guereza) | China | 4 | 0 | None | Captive | CM | [792] | ❸ |
| *Colobus guereza* (mantled guereza) | Uganda | 17 | 0 | None | Wild | CADT | [210] |  |
| *Colobus guereza* (mantled guereza) | China | 3 | 0 | None | Captive | PCR | [5] | ❸ |
| *Colobus guereza* (mantled guereza) | China | 2 | 1 | None | Captive | PCR | [6] | ❸ |
| *Colobus polykomos* (king colobus) | China | 7 | 1 | None | Captive | PCR | [791] |  |
| *Colobus polykomos* (king colobus) | Côte d’Ivoire | 125 | 7 | None | Wild | CM | [798] |  |
| *Colobus polykomos* (King colobus) | China | 9 | 0 | None | Captive | CM | [792] |  |
| *Colobus* sp. | Spain | 13 | 0 | None | Captive | PCR | [788] |  |
| *Piliocolobus tephrosceles* (Ugandan red colobus) | Uganda | 35 | 2 | None | Wild | CADT | [797] |  |
| *Piliocolobus tephrosceles* (Ugandan red colobus) | Uganda | 30 | 7 | None | Captive | PCR | [92] |  |
| *Piliocolobus badius* (western red colobus) | Côte d’Ivoire | 245 | 18 | None | Wild | CM | [798] |  |
| *Piliocolobus* sp. | China | 1 | 0 | None | Captive | PCR | [790] | ❸ |
| *Procolobus verus* (olive colobus) | Côte d’Ivoire | 129 | 12 | None | Wild | CM | [798] |  |
| **Langur group** |  |  |  |  |  |  |  |  |
| *Presbytis hosei* (hose's langur) | China | 1 | 0 | None | Captive | PCR | [790] | ❸ |
| *Semnopithecus* *entellus* (northern plains gray langur) | Belgium | 5 | 0 | None | Captive | CM | [789] |  |
| *Semnopithecus* *entellus* (northern plains gray langur) | Croatia | 3 | 2 | None | Captive | CADT | [1] | ❸ |
| *Semnopithecus* *entellus* (northern plains gray langur) | Bangladesh | 2 | 0 | None | Captive | PCR | [3] | ❸ |
| *Semnopithecus johnii* (Nilgiri langur) | Bangladesh | 5 | 1 | None | Captive | PCR | [3] |  |
| *Trachypithecus auratus* (east Javan langur) | Belgium | 10 | 0 | None | Captive | CM | [789] |  |
| *Trachypithecus* *francoisi* (François langur) | Japan | 2 | 0 | None | Captive | CADT | [258] | ❸ |
| *Trachypithecus* *francoisi* (François langur) | China | 15 | 0 | None | Captive | PCR | [790] |  |
| *Trachypithecus* *francoisi* (François langur) | China | 10 | 1 | None | Captive | PCR | [791] |  |
| *Trachypithecus* *francoisi* (François langur) | China | 78 | 0 | None | Captive | CM | [792] |  |
| *Trachypithecus* *francoisi* (François langur) | China | 2 | 0 | None | Captive | PCR | [6] | ❸ |
| *Trachypithecus* *leucocephalus* (white-headed langur) | China | 147 | 1 | None | Captive | PCR | [790] |  |
| *Trachypithecus* *leucocephalus* (white-headed langur) | China | 5 | 0 | None | Captive | CM | [792] |  |
| *Trachypithecus* *pileatus* (capped langur) | China | 1 | 0 | None | Captive | CM | [792] | ❸ |
| *Trachypithecus* *phayrei* (Phayre’s leaf monkey) | China | 4 | 0 | None | Captive | CM | [792] | ❸ |
| *Trachypithecus* sp. | Spain | 1 | 0 | None | Captive | PCR | [788] | ❸ |
| **Odd-nosed group** |  |  |  |  |  |  |  |  |
| *Rhinopithecus bieti* (black snub-nosed monkey) | China | 16 | 0 | None | Captive | CM | [792] |  |
| *Rhinopithecus bieti* (black snub-nosed monkey) | China | 20 | 2 | None | Wild | PCR | [803] |  |
| *Rhinopithecus brelichi* (gray snub-nosed monkey) | China | 1 | 0 | None | Captive | CM | [792] | ❸ |
| *Rhinopithecus roxellana* (golden snub-nosed monkey) | China | 60 | 0 | None | Captive | PCR | [790] |  |
| *Rhinopithecus roxellana* (golden snub-nosed monkey) | China | 63 | 0 | None | Captive | PCR | [795] |  |
| *Rhinopithecus roxellana* (golden snub-nosed monkey) | China | 95 | 0 | None | Captive | CM | [792] |  |
| *Rhinopithecus roxellana* (golden snub-nosed monkey) | China | 9 | 0 | None | Wild | PCR | [807] |  |
| *Rhinopithecus roxellana* (golden snub-nosed monkey) | China | 21 | 8 | None | Captive | PCR | [329] |  |
| *Rhinopithecus* sp. | China | 3 | 0 | None | Captive | PCR | [5] | ❸ |
| *Rhinopithecus* sp. | China | 15 | 0 | None | Captive | PCR | [6] |  |
| **Family Hominidae** |  |  |  |  |  |  |  |  |
| *Gorilla beringei* (eastern gorilla) | Uganda | 41 | 0 | None | Habituated | CM | [814] |  |
| *Gorilla beringei* (eastern gorilla) | Uganda | 100 | 2 | None | Wild | CADT/PCR | [46] |  |
| *Gorilla beringei* (eastern gorilla) | Belgium | 2 | 1 | None | Captive | CM | [789] | ❸ |
| *Gorilla beringei* (eastern gorilla) | Rwanda | 130 | 11 | None | Wild | CADT/PCR | [129] |  |
| *Gorilla beringei* (eastern gorilla) | Uganda | 68 | 0 | None | Habituated | PCR | [166] |  |
| *Gorilla beringei* (eastern gorilla) | Uganda | 147 | 0 | None | Captive | CADT | [815] |  |
| *Gorilla* *gorilla* (western gorilla) | Belgium | 1 | 0 | None | Captive | CM | [789] | ❸ |
| *Gorilla* *gorilla* (western gorilla) | Gabon | 95 | 20 | None | Wild | CADT | [816] |  |
| *Gorilla* *gorilla* (western gorilla) | Spain | 1 | 1 | None | Captive | PCR | [799] | ❸ |
| *Gorilla* *gorilla* (western gorilla) | Cen. Af. Rep. | 201 | 2 | None | Habituated | PCR | [257] |  |
| *Gorilla* *gorilla* (western gorilla) | China | 9 | 0 | None | Captive | CM | [792] |  |
| *Gorilla* *gorilla* (western gorilla) | Cameroon | 24 | 1 | None | Captive | CM | [801] |  |
| *Gorilla* *gorilla* (western gorilla) | Spain | 56 | 15 | None | Captive | PCR | [788] |  |
| *Gorilla* sp. | China | 1 | 0 | None | Captive | PCR | [5] | ❸ |
| *Pan* *paniscus* (bonobo) | Belgium | 9 | 0 | None | Captive | CM | [789] |  |
| *Pan troglodytes* (chimpanzee) | Poland | 4 | 1 | None | Captive | CM | [2] | ❸ |
| *Pan troglodytes* (chimpanzee) | Japan | 7 | 0 | None | Captive | CADT | [258] |  |
| *Pan troglodytes* (chimpanzee) | Belgium | 13 | 4 | None | Captive | CM | [789] |  |
| *Pan troglodytes* (chimpanzee) | Croatia | 5 | 2 | None | Captive | CADT | [1] |  |
| *Pan troglodytes* (chimpanzee) | Italy | 41 | 0 | None | Captive | CADT | [802] |  |
| *Pan troglodytes* (chimpanzee) | Spain | 1 | 1 | None | Captive | PCR | [799] | ❸ |
| *Pan troglodytes* (chimpanzee) | China | 2 | 0 | None | Captive | PCR | [795] | ❸ |
| *Pan troglodytes* (chimpanzee) | China | 14 | 0 | None | Captive | PCR | [791] |  |
| *Pan troglodytes* (chimpanzee) | China | 53 | 0 | None | Captive | CM | [792] |  |
| *Pan troglodytes* (chimpanzee) | Brazil | 3 | 0 | None | Captive | CADT | [638] | ❸ |
| *Pan troglodytes* (chimpanzee) | Russia | 10 | 0 | None | Pet | CM | [590] |  |
| *Pan troglodytes* (chimpanzee) | Cameroon | 73 | 0 | None | Captive | CM | [801] |  |
| *Pan troglodytes* (chimpanzee) | Uganda | 30 | 0 | None | Wild | CADT | [210] |  |
| *Pan troglodytes* (chimpanzee) | China | 3 | 0 | None | Captive | PCR | [5] | ❸ |
| *Pan troglodytes* (chimpanzee) | Australia | 26 | 14 | None | Captive | PCR | [817] |  |
| *Pan troglodytes* (chimpanzee) | China | 6 | 0 | None | Captive | PCR | [6] |  |
| *Pan troglodytes* (chimpanzee) | Serbia | 2 | 0 | None | Captive | PCR | [8] | ❸ |
| *Pan* sp. | Spain | 59 | 16 | None | Captive | PCR | [788] |  |
| *Pongo abelii* (orangutan) | Brazil | 2 | 0 | None | Captive | CADT | [638] | ❸ |
| *Pongo* *pygmaeus* (orangutan) | Poland | 3 | 0 | None | Captive | CM | [2] | ❸ |
| *Pongo* *pygmaeus* (orangutan) | Japan | 4 | 0 | None | Captive | CADT | [258] | ❸ |
| *Pongo* *pygmaeus* (orangutan) | Belgium | 2 | 0 | None | Captive | CM | [789] | ❸ |
| *Pongo* *pygmaeus* (orangutan) | Italy | 13 | 0 | None | Captive | CADT | [802] |  |
| *Pongo* *pygmaeus* (orangutan) | China | 23 | 5 | None | Captive | PCR | [791] |  |
| *Pongo* *pygmaeus* (orangutan) | China | 12 | 0 | None | Captive | CM | [792] |  |
| *Pongo* *pygmaeus* (orangutan) | Serbia | 1 | 0 | None | Captive | PCR | [8] | ❸ |
| *Pongo* sp. (orangutan) | China | 3 | 0 | None | Captive | PCR | [790] | ❸ |
| *Pongo* sp. (orangutan) | China | 1 | 0 | None | Captive | PCR | [5] | ❸ |
| *Pongo* sp. (orangutan) | Spain | 37 | 4 | None | Captive | PCR | [788] |  |
| **Family Hylobatidae** |  |  |  |  |  |  |  |  |
| *Hoolock hoolock* (western hoolock gibbon) | China | 14 | 0 | None | Captive | CM | [792] |  |
| *Hylobates agilis* (agile gibbon) | Thailand | 2 | 0 | None | Captive | CADT | [818] | ❸ |
| *Hylobates lar* (lar gibbon) | Belgium | 8 | 2 | None | Captive | CM | [789] |  |
| *Hylobates lar* (lar gibbon) | Croatia | 2 | 1 | None | Captive | CADT/PCR | [1] | ❸ |
| *Hylobates lar* (lar gibbon) | China | 8 | 0 | None | Captive | PCR | [791] |  |
| *Hylobates lar* (lar gibbon) | China | 1 | 0 | None | Captive | CM | [792] | ❸ |
| *Hylobates lar* (lar gibbon) | Brazil | 22 | 3 | None | Captive | CM | [320] |  |
| *Hylobates lar* (lar gibbon) | Thailand | 38 | 1 | None | Captive | CADT/PCR | [818] |  |
| *Hylobates moloch* (silvery gibbon) | China | 3 | 0 | None | Captive | PCR | [791] | ❸ |
| *Hylobates pileatus* (pileated gibbon) | China | 1 | 0 | None | Captive | PCR | [791] | ❸ |
| *Hylobates pileatus* (pileated gibbon) | Thailand | 15 | 0 | None | Captive | CADT | [818] |  |
| *Hylobates* sp. | China | 54 | 0 | None | Captive | PCR | [790] |  |
| *Hylobates* sp. | Spain | 8 | 1 | None | Captive | PCR | [788] |  |
| *Hylobates* sp. | China | 2 | 0 | None | Captive | PCR | [5] | ❸ |
| *Nomascus* *concolor* (black crested gibbon) | Belgium | 1 | 0 | None | Captive | CM | [789] | ❸ |
| *Nomascus* *concolor* (black crested gibbon) | China | 1 | 0 | None | Captive | CM | [792] | ❸ |
| *Nomascus* *leucogenys* (white-cheeked crested gibbon) | Belgium | 4 | 1 | None | Captive | CM | [789] | ❸ |
| *Nomascus* *leucogenys* (white-cheeked crested gibbon) | China | 14 | 2 | None | Captive | PCR | [791] |  |
| *Nomascus* *leucogenys* (white-cheeked crested gibbon) | China | 57 | 0 | None | Captive | CM | [792] |  |
| *Nomascus* *leucogenys* (white-cheeked crested gibbon) | China | 36 | 14 | None | Wild | PCR | [807] |  |
| *Nomascus* *leucogenys* (white-cheeked crested gibbon) | Spain | 7 | 1 | None | Captive | PCR | [794] |  |
| *Nomascus* *leucogenys* (white-cheeked crested gibbon) | China | 3 | 0 | None | Captive | PCR | [6] | ❸ |
| *Nomascus* sp. | Spain | 2 | 0 | None | Captive | PCR | [788] | ❸ |
| *Symphalangus syndactylus* (Siamang) | Japan | 2 | 0 | None | Captive | CADT | [258] | ❸ |
| *Symphalangus syndactylus* (Siamang) | Belgium | 6 | 2 | None | Captive | CM | [789] |  |
| **Family Galagidae** |  |  |  |  |  |  |  |  |
| *Galago* sp. | Russia | 10 | 0 | None | Pet | CM | [590] |  |
| *Galago* sp. | Spain | 2 | 1 | None | Captive | PCR | [788] | ❸ |
| *Otolemur crassicaudatus* (thick-tailed bushbabies) | Belgium | 2 | 0 | None | Captive | CM | [789] | ❸ |
| *Otolemur crassicaudatus* (thick-tailed bushbabies) | China | 13 | 0 | None | Captive | CM | [792] |  |
| **Family Indriidae** |  |  |  |  |  |  |  |  |
| *Propithecus diadema* (diademed sifaka) | Madagascar | 43 | 0 | None | Wild | CADT | [240] |  |
| **Family Lemuridae** |  |  |  |  |  |  |  |  |
| *Eulemur fulvus* (common brown lemur) | Spain | 1 | 1 | None | Captive | PCR | [799] | ❸ |
| *Eulemur fulvus* (common brown lemur) | Spain | 2 | 0 | None | Captive | PCR | [794] | ❸ |
| *Eulemur fulvus* (common brown lemur) | Serbia | 1 | 0 | None | Captive | PCR | [8] | ❸ |
| *Eulemur macaco* (clack lemur) | Italy | 4 | 0 | None | Captive | CADT | [802] | ❸ |
| *Eulemur macaco* (clack lemur) | China | 1 | 0 | None | Captive | CM | [792] | ❸ |
| *Eulemur mongoz* (mongoose lemur) | Spain | 1 | 0 | None | Captive | PCR | [799] | ❸ |
| *Eulemur rubriventer* (red-bellied lemur) | Spain | 1 | 1 | None | Captive | PCR | [799] | ❸ |
| *Eulemur rufus* (red lemur) | Spain | 1 | 1 | None | Captive | PCR | [799] | ❸ |
| *Eulemur* sp. | Spain | 12 | 3 | None | Captive | PCR | [788] |  |
| *Hapalemur aureus* (golden bamboo lemur) | Spain | 1 | 1 | None | Captive | PCR | [799] | ❸ |
| *Hapalemur griseus* (eastern lesser bamboo lemur) | Madagascar | 44 | 0 | None | Wild | CADT | [240] |  |
| *Lemur catta* (ring-tailed lemur) | Belgium | 20 | 9 | None | Captive | CM | [789] |  |
| *Lemur catta* (ring-tailed lemur) | Croatia | 4 | 3 | None | Captive | CADT | [1] | ❸ |
| *Lemur catta* (ring-tailed lemur) | Italy | 17 | 8 | None | Captive | CADT | [802] |  |
| *Lemur catta* (ring-tailed lemur) | Spain | 3 | 3 | None | Captive | PCR | [799] | ❸ |
| *Lemur catta* (ring-tailed lemur) | China | 23 | 0 | None | Captive | PCR | [790] |  |
| *Lemur catta* (ring-tailed lemur) | China | 45 | 26 | None | Captive | PCR | [791] |  |
| *Lemur catta* (ring-tailed lemur) | China | 139 | 0 | None | Captive | CM | [792] |  |
| *Lemur catta* (ring-tailed lemur) | Spain | 2 | 0 | None | Captive | PCR | [794] | ❸ |
| *Lemur catta* (ring-tailed lemur) | China | 43 | 2 | None | Captive | PCR | [800] |  |
| *Lemur catta* (ring-tailed lemur) | China | 21 | 4 | None | Captive | PCR | [6] |  |
| *Lemur catta* (ring-tailed lemur) | Serbia | 1 | 1 | None | Captive | PCR | [8] | ❸ |
| *Lemur* sp. | Spain | 42 | 16 | None | Captive | PCR | [788] |  |
| *Varecia rubra* (red ruffed lemur) | Belgium | 10 | 0 | None | Captive | CM | [789] |  |
| *Varecia rubra* (red ruffed lemur) | Italy | 5 | 0 | None | Captive | CADT | [802] |  |
| *Varecia rubra* (red ruffed lemur) | Spain | 1 | 1 | None | Captive | PCR | [799] | ❸ |
| *Varecia variegata* (black-and-white ruffed lemur) | Japan | 1 | 0 | None | Captive | CADT | [258] | ❸ |
| *Varecia variegata* (black-and-white ruffed lemur) | Belgium | 10 | 1 | None | Captive | CM | [789] |  |
| *Varecia variegata* (black-and-white ruffed lemur) | China | 28 | 0 | None | Captive | PCR | [790] |  |
| *Varecia variegata* (black-and-white ruffed lemur) | China | 5 | 0 | None | Captive | PCR | [791] |  |
| *Varecia variegata* (black-and-white ruffed lemur) | China | 1 | 0 | None | Captive | CM | [792] | ❸ |
| *Varecia variegata* (black-and-white ruffed lemur) | Spain | 3 | 0 | None | Captive | PCR | [799] | ❸ |
| *Varecia variegata* (black-and-white ruffed lemur) | Spain | 5 | 0 | None | Captive | PCR | [794] |  |
| *Varecia variegata* (black-and-white ruffed lemur) | China | 7 | 1 | None | Captive | PCR | [6] |  |
| *Varecia* sp. | Spain | 14 | 1 | None | Captive | PCR | [788] |  |
| **Family Lorisidae** |  |  |  |  |  |  |  |  |
| *Nycticebus coucang* (Sunda slow loris) | Croatia | 1 | 0 | None | Captive | CADT | [1] | ❸ |
| *Nycticebus coucang* (Sunda slow loris) | China | 10 | 0 | None | Captive | PCR | [790] |  |
| *Nycticebus coucang* (Sunda slow loris) | China | 4 | 2 | None | Captive | PCR | [791] | ❸ |
| *Nycticebus coucang* (Sunda slow loris) | China | 10 | 0 | None | Captive | CM | [792] |  |
| *Nycticebus* sp. | Spain | 1 | 0 | None | Captive | PCR | [788] | ❸ |
| *Perodicticus potto* (west African potto) | Croatia | 1 | 0 | None | Captive | CADT | [1] | ❸ |
| *Perodicticus* sp. | Spain | 1 | 0 | None | Captive | PCR | [788] | ❸ |
| *Xanthonycticebus* *pygmaeus* (pygmy slow loris) | China | 12 | 0 | None | Captive | CM | [792] |  |
| **Order Proboscidea** |  |  |  |  |  |  |  |  |
| **Family Elephantidae** |  |  |  |  |  |  |  |  |
| *Elephas maximus* (Asian elephant) | Poland | 1 | 0 | None | Captive | CM | [2] | ❸ |
| *Elephas maximus* (Asian elephant) | Japan | 3 | 0 | None | Captive | CADT | [258] | ❸ |
| *Elephas maximus* (Asian elephant) | China | 2 | 1 | None | Captive | PCR | [15] | ❸ |
| *Elephas maximus* (Asian elephant) | Bangladesh | 3 | 0 | None | Captive | PCR | [3] | ❸ |
| *Elephas maximus* (Asian elephant) | China | 6 | 0 | None | Captive | PCR | [6] |  |
| *Loxodonta africana* (African bush elephant) | Cen. Af. Rep. | 52 | 0 | None | Wild | PCR | [257] |  |
| **Order Rodentia** |  |  |  |  |  |  |  |  |
| **Family Calomyscidae** |  |  |  |  |  |  |  |  |
| *Calomyscus elburzensis* (Goodwin's mouse-like hamster) | Iran | 1 | 0 | None | Wild | CM | [819] | ❸ |
| **Family Castoridae** |  |  |  |  |  |  |  |  |
| *Castor canadensis* (North American beaver) | USA | 58 | 2 | None | Wild | CM | [306] |  |
| *Castor canadensis* (North American beaver) | USA | 68 | 36 | None | Wild | CM | [820] |  |
| *Castor canadensis* (North American beaver) | Canada | 299 | 58 | None | Wild | CM | [821] |  |
| *Castor canadensis* (North American beaver) | USA | 482 | 192 | 2 groups | Wild | CM | [822] |  |
| *Castor canadensis* (North American beaver) | USA | 775 | 337 | 2 groups | Wild | CM | [822] |  |
| *Castor canadensis* (North American beaver) | USA | 313 | 76 | None | Wild | CM | [314] |  |
| *Castor canadensis* (North American beaver) | USA | 41 | 2 | None | Wild | CM | [823] |  |
| *Castor canadensis* (North American beaver) | USA | 9 | 1 | None | Wild | CM | [824] |  |
| *Castor canadensis* (North American beaver) | USA | 662 | 271 | None | Wild | CM | [825] |  |
| *Castor canadensis* (North American beaver) | Canada | 14 | 4 | None | Wild | CM | [826] |  |
| *Castor canadensis* (North American beaver) | USA | 63 | 0 | None | Wild | CM | [827] |  |
| *Castor canadensis* (North American beaver) | Canada | 94 | 9 | None | Wild | CADT | [828] |  |
| *Castor canadensis* (North American beaver) | USA | 100 | 30 | None | Wild | CADT | [829] |  |
| *Castor canadensis* (North American beaver) | Canada | 334 | 29 | None | Wild | CADT | [7] |  |
| *Castor canadensis* (North American beaver) | USA | 107 | 33 | 2 groups | Wild | CM | [830] |  |
| *Castor canadensis* (North American beaver) | USA | 62 | 4 | 3 groups | Wild | CADT | [831] |  |
| *Castor fiber* (Eurasian beaver) | Norway | 241 | 0 | None | Wild | CM | [832] |  |
| *Castor fiber* (Eurasian beaver) | Poland | 52 | 4 | None | Fa/Wi | CADT | [10] |  |
| **Family Caviidae** |  |  |  |  |  |  |  |  |
| **Subfamily Caviinae [guinea pigs]** |  |  |  |  |  |  |  |  |
| *Cavia aperea aperea* (cavy) | Brazil | 5 | 5 | None | Wild | CM | [833] |  |
| *Cavia porcellus* (guinea pig) | Germany | 27 | 0 | None | Pet | CADT | [653] |  |
| *Cavia porcellus* (guinea pig) | Germany | 121 | 5 | None | Pet | CADT | [747] |  |
| *Cavia porcellus* (guinea pig) | Russia | 132 | 5 | None | Pet | CM | [590] |  |
| *Cavia porcellus* (guinea pig) | Ecuador | 48 | 0 | None | Farmed | CM | [351] |  |
| *Cavia porcellus* (guinea pig) | China | 92 | 0 | None | Laboratory | PCR | [834] |  |
| *Cavia porcellus* (guinea pig) | China | 7 | 0 | None | Wild | PCR | [317] |  |
| **Subfamily Dolichotinae** |  |  |  |  |  |  |  |  |
| *Dolichotis patagonum* (Patagonian mara) | Poland | 2 | 0 | None | Captive | CM | [2] | ❸ |
| *Dolichotis patagonum* (Patagonian mara) | Croatia | 1 | 1 | None | Captive | CADT/PCR | [1] | ❸ |
| *Dolichotis patagonum* (Patagonian mara) | Serbia | 2 | 0 | None | Captive | PCR | [8] | ❸ |
| *Dolichotis patagonum* (Patagonian mara) | China | 15 | 6 | None | Captive | PCR | [6] |  |
| **Subfamily Hydrochoerinae [capybaras]** |  |  |  |  |  |  |  |  |
| *Hydrochoerus hydrochaeris* (capybara) | Croatia | 2 | 1 | None | Captive | CADT | [1] | ❸ |
| *Hydrochoerus hydrochaeris* (capybara) | Colombia | 360 | 6 | None | Wild | CM | [835] |  |
| *Hydrochoerus hydrochaeris* (capybara) | Brazil | 30 | 30 | None | Wild | PCR | [836] |  |
| *Hydrochoerus hydrochaeris* (capybara) | Colombia | 46 | 0 | None | Wild | CADT | [837] |  |
| *Hydrochoerus hydrochaeris* (capybara) | Serbia | 2 | 0 | None | Captive | PCR | [8] | ❸ |
| *Hydrochoerus hydrochaeris* (capybara) | Brazil | 247 | 0 | None | Wild | PCR | [838] |  |
| **Family Chinchillidae** |  |  |  |  |  |  |  |  |
| *Chinchilla lanigera* (long-tailed chinchilla) | Brazil | 250 | 20 | 2 groups | Farmed | CM | [839] |  |
| *Chinchilla lanigera* (long-tailed chinchilla) | Germany | 195 | 130 | None | Pet | CADT | [653] |  |
| *Chinchilla lanigera* (long-tailed chinchilla) | Brazil | 220 | 80 | None | Wild | CM | [840] |  |
| *Chinchilla lanigera* (long-tailed chinchilla) | China | 96 | 36 | None | Pet | CM | [841] |  |
| *Chinchilla lanigera* (long-tailed chinchilla) | Italy | 104 | 41 | None | Farmed | CADT/PCR | [842] |  |
| *Chinchilla lanigera* (long-tailed chinchilla) | Germany | 531 | 326 | None | Pet | CADT/PCR | [747] |  |
| *Chinchilla lanigera* (long-tailed chinchilla) | Brazil | 100 | 38 | None | Farmed | CM | [843] |  |
| *Chinchilla lanigera* (long-tailed chinchilla) | China | 140 | 38 | None | Pet | PCR | [844] |  |
| *Chinchilla lanigera* (long-tailed chinchilla) | Argentina | 244 | 84 | None | Captive | CM | [845] |  |
| *Chinchilla lanigera* (long-tailed chinchilla) | Romania | 341 | 190 | 2 groups | Farmed | CM/PCR | [846] |  |
| *Chinchilla lanigera* (long-tailed chinchilla) | Russia | 217 | 103 | None | Pet | CM | [590] |  |
| *Chinchilla lanigera* (long-tailed chinchilla) | UAE | 1 | 1 | None | Captive | PCR | [218] | ❸ |
| *Lagostomus maximus* (plains vizcacha) | Poland | 2 | 0 | None | Captive | CM | [2] | ❸ |
| **Family Cricetidae** |  |  |  |  |  |  |  |  |
| **Subfamily Arvicolinae** |  |  |  |  |  |  |  |  |
| **Tribe Arvicolini** |  |  |  |  |  |  |  |  |
| *Arvicola amphibius* (Northern water vole) | UK | 109 | 33 | None | Wild | CADT | [847] |  |
| *Arvicola amphibius* (Northern water vole) | Iran | 1 | 0 | None | Wild | CM | [819] | ❸ |
| *Arvicola amphibius* (Northern water vole) | Iran | 5 | 1 | None | Wild | PCR | [848] |  |
| *Arvicola sapidus* (Southern water vole) | Portugal | 52 | 51 | None | Wild | PCR | [741] |  |
| **Tribe Clethrionomyini** |  |  |  |  |  |  |  |  |
| *Clethrionomys gapperi* (Southern red-backed vole) | USA | 21 | 20 | None | Wild | CM | [306] |  |
| *Clethrionomys glareolus* (bank vole) | Germany | 72 | 25 | None | Wild | CM | [28] |  |
| *Clethrionomys glareolus* (bank vole) | Poland | 445 | 418 | 3 groups | Wild | CM | [849] |  |
| *Clethrionomys glareolus* (bank vole) | Poland | 8 | 8 | None | Wild | CADT | [9] |  |
| *Clethrionomys glareolus* (bank vole) | Poland | 1,457 | 849 | None | Wild | CM | [850] |  |
| *Clethrionomys glareolus* (bank vole) | Poland | 69 | 24 | None | Wild | CM/PCR | [851] |  |
| *Clethrionomys glareolus* (bank vole) | Germany | 303 | 239 | None | Wild | CADT/PCR | [852] |  |
| *Clethrionomys glareolus* (bank vole) | Spain | 1 | 0 | None | Wild | PCR | [740] | ❸ |
| **Tribe Microtini** |  |  |  |  |  |  |  |  |
| *Microtus agrestis* (field vole) | Germany | 65 | 57 | None | Wild | CADT/PCR | [852] |  |
| *Microtus arvalis* (common vole) | Poland | 267 | 257 | 3 groups | Wild | CM | [849] |  |
| *Microtus arvalis* (common vole) | Poland | 7 | 7 | None | Wild | CADT | [9] |  |
| *Microtus arvalis* (common vole) | Poland | 407 | 302 | None | Wild | CM | [850] |  |
| *Microtus arvalis* (common vole) | Germany | 108 | 96 | None | Wild | CADT/PCR | [852] |  |
| *Microtus arvalis* (common vole) | Spain | 79 | 4 | None | Wild | PCR | [740] |  |
| *Microtus cabrerae* (Cabrera’s vole) | Portugal | 49 | 2 | None | Wild | PCR | [741] |  |
| *Microtus californicus* (meadow vole) | USA | 3 | 0 | None | Wild | CADT | [853] | ❸ |
| *Microtus duodecimcostatus* (Mediterranean pine vole) | Spain | 1 | 1 | None | Wild | PCR | [740] | ❸ |
| *Microtus longicaudus* (long-tailed vole) | USA | 3 | 1 | None | Wild | CM | [306] | ❸ |
| *Microtus longicaudus* (long-tailed vole) | USA | 18 | 18 | None | Wild | CM | [314] |  |
| *Microtus lusitanicus* (Lusitanian pine vole) | Portugal | 51 | 36 | None | Wild | PCR | [741] |  |
| *Microtus pennsylvanicus* (Eastern meadow vole) | USA | 6 | 2 | None | Wild | CM | [306] |  |
| *Microtus qazvinensis* (Qazvin vole) | Iran | 10 | 0 | None | Wild | CM | [848] |  |
| *Microtus richardsoni* (North American water vole) | USA | 23 | 23 | None | Wild | CM | [314] |  |
| **Tribe Ondatrini** |  |  |  |  |  |  |  |  |
| *Ondatra zibethicus* (muskrat) | USA | 7 | 7 | None | Wild | CM | [824] |  |
| *Ondatra zibethicus* (muskrat) | USA | 790 | 289 | None | Wild | CM | [825] |  |
| *Ondatra zibethicus* (muskrat) | Canada | 23 | 18 | None | Wild | CADT | [7] |  |
| **Tribe Pliophenacomyini** |  |  |  |  |  |  |  |  |
| *Phenacomys intermedius* (western heather vole) | USA | 4 | 0 | None | Wild | CM | [306] |  |
| **Subfamily Cricetinae** |  |  |  |  |  |  |  |  |
| *Mesocricetus auratus* (golden or Syrian hamster) | China | 11 | 11 | None | Pet | CM/PCR | [854] |  |
| *Mesocricetus auratus* (golden or Syrian hamster) | UAE | 1 | 1 | None | Captive | PCR | [218] | ❸ |
| *Nothocricetulus* *migratorius* (grey dwarf hamster) | Iran | 24 | 3 | None | Wild | CM | [855] |  |
| *Nothocricetulus* *migratorius* (grey dwarf hamster) | Iran | 1 | 0 | None | Wild | CM | [848] | ❸ |
| *Phodopus campbelli* (Campbell’s dwarf hamster) | China | 9 | 9 | None | Pet | CM/PCR | [854] |  |
| *Phodopus sungorus* (winter white dwarf hamster) | China | 87 | 45 | None | Pet | CM/PCR | [854] |  |
| **Subfamily Neotominae** |  |  |  |  |  |  |  |  |
| *Neotoma cinerea* (bushy-tailed woodrat) | USA | 2 | 0 | None | Wild | CM | [306] | ❸ |
| *Neotoma fuscipes* (dusky-footed woodrat) | USA | 6 | 1 | None | Wild | CADT | [853] |  |
| *Peromyscus* *boylii* (brush mouse) | USA | 3 | 0 | None | Wild | CADT | [853] | ❸ |
| *Peromyscus* *californicus* (California parasitic mouse) | USA | 38 | 5 | None | Wild | CADT | [853] |  |
| *Peromyscus maniculatus* (Eastern deermouse) | USA | 50 | 5 | None | Wild | CM | [306] |  |
| *Peromyscus maniculatus* (Eastern deermouse) | USA | 5 | 0 | None | Wild | CM | [314] |  |
| *Peromyscus maniculatus* (Eastern deermouse) | Canada | 2 | 0 | None | Wild | CADT | [7] | ❸ |
| *Peromyscus maniculatus* (Eastern deermouse) | USA | 214 | 54 | None | Wild | CADT | [853] |  |
| *Reithrodontomys megalotis* (Western harvest mouse) | USA | 1 | 0 | None | Wild | CM | [853] | ❸ |
| **Subfamily Sigmodontinae** |  |  |  |  |  |  |  |  |
| *Akodon cursor* (cursor grass mouse) | Brazil | 13 | 1 | None | Wild | PCR | [737] |  |
| *Hylaeamys laticeps* (large-headed rice rat) | Brazil | 81 | 1 | None | Wild | PCR | [737] |  |
| *Necromys lasiurus* (hairy-tailed bolo mouse) | Brazil | 7 | 1 | None | Wild | CM | [659] |  |
| *Nectomys squamipes* (scaly-footed water rat) | Brazil | 4 | 3 | None | Wild | CM | [659] | ❸ |
| *Oecomys catherinae* (Atlantic forest oecomys) | Brazil | 5 | 1 | None | Wild | PCR | [737] |  |
| *Oligoryzomys nigripes (*black-footed pygmy rice rat*)* | Brazil | 7 | 1 | None | Wild | PCR | [737] |  |
| *Rhipidomys mastacalis* (Atlantic forest climbing mouse) | Brazil | 11 | 0 | None | Wild | PCR | [737] |  |
| *Thaptomys nigrita* (blackish grass mouse) | Brazil | 9 | 0 | None | Wild | PCR | [737] |  |
| **Family Dasyproctidae** |  |  |  |  |  |  |  |  |
| *Dasyprocta aguti* (common agouti) | Poland | 1 | 0 | None | Captive | CM | [2] | ❸ |
| *Dasyprocta leporina* (red-rumped agouti) | Brazil | 24 | 1 | None | Farmed | CM | [856] |  |
| **Family dinomyidae** |  |  |  |  |  |  |  |  |
| *Dinomys branickii* (pacarana) | Poland | 2 | 0 | None | Captive | CM | [2] | ❸ |
| **Family Echimyidae** |  |  |  |  |  |  |  |  |
| *Myocastor coypus* (nutria or coypu) | USA | 30 | 20 | None | Wild | CADT | [829] |  |
| *Myocastor coypus* (nutria or coypu) | Japan | 1 | 0 | None | Captive | CADT | [258] | ❸ |
| *Myocastor coypus* (nutria or coypu) | Italy | 153 | 0 | 2 groups | Wild | CADT | [857] |  |
| *Myocastor coypus* (nutria or coypu) | Croatia | 1 | 0 | None | Captive | CADT | [1] | ❸ |
| *Myocastor coypus* (nutria or coypu) | China | 308 | 38 | 3 groups | Farmed | PCR | [858] |  |
| *Myocastor coypus* (nutria or coypu) | China | 5 | 0 | None | Wild | PCR | [317] |  |
| **Family Erethizontidae** |  |  |  |  |  |  |  |  |
| *Erethizon dorsatum* (North American porcupine) | Poland | 8 | 2 | None | Captive | CM | [2] |  |
| *Erethizon dorsatum* (North American porcupine) | Canada | 8 | 0 | None | Wild | CADT | [7] |  |
| **Family Heteromyidae** |  |  |  |  |  |  |  |  |
| *Chaetodipus californicus* (California pocket mouse) | USA | 4 | 1 | None | Wild | CADT | [853] | ❸ |
| *Dipodomys deserti* (desert kangaroo rat) | USA | 3 | 1 | None | Wild | CADT | [853] | ❸ |
| **Family Hystricidae** |  |  |  |  |  |  |  |  |
| *Hystrix brachyura hodgsoni* (Himalayan porcupine) | China | 3 | 0 | None | Captive | PCR | [6] | ❸ |
| *Hystrix cristata* (African crested porcupine) | Italy | 52 | 25 | None | Wild | CADT/PCR | [859] |  |
| *Hystrix cristata* (African crested porcupine) | China | 3 | 0 | None | Captive | PCR | [5] | ❸ |
| **Family Muridae** |  |  |  |  |  |  |  |  |
| **Subfamily Gerbillinae** |  |  |  |  |  |  |  |  |
| *Meriones libycus* (Libyan jird) | Iran | 1 | 0 | None | Wild | CM | [848] | ❸ |
| *Meriones persicus* (Persian jird) | Iran | 25 | 2 | None | Wild | CM | [819] |  |
| *Meriones persicus* (Persian jird) | Iran | 117 | 15 | None | Wild | CM | [855] |  |
| *Meriones persicus* (Persian jird) | Iran | 59 | 2 | None | Wild | CM | [848] |  |
| *Meriones tristrami* (Tristram's jird) | Iran | 17 | 1 | None | Wild | CM | [848] |  |
| **Subfamily Murinae** |  |  |  |  |  |  |  |  |
| **Tribe Apodemini** |  |  |  |  |  |  |  |  |
| *Apodemus agrarius* (striped field mouse) | Poland | 115 | 48 | None | Wild | CM/PCR | [851] |  |
| *Apodemus agrarius* (striped field mouse) | Germany | 35 | 24 | None | Wild | CADT/PCR | [852] |  |
| *Apodemus flavicollis* (yellow-nacked mouse) | Germany | 72 | 30 | None | Wild | CM | [28] |  |
| *Apodemus flavicollis* (yellow-nacked mouse) | Poland | 203 | 98 | 3 groups | Wild | CM | [849] |  |
| *Apodemus flavicollis* (yellow-nacked mouse) | Poland | 9 | 3 | None | Wild | CADT | [9] |  |
| *Apodemus flavicollis* (yellow-nacked mouse) | Poland | 616 | 150 | None | Wild | CM | [850] |  |
| *Apodemus flavicollis* (yellow-nacked mouse) | Poland | 82 | 20 | None | Wild | CM/PCR | [851] |  |
| *Apodemus flavicollis* (yellow-nacked mouse) | Germany | 38 | 13 | None | Wild | CADT/PCR | [852] |  |
| *Apodemus flavicollis* (yellow-nacked mouse) | Spain | 1 | 0 | None | Wild | PCR | [740] | ❸ |
| *Apodemus sylvaticus* (wood mouse) | Germany | 72 | 11 | None | Wild | CM | [28] |  |
| *Apodemus sylvaticus* (wood mouse) | Germany | 9 | 5 | None | Wild | CADT/PCR | [852] |  |
| *Apodemus sylvaticus* (wood mouse) | Spain | 32 | 0 | None | Wild | PCR | [740] |  |
| *Apodemus sylvaticus* (wood mouse) | Portugal | 43 | 2 | None | Wild | PCR | [741] |  |
| *Apodemus witherbyi* (steppe field mouse) | Iran | 10 | 1 | None | Wild | CM | [819] |  |
| *Apodemus* sp. | Iran | 2 | 0 | None | Wild | CM | [848] | ❸ |
| **Tribe Hydromyini** |  |  |  |  |  |  |  |  |
| *Conilurus penicillatus* (brush-tailed rabbit rat) | Australia | 37 | 1 | None | Wild | PCR | [735] |  |
| *Hydromys chrysogaster* (water rat) | Australia | 1 | 0 | None | Wild | CM | [733] | ❸ |
| *Notomys alexis* (spinifex hopping mouse) | Australia | 1 | 0 | None | Wild | CM | [733] | ❸ |
| *Pseudomys albocinereus* (ash-grey mouse) | Australia | 2 | 1 | None | Wild | PCR | [733] | ❸ |
| *Pseudomys nanus* (western chestnut mouse) | Australia | 6 | 0 | None | Wild | CM | [733] |  |
| *Pseudomys occidentalis* (western mouse) | Australia | 2 | 0 | None | Wild | CM | [733] | ❸ |
| *Pseudomys shortridgei* (heath mouse) | Australia | 4 | 0 | None | Wild | CM | [733] | ❸ |
| **Tribe Murini** |  |  |  |  |  |  |  |  |
| *Mus cookii* (Cook's mouse) | Thailand | 1 | 0 | None | Wild | PCR | [860] | ❸ |
| *Mus macedonicus* (Macedonian mouse) | Iran | 23 | 1 | None | Wild | CM | [848] |  |
| *Mus musculus* (house mouse) | New Zealand | 182 | 46 | None | Wild | CM | [739] |  |
| *Mus musculus* (house mouse) | Brazil | 1 | 0 | None | Laboratory | CM | [659] | ❸ |
| *Mus musculus* (house mouse) | New Zealand | 46 | 14 | None | Wild | CM | [652] |  |
| *Mus musculus* (house mouse) | Brazil | 763 | 144 | None | Laboratory | CM | [861] |  |
| *Mus musculus* (house mouse) | South Korea | 670 | 64 | None | Laboratory | CM | [862] |  |
| *Mus musculus* (house mouse) | Brazil | 344 | 18 | None | Laboratory | CM | [863] |  |
| *Mus musculus* (house mouse) | Iraq | 10 | 1 | None | Wild | CM | [864] |  |
| *Mus musculus* (house mouse) | USA | 3 | 2 | None | Wild | CADT | [853] | ❸ |
| *Mus musculus* (house mouse) | Spain | 165 | 29 | None | Wild | CM/PCR | [865] |  |
| *Mus musculus* (house mouse) | China | 31 | 1 | None | Wild | CM/PCR | [866] |  |
| *Mus musculus* (house mouse) | Iran | 35 | 31 | None | Laboratory | CM | [867] |  |
| *Mus musculus* (house mouse) | Iran | 61 | 1 | None | Wild | CM/PCR | [855] |  |
| *Mus musculus* (house mouse) | Argentina | 1,722 | 55 | None | Laboratory | CM | [868] |  |
| *Mus musculus* (house mouse) | Iran | 40 | 1 | None | Wild | CM/PCR | [869] |  |
| *Mus musculus* (house mouse) | China | 1,027 | 0 | None | Laboratory | PCR | [870] |  |
| *Mus musculus* (house mouse) | Spain | 4 | 0 | None | Wild | PCR | [740] | ❸ |
| *Mus pahari* (Gairdner's shrewmouse) | Thailand | 1 | 0 | None | Wild | PCR | [860] | ❸ |
| *Mus spretus* (western Mediterranean mouse) | Spain | 8 | 0 | None | Wild | PCR | [740] |  |
| *Mus spretus* (western Mediterranean mouse) | Portugal | 48 | 4 | None | Wild | PCR | [741] |  |
| **Tribe Rattini** |  |  |  |  |  |  |  |  |
| *Bandicota indica* (greater bandicoot rat) | Thailand | 46 | 16 | None | Wild | PCR | [860] |  |
| *Berylmys berdmorei* (small white-toothed rat) | Thailand | 1 | 0 | None | Wild | PCR | [860] | ❸ |
| *Berylmys bowersi* (Bower's white-toothed rat) | Thailand | 6 | 0 | None | Wild | PCR | [860] |  |
| *Leopoldamys edwardsi* (Edwards's long-tailed giant rat) | Thailand | 2 | 0 | None | Wild | PCR | [860] | ❸ |
| *Leopoldamys sabanus* (long-tailed giant rat) | Thailand | 2 | 0 | None | Wild | PCR | [860] | ❸ |
| *Maxomys surifer* (red spiny rat) | Thailand | 14 | 5 | None | Wild | PCR | [860] |  |
| *Niviventer fulvescens* (chestnut white-bellied rat) | Thailand | 1 | 0 | None | Wild | PCR | [860] | ❸ |
| *Rattus andamanensis* (Sikkim rat) | Thailand | 2 | 0 | None | Wild | PCR | [860] | ❸ |
| *Rattus argentiventer* (ricefield rat) | Thailand | 3 | 1 | None | Wild | PCR | [860] | ❸ |
| *Rattus exulans* (Polynesian rat) | Thailand | 11 | 1 | None | Wild | PCR | [860] |  |
| *Rattus fuscipes* (bush rat) | Australia | 12 | 1 | None | Wild | CM/PCR | [733] |  |
| *Rattus norvegicus* (brown rat) | Brazil | 304 | 48 | None | Laboratory | CM | [861] |  |
| *Rattus norvegicus* (brown rat) | South Korea | 265 | 0 | None | Laboratory | CM | [862] |  |
| *Rattus norvegicus* (brown rat) | Brazil | 111 | 0 | None | Laboratory | CM | [863] |  |
| *Rattus norvegicus* (brown rat) | Iraq | 25 | 3 | None | Laboratory | CM | [864] |  |
| *Rattus norvegicus* (brown rat) | Spain | 3 | 2 | None | Wild | CM | [865] | ❸ |
| *Rattus norvegicus* (brown rat) | China | 168 | 11 | None | Wild | CM | [866] |  |
| *Rattus norvegicus* (brown rat) | Iran | 15 | 7 | None | Wild | CM | [819] |  |
| *Rattus norvegicus* (brown rat) | Thailand | 1 | 0 | None | Wild | PCR | [860] | ❸ |
| *Rattus norvegicus* (brown rat) | Iran | 35 | 26 | None | Laboratory | CM | [867] |  |
| *Rattus norvegicus* (brown rat) | Grenada | 99 | 17 | None | Wild | CADT | [871] |  |
| *Rattus norvegicus* (brown rat) | China | 23 | 4 | None | Laboratory | CM/PCR | [854] |  |
| *Rattus norvegicus* (brown rat) | Argentina | 1,030 | 61 | None | Laboratory | CM | [868] |  |
| *Rattus norvegicus* (brown rat) | Peru | 79 | 6 | 2 groups | Captive | CM | [872] |  |
| *Rattus norvegicus* (brown rat) | Argentina | 118 | 24 | None | Wild | CM | [873] |  |
| *Rattus norvegicus* (brown rat) | Iran | 100 | 76 | None | Wild | PCR | [874] |  |
| *Rattus norvegicus* (brown rat) | China | 355 | 33 | None | Laboratory | PCR | [875] |  |
| *Rattus norvegicus* (brown rat) | Spain | 100 | 35 | None | Wild | PCR | [876] |  |
| *Rattus norvegicus* (brown rat) | Spain | 64 | 9 | None | Wild | PCR | [794] |  |
| *Rattus norvegicus* (brown rat) | China | 191 | 9 | None | Wild | PCR | [225] |  |
| *Rattus norvegicus* (brown rat) | China | 118 | 0 | None | Laboratory | PCR | [870] |  |
| *Rattus norvegicus* (brown rat) | Iran | 40 | 1 | None | Wild | CM/PCR | [869] |  |
| *Rattus rattus* (black rat) | New Zealand | 77 | 47 | None | Wild | CM | [739] |  |
| *Rattus rattus* (black rat) | Brazil | 168 | 57 | None | Laboratory | CM | [659] |  |
| *Rattus rattus* (black rat) | New Zealand | 19 | 8 | None | Wild | CM | [652] |  |
| *Rattus rattus* (black rat) | Spain | 116 | 42 | None | Wild | CM/PCR | [865] |  |
| *Rattus rattus* (black rat) | Australia | 12 | 0 | None | Wild | PCR | [328] |  |
| *Rattus rattus* (black rat) | Peru | 48 | 1 | 2 groups | Captive | CM | [872] |  |
| *Rattus rattus* (black rat) | Malaysia | 134 | 4 | None | Wild | PCR | [877] |  |
| *Rattus rattus* (black rat) | Madagascar | 40 | 21 | None | Wild | CADT | [240] |  |
| *Rattus rattus* (black rat) | Austria | 50 | 17 | 2 groups | Wild | PCR | [878] |  |
| *Rattus rattus* (black rat) | Spain | 21 | 8 | None | Wild | PCR | [740] |  |
| *Rattus rattus* (black rat) | Iran | 40 | 2 | None | Wild | CM/PCR | [869] |  |
| *Rattus tanezumi* (Asian house rat) | China | 33 | 2 | None | Wild | CM/PCR | [866] |  |
| *Rattus tanezumi* (Asian house rat) | Thailand | 188 | 37 | None | Wild | PCR | [860] |  |
| *Sundamys muelleri* (Müller's giant Sunda rat) | Thailand | 1 | 0 | None | Wild | PCR | [860] | ❸ |
| **Family Octodontidae** |  |  |  |  |  |  |  |  |
| *Octodon degus* (common degu or Western Chile) | Germany | 3 | 1 | None | Pet | CADT | [653] | ❸ |
| **Family Pedetidae** |  |  |  |  |  |  |  |  |
| *Pedetes capensis* (South African springhare) | Croatia | 2 | 0 | None | Captive | CADT | [1] | ❸ |
| **Family Sciuridae** |  |  |  |  |  |  |  |  |
| *Callosciurus notatus* (tricoloured squirrel) | Poland | 1 | 0 | None | Captive | CM | [2] | ❸ |
| *Callosciurus prevostii* (Asian tricolored squirrel) | Croatia | 1 | 1 | None | Captive | CADT/PCR | [1] | ❸ |
| *Cynomys ludovicianus* (black-tailed prairie dog) | Thailand | 79 | 11 | 2 groups | Pet | CM | [879] |  |
| *Eutamias asiaticus* (chipmunk) | China | 279 | 24 | None | Pet | PCR | [880] |  |
| *Funambulus palmarum* (Indian palm squirrel) | UAE | 5 | 5 | None | Captive | PCR | [218] |  |
| *Funambulus tristriatus* (jungle palm squirrel) | Poland | 1 | 0 | None | Captive | CM | [2] | ❸ |
| *Funisciurus pyrrhopus* (fire-footed rope squirrel) | Poland | 2 | 0 | None | Captive | CM | [2] | ❸ |
| *Marmota himalayana* (Himalayan marmot) | China | 399 | 6 | None | Wild | PCR | [881] |  |
| *Menetes berdmorei* (Berdmore's ground squirrel) | Thailand | 2 | 0 | None | Wild | PCR | [860] | ❸ |
| *Neotamias amoenus* (yellow-pine chipmunk) | USA | 8 | 0 | None | Wild | CM | [306] |  |
| *Otospermophilus beecheyi* (California ground squirrel) | USA | 2 | 1 | None | Wild | CADT | [853] | ❸ |
| *Petaurista leucogenys* (Japanese giant flying squirrel) | Japan | 1 | 0 | None | Captive | CADT | [258] | ❸ |
| *Rhinosciurus laticaudatus* (shrew-faced squirrel) | UAE | 1 | 0 | None | Captive | PCR | [218] | ❸ |
| *Sciurus carolinensis* (eastern gray squirrel) | Canada | 15 | 0 | None | Wild | CADT | [7] |  |
| *Sciurus carolinensis* (eastern gray squirrel) | Japan | 3 | 0 | None | Captive | CADT | [258] | ❸ |
| *Sciurus carolinensis* (eastern gray squirrel) | UAE | 1 | 0 | None | Captive | PCR | [218] | ❸ |
| *Sciurus vulgaris* (red squirrel) | Spain | 1 | 0 | None | Wild | PCR | [740] | ❸ |
| *Spermophilus alashanicus* (Alashan ground squirrel) | China | 99 | 2 | None | Wild | PCR | [881] |  |
| **Family Spalacidae** |  |  |  |  |  |  |  |  |
| *Eospalax fontanierii* (Chinese zokor) | China | 8 | 2 | None | Wild | CADT | [11] |  |
| *Rhizomys sinensis* (Chinese bamboo rat) | China | 480 | 52 | 4 groups | Farmed | PCR | [882] |  |
| **Family Zapodidae** |  |  |  |  |  |  |  |  |
| *Zapus* *princeps* (Southwestern jumping mouse) | USA | 4 | 0 | None | Wild | CM | [314] | ❸ |

E: Excluded datasets, in the selection process phase, where the pre-selected studies were evaluated through full text review to confirm whether they met the eligibility criteria

❶ Studies with fecal samples (especially related to stray or unwanted dogs and cats) which were collected from the ground (e.g. street or public park) and data from each animal was not independently retrievable.

❷ Follow up studies that could not report a correct estimate of prevalence.

❸ Studies with sample size < 20 for domestic animals and < 5 for captive and wild animals.

❹ Studies focused only on microscopically positive isolates, without primary sample size and a clear final result of prevalence.

❺ Studies associated with pooled faecal samples.

Ca: Cattery; BS: Breeding establishment; CADT: Copro-antigen detection techniques; Cl: Veterinary clinic; CM: Conventional microscopy; FD: Farm dog; Ho: Household; Hu: Hunting dog; Ke: Kennel; PCR: Polymerase chain reaction; Pe: Pet; Po: Police dog; PS: Pet shop; R: Rural area; Re: Refuge; Sh: Shelter; Sl: Sled dog; St: Stray; U: Urban area.

**REFERENCES**

1. Beck R, Sprong H, Bata I Lucinger S, Pozio E, Caccio SM (2011) Prevalence and molecular typing of *Giardia* spp. in captive mammals at the zoo of Zagreb, Croatia. *Veterinary Parasitology* 175: 40-46.
2. Peisert W, Taborski A, Pawlowski Z, Karlewiczowa R, Zdun M (1983) *Giardia* infection in animals in Poznan Zoo. *Veterinary Parasitology* 13: 183-186.
3. Karim MR, Li J, Rume FI, Sumon SMR, Selim ASM, Hoda N, Zhang L (2021) Occurrence and molecular characterization of *Cryptosporidium* spp. and *Giardia duodenalis* among captive mammals in the Bangladesh National Zoo. *Parasitology International* 84: 102414.
4. Geurden T, Goossens E, Levecke B, Vercammen F, Vercruysse J, Claerebout E (2009) Occurrence and molecular characterization of *Cryptosporidium* and *Giardia* in captive wild ruminants in Belgium. *Journal of Zoo and Wildlife Medicine* 40: 126-130.
5. Zhang K, Zheng S, Wang Y, Wang K, Wang Y, Gazizova A, Han K, Yu F, Chen Y, Zhang L (2021) Occurrence and molecular characterization of *Cryptosporidium* spp., *Giardia duodenalis*, *Enterocytozoon bieneusi*, and *Blastocystis* sp. in captive wild animals in zoos in Henan, China. *BMC* *Veterinary Research* 17: 332.
6. Zou Y, Li XD, Meng YM, Wang XL, Wang HN, Zhu XQ (2022) Prevalence and multilocus genotyping of *Giardia duodenalis* in zoo animals in three cities in China. *Parasitology Research* 121: 2359-2366.
7. Heitman TL, Frederick LM, Viste JR, Guselle NJ, Morgan UM, Thompson RCA, Olson ME (2002) Prevalence of *Giardia* and *Cryptosporidium* and characterization of *Cryptosporidium* spp. isolated from wildlife, human, and agricultural sources in the North Saskatchewan River Basin in Alberta, Canada. *Canadian Journal of Microbiology* 48: 530-541.
8. Ristanic M, Vuckovic J, Dominikovic N, Vucicevic M, Rajkovic M, Bogunovic D, Ozvegy J (2023) Use of parasitological and molecular methods in *Giardia* sp. detection in animals held in capacity. *Acta Veterinaria* 73: 195-204.
9. Bednarska M, Bajer A, Sinski E, Girouard AS, Tamang L, Graczyk TK (2007) Fluorescent in situ hybridization as a tool to retrospectively identify *Cryptosporidium parvum* and *Giardia lamblia* in samples from terrestrial mammalian wildlife. *Parasitology Research* 100: 455-460.
10. Paziewska A, Bednarska M, Niewęgłowski H, Karbowiak G, Bajer A (2007) Distribution of *Cryptosporidium* and *Giardia* spp. in selected species of protected and game mammals from North-Eastern Poland. *Annals of Agricultural Environment Medicine* 14: 265-270.
11. Ma L, Sotiriadou I, Cai Q, Karanis G, Wang G, Wang G, Lu Y, Li X, Karanis P (2014) Detection of *Cryptosporidium* and *Giardia* in agricultural and water environments in the Qinghai area of China by IFT and PCR. *Parasitology Research* 113:3177-3184.
12. Qi M, Cai J, Wang R, Li J, Jian F, Huang J, Zhou H, Zhang L (2015) Molecular characterization of *Cryptosporidium* spp. and *Giardia duodenalis* from yaks in the central western region of China. *BMC Microbiology* 15: 108
13. Song GY, Qin SY, Zhao GH, Zhu XQ, Zhou DH, Song MX (2016) Molecular characterization of *Giardia duodenalis* from white yaks in China. *Acta* *Parasitologica* 61: 397-400.
14. Jin Y, Fei J, Cai J, Wang X, Li N, Guo Y, Feng Y, Xiao L (2017) Multilocus genotyping of *Giardia duodenalis* in Tibetan sheep and yaks in Qinghai, China. *Parasitology Research* 247: 70-76.
15. Liu H, Shen Y, Liu A, Yin J, Yuan Z, Jiang Y, Pan W, Zhang Y, Zhao W, Cao J (2017) Occurrence and multilocus genotyping of *Giardia duodenalis* in pets and zoo animals in Shanghai, China. *Journal of Infection in Developing Countries* 11: 479-486.
16. Wang G, Wang G, Li XP, Ma LQ, Karanis G, Eleni CV, Karanis P (2017) Detection of *Giardia duodenalis* assemblage E infections at the Tibetan Plateau Area: Yaks are suitable hosts. *Acta Tropica* 169: 157-62.
17. Wang G, Wang G, Li X, Zhang X, Karanis G, Jian Y, Ma, Karanis P (2018) Prevalence and molecular characterization of *Cryptosporidium* spp. and *Giardia duodenalis* in 1-2-month-old d yaks in Qinghai Province, China. *Parasitology Research* 117: 1793-800.
18. Wu Y, Chang Y, Zhang X, Chen Y, Li D, Wang L, Zheng S, Wang R, Zhang S, Jian F, Ning C, Li J, Zhang L (2019) Molecular characterization and distribution of *Cryptosporidium* spp., *Giardia duodenalis*, and *Enterocytozoon bieneusi* from yaks in Tibet, China. *BMC Veterinary Research* 15: 417.
19. Zhang Q, Zgang Z, Ai S, Wang X, Zhang R, Duan Z (2019) *Cryptosporidium* spp., *Enterocytozoon bieneusi*, and *Giardia duodenalis* from animal sources in the Qinghai-Tibetan Plateau Area (QTPA) in China. *Comparative Immunology, Microbiology and Infectious Disease* 67: 101346.
20. Wu Y, Chen Y, Chang Y, Zhang X, Li D, Wang L, Zheng S, Wang R, Zhang S, Li J, Zhang L (2020) Genotyping and identification of *Cryptosporidium* spp., *Giardia duodenalis* and *Enterocytozoon bieneusi* from free-range Tibetan yellow cattle and cattle-yak in Tibet, China. *Acta Tropica* 212: 105671.
21. Song JK, Wang D, Ren M, Yang F, Wang PX, Zou M, Zhao GH, Lin Q (2021) Seasonal prevalence and novel multilocus genotypes of *Giardia duodenalis* in yaks (*Bos grunniens*) in Qinghai province, western China. *Iran Journal of Parasitology* 16: 548-554.
22. Chen X, Saeed NM, Ding J, Dong H, Kulyar MFEA, Bhutta ZA, Mehmood K, Ali MM, Irshad I, Zeng J, Liu J, Wu Q, Li K (2022) Molecular Epidemiology Investigation of *Cryptosporidium* sp., *Giardia duodenalis*, *Enterocytozoon bieneusi* and *Blastocystis* sp. Infection in free-ranged yaks and tibetan pigs on the Plateau. *Pakistan Veterinary Journal* 42: 533-539.
23. Deshpande PD, Shastri UV (1981) Incidence of *Giardia* infection in calves in Maharashtra state, India. *Tropical Animal Health Production* 13: 34.
24. Taminelli V, Eckert J (1989) Prevalence and geographical distribution of *Giardia* infections of ruminants in Switzerland. *Schweizer Archiv fur Tierheilkunde* 131: 251-258.
25. Buret A, denHollander N, Wallis PM, Befus D, Olson ME (1990) Zoonotic potential of giardiasis in domestic ruminants. *Journal of Infectious Diseases* 162: 231-237.
26. Xiao L, Herd RP, Rings DM (1993) Concurrent infections of *Giardia* and *Cryptosporidium* on two Ohio farms with calf diarrhea. *Veterinary* *Parasitology* 51: 41-48.
27. Quilez J, Sanchez-Acedo C, del Cacho E, Clavel A, Causape AC (1996) Prevalence of *Cryptosporidium* and *Giardia* infections in cattle in Aragon (northeastern Spain). *Veterinary Parasitology* 66: 139-46.
28. Karanis P, Opiela K, Al-Arousi M, Seitz HM (1996) A comparison of phase contrast microscopy and an immunofluorescence test for the detection of *Giardia* spp. in faecal specimens from cattle and wild rodents. *Transactions of the Royal Society of Tropical Medicine and Hygiene* 90: 250-251.
29. Diaz V, Campos M, Lozano J, Manas I, Gonzalez J (1996) Aspects of animal giardiasis in Granada province (southern Spain). *Veterinary* *Parasitology* 64: 171-176.
30. Rigolon LP, Vargas L (1996) Prevalence of *Giardia* in dairy cattle in in northwest of Parana-Brazil. *Revista UNIMAR* 18: 617-626.
31. Iburg T, Gasser RB, Henriksen SA (1996) First record of *Giardia* in cattle in Denmark. *Acta Veterinaria Scandinavica* 37: 337-341.
32. Olson ME, Thorlakson CL, Deselliers L, Morck DW, McAllister TA (1997) *Giardia* and *Cryptosporidium* in Canadian farm animals. *Veterinary* *Parasitology* 68: 375-381.
33. Olson ME, Guselle NJ, O'Handley RM, Swift ML, McAllister TA, Jelinski MD, Morck DW (1997) *Giardia* and *Cryptosporidium* in dairy calves in British Columbia. *Canadian Veterinary Journal* 38: 703-706.
34. Himonas CA, Antoniadou-Soteriodou KS, Sotiraki ST, Papazahariadou MG (1998) Intestinal protozoa of animals in the Macedonia region of Greece. *Journal of the Hellenic Veterinary Medical Society* 49: 300-306.
35. Bednarska M, Bajer A, Siński E (1998) [Calves as a potential reservoir of *Cryptosporidium parvum* and *Giardia* sp.](https://pubmed.ncbi.nlm.nih.gov/9860815/)  *Annals of Agricultural Environmental Medicine* 5: 135-8.
36. Ruest N, Faubert GM, Couture Y (1998) Prevalence and geographical distribution of *Giardia* spp. And *Cryptosporidium* spp. In dairy farms in Quebec. *Canadian Veterinary Journal* 39: 697-700.
37. Hoar BR, Atwill ER, Elmi C, Utterback WW, Edmondson AJ (1999) Comparison of fecal samples collected per rectum and off the ground for estimation of environmental contamination attributable to beef cattle. *American Journal of Veterinary Research* 60: 1352-1356.
38. Hsu BM, Huang C, Hsu YF, Hsu CLL., Hsu YF., Yeh JH (1999) Occurrence of *Giardia* and *Cryptosporidium* in the Kau-Ping river and its watershed in southern Taiwan. *Water Sciences Technology* 33: 2701-2707.
39. Vilchez QS, Pote L (1999) Concurrent infections of *Cryptosporidium* and *Giardia* in dairy farms, Mississippi state, USA. *Revista Cientifica*, *FCV-LUZ* 9: 519-523.
40. O’Handley RM, Cockwill C, McAllister TA, Jelinski M, Morck DW, Olson ME (1999) Duration of naturally acquired giardiasis and cryptosporidiosis in dairy calves and their association with diarrhea. *Journal of Americam Veterinary Association* 214: 391-396.
41. Wade SE, Mohammed HO, Schaaf SL (2000) Prevalence of *Giardia* sp., *Cryptosporidium parvum* and *Cryptosporidium muris* (*C. andersoni*) in 109 dairy herds in five counties of southeastern New York. *Veterinary Parasitology* 93: 1-11.
42. Fayer R, Trout JM, Graczyk TK, Lewis EJ (2000) Prevalence of *Cryptosporidium*, *Giardia* and *Eimeria* infections in post-weaned and adult cattle on three Maryland farms. *Veterinary Parasitology* 93: 103-112.
43. Hunt CL, Ionas G, Brown TJ (2000) Prevalence and strain differentiation of *Giardia intestinalis* in calves in the Manawatu and Waikato regions of North Island, New Zealand. *Veterinary Parasitology* 91: 7-13.
44. O’Handley RM, Olson ME, Fraser D, Adams P, Thompson RCA (2000) Prevalence and genotypic characterisation of *Giardia* in dairy calves from Western Australia and Western Canada. *Veterinary Parasitology* 90: 193-200.
45. Nizeyi J, Cranfield M, Graczyk T (2002) Cattle near the Bwindi Impenetrable National Park, Uganda, as a reservoir of *Cryptosporidium parvum* and *Giardia duodenalis* for local community and free-ranging gorillas. *Parasitology Research* 88: 380-385.
46. Graczyk TK, Bosco-Nizeyi J, Ssebide B, Thompson RC, Read C, Cranfield MR (2002) Anthropozoonotic *Giardia* *duodenalis* genotype (assemblage) a infections in habitats of free-ranging human-habituated gorillas, Uganda. *Journal of Parasitology* 88: 905-909.
47. Hoet AE, Nielsen PR, Hasoksuz M, Thomas C, Wittum TE, Saif LJ (2003) Detection of bovine torovirus and other enteric pathogens in feces from diarrhea cases in cattle. Journal of Veterinary Diagnostic Investigation 15: 205-212.
48. Björkman C, Svensson C, Christensson B, de Verdier K (2003) *Cryptosporidium parvum* and *Giardia intestinalis* in calf diarrhoea in Sweden. *Acta Veterinaria Scandinavica* 44: 145-52.
49. Barwick RS, Mohammed HO, White ME, Bryant RB (2003) Prevalence of *Giardia* spp. and *Cryptosporidium* spp. on dairy farms in southeastern New York state. *Preventive Veterinary Medicine* 59: 1-11
50. Learmonth JJ, Ionas G, Pita AB, Cowie RS (2003) Identification and genetic characterisation of *Giardia* and *Cryptosporidium* strains in humans and dairy cattle in the Waikato Region of New Zealand. *Water Science and Technology* 47: 21-26.
51. Appelbee AJ., Frederick LM., Heitman TL., Olson ME (2003) Prevalence and genotyping of *Giardia duodenalis* from beef calves in Alberta, Canada. *Veterinary Parasitology* 112: 289-294.
52. Epe C, Coati N, Schnieder T (2004) Results of parasitological examinations of faecal samples from horses, ruminants, pigs, dogs, cats, hedgehogs and rabbits between 1998 and 2002. *Deutsche Tierarztliche Wochenschrift* 111: 243-247.
53. Smith KE, Stenzel SA, Bender JB, Wagstrom E, Soderlund D, Leano FT, Taylor CM, Belle-Isle PA, Danila R (2004) Outbreaks of enteric infections caused by multiple pathogens associated with calves at a farm day camp. *The Pediatric Infectious Disease Journal* 23: 1098-1104
54. Geurden T, Claerebout E, Vercruysse J, Berkvens D (2004) Estimation of diagnostic test characteristics and prevalence of *Giardia* *duodenalis* in dairy calves in Belgium using a Bayesian approach. *International Journal of Parasitology* 34: 1121-1127.
55. Trout JM, Santin M, Ellis Greiner E, Fayer R (2004) Prevalence of *Giardia duodenalis* genotypes in pre-weaned dairy calves. *Veterinary Parasitology* 124: 179-186.
56. Berrilli F, Di Cave D, De Liberato C, Franco A, Scaramozzino P, Orecchia P (2004) Genotype characterisation of *Giardia duodenalis* isolates from domestic and farm animals by SSU-rRNA gene sequencing. *Veterinary Parasitology* 122: 193-199.
57. McAllister TA, Olson ME, Fletch, A, Wetzstein M, Entz T (2005) Prevalence of *Giardia* and *Cryptosporidium* in beef cows in southern Ontario and in beef calves in southern British Columbia. *Canadian Veterinary Journal* 46: 47-55.
58. Degerly S, Celiksoz A, Kalkan K, Ozcelik S (2005) Prevalence of *Cryptosporidium* spp. and *Giardia* spp. in Cows and Calves in Sivas. *Turkish Journal of Veterinary and Animal Sciences* 29: 995-999.
59. Trout JM, Santin M, Ellis Greiner E, Fayer R (2005) Prevalence and genotypes of *Giardia duodenalis* in post-weaned dairy calves. *Veterinary Parasitology* 130: 177-183.
60. Kusiluka LJM, Karimuribo ED, Mdegela RH, Luoga EJ, Munishi PKT, Mlozi MRS, Kambarage DM (2005) Prevalence and impact of water-borne zoonotic pathogens in water, cattle and humans in selected villages in Dodoma rural and Bagamoyo districts, Tanzania. *Physics and Chemistry of the Earth* 30: 818-825.
61. Goz Y, Altug N, Yuksek N, Ozkan C (2006) Parasites detected in neonatal and young calves with diarrhoea. *Bulletin of the Veterinary Institue in Pulawy* 50: 345-348.
62. Hamnes IS, Gjerde B, Robertson L (2006) Prevalence of *Giardia* and *Cryptosporidium* in dairy calves in three areas of Norway. *Veterinary* *Parasitology* 140: 204-216.
63. Trout JM, Santin M, Ellis Greiner E, Fayer R (2006) Prevalence and genotypes of *Giardia duodenalis* in 1-2 year old dairy cattle. *Veterinary Parasitology* 140: 217-22.
64. Maddox-Hyttel C, Langkjaer RB, Enemark HL, Vigre H (2006) *Cryptosporidium* and *Giardia* in different groups of Danish cattle and pigs - occurrence and management associated risk factors. *Veterinary Parasitology* 141: 48-59.
65. Uehlinger FD, Barkema HW, Dixon BR, Coklin T, O'Handley RM (2006) [*Giardia duodenalis* and *Cryptosporidium* spp. in a veterinary college bovine teaching herd.](https://pubmed.ncbi.nlm.nih.gov/16905259/)  *Veterinary Parasitology* 142: 231-237.
66. Gow S, Waldner C (2006) An examination of the prevalence of and risk factors for shedding of *Cryptosporidium* spp. and *Giardia* spp. in cows and calves from western Canadian cow-calf herds. *Veterinary Parasitology* 137: 50-61.
67. Haschek B, Klein D, Benetka V, Herrera C, Sommerfeld-Stur I, Vilcek S, Moestl K, Baumgartner W (2006) Detection of Bovine Torovirus in neonatal calf diarrhoea in lower Austria and Styria (Austria). *Journal of Veterinary Medicine B Infectious Diseases and Veterinary Public Health* 53: 160-165.
68. Rhaymah MSH, Mohammed BA (2006) Preliminary study on the prevalence of *Giardia* in ruminants in ninevah province. *Iraqi Journal of Veterinary Sciences* 20: 153-163.
69. Castro-Hermida JA, Carro-Corral C, González-Warleta M, Mezo M (2006) [Prevalence and intensity of infection of *Cryptosporidium* spp. and *Giardia duodenalis* in dairy cattle in Galicia (NW Spain).](https://pubmed.ncbi.nlm.nih.gov/16732884/)  *Journal of Veterinary Medicine B Infectious Diseases and Veterinary Public Health* 53: 244-246.
70. Trout JM, Santin M., Fayer R (2007) Prevalence of *Giardia duodenalis* genotypes in adult dairy cows. *Veterinary Parasitology* 147: 205-209.
71. Mendonca C, Almeida A, Castro A, Delgado ML, Soares S, Correia da Costa JM, Canada N (2007) Molecular characterization of *Cryptosporidium* and *Giardia* isolates from cattle from Portugal. *Veterinary Parasitology* 147: 47-50.
72. Castro-Hermida JA, Almeida A, González-Warleta M, Correia da Costa JM, Rumbo-Lorenzo C, Mezo M (2007) Occurrence of *Cryptosporidium parvum* and *Giardia duodenalis* in healthy adult domestic ruminants. *Parasitology Research* 101: 1443-1448.
73. Hsu BM, Wun HY, Hsu PC (2007) Prevalence and genotyping of *Giardia* in husbandry systems in Taiwan. *Parasitology Research* 101: 275-280.
74. Coklin T, Farber J, Parrington L, Dixon B (2007) Prevalence and molecular characterization of *Giardia duodenalis* and *Cryptosporidium* spp. in dairy cattle in Ontario, Canada. *Veterinary Parasitology* 150: 297-305
75. Geurden T, Geldhof P, Levecke B, Martens C, Berkvens D, Casaert S, Vercruysse J, Claerebout E (2008) Mixed *Giardia duodenalis* assemblage A and E infections in calves. *International Journal for Parasitology* 38: 259-264.
76. Winkworth CL, Matthaei CD, Townsend CR (2008) Prevalence of *Giardia* and *Cryptosporidium* spp. in calves from a region in New Zealand experiencing intensification of dairying. *New Zealand Veterinary Journal* 56: 15-20.
77. Gül A, Ciçek M, Kilinç O (2008) Prevalence of *Eimeria* spp., *Cryptosporidium* spp. And *Giardia* spp. in calves in the Van province. *Turkiye* *Parazitoloji* *Dergisi* 32: 202-204
78. Moriarty EM, Sinton LW, Mackenzie ML, Karki N, Wood DR (2008) A survey of enteric bacteria and protozoans in fresh bovine faeces on New Zealand dairy farms. *Journal of Applied Microbiology* 105: 2015-2025.
79. Uehlinger FD, Barkema HW, O’Handley RM, Parenteau M, Parrington LJ, Van Leeuwen JA, Dixon BR (2008) Comparison of flow cytometry and immunofluorescence microscopy for the detection of *Giardia duodenalis* in bovine fecal samples. Journal of Veterinary Diagnostic Investigation 20: 178-185.
80. Barigye R, Dyer NW, Newell TK, Khaitsa ML, Trout JM, Santin M, Fayer R (2008) Molecular and immunohistochemical detection of assemblage E, *Giardia duodenalis* in scouring North Dakota calves. *Veterinary Parasitology* 157: 196-202.
81. Geurden T, Somers R, Thanh NT, Vien LV, Nga VT, Giang HH, Dorny P, Giao HK, Vercruysse J (2008) Parasitic infections in dairy cattle around Hanoi, northern Vietnam. *Veterinary Parasitology* 153: 384-8.
82. Singh BB, Sharma R, Sharma JK, Banga HS, Kumar H, Aulakh RS, Gill JPS (2008) Prevalence of *Giardia intestinalis* infection in cattle. Journal of *Veterinary Parasitology* 22: 91-92.
83. Santin M, Trout JM, Fayer R (2009) A longitudinal study of *Giardia duodenalis* genotypes in dairy cows from birth to 2 years of age. *Veterinary Parasitology* 162: 40-45.
84. Sabry MA, Taher ES, Meabed EMH (2009) Prevalence and genotyping of zoonotic *Giardia* from Fayoum Governorate, Egypt. *Research Journal of Parasitology* 4: 105-114.
85. Hoar BR, Paul RR, Siembieda J. Pereira MdGC, Atwill ER (2009) *Giardia duodenalis* in feedlot cattle from the central and western United States. *BMC Veterinary Research* 5: 37.
86. Castro-Hermida JA, Garcia-Presedo I, Almeida A, Gonzalez-Warleta M, Da Costa JMC, Mezo M (2009) Detection of *Cryptosporidium* spp. and *Giardia duodenalis* in surface water: A health risk for humans and animals. *Water Research* 43: 4133-4142.
87. Borchard P, Wright IA, Eldridge DJ (2010) Wombats and domestic livestock as potential vectors of *Cryptosporidium* and *Giardia* in an agricultural riparian area. *Australian Journal of Zoology* 58: 150-153.
88. Ayaz S, Ullah M, Khan FU, Bibi A, Noreen S, Rahim H, Akhtar M (2010) Epidemiological studies and molecular diagnosis of giardiasis in bovine. *Pakistan Journal of Life Social Sciences* 8: 148-155.
89. Geurden T, Levecke B, Pohle H, De Wilde N, Vercruysse J, Claerebout E (2010) A Bayesian evaluation of two dip-stick assays for the on-site diagnosis of infection in calves suspected of clinical giardiasis. *Veterinary Parasitology* 172: 337-340.
90. Ghadrdan-Mashhadi A, Bokaie S, Rezae A (2010) A sturvey on giardiasis in calves in some dairy cattle farms around Tehran. *Veterinary Research Bulletin* 6: 57-61. (in Persia)
91. Kanyari PWN, Kagira JM, Mhoma JRL (2010) Prevalence of endoparasites in cattle within urban and peri-urban areas of Lake Victoria Basin, Kenya with special reference to zoonotic potential. Science Parasitology 11: 171-178.
92. Johnston AR, Gillespie TR, Rwego IB, Tranby McLachlan TL, Kent AD, Goldberg TL (2010) Molecular epidemiology of Cross-species *Giardia duodenalis* transmission in Western Uganda. *PLoS Neglected Tropical Diseases* 4: e638.
93. Ng J, Yang R, Whiffin V, Cox P, Ryan U (2011) Identification of zoonotic *Cryptosporidium* and *Giardia* genotypes infecting animals in Sydney’s water catchments. *Experimental Parasitology* 128: 138-144.
94. Ng J, Yang R, McCarthy S, Gordon C, Hijjawi N, Ryan U (2011) Molecular characterization of *Cryptosporidium* and *Giardia* in pre-weaned calves in Western Australia and New South Wales. *Veterinary Parasitology* 176: 145-150.
95. Uehlinger FD, Greenwood SJ, O'Handley R, McClure JT, Coklin T, Dixon BR, de Boer M, Zwiers H, Barkema HW (2011) Prevalence and genotypes of *Giardia duodenalis* in dairy and beef cattle in farms around Charlottetown, Prince Edward Island, Canada. *Canadian Veterinary Journal* 52: 967-72.
96. Otero-Negrete JJ, Ibarra-Velarde F, Martínez-Gordillo MN, Ponce-Macotela M (2011) Prevalence of *Giardia intestinalis* and zoonotic genotype predominance in small scale sheep and cattle farms in five states of the Mexican Republic. *Veterinaria México* 42: 219-226.
97. Lwin KS (2011) Prevalence of *Cryptosporidium, Giardia* and other internal parasites in dairy and beef cattle of Mae on District, Chiang Mai Thailand*.* Thesis, Chiang Mai University and Freie Universität Berlin, Chiang Mai, Thailand.
98. Tiranti K, Larriestra A, Vissio C, Picco N, Alustiza F, Degioanni A, Vivas A (2011) Prevalence of *Cryptosporidium* spp. and *Giardia* spp., spatial clustering and patterns of shedding in dairy calves from Córdoba, Argentina. Revista Brasileira de Parasitologia Veterinaria 20: 140-147.
99. Demeu FA, Lopes MA, Perazza CA, dos Santos G, de Carvalho AHO, Ribeiro ADB, Pedrosa MF, Guimaraes AM, Brhun FRP (2011) Prevalence of *Giardia duodenalis* and *Cryptosporidium* spp. in females of the Holstein breed during the post weaning phase in the herd in the southern state of Minas Gerais during the fall winter season of 2008. Boletim de Indústria Animal 68: 053-057. (in Spanish)
100. Khan SM, Debnath C, Pramanik AK, Xiao L, Nozaki T, Ganguly S (2011) Molecular evidence for zoonotic transmission of *Giardia duodenalis* among dairy farm workers in West Bengal, India. *Veterinary Parasitology* 178: 342-345.
101. Cardona GA, Carabin H, Goñi P, Arriola L, Robinson G, Fernández-Crespo JC, Clavel A, Chalmers RM, Carmena D (2011) Identification and molecular characterization of *Cryptosporidium* and *Giardia* in children and cattle populations from the province of Álava, North of Spain. *Science of the Total Environment* 412-413: 101-108.
102. Ilie MS, Sorescu ID, Oprescu I, Ilie A, Morariu F, Darabus G (2011) Prevalence of *Giardia* spp. infection in calves in Western Romania. *Current Opinion in Biotechnology* 22: S112
103. Paz e Silva FM, Lopes RS, Araújo JP Jr (2012) Genetic characterisation of *Giardia duodenalis* in dairy cattle in Brazil. *Folia Parasitologica* 59: 15-20.
104. Budu-Amoako E, Greenwood SJ, Dixon BR, Barkema HW, McClure JT (2012) *Giardia* and *Cryptosporidium* on dairy farms and the role these farms may play in contaminating water Sources in Prince Edward Island, Canada. *Journal of Veterinary Internal Medicine* 26: 668-673.
105. Budu-Amoako E, Greenwood SJ, Dixon BR, Barkema H.W, McClure JT (2012) Occurrence of *Cryptosporidium* and *Giardia* on beef farms and water sources within the vicinity of the farms on Prince Edward Island, Canada. *Veterinary Parasitology* 184: 1-9.
106. Ouchene N, Ouchene-Khelifi NA, Aissi M, Benakhla A (2012) Prévalence de *Cryptosporidium* spp*.* et *Giardia* spp. chez les bovins de la région de Sétif au nord-est de l’Algérie. *Revue d'Élevage et de Médecine Vétérinaire des Pays Tropicaux* 65: 53-56. (in French)
107. Mark-Carew MP, Wade SE, Chang YF, Schaaf S, Mohammed HO (2012) Prevalence of *Giardia duodenalis* assemblages among dairy herds in the New York City Watershed. *Veterinary Parasitology* 185: 15-157.
108. Santin M, Dargatz D, Fayer R (2012) Prevalence of *Giardia duodenalis* assemblages in weaned cattle on cow-calf operations in the United States. *Veterinary Parasitology* 183: 231-236.
109. Oates SC, Miller MA, Hardin D, Conrad PA, Melli A, Jessup DA, Dominik C, Roug A, Tinker MT, Miller WA (2012) Prevalence, environmental loading, and molecular Characterization of *Cryptosporidium* and *Giardia* Isolates from domestic and wild animals along the Central California Coast. *Applied Environmental Microbiology* 78: 8762-8772.
110. Muhid A, Robertson I, Ng J, Yang R, Ryan U (2012) Prevalence of *Giardia* spp. infection in pre-weaned and weaned calves in relation to management factors. *The Veterinary Journal* 191: 135-137.
111. Abeywardena H, Jex AR, Nolan MJ, Haydon SR, Stevens MA, McAnulty RW, Gasser RB (2012) Genetic characterisation of *Cryptosporidium* and *Giardia* from dairy calves: Discovery of species/genotypes consistent with those found in humans. *Infection, Genetics and Evolution* 12: 1984-1993.
112. Hernández-Gallo N, Cortés-Vecino JA (2012) *Cryptosporidium* spp. and *Giardia* spp. prevalence and risk factors in dairy calves of the north-western zone of the Bogota Savanna. *Revista de Salud Publica* 2012 4: 169-181. (in Spanish).
113. Liu A, Zhang X, Zhang L, Wang R, Li X, Shu J, Zhang X, Shen Y, Zhang W, Ling H (2012) Occurrence of bovine giardiasis and endemic genetic characterization of *Giardia duodenalis* isolates in Heilongjiang Province, in the Northeast of China. *Parasitology Reseaech* 111: 655-661.
114. Geurden T, Vanderstichel R, Pohle H, Ehsan A, von Samson-Himmelstjerna G, Morgan ER, Camuset P, Capelli G, Vercruysse J, Claerebout E (2012) A multicentre prevalence study in Europe on *Giardia duodenalis* in calves, with molecular identification and risk factor analysis. *Veterinary Parasitology* 190: 383-390.
115. Sorescu ID, Oprescu I, Morariu S, Mederle N, Ilie MS, Hotea I, Darabus GH (2012) The parasitism with *Giardia* spp. in calves from Caras-Severin county. *Lucrari Stiintifice Medicina Veterinara* XLV: 167-172.
116. Fayer R, Santin M, Macarisin D (2012) Detection of concurrent infection of dairy cattle with *Blastocystis*, *Cryptosporidium*, *Giardia*, and *Enterocytozoon* by molecular and microscopic methods. *Parasitology Research* 111: 1349-1355.
117. Zhang J, Su Y, Bai G, Wang C, Gao Y, Han F, et al (2012) Genotype identification of *Giardia* from cattle in part areas of northeast China. *China Journal of Veterinary Science* 32: 1679-1682 (in Chinese).
118. Wegayehu T, Adamu H, Petros B (2013) Prevalence of *Giardia duodenalis* and *Cryptosporidium* species infections among children and cattle in North Shewa Zone, Ethiopia. *BMC Infectious Diseases* 13: 419-426.
119. de Godoy EAM, Santos Junior JE, Belloto MVT, de Moraes MVP, Cassiano GC, Volotão ACC, Luvizotto MCR, Carareto CMA, de Moraes Silva MC, Machado RLD (2013) molecular investigation of zoonotic genotypes of *Giardia intestinalis* isolates in humans, dogs and cats, sheep, goats and Cattle in Araçatuba (São Paulo State, Brazil) by the analysis of β-giardin gene fragments. *Microbiology Research* 4: e6.
120. Magaji AA, Ibrahim K, Saulawa MA, Salihu MD, Mohammed AA (2013) Prevalence of giardiasis in cattle slaughtered in Sokoto metropolitan abattoir, Sokoto, Nigeria. *Scientific Journal of Veterinary Advances* 2: 76-78.
121. Sorescu ID, Ilie MS, Oprescu I, Morariu S, Mederle N, Hotea I, Imre M, Darabus G (2013) Immunoenzymatic assay to detect *Giardia* spp. in calves from western and south-western Romania. *Lucrari Stiintifice Medicina Veterinara* 46: 144-152.
122. Fava NMN, Soares RM, Scalia LAM, Kalapothakis E, Pena IF, Vieira CU, Faria ESM, Cunha MJ, Couto TR, Cury MC (2013) Performance of glutamate dehydrogenase and triose phosphate isomerase genes in the analysis of genotypic variability of isolates of *Giardia duodenalis* from livestocks. *BioMed Research International* Article ID 875048: 9 pages.
123. Ferreira-Feitosa T, Longo Riberio Vilela V, Rudrigues Athayde AC (2013) First report of *Cryptosporidium* spp. and *Giardia* *duodenalis* in calves from northeastern Brazil. *Turkish Journal of Veterinary and Animal Sciences* 37: 743-746.
124. Ferreira-Fagundes TF, Vidal LGP, Alves PAM, McIntosh D, Menezes R. CAA, Fonseca AH, Pereira MJS (2013) Molecular characterization of *Giardia intes­tinalis* in heifer calves reared in individual shelters within paddocks at the Munici­pality of Piraí in the State of Rio de Janeiro, Brazil. *Revista Brasileira de Medicina Veterinária* 35: 17-21.
125. Roug A, Byrne BA, Conrad PA, Miller WA (2013) Zoonotic fecal pathogens and antimicrobial resistance in county fair animals. *Comparative Immunology, Microbiology and Infectous Diseases* 36: 303-308.
126. Minetti C, Taweenan W, R. Hogg R, Featherstone C, Randle N, Latham SM, Wastling JM (2014) Occurrence and diversity of *Giardia duodenalis* assemblages in livestock in the UK. *Transboundary and Emerging Diseases* 61: e60-e67.
127. Wang H, Zhao G, Chen G, Jian F, Zhang S, Feng C, et al. (2014) Multilocus genotyping of *Giardia duodenalis* in dairy cattle in Henan, China. *PLoS* *ONE* 9: e100453.
128. Ouchene N, Ouchene-Khelifi NA, Zeroual F, Benakhla A, Adjou K (2014) Study of *Giardia* spp., *Cryptosporidium* spp. and *Eimeria* spp. infections in dairy cattle in Algeria. *Journal of Parasitology and Vector Biology* 6: 61-65.
129. Hogan JN, [Miller](https://pubmed.ncbi.nlm.nih.gov/?term=Miller+WA&cauthor_id=24171566) WA, [Cranfield](https://pubmed.ncbi.nlm.nih.gov/?term=Cranfield+MR&cauthor_id=24171566) MR, [Ramer](https://pubmed.ncbi.nlm.nih.gov/?term=Ramer+J&cauthor_id=24171566) J, [Hassell](https://pubmed.ncbi.nlm.nih.gov/?term=Hassell+J&cauthor_id=24171566) J, [Noheri](https://pubmed.ncbi.nlm.nih.gov/?term=Noheri+JB&cauthor_id=24171566) JB, [Conrad](https://pubmed.ncbi.nlm.nih.gov/?term=Conrad+PA&cauthor_id=24171566) PA,  [Gilardi](https://pubmed.ncbi.nlm.nih.gov/?term=Gilardi+KV&cauthor_id=24171566) KVK (2014) *Giardia* in mountain gorillas (*Gorilla beringei beringei*), forest buffalo (*Syncerus caffer*), and domestic cattle in Volcanoes National Park, Rwanda. *Journal of Wildlife Diseases* 50: 21-30.
130. Helmy YA, Klotz C, Wilking H, Krücken J, Nöckler K, Von Samson-Himmelstjerna G, et al. (2014) Epidemiology of *Giardia duodenalis* infection in ruminant livestock and children in the Ismailia province of Egypt: insights by genetic characterization. *Parasites & Vectors* 7: 321.
131. Huang J, Yue D, Qi M, Wang R, Zhao J, Li J, Shi K, Wang M, Zhang L (2014) Prevalence and molecular characterization of *Cryptosporidium* spp. and *Giardia duodenalis* in dairy cattle in Ningxia, northwestern China. *BMC Veterinary Research* 10: 292.
132. Ananta SM, Suharno, Hidayat A, Matsubayashi M (2014) Survey on gastrointestinal parasites and detection of *Cryptosporidium* spp. On cattle in West Java, Indonesia. *Asian Pacific Journal of Tropical Medicine* 7: 197-201.
133. Wang H, Qi M, Li A, Luo N, Zhou H, Wang M (2014) Investigation of intestinal parasites infection in dairy cattle in Kaifeng, China. Animal Husbandry Veterinary Medicine 46: 84-87 (in Chinese).
134. Gillhuber J, Rügamer D, Pfister K, Scheuerle M (2014) Giardiosis and other enteropathogenic infections: a study on diarrhoeic calves in Southern Germany. *BMC Research Notes* 7: 112-120.
135. Abeywardena H, Jex AR, Koehler AV, Rajapakse RJ, Udayawarna K, Haydon SR, et al. (2014) First molecular characterization of *Cryptosporidium* and *Giardia* from bovines (*Bos taurus* and *Bubalus bubalis*) in Sri Lanka: unexpected absence of *C. parvum* from pre-weaned calves. *Parasites & Vectors* 7:75.
136. Al-Saad RK, Al-Emarah GY (2014) Giardiasis in cattle: clinical assessment of *Giardia lamblia* in cattle at Basrah, Iraq. *International Journal of Scientific World* 2: 80-83.
137. Tronciu C, Miron LD, Acatrinei DM, Pavel I, Alexa AM, Ciucă L, Ișan E (2014) Preliminary research on the parasitic complex of *Eimeria, Giardia* and *Cryptosporidium* in young cattle from northern Moldavia. *Lucrari Stiintifice Medicina Veterinara* 47: 120-125.
138. Sharma S, Katoch R, Yadav A, Godara R (2014) *Giardia* prevalence in bovine calves in Jammu, India. Veterinary Practitioner 15: 272-273
139. Ehsan AM, Geurden T, Casaert S, Parvin SM, Islam TM, Ahmed UM, Levecke B, Vercruysse J, Claerebout E (2015) Assessment of zoonotic transmission of *Giardia* and *Cryptosporidium* between cattle and humans in rural villages in Bangladesh. *PLoS ONE* 10: e0118239.
140. Cardona GA, de Lucio A., Bailo B., Cano L, de Fuentes I, Carmena D (2015) Unexpected finding of feline-specific *Giardia duodenalis* assemblage F and *Cryptosporidium felis* in asymptomatic adult cattle in Northern Spain. *Veterinary Parasitology* 209: 258-263.
141. Liu G, Su Y, Zhou M, Zhao J, Zhang T, Ahmad W, Lu H, Jiang N, Chen Q, Xiang M, Yin J (2015) Prevalence and molecular characterization of *Giardia duodenalis* isolates from dairy cattle in northeast China. *Experimental Parasitology* 154: 20-24
142. Das M, Deka DK, Sarmah PC, Islam S, Laha R (2015) *Giardia duodenalis* infection in dairy cattle of Assam, India. *Asian Journal of Animal and Veterinary Advances* 10: 911-917.
143. Peter SG, Gitau GK, Mulei CM, Vanleeuwen J, Richards S, Wichtel J, Uehlinger F, Mainga O (2015) Prevalence of *Cryptosporidia*, *Eimeria*, G*iardia,* and *Strongyloides* in pre-weaned calves on smallholder dairy farms in Mukurwe-ini district, Kenya. *Veterinary World* 8: 1118-1125.
144. Inpankaew T, Jiyipong T, Thadtapong N, Kengradomkij C, Pinyopanuwat N, Chimnoi W, Jittapalapong S (2015) Prevalence and genotype of *Giardia duodenalis* in dairy cattle from northern and northeastern part of Thailand. *Acta Parasitology* 60: 459-461.
145. Daniels ME, Shrivastava A, Smith WA, Sahu P, Odagiri M, Misra PR, Panigrahi P, Suar M, Clasen T, Jenkins MW (2015) *Cryptosporidium* and *Giardia* in humans, domestic animals, and village water sources in rural India. *American Journal of Tropical Medicine and Hygiene* 93:596-600.
146. Stojecki K., Sroka J., Cencek T., Dutkiewicz J (2015) Epidemiological survey in Łęczyńsko-Włodawskie lake district of eastern Poland reveals new evidence of zoonotic potential of *Giardia intestinalis*. *Annals of Agricultural and Environmental Medicine* 22: 594-598.
147. Onac D, Jarca A, Kalmar Z, Cozma V (2015) *Giardia duodenalis* in calves from an isolated farm from northwestern Romania. Scientica Parasitologica 16: 133-137
148. Qi M, Wang H, Jing B, Wang R, Jian F, Ning C, Zhang L (2016) Prevalence and multilocus genotyping of *Giardia duodenalis* in dairy calves in Xinjiang, Northwestern China. *Parasites & Vectors* 9:546.
149. Ahmed SO, Hamed MI, Yones DA (2016) Molecular and conventional detection of zoonotic *Giardia* and *Cryptosporidium* in children and calves in upper Egypt. *American Journal of Infectious Diseases and Microbiology* 4: 91-94
150. Al-Difaie RS (2016) Molecular study to detect genotyping of *Giardia lamblia* from human and cattle feces in Al-Qadisiya governorate, Iraq. *Ibn Al-Haitham Journal for Pure & Applied Sciences* 29: 1-13.
151. Kakandelwa C, Siwila J, Nalubamba KS, Muma JB, Phiri IGK (2016) Prevalence of *Giardia* in dairy cattle in Lusaka and Chilanga districts, Zambia. *Veterinary Parasitology* 215: 114-116.
152. Lee SH, VanBik D, Kim HY, Cho A, Kim JW, Byun JW, Oem JK, Oh SI, D. Kwak D (2016) Prevalence and molecular characterisation of *Giardia duodenalis* in calves with diarrhoea. *Veterinary Record* 178: 633-633*.*
153. Wang C, Zhang Z, Li J, Yu F, Cao J, Zhang L (2016) The investigation of infection and multilocus sequence of *Giardia duodenalis* in dairy cattle from an imported farm. *Acta Veterinaria et Zootechnica Sinica* 47: 165-171 (in Chinese).
154. Zhao J, Wang H, Qi M, Yu F, Liu Q, Zhang L (2016) Prevalence and multilocus genotyping of *Giardia duodenalis* in dairy cattle in Kaifeng, China. Veterinary Science China 4: 496-501 (in Chinese).
155. Wegayehu T, Karim MR, Erko B, Zhang L, Tilahun G (2016) Multilocus genotyping of *Giardia duodenalis* isolates from calves in Oromia Special Zone, Central Ethiopia. *Infection,* *Genetics and Evolution* 43: 281-288.
156. Li F, Wang H, Zhang Z, Li J, Wang C, Zhao J, Hu S, Wang R, Zhang L, Wang M (2016) Prevalence and molecular characterization of *Cryptosporidium* spp. and *Giardia duodenalis* in dairy cattle in Beijing, China. *Veterinary Parasitology* 219: 61-65.
157. Nasiri V, Karimi G, Ashtari A, Rivaz S, Paykari H (2016) Prevalence of gastrointestinal protozoa infections in diarrheal neonatal and young calves in Mahdasht region, Karaj, Alborz. In: *9^th^ International Congress of Laboratory and Clinic, Iran*.
158. Nguyen ST, Fukuda Y, Nguyen DT, Tada C, Nakai Y (2016) Prevalence and first genotyping of *Giardia duodenalis* in beef calves in Vietnam. *Tropical Animal Health and Production* 48: 837-841.
159. Asher AJ, Hose G, Power ML (2016) Giardiasis in NSW: Identification of *Giardia duodenalis* assemblages contributing to human and cattle cases, and an epidemiological assessment of sporadic human giardiasis. *Infection, Genetics and Evolution* 44: 157-161.
160. Wang XT, Wang RJ, Ren GJ, Yu ZQ, Zhang LX, Zhang SY, Lu H, Peng XQ, Zhao GH (2016) Multilocus genotyping of *Giardia duodenalis* and *Enterocytozoon bieneusi* in dairy and native beef (Qinchuan) calves in Shaanxi province, northwestern China. *Parasitology Research* 115: 1355-1361.
161. Zhang XX, Tan QD, Zhao GH, Ma JG, Zheng WB, Ni XT, et al. (2016) Prevalence, risk factors and multilocus genotyping of *Giardia intestinalis* in dairy cattle, Northwest China. *Journal of Eukaryotic Microbiology* 63: 498-504.
162. Raue K, Heuer L, Böhm C, Wolken S, Epe C, Strube C (2017) 10-year parasitological examination results (2003 to 2012) of faecal samples from horses, ruminants, pigs, dogs, cats, rabbits and hedgehogs. *Parasitology Research* 116: 3315-3330.
163. Baroudi D, Khelef D, Hakem A, Abdelaziz A, Chen X, Lysen C, Roellig D, Xiao L (2017) Molecular characterization of zoonotic pathogens *Cryptosporidium* spp., *Giardia duodenalis* and *Enterocytozoon bieneusi* in calves in Algeria. *Veterinary Parasitology: Regional Studies and Reports* 8: 66-69.
164. Squire SA, Yang R, Robertson I, Ayi I, Ryan U (2017) Molecular characterization of *Cryptosporidium* and *Giardia* in farmers and their ruminant livestock from the Coastal Savannah zone of Ghana. *Infection Genetics and Evolution* 55: 236-243.
165. Volpato A, Tonin AA, Machado G, Stefani LM, Campigotto G, Glombowsky P, Galli GM, Favero JF, da Silva AS (2017) Gastrointestinal protozoa in dairy calves: identification of risk factors for infection. *Revista MVZ Córdoba* 22: 5910-5924
166. Nolan MJ, Unger M, Yeap YT, Rogers E, Millet I, Harman K, Fox M, Kalema-Zikusoka G, Blake DP (2017) Molecular characterisation of protest parasites in human habituated mountain gorillas (*Gorilla beringei beringei*), humans and livestock, from Bwindi Impenetrable National Park, Uganda. *Parasites & Vectors* 10: 340.
167. Wang X, Cai M, Jiang W, Wang Y, Jin Y, Li N, Guo Y, Feng Y, Xiao L (2017) High genetic diversity of *Giardia duodenalis* assemblage E in pre-weaned dairy calves in Shanghai, China, revealed by multilocus genotyping. *Parasitology Research* 116: 2101-2110.
168. Fan Y, Wang T, Koehler AV, Hu M, Gasser RB (2017) Molecular investigation of *Cryptosporidium* and *Giardia* in pre- and post-weaned calves in Hubei Province, China. *Parasites & Vectors* 10: 519.
169. Hu S, Liu Z, Yan F, Zhang Z, Zhang G, Zhang L, Jian F, Zhang S, Ning C, Wang R (2017) Zoonotic and host-adapted genotypes of *Cryptosporidium* spp., *Giardia duodenalis* and *Enterocytozoon bieneusi* in dairy cattle in Hebei and Tianjin, China. *Veterinary Parasitology* 248: 68-73.
170. Gultekin M, Ural K, Aysul N, Ayan A, Balikci C, Toplu S, Akyildiz G (2017) Prevalence and molecular characterization of *Giardia duodenalis* in calves in Turkey. *Acta Scientiae Veterinariae* 45: 1450.
171. Mahato MK, Singh DK, Rana HB, Acharya KP (2018) Prevalence and risk factors associated with *Giardia duodenalis* infection in dairy cattle of Chitwan, Nepal. *Journal of Parasitic Diseases* 42: 122-126.
172. Urie NJ, Lombard JE, Shivley CB, Adams AE, Kopral CA, Santin M (2018) Preweaned heifer management on US dairy operations: Part III. Factors associated with *Cryptosporidium* and *Giardia* in preweaned dairy heifer calves. *Journal of Dairy Science* 101: 9199-9213.
173. Malekifard F, Ahmadpour M (2018) Molecular detection and identification of *Giardia duodenalis* in cattle of Urmia, northwest of Iran. *Veterinary Research Forum* 9: 81-85.
174. Jian Y, Zhang X, Li X, Karanis G, Ma L, Karanis P (2018) Prevalence and molecular characterization of *Giardia duodenalis* in cattle and sheep from the Qinghai-Tibetan Plateau Area (QTPA), northwestern China. *Veterinary Parasitology* 250: 40-44.
175. Naguib D, El-Gohary AH, Mohamed AA, Roellig DM, Arafat N, Xiao L (2018) Age patterns of *Cryptosporidium* species and *Giardia duodenalis* in dairy calves in Egypt. *Parasitology International* 67: 736-741.
176. Cui Z, Wang L, Cao L, Sun M, Liang N, Wang H, Chang Y, Lin X, Yu L, Wang R, Zhang S, Ning C, Zhang L (2018) Genetic characteristics and geographic segregation of *Giardia duodenalis* in dairy cattle from Guangdong Province, southern China. *Infection, Genetics and Evolution* 66: 95-100
177. Zhong Z, Dan J, Yan G, Tu R, Tian Y, Cao S, Shen L, Deng J, Yu S, Geng Y, Gu X, Wang Y, Liu H, Peng G (2018) Occurrence and genotyping of *Giardia duodenalis* and *Cryptosporidium* in pre-weaned dairy calves in central Sichuan province, China. *Parasite* 25: 45.
178. Sevá AP, Pena HFJ, Nava A, Sousa AO, Holsback L, Soares RM (2018) Endoparasites in domestic animals surrounding an Atlantic Forest remnant, in São Paulo state, Brazil. *Brazilian Journal of Veterinary Parasitology* 27: 1218.
179. Lee YJ, Han DG, Ryu JH, Chae JB, Chae JS, Yu DH, Park J, Park BK, Kim HC, Choi KS (2018) Identification of zoonotic *Giardia duodenalis* in Korean native calves with normal feces. *Parasitology Research* 117: 1969-1973.
180. Kozat S, Tuncay I (2018) Prevalence of *Rotavirus*, *Coronavirus*, *Cryptosporidium* spp., *Escherichia* *coli* K99, and *Giardia lamblia* pathogens in neonatal calves with diarrheic in Siirt Region. *Van Veterinary Journal* 29: 17-22.
181. Mensah GT, Annang AK, Ayeh-Kumi PF, Oppong JA, Niampoma S (2019) Prevalence of *Giardia* species in cattle faecal matter in selected farms in Weija and Kpong major water supply heads to Accra, Ghana. *Ghana Journal of Science* 60: 63-73.
182. Li X, Flores KA, Barry S, Becchetti TA, Doran M, Finzel JA, Larsen R, Lile D, McDougald N, Nguyen T, Xiao C, Atwill ER (2019) Statewide cross-sectional survey of *Cryptosporidium* and *Giardia* in California cow-calf herds. *Rangeland Ecology & Management* 72: 461-466.
183. Miambo RD, Laitela B, Malatji MP, De Santana Afonso SM, Junior AP, Lindh J, et al. (2019) Prevalence of *Giardia* and *Cryptosporidium* in young livestock and dogs in Magude District of Maputo Province, Mozambique. *Onderstepoort Journal of Veterinary Research* 86: a1709.
184. Lee SH, Kim HY, Choi EW, Kim D (2019) Causative agents and epidemiology of diarrhea in Korean native calves. *Journal of Veterinary Science* 20: e64.
185. Dan J, Zhang X, Ren Z, Wang L, Cao S, Shen L, Deng J, Zuo Z, Yu S, Wang Y, Ma X, Liu H, Zhou Z, Hu Y, Fu H, He C, Geng Y, Gu X, Peng G, Zhong Z (2019) Occurrence and multilocus genotyping of *Giardia duodenalis* from post-weaned dairy calves in Sichuan province, China. *PLoS ONE* 14: e0224627.
186. Bartley PM, Roehe BK, Thomson S, Shaw HJ, Peto F, Innes EA, Katzer F (2019) Detection of potentially human infectious assemblages of *Giardia duodenalis* in fecal samples from beef and dairy cattle in Scotland. *Parasitology* 146: 1123-1130.
187. Hastutiek P, Yuniarti WM, Djaeri M, Lastuti NDR, Suprihati E, Suwanti LT (2019) Prevalence and diversity of gastrointestinal protozoa in Madura cattle at Bangkalan Regency, East Java, Indonesia. *Veterinary World* 12: 198-204
188. Kiani-Salmi N, Fattahi-Bafghi A, Astani A, Sazmand A, Zahedi A, Firoozi Z, Ebrahimi B, Dehghani-Tafti A, Ryan U, Akrami-Mohajeri F (2019) Molecular typing of *Giardia* *duodenalis* in cattle, sheep and goats in an arid area of central Iran. *Infection, Genetics and Evolution* 75: 104021.
189. Sawitri DH, Wardhana AH, Martindah E, Ekawasti F, Dewi DA, Utomo BN, Shibahara T, Kusumoto M, Tokoro M, Sasai K, Matsubayashi M (2020) Detections of gastrointestinal parasites, including *Giardia intestinalis* and *Cryptosporidium* spp., in cattle of Banten province, Indonesia. *Journal of Parasitic Diseases* 44: 174-179.
190. Wang R, Li N, Jiang W, Guo Y, Wang X, Jin Y, Feng Y, Xiao L (2019) Infection patterns, clinical significance, and genetic characteristics of *Enterocytozoon bieneusi* and *Giardia duodenalis* in dairy cattle in Jiangsu, China. *Parasitology Research* 118: 3053-3060.
191. Feng Y, Gong X, Zhu K, Li N, Yu Z, Guo Y, Weng Y, Kváč M, Feng Y, Xiao L (2019) Prevalence and genotypic identification of *Cryptosporidium* spp., *Giardia duodenalis* and *Enterocytozoon bieneusi* in pre-weaned dairy calves in Guangdong, China. *Parasites & Vectors* 12: 41.
192. Lichtmannsperger K, Hinney B, Joachim A, Wittek T (2019) Molecular characterization of *Giardia intestinalis* and *Cryptosporidium* *parvum* from calves with diarrhoea in Austria and evaluation of point-of-care tests. *Comparative Immunology, Microbiology and Infectious Disease* 66: 101333.
193. Koh BRD, Kim HJ, Oh AR, Jung BR, Park JS, Lee JG, Na HM, Kim YH (2019) Prevalence of enteropathogens in the feces from diarrheic Korean native cattle in Gwangju area, Korea. *Korean Journal of Veterinary Service* 42: 93-112. (In Korean)
194. Susana Y, Tri Suwanti L, Suprihati E (2019) Identification and prevalence of gastrointestinal parasites in beef cattle in Siak Sri Indrapura, Riau, Indonesia. *Indonesian Journal of Tropical and Infectious Disease* 7: 155-160.
195. Afable AL, De Belen Coquilla KJ, Battad II ZG, Pornobi KO (2019) Detection of potentially zoonotic *Cryptosporidium* and *Giardia* among livestock in Sariaya, Quezon, Philippines. *Pertanika Journao of Tropical Agricultural Science* 42: 557-568
196. Lee YJ, Ryu JH, Shin SU, Choi KS (2019) Prevalence and molecular characterization of *Cryptosporidium* and *Giardia* in pre-weaned native calves in the Republic of Korea. *Parasitology Research*; 118: 3509-3517.
197. Ayan A, Ural DA, Erdogan H, Kilinc OO, Gultekin M, Ural K (2019) Prevalence and molecular characterization of *Giardia duodenalis* in livestock in Van, Turkey. *International Journal of Ecosystems and Ecology Science* 9: 289-296.
198. Hailu M, Asmare K, Gebremedhin EZ, Sheferaw D, Gizaw D, Di Marco V, Vitale M (2020) *Cryptosporidium* and *Giardia* infections in dairy calves in southern Ethiopia. *Parasite Epidemiology and Control* 10: e00155.
199. Onder Z, Simsek E, Duzlu O, Yetismis G, Ciloglu A, Okur M, Kokcu ND, Inci A, Yildirim A (2020) Molecular prevalence and genotyping of *Giardia duodenalis* in cattle in Central Anatolia Region of Turkey. *Parasitology Research* 119: 2927-2934.
200. Makawi ZA, Abbas AK (2020) Prevalence of intestinal protozoa in cattle in some areas of Wasit province. *Plant Archives* 20: 3667-3672.
201. Li S, Zou Y, Zhang XL, Wang P, Chen XQ, Zhu XQ (2020) Prevalence and multilocus genotyping of *Giardia lamblia* in cattle in Jiangxi Province, China: novel assemblage E subtypes identified. *Korean Journal of Parasitology* 58: 681-687.
202. Alhayali NS, Alneema MS, Suleiman EG (2020) Detection of *Giardia duodenalis* in cattle in Mosul city, Iraq. *Egyptian Journal of Veterinary Sciences* 51: 381-390
203. Ganai A, Yadav A, Katoch R, Godara R., Borkataki S (2020) Epidemiological factors affecting prevalence of *Giardia intestinalis* infection in cattle calves of Jammu region. *Journal of Community Mobilization and Sustainable Development* 15: 505-511.
204. Madlol NAB, Ameer QJ, Al-Kaabawi NAM (2020) Molecular detection of *Giardia lamblia* isolated from cattle feces. *Indian Journal of Public Health Research & Development* 11: 1778-1783.
205. Peña-Quistial MG, Benavides-Montaño JA, Duque NJR, Benavides-Montaño GA (2020) Prevalence and associated risk factors of intestinal parasites in rural high-mountain communities of the Valle del Cauca - Colombia. *PLoS Neglected Tropical Diseases* 14: e0008734.
206. Zahedi A, Odgers T, Ball A, Watkinson A, Robertson I, Ryan UM (2020) Longitudinal analysis of *Giardia duodenalis* assemblages in animals inhabiting drinking water catchments in New South Wales and Queensland - Australia (2013-2015). *Science of the Total Environment* 718: 137433.
207. Wang Y, Cao J, Chang Y, Yu F, Zhang S, Wang R, Zhang L (2020) Prevalence and molecular characterization of *Cryptosporidium* spp. and *Giardia duodenalis* in dairy cattle in Gansu, northwest China. *Parasite* 27: 62.
208. Jasim GA, Alkhanaq MN, Al-Ardi MH, Alrammah HSAA (2021) Use of molecular method to detect giardiasis in different animal in Al-Qadissiya province - Iraq. *Indian Journal of Forensic* *Medicine & Toxicology* 15: 2949-2957.
209. Iwashita H, Sugamoto T, Takemura T, Tokizawa A, Vu TD, Nguyen TH, Pham TD, Tran NL, Doan HT, Pham AHQ, Yamashiro T (2020) Molecular epidemiology of *Giardia* spp. in northern Vietnam: potential transmission between animals and humans. *Parasite Epidemiology and Control* 12: e00193.
210. Cibot M, McLennan MR, Kváˇc M, Sak B, Asiimwe C, Petrželková K (2021) Sparse evidence for *Giardia intestinalis*, *Cryptosporidium* spp. and *Microsporidia* infections in humans, domesticated animals and wild nonhuman primates sharing a farm-forest mosaic landscape in Western Uganda. *Pathogens* 10: 933.
211. Ma N, Wang HX, Tao WF, Xue NY, Bai JY, Zhao Q, Jiang J, Lyu C (2021) Detection of point prevalence and assemblages of *Giardia* spp. in dairy calves and sika deer, Northeast China. *Vector Borne Zoonotic Diseases* 21: 685-691.
212. Kuthyar S, Kowalewski MM, Seabolt M, Roellig DM, Gillespie TR (2021) Molecular characterization of *Giardia duodenalis* and evidence for cross-species transmission in Northern Argentina. *Transboundary and Emerging Diseases* 69: 2209-2218.
213. Lam HYP, Chen TTW, Tseng YC, Chang KC, Yang TH, Peng S (2021) Detection and genotyping of *Giardia duodenalis* from cattle and pigs in Hualien country, Eastern Taiwan. *Journal of Microbiology, Immunology and Infection* 54: 718-727.
214. Hossain MS, Sultana NN, Akter S, Labony SS, Anisuzzaman (2021) A retrospective survey of gastrointestinal parasites in livestock of Hilly areas in Mymensingh. *Journal of Bangladesh Agricultural University* 19: 332-339.
215. Liang XX, Zou Y, Li TS, Chen H, Wang SS, Cao FQ, Yang JF, Sun XL, Zhu XQ, Zou FC (2021) First report of the prevalence and genetic characterization of *Giardia duodenalis* and *Cryptosporidium* spp. in Yunling cattle in Yunnan Province, southwestern China. *Microbial* *Pathogenesis* 158: 105025.
216. Barghash SM, Taha SA, Serag SAS, Ragab EA (2021) Identifications and genotypes of *Giardia intestinalis* in ruminant livestock in south Sinai Governorate, Egypt. *Journal of the Egyptian Society of Parasitology* 51: 617-626.
217. González-Ramírez LC, Vázquez CJ, Chimbaina MB, Djabayan-Djibeyan P, Prato-Moreno JG, Trelis M, Fuentes MV (2021) Ocurrence of enteroparasites with zoonotic potential in animals of the rural area of San Andres, Chimborazo, Ecuador. *Veterinary Parasitology: Regional Studies and Reports* 26: 100630.
218. ElBakri A, Salahat DF, Hussein NM, Ibrahim ZA, Abu Odeh RO (2021) First report of *Giardia lamblia* in different animals in the United Arab Emirates. *Tropical Biomedicine* 38: 180-182.
219. Oh SI, Jung SH, Lee HK, Choe C, Hur TY, So KM (2021) Multilocus genotyping of *Giardia duodenalis* occurring in Korean native calves. *Veterinary* *Sciences* 8: 118.
220. Wei X, Wang W, Dong Z, Cheng F, Zhou X, Li B, Zhang J (2021) Detection of infectious agents causing neonatal calf diarrhea on two large dairy farms in Yangxin county, Shandong province, China. *Frontiers in Veterinary Science* 7: 589126.
221. Hublin JSY, Maloney JG, George NS, Molokin A, Lombard JE, Urie NJ, Shivley CB, Santin M (2022) Enhanced detection of *Giardia duodenalis* mixed assemblage infections in pre-weaned dairy calves using next generation sequencing. *Veterinary Parasitology* 304: 109702.
222. Kifleyohannes T, Nødtvedt A, Debenham JJ, Terefe G, Robertson LJ (2022) *Cryptosporidium* and *Giardia* in livestock in Tigray, Northern Ethiopia and associated risk factors for infection: A cross-sectional study. *Frontiers in Veterinary Science* 8: 825940.
223. Fu Y, Dong H, Bian X, Qin Z, Han H, Lang J, Zhang J, Zhao G, Li J, Zhang L (2022) Molecular characterizations of *Giardia duodenalis* based on multilocus genotyping in sheep, goats, and beef cattle in Southwest Inner Mongolia, China. *Parasite* 29: 33.
224. Heng ZJ, Yang JF, Xie XY, Xu CR, Chen JR, Ma J, He JJ, Mao HM (2022) Prevalence and multilocus genotyping of *Giardia duodenalis* in Holstein cattle in Yunnan, China. *Frontiers in Veterinary Science* 9: 949462.
225. Wu Y, Yao L, Chen H, Zhang W, Jiang Y, Yang F, Liu A, Shen Y (2022) *Giardia duodenalis* in patients with diarrhea and various animals in northeastern China: prevalence and multilocus genetic characterization. *Parasites & Vectors* 15: 165.
226. Awais MM, Ihsan-Ul-Haq H, Akhtar M, Anwar MI, Shirwany ASAK, Abdul Razzaq A, Bhatti MS (2023) Copro-ELISA-based prevalence and risk determinants of giardiasis in cattle and sheep populations raised by socio-economically deprived urban nomadic communities located in and around Multan, Punjab-Pakistan. *Biological Rhythm Research* 54: 291-306.
227. Celik BA, Çelik OY, Ayan A, Akyildiz G, Kilinç OO, Ayan OO, Ercan K (2023) Molecular prevalence of *Giardia duodenalis* and subtype distribution (assemblage E and B) in calves in Siirt, Turkey. *Egyptian Journal of Veterinary Sciences* 54: 457-463.
228. Li J, Karim MR, Siddiki SHMF, Chen Y, Qin Z, Rume FI, Zhang L (2023) Potential zoonotic transmission of *Giardia duodenalis* between children and calves in Bangladesh. *Transboundary and Emerging Diseases* Article ID:8224587.
229. Hatam-Nahavandi K, Carmena D, Rezaeian M, Mirjalali H, Mohammad Rahimi H, Badri M, Vafae Eslahi A, Faraji Shahrivar F, Rodroguez Oliveira SM, de Lourdes Pereira M, Ahmadpour E (2023) Gastrointestinal parasites of domestic mammalian hosts in southeastern Iran. *Veterinary* *Sciences* 10: 261.
230. Zhao L, Zhang ZS, Han WX, Yang B, Chai HL, Wang MY, Wang Y, Zhang S, Zhao WH, Ma YM, Zhan YJ, Wang LF, Ding YL, Wang JL, Liu YH (2023) Prevalence and molecular characterization of *Giardia duodenalis* in dairy cattle in central inner Mongolia, Northern China. *Scientific Reports* 13: 13960.
231. Park YJ, Cho HC, Jang DH, Park J, Choi KS (2023) Multilocus genotyping of *Giardia duodenalis* in pre-weaned calves with diarrhea in the Republic of Korea. *PLoS One* 18: e0279533.
232. Gao H, Liang G, Su N, Li Q, Wang D, Wang J, Zhao L, Kang X, Guo K (2023) Prevalence and molecular characterization of *Cryptosporidium* spp., *Giardia duodenalis*, and *Enterocytozoon bieneusi* in diarrheic and non-diarrheic calves from Ningxia, Nouthwestern China. *Animals* 13: 1983.
233. Alseady HH, Al-Dabbagh SMK, Marhash AD (2023) Prevalence and molecular characterization of *Giardia intestinalis* isolates from children and calves in Babylon province, Iraq. *Veterinary World* 16: 1781-1789.
234. Mamak N, Kiyici R, Sahinduran S, Sensoy S, Akkan HA, Karaca M, Yildiz R, Musabesoglu Y, Gokce HI (2023) Etiological examination of neonatal calf diarrhea cases detected in Burder region. *Veterinary Journal of Mehmet Akif Ersoy University* 8: 55-60.
235. Figueiredo AN, Koster PC, Dashti A, Torres RT, Fonseca C, Mysterud A, Bailo B, Carvalho J, Ferreira E, Hipolito D, Fernandes J, Lino A, Palmeira JD, Sarmento P, Neves N, Carrapato C, Calero-Bernal R, Carmena D (2023) Molecular detection and distribution of *Giardia duodenalis* and *Cryptosporidium* spp. infections in wild and domestic animals in Portugal. *Transboundary and Emerging Diseases* Article ID:5849842.
236. Di Cristanziano V, Santoro M, Parisi F, Albonico M, Shaali MA, Di Cave D, Berrilli F (2014) Genetic characterization of *Giardia duodenalis* by sequence analysis in humans and animals in Pemba Island, Tanzania. *Parasitology International* 63: 438-441.
237. Bawm S, Kyi S, Lay KK, Htun LL, Myaing TT (2014) Prevalence and associated risk factors of *Cryptosporidium* and *Giardia* species in cattle within Mandalay region, Myanmar. *Journal of Advances in Parasitology* 1: 49-53.
238. Toledo RdS, Martins FDC, Ferreira FP, de Almeida JC, Ogawa L, dos Santos HLEPL, dos Santos MM, Pinheiro FA, Navarro IT, Garcia JL, Freire RL (2017) *Cryptosporidium* spp. and *Giardia* spp. in feces and water and the associated exposure factors on dairy farms. *PLoS ONE* 12: e0175311.
239. Debenham JJ, Tysnes K, Khunger S, Robertson LJ (2017) Occurrence of *Giardia*, *Cryptosporidium*, and *Entamoeba* in wild rhesus macaques (*Macaca mulatta*) living in urban and semi-rural North-West India. International Journal for Parasitology: *Parasites and Wildlife* 6: 29-34.
240. Spencer LA., Irwin MT (2020) *Cryptosporidium* and *Giardia* prevalence amongst lemurs, humans, domestic animals and black rats in Tsinjoarivo, Madagascar. *Heliyon* 6: e05604
241. Ribeiro MG, Langoni H, Jerez JA, Leite DS, Ferreira F, Gennari SM (2000) Identification of enteropathogenes from buffalo calves with and without diarrhoea in the Ribeira Valley, State of Sao Paulo, Brazil. *Brazilian Journal of Veterinary Research and Animal Science* 37: 159-165.
242. Goraya K, Saeed K, Hashmi HA, Khan MS, Hussain MH (2004) Prevalence and shedding intensity of giardiasis in naturally infected buffaloes. *International Journal of Agriculture and Biology* 6: 471-473.
243. Caccio SM, Rinaldi L, Cringoli G, Condoleo R, Pozio E (2007) Molecular identification of *Cryptosporidium parvum* and *Giardia duodenalis* in the Italian water buffalo (*Bubalus* *bubalis*). *Veterinary Parasitology* 150: 146-149.
244. Rinaldi L, Musella V, Condoleo R, Saralli G, Veneziano V, Bruni G, Condoleo RU, Cringoli G (2007) *Giardia* and *Cryptosporidium* in water buffaloes (*Bubalus* *bubalis*). *Parasitology Research* 100: 1113-1118.
245. Abeywardena H, Jex AR, von Samson-Himmelstjerna G, Haydon SR, Stevens MA, Gasser RB (2013) First molecular characterisation of *Cryptosporidium* and *Giardia* from *Bubalus bubalis* (water buffalo) in Victoria, Australia. *Infection, Genetics and Evolution* 20: 96-102.
246. Khurajog B, Masakul A, Inpankaew T, Kamyingkird K, Wongpanit K, Jittapalapong S (2014) Prevalence of *Giardia duodenalis* and factors associated with its infection in water buffaloes in Northeast Thailan. *Journal of Tropical Medicine and Parasitology* 37: 35-41.
247. Gharieb RMA, El-Ghany AMA (2016) *Giardia lamblia* in Ho persons and buffalo calves; prevalence, molecular identification and associated risk factors. *Japanese Journal of Veterinary Research* 64: S15-22.
248. Tavassoli M, Dalir-Naghadeh B, Valipour S, Maghsoudlo M (2018) Prevalence of gastrointestinal parasites in water buffalo (*Bubalus bubalis*) calves raised with cattle in smallholder farming system in the northwest of Iran. *Acta Veterinaria Eurasia* 44: 6-11.
249. Utaaker KS, Chaudhary S, Bajwa RS, Robertson LJ (2018) Prevalence and zoonotic potential of intestinal protozoans in bovines in Northern India. *Veterinary Parasitology: Regional Studies and Reports* 13: 92-97.
250. de Aquino MCC, Harvey TV, Inácio SV, Nagata WB, Ferrari ED, Oliveira BCM, Albuquerque GR, Widmer G, Meireles MV, Bresciani KDS (2019) First description of *Giardia duodenalis* in buffalo calves (*Bubalus* *bubalis*) in southwest region of São Paulo State, Brazil. *Food and Waterborne Parasitology* 16: e00062.
251. Russell S, Power M, Ens E (2020) *Cryptosporidium* and *Giardia* in feral water buffalo (*Bubalus bubalis*) in the South East Arnhem Land Indigenous Protected Area, Australia. *Parasitology Research* 119: 2149-2157.
252. Barburas DA, Cozma V, Ionică AM, Abbas I, Bărburaș R, Mircean V, D’Amico G, Dubey JP, Györke A (2022) Intestinal parasites of buffalo calves from Romania: molecular characterisation of *Cryptosporidium* spp. and *Giardia duodenalis*, and the first report of *Eimeria bareillyi. Folia* *Parasitologica* 69: 015.
253. Adhikari RB, Adhikari Dhakal M, Ghimire TR (2022) Prevalence and diversity of gastrointestinal parasites in domestic buffaloes (*Bubalus bubalis* Linnaeus, 1758) reared under captive and semi-captive conditions in Ratnanagar, Chitwan, Nepal. *Annals of Parasitology* 68: 701-713.
254. Kilinc OO, Ayan A, Çelik BA, Çelik OY, Yüksek N, Akyıldız G, Oguz FE (2023) The investigation of giardiasis (foodborne and waterborne diseases) in buffaloes in Van region, Türkiye: first molecular report of *Giardia duodenalis* assemblage B from buffaloes. *Pathogens* 12: 106.
255. Tokseiit Y, Albahadly WKY, Radhi H, Abduljabbar MH, Ahmed M, Alwash SW, Yousif ZS, Obeed AN, Saule J (2022) Determination of *Giardia duodenalis* (Metamonada: Hexamitidae) genotypes in water buffalo. *Caspian Journal of Environmetal Sciences* 21: 389-393.
256. Paltin AC, Mitrea E, Cerbu D, Ionita M, Mitrea IL (2023) Survey on intestinal parasites infections in water buffalo calves, in center Romania. *AgriLife Scientific Journal* 12: 134-139.
257. Sak B, Petrzelkova KJ, Kvetonova D, Mynarova A, Kathryn A, Shutt KA, Pomajbikova K, Kalousova B, Modry D, Benavides J, Todd A, Kvac M (2013) Long-term monitoring of *Microsporidia*, *Cryptosporidium* and *Giardia* infections in western lowland gorillas (*Gorilla gorilla gorilla*) at different stages of habituation in Dzanga Sangha protected areas, Central African Republic. *PLoS ONE* 8: e71840.
258. Matsubayashi M, Takami K, Kimata I, Nakanishi T, Tani H, Sasai K, Eiichiroh Baba E (2005) Survey of *Cryptosporidium* spp. And *Giardia* spp. infections in various animals at a zoo in Japan. *Journal of Zoo and Wildlife Medicine* 36: 331-335.
259. Dashti A, Koster PC, Bailo B, Sanchez de las Matas A, Habela MA, Rivero-Juarez A, Vicente J, Serrano E, Arnal MC, de Luco DF, Morrondo P, Armenteros JA, Balseiro A, Cardona GA, Martinez-Carrasco C, Ortiz JA, Carpio AJ, Calero-Bernal R, Gonzalez-Barrio D, David Carmena D (2023) Occurrence and limited zoonotic potential of *Cryptosporidium* spp., *Giardia duodenalis*, and *Balantioides coli* infections in free-ranging and farmed wild ungulates in Spain. *Research in Vetrinary Science* 159: 189-197.
260. Zhao GH, Du SZ, Wang HB, Hu XF, Deng MJ, Yu SK, Zhang LX, Zhu XQ (2015) First report of zoonotic *Cryptosporidium* spp., *Giardia intestinalis* and *Enterocytozoon bieneusi* in golden takins (*Budorcas taxicolor bedfordi*). *Infection, Genetics and Evolution* 34: 394-401.
261. Castro-Hermida JA, Delafosse A, Pors I, Ares-Mazás E, Chartier C (2005) *Giardia duodenalis* and *Cryptosporidium parvum* infections in adult goats and their implications for neonatal kids. *Veterinary Record* 157: 623-627
262. Ruiz A, Foronda P, Gonzalez JF, Guedes A, Abreu-Acosta N, Molina JM, Valladares B (2008) Occurrence and genotype characterization of *Giardia duodenalis* in goat kids from the Canary Islands, Spain. *Veterinary Parasitology* 154: 137-141.
263. Geurden T, Thomas P, Casaert S, Vercruysse J, Claerebout E (2008) Prevalence and molecular characterization of *Cryptosporidium* and *Giardia* in lambs and goat kids in Belgium. *Veterinary Parasitology* 155: 142-145.
264. Berrilli F, D’Alfonso R, Giangaspero A, Marangi M, Brandonisio O, Kabore Y, Gle C, Cianfanelli C, Lauro R, Di Cave D (2012) *Giardia duodenalis* genotypes and *Cryptosporidium* species in humans and domestic animals in Cote d’Ivoire: occurrence and evidence for environmental contamination. *Transactions of the Royal Society of Tropical Medicine and Hygiene* 106: 191-195.
265. Zhang W, Zhang X, Wang R, Liu A, Shen Y, Ling H, Cao J, Yang F, Zhang X, Zhang L (2012) Genetic characterizations of *Giardia duodenalis* in sheep and goats in Heilongjiang province, China and possibility of zoonotic transmission. *PLoS Neglected Tropical Diseases* 6: e1826.
266. Lim YAL, Mahdy MAK, Tan TK, Goh XT, Jex AR, Nolan MJ, Sharma RSK, Gasser RB (2013) First molecular characterization of *Giardia duodenalis* from goats in Malaysia. *Molecular and Cellular Probes* 27: 28-31.
267. Jafari H, Jalali MHR, Seyfi Abad Shapouri M, Haji Hajikolaii MR (2014) Determination of *Giardia duodenalis* genotypes in sheep and goat from Iran. *Journal of Parasitic Diseases* 38: 81-84.
268. Sudre AP, Leles D, Lima MF, Bomfim TCB (2014) First molecular characterisation of *Giardia duodenalis* infection in dairy goats in Brazil. *Veterinarni Medicina* 59: 283-292.
269. Gu YF, Wang LK, Li Y, Li L, Chu XH, Xin DW, Ma CX, Xu WH, Wu SB, Wang HY, Li WC (2014) Prevalence and molecular characterization of *Giardia lamblia* isolates from goats in Anhui province. *Zhongguo Ji Sheng Chong Xue Yu Ji Sheng Chong Bing Za Zhi* 32: 401-403 (in Chinese).
270. Hossain MA, Mina SA, Marzan LW, Emon MAIK, Das R, Siddiki AZ (2015) Molecular characterization of *Giardia intestinalis* assemblage E from goat kids in Bangladesh. *Asian Pacific Journal of Tropical Disease* 5: 374-379.
271. Akinkuotu OA, Okwelum N, Famakinde SA, Akinkuotu AC, Oseni OT (2016) *Giardia* infection in recently acclimatized kalahari red goats in Nigeria. *Nigerian Veterinary Journal* 37: 16-23.
272. Utaaker KS, Myhr N, Bajwa RS, Joshi H, Kumar A, Robertson LJ (2017) Goats in the city: prevalence of *Giardia duodenalis* and *Cryptosporidium* spp. in extensively reared goats in northern India. *Acta Veterinaria Scandinavica* 59: 86.
273. Xie SC, Zou Y, Chen D, Jiang MM, Yuan XD, Li Z, Zou FC, Yang JF, Sheng JL, Zhu XQ (2018) Occurrence and multilocus genotyping of *Giardia duodenalis* in yunnan black goats in China. *BioMed Research International* Article ID: 4601737.
274. Zhong Z, Tu R, Ou H, Yan G, Dan J, Xiao Q, Wang Y, Cao S, Shen L, Deng J, Zuo Z, Ma X, Zhou Z, Liu H, Yu S, Ren Z, Hu Y (2018) Occurrence and genetic characterization of *Giardia duodenalis* and *Cryptosporidium* spp. from adult goats in Sichuan Province, China. *PLoS ONE* 13: e0199325.
275. Chen D, Zou Y, Li Z, Wang SS, Xie SC, Shi LQ, Zou FC, Yang JF, Zhao GH, Zhu XQ (2019) Occurrence and multilocus genotyping of *Giardia duodenalis* in black‑boned sheep and goats in southwestern China. *Parasites & Vectors* 12: 102.
276. Chang Y, Yange Wang Y, Wu Y, Niu Z, Li J, Zhang S, Wang R, Jian F, Ning C, Zhang L (2019) Molecular characterization of *Giardia duodenalis* and *Enterocytozoon bieneusi* isolated from tibetan sheep and tibetan goats under natural grazing conditions in tibet. *Journal of Eukaryotic* *Microbiology* 67: 100-106.
277. Akinkuotu OA, Takeet MI, Otesile EB, Olufemi F, Greenwood SJ, McClure JT (2019) Multilocus genotyping and phylogenetic analyses of *Giardia intestinalis* isolates from indigenous goats in Ogun State, Nigeria. *Acta Tropica* 195: 15-22.
278. Faridi A, Tavakoli Kareshk A, Sadooghian S, Firouzeh N (2020) Frequency of different genotypes of *Giardia duodenalis* in slaughtered sheep and goat in east of iran. Journal of Parasitic Diseases 44: 618-624.
279. Wang P, Zheng L, Liu L, Yu F, Jian Y, Wang R, Zhang S, Zhang L, Ning C, Jian F (2022) Genotyping of *Cryptosporidium* spp., *Giardia duodenalis* and *Enterocytozoon bieneusi* from sheep and goats in China. *BMC Veterinary Research* 18: 361.
280. Terlumun IR, Nathaniel AL, Samuel AO (2022) Prevalence study of zoonotic gastrointestinal parasitic infections in goats in Makurdi metropolis, Nigeria. *Journal of Zoonotic Diseases* 6: 11-16.
281. Yang X, Wang J, Huang S, Song J, Fan Y, Zhao G (2023) Molecular characterization of *Cryptosporidium* spp., *Giardia duodenalis*, *Enterocytozoon bieneusi* and *Escherichia coli* in dairy goat kids with diarrhea in partial regions of Shaanxi province, China. *Animals* 13: 2922.
282. Yu X, Wang H, Li Y, Mu X, Yuan K, Wu A, Guo J, Hong Y, Zhang H (2023) Occurnce and genotypic identification of Blastocystis spp., Enterocytozoon bieneusi, and Giardia duodenalis in Leizhou black goats in Zhanjiang city, Guangdong province, China. *Animals* 13: 2777.
283. Kutz S, Thompson RCA, Polley L, Kandola K, Nagy J, Wielinga CM, Elkin BT (2008) *Giardia* assemblage A: human genotype in muskoxen in the Canadian Arctic. *Parasites & Vectors* 1: 32.
284. Berg RPKD, Stensvold CR, Jokelainen P, Grønlund AK, Nielsen HV, Kutz S, Kapel CMO (2021) Zoonotic pathogens in wild muskoxen (*Ovibos* *moschatus*) and domestic sheep (*Ovis aries*) from Greenland. Veterinary Medicine and Science 7: 1-13.
285. Ryan UM, Bath C, Robertson I, Read C, Elliot A, Mcinnes L, Traub R, Besier B (2005) Sheep may not be an important zoonotic reservoir for *Cryptosporidium* and *Giardia* parasites. *Applied and Environmental Microbiology* 71: 4992-4997.
286. Giangaspero A, Paoletti B, Iorio R, Traversa D (2005) Prevalence and molecular characterization of *Giardia duodenalis* from sheep in central Italy. *Parasitology Research* 96: 32-37.
287. Santín M, Trout JM, Fayer R (2007) Prevalence and molecular characterization of *Cryptosporidium* and *Giardia* species and genotypes in sheep in Maryland. *Veterinary Parasitology* 146: 17-24.
288. Ozdal N, Tanritanir P, Goz Yasar, Deger S, Kozat S (2009) Parasitic protozoans (*Eimeria*, *Giardia* and *Cryptosporidium*) in lambs with diarrhoea in the Van province (Turkey). *Bulletin of the Veterinary Institute Pulawy* 53: 47-51.
289. Yang R, Jacobson C, Cameron Gordon C, Ryan U (2009) Prevalence and molecular characterisation of *Cryptosporidium* and *Giardia* species in pre-weaned sheep in Australia. *Veterinary Parasitology* 161: 19-24.
290. Nolan MJ, Jex AR, Pangasa A, Young ND, Campbell AJ, Stevens M, Gasser RB (2010) Analysis of nucleotide variation within the triose-phosphate isomerase gene of *Giardia* *duodenalis* from sheep and its zoonotic implications. *Electrophoresis* 31: 287-298.
291. Gómez-Muñoz MT, Cámara-Badenes C, Martínez-Herrero MC, Dea-Ayuela MA, Pérez-Gracia MT, Fernández-Barredo S, Santín M, Fayer R (2012) Multilocus genotyping of *Giardia duodenalis* in lambs from Spain reveals a high heterogeneity. *Resrech in Veterinary Science* 93: 836-842.
292. Paz e Silva FM, Lopes RS, Bresciani KDS, Amarante AFT, Araujo Jr JP (2014) High occurrence of *Cryptosporidium ubiquitum* and *Giardia duodenalis* genotype E in sheep from Brazil. *Acta Parasitologica* 59: 193-196.
293. Ye J, Xiao L, Wang Y, Guo Y, Roellig DM, Feng Y (2015) Dominance of *Giardia duodenalis* assemblage A and *Enterocytozoon bieneusi* genotype BEB6 in sheep in Inner Mongolia, China. *Veterinary Parasitology* 210: 235-239.
294. Vasco K, Graham JP, Trueba G (2016) Detection of zoonotic enteropathogens in children and domestic animals in a semirural community in Ecuador. *Applied and Environmental Microbiology* 82: 4218-4224.
295. Wang H, Qi M, Zhang K, Li J, Huang J, Ning C, Zhang L (2016) Prevalence and genotyping of *Giardia duodenalis* isolated from sheep in Henan Province, central China. *Infections, Genetics and Evolution* 39: 330-335.
296. Wegayehu T, Karim MR, Li J, Adamu H, Erko B, Zhang L, Tilahun G (2017) Prevalence and genetic characterization of *Cryptosporidium* species and *Giardia duodenalis* in lambs in Oromia Special Zone, Central Ethiopia. *BMC Veterinary Research* 13: 22.
297. Wu Y, Chang Y, Chen Y, Zhang X, Li D, Zheng S, Wang L, Li J, Ning C, Zhang L (2018) Occurrence and molecular characterization of *Cryptosporidium* spp., *Giardia duodenalis*, and *Enterocytozoon bieneusi* from Tibetan sheep in Gansu, China. *Infections, Genetics and Evolution* 64: 46-51.
298. Qi M, Zhang Z, Zhao A, Jing B, Guan G, Luo J, Zhang L (2019) Distribution and molecular characterization of *Cryptosporidium* spp., *Giardia* *duodenalis*, and *Enterocytozoon bieneusi* amongst grazing adult sheep in Xinjiang, China. *Parasitology International* 71: 80-86.
299. Benhassine S, Baroudi D, Hakem A, Thomas M, Laatamna AM, Belkessa S, Feng Y, Roellig D, Chen X, Adjou KT, Xiao L (2020) Occurrence and molecular characterization of *Giardia* *duodenalis* in lambs in Djelfa, the central steppe of Algeria. *Parasitology Research* 119: 2965-2973.
300. Cao L, Han K, Wang L, Hasi S, Yu F, Cui Z, Hai Y, Zhai X, Zhang L (2020) Genetic characteristics of *Giardia duodenalis* from sheep in Inner Mongolia, China. *Parasite* 27: 60.
301. Peng JJ, Zou Y, Li ZX, Liang QL, Song HY, Li TS, Ma YY, Zhu XQ, Zhou DH (2020) Prevalence and multilocus genotyping of *Giardia duodenalis* in Tan sheep (*Ovis aries*) in northwestern China. *Parasitology International* 77: 102126.
302. Yang F, Ma L, Gou JM, Yao HZ, Ren M, Yang BK, Lin Q (2022) Seasonal distribution of *Cryptosporidium* spp., *Giardia duodenalis* and *Enterocytozoon bieneusi* in Tibetan sheep in Qinghai, China. *Parasites & Vectors* 15: 394.
303. Zhao Q, Lu C, Pei Z, Gong P, Li J, Jian F, Jing B, Qi M, Ning C (2023) *Giardia duodenalis* in Hu sheep: occurrence and environmental contamination on large-scale housing farms. *Parasite* 30: 2.
304. Celik OY, Celik BA, Ayan A, Kilinc OO, Ercan K, Selcuk MA, Ayan OO (2023) A microscopic and molecular survey of *Giardia duodenalis* in lambs in Siirt, Turkiye. *Dicle Universitesi Veteriner Fakultesi Dergisi* 16: 71-74.
305. De Liberato C, Berrilli F, Marangi M, Santoro M, Trogu T, Putignani L, Lanfranchi P, Ferretti F, D’Amelio S, Giangaspero A (2015) *Giardia duodenalis* in Alpine (*Rupicapra rupicapra rupicapra*) and Apennine (*Rupicapra pyrenaica ornata*) chamois. *Parasites & Vectors* 8: 650.
306. Wallis PM, Buchanan-Mappin JM, Faubert GM, Belosevic M (1984) Reservoirs of *Giardia* spp. in southwestern Alberta. *Journal of Wildlife* *Diseases* 20: 279-283.
307. Siefker C, Rickard LG, Pharr GT, Simmons JS, O'Hara TM (2002) Molecular characterization of *Cryptosporidium* sp. isolated from Northern Alaskan caribou (*Rangifer tarandus*). *Journal of Parasitology* 88: 213-216.
308. Hamnes IS, Gjerde B, Robertson L, Vikøren T, Handeland K (2006) Prevalence of *Cryptosporidium* and *Giardia* in free-ranging wild cervids in Norway. wild cervids in Norway. *Veterinary Parasitology* 141: 30-41.
309. Solarczyk P, Majewska AC, Moskwa B, Cabaj W, Dabert M, Nowosad P (2012) Multilocus genotyping of *Giardia duodenalis* isolates from red deer (*Cervus elaphus*) and roe deer (*Capreolus capreolus*) from Poland. *Folia Parasitologica* 59: 237-240.
310. Stojecki K, Sroka J, Caccio SM, Cencek T, Dutkiewicz J, Kusyk P (2015) Prevalence and molecular typing of *Giardia duodenalis* in wildlife from eastern Poland. *Folia Parasitologica* 62: 042.
311. Beck R, Sprong H, Lucinger S, Pozio E, Caccio SM (2011) A large survey of croatian wild mammals for *Giardia duodenalis* reveals a low prevalence and limited zoonotic potential. *Vector-Borne and Zoonotic Diseases* 11: 1049-1055.
312. Castro-Hermida JA, García-Presedo I, González-Warleta M, Mezo M (2011) Prevalence of *Cryptosporidium* and *Giardia* in roe deer (*Capreolus capreolus*) and wild boars (*Sus scrofa*) in Galicia (NW, Spain). *Veterinary Parasitology* 179: 216-219.
313. Garcia-Presedo I, Pedraza-Díaz S, González-Warleta M, Mezo M, Gómez-Bautista M, Ortega-Mora LM, Castro-Hermida JA (2013) The first report of *Cryptosporidium bovis*, *C. ryanae* and *Giardia duodenalis* sub-assemblage A-II in roe deer (*Capreolus capreolus*) in Spain. *Veterinary* *Parasitology* 197: 658-664.
314. Pacha RE, Clark GW, Williams EA, Carter AM, Scheffelmaier JJ, Debusschere P (1987) Small rodents and other mammals associated with mountain meadows as reservoirs of *Giardia* spp. and *Campylobacter* spp. *Applied and Environmental Microbiology* 53: 1574-1579.
315. Deng MQ, Cliver DO (1999) Improved immunouorescence assay for detection of *Giardia* and *Cryptosporidium* from asymptomatic adult cervine animals. *Parasitology* *Research* 85: 733-736.
316. Huang J, Zhang Z, Zhang Y, Yang Y, Zhao J, Wang R, Jian F, Ning C, Zhang W, Zhang L (2018) Prevalence and molecular characterization of *Cryptosporidium* spp. and *Giardia duodenalis* in deer in Henan and Jilin, China. *Parasites & Vectors* 11: 239.
317. Ren G, Li J, Xiong J, Lai X, Wang Y, Lei S, Lu X, He T, Zhou Y, Zhang Y, Lv G (2023) Molecular detection and public health risk assessment of *Cryptosporidium* spp., *Giardia duodenalis*, *Enterocytozoon bieneusi*, and *Blastocystis* sp. of animals in a tropical wildlife park of Hainan Island, China. *One Health Bulletin* 3: 1-10.
318. Yamazaki A, Izumiyama S, Yagita K, Kishida N, Kubosaki A, Hara-Kudo Y, Kamata Y, Terajima J (2018) The Molecular Detection of *Cryptosporidium* and *Giardia* in Sika Deer (*Cervus Nippon Centralis*) in Japan. *Food Safety* 6: 88-95.
319. Lalle M, Frangipane di Regalbono A, Poppi L, Nobili G, Tonanzi D, Pozio E, Cacciò SM (2007) A novel *Giardia duodenalis* assemblage A aubtype in fallow deer. *The Journal of Parasitology* 93: 426-428.
320. Chagas CRF, Gonzalez IHL, Salgado BAB, Rodrigues B, Ramos PL (2019) *Giardia* spp., ten years of parasitological data in the biggest zoo of Latin America. *Annals of Parasitology* 65: 35-51.
321. Song Y, Li W, Liu H, Zhong Z, Luo Y, Wei Y, Fu W, Ren Z, Zhou Z, Deng L, Cheng J, Peng G (2018) First report of *Giardia duodenalis* and *Enterocytozoon bieneusi* in forest musk deer (*Moschus berezovskii*) in China. *Parasites & Vectors* 11: 204.
322. Cui Z, Wang Q, Huang X, Bai J, Zhu B, Wang B, Guo X, Qi M, Li J (2022) Multilocus genotyping of *Giardia duodenalis* in Alpine musk deer (*Moschus* *chrysogaster*) in China. *Frontiers in Cellular and Infection Microbiology* 12: 856429.
323. Rickard LG, Siefker C, Boyle CR, Gentz EJ (1999) The prevalence of *Cryptosporidium* and *Giardia* spp. in fecal samples from free-ranging white-tailed deer (*Odocoileus virginianus*) in the southeastern United States. *Journal of Veterinary Diagnostic Investigation* 11: 65-72.
324. Trout JM, Santin M, Fayer R (2003) Identifification of assemblage A *Giardia* in white-tailed deer. Journal of Parasitology 89: 1254-1255.
325. Santin M, Fayer R (2015) *Enterocytozoon bieneusi*, *Giardia* and *Cryptosporidium* infecting white-tailed deer. *Journal of Eukaryotic Microbiology* 62: 34-43.
326. Johnson D, Harms NJ, Larter NC, Elkin BT, Tabel H, Wei G (2010) Serum biochemistry, serology, and parasitology of boreal caribou (*Rangifer tarandus caribou*) in the Northwest Territories, Canada. *Journal of Wildlife Diseases* 46: 1096-1107.
327. Van Hemert C, Ballweber LR, Sinnett DR, Atwood TC, Fischbach A, Gustine DD, Pabilonia KL (2023) *Giardia* and *Cryptosporidium* in resident wildlife species in Arctic Alaska. *Food and Waterborne Parasitology* 32: e00206.
328. Koehler AV, Haydon SR, Jex AR, Gasser RB (2016) *Cryptosporidium* and *Giardia* taxa in faecal samples from animals in catchments supplying the city of Melbourne with drinking water (2011 to 2015). *Parasites & Vectors* 9: 315.
329. Zhang Y, Mi R, Yang L, Gong H, Xu C, Feng Y, Chen X, Huang Y, Han X, Chen Z (2021) Wildlife is a potential source of human infections of *Enterocytozoon bieneusi* and *Giardia duodenalis* in southeastern China. *Frontiers in Microbiology* 12: 692837.
330. Atwill ER, Sweitzer RA, Pereira MDGC, Gardner IA, Vuren DV, Boyce WM (1997) Prevalence of and associated risk factors for shedding *Cryptosporidium parvum* oocysts and *Giardia* cysts within feral pig populations in California. *Applied and Environmental Microbiology* 63: 3946-3949.
331. Yaghoobi K, Sarkari B, Mansouri M, Motazedian MH (2016) Zoonotic intestinal protozoan of the wild boars, *Sus scrofa*, in Persian Gulf’s coastal area (Bushehr province), Southwestern Iran, *Veterinary World* 9: 1047-1050.
332. Rodriguez-Rivera LD, Cummings KJ, McNeely I, Suchodolski JS, Scorza AV, Lappin MR, Mesenbrink BT, Leland BR, Bodenchuk MJ (2016) Prevalence and diversity of *Cryptosporidium* and *Giardia* identified among feral pigs in Texas. *Vector-Borne and Zoonotic Diseases* 16: 765-768.
333. Li W, Deng L, Wu K, Huang X, Song Y, Su H, Hu Y, Fu H, Zhong Z, Peng G (2017) Presence of zoonotic *Cryptosporidium* *scrofarum*, *Giardia duodenalis* assemblage A and *Enterocytozoon bieneusi* genotypes in captive Eurasian wild boars (*Sus scrofa*) in China: potential for zoonotic transmission. *Parasites & Vectors* 10: 10.
334. Lee H, Kwak D (2023) Molecular detection and assemblage analysis of the intestinal protozoan *Giardia duodenalis* in wild boars in Korea. *Frontiers in Veterinary Science* 10: 1139060.
335. Marti-Marco A, Moratal S, Torres-Blas I, Cardells J, Lizana V, Dea-Ayuela MA (2023) Molecular detection of epidemiology of potentially zoonotic *Cryptosporidium* spp. and *Giardia duodenalis* in wild boar (*Sus scrofa*) from Eastern Spain. *Animals* 13: 2501.
336. Xiao L, Herd RP, Bowman GL (1994) Prevalence of *Cryptosporidium* and *Giardia* infections on two Ohio pig farms with different management systems. *Veterinary Parasitology* 52: 331-339.
337. Ryan UM, Samarasinghe B, Read C, Buddle JR, Robertson ID, Thompson RCA (2003) Identification of a novel *Cryptosporidium* genotype in pigs. *Applied and Environmental Microbiology* 69: 3970-3974.
338. Hamnes IS, Gjerde BK, Forberg T, Robertson LJ (2007) Occurrence of *Cryptosporidium* and *Giardia* in suckling piglets in Norway. *Veterinary* *Parasitology* 144: 222-233.
339. Armson A, Yang R, Thompson J, Johnson J, Reid S, Ryan UM (2009) *Giardia* genotypes in pigs in Western Australia: prevalence and association with diarrhea. *Experimental Parasitology* 121: 381-383.
340. Uysal HK, Boral O, Metiner K, Ilgaz A (2009) Investigation of intestinal parasites in pig feces that are also human pathogens. *Türkiye Parazitoloji* *Dergisi* 33: 218-221.
341. Farzan A, Parrington L, Coklin T, Cook A, Pintar K, Pollari F, Friendship R, Farber J, Dixon B (2011) Detection and characterization of *Giardia duodenalis* and *Cryptosporidium* spp. on swine farms in Ontario, Canada. *Foofborne Pathogens and Disease* 8: 1207-1213.
342. Budu-Amoako E, Greenwood SJ, Dixon BR, Barkema HW, Hurnik D, Estey C, McClure JT (2012) Occurrence of *Giardia* and *Cryptosporidium* in pigs on Prince Edward sland, Canada. *Veterinary Parasitology* 184: 18-24.
343. Siwila J, Mwape KE (2012) Prevalence of *Cryptosporidium* spp. and *Giardia duodenalis* in pigs in Lusaka, Zambia. *Onderstepoort Journal of Veterinary Research* 79: 5 pages.
344. Schär F, Inpankaew T, Traub RJ, Khieu V, Dalsgaard A, Chimnoi W, Chhoun C, Sok D, Marti H, Muth S, Odermatt P (2014) The prevalence and diversity of intestinal parasitic infections in humans and domestic animals in a rural Cambodian village. *Veterinary Parasitology* 63: 597-603.
345. Petersen HH, Jianmin W, Katakam KK, Mejer H, Thamsborg SM, Dalsgaard A, Olsen A, Enemark HL (2015) *Cryptosporidium* and *Giardia* in Danish organic pig farms: Seasonal and age-related variation in prevalence, infection intensity and species/genotypes. *Veterinary* *Parasitology* 214: 29-39.
346. Wang SS, Yuan YJ, Yin YL, Hu RS, Song JK, Zhao GH (2017) Prevalence and multilocus genotyping of *Giardia duodenalis* in pigs of Shaanxi Province, northwestern China. *Parasites & Vectors* 10: 490.
347. Jing B, Zhang Y, Xu C, Li D, Xing J, Tao D, Zhang L, Qi M, Wang H (2019) Detection and genetic characterization of *Giardia duodenalis* in pigs from large-scale farms in Xinjiang, China. *Parasite* 26: 53.
348. Liu H, Xu N, Yin J, Yuan Z, Shen Y, Cao J (2019) Prevalence and multilocus genotyping of potentially zoonotic *Giardia duodenalis* in pigs in Shanghai, China. *Parasitology* 146: 1199-1205.
349. Akinkuotu OA, Takeet MI, Otesile EB, Olufemi F, Greenwood SJ, McClure JT (2019) Prevalence and multilocus genotypes of *Giardia duodenalis* infecting pigs in Ogun state, Nigeria. *Infection, Genetics and Evolution* 70: 53-60.
350. Zhang HJ, Song JK, Wu XM, Li YH, Wang Y, Lin Q, Zhao GH (2019) First report of *Giardia duodenalis* genotypes in Zangxiang pigs from China. *Parasitology Research* 118: 2305-2310.
351. Gonzalez-Ramírez LC, Vazquez CJ, Chimbaina MB, Pablo Djabayan-Djibeyan P, Prato-Moreno JG, Trelis M, Fuentes MV (2021) Ocurrence of enteroparasites with zoonotic potential in animals of the rural area of San Andres, Chimborazo, Ecuador. *Veterinary Parasitology: Regional Studies and Reports* 26: 100630.
352. Zou Y, Yuan XD, Zhang SY, Zhang HY, Chen XQ (2021) Molecular detection and characterization of *Giardia duodenalis* in farmed pigs in three provinces of Southern China. *Pathogens* 10: 1481.
353. Adhikari RB, Adhikari Dhakal M, Thapa S, Ghimire TR (2021) Gastrointestinal parasites of indigenous pigs (*Sus domesticus*) in south-central Nepal. *Veterinary Medicine and Science* 7: 1820-1830.
354. Zhao FR, Zhang N, Miao WY, Wu R, Cui LL, Huang CQ, Zhou DH (2022) Molecular detection and multilocus genotyping of *Giardia duodenalis* in pigs in Fujian province, Southeastern China. *Animals* 12: 3148.
355. Li D, Deng H, Zheng Y, Zhang H, Wang S, He L, Zhao J (2022) First characterization and zoonotic potential of *Cryptosporidium* spp. and *Giardia duodenalis* in pigs in Hubei province of China. *Frontiers in Cellular and Infection Microbiology* 12:949773.
356. Ghebremichael ST, Meng X, Yang Y, Andegiorgish AK, Wu Z, Chen J, Wei J, Li T, Bao J, Zhou Z, Pan G (2023) First identofication and coinfection detection of *Enterocytozoon bieneusi*, *Encephalitozoon* spp., *Cryptosporidium* spp. and *Giardia duodenalis* in diarrheic pigs in southwest China. *BMC* *Microbiology* 23: 334.
357. Zhao SS, Li YH, Zhang Y, Zhou Q, Jing B, Xu CY, Zhang LX, Song JK, Qi M, Zhao GH (2020) Multilocus genotyping of *Giardia duodenalis* in Bactrian camels (*Camelus bactrianus*) in China. *Parasitology Research* 119: 3873-3880.
358. Locklear TR, Videla R, Breuer RM, Mulon PY, Passmore M, Mochel JP, Gerhold R, Schaefer JJ, Smith JS (2021) Presentation, clinical pathology abnormalities, and identification of gastrointestinal parasites in camels (*Camelus bactrianus* and *Camelus dromedarius*) presenting to two north american veterinary teaching hospitals. A Retrospective Study: 1980-2020. Frontiers in Veterinary Science 8: 651672.
359. Al-Jabr OA, Mohammed GE, Al-Hamdan BA (2005) Giardiosis in camels (*Camelus dromedarius*). *Veterinary Record* 157: 350-352.
360. Khedr EA, El‐Shanat SK, Fadly RS, Alsokkary MY, Otify YZ (2015) Studies on blood and enteric protozoans infecting camels at Behera province, Egypt. *Egyptan Veterinary Medical Society of Parasitology Journal* 11: 123‐130.
361. Hussin AG, Khalaf JM, Ali HM (2016) Detection of intestinal protozoa in camels and their breeders in Najef, Iraq. *Research Journal for Veterinary Practitioners* 3: 53-57.
362. Jawad HH, Jasim GA (2016) Molecular study of *Cryptosporidium* spp. and *Giardia lamblia* which cause diarrhea in camels (*Camillus dromedaries*) in Al-Diwaniyah and Al-Najaf provinces /Iraq. *AL-Qadisiyah Journal of Veterinary Medicine Sciences* 15: 70-75.
363. Bouragba M, Laatamna AK, Cheddad FE, Baroudi D, Houali K, Hakem A (2020) Gastrointestinal parasites of dromedary camel (*Camelus dromedarius*) in Algeria. *Veterinary World* 13: 1635-1640.
364. Hasan MH, Alani AAJ, Aghwan SS (2021) Investigations on gastrointestinal parasites in camels rearing in Nineveh Governorate. *Egyptian Journal of Veterinary Sciences* 52: 131-138.
365. Maxamhud S, Reghaissia N, Laatamna A, Samari H, Remdani N, Gentekaki E, Tsaousis AD (2023) Molecular identification of *Cryptosporidium* spp., and *Giardia duodenalis* in dromedary camels (*Camelus dromedarius*) from the Algerian Sahara. *Parasitologia* 3: 151-159.
366. Elmahallawy EK, Köster PC, Dashti A, Alghamdi SQ, Saleh A, Gareh A, Alrashdi BM, Hernández-Castro C, Bailo B, Lokman MS, Hassanen EAA, González-Barrio D, Carmena D (2023) Molecular detection and characterization of *Cryptosporidium* spp., *Giardia duodenalis*, and *Enterocytozoon* *bieneusi* infections in dromedary camels (*Camelus dromedaries*) in Egypt. *Frontiers in Veterinary Science* 10: 1139388.
367. Salama A, Noaman EA, Nayel M, El-Kattan AM, Mahmoud MA, Dawood AS, El-Hamid ISA, Elsify A, Zaghawa A, Arbaga AA, Mousa W (2023) Prevalence and molecular characterization of four enteric protozoa in dromrdary camels (*Camelus dromedarius*). *Alexandria Journal of Veterinary Sciences* 78: 17-27.
368. Rulofson FC, Atwill ER, Holmberg CA (2001) Fecal shedding of *Giardia duodenalis*, *Cryptosporidium parvum*, *Salmonella* organisms, and *Escherichia coli* O157:H7 from llamas in California. *American Journal of Veterinary Research* 62: 637-642.
369. Cebra CA, Mattson DE, Baker RJ, Sonn RJ, Dearing PL (2003) Potential pathogens in feces from unweaned llamas and alpacas with diarrhea. *Journal of the American Veterinary Medical Association* 223: 1806-1808.
370. Trout JM, Santın M, Fayer F (2008) Detection of Assemblage A, *Giardia duodenalis* and *Eimeria* spp. in alpacas on two Maryland farms. *Veterinary* *Parasitology* 153: 203-208.
371. Gómez-Couso H, Ortega-Mora LM, Aguado-Martínez A, Rosadio-Alcántara R, Maturrano-Hernández L, Espinoza LL, Zanabria-Huisa V, Pedraza-Díaz S (2012) Presence and molecular characterisation of *Giardia* and *Cryptosporidium* in alpacas (*Vicugna pacos*) from Peru. *Veterinary* *Parasitology* 187: 414-420.
372. Gomez-Puerta LA, Lopez-Urbina MT, Alarcon V, Cama V, Gonzalez AE, Xiao L (2014) Occurrence of *Giardia duodenalis* assemblages in alpacas in the Andean region. *Veterinary Parasitology* 63: 31-34.
373. Koehler AV, Rashid MH, Zhang Y, Vaughan JL, Gasser RB, Jabbar A (2018) First cross-sectional, molecular epidemiological survey of *Cryptosporidium*, *Giardia* and *Enterocytozoon* in alpaca (*Vicugna pacos*) in Australia. *Parasites & Vectors* 11: 498.
374. Olson ME, Roach PD, Stabler M, Chan W (1997) Giardiasis in ringed seals from the western arctic. *Journal of Wildlife Diseases* 33: 646-648.
375. Measures LN, Olson M (1999) Giardiasis in pinnipeds from eastern Canada. *Journal of Wildlife Diseases* 35: 779-782.
376. Hughes-Hanks JM, Rickard LG, Panuska C, Saucier JR, O'Hara TM, Dehn L, Rolland RM (2005) Prevalence of *Cryptosporidium* spp. and *Giardia* spp. in five marine mammal species. *Journal of Parasitology* 91: 1225-1228.
377. Reboredo-Fernández A, Ares-Mazás E, Martínez-Cedeira JA, Romero-Suances R, Cacciò SM, Gómez-Couso H (2014) *Giardia* and *Cryptosporidium* in cetaceans on the European Atlantic coast. *Parasitology Research* 114: 693-698.
378. Lasek-Nesselquist E, Welch DM, Sogin ML (2010) The identification of a new *Giardia duodenalis* assemblage in marine vertebrates and a preliminary analysis of *G. duodenalis* population biology in marine systems. *International Journal for Parasitology* 40: 1063-1074.
379. Reboredo-Fernández A, Gómez-Couso H, Martínez-Cedeira JA, Cacciò SM, Ares-Mazás E (2014) Detection and molecular characterization of *Giardia* and *Cryptosporidium* in common dolphins (*Delphinus delphis*) stranded along the Galician coast (Northwest Spain). *Veterinary* *Parasitology*. 202: 132-137.
380. Marangi M, Carlucci R, Carlino P, Fanizza C, Cirelli G, Maglietta R, Beneduce L (2022) Dolphins and sea turtles may host zoonotic parasites and pathogenic bacteria as indicators of anthropic pressure in the Gulf of Taranto (Northern Ionion Sea, Central-Eastern Mediterranean Sea). *Veterinary Research Communications* 46: 1157-1166.
381. Kleinertz S, Hermosilla C, Ziltener A, Kreicker S, Hirzmann J, Abdel-Ghaffar F, Taubert A (2014) Gastrointestinal parasites of free-living indo-pasific bottlenose dolphins (*Tursiops aduncus*) in the Northern Red Sea, Egypt. *Parasitology Research* 113: 1405-1415.
382. Hermosilla C, Hirzmann J, Silva LMR, Brotons JM, Cerda M, Prenger-Berninghoff E, Ewers C, Taubert A (2018) Occurrence of anthropozoonotic parasitic infections and faecal microbes in free-ranging sperm whales (*Physeter macrocephalus*) from the Mediterranean Sea. *Parasitology* *Research* 117: 2531-2541.
383. Hoskins JD, Malone JB, Smith PH, Uhl SA (1982) Prevalence of parasitism diagnosed by fecal examination in Louisiana dogs. *American Journal of Veterinary Research* 43: 1106-1109.
384. Burnie AG, Simpson JW, Lindsay D, Miles RS (1983) The excretion of *Campylobacter*, *Salmonella* and *Giardia lamblia* in the faeces of stray dogs. *Veterinary Research Communications* 6: 133-138.
385. Swan JM, Thompson RCA (1986) The prevalence of *Giardia* in dogs and cats in Perth, Western Australia. *Australian Veterinary Journal* 63: 110-112.
386. Collins GH, Pope SE, Griffin DL, Walker J, Connor G (1987) Diagnosis and prevalence of *Giardia* spp in dogs and cats. *Australian Veterinary* *Journal* 64: 89-90.
387. Kirkpatrick CL (1988) Epizootiology of endoparasitic infections in pet dogs and cats presented to a veterinary teaching hospital. *Veterinary* *Parasitology* 30: 113-124.
388. Hahn NE, Glaser CA, Hird DW, Hirsh DC (1988) Prevalence of *Giardia* in the feces of pups. *Journal of the American Veterinary Medical Association* 192: 1428-1429.
389. Brown TJ, Brown TJ, Ionas G (1991) *Giardia* infection of cats and dogs in New Zealand. *New Zealand Veterinary Journal* 39: 33-34.
390. Vanparijs O, Hermans L, van der Flaes L (1991) Helminth and protozoan parasites in dogs and cats in Belgium. *Veterinary Parasitology* 38: 67-73.
391. Epe C, Ising-Volmer S, Stoye M (1993) Parasitological fecal studies of equids, dogs, cats and hedgehogs during the years 1984-1991. *Deutsche Tierarztliche Wochenschrift* 100: 426-428. (in Germany).
392. Hopkins RM, Deplazes P, Meloni BP, Reynoldson JA, Thompson RCA (1993) A field and laboratory evaluation of a commercial ELISA for the detection of *Giardia* coproantigens in humans and dogs. *Transactions of the Royal Society of Tropical Medicine and Hygiene* 87: 39-41.
393. Savini G, Dunsmore JD, Robertson ID (1993) A survey of Western Australian dogs for *Sarcocystis* spp and other intestinal parasites. *Australian Veterinary Association* 70: 275-276.
394. Meloni BP, Thompson RC, Hopkins RM, Reynoldson JA, Gracey M (1993) The prevalence of *Giardia* and other intestinal parasites in children, dogs and cats from aboriginal communities in the Kimberley. *The Medical Journal of Australia* 158: 157-159.
395. Milstein TC, Goldsmid GM (1995) The presence of *Giardia* and other zoonotic parasites of urban dogs in Hobart, Tasmania. *Australian Veterinary Journal* 72: 154-155.
396. Nolan TJ, Smith G (1995) Time series analysis of the prevalence of endoparasitic infections in cats and dogs presented to a veterinary teaching hospital. *Veterinary Parasitology* 59: 87-96.
397. Blagburn BL, Lindsay DS, Vaughan JL, Rippey NS, Wrght JC, Lynn RC, Kelch WJ, Ritchie GC, Hepler DI (1996) Prevalence of canine parasites based on fecal flotation. *The Compendium on continuing education for the practicing veterinarian* 18: 483-509.
398. Coggins JR (1998) Effect of season, sex, and age on prevalence of parasitism in dogs from Southeastern Wisconsin. *Journal of the Helminthological Society of Washington* 65: 219-224.
399. Bugg RJ, Robertson ID, Eliot AD, Thompson RCA (1999) Gastrointestinal parasites of urban dogs in Perth, western Australia. *The Veterinary Journal* 157: 295-301.
400. Carollo MCC, Amato Neto V, Braz LMA, Kim D (2001) Detection of *Cyclospora* sp. oocysts in the faeces of stray dogs in Greater São Paulo,São Paulo State, Brazil. *Revista da Sociedade Brasileira de Medicina Tropical* 34: 597-598.
401. Itoh N, Muraoka N, Aoki M, Itagaki T (2001) Prevalence of *Giardia* *lamblia* infection in Ho dogs. *Kansenshogaku Zasshi* 75: 671-677.
402. Jacobs SR, Forrester CP, Yang J (2001) A survey of the prevalence of *Giardia* in dogs presented to Canadian veterinary practices. *The Canadian* *Veterinary Journal* 42: 45-46.
403. Mochizuki M, Hashimoto M, Ishida T (2001) Recent epidemiologicalstatus of canine viral enteric infections and *Giardia* infection in Japan. *The Journal of Veterinary Medical Science* 63: 573-575.
404. Nikolic A, Dimitrijevic S, Djurkovic-Djakovic O, Bobic B, Maksimovic-Mihajlovic O (2002) Giardiasis in dogs and cats in the Belgrade area. *Acta* *Veterinaria* 52: 43-47.
405. Oliveira-Sequeira TC, Amarante AF, Ferrari TB, Nunes LC (2002) Prevalence of intestinal parasites in dogs from Sao Paulo State, Brazil. *Veterinary* *Parasitology* 103: 19-27.
406. Rufino SM, Fernandes CGN, Moura ST, Grosz dLCB (2002) Descriptive epidemiological study of giardiasis in dogs attended at the Veterinary Hospital of the University of Cuiaba, Mato Grosso State. *Revista Brasileira de Medicina Veterinaria* 24: 198-202.
407. Zajac AM, Johnson J, King SE (2002) Evaluation of the importance ofcentrifugation as a component of zinc sulfate fecal flotation examinations. *Journal of the American Animal Hospital Association* 38: 221-224.
408. Barutzki D, Schaper R (2003) Endoparasites in dogs and cats in Germany 1999-2002. *Parasitology Research* 90: S148-S150.
409. Hackett T, Lappin MR (2003) Prevalence of enteric pathogens in dogs ofnorth-central Colorado. *Journal of the American Animal Hospital Association* 39: 52-56.
410. Mundim MJS, Souza SZ, Hortêncio SM, Cury MC (2003) Frequency of *Giardia* spp. shown by two diagnostic methods in faeces of dogs. *Arquivo Brasileiro de Medicina Veterinaria e Zootecnia* 55: 770-773.
411. Svobodova V (2003) Parasitic infections in an animal shelter. *Acta Veterinaria Brno* 72: 415-420.
412. Traub RJ, Robertson ID, Irwin P, Mencke N, Monis P, Thompson RC (2003) Humans, dogs and parasitic zoonoses - unravelling the relationships in a remote endemic community in northeast India usingmolecular tools. *Parasitology* *Research* 90: S156-S157.
413. Capelli G, Paoletti B, Iorio R, Frangipane di Regalbono A, Pietrobelli M, Bianciardi P, Giangaspero A (2003) Prevalence of *Giardia* spp. in dogs and humans in northern and central Italy. *Parasitilogy Research* 90: S154-S155.
414. Anderson KA, Brooks AS, Morrison AL, Reid-Smith RJ, Martin SW, Benn DM, Peregrine AS (2004) Impact of *Giardia* vaccination on asymptomatic *Giardia* infections in dogs at a research facility. *The Canadian Veterinary Journal* 45: 924-930.
415. Asano K, Suzuki K, Matsumoto T, Sakai T, Asano R (2004) Prevalenceof dogs with intestinal parasites in Tochigi, Japan in 1979, 1991 and 2002. *Veterinary Parasitology* 120:243-248.
416. Bianciardi R, Papini R, Giuliani G, Cardini G (2004) Prevalence of *Giardia* antigen in stool samples from dogs and cats. *Revue de Médecine Véterinaire* 155: 417-421.
417. Cirak VY, Bauer C (2004) Comparison of conventional coproscopicalmethods and commercial coproantigen ELISA kits for the detection of *Giardia* and *Cryptosporidium* infections in dogs and cats. *Berliner Munchener Tierarztliche Wochenschrift* 117: 410-413.
418. Ramirez-Barrios RA, Barboza-Mena G, Muñoz J, Angulo-Cubillán F, Hernández E, González F, Escalona F (2004) Prevalence of intestinal parasites in dogs under veterinary care in Maracaibo, Venezuela. *Veterinary Parasitology* 121: 11-20.
419. Alves ODF, Gomes AG, Silva ACD (2005) Occurrence of endoparasites in dogs from Goiânia county, Goiás: comparison of diagnostictechniques. *Ciência Animal Brasileira* 6: 127-133.
420. Gundluch JL, Sadzikowski AB, Stepien-Rukasz H, Studzinska MB, Tomczuk K (2005) Comparison of some serological methods and coproscopic examinations for diagnosis of *Giardia* spp. invasion in dogs. *Polish Journal of Veterinary Sciences* 8: 137-140.
421. Huber F, Bomfim TCB, Gomes RS (2005) Comparison between natural infection by *Cryptosporidium* sp., *Giardia* sp. in dogs in two living situations in the West Zone of the municipality of Rio de Janeiro .*Veterinary Parasitology* 130: 69-72.
422. Itoh N, Muraoka N, Saeki H, Aoki M, Itagaki T (2005) Prevalence of *Giardia intestinalis* infection in dogs of breeding kennels in Japan. *The Journal of Veterinary Medical Science* 67: 717-718.
423. Itoh N, Muraoka N, Kawamata J, Aoki M, Itagaki T (2005) Detection of *Giardia* antigen in puppies using enzyme-linked immunosorbent assay. *Journal of the Japan Veterinary Medical Association* 57: 579-582. (in Japanese)
424. Papini R, Gorini G, Spaziani A, Cardini G (2005) Survey on giardiosis in shelter dog populations. *Veterinary Parasitology* 128: 333-339.
425. Ponce-Macotela M, Peralta-Abarca GE, Martinez-Gordillo MN (2005) *Giardia intestinalis* and other zoonotic parasites: prevalence in adult dogs from the southern part of Mexico City. *Veterinary Parasitology* 131: 1-4.
426. Sokolow SH, Rand C, Marks SL, Drazenovich NL, Kather EJ, Foley JE (2005) Epidemiologic evaluation of diarrhea in dogs in an animal shelter. *American Journal of Veterinary Research* 66: 1018-1024.
427. Arguedas Zeledón D, Bitter E, Oliveira J, Romero DJJ (2006) Prevalenceof *Toxocara canis* and other gastrointestinal parasites in dogs treatedat a veterinary clinic in San José, Costa Rica. *Ciencias Veterinarias* 24: 137-150.
428. Capelli G, Frangipane di Regalbono A, Iorio R, Pietrobelli M, Paoletti B, Giangaspero A (2006) *Giardia* species and other intestinal parasites in dogs in northeast and central Italy. *Veterinary Record* 159: 422-424.
429. Carlin EP, Bowman DD, Scarlett JM, Garrett J, Lorentzen L (2006) Prevalence of *Giardia* in symptomatic dogs and cats through out the United States as determined by the IDEXXSNAP *Giardia* test. *Veterinary Therapeutics* 7: 199-206.
430. Fontanarrosa MF, Vezzani D, Basabe J, Eiras DF (2006) An epidemiological study of gastrointestinal parasites of dogs from Southern Greater Buenos Aires (Argentina): age, gender, breed, mixed infections, and seasonal and spatial patterns. *Veterinary Parasitology* 136: 283-295.
431. Labruna MB, Pena HFJ, Souza SLP, Pinter A, Silva JCR, Ragozo AMA, Camargo LMA, Gennari SM (2006) Prevalence of endoparasites in dogs from the urban area of Monte Negro Municipality,Rondônia, Brazil. *Arquivos do Instituto Biologico* 73: 183-193.
432. Lefebvre SL, Waltner-Toews D, Peregrine AS, Reid-Smith R, Hodge L, Arroyo LG, Weese JS (2006) Prevalence of zoonotic agents in dogs visiting hospitalized people in Ontario: implications for infectioncontrol. *Journal of Hospital Infection* 62: 458-466.
433. Lopez J, Abarca K, Paredes P, Inzunza E (2006) Intestinal parasites in dogs and cats with gastrointestinal symptoms in Santiago, Chile. *Revista Medica de Chile* 134: 193-200.
434. Shukla R, Giraldo P, Kraliz A, Finnigan M, Sanchez AL (2006) *Cryptosporidium* spp. and other zoonotic enteric parasites in a sample of domestic dogs and cats in the Niagara region of Ontario. *The Canadian Veterinary Journal* 47: 1179-1184.
435. Zygner W, Jaros D, Skowrońska M, Bogdanowicz-Kamirska M, Wędrychowicz H (2006) Prevalence of *Giardia intestinalis* in domestic dogs in Warsaw. *Wiadomosci Parazytologiczne* 52: 311-315. (in Polish)
436. Dubna S, Langrova I, Napravnik J, Jankovska I, Vadlejch J, Pekar S, Fechtner J (2007) The prevalence of intestinal parasites in dogs from Prague, rural areas, and shelters of the Czech Republic. *Veterinary* *Parasitology* 145: 120-128.
437. Guest CM, Stephen JM, Price CJ (2007) Prevalence of *Campylobacter* and four endoparasites in dog populations associated with hearing dogs. *Journal of Small Animal Practice* 48: 632-637.
438. Hamnes IS, Gjerde BK, Robertson LJ (2007) A longitudinal study on the occurrence of *Cryptosporidium* and *Giardia* in dogs during their first year of life. *Acta Veterinaria Scandinavica* 49:22.
439. Inpankaew T, Traub R, Thompson RC, Sukthana Y (2007) Canine parasitic zoonoses in Bangkok temples. *The Southeast Asian Journal of Tropical Medicine and Public Health* 38: 247-255.
440. Lorenzini G, Tasca T, Carli Gad (2007) Prevalence of intestinal parasitesin dogs and cats under veterinary care in Porto Alegre, Rio Grande doSul, Brazil. *Brazilian Journal of Veterinary Research and Animal Science* 44: 137-145.
441. Martinez-Moreno FJ, Hernandez S, Lopez-Cobos E, Becerra C, Acosta I, Martinez-Moreno A (2007) Estimation of canine intestinal parasitesin Cordoba (Spain) and their risk to public health. *Veterinary* *Parasitology* 143: 7-13.
442. Martinez-Carrasco C, Berriatua E, Garijo M, Martinez J, Alonso FD, de Ybanez RR (2007) Epidemiological study of non-systemic parasitismin dogs in southeast mediterranean Spain assessed by coprologicaland post-mortem examination. *Zoonoses and Public Health* 54: 195-203.
443. Miro G, Mateo M, Montoya A, Vela E, Calonge R (2007) Survey of intestinal parasites in stray dogs in the Madrid area and comparison ofthe efficacy of three anthelmintics in naturally infected dogs. *Parasitology Research* 100: 317-320.
444. Mundim MJ, Rosa LA, Hortencio SM, Faria ES, Rodrigues RM, Cury MC (2007) Prevalence of *Giardia duodenalis* and *Cryptosporidium* spp. in dogs from different living conditions in Uberlandia, Brazil. *Veterinary Parasitology* 144: 356-359.
445. Papazahariadou M, Founta A, Papadopoulos E, Chliounakis S, Antoniadou-Sotiriadou K, Theodorides Y (2007) Gastrointestinal parasites of shepherd and hunting dogs in the Serres Prefecture, Northern Greece. *Veterinary Parasitology* 148: 170-173.
446. Pinto LD, Marques SMT, Bigatti LE, Araujo FAPd (2007) Enteroparasites in dogs: prevalence and owners’ knowledge about epidemiological factors. *Veterinaria em foco* 5: 10-15.
447. Rimhanen-Finne R, Enemark HL, Kolehmainen J, Toropainen P, Han-ninen ML (2007) Evaluation of immunofluorescence microscopy andenzyme-linked immunosorbent assay in detection of *Cryptosporidium* and *Giardia* infections in asymptomatic dogs. *Veterinary Parasitology* 145: 345-348.
448. Santos FAGd, Yamamura MH, Vidotto O., Camargo PLd (2007) Occurrence of gastrointestinal parasites in dogs (*Canis familiaris*) with acutediarrhea from metropolitan region of Londrina, Paraná State, Brazil. *Semina Ciencias Agrarias* 28: 257-268.
449. Szabová E, Juris P, Miterpáková M, Antolová D, Papajová I, Sefcíková H (2007) Prevalence of important zoonotic parasitesin dog populations from the Slovak Republic. *Helminthologia* 44: 170-176.
450. Szénási Z, Marton S, Kucsera I, Tánczos B, Horváth K, Orosz E, Lukács Z, Szeidemann Z (2007) Preliminary investigation of the prevalenceand genotype distribution of *Giardia intestinalis* in dogs in Hungary. *Parasitology Research* 101: S145-S152.
451. Volotao AC, Costa-Macedo LM, Haddad FSM, Brandão A, Peralta JM, Fernandes O (2007) Genotyping of *Giardia duodenalis* from human and animal samples from Brazil using ꞵ-giardin gene: a phylogeneticanalysis. *Acta Tropica* 102: 10-19.
452. Funada MR., Pena HFJ., Soares RM., Amaku M., Gennari SM., Frequencia de parasitos gastrintestinais em caes e gatos atendidos em hospital-escola veterinario da cidade de Sao Paulo. *Arquivo Brasileiro de Medicina Veterinaria e Zootecnia* 59: 1338-1340.
453. Batchelor DJ, Tzannes S, Graham PA, Wastling JM, Pinchbeck GL, German AJ (2008) Detection of endoparasites with zoonotic potentialin dogs with gastrointestinal disease in the UK. *Transboundary and Emerging Diseases* 55: 99-104.
454. Geurden T, Berkvens D, Casaert S, Vercruysse J, Claerebout E (2008) A Bayesian evaluation of three diagnostic assays for the detection of *Giardia duodenalis* in symptomatic and asymptomatic dogs. *Veterinary Parasitology* 157: 14-20.
455. Jafari-Shoorijeh S, Sadjjadi SM, Asheri A, Eraghi K (2008) *Giardia* spp.and Sarcocystis spp. status in pet dogs of Shiraz, Southern part of Iran. *Tropical Biomedicine* 25: 154-159.
456. Katagiri S, Oliveira-Sequeira TC (2008) Prevalence of dog intestinal parasites and risk perception of zoonotic infection by dog owners in Sao Paulo State, Brazil. *Zoonoses and Public Health* 55: 406-413.
457. Labarthe N, Mendes-De-Almeida F, Balbi M, Salomão M, Paiva J, Crissiuma AL, Garcia R, Miranda dCNCM (2008) Prevalence of *Giardia* in Ho dogs and cats in the State of Rio de Janeirousing the IDEXX SNAP®*Giardia* test. *International Journal of Applied Research in Veterinary Medicine* 6: 200-206
458. Liu J, Lee SE, Song KH (2008) Prevalence of canine giardiosis in SouthKorea. *Research in Veterinary Science* 84: 416-418.
459. Meireles P, Montiani-Ferreira F, Thomaz-Soccol V (2008) Survey of giardiosis in Ho and shelter dogs from metropolitan areas ofCuritiba, Parana state, Southern Brazil. *Veterinary Parasitology* 152: 242-248.
460. Nikolic A, Dimitrijevic S, Katic-Radivojevic S, Klun I, Bobic B, Djurkovic-Djakovic O (2008) High prevalence of intestinal zoonotic parasites in dogs from Belgrade, Serbia. *Acta Veterinaria Hungarica* 56: 335-340.
461. Palmer CS, Thompson RC, Traub RJ, Rees R, Robertson ID (2008) National study of the gastrointestinal parasites of dogs and cats in Australia. *Veterinary Parasitology* 151: 181-190.
462. Paoletti B, Iorio R, Capelli G, Sparagano OAE, Giangaspero A (2008) Epidemiological scenario of giardiosis in dogs from central Italy. *Animal* *Biodiversity and Emerging Diseases* 1149: 371-374.
463. Rinaldi L, Maurelli MP, Musella V, Veneziano V, Carbone S, Di Sarno A, Paone M, Cringoli G (2008) *Giardia* and *Cryptosporidium* in canine faecal samples contaminating an urban area. *Research in Veterinary Science* 84: 413-4.
464. Claerebout E, Casaert S, Dalemans AC, DeWilde N, Levecke B, Vercruysse J, Geurden T (2009) *Giardia* and other intestinal parasites in different dog populations in Northern Belgium. *Veterinary Parasitology* 161: 41-46.
465. Coman S, Dida IC, Bacescu B (2009) Incidence and treatment of the diarrhoeic syndrome with parasite aetiology in dogs and cats. *Scientica* *Parasitologica* 10: 106-111.
466. Gates MC, Nolan TJ (2009) Endoparasite prevalence and recurrence across different groups of dogs and cats. *Veterinary Parasitology* 166: 153-158.
467. Gracenea M, Gómez MS, Torres J (2009) Prevalence of intestinal parasites in shelter dogs and cats in the metropolitan area of Barcelona (Spain). *Acta Parasitolgica* 54: 73-77.
468. Itoh N, Kanai K, Hori Y, Hoshi F, Higuchi S (2009) Prevalence of *Giardia intestinalis* and other zoonotic intestinal parasites in private house-hold dogs of the Hachinohe area in Aomori prefecture, Japanin 1997, 2002 and 2007. *Journal of Veterinary Science* 10: 305-308.
469. Little SE, Johnson EM, Lewis D, Jaklitsch RP, Payton ME, Blagburn BL, Bowman DD, Moroff S, Tams T, Rich L, Aucoin D (2009) Prevalence of intestinal parasites in pet dogs in the United States. *Veterinary* *Parasitology* 166: 144-152.
470. Overgaauw PAM, van Zutphen L, Hoek D, Yaya FO, Roelfsema J, Pinelli E, van Knapen F, Kortbeek LM (2009) Zoonotic parasites infecal samples and fur from dogs and cats in The Netherlands. *Veterinary Parasitology* 163: 115-122.
471. Papini R, Marangi M, Mancianti F, Giangaspero A (2009) Occurrenceand cyst burden of *Giardia* *duodenalis* in dog faecal deposits from urban green areas: implications for environmental contamination andrelated risks. *Preventive Veterinary Medicine* 92: 158-162.
472. Prates L, Pacheco LS, Kuhl JB, Dias MLGG, Araújo SM, Pupulin ART (2009) Frequency of intestinal parasites in domiciled dogs fromMaringá city, Brazil. *Arquivo Brasileiro de Medicina Veterinaria Zootecnia* 61: 1468-1470.
473. Razmi GR (2009) Survey of dogs’ parasites in Khorasan Razavi Province, Iran. *Iranian Journal of Parasitology* 4: 48-54.
474. Scaramozzino P, Di Cave D, Berrilli F, D’Orazi C, Spaziani A, Mazzanti S, Scholl F, De Liberato C (2009) A study of the prevalence and genotypes of *Giardia duodenalis* infecting kennelled dogs. *The Veterinary Journal* 182: 231-234.
475. Traub RJ, Inpankaew T, Reid SA, Sutthikornchai C, Sukthana Y, Robertson ID, Thompson RCA (2009) Transmission cycles of *Giardia duodenalis* in dogs and humans in temple communities in Bangkok - a critical evaluation of its prevalence using three diagnostic tests in the field in the absence of a gold standard. *Acta Tropica* 111: 125-132.
476. Epe C, Rehkter G, Schnieder T, Lorentzen L, Kreienbrock L (2010) *Giardia* in symptomatic dogs and cats in Europe - results of a European study. *Veterinary Parasitology* 173: 32-38.
477. Gingrich EN, Scorza AV, Clifford EL, Olea-Popelka FJ, Lappin MR (2010) Intestinal parasites of dogs on the Galapagos Islands. *Veterinary* *Parasitology* 169: 404-407.
478. Himsworth CG, Skinner S, Chaban B, Jenkins E, Wagner BA, Harms NJ, Leighton FA, Thompson RC, Hill JE (2010) Multiple zoonotic pathogens identified in canine feces collected from a remote Canadian indigenous community. *American Journal of Tropical Medicine and Hygiene* 83: 338-341
479. Jiménez-Cardoso E, Eligio-García L, Cortés-Campos A, Estrada AC, Pinto-Sagahón M, Noguera-Estrada C (2010) The frequency of intestinal parasites in puppies from Mexican kennels. *Health* 2: 1316-1319.
480. Klimpel S, Heukelbach J, Pothmann D, Ruckert S (2010) Gastrointestinal and ectoparasites from urban stray dogs in Fortaleza (Brazil): highinfection risk for humans? *Parasitology* *Research* 107: 713-719.
481. Marangi M, Berrilli F, Otranto D, Giangaspero A (2010) Genotyping of *Giardia duodenalis* among children and dogs in a closed society deprived community from Italy. *Zoonoses and Public Health* 57: e54-8.
482. McKenzie E, Riehl J, Banse H, Kass PH, Nelson S, Marks SL (2010) Prevalence of diarrhea and enteropathogens in racing sled dogs. *Journal of Veterinary Internal Medicine* 24: 97-103.
483. Mirzaei M (2010) Prevalence of stray dogs with intestinal protozoan parasites. *American Journal of Animal Veterinary Sciences* 5: 86-90.
484. Mosallanejad B, Avizeh R, Jalali MHR, Alborzi AR (2010) Antigenic detection of *Giardia duodenalis* in companion dogs of Ahvaz area, south-west of Iran. *Jundishapur Journal of Microbiology* 3: 187-193.
485. Mukaratirwa S, Singh VP (2010) Prevalence of gastrointestinal parasites of stray dogs impounded by the Society for the Prevention of Cruelty to Animals (SPCA), Durban and Coast, South Africa. *Journal of South African Veterinary Association* 81: 123-125.
486. Olson ME, Leonard NJ, Strout J (2010) Prevalence and diagnosis of *Giardia* infection in dogs and cats using a fecal antigen test and fecal smear. *The Canadian Veterinary Journal* 51: 640-642.
487. Solarczyk P, Majewska AC (2010) A survey of the prevalence and genotypes of *Giardia duodenalis* infecting Ho and sheltered dogs. *Parasitology Research* 106: 1015-1019.
488. Soriano SV, Pierangeli NB, Roccia I, Bergagna HF, Lazzarini LE, Celescinco A, Saiz MS, Kossman A, Contreras PA, Arias C, Basualdo JA (2010) A wide diversity of zoonotic intestinal parasites infects urban and rural dogs in Neuquen, Patagonia, Argentina. *Veterinary Parasitology* 167: 81-85.
489. Upjohn M, Cobb C, Monger J, Geurden T, Claerebout E, Fox M (2010) Prevalence, molecular typing and risk factor analysis for *Giardia duodenalis* infections in dogs in a central London rescue shelter. *Veterinary Parasitology* 172: 341-346.
490. Yoshiuchi R, Matsubayashi M, Kimata I, Furuya M, Tani H, Sasai K (2010) Survey and molecular characterization of *Cryptosporidium* and *Giardia* spp. in owned companion animal, dogs and cats, in Japan. *Veterinary Parasitology* 174: 313-316.
491. Amfim A, Pârvu M, Bacescu B, Simion VE (2011) Estimation of canine intestinal parasites in Bucharest and their risk to public health. Bull. *UASVM Veterinary Medicine* 68: 26-30.
492. Bahrami A, Doosti A, Nahravanian H, Noorian AM, Ahmadi Asbchin S (2011) Epidemiological survey of gastrointestinal parasites in stray dogs and cats. *Australian Journal of Basic and Applied Sciences* 5: 1944-1948.
493. Bajer A, Bednarska M, Rodo A (2011) Risk factors and control of intestinal parasite infections in sled dogs in Poland. *Veterinary Parasitology* 175: 343-350.
494. Barutzki D, Schaper R (2011) Results of parasitological examinations of faecal samples from cats and dogs in Germany between 2003 and 2010. *Parasitology Research* 109: S45-S60.
495. Bryan HM, Darimont CT, Paquet PC, Ellis JA, Goji N, Gouix M, Smits JE (2011) Exposure to infectious agents in dogs in remote coastal British Columbia: possible sentinels of diseases in wildlife and humans. *Canadian Journal of Veterinary Research* 75: 11-17.
496. Covacin C, Aucoin DP, Elliot A, Thompson RCA (2011) Genotypic characterisation of *Giardia* from domestic dogs in the USA. *Veterinary* *Parasitology* 177: 28-32.
497. Ferreira FS, Pereira-Baltasar P, Parreira R, Padre L, Vilhena M, Tavora Tavira L, Atouguia J, Centeno-Lima S (2011) Intestinal parasites in dogs and cats from the district of Evora, Portugal. *Veterinary Parasitology* 179: 242-245.
498. Itoh N, Itagaki T, Kawabata T, Konaka T, Muraoka N, Saeki H, Kanai K, Chikazawa S, Hori Y, Hoshi F, Higuchi S (2011) Prevalence of intestinal parasites and genotyping of *Giardia intestinalis* in pet shop puppies in east Japan. *Veterinary* *Parasitology* 176: 74-78.
499. Itoh N, Kanai K, Tominaga H, Kawamata J, Kaneshima T, Chikazawa S, Hori Y, Hoshi F, Higuchi S (2011) *Giardia* and other intestinal parasites in dogs from veterinary clinics in Japan. *Parasitology* *Research* 109: 253-25.
500. Joffe D, Van Niekerk D, Gagne F, Gilleard J, Kutz S, Lobingier R (2011) The prevalence of intestinal parasites in dogs and cats in Calgary, Alberta. *The Canadian Veterinary Journal* 52: 1323-1328.
501. Lavallen CM, Dopchiz MC, Lobianco E, Hollmann P, Denegri G (2011) Intestinal parasites of zoonotic importance in dogs from the districtof general Pueyrredón (Buenos Aires, Argentina). *Revista Veterinaria* 22: 19-24.
502. Ortuno A, Castellà J (2011) Intestinal parasites in shelter dogs and risk factors associated with the facility and its management. *Israel Journal of Veterinary Medicine* 66: 103-107.
503. Scorza AV, Duncan C, Miles L, Lappin MR (2011) Prevalence of selected zoonotic and vector-borne agents in dogs and cats in Costa Rica. *Veterinary Parasitology* 183: 178-183.
504. Becker AC, Rohen M, Epe C, Schnieder T (2012) Prevalence of endoparasites in stray and fostered dogs and cats in Northern Germany. *Parasitology Research* 111: 849-857.
505. Dado D, Montoya A, Blanco MA, Miró G, Saugar JM, Bailo B, Fuentes I (2012) Prevalence and genotypes of *Giardia duodenalis* from dogs in Spain: possible zoonotic transmission and public health importance. *Parasitology Research* 111: 2419-2422.
506. Li J, Zhang P, Wang P, Alsarakibi M, Zhu H, Liu Y, Meng X, Li J, Guo J, Li G (2012) Genotype identification and prevalence of *Giardia duodenalis* in pet dogs of Guangzhou, southern China. *Veterinary Parasitology* 188: 368-371.
507. Mahmuda A, Magaji AA, Yakubu Y, Salihu MD, Lawal MD, Mahmud U, N. Suleiman N, Danmaigoro N (2012) Prevalence of intestinal parasites of dogs slaughtered at Mami market area, Sokoto, Nigeria. *Scientific Journal of Animal Science* 1: 126-130.
508. Mircean V, Gyorke A, Cozma V (2012) Prevalence and risk factors of *Giardia duodenalis* in dogs from Romania. *Veterinary Parasitology* 184: 325-329.
509. Pablo JO, Chávez V, Suárez AA, Pinedo FV, Falcón RPN (2012) *Giardia* spp. in dogs and children in rural communities of three districts of Puno, Peru. *Revista de Investigaciones Veterinarias del Perú* 23: 462-468.
510. Paz e Silva FM, Monobe MM, Lopes RS, Araujo Jr. JP (2012) Molecularcharacterization of *Giardia duodenalis* in dogs from Brazil. *Parasitology* *Research* 110: 325-33.
511. Schurer JM, Hill JE, Fernando C, Jenkins EJ (2012) Sentinel surveillancefor zoonotic parasites in companion animals in indigenous communities of Saskatchewan. *American Journal of Tropical Medicine and Hygiene* 87: 495-498.
512. Tupler T, Levy JK, Sabshin SJ, Tucker SJ, Greiner EC, Leutenegger CM (2012) Enteropathogens identified in dogs entering a Florida animal shelter with normal feces or diarrhea. *Journal of the American Veterinary Medical Association* 241: 338-343.
513. Wang A, Ruch-Gallie R, Scorza V, Lin P, Lappin MR (2012) Prevalenceof *Giardia* and *Cryptosporidium* species in dog park attending dogs compared to non-dog park attending dogs in one region of Colorado. *Veterinary Parasitology* 184: 335-340.
514. Farias A, Silva dNS, Oliveira MD, Rocha JBS, Santos LBKRD (2013) Diagnosis of gastrointestinal parasites in dogs from Bom Jesus, Piaui, Brazil. *Revista Academica Ciências Agrarias e Ambientais* 11: 431-435.
515. Li W, Liu C, Yu Y, Li J, Gong P, Song M, Xiao L, Zhang X (2013) Molecular characterization of *Giardia* *duodenalis* isolates from police and farm dogs in China. *Experimental Parasitology* 135: 223-226.
516. Mark-Carew MP, Adesiyun AA, Basu A, Georges KA, Pierre T, Tilitz S, Wade SE, Mohammed HO (2013) Characterization of *Giardia duodenalis* infections in dogs in Trinidad and Tobago. *Veterinary Parasitology* 196: 199-202.
517. Mohamed AS, Glickman LT, Camp Jr. JW, Lund E, Moore GE (2013) Prevalence and risk factors for *Giardia* spp. infection in a large national sample of pet dogs visiting veterinary hospitals in the United States (2003-2009). *Veterinary Parasitology* 195: 35-41.
518. Nolan MJ, Jex AR, Koehler AV, Haydon SR, Stevens MA, Gasser RB (2013) Molecular bades investigation of *Cryptosporidium* and *Giardia* from animals in water catchments in southeastern Australia. *Water Research* 47: 1726-1740.
519. Perfetti DC, Moreno PM (2013) Intestinal parasites of zoonotic importance in domiciliary canines of a rural village from Falcón state, Venezuela. *Boletin de Malariologia y Salud Ambiental* LIII: 19-28.
520. Quadros RM, Weiss PHE, Ezequiel GW, Tamanho RB, Lepo G, Silva MR, Silva Junior dCRJ, Araujo dFAP, Miletti dLC (2013) Prevalence of *Giardia* *duodenalis* among dogs seized by the Center for Control of Zoonoses (CCZ) of the city of Lages, Santa Catarina, Brazil. *Health* 5: 119-124.
521. Riggio F, Mannella R, Ariti G, Perrucci S (2013) Intestinal and lung parasites in owned dogs and cats from central Italy. *Veterinary Parasitology* 193: 78-84.
522. Shikha S, Katoch R, Anish Y, Rajesh G (2013) *Giardia* prevalence in pet dogs in Jammu, India. *Veterinary Practitioner* 14: 181-182.
523. Sotelo PH, Chávez V, Casas AA, Pinedo EV, Falcón RPN (2013) Giardiasis and Cryptosporidiosis in dogs of the western area of Metropolitan Lima. *Revista de Investigaciones Veterinarias del Perú* 24: 353-359.
524. Uehlinger FD, Greenwood SJ, McClure JT, Conboy G, O’Handley R, Barkema HW (2013) Zoonotic potential of *Giardia duodenalis* and *Cryptosporidium* spp. and prevalence of intestinal parasites in young dogs from different populations on Prince Edward Island, Canada. *Veterinary Parasitology* 196: 509-514.
525. Ahmed WM, Mousa WM, Aboelhadid SM, Tawfik MM (2014) Prevalence of zoonotic and other gastrointestinal parasites in police and house dogs in Alexandria, Egypt. *Veterinary World* 7: 275-280.
526. Gizzi AB, Oliveira ST, Leutenegger CM, Estrada M, Kozemjakin DA, Stedile R, Marcondes M, Biondo AW (2014) Presence of infectious agents and co-infections in diarrheic dogs determined with a real-time polymerase chain reaction-based panel. *BMC Veterinary Research* 10: 23.
527. Gharekhani J (2014) Study on gastrointestinal zoonotic parasites inpet dogs in Western Iran. *Turkiye Parazitoloji Dergisi* 38: 172-176.
528. Garedaghi Y, Karimi B (2014) Prevalence of intestinal protozoan parasites in stray dogs of Tabriz city, Iran. Indian Journal of Fundamental Applied Life Science 4: 20-24.
529. Johansen KM, Castro NS, Lancaster KE, Madrid E, Havas A, Simms J, Sterling CR (2014) Characterization of *Giardia lamblia* genotypes in dogs from Tucson, Arizona using SSU-rRNA and beta-giardin sequences. *Parasitology Research* 113: 387-390.
530. Mahmud MAA, Belal SMSH, Uddin FMJ (2014) Prevalence of protozoan diseases in pet dogs at district veterinary hospital, Sirajganj, Bangladesh. *Bangladesh Journal of Veterinary Medicine* 12: 191-196
531. Mota KCP, Gómez-Hernández C, Rezende-Oliveira K (2014) Frequency of intestinal parasites in dog stool samples in a town of the Pontaldo Triângulo region, Minas Gerais state, Brazil. *Revista de Patologia Tropical* 43: 219-227.
532. Neves D, Lobo L, Simoes PB, Cardoso L (2014) Frequency of intestinal parasites in pet dogs from an urban area (greater Oporto, northern Portugal). *Veterinary Parasitology* 200: 295-298.
533. Ortuno A, Scorza V, Castella J, Lappin M (2014) Prevalence of intestinalparasites in shelter and hunting dogs in Catalonia, Northeastern Spain. *The Veterinary Journal* 199: 465-467.
534. Pipia AP, Varcasia A, Tamponi C, Sanna G, Soda M, Paoletti B, Traversa D, Scala A (2014) Canine giardiosis in Sardinia Island, Italy:prevalence, molecular characterization, and risk factors. *The Journal of Infection in Developing Countries* 8: 655-660.
535. Procter TD, Pearl DL, Finley RL, Leonard EK, Janecko N, Reid-Smith RJ, Weese JS, Peregrine AS, Sargeant JM (2014) Across-sectional study examining *Campylobacter* and other zoonotic enteric pathogens in dogs that frequent dog parks in three cities insouth-western Ontario and risk factors for shedding of *Campylobacter* spp. *Zoonoses and Public Health* 61: 208-218.
536. Schar F, Inpankaew T, Traub RJ, Khieu V, Dalsgaard A, Chimnoi W, Chhoun C, Sok D, Marti H, Muth S, Odermatt P (2014) The prevalence and diversity of intestinal parasitic infections in humans and domestic animals in a rural Cambodian village. *Parasitology International* 63: 597-603.
537. Smith AF, Semeniuk CA, Kutz SJ, Massolo A (2014) Dog-walking behaviours affect gastrointestinal parasitism in park-attending dogs. *Parasites & Vectors* 7: 429.
538. Tseng YC, Ho GD, Chen TT, Huang BF, Cheng PC, Chen JL, Peng SY (2014) Prevalence and genotype of *Giardia duodenalis* from faecal samples of stray dogs in Hualien city of eastern Taiwan. *Tropical Biomedicine* 31: 305-311.
539. Yang D, Zhang Q, Zhang L, Dong H, Jing Z, Li Z, Liu J (2014) Prevalence and risk factors of *Giardia* *doudenalis* in dogs from China. *International Journal of Environmental Health Research* 25: 207-213.
540. Zanzani SA, Gazzonis AL, Scarpa P (2014) Intestinal Parasites of owned dogs and cats from metropolitan and micropolitan areas: prevalence, zoonotic risks, and pet owner awareness in Northern Italy. *BioMed Research International* Article ID: 696508.
541. Zheng G, Alsarakibi M, Liu Y, Hu W, Luo Q, Tan L, Li G (2014) Genotyping of *Giardia duodenalis* isolates from dogs in Guangdong, China based on multilocus sequence. *Korean Journal of Parasitology* 52: 299-304.
542. Alvarado-Esquivel C, Romero-Salas D, Aguilar-Domínguez M, Cruz-Romero A, Ibarra-Priego N, Pérez-de-León AA (2015) Epidemiological assessment of intestinal parasitic infections in dogs at animal shelter in Veracruz, Mexico. *Asian Pacific Journal of Tropical Biomedicine* 5: 34-39.
543. Gu YF, Wang K, Liu DY, Mei N, Chen C, Chen T, Han MM, Zhou L, Cao JT, Zhang H, Zhang XL, Fan ZL, Li WC (2015) Molecular detection of *Giardia lamblia* and *Cryptosporidium* species in pet dogs. *Zhongguo Ji Sheng Chong Xue Yu Ji Sheng Chong Bing Za Zhi* 33: 362-367. (in Chinese)
544. Li W, Li Y, Song M, Lu Y, Yang J, Tao W, Jiang Y, Wan Q, Zhang S, Xiao L (2015) Prevalence and genetic characteristics of *Cryptosporidium*, *Enterocytozoon bieneusi* and *Giardia duodenalis* in cats and dogs in Heilongjiang province, China. *Veterinary Parasitology* 208: 125-134.
545. Moskvina TV, Zheleznova LV (2015) A survey on endoparasites and ectoparasites in domestic dogs and cats in Vladivostok, Russia 2014. *Veterinary Parasitology: Regional Studies and Reports* 1-2: 31-34.
546. Paoletti B, Traversa D, Iorio R, De Berardinis A, Bartolini R, Salini R, Di Cesare A (2015) Zoonotic parasites in feces and fur of stray and private dogs from Italy. *Parasitology Research* 114: 135-141.
547. Sardarian K, Maghsood AH, Ghiasian SA, Zahirnia AH (2015) Prevalence of zoonotic intestinal parasites in Ho and stray dogs in rural areas of Hamadan, Western Iran. *Tropical Biomedicine* 32: 240-246.
548. Shin JC, Wehdnesday Bernardo Reyes A, Kim SH, Kim S, Park HJ, Seo KW, Song KH (2015) Molecular detection of *Giardia intestinalis* from stray dogs in animal shelters of Gyeongsangbuk-do (Province) and Daejeon, Korea. *Korean Journal of Parasitology* 53: 477-481.
549. Simonato G, di Regalbono AF, Cassini R, Traversa D, Beraldo P, Tessarin C, Pietrobelli M (2015) Copromicroscopic and molecular investigations on intestinal parasites in kenneled dogs. *Parasitology Research* 114: 1963-1970.
550. Villeneuve A, Polley L, Jenkins E, Schurer J, Gilleard J, Kutz S, Conboy G, Benoit D, Seewald W, Gagné F (2015) Parasite prevalence in fecal samples from shelter dogs and cats across the Canadian provinces. *Parasites & Vectors* 8: 281.
551. Ayinmode AB, Obebe OO, Olayemi E (2016) Prevalence of potentially zoonotic gastrointestinal parasites in canine faeces in Ibadan, Nigeria. *Ghana Medical Journal* 50: 201-206.
552. Ferreira JIGdS, Pena HFJ, Azevedo SS, Labruna MB, Gennari SM (2016) Occurrences of gastrointestinal parasites in fecal samples from domestic dogs in São Paulo, SP, Brazil. *Brazilian Journal of Veterinary Parasitology* 25: 435-440.
553. Hascall KL, Kass PH, Saksen J, Ahlmann A, Scorza AV, Lappin MR, Marks SL (2016) Prevalence of enteropathogens in dogs attending 3 regional dog parks in Northern California. *Journal of Veterinary Internal Medicine* 30: 1838-1845.
554. Piekarska J, Bajzert J, Gorczykowski M, Kantyka M, Podkowik M (2016) Molecular identification of *Giardia duodenalis* isolates from domestic dogs and cats in Wroclaw, Poland. *Annals of Agricultural Environmental Medicine* 23: 410-415.
555. Oliveira-Arbex AP, David EB, Oliveira-Sequeira TCG, Bittencourt GN, Guimaraes S (2016) Genotyping of *Giardia duodenalis* isolates in asymptomatic children attending daycare centre: evidence of high risk for anthroponotic transmission. *Epidemiology & Infection* 144: 1418-1428.
556. Qi M, Dong H, Wang R, Li J, Zhao J, Zhang L, Luo J (2016) Infection rate and genetic diversity of *Giardia duodenalis* in pet and stray dogs in Henan Province, China. *Parasitology International* 65: 159-162.
557. Quadros RMd, Wiess PHE, Marques SMT, Miletti LC (2016) Potential ceoss-contamination of similar *Giardia* *duodenalis* assemblages in children and pet dogs in southern Brazil, as determined by PCR-RFLP. *Revista do Instituto de Medicina Tropical de Sao Paulo* 58: 66.
558. Xu H, Jin Y, Wu W, Li P, Wang L, Li N, Feng Y, Xiao L (2016) Genotypes of *Cryptosporidium* spp., *Enterocytozoon bieneusi* and *Giardia duodenalis* in dogs and cats in Shanghai, China. *Parasites & Vectors* 9: 121.
559. Lee MF, Cadogan P, Copeland S, Walochnik J, Lindo JF (2017) Molecular epidemiology and multilocus sequence analysis of potentially zoonotic *Giardia* spp. from humans and dogs in Jamaica. *Prasitology Research* 116: 406-414.
560. Al-Jassim KBN, Mahmmod YS, Salem ZM, Al-Jubury A (2017) Epidemiological investigation of gastrointestinal parasites in dog populations in Basra province, Southern Iraq. *Journal of Parasitic Diseases* 41: 1006-1013.
561. Bartosik J, Dziwirek K, Lojek J, Kaczyk J, Gorski P (2017) Prevalence of intestinal parasite infection in dogs from selected rural areas of central and southern Poland. *Scientific Annals of Polish Society of Animal Production* 13: 61-69.
562. de Lucio A, Bailo B, Aguilera M, Cardona GA, Fernandez-Crespo JC, Carmena D (2017) No molecular epidemiological evidence supporting Ho transmission of zoonotic *Giardia* *duodenalis* and *Cryptosporidium* spp. from pet dogs and cats in the province of Álava, Northern Spain. *Acta* *Tropica* 170: 48-56.
563. Gil H, Cano L, de Lucio A, Bailo B, de Mingo MH, Cardona GA, Fernández-Basterra JA, Aramburu-Aguirre J, López-Molina N, Carmena D (2017) Detection and molecular diversity of *Giardia duodenalis* and *Cryptosporidium* spp. in sheltered dogs and cats in Northern Spain. *Infection, Genetics and Evolution* 50: 62-69.
564. Gillespie S, Bradbury RS (2017) A survey of intestinal parasites of domestic dogs in central Queensland. *Tropical Medicine Infectious Disease* 2: 60.
565. Kohansal MH, Fazaeli A, Nourian A, Haniloo A, Kamali K (2017) Dogs’ gastrointestinal parasites and their association with public health in Iran. *Journal of Veterinary Research* 61: 189-195.
566. Kostopoulou D, Claerebout E, Arvanitis D, Ligda P, Voutzourakis N, Casaert S, Sotiraki S (2017) Abundance, zoonotic potential and risk factors of intestinal parasitism amongst dog and cat populations: The scenario of Crete, Greece. *Parasites & Vectors* 10: 43.
567. Puebla LEJ, Núñez FA., Rivero LR, Hernández YR, Millán IA, Müller N (2017) Prevalence of intestinal parasites and molecular characterization of *Giardia duodenalis* from dogs in La Habana, Cuba. *Veterinary Parasitology: Regional Studies and Reports* 8: 107-112.
568. Simonato G, di Regalbono AF, Cassini R, Traversa D, Tessarin C, Di Cesare A, Pietrobelli M (2017) Molecular detection of *Giardia duodenalis* and *Cryptosporidium* spp. in canine faecal samples contaminating public areas in Northern Italy. *Parasitology Research* 116: 3411-3418.
569. Szwabe K, Błaszkowska J (2017) Stray dogs and cats as potential sources of soil contamination with zoonotic parasites. *Annals of Agricultural and Environmental Medicine* 24: 39-43.
570. Ferreira A, Alho AM, Otero D, Gomes L, Nijsse R, Overgaauw PAM, de Carvalho LM (2017) Urban dog parks as sources of canine parasites: contamination rates and pet owner behaviours in Lisbon, Portugal. *Journal of Environmental and Public Health* Article ID: 5984086.
571. Tangtrongsup S, Scorza AV, Reif JS, Ballweber LR, Lappin MR, Salman MD (2017) Prevalence and multilocus genotyping analysis of *Cryptosporidium* and *Giardia* isolates from dogs in Chiang Mai, Thailand. *Veterinary Sciences* 4: 26.
572. Zhang Y, Zhong Z, Deng L, Wang M, Li W, Gong C, Fu H, Cao S, Shi X, Wu K, Peng G (2017) Detection and multilocus genotyping of *Giardia duodenalis* in dogs in Sichuan province, China. *Parasite* 24: 31.
573. Adell-Aledón M, Köster PC, de Lucio A, Puente P, Hernández-de-Mingo M, Sánchez-Thevenet P, Dea-Ayuela MA, Carmena D (2018) Occurrence and molecular epidemiology of *Giardia duodenalis* infection in dog populations in eastern Spain. *BMC Veterinary Research* 14: 26.
574. De Liberato C, Berrilli F, Odorizi L, Scarcella R, Barni M, Amoruso C, Scarito A, Di Filippo MM, Carvelli A, Iacoponi F, Scaramozzino P (2018) Parasites in stray dogs from Italy: prevalence, risk factors and management concerns. *Acta Parasitologica* 63: 27-32.
575. Luis Enrique JP, Moreno LR, Núñez Fernández FA, Millán IA, Rivero LR, González FR, Pérez Rodríguez JC (2018) Prevalence of intestinal parasitic infections in dogs from Havana, Cuba: risk of zoonotic infections to humans. *Animal Husbandry, Dairy and Veterinary Science* 2: 1-5.
576. Mohaghegh MA, Vafaei MR, Ghomashlooyan M, Azami M, Falahati M, Azadi Y, Yousefi HA, Jabalameli Z, Hejazi SH (2018) A wide diversity of zoonotic intestinal parasites in domestic and stray dogs in rural areas of Kermanshah province, Iran. *Tropical Biomedicine* 35: 82-90.
577. Pan W, Wang M, Abdullahi AY, Fu Y, Yan X, Yang F, Shi X, Zhang P, Hang J, Li G (2018) Prevalence and genotypes of *Giardia lamblia* from stray dogs and cats in Guangdong, China. *Veterinary Parasitology: Regional Studies and Reports* 13: 30-34.
578. Sauda F, Malandrucco L, Macrì G, Scarpulla M, De Liberato C, Terracciano G, Fichi G, Berrilli F, Perrucci S (2018) *Leishmania* *infantum*, *Dirofilaria* spp. and other endoparasite infections in kennel dogs in central Italy. *Parasite* 25: 2.
579. Scaramozzino P, Carvelli A, Iacoponi F, De Liberato C (2018) Endoparasites in Ho and shelter dogs from Central Italy. *International Journal of Veterinary Science and Medicine* 6: 45-47.
580. Sommer MF, Rupp P, Pietsch M, Kaspar A, Beelitz P (2018) *Giardia* in a selected population of dogs and cats in Germany - diagnostics, coinfections and assemblages. *Veterinary Parasitology* 249: 49-56.
581. Uiterwijk M, Nijsse R, Kooyman FNJ, Wagenaar JA, Mughini-Gras L, Koop G, Ploeger HW (2018) Comparing four diagnostic tests for *Giardia duodenalis* in dogs using latent class analysis. *Parasites & Vectors* 11: 439.
582. Utaaker KS, Tysnes KR, Krosness MM, Robertson LJ (2018) Not just a walk in the park: Occurrence of intestinal parasites in dogs roaming recreational parks in Chandigarh, Northern India. *Veterinary Parasitology: Regional Studies and Reports* 14: 176-180.
583. Yu Z, Ruan Y, Zhou M, Chen S, Zhang Y, Wang L, Zhu G, Yu Y (2018) Prevalence of intestinal parasites in companion dogs with diarrhea in Beijing, China, and genetic characteristics of *Giardia* and *Cryptosporidium* species. *Parasitology Research* 117: 35-43.
584. Garcia-Campos A, Power C, O'Shaughnessy J, Browne C, Lawlor A, McCarthy G, O'Neill EJ, de Waal T (2019) One-year parasitological screening of stray dogs and cats in County Dublin, Ireland. *Parasitology* 146: 746-752.
585. Bartosik J, Lojek J, Pucha la M, Kaczyk J, Gorski P, Dlugosz E, Zygner W (2019) Prevalence of intestinal parasites detected in routine coproscopic methods in dogs and cats from the Masovian voivodeship in 2012-2015. *Medycyna Weterynaryjna* 75: 293-297.
586. Godínez-Galaz EM, Veyna-Salazar NP, Olvera-Ramírez AM, Milián-Suazo F, Perea-Razo CA, Bernal-Reynaga R, Cantó-Alarcón GJ (2019) Prevalence and zoonotic potential of *Giardia intestinalis* in dogs of the central region of Mexico. *Animals* 9: 325.
587. Homayouni MM, Razavi SM, Shaddel M, Asadpour M (2019) Prevalence and molecular characterization of *Cryptosporidium* spp. and *Giardia intestinalis* in Ho dogs and cats from Shiraz, Southwestern Iran. *Veterinaria Italiana* 55: 311-318.
588. Julien DA, Sargeant JM, Guy RA, Shapiro K, Imai RK, Bunce A, Sudlovenick E, Chen S, Li J, Harper SL (2019) Prevalence and genetic characterization of *Giardia* spp. and *Cryptosporidium* spp. in dogs in Iqaluit, Nunavut, Canada. *Zoonoses and Public Health* 66: 813-825.
589. Kim HY, Lee H, Lee SH, Seo MG, Yi S, Kim JW, Kim CH, Lee YR, So BJ, Kwon OD, Kwak D (2019) Multilocus genotyping and risk factor analysis of *Giardia duodenalis* in dogs in Korea. *Acta Tropica* 199: 105113.
590. Kurnosova OP, Arisov MV, Odoyevskaya IM (2019) Intestinal parasites of pets and other house-kept animals in Moscow. Helminthologia. 56: 108-117.
591. Li J, Dan X, Zhu K, Li N, Guo Y, Zheng Z, Feng Y, Xiao L (2019) Genetic characterization of *Cryptosporidium* spp. and *Giardia duodenalis* in dogs and cats in Guangdong, China. *Parasites & Vectors* 12: 571.
592. Lopez-Arias A, Villar D, Lopez-Osorio S, Calle-Velez D, Chaparro-Gutierrez JJ (2019) *Giardia* is the most prevalent parasitic infection in dogs and cats with diarrhea in the city of Medellin, Colombia. *Veterinary Parasitology: Regional Studies and Reports* 18: 100335.
593. Roegner AF, Daniels ME, Smith WA, Gottdenker N, Schwartz LM, Liu J, Campbell A, Fiorello CV (2019) *Giardia* infection and *Trypanosoma cruzi* exposure in dogs in the Bosawa´s biosphere reserve, Nicaragua. *Ecohealth* 16: 512-522.
594. Saleh MN, Heptinstall JR, Johnson EM, Ballweber LR, Lindsay DS, Werre S, Herbein JF, Zajac AM (2019) Comparison of diagnostic techniques for detection of *Giardia duodenalis* in dogs and cats. *Journal of Veterinary Internal Medicine* 33: 1272-1277.
595. Tangtrongsup S, Scorza AV, Reif JS, Ballweber LR, Lappin MR, Salman MD (2020) Seasonal distributions and other risk factors for *Giardia duodenalis* and *Cryptosporidium* spp. infections in dogs and cats in Chiang Mai, Thailand. *Preventive Veterinary Medicine* 174: 104820.
596. Kuzi S, Argentaro SE, Baneth G (2020) Prevalence of *Giardia duodenalis* infection, co-morbidities and associated risk factors in dogs admitted to a veterinary teaching hospital in Israel. *Comparative Immunology, Microbiology and Infectious Diseases* 68: 101401.
597. Liao S, Lin X, Sun Y, Qi N, Lv M, Wu C, Li J, Hu J, Yu L, Cai H, Xiao W, Sun M, Li G (2020) Occurrence and genotypes of *Cryptosporidium* spp., *Giardia* *duodenalis*, and *Blastocystis* sp. in Ho, shelter, breeding, and pet market dogs in Guangzhou, southern China. *Scientific Reports* 10: 17736.
598. Perrucci S, Berrilli F, Procopio C, Di Filippo MM, Pierini A, Marchetti V (2020) *Giardia duodenalis* infection in dogs affected by primary chronic enteropathy. *Open Veterinary Journal* 10: 74-79.
599. Smith AF, Neumann N, Banting G, Claudia Klein C, Liccioli S, Massolo A (2020) Molecular characterization of *Giardia* spp. and *Cryptosporidium* spp. from dogs and coyotes in an urban landscape suggests infrequent occurrence of zoonotic genotypes. *Veterinary Parasitology* 281: 109115.
600. Salant H, Kuzi S, Navarro D, Baneth G (2020) Prevalence and molecular characterization of *Giardia duodenalis* in dogs in Israel. *Comparative Immunology, Microbiology and Infectious Diseases* 73: 101548.
601. Trevisan YPA, de Almeida AdBPF, Nakazato L, Pacheco TdA, de Souza LI, Canei DH, Pereira ME, Maia MO, Pacheco RC, Sousa VRF (2020) Frequency of *Giardia duodenalis* infection and its genetic variability in dogs in Cuiabá, Midwest Brazil. *The Journal of Infection in Developing Countries* 14: 1431-1436.
602. Regidor-Cerrillo J, Arranz-Solís D, Moreno-Gonzalo J, Pedraza-Díaz S, Gomez-Bautista M, Ortega-Mora LM, Esther Collantes-Fernandez E (2020) Prevalence of intestinal parasite infections in stray and farm dogs from Spain. *Brazilian Journal of Veterinary Parasitology* 29: e014920.
603. Piekara-Stepinska A, Piekarska J, Gorczykowski M, Bania J (2021) Genotypes of *Giardia duodenalis* in Ho dogs and cats from Poland. *Acta* *Parasitologica* 66: 428-435.
604. Smigova J, Papajova I, Soltys J, Pipikova J, Smiga L, Snabel V, Takacova J, Takac L (2021) The occurrence of endoparasites in Slovakian Ho dogs and cats. *Veterinary Research Communications* 45: 243-249.
605. Abdelaziz AR, Sorour SSG (2021) Prevalence and molecular characterization of *Giardia duodenalis* assemblage D of dogs in Egypt, and its zoonotic implication. *Microbes, Infection and Chemotherapy* 1: e1268.
606. Raicevic JG, Pavlovic IN, Galonja-Coghill TA (2021) Canine intestinalis parasites as a potential source of soil contamination in the public areas of Krusevac, Serbia. *The Journal of Infection in Developing Countries* 15: 147-154.
607. Othman RA, Abuseir S (2021) The prevalence of gastrointestinal parasites in native dogs in Palestine. *Iranian Journal of Parasitology* 16: 435-442.
608. Pereira A, Teixeira J, Sousa S, Parreira R, Campino L, Meireles J, Maia C (2021) *Giardia duodenalis* infection in dogs from the metropolitan area of Lisbon, Portugal: prevalence, genotyping and associated risk factors. *Journal of Parasitic Diseases* 45: 372-379.
609. Sobotyk C, Upton KE, Lejeune M, Nolan TJ, Marsh AE, Herrin BH, Borst MM, Piccione J, Zajac AM, Camp LE, Pulaski CN, Starkey LA, Simson C, Verocai GG (2021) Retrospective study of canine endoparasites diagnosed by fecal flotation methods analyzed across veterinary parasitology diagnostic laboratories, United States, 2018. *Parasites & Vectors* 14: 439.
610. Osmari V, Alves MEM, Rodrigues FS, Braunig P, Cargnelutti JF, Vogel FSF, Botton SA, Sangioni LA (2021) Occurrence and molecular characterization of *Giardia duodenalis* from naturally infected dogs in the municipality of Santa Maria, Rio Grande do Sul, Brazil. *Pesquisa* *Veterinaria Brasileira* 41: e06670.
611. Grandi G, Victorsson I, Osterman-Lind E, Höglund J (2021) Occurrence of endoparasites in adult Swedish dogs: A coprological investigation. Front. *Veterinary Sciences* 8: 691853.
612. Agresti A, Berrilli F, Maestrini M, Guadano Procesi I, Loretti E, Vonci N, Perrucci S (2022) Prevalence, risk factors and genotypes of *Giardia duodenalis* in sheltered dogs in Tuscany (central Italy). *Pathogens* 11: 12.
613. Zhang X, Jian Y, Ma Y, Li Z, Fu Y, Cairang Z, Wang X, Duo H, Guo Z (2022) Prevalence of intestinal parasites in dog faecal samples from public environments in Qinghai province, China. *Pathogens* 11: 1240.
614. Pavlovic I (2022) Intestinal parasites of pet dogs in belgrade area in period 2020-2021. In: *15^th^ International Congress of Parasitology*, *Copenhagen, Denmark*.
615. Sui Y, Zhang X, Wang H, Yu F, Zheng L, Guo Y, Lu Y, Chen M, Wang B, Dai H, Liu F, Li J, Dong H, Tong C, Zhang L (2022) Prevalence and genetic diversity of *Giardia duodenalis* in pet dogs from Zhengzhou, central China and the association between gut microbiota and fecal characteristics during infection. *One Health* 14: 100401.
616. Cao Y, Fang C, Deng J, Yu F, Ma D, Chuai L, Wang T, Qi M, Li J (2022) Molecular characterization of *Cryptosporidium* spp. and *Giardia duodenalis* in pet dogs in Xinjiang, China. *Parasitology Research* 121: 1429-1435.
617. Krumrie S, Capewell P, McDonald M, Dunbar D, Panarese R, Katzer F, El Sakka N, Mellor D, Alexander CL, Weir W (2022) Molecular characterisation of *Giardia duodenalis* from human and companion animal sources in the United Kingdom using an improved triosephosphate isomerase molecular marker. *Current Research in Parasitology & Vector-Borne Diseases* 2: 100105.
618. Murnik LC, Daugschies A, Delling C (2023) Gastrointestinal parasites in young dogs and risk factors associated with infection. *Parasitology* *Research* 122: 585-596.
619. Faraguna S, Vlahek I, Miocic KT, Andeanszky T, Pecin M (2023) Prevalence of intestinal parasites in dogs and cats from the Kvarner region in Croatia. *Acta Veterinaria-Beograd* 73: 41-54.
620. Harvey TV, Carvalho JPS, Aquino MCC, Oliveira BCM, Barros LD, Fehlberg HF, Rocha CMBM, Albuquerque GR (2023) Giardiasis in children and dogs, and the first report of assemblage E in dogs from northeastern Brazil. *Brazilian Journal of Veterinary Parasitology* 32(1):e012222.
621. Esmailzadeh R, Malekifard F, Rakhshanpour A, Tavassoli M (2023) Frequency and genotyping of *Giardia duodenalis* in dogs of Urmia, northwest of Iran. *Veterinary Research Forum* 14: 335-340.
622. Kurnosova OP, Panova OA, Arisov MV (2023) The prevalence of potentially zoonotic intestinal parasites in dogs and cats in Moscow, Russia. *Helminthologia* 60: 44-51.
623. Taylor LA, Saleh MN, Kneese EC, Vemulapalli TH, Verocai GG (2023) Comparison of 3 diagnostic tests for the detection of *Giardia* and *Cryptosporidium* spp. in asymptomatic dogs (*Canis lupis familiaris*). *Journal of the American Association for Laboratory Animal Science* 62: 1-8.
624. Elmahallawy EK, Gareh A, Abu-Okail A, Köster PC, Dashti A, Asseri J, Gouda AA, Mubaraki MA, Mohamed SA-A, Mohamed YM, Hassan EA, Elgendy M, Hernández-Castro C, Bailo B, González-Barrio D, Xiao L, Carmena D (2023) Molecular characteristics and zoonotic potential of enteric protists in domestic dogs and cats in Egypt. Frontiers in Veterinary Science 10: 1229151.
625. de Waal T, Aungier S, Lawlor A, Goddu T, Jones M, Szlosek D (2023) Retrospective survey of dog and cat endoparasites in Ireland: antigen detection. *Animals* 13: 137.
626. Zhang J, Qin Y, Shen Y, Wang Y, Cao J, Su Y, Liu H (2023) Prevalence and genotyping of *Cryptosporidium* spp. and *Giardia lamblia* in dogs and cats from a pet hospital in Shanghai Municipality. *Zhongguo Xue Xi Chong Bing Fang Zhi Za Zhi* 35: 258-262. (in Chinese)
627. Mateo M, Montoya A, Bailo B, Köster PC, Dashti A, Hernández-Castro C, Saugar JM, Matas P, Xiao L, Carmena D (2023) Prevalence and public health relevance of enteric parasites in domestic dogs and cats in the region ofMadrid (Spain) with an emphasis on *Giardia* *duodenalis* and *Cryptosporidium* sp.. *Veterinary Medicine and Science* 9: 2542-2558.
628. Souza JBB, Silva ZMdA, Alves-Ribeiro BS, Moraes IdS, Alves-Sobrinho AV, Saturnino KC, Ferraz HT, Machado MRF, Braga ÍA, Ramos DGdS (2023) Prevalence of intestinal parasites, risk factors and zoonotic aspects in dog and cat population from Goiás, Brazil. *Veterinary Sciences* 10: 492.
629. Joachim A, Auersperg V, Drue J, Wiedermann S, Hinney B, Spergser J (2023) Parasites and zoonotic bacteria in the feces of cats and dogs from animal shelters in Carinthia, Austria. *Research in Veterinary Science* 164: 105022.
630. Idan SR, Al-Hasnawy MH (2023) Microscopic and molecular diagnoses of *Giardia duodenalis* in pet animals in Babylon Province, Iraq. *Veterinary* *World* 16: 2263-2270.
631. Hsu CH, Liang C, Chi SC, Lee KJ, Chou CH, Lin CS, Yang WY (2023) An epidemiological assessment of *Cryptosporidium* and *Giardia* spp. infection in pet animals from Taiwan. *Animals* 13: 3373.
632. Ipek DNS (2023) Molecular characterization and zoonotic significance of *Cryptosporidium spp.* and *Giardia duodenalis* in asymptomatic adult stray cats and dogs in Turkey. *Indian Journal of Animal Research* DOI: 10.18805/IJAR.BF-1707.
633. Trout J, Santin M, Fayer R (2006) *Giardia* and *Cryptosporidium* species and genotypes in coyotes (*Canis latrans*). *Journal of Zoo and Wildlife Medicine* 37:141-144.
634. Thompson RCA, Colwell DD, Shury T, Appelbee AJ, Read C, Njiru Z, Olson ME (2009) The molecular epidemiology of *Cryptosporidium* and *Giardia* infections in coyotes from Alberta, Canada, and observations on some cohabiting parasites. *Veterinary Parasitology* 159: 167-170.
635. Kloch A, Bednarska M, Bajer A (2005) Intestinal macro and microparasites of wolves (*Canis lupus l.*) from north-eastern Poland recovered by coprological study. *Annals of Agricultural and Environmental Medicine* 12: 237-245.
636. Perrucci S, Maestrini M, Coppola F, Di Marco M, Rosso AD, Pacini MI, Zintu P, Felicioli A (2023) gray wolf (*Canis lupus italicus*) and red fox (*Vulpes vulpes*) parasite survey in anthropized and natural areas of central Italy. Veterinary Sciences 10: 108.
637. Mateo M, Mingo MHd, Lucio Ad, Morales L, Balseiro A, Espi A, Barral M, Lima Barbero JF, Habela MA, Fernandez-Garcia JL, Bernal RC, Koster PC, Cardona GA, Carmena D (2017) Occurrence and molecular genotyping of *Giardia duodenalis* and *Cryptosporidium* spp. in wild mesocarnivores in Spain. *Veterinay Parasitology* 235: 86-93.
638. Barbosa AdS, Pinheiro JL, dos Santos CR, de Lima CSCC, Dib LV, Echarte GV, Augusto AM, Bastos ACMP, Antunes Uchôa CM, Bastos OMP, Santos FN, Fonseca ABM, Amendoeira MRR (2020) gastrointestinal parasites in captive animals at the Rio de Janeiro zoo. *Acta Parasitologica* 65: 237-249.
639. Ash A, Lymbery A, Lemon J, Vitali S, Thompson RCA (2010) Molecular epidemiology of *Giardia duodenalis* in a endangered carnivore - the African painted dog. *Veterinary Parasitology* 174: 206-212.
640. Solarczyk P, Majewska AC, Jędrzejewski S, Górecki MT, Nowicki S, Przysiecki P (2016) First record of *Giardia* assemblage D infection in farmed raccoon dogs (*Nyctereutes procyonoides*). *Annals of Agricultural and Environmental Medicine* 23: 696-698
641. Zhang XX, Zheng WB, Ma JG, Yao QX, Zou Y, Bubu CJ, Zhao Q, Zhu XQ (2016) Occurrence and multilocus genotyping of *Giardia intestinalis* assemblage C and D in farmed raccoon dogs, *Nyctereutes procyonoides*, in China. *Parasites & Vectors* 9: 471.
642. Elmore SA, Lalonde LF, Samelius G, Alisauskas RT, Gajadhar AA, Jenkins EJ (2013) Endoparasites in the feces of arctic fox in a terrestrial ecosystem in a Canada. *International Journal for Parasitology: Parasites and Wildlife* 2: 90-92.
643. Hamnes IS, Gjerde BK, Forberg T, Robertson LJ (2007) Occurrence of *Giardia* and *Cryptosporidium* in Norwegian red foxes (*Vulpes vulpes*). *Veterinary Parasitology* 143: 347-353.
644. Hodzic A, Alic A, Omeragic J (2014) Occurrence of *Cryptosporidium* spp. and *Giardia duodenalis* in red foxes (*Vulpes vulpes*) in Bosnia and Herzegovina. *Macedonia Veterinary Reviw* 37: 189-192.
645. Onac D, Oltean M, Mircean V, Jarca A, Cozma V (2015) Occurrence of *Giardia duodenalis* zoonotic assemblages in red foxes from Romania. Science Parasitology 16: 177-180.
646. Debenham JJ, Landuyt H, Troell K, Tysnes K, Robertson LJ (2017) Occurrence of *Giardia* in Swedish red foxes (*Vulpes vulpes*). *Journal of Wildlife* *Diseases* 53: 649-652.
647. Papini RA, Verin R (2019) *Giardia* and *Cryptosporidium* in red foxes (*Vulpes vulpes*): screening for coproantigens in a population of central Italy and mini-review of the literature. *Macedonia Veterinary Reviw* 42: 101-106.
648. Barrera JP, Carmena D, Rodríguez E, Checa R, Lopez AM, Fidalgo LE, Galvez R, Marino V, Fuentes I, Miro G, Montoya A (2020) The red fox (*Vulpes vulpes*) as a potential natural reservoir of human cryptosporidiosis by *Cryptosporidium* *hominis* in Northwest Spain. *Transboundary Emerging Diseases* 67: 2172-2182.
649. Mendez-Hermida F, Gomez-Couso H, Romero-Suances R, Ares-Mazas E (2007) *Cryptosporidium* and *Giardia* in wild otters (*Lutra lutra*). *Veterinary Parasitology* 144: 153-156.
650. Maestrini M, Berrilli F, Di Rosso A, Coppola F, Guadano Procesi I, Mariacher A, Felicioli A, Perrucci S (2022) Zoonotic *Giardia duodenalis* genotypes and other gastrointestinal parasites in a badger population living in an anthropized area of central Italy. *Pathogens* 11: 906.
651. Williams ES, Thorne ET, Appel MJG, Belitsky DW (1988) Canine disaster in black-footed ferrets (*Mustela nigripes*) from Wyoming. *Journal of* *Wildlife Diseases* 24: 385-398.
652. Chilvers BL, Cowan PE, Waddington DC, Kelly PJ, Brown TJ (1998) The prevalence of infection of *Giardia* spp. and *Cryptosporidium* spp. in wild animals on farmland, southeastern North Island, New Zealand. *International Journal of Environmental Health Research* 8: 59-64.
653. Pantchev N, Globokar-Vrhovec M, Beck W (2005) Endoparasites from indoor kept small mammals and hedgehogs. Laboratory evaluation of fecal, serological, and urinary samples (2002–2004). *Tierarztliche Praxis* 33: 296-306. (in Germany).
654. Pantchev N, Gassmann D, Globokar-Vrhovec M (2011) Increasing numbers of *Giardia* (but not coccidian) infections in ferrets, 2002 to 2010. *Veterinary Record* 168: 519.
655. Delport TC, Asher AJ, Beaumont LJ, Webster KN, Harcourt RG, Power ML (2014) *Giardia duodenalis* and *Cryptosporidium* occurrence in Australian sea lions (*Neophoca cinerea*) exposed to varied levels of human interaction. *International Journal for Parasitology:* *Parasites and* *Wildlife* 3: 269-275.
656. Appelbee AJ, Thompson RCA, Measures LM, Olson ME (2010) *Giardia* and *Cryptosporidium* in harp and hooded seals from the Gulf of St. Lawrence, Canada. *Veterinary Parasitology* 173: 19-23.
657. Dixon BR, Parrington LJ, Parenteau M, Leclair D, Santin M, Fayer R (2008) *Giardia duodenalis* and *Cryptosporidium* spp. in the intestinal contents of ringed seals (*Phoca hispida*) and bearded seals (*Erignathus barbatus*) in Nunavik, Quebec, Canada. *Journal of Parasitology* 94: 1161-1163.
658. Gaydos JK, Miller WA, Johnson C, Zornetzer H, Melli A, Packham A, Jeffries SJ, Lance MM, Conrad PA (2008) Novel and canine genotypes of *Giardia duodenalis* in harbor seals (*Phoca vitulina richardsi*). *Journal of Parasitology* 94: 1264-1268.
659. Sogayar MIL, Yoshida EL (1995) *Giardia* survey in live-tapped small domestic and wild mammals in four regions in the Southwest region or the state of Sao Paulo, Brazil. *Memorias do Instituo Oswaldo Cruz* 90: 675-678.
660. Pantoja DKSQ, Pereira WLA, Bernal MKM, Tavares HA, da Silva ALM, Silva MCdM (2017) *Cryptosporidium* spp. oocysts and *Giardia* spp. cysts in coatis (*Nasua nasua* L. 1766) from Para, Brazil. *Acta Veterinaria Brasilica* 11: 175-179.
661. Lombardo A, Diano M, Brocherel G, Palmerini L, Giovannini S, Mezher Z, Iurescia M, Cerci T, Caprioli A, Eleni C, Raso C, Mariacher A, Del Lesto I, Cappai N, Mattioli L, De Liberato C, Fichi G (2023) Detection of endoparasites in non-native raccoons from central Italy. *Veterinary Sciences* 10: 171.
662. Figueroa J (2015) New records of parasites in free-rangeing andean bears from Peru. *Ursus* 26: 21-27.
663. Aghazadeh M, Elson-Riggins J, Reljić S, De Ambrogi M, Huber D, Majnarić D, Carlos Hermosilla C (2015) Gastrointestinal parasites and the fi rst report of *Giardia* spp. in a wild population of European brown bears (*Ursus arctos*) in Croatia. *Veterinarski Arhiv* 85: 201-210.
664. Hill SL, Cheney JM, Taton-Allen GF, Reif JS, Bruns C, Lappin MR (2000) Prevalence of enteric zoonotic organisms in cats. *Journal of the American Veterinary Medical Association* 216: 687-692.
665. Spain CV, Scarlett JM, Wade SE, McDonough P (2001) Prevalence of enteric zoonotic agents in cats less than 1 year old in central New York State. *Journal of Veterinary Internal Medicine* 15: 33-38.
666. McGlade TR, Robertson ID, Elliot AD, Read C, Thompson RCA (2003) Gastrointestinal parasites of domestic cats in Perth, Western Australia. *Veterinary Parasitology* 117: 251-262.
667. Serra CMB, Uchôa CMA, Coimbra RA (2003) Parasitological study with faecal samples of stray and domiciliated cats (*Felis catus domesticus*) from the Metropolitan Area of Rio de Janeiro, Brazil. *Revista da Sociedade Brasileira de Medicina Tropical* 36: 331-334.
668. Gookin JL, Stebbins ME, Hunt E, Burlone K, Fulton M, Hochel R, Talaat M, Poore M, Levy MG (2004) Prevalence of and risk factors for feline *Tritrichomonas foetus* and *Giardia* infection. *Journal of Clinical Microbiology* 42: 2707-2710.
669. Pezeshki A, Rezaeian M, Zarebavani M (2005) Study of the genetic resemblance of tpi gene in cat and human *Giardia* using PCR-RFLP method. *Journal of Ardabil University of Medical Sciences* 13: 164-173.
670. De Santis-Kerr AC, Raghavan M, Glickman NW, Caldanaro RJ, Moore GE, Lewis HB, Schantz PM, Glickman LT (2006) Prevalence and risk factors for *Giardia* and coccidia species of pet cats in 2003-2004. *Journal of Feline Medicine and Surgery* 8: 292-301.
671. Itoh N, Muraoka N, Kawamata J, Aoki M, Itagaki T (2006) Prevalence of *Giardia duodenalis* infection in Ho cats of Tohoku district in Japan. *The Journal of Veterinary Medical Science* 68: 161-163.
672. Santin M, Trout JM, Vecino JAC, Dubey JP, Fayer R (2006) *Cryptosporidium*, *Giardia* and *Enterocytozoon* *bieneusi* in cats from Bogota (Colombia) and genotyping of isolates. *Veterinary Parasitology* 141: 334-339.
673. Mekaru SR, Marks SL, Felley AJ, Chouicha N, Kass PH (2007) Comparison of direct immunofluorescence, immunoassays, and fecal flotation for detection of *Cryptosporidium* spp. and *Giardia* spp. in naturally exposed cats in 4 Northern California Animal Shelters. *Journal of Veterinary Internal Medicine* 21: 959-965.
674. Natale A, di Regalbono AF, Zanellato G, Cavalletto M, Danesi P, Capelli G, Pietrobelli M (2007) Parasitological survey on stray cat colonies from the Veneto region. *Veterinary Research Communications* 31: 241-244.
675. Papini R, Giuliani G, Gorini G, Cardini G (2007) Survey of feline giardiasis by ELISA test in Italy. *Veterinary Research Communications* 31: 297-303.
676. Vasilopulos RJ, Rickard LG, Mackin AJ, Pharr GT, Huston CL (2007) Genotypic analysis of *Giardia duodenalis* in domestic cats. *Journal of Veterinary Internal Medicine* 21: 352-355.
677. Tzannes S, Batchelor DJ, Graham PA, Pinchbeck GL, Wastling J, German AJ (2008) Prevalence of *Cryptosporidium*, *Giardia* and *Isospora* species infections in pet cats with clinical signs of gastrointestinal disease. *Journal of Feline Medicine and Surgery* 10: 1-8.
678. Arbabi M, Hooshyar H (2009) Gastrointestinal parasites of stray cats in Kashan, Iran. *Tropical Biomedicine* 26: 16-22.
679. Ballweber LR, Panuska C, Huston CL, Vasilopulos R, Pharr RG, Mackin A (2009) Prevalence of and risk factors associated with shedding of *Cryptosporidium felis* in domestic cats of Mississippi and Alabama. *Veterinary Parasitology* 160: 306-310.
680. Bissett SA, Stone ML, Malik R, Norris JM, O’Brien C, Mansfield CS, Nicholls JM, Griffin A, Gookin JL (2009) Observed occurrence of *Tritrichomonas* *foetus* and other enteric parasites in Australian cattery and shelter cats. *Journal of Feline Medicine and Surgery* 11: 803-807.
681. Coelho WMD, Amarante AFTd, de Soutello RVG, Meireles MV, Bresciani KDS (2009) Occurrence of gastrointestinal parasites in fecal samples of cats in Andradina City, São Paulo. *Revista Brasileira de Parasitologia Veterinaria* 18: 46-49.
682. Gow AG, Gow DJ, Hall EJ, Langton D, Clarke C, Papasouliotis K (2009) Prevalence of potentially pathogenic enteric organisms in clinically healthy kittens in the UK. *Journal of Feline Medicine and Surgery* 11: 655-662.
683. Dall´Agnol LP, Otto MA, da Silva AS, Monteiro SG (2010) Gastrointestinal parasites in naturally infected cats in the municipality of Santa Maria in Rio Grande do Sul, Brazil. *Acta Veterinaria Brasilica* 4: 181-184.
684. Kingsbury DD, Marks SL, Cave NJ, Grahn RA (2010) Identification of *Tritrichomonas foetus* and *Giardia* spp. infection in pedigree show cats in New Zealand. *New Zealand Veterinary Journal* 58: 6-10.
685. Mircean V, Titilincu A, Vasile C (2010) Prevalence of endoparasites in Ho cat (*Felis catus*) populations from Transylvania (Romania) and association with risk factors. *Veterinary Parasitology* 171: 163-166.
686. Borji H, Razmi G, Ahmadi A, Karami H, Yaghfoori S, Abedi V (2011) A survey on endoparasites and ectoparasites of stray cats from Mashhad (Iran) and association with risk factors. *Journal of Parasitic Diseases* 35: 202-206.
687. Lucio-Forster A, Bowman DD (2011) Prevalence of fecal-borne parasites detected by centrifugal flotation in feline samples from two shelters in upstate New York. *Journal of Feline Medicine and Surgery* 13: 300-303.
688. Jaros D, Zygner W, Jaros S, Wedrychowicz H (2011) Detection of *Giardia intestinalis* assemblages A, B and D in domestic cats from Warsaw, Poland. *Polish Journal of Microbiology* 60: 259-263.
689. Khalafalla RE (2011) A survey study on gastrointestinal parasites of stray cats in Northern region of Nile Delta, Egypt. *PLoS ONE* 6: e20283.
690. Mircean V, Gyorke A, Jarca A, Cozma V (2011) Prevalence of *Giardia* species in stool samples by ELISA in Ho cats from Romania and risk factors. *Journal of Feline Medicine and Surgery* 13: 479-482.
691. Paoletti B, Otranto D, Weigl S, Giangaspero A, Di Cesare A, Traversa D (2011) Prevalence and genetic characterization of *Giardia* and *Cryptosporidium* in cats from Italy. *Research in Veterinary Science* 91: 397-399.
692. Suzuki J, Murata R, Kobayashi S, Sadamasu K, Kai A, Takeuchi T (2011) Risk of human infection with *Giardia duodenalis* from cats in Japan and genotyping of the isolates to assess the route of infection in cats. *Parasitology* 138: 493-500.
693. Tysnes K, Gjerde B, Nødtvedt A, Skancke E (2011) A cross-sectional study of *Tritrichomonas foetus* infection among healthy cats at shows in Norway. *Acta Veterinaria Scandinavica* 53: 39.
694. Nareaho A, Puomio J, Saarinen K, Jokelainen P, Juselius T, Antti Sukura A (2012) Feline intestinal parasites in Finland: prevalence, risk factors and anthelmintic treatment practices. *Journal of Feline Medicine and Surgery* 14: 378-383.
695. Queen EV, Marks SL, Farver TB (2012) Prevalence of selected bacterial and parasitic agents in feces from diarrheic and healthy control cats from Northern California. *Journal of Veterinary Internal Medicine* 26: 54-60.
696. Capari B, Hamel D, Visser M, Winter R, Pfister K, Rehbein S (2013) Parasitic infections of domestic cats, *Felis catus*, in western Hungary. *Veterinary Parasitology* 192: 33-42.
697. Hoopes JH, Polley L, Wagner B, Jenkins EJ (2013) A retrospective investigation of feline gastrointestinal parasites in western Canada. *The* *Canadian Veterinary Journal* 54: 359-362.
698. Pivoto FL, Lopes LFD, Voge FSF, Botton SdA, SangioniI LA (2013) Occurrence of gastrointestinal parasites and parasitism risk factors in domestic cats in Santa Maria, RS, Brazil. *Ciência Rural* 43: 1453-1458.
699. Spada E, Proverbio D, Pepa AD, Domenichini G, De Giorgi GB, Traldi G, Ferro E (2013) Prevalence of faecal-borne parasites in colony stray cats in northern Italy. *Journal of Feline Medicine and Surgery* 15: 672-677.
700. Beugnet F, Bourdeau P, Chalvet-Monfray K, Cozma V, Farkas R, Guillot J, Halos L, Joachim A, Losson B, Miró G, Otranto D, Renaud M, Rinaldi L (2014) Parasites of domestic owned cats in Europe: co-infestations and risk factors. *Parasites & Vectors* 7: 291.
701. Khademvatan S, Abdizadeh R, Rahim F, Hashemitabar M, Ghasemi M, Tavalla M (2014) Stray cats gastrointestinal parasites and its association with public health in Ahvaz City, South Western of Iran. *Jundishapur Journal of Microbiology* 7: e11079.
702. Knaus M, Rapti D, Shukullari E, Kusi I, Postoli R, Xhaxhiu D, Silaghi C, Hamel D, Visser M, Winter R, Rehbein S (2014) Characterisation of ecto- and endoparasites in domestic cats from Tirana, Albania. *Parasitology Research* 113: 3361-3371.
703. Paris JK, Wills S, Balzer HJ, Shaw DJ, Gunn-Moore DA (2014) Enteropathogen co-infection in UK cats with diarrhoea. BMC Veterinary Research 10: 13.
704. Polak KC, Levy JK, Crawford PC, Leutenegger CM, Moriello KA (2014) Infectious diseases in large-scale cat hoarding investigations. *The* *Veterinary Journal* 201: 189-195.
705. Hadi AM, Azhar AF (2014) Role of domestic cats *Felis catus* as reservoir hosts of internal parasites and protozoa in Baghdad. *Bulletin of the Iraq* *Natural History Museum* 13: 89-94.
706. Mancianti F, Nardoni S, Mugnaini L, Zambernardi L, Guerrini A, Gazzola V, Papini RA (2015) A retrospective molecular study of select intestinal protozoa in healthy pet cats from Italy. *Journal of Feline Medicine and Surgery* 17: 163-167.
707. Yang R, Ying JLJ, Monis P, Ryan U (2015) Molecular characterisation of *Cryptosporidium* and *Giardia* in cats (*Felis catus*) in Western Australia. *Experimental Parasitology* 155: 13-18.
708. Zheng G, Hu W, Liu Y, Luo Q, Tan L, Li G (2015) Occurrence and molecular identification of *Giardia duodenalis* from stray cats in Guangzhou, Southern China. *Korean Journal of Parasitology* 53: 119-124.
709. Hinney B, Ederer C, Stengl C, Wilding K, Strkolcova G, Harl J, Flechl E, Fuehrer HP, Joachim A (2015) Enteric protozoa of cats and their zoonotic potential-a field study from Austria. *Parasitology Research* 114: 2003-2006.
710. Ito Y, Iijima Y, Itoh N, Kimura Y (2017) Multilocus genotyping of *Giardia duodenalis* isolates from breeding cattery cats in Japan. *Journal of Feline Medicine and Surgery Open Reports* 3: 2055116917745237.
711. Nagamori Y, Payton ME, Duncan-Decocq R, Johnson EM (2018) Fecal survey of parasites in free-roaming cats in northcentral Oklahoma, United States. *Veterinary Parasitology: Regional Studies and Reports* 14: 50-53.
712. Symeonidou I, Gelasakis AI, Arsenopoulos K, Angelou A, Beugnet F, Papadopoulos E (2018) Feline gastrointestinal parasitism in Greece: emergent zoonotic species and associated risk factors. *Parasites & Vectors* 11: 227.
713. Li W, Liu X, Gu Y, Liu J, Luo J (2019) Prevalence of *Cryptosporidium*, *Giardia*, *Blastocystis*, and trichomonads in domestic cats in East China. *The* *Journal of Veterinary Medical Sciences* 81: 890-896.
714. Enemark HL, Starostka TP, Larsen B, Takeuchi-Storm N, Thamsborg SM (2020) *Giardia* and *Cryptosporidium* infections in Danish cats: risk factors and zoonotic potential. *Parasitology Research* 119: 2275-2286.
715. Kwak D, Seo MG (2020) Genetic analysis of zoonotic gastrointestinal protozoa and microsporidia in shelter cats in South Korea. *Pathogens* 9: 894.
716. Sursal N, Simsek E, Yildiz K (2020) Feline giardiasis in Turkey: prevalence and genetic and haplotype diversity of *Giardia duodenalis* based on the ꞵ-giardin gene sequence in symptomatic cats. *The Journal of Parasitology* 106: 699-706.
717. Oh YI, Seo KW, Kim DH, Cheon DS (2021) Prevalence, co-infection and seasonality of fecal enteropathogens from diarrheic cats in the Republic of Korea (2016-2019): a retrospective study. *BMC Veterinary Research* 17: 367.
718. Procesi IG, Carnio A, Berrilli F, Di Filippo MM, Scarito A, Amoruso C, Barni M, Ruffini M, Barlozzari G, Scarpulla M, De Liberato C (2022) *Giardia duodenalis* in colony stray cats from Italy. *Zoonoses and Public Health* 69: 46-54.
719. Lee Dk, Lee Hj, Song Jh, Song Kh (2022) Prevalence of giardiasis of stray cats in the Daejeon city. *Korean Journal of Veterinary Services* 45: 249-252.
720. Veyna-Salazar NP, Cantó-Alarcón GJ, Olvera-Ramírez AM, Ruiz-López FJ, Bernal-Reynaga R, Bárcenas-Reyes I, Durán-Aguilar M (2023) Occurrence of *Giardia duodenalis* in cats from Queretaro and the risk to public health. *Animals* 13: 1098.
721. Karimi P, Shafaghi‑Sisi S, Meamar AR, Razmjou E (2023) Molecular identification of *Cryptosporidium*, *Giardia*, and *Blastocystis* from stray and Ho cats and cat owners in Tehran, Iran. *Scientific Reports* 13: 1554.
722. Celik BA, Celik OY, Ayan A, Akyildiz G, Kiling OO, Ayan OO, Ercan K (2023) Preliminary investigation of the prevalence and genotype distribution of *Cryptosporidium* spp., and *Giardia duodenalis* in cats in Siirt, Turkey. *Acta Veterinaria* 73: 317-324.
723. Yun CS, Moon BY, Lee K, Kang SM, Ku BK, Hwang MH (2023) The detection and phylogenetic characterization of *Cryptosporidium*, *Cystoisospora*, and *Giardia duodenalis* of cats in South Korea. *Frontiers in Cellular and Infection Microbiology* 13: 1296118.
724. Milstein TC, Goldsmid JM (1997) Parasites of feral cats from southern Tasmania and their potential significance. *Australian Veterinary Journal* 75: 218-219.
725. Solarczyk P, Osten-Sacken N, Frantz AC, Schneider S, Pir JB, Heddergott M (2019) First molecular detection of *Giardia duodenalis* assemblage B in a free-living European wildcat (*Felis s. silvestris*) from Luxembourg. *Acta Protozoologica* 58: 1-5.
726. Velante NAP, Oronan RB, Reyes MF, Divina BP (2017) *Giardia guodenalis* in captive tigers (*Panthera tigris*), palawan bearcats (*Arctictis binturong whitei*) and asian palm civet (*Paradoxurus hermaphroditus*) at a wildlife facility in Manila, Philippines. *Iranian Journal of Parasitology* 12: 348-354.
727. Yu Z, Wen X, Huang X, Yang R, Guo Y, Feng Y, Xiao L, Li N (2020) Molecular characterization and zoonotic potential of *Enterocytozoon bieneusi*, *Giardia* *duodenalis* and *Cryptosporidium* sp. in farmed masked palm civets (*Paguma larvata*) in southern China. *Parasites & Vectors* 13: 403.
728. Lima VFS, Rocha PA, Silva MAD, Beltrão-Mendes R, Ramos RAN, Giannelli A, Rinaldi L, Cringoli G, Estrela PC, Alves LC (2018) Survey on helminths and protozoa of free-living Neotropical bats from Northeastern Brazil. *Acta Tropica* 185: 267-272.
729. Carbonara M., Mendonza-Roldan JA., Perles L., Alfaro-Alarcon A., Romero LM., Murillo DB., Piche-Ovares M., Corrales-Aguilar E., Latta R., Walochnik J, Santoro M, Otanto D (2023) Parasitic fauna of bats from Costa Rica. *International Journal for Parasitology*: *Parasites and Wildlife* 20: 63-72.
730. Li N, Ayinmode AB, Zhang H, Feng Y, Xiao L (2019) Host-adapted *Cryptosporidium* and *Enterocytozoon bieneusi* genotypes in straw-colored fruit bats in Nigeria. *International Journal for Parasitology: Parasites and Wildlife* 8: 19-24.
731. Adhikari RB, Maharjan M, Ghimire TR (2020) Prevalence of gastrointestinal parasites in the frugivorous and the insectivorous bats in Southcentral Nepal. *Journal of Parasitology Research* Article ID: 8880033.
732. Thompson J, Yang R, Power M, Hufschmid J, Beveridge I, Reid S, Ng J, Armson A, Ryan U (2008) Identification of zoonotic *Giardia* genotypes in marsupials in Australia. *Experimental Parasitology* 120: 88-93
733. Thompson RCA, Smith A, Lymbery AJ, Averis S, Morris KD, Wayne AF (2010) *Giardia* in Western Australia wildlife. *Veterinary Parasitology* 170: 207-211.
734. Bettiol SS, Kettlewell JS, Davies NJ, Goldsmid J (1997) Giardiasis in native marsupials of Tasmania. *Journal of Wildlife diseases* 33: 352-354.
735. Barbosa A, Reiss A, Jackson B, Warren K, Paparini A, Gillespie G, Stokeld D, Irwin P, Ryan U (2017) Prevalence, genetic diversity and potential clinical impact of blood-borne and enteric protozoan parasites in native mammals from northern Australia. *Veterinary Parasitology* 238: 94-105.
736. Wait LF, Fox S, Peck S, Power ML (2017) Molecular characterization of *Cryptosporidium* and *Giardia* from the Tasmanian devil (*Sarcophilus* *harrisii*). *PLoS ONE* 12: e174994.
737. Fehlberg HF, Matos Ribeiro C, Brito Junior PdA, Miranda Oliveira BC, Albano dos Santos C, del Valle Alvarez MR, Harvey TV, Albuquerque GR (2021) Detection of *Cryptosporidium* spp. and *Giardia* *duodenalis* in small wild mammals in northeastern Brazil. *PLoS ONE* 16: e0256199.
738. McCarthy S, Ng J, Gordon C, Miller R, Wyber A, Ryan UM (2008) Prevalence of *Cryptosporidium* and *Giardia* species in animals in irrigation catchments in the southwest of Australia. *Experimental Parasitology* 118: 596-599.
739. Marino MR, Brown TJ, Waddington DC, Brockie RE, Kelly PJ (1992) *Giardia intestinalis* in north island possums, house mice and ship rats. *New* *Zealand Veterinary Journal* 40: 24-27.
740. Vioque F, Dashti A, Santin M, Ruiz-Fons F, Koster PC, Hernandez-Castro C, Garcia JT, Bailo B, Ortega S, Olea PP, Arce F, Chicharro C, Nieto J, Gonzalez F, Vinuela J, Carmena D, Gonzalez-Barrio D (2022) Wild micromammal host spectrum of zoonotic eukaryotic parasites in Spain. Occurrence and genetic characterisation. *Transboundary and Emerging Diseases* 1-17.
741. Lux L, Ulrich RG, Santos-Silva S, Queirós J, Imholt C, Klotz C, Paupério J, Pita R, Vale-Gonçalves H, Alves PC, Mesquita JR (2023) Detection and molecular characterization of *Giardia* and *Cryptosporidium* spp. circulating in wild small mammals from Portugal. *Animals* 13: 515.
742. Krawczyk AI, Van Leeuwen AD, Jacobs-Reitsma W, Wijnands LM, Bouw E, Jahfari S, Van Hoek AHAM, Van der Giessen JWB, Roelfsema JH, Kroes M, Kleve J, Dullemont Y, Sprong H, De Bruin A (2015) Presence of zoonotic agents in engorged ticks and hedgehog faeces from Erinaceus europaeus in (sub) urban areas. *Parasites & Vectors* 8: 210.
743. Rasmussen SL, Hallig J, van Wijk RE, Petersen HH (2021) An investigation of endoparasites and the determinants of parasite infection in European hedgehogs (*Erinaceus europaeus*) from Denmark. *International Journal for Parasitology: Parasites and Wildlife* 16: 217-227.
744. Rego L, Castro-Scholten S, Cano C, Jimenez -Martin D, Koster PC, Caballero-Gomez J, Bailo B, Dashti A, Hernandez-Castro C, Cano-Terriza D, Vioque F, Maloney JG, Santin M, Garcia-Bocanegra I, Carmena D, Gonzalez-Barrio D (2023) Iberian wild leporidae as hosts of zoonotic enteroparasites in Mediterranean ecosystems of Southern Spain. *Zoonoses and Public Health* 70: 223-237.
745. Li TS, Zou Y, Peng JJ, Wang LQ, Zhang HS, Cong W, Zhu XQ, Sun XL (2020) Prevalence and genotype distribution of *Giardia duodenalis* in rabbits in Shandong province, eastern China. *BioMed Research International* Article ID: 4714735.
746. Zhang W, Shen Y, Wang R, Liu A, Ling H, Li Y, Cao J, Zhang X, Shu J, Zhang L (2012) *Cryptosporidium cuniculus* and *Giardia duodenalis* in rabbits: genetic diversity and possible zoonotic transmission. *PLoS ONE* 7: e31262.
747. Pantchev N, Broglia A, Paoletti B, Vrhovec MG, Bertram A, Nöckler K, Cacciò SM (2014) Occurrence and molecular typing of *Giardia* isolates in pet rabbits, chinchillas, guinea pigs and ferrets collected in Europe during 2006-2012. *Veterinary Record* 175: 18.
748. Qi M, Xi J, Li J, Wang H, Ning C, Zhang L (2015) Prevalence of zoonotic *Giardia duodenalis* assemblage B and first identification of assemblage E in rabbit fecal samples isolates from central China. *Journal of Eukaryotic Microbiology* 62: 810-814.
749. Mosallanejad B, Avizeh R, Razi Jalali MH (2016) Detection of *Giardia duodenalis* antigen in companion rabbits of Ahvaz district, South-West of Iran. *Iranian Journal of Veterinary Sciences and Technology* 8: 58-63.
750. Jiang J, Ma JG, Zhang MZ, Xu P, Hou G, Zhao Q, Zhang XX (2018) Prevalence and risk factors of *Giardia duodenalis* in domestic rabbbits (*Oryctolagus cuniculus*) in Jilin and Liaoning province, northeastern China. *Journal of Infection and Public Health* 11: 723-726.
751. Zhang X, Qi M, Jing B, Yu F, Wu Y, Chang Y, Zhao A, Wei Z, Dong H, Zhang L (2018) Molecular Characterization of *Cryptosporidium* spp., *Giardia duodenalis*, and *Enterocytozoon bieneusi* in Rabbits in Xinjiang, China. *Journal of Eukaryotic Microbiology* 65: 854-859.
752. Akinkuotu OA, Greenwood SJ, McClure JT, Takeet MI, Otesile EB, Olufemi F (2018) Multilocus genotyping of *Giardia duodenalis* infecting rabbits in Ogun State, Nigeria. *Veterinary Parasitology: Regional Studies and Reports* 13: 171-176.
753. Tang H, Ye Y, Kang R, Yu J, Cao Y (2021) Prevalence and multilocus genotyping of *Giardia duodenalis* in rabbits from Shaanxi province in northwestern China. *Parasite* 28: 54.
754. Baptista CB, Araujo MJ, Inacio SV, de Araujo Mendes BC, Costa de Aquino MC, Ferrari ED, Bresciani KDS, da Costa AJ (2023) First report of *Giardia* *duodenalis* in pet rabbits in Brazil. *Preventive Veterinary Medicine* 218: 105981.
755. Adams PJ, Monis PT, Elliot AD, Thompson RCA (2004) Cyst morphology and sequence analysis of the small subunit rDNA and *ef1*- identifies a novel *Giardia* genotype in a quenda (*Isoodon obesulus*) from Western Australia. *Infection, Genetics and Evolution* 4: 365-370.
756. Hillman A, Ash A, Elliot A, Lymbery A, Perez C, Thompson RCA (2016) Confirmation of a unique species of *Giardia*, parasitic in the quenda (*Isoodon obesulus*). International Journal for Parasitology: *Parasites and Wildlife* 5: 110-115.
757. Zhang XX, Zhang FK, Li FC, Hou JL, Zheng WB, Du SZ, Zhao Q, Zhu XQ (2017) The presence of *Giardia intestinalis* in donkeys, *Equus asinus*, in China. *Parasites & Vectors* 10: 3.
758. Mukbel RM, Ghaith AO, Abu Halaweh M, Abo-Shehada MN (2017) Prevalence of *Giardia* assemblages among equines in Jordan. *Journal of* *Equine Veterinary Science* 57: 1-7.
759. Li F, Wang R, Guo Y, Li N, Feng Y, Xiao L (2020) Zoonotic potential of *Enterocytozoon bieneusi* and *Giardia duodenalis* in horses and donkeys in northern China. *Parasitology Research* 119: 1101-1108.
760. Xu C, Tuo H, Wang W, Zhang Z, Yu F, Chuai L, Qi M, Jing B (2023) Occurrence and genetic characteristics of *Giardia duodenalis* in donkeys in Xinjiang, China. *Parasite* 30: 50.
761. Atwill ER, McDougald NK, Perea L (2000) Cross-sectional study of faecal shedding of *Giardia duodenalis* and *Cryptosporidium parvum* among packstock in the Sierra Nevada Range. *Equine Veterinary Journal* 32: 247-252.
762. Xiao L, Herd RP (1994) Epidemiology of equine *Cryptosporidium* and *Giardia* infections. *Equine Veterinary Journal* 26: 14-17.
763. Pavlasek I, Hess L, Stehlik I, Stika V (1995) The first detection of *Giardia* spp. in horses in the Czech Republic. *Veterinary Medicine* (*Praha*) 40: 81-86.
764. Beelitz P, Göbel E, Gothe R (1996) Spectrum of species and incidence of endoparasites in foals and their mother mares from breeding farms with and without anthelmintic prophylaxis in upper Bavaria. Tierarztliche Praxis 24: 48-54.
765. Johnson E, Atwill ER, Filkins ME, Kalush J (1997) The prevalence of shedding of *Cryptosporidium* and *Giardia* spp. based on a single fecal sample collection from each of 91 horses used for backcountry recreation. Journal of Veterinary Diagnostic Investigation 9: 56-60.
766. Forde KN, Swinker AM, Traub-Dargatz JL, Cheney JM (1998) The prevalence of *Cryptosporidium*/*Giardia* in the trial horse population utilizing public lands in Colorado. Journal of Equine Veterinary Science 18: 38-40.
767. Bray RE, Wickler SJ, Cogger EA, Atwill ER, London C, Gallino JL, Anderson TP (1998) Endoparasite infection and *Cryptosporidium*/*Giardia* in feral horses on public lands. *Journal of Equine Veterinary Science* 18: 41-43.
768. Gomes AD, Barretta C, Ziegler DP, Sausen L, Stoever N, Sangioni LA, Vogel FSF, Monteiro SG, Zanella A (2008) Prevalence of *Cryptosporidium* spp*. and Giardia* sp*.* infection in horses stabled in the Jockey Club of Santa Maria - RS, Brazil. *Ciência Rural* 38: 2662-2665.
769. Frederick J, Giguere S, Sanchez LC (2009) Infectious agents detected in the feces of diarrheic foals: A retrospective study of 223 cases (2003-2008). *Journal of Veterinary Internal Medicine* 23: 1254-1260.
770. De Souza PNB, Bomfim TCB, Huber F, Abboud LCS, Gomes RS (2009) Natural infection by *Cryptosporidium* sp., *Giardia* sp. and *Eimeria leuckarti* in three groups of equines with different handlings in Rio de Janeiro, Brazil. *Veterinary Parasitology* 160: 327-333.
771. Veronesi F, Passamonti F, Caccio S, Diaferia M, Piergili Fioretti D (2010) Epidemiological survey on equine *Cryptosporidium* and *Giardia* infections in Italy and molecular characterization of isolates. *Zoonoses and Public Health* 57: 510-517.
772. Butty ET (2011) Detection of *Cryptosporidium* and *Giardia* duodenalis in equines in Nineveh, Iraq. *Iraqi Journal of Veterinary Sciences* 25: 43-46.
773. Traversa D, Otranto D, Milillo P, Latrofa MS, Giangaspero A, Di Cesare A, Paoletti B (2012) *Giardia duodenalis* sub-assemblage of animal and human origin in horses. *Infection, Genetics and Evolution* 12: 1642-1646.
774. Ghadrdan-Mashhadi A, Hamidi NH, Alizadehnia P (2012) A study of the prevalence of giardiasis in horses in Ahvaz city. *Iranian Veterinary* *Journal* 7: 79-83.
775. Santin M, Cortes Vecino JA, Fayer R (2013) A large scale molecular study of *Giardia duodenalis* in horses from Colombia. *Veterinary Parasitology* 196: 31-36.
776. Qi M, Huan H, Wang H, Wang R, Xiao L, Arrowood MJ, Li J, Zhang L (2015) Molecular identification of *Cryptosporidium* spp. and *Giardia duodenalis* in grazing horses from Xinjiang, China. *Veterinary Parasitology* 209: 169-172.
777. Kostopoulou D, Casaert S, Tzanidakis N, van Doorn D, Demeler J, von Samson-Himmelstjerna G, Saratsis A, Voutzourakis N, Ehsan A, Doornaert T, Looijen M, De Wilde N, Sotiraki S, Claerebout E, Geurden T (2015) The occurrence and genetic characterization of *Cryptosporidium* and *Giardia* species in foals in Belgium, The Netherlands, Germany and Greece. *Veterinary Parasitology* 211: 170-174.
778. Jafari H, Razi Jalali MH, Seyfi Abad Shapouri M, Haji Hajikolaii MR (2016) Prevalence and genotyping of *Giardia duodenalis* among Arabian horses in Ahvaz, southwest of Iran. *Archives of Razi Institute* 71: 177-181.
779. Deng L, Li W, Zhong Z, Liu X, Chai Y, Luo X, Song Y, Wang W, Gong C, Huang X, Hu Y, Fu H, He M, Wang Y, Zhang Y, Wu K, Cao S, Peng G (2017) Prevalence and molecular characterization of *Giardia intestinalis* in racehorses from the Sichuan province of southwestern China. *PLoS ONE* 12: e0189728.
780. Demircan K, Onder Z, Duzlu O, Yildirim A, Okur M, Ciloglu A, Yetismis G, Inci A (2019) First Molecular detection and phylogenetic analyses of zoonotic *Giardia intestinalis* in horses in Turkey. *Journal of Equine Veterinary Science* 80: 56-60.
781. Qi M, Ji X, Zhang Y, Wei Z, Jing B, Zhang L, Lin X, Karim MdR, Wang H, Sun M (2020) Prevalence and multilocus analysis of *Giardia duodenalis* in racehorses in China. *Parasitology Research* 119: 483-490.
782. Carminatti A, Chitolina MB, Ribeiro AB, Forest M, Collet SG, Prestes AM, Camillo G (2023) Occurrence and risk factors associated with gastrointestinal parasitism in horses reared in different systems. *Veterinary Parasitology: Regional Studies and Reports* 42: 100890.
783. Dos Reis LL, de Souza LSS, Braga FCO, Lima DCS, Lima NAS, Padinha JDS, Nava AFD, Vicente ACP (2023) Zoonotic *Giardia duodenalis* assemblage A in northern sloth from Brazilian Amazon. *Memorias do Instituo Oswaldo Cruz* 118: e230088.
784. Kowalewski MM, Salazer JS, Deutsch JC, Rano M, Kuhlenschmidt MS, Gillespie TR (2011) Black and gold howler monkeys (*Alouatta caraya*) as sentinels of ecosystem health: patterns of zoonotic protozoa infection relative to degree of human-primate contact. *American Journal of Primatology* 73: 7583.
785. David EB, Patti M, Coradi ST, Oliveira-Sequeira TCG, Ribolla PEM, Guimaraes S (2014) Molecular typing of *Giardia duodenalis* isolates from nonhuman primates housed in a Brazilian zoo. *Revista Instituto de Medicina Tropical de Sao Paulo* 56: 49-54.
786. Volotao ACC, Souza Junior JC, Grassini C, Peralta JM, Fernandes O (2008) Genotyping of *Giardia duodenalis* from southern brown howler monkeys (*Alouatta clamitans*) from Brazil. *Veterinary Parasitology* 158: 133-137.
787. Vitazkova SK, Wade SE (2006) Parasites of free-ranging black howler monkeys (*Alouatta pigra*) from Belize and Mexico. *American Journal of Primatology* 68: 1089-1097.
788. Köster PC, Martínez-Nevado E, González A, Abelló-Poveda MT, Fernández-Bellon H, de la Riva-Fraga M, Marquet B, Guéry JP, Knauf-Witzens T, Weigold A, Dashti A, Bailo B, Imaña E, Muadica AS, González-Barrio D, Ponce-Gordo F, Calero-Bernal R, Carmena D (2022) Intestinal protists in captive non-human primates and their handlers in six european zoological gardens. molecular evidence of zoonotic transmission. *Frontiers in* *Veterinary Science* 8: 819887.
789. Levecke B, Dorny P, Geurden T, Vercammen F, Vercruysse J (2007) Gastrointestinal protozoa in non-human primates of four zoological gardens in Belgium. *Veterinary Parasitology* 148: 236-246.
790. Karim MdR, Zhang S, Jian F, Li J, Zhou C, Zhang L, Sun M, Yang G, Zou F, Dong H, Li J, Rume FI, Qi M, Wang R, Ning C, Xiao L (2014) Multilocus typing of *Cryptosporidium* spp. and *Giardia duodenalis* from non-human primates in China. *International Journal for Parasitology* 44: 1039-1047.
791. Karim MdR, Wang R, Yu F, Li T, Dong H, Li D, Zhang L, Li J, Jian F, Zhang S, Rume FI, Ning C, Xiao L (2015) Multilocus analysis of *Giardia duodenalis* from nonhuman primates kept in zoos in China: geographical segregation and host-adaptation of assemblage B isolates. *Infection,* *Genetics and Evolution* 30: 82-88.
792. Li M, Zhao B, Li B, Wang Q, Niu L, Deng J, Gu X, Peng X, Wang T, Yang G (2015) Prevalence of gastrointestinal parasites in captive non-human primates of twenty-four zoological gardens in China. *Journal of Medical Primatology* 44: 168-173.
793. Perea-Rodriguez JP, Milano AM, Osherov BE, Fernandez-Duque E (2010) Gastrointestinal parasites of owl monkeys (*Aotus Azarai Azarai*) in the Argentinean Chaco. *Neotropical Primates* 17: 7-11.
794. Köster PC, Dashti A, Bailo B, Muadica AS, Maloney JG, Santín M, Chicharro C, Migueláñez S, Nieto FJ, Cano-Terriza D, García-Bocanegra I, Guerra R, Ponce-Gordo F, Calero-Bernal R, González-Barrio D, Carmena D (2021) Occurrence and genetic diversity of protist parasites in captive non-human primates, zookeepers, and free-living sympatric rats in the Córdoba zoo conservation centre, Southern Spain. *Animals* 11: 700.
[truncated: 21,379 more chars]
