# Supplementary material for: Global prevalence of Giardia infection in nonhuman mammalian hosts: A systematic review and meta-analysis of five million animals
Source: PLoS Negl Trop Dis. 2025 Apr 24;19(4):e0013021. doi: 10.1371/journal.pntd.0013021 (PMC12052165; doi:10.1371/journal.pntd.0013021)
Supplement: S3 Table — (DOC) [file pntd.0013021.s004.doc]

**S3 Table.** Stratified prevalence of *Giardia duodenalis* infection in cattle according to *a priori* defined sub-groups.

| **Variables and subgroups** | **No. of dataset** | **Total**  **(*n*)** | **Pos.**  **(*n*)** | **Effect size**  **(95% CI)** | **POR**  **(95% CI)** | **Weight (%)** | **I^2^**^*^  **(%)** | **Q**^*^ |
| --- | --- | --- | --- | --- | --- | --- | --- | --- |
| **Species** |  |  |  |  |  |  |  |  |
| *Bos taurus* | 208 | 94,085 | 18,705 | 0.22 (0.21–0.24) | 5.35 (4.58–6.26) | 93.04 | 99.12 | 22276.15 |
| *Bos indicus* | 6 | 1,640 | 199 | 0.19 (0.10–0.28) | 2.98 (2.39–3.71) | 1.98 | 93.67 | 63.21 |
| *Bos grunniens* | 14 | 3,887 | 172 | 0.05 (0.03–0.07) | 1 | 4.98 | 85.47 | 68.81 |
| **Keeping status** |  |  |  |  |  |  |  |  |
| Farmed | 208 | 95,464 | 18,769 | 0.22 (0.20–0.24) | 5.47 (4.63–6.47) | 95.53 | 99.12 | 22207.10 |
| Free range | 10 | 3,528 | 151 | 0.05 (0.03–0.07) | 1 | 4.47 | 87.18 | 62.42 |
| **Type of farm** |  |  |  |  |  |  |  |  |
| Dairy | 85 | 53,413 | 11,941 | 0.25 (0.22–0.28) | 1.12 (1.06–1.18) | 74.66 | 99.36 | 13157.90 |
| Beef | 29 | 9,321 | 1,905 | 0.19 (0.14–0.23) | 1 | 25.34 | 97.57 | 1153.11 |
| **Age groups** |  |  |  |  |  |  |  |  |
| Pre-weaned ^**^ | 60 | 23,557 | 7,153 | 0.27 (0.23–0.31) | 4.99 (4.59–5.43) | 51.31 | 98.59 | 4196.50 |
| Post-weaned ^***^ | 27 | 8,413 | 1,316 | 0.15 (0.12–0.19) | 2.12 (1.92–2.34) | 23.42 | 98.49 | 1727.13 |
| Heifer ^****^ | 13 | 3,875 | 354 | 0.12 (0.07–0.16) | 1.15 (1.00–1.32) | 7.94 | 98.24 | 453.43 |
| Adult ^*****^ | 24 | 8,409 | 675 | 0.10 (0.08–0.12) | 1 | 17.33 | 97.56 | 778.33 |
| **Sex groups** |  |  |  |  |  |  |  |  |
| Female | 20 | 5,524 | 937 | 0.19 (0.14–0.25) | 1.08 (0.93–1.24) | 50.09 | 97.94 | 921.29 |
| Male | 15 | 1,954 | 310 | 0.17 (0.13–0.22) | 1 | 40.91 | 89.57 | 134.29 |
| **Clinical signs** |  |  |  |  |  |  |  |  |
| Diarrheic | 33 | 6,372 | 1,004 | 0.21 (0.17–0.25) | 1.15 (1.05–1.25) | 46.69 | 94.09 | 541.15 |
| Non-diarrheic | 35 | 12,970 | 1,810 | 0.18 (0.14–0.21) | 1 | 53.31 | 98.43 | 2039.04 |
| **Season** |  |  |  |  |  |  |  |  |
| Spring | 12 | 5,221 | 1,101 | 0.27 (0.17–0.36) | 2.23 (1.96–2.54) | 24.56 | 99.18 | 1339.15 |
| Summer | 17 | 3,876 | 621 | 0.25 (0.19–0.31) | 1.59 (1.38–1.83) | 31.06 | 98.27 | 868.06 |
| Autumn | 11 | 3,410 | 364 | 0.17 (0.12–0.23) | 1 | 21.72 | 97.23 | 361.27 |
| Winter | 11 | 4,516 | 511 | 0.17 (0.07–0.26) | 1.06 (0.92–1.23) | 22.66 | 99.50 | 1985.80 |

CI: confidence intervals; POR: prevalence odds ratios; I^2^ and Q: heterogeneity measures.

^*^*p*-value for heterogeneity in all sub-groups was statistically significant (*p* < 0.05).

^**^Age group ≤ 3 months

^***^Age group 3–12 months

^****^Age group 1–2 yeas

^*****^Age group > 2 years
