# Supplementary material for: Global prevalence of Giardia infection in nonhuman mammalian hosts: A systematic review and meta-analysis of five million animals
Source: PLoS Negl Trop Dis. 2025 Apr 24;19(4):e0013021. doi: 10.1371/journal.pntd.0013021 (PMC12052165; doi:10.1371/journal.pntd.0013021)
Supplement: S4 Table — (DOC) [file pntd.0013021.s005.doc]

**S4 Table.** Stratified prevalence for *Giardia duodenalis* infection in buffaloes according to *a priori* defined sub-groups.

| **Variables and subgroups** | **No. of dataset** | **Total (*n*)** | **Pos. (*n*)** | **Effect size**  **(95% CI)** | **POR**  **(95% CI)** | **Weight (%)** | **I^2^**^*^  **(%)** | **Q**^*^ |
| --- | --- | --- | --- | --- | --- | --- | --- | --- |
| **Species** |  |  |  |  |  |  |  |  |
| *Bubalis bubalis* | 23 | 4,845 | 645 | 0.12 (0.08–0.15) | 11.3 (1.96–455.4) | 83.63 | 96.86 | 636.10 |
| *Bison bison* | 2 | 123 | 25 | 0.20 (0.13–0.27) | 18.8 (2.92–785.1) | 6.11 | - | - |
| *Bison bonasus* | 3 | 78 | 6 | 0.08 (0.02–0.14) | 6.16 (0.71–287.3) | 5.69 | - | - |
| *Syncerus caffer* | 2 | 75 | 1 | 0.02 (0.00–0.10) | 1 | 4.57 | - | - |
| **Clinical signs** |  |  |  |  |  |  |  |  |
| Diarrheic | 4 | 340 | 96 | 0.21 (0.03–0.38) | 1.63 (1.22–2.17) | 25.75 | 95.39 | 65.03 |
| Non-diarrheic | 14 | 1,124 | 218 | 0.12 (0.06–0.17) | 1 | 74.25 | 90.12 | 121.48 |

CI: confidence intervals; POR: prevalence odds ratios; I^2^ and Q: heterogeneity measures.

^*^*p*-value for heterogeneity in all sub-groups was statistically significant (*p* < 0.05).
