# Supplementary material for: Global prevalence of Giardia infection in nonhuman mammalian hosts: A systematic review and meta-analysis of five million animals
Source: PLoS Negl Trop Dis. 2025 Apr 24;19(4):e0013021. doi: 10.1371/journal.pntd.0013021 (PMC12052165; doi:10.1371/journal.pntd.0013021)
Supplement: S5 Table — (DOC) [file pntd.0013021.s006.doc]

**S5 Table.** Stratified prevalence of *Giardia duodenalis* infection in sheep and goat according to *a priori* defined sub-groups.

| **Variables and subgroups** | **No. of dataset** | **Total**  **(*n*)** | **Pos.**  **(*n*)** | **Effect size**  **(95% CI)** | **POR**  **(95% CI)** | **Weight (%)** | **I^2^**^*^  **(%)** | **Q**^*^ |
| --- | --- | --- | --- | --- | --- | --- | --- | --- |
| **Species** |  |  |  |  |  |  |  |  |
| *Capra hircus* | 42 | 8,791 | 1,211 | 0.18 (0.15–0.21) | 1 | 42.09 | 97.63 | 1642.94 |
| *Ovis aries* | 59 | 15,311 | 2,285 | 0.21 (0.18–0.24) | 1.09 (1.01–1.18) | 57.91 | 98.25 | 3094.05 |
| **Keeping status** |  |  |  |  |  |  |  |  |
| Free range | 12 | 4,028 | 225 | 0.06 (0.03–0.08) | 1 | 14.00 | 94.12 | 170.03 |
| Farmed | 80 | 19,090 | 3,147 | 0.22 (0.20–0.25) | 3.33 (2.89–3.84) | 86.00 | 98.48 | 4873.53 |
| **Age groups** |  |  |  |  |  |  |  |  |
| Pre-weaned ^**^ | 23 | 2,856 | 637 | 0.28 (0.19–0.37) | 2.77 (2.41–3.17) | 35.75 | 98.27 | 1215.42 |
| Post-weaned ^***^ | 19 | 2,826 | 443 | 0.16 (0.12–0.21) | 1.79 (1.55–2.07) | 32.11 | 95.86 | 434.82 |
| Adult ^****^ | 19 | 4,380 | 411 | 0.10 (0.07–0.13) | 1 | 32.14 | 94.71 | 321.07 |
| **Sex groups** |  |  |  |  |  |  |  |  |
| Female | 11 | 2,300 | 213 | 0.12 (0.08–0.17) | 1 | 55.58 | 94.90 | 196.25 |
| Male | 9 | 1,830 | 172 | 0.12 (0.07–0.17) | 1.01 (0.81–1.26) | 44.42 | 95.06 | 161.99 |
| **Clinical signs** |  |  |  |  |  |  |  |  |
| Diarrheic | 6 | 579 | 196 | 0.39 (0.16–0.63) | 2.91 (2.42–3.51) | 12.99 | 98.39 | 310.90 |
| Non-diarrheic | 32 | 8,916 | 1,330 | 0.19 (0.15–0.22) | 1 | 87.01 | 97.72 | 1357.45 |

CI: confidence intervals; POR: prevalence odds ratios; I^2^ and Q: heterogeneity measures.

^*^*p*-value for heterogeneity in all sub-groups was significant (*p* < 0.05).

^**^Age group ≤ 3 months

^***^Age group 3–12 months

^****^Age group > 1 year
