# Supplementary material for: Global prevalence of Giardia infection in nonhuman mammalian hosts: A systematic review and meta-analysis of five million animals
Source: PLoS Negl Trop Dis. 2025 Apr 24;19(4):e0013021. doi: 10.1371/journal.pntd.0013021 (PMC12052165; doi:10.1371/journal.pntd.0013021)
Supplement: S6 Table — (DOC) [file pntd.0013021.s007.doc]

**S6 Table.** Stratified prevalence of *Giardia duodenalis* infection in camels and alpacas according to *a priori* defined sub-groups.

| Variables and subgroups | No. of dataset | Total  (*n*) | Pos. (*n*) | Effect size  (95% CI) | POR  (95% CI) | Weight (%) | I^2*^  (%) | Q^*^ |
| --- | --- | --- | --- | --- | --- | --- | --- | --- |
| **Species** |  |  |  |  |  |  |  |  |
| *Camelus bactrianus* | 4 | 1,011 | 90 | 0.06 (0.00–0.11) | 1.52 (0.95–2.46) | 22.32 | 89.05 | 27.41 |
| *Camelus dromedarious* | 9 | 1,578 | 107 | 0.12 (0.06–0.17) | 1.13 (0.72–1.84) | 37.91 | 88.08 | 50.34 |
| *Lama glama* | 4 | 432 | 26 | 0.12 (0.01–0.23) | 1 | 15.40 | 76.56 | 12.80 |
| *Lama pacous* | 6 | 2,146 | 193 | 0.17 (0.01–0.33) | 1.54 (1.00–2.45) | 24.38 | 98.75 | 319.80 |
| **Geographical distribution** |  |  |  |  |  |  |  |  |
| Old world camelids**^a^** | 13 | 2,589 | 197 | 0.09 (0.06–0.13) | 1 | 60.22 | 87.51 | 80.04 |
| New world camelids**^b^** | 10 | 2,578 | 219 | 0.15 (0.08–0.23) | 1.12 (0.91–1.38) | 39.78 | 97.68 | 344.89 |
| **Age groups** |  |  |  |  |  |  |  |  |
| ≤ 1 year | 6 | 453 | 166 | 0.24 (0.09–0.40) | 8.82 (6.57–11.80) | 42.34 | 93.19 | 73.40 |
| > 1 year | 11 | 1,496 | 92 | 0.09 (0.04–0.13) | 1 | 57.66 | 84.40 | 44.87 |

CI: confidence intervals; POR: prevalence odds ratios; I^2^ and Q: heterogeneity measures.

^*^*p*-value for heterogeneity in all sub-groups was significant (*p* < 0.05).

^**^Age group ≤ 3 months

^***^Age group 3–12 months

^****^Age group > 1 year

^a^ *Lama* sp.

^b^ *Camelus* sp.
