# Supplementary material for: Global prevalence of Giardia infection in nonhuman mammalian hosts: A systematic review and meta-analysis of five million animals
Source: PLoS Negl Trop Dis. 2025 Apr 24;19(4):e0013021. doi: 10.1371/journal.pntd.0013021 (PMC12052165; doi:10.1371/journal.pntd.0013021)
Supplement: S7 Table — (DOC) [file pntd.0013021.s008.doc]

**S7 Table.** Stratified prevalence of *Giardia duodenalis* infection in domestic and feral pigs according to *a priori* defined sub-groups.

| **Variables and subgroups** | **No. of dataset** | **Total (*n*)** | **Pos.**  **(*n*)** | **Effect size**  **(95% CI)** | **POR**  **(95% CI)** | **Weight (%)** | **I^2^**^*^  **(%)** | **Q**^*^ |
| --- | --- | --- | --- | --- | --- | --- | --- | --- |
| **Species** |  |  |  |  |  |  |  |  |
| *Sus scrofa domesticus***^a^** | 33 | 10,141 | 1,033 | 0.11 (0.09–0.14) | 1.42 (1.22–1.65) | 71.68 | 97.10 | 930.79 |
| *Sus scrofa***^b^** | 12 | 3,106 | 229 | 0.07 (0.04–0.10) | 1 | 28.32 | 94.99 | 179.57 |
| **Age groups** |  |  |  |  |  |  |  |  |
| Pre-weaned piglets ^**^ | 7 | 934 | 98 | 0.18 (0.07–0.29) | 1.56 (1.20–2.03) | 13.25 | 96.22 | 105.86 |
| Post-weaned pigs ^***^ | 8 | 1,410 | 163 | 0.13 (0.07–0.18) | 1.74 (1.39–2.18) | 24.18 | 95.92 | 171.36 |
| Fatteninig pigs ^****^ | 6 | 667 | 52 | 0.08 (0.06–0.11) | 1.13 (0.80–1.56) | 14.11 | 0.00 | 3.90 |
| Adult pigs ^*****^ | 17 | 2,934 | 204 | 0.07 (0.04–0.09) | 1 | 48.46 | 90.29 | 164.78 |
| **Sex groups** |  |  |  |  |  |  |  |  |
| Female | 8 | 1,213 | 70 | 0.06 (0.03–0.09) | 1 | 50.68 | 88.35 | 51.51 |
| Male | 8 | 1,220 | 83 | 0.07 (0.03–0.10) | 1.19 (0.84–1.68) | 49.32 | 90.29 | 61.80 |

CI: confidence intervals, POR: prevalence odds ratios; I^2^ and Q: heterogeneity measures.

^*^*p*-value for heterogeneity in all sub-groups was significant (*p* < 0.05).

^**^Age group ≤ 1 month

^***^Age group 1–4 months

^****^Age group 4–6 months

^*****^Age group > 6 months.

^a^ Domestic pig.

^b^ Wild boar.
