# Supplementary material for: Global prevalence of Giardia infection in nonhuman mammalian hosts: A systematic review and meta-analysis of five million animals
Source: PLoS Negl Trop Dis. 2025 Apr 24;19(4):e0013021. doi: 10.1371/journal.pntd.0013021 (PMC12052165; doi:10.1371/journal.pntd.0013021)
Supplement: S8 Table — (DOC) [file pntd.0013021.s009.doc]

**S8 Table.** Stratified prevalence of *Giardia duodenalis* infection in horses and donkeys according to *a priori* defined sub-groups.

| **Variables and subgroups** | **No. of dataset** | **Total**  **(*n*)** | **Pos. (*n*)** | **Effect size**  **(95% CI)** | **POR**  **(95% CI)** | **Weight (%)** | **I^2^**^*^  **(%)** | **Q**^*^ |
| --- | --- | --- | --- | --- | --- | --- | --- | --- |
| **Species** |  |  |  |  |  |  |  |  |
| *Equus asinus***^a^** | 7 | 920 | 105 | 0.12 (0.08–0.15) | 6.82 (5.39–8.57) | 13.02 | 52.08 | 6.26 |
| *Equus ferus caballus***^b^** | 39 | 22,895 | 424 | 0.09 (0.07–0.11) | 1 | 86.98 | 92.11 | 354.98 |
| **Keeping status** |  |  |  |  |  |  |  |  |
| Breeding farm | 19 | 4,108 | 414 | 0.10 (0.07–0.14) | 1.58 (1.03–2.53) | 57.95 | 95.24 | 336.46 |
| Racing | 7 | 1,837 | 170 | 0.13 (0.08–0.19) | 1.44 (0.91–2.34) | 24.43 | 96.10 | 153.75 |
| Riding | 4 | 529 | 40 | 0.07 (0.05–0.10) | 1.15 (0.66–2.04) | 7.18 | – | – |
| Working | 3 | 363 | 24 | 0.07 (0.02–0.13) | 1 | 10.45 | – | – |
| **Age groups** |  |  |  |  |  |  |  |  |
| Young (≤ 1 year) | 14 | 2,048 | 258 | 0.13 (0.08–0.17) | 1.67 (1.37–2.04) | 45.77 | 91.40 | 127.98 |
| Adult (> 1 year) | 13 | 2,603 | 206 | 0.09 (0.06–0.12) | 1 | 54.23 | 91.98 | 149.61 |
| **Sex groups** |  |  |  |  |  |  |  |  |
| Stallion | 5 | 633 | 56 | 0.09 (0.05–0.12) | 1 | 44.42 | 54.00 | 8.70 |
| Mare | 5 | 938 | 89 | 0.10 (0.05–0.14) | 1.08 (0.75–1.56) | 55.58 | 82.69 | 23.11 |

CI: confidence intervals; POR: prevalence odds ratios; I^2^ and Q: heterogeneity measures.

^*^*p*-value for heterogeneity in all sub-groups was significant (*p* < 0.05).

^a^ Donkey.

^b^ Horse.
