# Supplementary material for: Global prevalence of Giardia infection in nonhuman mammalian hosts: A systematic review and meta-analysis of five million animals
Source: PLoS Negl Trop Dis. 2025 Apr 24;19(4):e0013021. doi: 10.1371/journal.pntd.0013021 (PMC12052165; doi:10.1371/journal.pntd.0013021)
Supplement: S9 Table — (DOC) [file pntd.0013021.s010.doc]

**S9 Table.** Stratified prevalence of *Giardia duodenalis* infection in wild and domestic canids according to *a priori* defined sub-groups.

| **Variables and subgroups** | **No. of dataset** | **Total**  **(*n*)** | **Pos.**  **(*n*)** | **Effect size**  **(95% CI)** | **POR**  **(95% CI)** | **Weight (%)** | **I^2^**^*^  **(%)** | **Q**^*^ |
| --- | --- | --- | --- | --- | --- | --- | --- | --- |
| **Species** |  |  |  |  |  |  |  |  |
| *Canis familiaris***^a^** | 270 | 4,399,339 | 118,949 | 0.13 (0.13–0.14) | 1 | 93.81 | 99.73 | 97087.05 |
| *Vulpes* sp.**^b^** | 17 | 1,709 | 188 | 0.12 (0.09–0.16) | 4.44 (3.81–5.18) | 3.72 | 86.61 | 97.07 |
| *Canis lupus* | 11 | 414 | 58 | 0.16 (0.07–0.25) | 5.86 (4.36–7.75) | 1.10 | 81.47 | 37.77 |
| *Canis latrans* | 5 | 402 | 47 | 0.15 (0.07–0.22) | 4.76 (3.43–6.47) | 1.00 | 79.80 | 19.80 |
| *Lycaon pictus* | 4 | 94 | 32 | 0.37 (0.21–0.53) | 18.5 (11.7–28.9) | 0.21 | 61.18 | 7.73 |
| *Canis f. dingo* | 1 | 44 | 13 | 0.30 (0.18–0.44) | 15.1 (7.24–29.7) | 0.09 | - | - |
| *Canis aureus* | 2 | 20 | 4 | 0.18 (0.02–0.35) | 8.99 (2.18–27.8) | 0.07 | - | - |
| **Origin** |  |  |  |  |  |  |  |  |
| Domestic | 262 | 4,393,223 | 118,400 | 0.13 (0.13–0.14) | 1 | 94.97 | 99.74 | 96538.07 |
| Wild | 32 | 2,268 | 281 | 0.15 (0.12–0.19) | 5.10 (4.49–5.79) | 5.03 | 83.28 | 149.53 |
| **Keeping status^a^** |  |  |  |  |  |  |  |  |
| Pet dog**^c^** | 171 | 4,340,372 | 112,143 | 0.11 (0.10–0.11) | 1 | 62.00 | 99.81 | 88305.78 |
| Sheltered dog**^d^** | 89 | 31,357 | 3,306 | 0.18 (0.16–0.19) | 4.44 (4.28–4.60) | 26.04 | 97.92 | 4087.72 |
| Breeding dog | 33 | 6.024 | 1,353 | 0.29 (0.24–0.35) | 10.9 (10.2–11.6) | 6.74 | 96.99 | 963.69 |
| Hunting dog**^e^** | 14 | 1,132 | 153 | 0.21 (0.12–0.29) | 5.89 (4.94–7.01) | 2.81 | 96.33 | 300.13 |
| Shepherd dog**^f^** | 10 | 1,098 | 90 | 0.12 (0.07–0.16) | 3.36 (2.68–4.18) | 2.40 | 88.86 | 80.77 |
| **Sex group^a^** |  |  |  |  |  |  |  |  |
| Mele | 55 | 1,194,997 | 7,734 | 0.16 (0.13–0.19) | 1.04 (1.01–1.08) | 53.87 | 98.42 | 3410.15 |
| Female | 55 | 1,301,160 | 8,030 | 0.17 (0.14–0.20) | 1 | 46.13 | 98.14 | 2855.36 |
| **Age groups^a^** |  |  |  |  |  |  |  |  |
| < 1 year | 98 | 1,260,523 | 30,765 | 0.23 (0.21–0.25) | 3.44 (3.36–3.52) | 41.55 | 99.66 | 26714.71 |
| ≥ 1 year | 85 | 1,314,801 | 9,469 | 0.04 (0.04–0.04) | 1 | 58.45 | 98.64 | 5812.10 |
| **Clinical signs^a^** |  |  |  |  |  |  |  |  |
| Diarrheic | 44 | 35,066 | 6,294 | 0.25 (0.22–0.28) | 2.88 (2.80–2.97) | 39.19 | 96.81 | 1347.38 |
| Non-diarrheic | 56 | 538,763 | 37,950 | 0.15 (0.13–0.17) | 1 | 60.81 | 96.71 | 1641.33 |

CI: confidence intervals; POR: prevalence odds ratios; I^2^ and Q: heterogeneity measures.

^*^*p*-value for heterogeneity in all sub-groups was significant (*p* < 0.05).

^a^ Domestic dog.

^b^ *Vulpes vulpes* and *Vulpes lagopus*.

^c^ Dogs in household environments and/or attended at veterinary clinics.

^d^ Stray and unwanted dogs.

^e^ Hunting, police, military, guard, racing, and sled dogs.

^f^ Shepherd and farm dogs.
