# Supplementary material for: Global prevalence of Giardia infection in nonhuman mammalian hosts: A systematic review and meta-analysis of five million animals
Source: PLoS Negl Trop Dis. 2025 Apr 24;19(4):e0013021. doi: 10.1371/journal.pntd.0013021 (PMC12052165; doi:10.1371/journal.pntd.0013021)
Supplement: S10 Table — (DOC) [file pntd.0013021.s011.doc]

**S10 Table.** Stratified prevalence of *Giardia duodenalis* infection in domestic, captive and wild felids according to *a priori* defined sub-groups.

| **Variables and subgroups** | **No. of dataset** | **Total**  **(*n*)** | **Pos. (*n*)** | **Effect size**  **(95% CI)** | **POR**  **(95% CI)** | **Weight (%)** | **I^2*^ (%)** | **Q^*^** |
| --- | --- | --- | --- | --- | --- | --- | --- | --- |
|  |  |  |  |  |  |  |  |  |
| **Size of felids** |  |  |  |  |  |  |  |  |
| Small**^a^** | 149 | 277,104 | 7,096 | 0.08 (0.08–0.09) | 1 | 98.41 | 97.88 | 6657.10 |
| Medium**^b^** and large**^c^** | 16 | 244 | 17 | 0.12 (0.03–0.21) | 2.84 (1.63–4.66) | 1.59 | 61.33 | 18.10 |
| **Origin** |  |  |  |  |  |  |  |  |
| Domestic | 143 | 276,878 | 7,071 | 0.08 (0.08–0.09) | 1 | 96.39 | 97.96 | 6629.38 |
| Captive | 15 | 305 | 9 | 0.01 (0.00–0.03) | 1.16 (0.52–2.23) | 2.99 | 0.00 | 6.50 |
| Wild | 7 | 165 | 33 | 0.20 (0.14–0.26) | 8.22 (3.70–20.0) | 0.62 | 0.00 | 1.83 |
| **Keeping status^d^** |  |  |  |  |  |  |  |  |
| Pet cats | 99 | 265,049 | 6,118 | 0.08 (0.08–0.09) | 1 | 68.63 | 98.35 | 5513.56 |
| Sheltered cats | 50 | 9,008 | 710 | 0.09 (0.08–0.11) | 3.62 (3.33–3.92) | 30.26 | 91.87 | 541.52 |
| Breeding cats | 8 | 297 | 93 | 0.42 (0.25–0.59) | 19.3 (14.9–24.8) | 1.11 | 89.88 | 69.14 |
| **Sex categories^d^** |  |  |  |  |  |  |  |  |
| Mele | 25 | 108,948 | 1,407 | 0.14 (0.11–0.18) | 1 | 49.68 | 97.68 | 993.02 |
| Female | 25 | 110,917 | 1,436 | 0.13 (0.10–0.17) | 1.00 (0.93–1.08) | 50.32 | 97.82 | 1052.85 |
| **Age categories^d^** |  |  |  |  |  |  |  |  |
| < 1 year | 38 | 153,592 | 1,744 | 0.04 (0.03–0.05) | 1 | 50.89 | 97.05 | 1152.25 |
| ≥ 1 year | 30 | 76,360 | 871 | 0.03 (0.03–0.04) | 1.00 (0.92–1.09) | 49.11 | 96.24 | 771.41 |
| **Clinical signs^d^** |  |  |  |  |  |  |  |  |
| Diarrheic | 29 | 14,615 | 2,146 | 0.18 (0.14–0.21) | 1.49 (1.36–1.64) | 44.80 | 96.96 | 855.91 |
| Non-diarrheic | 30 | 6,091 | 628 | 0.10 (0.08–0.12) | 1 | 55.20 | 94.74 | 550.91 |

CI: confidence intervals; POR: prevalence odds ratios; I^2^ and Q: heterogeneity measures.

^*^*p*-value for heterogeneity in all sub-groups was significant (*p* < 0.05).

^a^ Small-sized cats (*Felis catus*, *Felis silvestris*, *Leopardus colocola*, and *Leopardus* *geoffroyi*).

^b^ Medium-sized cats (*Leptailurus serval, Lynx lynx, Lynx pardinus*, and *Prionailurus viverrinus*).

^c^ Large-sized cats (*Panthera leo*, *Panthera onca*, *Panthera pardus*, *Panthera tigris*, and *Puma concolor*)

^d^ Status, sex, age group, and clinical signs refer to domestic cats (*Felis catus*) only.
