# Supplementary material for: Global prevalence of Giardia infection in nonhuman mammalian hosts: A systematic review and meta-analysis of five million animals
Source: PLoS Negl Trop Dis. 2025 Apr 24;19(4):e0013021. doi: 10.1371/journal.pntd.0013021 (PMC12052165; doi:10.1371/journal.pntd.0013021)
Supplement: S11 Table — (DOC) [file pntd.0013021.s012.doc]

**S11 Table.** Worldwide occurrence of *Giardia* species or genotypes in domestic and wild populations of non-human mammalian species.

| Host species | Country | Isolates (*n*) | *Giardia* species (*n*) | *G. duodenalis* assemblages (*n*) | References |
| --- | --- | --- | --- | --- | --- |
| Order Artiodactyla |  |  |  |  |  |
| Family Bovidae |  |  |  |  |  |
| Subfamily Bovinae |  |  |  |  |  |
| *Bison bison* | Belgium | 13 | *G. duodenalis* | A (5); E (7) | [1] |
| *Bison bonasus* | Croatia | 1 | *G. duodenalis* | C (1) | [2] |
| *Bos grunniens* | China | 149 | *G. duodenalis* | A (6); B (9); D (1); E (132); A/E (1) | [3–11] |
| *Bos taurus* | Algeria | 28 | *G. duodenalis* | A (14); E (6); A/E (8) | [12] |
| *Bos taurus* | Argentina | 18 | *G. duodenalis* | A (17); E (1) | [13] |
| *Bos taurus* | Australia | 349 | *G. duodenalis* (348); *G. peramelis* (1) | A (60); B (47); E (237); A/E (2); B/E (1); A/B/E (1) | [14–19] |
| *Bos taurus* | Austria | 31 | *G. duodenalis* | A (1); E (30) | [20] |
| *Bos taurus* | Bangladesh | 94 | *G. duodenalis* | A (14); B (5); E (75) | [21, 22] |
| *Bos taurus* | Belgium | 120 | *G. duodenalis* | A (43); E (77) | [23] |
| *Bos taurus* | Brazil | 37 | *G. duodenalis* | A (4); E (33) | [24–26] |
| *Bos taurus* | Canada | 449 | *G. duodenalis* | A (15); B (45); E (384); A/E (5) | [14, 27–32] |
| *Bos taurus* | China | 2,389 | *G. duodenalis* | A (97); B (23); D (1); E (2,244); A/E (24) | [9, 10, 33–57] |
| *Bos taurus* | Egypt | 125 | *G. duodenalis* | A (18); B (20); E (86); A/E (1) | [58–61] |
| *Bos taurus* | Ethiopia | 36 | *G. duodenalis* | A (4); B (1); E (31) | [62, 63] |
| *Bos taurus* | France | 26 | *G. duodenalis* | A (16); E (10) | [64] |
| *Bos taurus* | Germany | 137 | *G. duodenalis* | A (19); E (117); A/E (1) | [64, 65] |
| *Bos taurus* | India | 22 | *G. duodenalis* | A (5); E (14); A/E (3) | [66] |
| *Bos taurus* | Iran | 23 | *G. duodenalis* | A (4); E (19) | [67] |
| *Bos taurus* | Iraq | 103 | *G. duodenalis* | A (47); B (51); A/B (5) | [68–70] |
| *Bos taurus* | Italy | 43 | *G. duodenalis* | A (19); B (5); E (19) | [64, 71, 72] |
| *Bos taurus* | Mexico | 5 | *G. duodenalis* | A (1); A/B (2); B/E (2) | [73] |
| *Bos taurus* | New Zealand | 99 | *G. duodenalis* | A (63); B (27); E (9) | [74–76] |
| *Bos taurus* | Poland | 4 | *G. duodenalis* | A (1); E (3) | [77] |
| *Bos taurus* | Portugal | 15 | *G. duodenalis* | A (2); B (1); E (12) | [78, 79] |
| *Bos taurus* | Romania | 2 | *G. duodenalis* | A (2) | [80] |
| *Bos taurus* | Scotland | 66 | *G. duodenalis* | A (3); B (12); E (51) | [81] |
| *Bos taurus* | South Korea | 112 | *G. duodenalis* | A (11); E (99); A/E (2) | [82–86] |
| *Bos taurus* | Sri Lanka | 141 | *G. duodenalis* | A (4); E (137) | [87] |
| *Bos taurus* | Taiwan | 35 | *G. duodenalis* | A (2); D (1); E (32) | [88, 89] |
| *Bos taurus* | Thailand | 54 | *G. duodenalis* | A (12); E (42) | [90] |
| *Bos taurus* | Turkey | 222 | *G. duodenalis* | A (96); B (1); E (125) | [91–94] |
| *Bos taurus* | Uganda | 8 | *G. duodenalis* | A (6); E (2) | [95–97] |
| *Bos taurus* | UK | 77 | *G. duodenalis* | A (20); C (2); D (4); E (43); A/C (1); A/D (1); A/E (6) | [64, 98] |
| *Bos taurus* | USA | 2,721 | *G. duodenalis* | A (413); C (15); E (2,266); A/E (21); A/E/F (6) | [99–110] |
| *Bos taurus* | Vietnam | 27 | *G. duodenalis* | A (2); E (25) | [111, 112] |
| *Bos taurus indicus* | Ethiopia | 25 | *G. duodenalis* | A (1); E (24) | [62] |
| *Bos taurus indicus* | India | 5 | *G. duodenalis* | A (3); E (1); A/E (1) | [113] |
| *Bos taurus indicus* | Tanzania | 4 | *G. duodenalis* | A (3); B (1) | [114] |
| **Bubalus bubalis** | Australia | 68 | *G. duodenalis* | A (62); E (6) | [115, 116] |
| **Bubalus bubalis** | Brazil | 6 | *G. duodenalis* | E (6) | [117] |
| **Bubalus bubalis** | Egypt | 8 | *G. duodenalis* | A (2); E (6) | [60] |
| **Bubalus bubalis** | India | 9 | *G. duodenalis* | A (1); B (2); E (6) | [118] |
| **Bubalus bubalis** | Italy | 8 | *G. duodenalis* | A (2); E (6) | [119] |
| **Bubalus bubalis** | Romania | 1 | *G. duodenalis* | E (1) | [120] |
| **Bubalus bubalis** | Sri Lanka | 2 | *G. duodenalis* | E (2) | [87] |
| **Bubalus bubalis** | Thailand | 2 | *G. duodenalis* | D (1); E (1) | [121] |
| **Bubalus bubalis** | Turkey | 4 | *G. duodenalis* | B (2); E (2) | [122] |
| **Bubalus bubalis** | Vietnam | 3 | *G. duodenalis* | A (2); E (1) | [112] |
| **Syncerus caffer** | Croatia | 1 | *G. duodenalis* | D (1) | [2] |
| Subfalimy Caprinae |  |  |  |  |  |
| *Budorcas taxicolor bedfordi* | China | 17 | *G. duodenalis* | B (3); E (14) | [123] |
| *Capra aegagrus hircus* | C.A.R. | 1 | *G. duodenalis* | E (1) | [124] |
| *Capra hircus* | Australia | 1 | *G. duodenalis* | C (1) | [17] |
| *Capra hircus* | Bangladesh | 3 | *G. duodenalis* | E (3) | [125] |
| *Capra hircus* | Belgium | 28 | *G. duodenalis* | A (6); E (12); A/E (5); unknown (5) | [126] |
| *Capra hircus* | Brazil | 8 | *G. duodenalis* | E (8) | [127] |
| *Capra hircus* | China | 311 | *G. duodenalis* | A (11); E (297); A/E (2); B/E (1) | [9, 53, 128–135] |
| *Capra hircus* | Côte d’Ivoire | 1 | *G. duodenalis* | A/B (1) | [136] |
| *Capra hircus* | Ethiopia | 6 | *G. duodenalis* | E (6) | [63] |
| *Capra hircus* | Ghana | 3 | *G. duodenalis* | E (3) | [137] |
| *Capra hircus* | India | 28 | *G. duodenalis* | A (10); B (9); D (1); E (8) | [138] |
| *Capra hircus* | Iran | 16 | *G. duodenalis* | E (16) | [139, 140] |
| *Capra hircus* | Malaysia | 16 | *G. duodenalis* | A (3); B (1); E (12) | [141] |
| *Capra hircus* | Nigeria | 48 | *G. duodenalis* | E (40); A/E (5); B/E (3) | [142] |
| *Capra hircus* | Spain | 32 | *G. duodenalis* | E (32) | [143, 144] |
| *Capra hircus* | Tanzania | 9 | *G. duodenalis* | A (1); B (2); E (6) | [114] |
| *Capra hircus* | Turkey | 24 | *G. duodenalis* | A (24) | [92] |
| *Ovibos muschatus* | Canada | 4 | *G. duodenalis* | A (4) | [145] |
| *Ovibos muschatus* | Greenland | 1 | *G. duodenalis* | A (1) | [146] |
| *Ovis aries* | Australia | 141 | *G. duodenalis* | A (20); E (108); A/E (11); unknown (2) | [147–149] |
| *Ovis aries* | Belgium | 8 | *G. duodenalis* | A (2); E (4); A/E (2) | [126] |
| *Ovis aries* | Brazil | 50 | *G. duodenalis* | E (50) | [25, 150] |
| *Ovis aries* | China | 745 | *G. duodenalis* | A (88); B (2); E (630); A/E (25) | [4, 9, 53, 55, 128, 132, 133, 151–159] |
| *Ovis aries* | Ethiopia | 22 | *G. duodenalis* | E (22) | [63, 160] |
| *Ovis aries* | Ghana | 12 | *G. duodenalis* | E (12) | [137] |
| *Ovis aries* | Iran | 23 | *G. duodenalis* | E (23) | [139, 140] |
| *Ovis aries* | Portugal | 2 | *G. duodenalis* | E (2) | [79] |
| *Ovis aries* | Spain | 107 | *G. duodenalis* | A (59); E (21); A/E (27) | [161] |
| *Ovis aries* | Turkey | 21 | *G. duodenalis* | A (21) | [92, 162] |
| *Ovis aries* | UK | 24 | *G. duodenalis* | A (4); A/D (1); A/E (19) | [98] |
| *Ovis gmelini* | Spain | 1 | *G. duodenalis* | E (1) | [163] |
| *Ovis* sp. | China | 2 | *G. duodenalis* | E (1); C (1) | [5] |
| *Rupicapra pyrenaica* | Italy | 1 | *G. duodenalis* | A (1) | [164] |
| *Rupicapra pyrenaica* | Spain | 1 | *G. duodenalis* | E (1) | [163] |
| *Rupicapra rupicapra* | Italy | 12 | *G. duodenalis* | A (6); E (5); A/E (1) | [164, 165] |
| Subfamily Hippotraginae |  |  |  |  |  |
| *Oryx dammah* | Croatia | 1 | *G. duodenalis* | A (1) | [2] |
| *Oryx gazella* | China | 1 | *G. duodenalis* | E (1) | [166] |
| *Oryx leucoryx* | Belgium | 1 | *G. duodenalis* | A (1) | [1] |
| Subfamily Reduncinae |  |  |  |  |  |
| *Kobus ellipsiprymnus* | Bangladesh | 1 | *G. duodenalis* | A (1) | [167] |
| Family Cervidae |  |  |  |  |  |
| *Axis axis* | Bangladesh | 1 | *G. duodenalis* | A (1) | [167] |
| *Capreolus capreolus* | Croatia | 5 | *G. duodenalis* (4); *G. microti* (1) | A (2); D (2) | [168] |
| *Capreolus capreolus* | Poland | 10 | *G. duodenalis* | A (2); B (8) | [169, 170] |
| *Capreolus capreolus* | Spain | 7 | *G. duodenalis* | A (7) | [171] |
| *Cervus canadensis* | Belgium | 1 | *G. duodenalis* | A (1) | [1] |
| *Cervus elaphus* | China | 7 | *G. duodenalis* | A (1); E (6) | [55] |
| *Cervus elaphus* | Croatia | 4 | *G. duodenalis* | A (3); D (1) | [168] |
| *Cervus elaphus* | Poland | 5 | *G. duodenalis* | A (1); B (4) | [169, 170] |
| *Cervus elaphus* | Portugal | 1 | *G. duodenalis* | A (1) | [79] |
| *Cervus elaphus* | Spain | 3 | *G. duodenalis* | B (1); E (2) | [163] |
| *Cervus eldii* | China | 1 | *G. duodenalis* | E (1) | [172] |
| *Cervus nippon* | China | 16 | *G. duodenalis* | A (2); E (14) | [52, 55, 172, 173] |
| *Cervus nippon* | Japan | 2 | *G. duodenalis* | A (2) | [174] |
| *Dama* *dama* | Italy | 8 | *G. duodenalis* | A (8) | [175] |
| *Elaphurus davidianus* | China | 6 | *G. duodenalis* | E (6) | [166] |
| *Moschus* *berezovskii* | China | 5 | *G. duodenalis* | A (2); E (3) | [176] |
| *Moschus* *chrysogaster* | China | 39 | *G. duodenalis* | A (26); E (7); A/E (6) | [177] |
| *Odocoileus virginianus* | USA | 2 | *G. duodenalis* | A (2) | [178, 179] |
| *Rangifer tarandus* | China | 9 | *G. duodenalis* | A (7); E (2) | [55] |
| ***Rusa unicolor*** | Australia | 39 | *G. duodenalis* | A (28); B (1); E (7); A/B (1); A/E (2) | [17, 180] |
| Family Giraffidae |  |  |  |  |  |
| *Giraffa camelopardalis* | Bangladesh | 1 | *G. duodenalis* | A (1) | [167] |
| *Giraffa camelopardalis* | China | 11 | *G. duodenalis* | A (3); E (8) | [166, 181] |
| Family Suidae |  |  |  |  |  |
| *Sus scrofa* | China | 57 | *G. duodenalis* | A (48); E (9) | [182, 183] |
| *Sus scrofa* | Croatia | 1 | *G. duodenalis* | A (1) | [168] |
| *Sus scrofa* | Poland | 6 | *G. duodenalis* | B (6) | [170] |
| *Sus scrofa* | Portugal | 1 | *G. duodenalis* | E (1) | [79] |
| *Sus scrofa* | South Korea | 19 | *G. duodenalis* | A (16); B (2); E (1) | [184] |
| *Sus scrofa* | Spain | 7 | *G. duodenalis* | A (1); E (6) | [163, 185] |
| *Sus scrofa* | USA | 3 | *G. duodenalis* | A (2); E (1) | [186] |
| *Sus scrofa domesticus* | Australia | 57 | *G. duodenalis* | A (19); E (37); F (1) | [187] |
| *Sus scrofa domesticus* | Canada | 63 | *G. duodenalis* | A (58); E (5) | [188] |
| *Sus scrofa domesticus* | China | 344 | *G. duodenalis* | A (17); B (24); C (2); E (301) | [55, 189–195] |
| *Sus scrofa domesticus* | Denmark | 76 | *G. duodenalis* | A (12); D (1); E (63) | [196, 197] |
| *Sus scrofa domesticus* | Nigeria | 53 | *G. duodenalis* | B (14); E (37); B/E (2) | [198] |
| *Sus scrofa domesticus* | Poland | 5 | *G. duodenalis* | B (1); E (4) | [77] |
| *Sus scrofa domesticus* | South Korea | 50 | *G. duodenalis* | A (1); C (12); D (17); E (20) | [199] |
| *Sus scrofa domesticus* | Taiwan | 6 | *G. duodenalis* | A (1); D (1); E (4) | [89] |
| *Sus scrofa domesticus* | UK | 3 | *G. duodenalis* | C (1); F (2) | [98] |
| *Sus scrofa domesticus* | Vietnam | 7 | *G. duodenalis* | A (1); E (4); A/E (2) | [112] |
| Family Tayassuidae |  |  |  |  |  |
| *Dicotyles tajacu* | Croatia | 2 | *G. duodenalis* | A (1); C (1) | [2] |
| Family Camelidae |  |  |  |  |  |
| *Camelus bactrianus* | China | 43 | *G. duodenalis* | A (16); E (26); A/E (1) | [9, 200, 201] |
| *Camelus* sp. | China | 1 | *G. duodenalis* | E (1) | [166] |
| *Lama glama* | China | 2 | *G. duodenalis* | A (1); E (1) | [166] |
| *Lama pacos* | Australia | 6 | *G. duodenalis* | A (5); E (1) | [202] |
| *Lama pacos* | Peru | 134 | *G. duodenalis* | A (104); E (30) | [203, 204] |
| *Lama pacos* | UK | 1 | *G. duodenalis* | A/E (1) | [98] |
| *Lama pacos* | USA | 3 | *G. duodenalis* | A (3) | [205] |
| Family Balaenopteridae |  |  |  |  |  |
| *Balaenoptera acutorostrata* | Spain | 2 | *G. duodenalis* | A (1); A/F (1) | [206] |
| Family Delphinidae |  |  |  |  |  |
| *Delphinus delphis* | USA | 1 | *G. duodenalis* | A (1) | [207] |
| *Delphinus delphis* | Spain | 8 | *G. duodenalis* | A (6); B (2) | [208] |
| *Stenella coeruleoalba* | Spain | 3 | *G. duodenalis* | A (1); D (1); F (1) | [206] |
| *Stenella coeruleoalba* | Italy | 2 | *G. duodenalis* | A (2) | [209] |
| Family Kogiidae |  |  |  |  |  |
| *Kogia breviceps* | Spain | 1 | *G. duodenalis* | C (1) | [206] |
| Family Phocoenidae |  |  |  |  |  |
| *Phocoena phocoena* | Spain | 1 | *G. duodenalis* | F (1) | [206] |
| Order Carnivora |  |  |  |  |  |
| Family Canidae |  |  |  |  |  |
| *Canis aureus* | Bangladesh | 3 | *G. duodenalis* | D (3) | [167] |
| *Canis aureus* | Croatia | 1 | *G. duodenalis* | A/B (1) | [168] |
| *Canis familiaris* | Argentina | 12 | *G. duodenalis* | A (10); A/C (1); A/D (1) | [13] |
| *Canis familiaris* | Belgium | 119 | *G. duodenalis* | A (40); B (4); C (26); D (49) | [210] |
| *Canis familiaris* | Brazil | 63 | *G. duodenalis* | A (17); B (3); C (25); D (11); E (2); C/D (5) | [211–216] |
| *Canis familiaris* | Canada | 111 | *G. duodenalis* | A (44); B (2); C (24); D (34); E (2); A/C (1); A/D (1); B/D (1); C/D (1); D/E (1) | [217–221] |
| *Canis familiaris* | China | 518 | *G. duodenalis* | A (104); B (7); C (158); D (199); E (5); F (2); A/C (5); A/D (12); C/D (26) | [5, 55, 222–236] |
| *Canis familiaris* | Costa Rica | 2 | *G. duodenalis* | D (2) | [237] |
| *Canis familiaris* | Côte d’Ivoire | 6 | *G. duodenalis* | A (2); C (1); D (2); A/B (1) | [136] |
| *Canis familiaris* | Cuba | 11 | *G. duodenalis* | A (7); B (1); A/B (3) | [238] |
| *Canis familiaris* | Ecuador | 1 | *G. duodenalis* | D (1) | [239] |
| *Canis familiaris* | Egypt | 8 | *G. duodenalis* | C (1); D (7) | [240, 241] |
| *Canis familiaris* | Finland | 6 | *G. duodenalis* | C (2); D (3); E (1) | [242] |
| *Canis familiaris* | Germany | 250 | *G. duodenalis* | A (44); C (86); D (89); E (2); F (2); A/C (18); A/D (4); C/D (5) | [243–245] |
| *Canis familiaris* | Greece | 90 | *G. duodenalis* | A (4); C (44); D (27); A/C (2); A/D (1); C/D (12) | [246] |
| *Canis familiaris* | Hungary | 15 | *G. duodenalis* | C (5); D (9); C/D (1) | [247] |
| *Canis familiaris* | India | 19 | *G. duodenalis* | A (1); B (1); C (9); D (7); E (1) | [248] |
| *Canis familiaris* | Iran | 13 | *G. duodenalis* | A (3); C (6); D (4) | [249, 250] |
| *Canis familiaris* | Israel | 29 | *G. duodenalis* | C (5); D (24) | [251] |
| *Canis familiaris* | Italy | 331 | *G. duodenalis* | A (52); B (7); C (152); D (113); A/B (1); A/C (2); C/D (4) | [72, 252–264] |
| *Canis familiaris* | Jamaica | 44 | *G. duodenalis* | A (44) | [265] |
| *Canis familiaris* | Japan | 31 | *G. duodenalis* | C (9); D (22) | [266, 267] |
| *Canis familiaris* | Mexico | 45 | *G. duodenalis* | A (45) | [268–270] |
| *Canis familiaris* | Netherlands | 13 | *G. duodenalis* | A (1); C (7); D (3); C/D (1); unknown (1) | [271] |
| *Canis familiaris* | Poland | 72 | *G. duodenalis* | A (7); B (1); C (28); D (35); E (1) | [77, 272–276] |
| *Canis familiaris* | Portugal | 47 | *G. duodenalis* | B (1); C (18); D (24); C/D (4) | [79, 277, 278] |
| *Canis familiaris* | South Korea | 43 | *G. duodenalis* | A (2); C (17); D (24) | [279, 280] |
| *Canis familiaris* | Spain | 169 | *G. duodenalis* | A (23); B (62); C (25); D (44); E (6); A/B (4); A/D (1); C/D (2); A/B/D (2) | [281–286] |
| *Canis familiaris* | Taiwan | 11 | *G. duodenalis* | C (7); D (4) | [287] |
| *Canis familiaris* | Thailand | 163 | *G. duodenalis* | A (67); B (19); C (19); D (57); C/D (1) | [288–291] |
| *Canis familiaris* | Trinidad | 26 | *G. duodenalis* | C (4); D (21); E (1) | [292] |
| *Canis familiaris* | Turkey | 22 | *G. duodenalis* | A (1); B (1); C (5); D (4); E (9); unknown (2) | [293, 294] |
| *Canis familiaris* | UK | 60 | *G. duodenalis* | A (2); C (20); D (29); F (8); C/D (1) | [295, 330] |
| *Canis familiaris* | USA | 184 | *G. duodenalis* | C (97); D (76); C/D (11) | [107, 296–298] |
| *Canis familiaris* | Vietnam | 2 | *G. duodenalis* | E (2) | [112] |
| *Canis latrans* | Canada | 12 | *G. duodenalis* | A (6); C (1); D (3); A/C (1); A/D (1) | [221, 299] |
| *Canis latrans* | USA | 13 | *G. duodenalis* | A (2); B (3); C (3); D (5) | [107, 300] |
| *Canis lupus* | China | 1 | *G. duodenalis* | D (1) | [5] |
| *Canis lupus* | Croatia | 15 | *G. duodenalis* (14); *G. microti* (1) | A (7); C (2); D (1); A/C (2); A/D (1); C/D (1) | [2, 168] |
| *Canis lupus* | Poland | 2 | *G. duodenalis* | D (2) | [170] |
| *Canis lupus signatus* | Portugal | 6 | *G. duodenalis* | D (4); C/D (2) | [79] |
| *Chrysocyon brachyurus* | Croatia | 1 | *G. duodenalis* | A/B (1) | [2] |
| ***Lycaon pictus*** | China | 3 | *G. duodenalis* | D (1); E (2) | [166] |
| *Nyctereutes procyonoides* | China | 23 | *G. duodenalis* | C (21); C/D (2) | [55, 301] |
| *Nyctereutes procyonoides* | Poland | 2 | *G. duodenalis* | D (2) | [302] |
| *Vulpes vulpes* | Australia | 6 | *G. duodenalis* | D (1); E (1); A/D (1); A/E (3) | [17] |
| *Vulpes vulpes* | Croatia | 1 | *G. duodenalis* | A (1) | [168] |
| *Vulpes vulpes* | Norway | 7 | *G. duodenalis* | A (5); B (2) | [303] |
| *Vulpes vulpes* | Portugal | 1 | *G. duodenalis* | C/D (1) | [79] |
| *Vulpes vulpes* | Romania | 3 | *G. duodenalis* | A (2); B (1) | [304] |
| *Vulpes vulpes* | Sweden | 4 | *G. duodenalis* | B (4) | [305] |
| Family Mustelidae |  |  |  |  |  |
| *Meles meles* | Italy | 10 | *G. duodenalis* | A (8); mixed A/B (2) | [306] |
| Family Otariidae |  |  |  |  |  |
| *Neophoca cinerea* | Australia | 28 | *G. duodenalis* | A (1); B (27) | [307] |
| Family Phocidae |  |  |  |  |  |
| *Halichoerus grypus* *atlantica* | USA | 21 | *G. duodenalis* | A (6); B (5); H (10) | [207] |
| *Phoca hispida* | Canada | 2 | *G. duodenalis* | B (2) | [308] |
| *Phoca vitulina* *richardsi* | USA | 19 | *G. duodenalis* | B (5); D (3); H (11) | [207, 309] |
| *Phoca vitulina vitulina* | USA | 3 | *G. duodenalis* | A (2); A/B (1) | [207] |
| Family Procyonidae |  |  |  |  |  |
| *Nasua nasua* | Croatia | 1 | *G. duodenalis* | A (1) | [2] |
| Family Ursidae |  |  |  |  |  |
| *Helarctos malayanus* | Croatia | 1 | *G. duodenalis* | B (1) | [2] |
| Family Felidae |  |  |  |  |  |
| *Acinonyx jubatus* | Croatia | 2 | *G. duodenalis* (1); *G. microti* (1) | A/D (1); Mixed A/*G. microti* (1) | [2] |
| *Felis catus* | Australia | 7 | *G. duodenalis* | A (1); F (6) | [310] |
| *Felis catus* | Austria | 26 | *G. duodenalis* | A (6); F (20) | [311, 312] |
| *Felis catus* | Brazil | 3 | *G. duodenalis* | A (3) | [211, 213] |
| *Felis catus* | China | 68 | *G. duodenalis* | A (25); B (6); C (3); D (1); F (29); A/C (1); A/F (3) | [5, 55, 226, 228, 230, 232, 313–315] |
| *Felis catus* | Colombia | 3 | *G. duodenalis* | F (3) | [316] |
| *Felis catus* | Costa Rica | 2 | *G. duodenalis* | A (2) | [237] |
| *Felis catus* | Denmark | 10 | *G. duodenalis* | A (8); F (1); A/F (1) | [317] |
| *Felis catus* | Greece | 14 | *G. duodenalis* | A (6); B (1); C (1); F (2); A/F (4) | [246] |
| *Felis catus* | Germany | 19 | *G. duodenalis* | A (2); D (1); F (14); A/F (2) | [244] |
| *Felis catus* | Iran | 10 | *G. duodenalis* | A (5); B (1); F (4) | [249, 318, 319] |
| *Felis catus* | Italy | 80 | *G. duodenalis* | A (56); C (2); D (3); F (17); A/B (2) | [72, 256, 258, 320–322] |
| *Felis catus* | Japan | 68 | *G. duodenalis* | A (11); C (1); F (49); A/F (4); C/D (1); F/C (2) | [266, 323, 324] |
| *Felis catus* | Netherlands | 2 | *G. duodenalis* | A (1); F (1) | [271] |
| *Felis catus* | Poland | 14 | *G. duodenalis* | A (4); B (2); D (2); F (6) | [275, 276, 325] |
| *Felis catus* | Portugal | 2 | *G. duodenalis* | A (2) | [277] |
| *Felis catus* | South Korea | 29 | *G. duodenalis* | A (6); B (10); C (4); D (2); F (6); C/D (1) | [326, 327] |
| *Felis catus* | Spain | 5 | *G. duodenalis* | A (1); F (3); A/F (1) | [281, 283, 284] |
| *Felis catus* | Thailand | 6 | *G. duodenalis* | A (1); C (4); D (1) | [291] |
| *Felis catus* | Turkey | 41 | *G. duodenalis* | A (5); B (31); E (5) | [294, 328, 329] |
| *Felis catus* | UK | 46 | *G. duodenalis* | A (4); B (4); F (38) | [330] |
| *Felis catus* | USA | 22 | *G. duodenalis* | A (6); B (4); E (1); F (11) | [107, 331] |
| *Felis silvestris* | Luxembourg | 1 | *G. duodenalis* | B (1) | [332] |
| *Leptailurus serval* | Croatia | 1 | *G. duodenalis* | A (1) | [2] |
| *Lynx lynx* | Croatia | 1 | *G. duodenalis* | A/D (1) | [2] |
| *Panthera leo bleyenberghi* | China | 2 | *G. duodenalis* | A (1); E (1) | [166] |
| **Panthera pardus** | China | 1 | *G. duodenalis* | D/F (1) | [5] |
| **Panthera pardus fusca** | China | 1 | *G. duodenalis* | A (1) | [166] |
| *Panthera pardus* *orientalis* | Croatia | 1 | *G. duodenalis* (1); *G. microti* (1) | Mixed A/*G. microti* (1) | [2] |
| *Panthera tigris altaica* | China | 1 | *G. duodenalis* | B (1) | [166] |
| *Panthera uncia* | Croatia | 1 | *G. duodenalis* | A/C (1) | [2] |
| **Puma concolor** | USA | 1 | *G. duodenalis* | E (1) | [107] |
| Family Viverridae |  |  |  |  |  |
| *Paguma larvata* | China | 34 | *G. duodenalis* | B (33); B/D (1) | [333] |
| Order Dasyuromorphia |  |  |  |  |  |
| Family Dasyuridae |  |  |  |  |  |
| **Dasyurus** *maculatus* | Australia | 1 | *G. duodenalis* | B (1) | [334] |
| **Planigale maculata** | Australia | 1 | *G. duodenalis* | A (1) | [335] |
| **Sarcophilus harrisii** | Australia | 8 | *G. duodenalis* | B (1); TD genotype 1 (4); TD genotype 2 (3) | [336] |
| Order Didelphimorphia |  |  |  |  |  |
| Family Didelphidae |  |  |  |  |  |
| **Gracilinanus agilis** | Brazil | 1 | *G. duodenalis* | A (1) | [337] |
| *Monodelphis americana* | Brazil | 1 | *G. duodenalis* | A (1) | [337] |
| Order Diprotodontia |  |  |  |  |  |
| Family Macropodidae |  |  |  |  |  |
| *Macropus* **eugenii** | Australia | 3 | *G. duodenalis* | A (1); B (2) | [334] |
| **Macropus fuliginosus** | Australia | 10 | *G. duodenalis* | A (7); B (3) | [334, 338] |
| **Macropus giganteus** | Australia | 19 | *G. duodenalis* | A (9); B (2); C (4); D (4) | [17, 180] |
| **Macropus parma** | Australia | 2 | *G. duodenalis* | A (1); B (1) | [334] |
| *Macropus* **rufogriseus** | China | 1 | *G. duodenalis* | E (1) | [166] |
| *Macropus rufus* | Australia | 6 | *G. duodenalis* | A (5); B (1) | [334] |
| *Macropus* sp. | China | 2 | *G. duodenalis* | E (2) | [166, 181] |
| **Petrogale xanthopus** | Australia | 1 | *G. duodenalis* | A (1) | [334] |
| **Setonix brachyurus** | Australia | 1 | *G. duodenalis* | A (1) | [334] |
| **Wallabia bicolor** | Australia | 2 | *G. duodenalis* | A (2) | [334] |
| Family Phalangeridae |  |  |  |  |  |
| **Trichosurus cunninghami** | Australia | 7 | *G. duodenalis* | A (7) | [334] |
| **Trichosurus vulpecula** | Australia | 12 | *G. duodenalis* (11); *G. peramelis* (1) | A (11) | [334, 339] |
| Family Phascolarctidae |  |  |  |  |  |
| **Phascolarctos cinereus** | Australia | 5 | *G. duodenalis* | A (5) | [334] |
| Family Potoroidae |  |  |  |  |  |
| **Aepyprymnus rufescens** | Australia | 1 | *G. duodenalis* | A (1) | [334] |
| **Potorous tridactylus** | Australia | 1 | *G. duodenalis* | A (1) | [334] |
| Family Vombatidae |  |  |  |  |  |
| **Lasiorhinus** *latifrons* | Australia | 1 | *G. duodenalis* | A (1) | [334] |
| **Vombatus ursinus** | Australia | 1 | *G. duodenalis* | A (1) | [180] |
| Order Eulipotyphla |  |  |  |  |  |
| Family Erinaceidae |  |  |  |  |  |
| **Erinaceus europaeus** | Netherlands | 10 | *G. duodenalis* | A (10) | [340] |
| Order Lagomorpha |  |  |  |  |  |
| Family Leporidae |  |  |  |  |  |
| *Lepus tolai* | China | 51 | *G. duodenalis* | A (1); E (50) | [341] |
| *Oryctolagus cuniculus* | Australia | 1 | *G. duodenalis* | A (1) | [180] |
| *Oryctolagus cuniculus* | Brazil | 2 | *G. duodenalis* | A (2) | [342] |
| *Oryctolagus cuniculus* | China | 212 | *G. duodenalis* | B (187); E (19); A/E (1); B/E (5) | [55, 341, 343–347] |
| *Oryctolagus cuniculus* | Germany | 1 | *G. duodenalis* | B (1) | [348] |
| *Oryctolagus cuniculus* | Nigeria | 60 | *G. duodenalis* | B (60) | [349] |
| *Oryctolagus cuniculus* | Spain | 1 | *G. duodenalis* | B (1) | [350] |
| Order Peramelemorphia |  |  |  |  |  |
| Family Peramelidae |  |  |  |  |  |
| *Isoodon obesulus* | Australia | 87 | *G. duodenalis* (5); *G. peramelis* (82) | A (1); C (3); E (1) | [335, 351, 352] |
| *Isoodon macrourus* | Australia | 1 | *G. peramelis* (1) |  | [339] |
| Order Perissodactyla |  |  |  |  |  |
| Family Equidae |  |  |  |  |  |
| ***Equus asinus*** | China | 219 | *G. duodenalis* | A (53); B (159); A/B (3); E (4) | [353–355] |
| ***Equus asinus*** | Jordan | 11 | *G. duodenalis* | A (5); B (2); E (4) | [356] |
| *Equus ferus caballus* | Australia | 1 | *G. duodenalis* | A (1) | [17] |
| *Equus ferus caballus* | Colombia | 34 | *G. duodenalis* | A (2); B (32) | [357] |
| *Equus ferus caballus* | China | 88 | *G. duodenalis* | A (19); B (58); E (8); G (3) | [9, 166, 354, 358–360] |
| *Equus ferus caballus* | Iran | 13 | *G. duodenalis* | A (4); E (9) | [361] |
| *Equus ferus caballus* | Italy | 57 | *G. duodenalis* | A (16); B (11); E (30) | [362, 363] |
| *Equus ferus caballus* | Jordan | 19 | *G. duodenalis* | A (13); B (2); E (4) | [356] |
| *Equus ferus caballus* | Poland | 1 | *G. duodenalis* | E (1) | [77] |
| *Equus ferus caballus* | Portugal | 1 | *G. duodenalis* | E (1) | [79] |
| *Equus ferus caballus* | Turkey | 25 | *G. duodenalis* | A (25) | [364] |
| *Equus zebra* | China | 4 | *G. duodenalis* | E (4) | [166] |
| Order Pholidota |  |  |  |  |  |
| Family Manidae |  |  |  |  |  |
| *Manis palaeojavanica* | Bangladesh | 1 | *G. duodenalis* | B (1) | [167] |
| Order Pilosa |  |  |  |  |  |
| Family Bradypodidae |  |  |  |  |  |
| *Bradypus tridactylus* | Brazil | 2 | *G. duodenalis* | A (2) | [365] |
| Order Primates |  |  |  |  |  |
| New world monkey species |  |  |  |  |  |
| Family Atelidae |  |  |  |  |  |
| Subfamily Alouattinae |  |  |  |  |  |
| *Alouatta caraya* | Argentina | 47 | *G. duodenalis* | B (47) | [13] |
| *Alouatta caraya* | Brazil | 1 | *G. duodenalis* | A (1) | [366] |
| *Alouatta fusca* | Brazil | 1 | *G. duodenalis* | A (1) | [366] |
| *Alouatta guariba* | Brazil | 16 | *G. duodenalis* | A (16) | [367] |
| **Alouatta pigra** | Mexico | 3 | *G. duodenalis* | A (1); B (2) | [368] |
| Subfamily Atelinae |  |  |  |  |  |
| *Ateles belzebuth* | Brazil | 1 | *G. duodenalis* | A (1) | [366] |
| Family Callitrichidae |  |  |  |  |  |
| *Callithrix* sp. | Spain | 1 | *G. duodenalis* | B (1) | [369] |
| *Callimico* sp. | Spain | 1 | *G. duodenalis* | B (1) | [369] |
| *Cebuella* sp. | Spain | 1 | *G. duodenalis* | B (1) | [369] |
| Family Cebidae |  |  |  |  |  |
| Subfamily Cebinae |  |  |  |  |  |
| *Cebus* sp. | Spain | 1 | *G. duodenalis* | A (1) | [369] |
| Subfamily Saimiriinae |  |  |  |  |  |
| *Saimiri* *sciureus* | China | 1 | *G. duodenalis* | E (1) | [370] |
| *Saimiri* sp. | China | 10 | *G. duodenalis* | E (10) | [371] |
| Old world monkey species |  |  |  |  |  |
| Family Cercopithecidae |  |  |  |  |  |
| Subfamily Cercopithecinae |  |  |  |  |  |
| Tribe Cercopithecini |  |  |  |  |  |
| *Cercopithecus ascanius* | Uganda | 1 | *G. duodenalis* | F (1) | [372] |
| *Cercopithecus* *hamlyni* | Spain | 1 | *G. duodenalis* | A (1) | [373] |
| *Cercopithecus kandti* | China | 17 | *G. duodenalis* | B (17) | [371, 374] |
| *Cercopithecus mona* | China | 1 | *G. duodenalis* | B (1) | [371] |
| *Cercopithecus neglectus* | Spain | 1 | *G. duodenalis* | A (1) | [373] |
| *Cercopithecus roloway* | China | 1 | *G. duodenalis* | B (1) | [371] |
| *Chlorocebus sabaeus* | China | 4 | *G. duodenalis* | A (2); B (2) | [166, 371] |
| *Erythrocebus patas* | China | 5 | *G. duodenalis* | B (5) | [371] |
| Tribe Papionini |  |  |  |  |  |
| *Cercocebus atys* | Spain | 1 | *G. duodenalis* | A (1) | [373] |
| *Cercocebus* *torquatus* | Spain | 1 | *G. duodenalis* | A (1) | [369] |
| *Macaca assamensis* | China | 1 | *G. duodenalis* | B (1) | [375] |
| *Macaca fascicularis* | China | 483 | *G. duodenalis* | A (3); B (480) | [371, 375–378] |
| *Macaca fascicularis* | Thailand | 14 | *G. duodenalis* | A (1); B (12); unknown (1) | [379] |
| *Macaca fuscata* | China | 3 | *G. duodenalis* | B (3) | [375] |
| *Macaca mulatta* | Bangladesh | 3 | *G. duodenalis* | B (3) | [167] |
| *Macaca mulatta* | China | 102 | *G. duodenalis* | A (13); B (86); E (3) | [370, 371,375, 377, 380, 381] |
| *Macaca mulatta* | India | 17 | *G. duodenalis* | B (17) | [113] |
| *Macaca nemestrina* | China | 9 | *G. duodenalis* | B (9) | [371] |
| *Macaca thibetana* | China | 2 | *G. duodenalis* | A (1); E (1) | [166] |
| *Mandrillus leucophaeus* | Spain | 1 | *G. duodenalis* | A (1) | [373] |
| *Mandrillus* *sphinx* | China | 2 | *G. duodenalis* | B (2) | [371] |
| *Papio anubis* | China | 3 | *G. duodenalis* | B (2); E (1) | [166, 375] |
| *Papio cynocephalus* | China | 2 | *G. duodenalis* | B (2) | [371] |
| *Papio hamadryas* | China | 1 | *G. duodenalis* | B (1) | [371] |
| Subfamily Colobinae |  |  |  |  |  |
| African group |  |  |  |  |  |
| *Colobus guereza* | China | 1 | *G. duodenalis* | A (1) | [166] |
| *Colobus guereza* | Croatia | 1 | *G. duodenalis* | B (1) | [2] |
| *Colobus guereza* | Uganda | 1 | *G. duodenalis* | F (1) | [372] |
| *Colobus polykomos* | China | 1 | *G. duodenalis* | B (1) | [371] |
| Langur group |  |  |  |  |  |
| *Semnopithecus johnii* | Bangladesh | 1 | *G. duodenalis* | A/B (1) | [167] |
| *Trachypithecus* *francoisi* | China | 1 | *G. duodenalis* | A (1) | [371] |
| *Trachypithecus leucocephalus* | China | 1 | *G. duodenalis* | B (1) | [375] |
| Odd-nosed group |  |  |  |  |  |
| **Rhinopithecus bieti** | China | 2 | *G. duodenalis* | B (2) | [381] |
| *Rhinopithecus roxellana* | China | 8 | *G. duodenalis* | A (8) | [181] |
| Family Hominidae |  |  |  |  |  |
| *Gorilla beringei* | Rwanda | 6 | *G. duodenalis* | B (6) | [382] |
| *Gorilla beringei* | Uganda | 2 | *G. duodenalis* | A (2) | [95] |
| *Gorilla* *gorilla* | Spain | 1 | *G. duodenalis* | A (1) | [373] |
| *Pan troglodytes* | Australia | 4 | *G. duodenalis* | B (4) | [383] |
| *Pan troglodytes* | Croatia | 1 | *G. duodenalis* | B (1) | [2] |
| *Pan troglodytes* | Spain | 1 | *G. duodenalis* | B (1) | [373] |
| *Pan* sp. | Spain | 2 | *G. duodenalis* | A (2) | [369] |
| *Pongo* *pygmaeus* | China | 5 | *G. duodenalis* | B (5) | [371] |
| Family Hylobatidae |  |  |  |  |  |
| *Hylobates lar* | Croatia | 1 | *G. duodenalis* | B (1) | [2] |
| *Hylobates lar* | Thailand | 1 | *G. duodenalis* | B (1) | [384] |
| *Nomascus* *leucogenys* | China | 16 | *G. duodenalis* | B (16) | [371, 377] |
| Family Lemuridae |  |  |  |  |  |
| *Eulemur fulvus* | Spain | 1 | *G. duodenalis* | B (1) | [373] |
| *Eulemur rubriventer* | Spain | 1 | *G. duodenalis* | A (1) | [373] |
| *Eulemur rufus* | Spain | 1 | *G. duodenalis* | A (1) | [373] |
| *Hapalemur aureus* | Spain | 1 | *G. duodenalis* | A (1) | [373] |
| *Lemur catta* | China | 32 | *G. duodenalis* | A (1); B (31) | [166, 371, 374] |
| *Lemur catta* | Croatia | 3 | *G. duodenalis* | B (3) | [2] |
| *Lemur catta* | Italy | 8 | *G. duodenalis* | B (8) | [385] |
| *Lemur catta* | Spain | 3 | *G. duodenalis* | B (3) | [373] |
| *Lemur* sp. | Spain | 12 | *G. duodenalis* | B (12) | [369] |
| *Varecia rubra* | Spain | 1 | *G. duodenalis* | A (1) | [373] |
| *Varecia variegata* | China | 1 | *G. duodenalis* | E (1) | [166] |
| Family Lorisidae |  |  |  |  |  |
| *Nycticebus coucang* | China | 2 | *G. duodenalis* | B (2) | [371] |
| Order Rodentia |  |  |  |  |  |
| Family Castoridae |  |  |  |  |  |
| *Castor canadensis* | USA | 4 | *G. duodenalis* | B (4) | [386] |
| Family Caviidae |  |  |  |  |  |
| ***Dolichotis patagonum*** | China | 6 | *G. duodenalis* | A (3); B (1); E (2) | [166] |
| ***Dolichotis patagonum*** | Croatia | 1 | *G. duodenalis* | B (1) | [2] |
| Family Chinchillidae |  |  |  |  |  |
| *Chinchilla lanigera* | China | 38 | *G. duodenalis* | A (5); B (33) | [387] |
| *Chinchilla lanigera* | Germany | 23 | *G. duodenalis* | B (19); D (1); A/B (3) | [348] |
| *Chinchilla lanigera* | Italy | 31 | *G. duodenalis* | B (29); C (2) | [388] |
| *Chinchilla lanigera* | Romania | 190 | *G. duodenalis* | B (151); D (33); E (6) | [389] |
| Family Cricetidae |  |  |  |  |  |
| Subfamily Arvicolinae |  |  |  |  |  |
| *Arvicola sapidus* | Portugal | 6 | *G. microti* (6) |  | [390] |
| *Clethrionomys glareolus* | Germany | 180 | *G. duodenalis* (4); *G. microti* (173); *G. muris* (3) | A (2); B (2) | [391] |
| *Clethrionomys glareolus* | Poland | 3 | *G. microti* (1); *G. muris* (2) |  | [392] |
| *Microtus agrestis* | Germany | 51 | *G. microti* (49); *G. muris* (2) |  | [391] |
| *Microtus arvalis* | Germany | 78 | *G. microti* (78) |  | [391] |
| *Microtus arvalis* | Spain | 4 | *G. duodenalis* (4) |  | [393] |
| *Microtus cabrerae* | Portugal | 1 | *G. mcroti* (1) |  | [390] |
| *Microtus* **duodecimcostatus** | Spain | 1 | *G. duodenalis* (1) |  | [393] |
| *Microtus lusitanicus* | Portugal | 7 | *G. mcroti* (7) |  | [390] |
| Subfamily Cricetinae |  |  |  |  |  |
| *Mesocricetus auratus* | China | 11 | *G. cricetidarum* (11) |  | [394] |
| *Nothocricetulus* *migratorius* | Iran | 3 | *G. muris* (3) |  | [395] |
| *Phodopus campbelli* | China | 9 | *G. cricetidarum* (9) |  | [394] |
| *Phodopus sungorus* | China | 45 | *G. duodenalis* (6); *G. muris* (3); *G. cricetidarum* (36) |  | [394] |
| Subfamily Neotominae |  |  |  |  |  |
| *Peromyscus maniculatus* | USA | 10 | *G. microti* (10) |  | [396] |
| Subfamily Sigmodontinae |  |  |  |  |  |
| *Akodon cursor* | Brazil | 1 | *G. duodenalis* | A (1) | [337] |
| *Hylaeamys laticeps* | Brazil | 1 | *G. duodenalis* | A (1) | [337] |
| *Oecomys catherinae* | Brazil | 1 | *G. duodenalis* | A (1) | [337] |
| *Oligoryzomys nigripes* | Brazil | 1 | *G. duodenalis* | A (1) | [337] |
| Family Echimyidae |  |  |  |  |  |
| *Myocastor coypus* | China | 38 | *G. duodenalis* | A (2); B (35); A/B (1) | [397] |
| Family Hystricidae |  |  |  |  |  |
| *Hystrix cristata* | Italy | 15 | *G. duodenalis* | A (2); B (13) | [398] |
| Family Muridae |  |  |  |  |  |
| Subfamily Gerbillinae |  |  |  |  |  |
| *Meriones persicus* | Iran | 15 | *G. muris* (15) |  | [395] |
| Subfamily Murinae |  |  |  |  |  |
| Tribe Apodemini |  |  |  |  |  |
| **Apodemus agrarius** | Germany | 23 | *G. microti* (2); *G. muris* (21) |  | [391] |
| **Apodemus agrarius** | Poland | 2 | *G. muris* (2) |  | [392] |
| *Apodemus flavicollis* | Germany | 13 | *G. microti* (5); *G. muris* (8) |  | [391] |
| *Apodemus flavicollis* | Poland | 5 | *G. microti* (1); *G. muris* (4) |  | [392] |
| **Apodemus sylvaticus** | Germany | 2 | *G. muris* (2) |  | [391] |
| **Apodemus sylvaticus** | Portugal | 2 | *G. microti* (2) |  | [390] |
| Tribe Hydromyini |  |  |  |  |  |
| *Conilurus penicillatus* | Australia | 1 | *G. peramelis* (1) |  | [339] |
| *Pseudomys albocinereus* | Australia | 1 | *G. duodenalis* | E (1) | [335] |
| Tribe Murini |  |  |  |  |  |
| *Mus musculus* | Argentina | 55 | *G. muris* (55) |  | [399] |
| *Mus musculus* | China | 1 | *G. duodenalis* | G (1) | [400] |
| *Mus musculus* | Iran | 2 | *G. duodenalis* (1); *G. muris* (1) | G (1) | [395, 401] |
| *Mus musculus* | Spain | 3 | *G. duodenalis* | G (3) | [402] |
| *Mus spretus* | Portugal | 4 | *G. microti* (4) |  | [390] |
| Tribe Rattini |  |  |  |  |  |
| *Bandicota indica* | Thailand | 16 | *G. duodenalis* | A (14); B (2) | [403] |
| *Rattus fuscipes* | Australia | 1 | *G. duodenalis* | F/C (1) | [335] |
| *Rattus norvegicus* | Argentina | 61 | *G. muris* (61) |  | [399] |
| *Rattus norvegicus* | China | 57 | *G. duodenalis* (53); *G. muris* (4) | G (53) | [55, 394, 400, 404] |
| *Rattus norvegicus* | Iran | 1 | *G. duodenalis* |  | [401] |
| *Rattus norvegicus* | Spain | 35 | *G. duodenalis* (35) | G (1) | [405] |
| *Rattus norvegicus* | Austria | 12 | *G. duodenalis* | A (9); G (3) | [406] |
| *Rattus rattus* | Iran | 2 | *G. duodenalis* | B (1); G (1) | [401] |
| *Rattus rattus* | Malaysia | 1 | *G. duodenalis* | B (1) | [407] |
| *Rattus rattus* | Spain | 28 | *G. duodenalis* | B (1); G (19); unknown (8) | [393, 402] |
| *Rattus tanezumi* | China | 2 | *G. duodenalis* | G (2) | [400] |
| *Rattus tanezumi* | Thailand | 37 | *G. duodenalis* | A (35); C (2) | [403] |
| Family Sciuridae |  |  |  |  |  |
| *Callosciurus prevostii* | Croatia | 1 | *G. duodenalis* | B (1) | [2] |
| *Eutamias asiaticus* | China | 24 | *G. duodenalis* | A (13); G (11) | [408] |
| *Marmota himalayana* | China | 6 | *G. duodenalis* | A (1); B (4); E (1) | [409] |
| *Spermophilus alashanicus* | China | 2 | *G. duodenalis* | B (2) | [409] |
| Family Spalacidae |  |  |  |  |  |
| **Rhizomys sinensis** | China | 52 | *G. duodenalis* | B (52) | [410] |

**REFERENCES**

1. Geurden T, Goossens E, Levecke B, Vercammen F, Vercruysse J, Claerebout E (2009) Occurrence and molecular characterization of *Cryptosporidium* and *Giardia* in captive wild ruminants in Belgium. *Journal of Zoo and Wildlife Medicine* 40: 126-130.
2. Beck R, Sprong H, Bata I Lucinger S, Pozio E, Caccio SM (2011) Prevalence and molecular typing of *Giardia* spp. in captive mammals at the zoo of Zagreb, Croatia. *Veterinary Parasitology* 175: 40-46.
3. Song GY, Qin SY, Zhao GH, Zhu XQ, Zhou DH, Song MX (2016) Molecular characterization of *Giardia duodenalis* from white yaks in China. *Acta* *Parasitologica* 61: 397-400.
4. Jin Y, Fei J, Cai J, Wang X, Li N, Guo Y, Feng Y, Xiao L (2017) Multilocus genotyping of *Giardia duodenalis* in Tibetan sheep and yaks in Qinghai, China. *Parasitology Research* 247: 70-76.
5. Liu H, Shen Y, Liu A, Yin J, Yuan Z, Jiang Y, Pan W, Zhang Y, Zhao W, Cao J (2017) Occurrence and multilocus genotyping of *Giardia duodenalis* in pets and zoo animals in Shanghai, China. *Journal of Infection in Developing Countries* 11: 479-486.
6. Wang G, Wang G, Li XP, Ma LQ, Karanis G, Eleni CV, Karanis P (2017) Detection of *Giardia duodenalis* assemblage E infections at the Tibetan Plateau Area: Yaks are suitable hosts. *Acta Tropica* 169: 157-62.
7. Wang G, Wang G, Li X, Zhang X, Karanis G, Jian Y, Ma, Karanis P (2018) Prevalence and molecular characterization of *Cryptosporidium* spp. and *Giardia duodenalis* in 1-2-month-old d yaks in Qinghai Province, China. *Parasitology Research* 117: 1793-800.
8. Wu Y, Chang Y, Zhang X, Chen Y, Li D, Wang L, Zheng S, Wang R, Zhang S, Jian F, Ning C, Li J, Zhang L (2019) Molecular characterization and distribution of *Cryptosporidium* spp., *Giardia duodenalis*, and *Enterocytozoon bieneusi* from yaks in Tibet, China. *BMC Veterinary Research* 15: 417.
9. Zhang Q, Zgang Z, Ai S, Wang X, Zhang R, Duan Z (2019) *Cryptosporidium* spp., *Enterocytozoon bieneusi*, and *Giardia duodenalis* from animal sources in the Qinghai-Tibetan Plateau Area (QTPA) in China. *Comparative Immunology, Microbiology and Infectious Disease* 67: 101346.
10. Wu Y, Chen Y, Chang Y, Zhang X, Li D, Wang L, Zheng S, Wang R, Zhang S, Li J, Zhang L (2020) Genotyping and identification of *Cryptosporidium* spp., *Giardia duodenalis* and *Enterocytozoon bieneusi* from free-range Tibetan yellow cattle and cattle-yak in Tibet, China. *Acta Tropica* 212: 105671.
11. Song JK, Wang D, Ren M, Yang F, Wang PX, Zou M, Zhao GH, Lin Q (2021) Seasonal prevalence and novel multilocus genotypes of *Giardia duodenalis* in yaks (*Bos grunniens*) in Qinghai province, western China. *Iran Journal of Parasitology* 16: 548-554.
12. Baroudi D, Khelef D, Hakem A, Abdelaziz A, Chen X, Lysen C, Roellig D, Xiao L (2017) Molecular characterization of zoonotic pathogens *Cryptosporidium* spp., *Giardia duodenalis* and *Enterocytozoon bieneusi* in calves in Algeria. *Veterinary Parasitology: Regional Studies and Reports* 8: 66-69.
13. Kuthyar S, Kowalewski MM, Seabolt M, Roellig DM, Gillespie TR (2021) Molecular characterization of *Giardia duodenalis* and evidence for cross-species transmission in Northern Argentina. *Transboundary and Emerging Diseases* 69: 2209-2218.
14. O’Handley RM, Olson ME, Fraser D, Adams P, Thompson RCA (2000) Prevalence and genotypic characterisation of *Giardia* in dairy calves from Western Australia and Western Canada. *Veterinary Parasitology* 90: 193-200.
15. Borchard P, Wright IA, Eldridge DJ (2010) Wombats and domestic livestock as potential vectors of *Cryptosporidium* and *Giardia* in an agricultural riparian area. *Australian Journal of Zoology* 58: 150-153.
16. Ng J, Yang R, McCarthy S, Gordon C, Hijjawi N, Ryan U (2011) Molecular characterization of *Cryptosporidium* and *Giardia* in pre-weaned calves in Western Australia and New South Wales. *Veterinary Parasitology* 176: 145-150.
17. Ng J, Yang R, Whiffin V, Cox P, Ryan U (2011) Identification of zoonotic *Cryptosporidium* and *Giardia* genotypes infecting animals in Sydney’s water catchments. *Experimental Parasitology* 128: 138-144.
18. Asher AJ, Hose G, Power ML (2016) Giardiasis in NSW: Identification of *Giardia duodenalis* assemblages contributing to human and cattle cases, and an epidemiological assessment of sporadic human giardiasis. *Infection, Genetics and Evolution* 44: 157-161.
19. Zahedi A, Odgers T, Ball A, Watkinson A, Robertson I, Ryan UM (2020) Longitudinal analysis of *Giardia duodenalis* assemblages in animals inhabiting drinking water catchments in New South Wales and Queensland - Australia (2013-2015). *Science of the Total Environment* 718: 137433.
20. Lichtmannsperger K, Hinney B, Joachim A, Wittek T (2019) Molecular characterization of *Giardia intestinalis* and *Cryptosporidium* *parvum* from calves with diarrhoea in Austria and evaluation of point-of-care tests. *Comparative Immunology, Microbiology and Infectious Disease* 66: 101333.
21. Ehsan AM, Geurden T, Casaert S, Parvin SM, Islam TM, Ahmed UM, Levecke B, Vercruysse J, Claerebout E (2015) Assessment of zoonotic transmission of *Giardia* and *Cryptosporidium* between cattle and humans in rural villages in Bangladesh. *PLoS ONE* 10: e0118239.
22. Li J, Karim MR, Siddiki SHMF, Chen Y, Qin Z, Rume FI, Zhang L (2023) Potential zoonotic transmission of *Giardia duodenalis* between children and calves in Bangladesh. *Transboundary and Emerging Diseases* Article ID:8224587.
23. Geurden T, Geldhof P, Levecke B, Martens C, Berkvens D, Casaert S, Vercruysse J, Claerebout E (2008) Mixed *Giardia duodenalis* assemblage A and E infections in calves. *International Journal for Parasitology* 38: 259-264.
24. Paz e Silva FM, Lopes RS, Araújo JP Jr (2012) Genetic characterisation of *Giardia duodenalis* in dairy cattle in Brazil. *Folia Parasitologica* 59: 15-20.
25. Fava NMN, Soares RM, Scalia LAM, Kalapothakis E, Pena IF, Vieira CU, Faria ESM, Cunha MJ, Couto TR, Cury MC (2013) Performance of glutamate dehydrogenase and triose phosphate isomerase genes in the analysis of genotypic variability of isolates of *Giardia duodenalis* from livestocks. *BioMed Research International* Article ID 875048: 9 pages.
26. Ferreira-Fagundes TF, Vidal LGP, Alves PAM, McIntosh D, Menezes R. CAA, Fonseca AH, Pereira MJS (2013) Molecular characterization of *Giardia intestinalis* in heifer calves reared in individual shelters within paddocks at the Municipality of Piraí in the State of Rio de Janeiro, Brazil. *Revista Brasileira de Medicina Veterinária* 35: 17-21.
27. Appelbee AJ., Frederick LM., Heitman TL., Olson ME (2003) Prevalence and genotyping of *Giardia duodenalis* from beef calves in Alberta, Canada. *Veterinary Parasitology* 112: 289-294.
28. Uehlinger FD, Barkema HW, Dixon BR, Coklin T, O'Handley RM (2006) [*Giardia duodenalis* and *Cryptosporidium* spp. in a veterinary college bovine teaching herd.](https://pubmed.ncbi.nlm.nih.gov/16905259/)  *Veterinary Parasitology* 142: 231-237.
29. Coklin T, Farber J, Parrington L, Dixon B (2007) Prevalence and molecular characterization of *Giardia duodenalis* and *Cryptosporidium* spp. in dairy cattle in Ontario, Canada. *Veterinary Parasitology* 150: 297-305
30. Uehlinger FD, Greenwood SJ, O'Handley R, McClure JT, Coklin T, Dixon BR, de Boer M, Zwiers H, Barkema HW (2011) Prevalence and genotypes of *Giardia duodenalis* in dairy and beef cattle in farms around Charlottetown, Prince Edward Island, Canada. *Canadian Veterinary Journal* 52: 967-72.
31. Budu-Amoako E, Greenwood SJ, Dixon BR, Barkema HW, McClure JT (2012) *Giardia* and *Cryptosporidium* on dairy farms and the role these farms may play in contaminating water Sources in Prince Edward Island, Canada. *Journal of Veterinary Internal Medicine* 26: 668-673.
32. Budu-Amoako E, Greenwood SJ, Dixon BR, Barkema H.W, McClure JT (2012) Occurrence of *Cryptosporidium* and *Giardia* on beef farms and water sources within the vicinity of the farms on Prince Edward Island, Canada. *Veterinary Parasitology* 184: 1-9.
33. Liu A, Zhang X, Zhang L, Wang R, Li X, Shu J, Zhang X, Shen Y, Zhang W, Ling H (2012) Occurrence of bovine giardiasis and endemic genetic characterization of *Giardia duodenalis* isolates in Heilongjiang Province, in the Northeast of China. *Parasitology Reseaech* 111: 655-661.
34. Huang J, Yue D, Qi M, Wang R, Zhao J, Li J, Shi K, Wang M, Zhang L (2014) Prevalence and molecular characterization of *Cryptosporidium* spp. and *Giardia duodenalis* in dairy cattle in Ningxia, northwestern China. *BMC Veterinary Research* 10: 292.
35. Wang H, Zhao G, Chen G, Jian F, Zhang S, Feng C, et al. (2014) Multilocus genotyping of *Giardia duodenalis* in dairy cattle in Henan, China. *PLoS* *ONE* 9: e100453.
36. Liu G, Su Y, Zhou M, Zhao J, Zhang T, Ahmad W, Lu H, Jiang N, Chen Q, Xiang M, Yin J (2015) Prevalence and molecular characterization of *Giardia duodenalis* isolates from dairy cattle in northeast China. *Experimental Parasitology* 154: 20-24
37. Li F, Wang H, Zhang Z, Li J, Wang C, Zhao J, Hu S, Wang R, Zhang L, Wang M (2016) Prevalence and molecular characterization of *Cryptosporidium* spp. and *Giardia duodenalis* in dairy cattle in Beijing, China. *Veterinary Parasitology* 219: 61-65.
38. Qi M, Wang H, Jing B, Wang R, Jian F, Ning C, Zhang L (2016) Prevalence and multilocus genotyping of *Giardia duodenalis* in dairy calves in Xinjiang, Northwestern China. *Parasites & Vectors* 9:546.
39. Wang C, Zhang Z, Li J, Yu F, Cao J, Zhang L (2016) The investigation of infection and multilocus sequence of *Giardia duodenalis* in dairy cattle from an imported farm. *Acta Veterinaria et Zootechnica Sinica* 47: 165-171 (in Chinese).
40. Zhang XX, Tan QD, Zhao GH, Ma JG, Zheng WB, Ni XT, et al. (2016) Prevalence, risk factors and multilocus genotyping of *Giardia intestinalis* in dairy cattle, Northwest China. *Journal of Eukaryotic Microbiology* 63: 498-504.
41. Fan Y, Wang T, Koehler AV, Hu M, Gasser RB (2017) Molecular investigation of *Cryptosporidium* and *Giardia* in pre- and post-weaned calves in Hubei Province, China. *Parasites & Vectors* 10: 519.
42. Hu S, Liu Z, Yan F, Zhang Z, Zhang G, Zhang L, Jian F, Zhang S, Ning C, Wang R (2017) Zoonotic and host-adapted genotypes of *Cryptosporidium* spp., *Giardia duodenalis* and *Enterocytozoon bieneusi* in dairy cattle in Hebei and Tianjin, China. *Veterinary Parasitology* 248: 68-73.
43. Wang X, Cai M, Jiang W, Wang Y, Jin Y, Li N, Guo Y, Feng Y, Xiao L (2017) High genetic diversity of *Giardia duodenalis* assemblage E in pre-weaned dairy calves in Shanghai, China, revealed by multilocus genotyping. *Parasitology Research* 116: 2101-2110.
44. Cui Z, Wang L, Cao L, Sun M, Liang N, Wang H, Chang Y, Lin X, Yu L, Wang R, Zhang S, Ning C, Zhang L (2018) Genetic characteristics and geographic segregation of *Giardia duodenalis* in dairy cattle from Guangdong Province, southern China. *Infection, Genetics and Evolution* 66: 95-100
45. Jian Y, Zhang X, Li X, Karanis G, Ma L, Karanis P (2018) Prevalence and molecular characterization of *Giardia duodenalis* in cattle and sheep from the Qinghai-Tibetan Plateau Area (QTPA), northwestern China. *Veterinary Parasitology* 250: 40-44.
46. Zhong Z, Dan J, Yan G, Tu R, Tian Y, Cao S, Shen L, Deng J, Yu S, Geng Y, Gu X, Wang Y, Liu H, Peng G (2018) Occurrence and genotyping of *Giardia duodenalis* and *Cryptosporidium* in pre-weaned dairy calves in central Sichuan province, China. *Parasite* 25: 45.
47. Dan J, Zhang X, Ren Z, Wang L, Cao S, Shen L, Deng J, Zuo Z, Yu S, Wang Y, Ma X, Liu H, Zhou Z, Hu Y, Fu H, He C, Geng Y, Gu X, Peng G, Zhong Z (2019) Occurrence and multilocus genotyping of *Giardia duodenalis* from post-weaned dairy calves in Sichuan province, China. *PLoS ONE* 14: e0224627.
48. Feng Y, Gong X, Zhu K, Li N, Yu Z, Guo Y, Weng Y, Kváč M, Feng Y, Xiao L (2019) Prevalence and genotypic identification of *Cryptosporidium* spp., *Giardia duodenalis* and *Enterocytozoon bieneusi* in pre-weaned dairy calves in Guangdong, China. *Parasites & Vectors* 12: 41.
49. Wang R, Li N, Jiang W, Guo Y, Wang X, Jin Y, Feng Y, Xiao L (2019) Infection patterns, clinical significance, and genetic characteristics of *Enterocytozoon bieneusi* and *Giardia duodenalis* in dairy cattle in Jiangsu, China. *Parasitology Research* 118: 3053-3060.
50. Li S, Zou Y, Zhang XL, Wang P, Chen XQ, Zhu XQ (2020) Prevalence and multilocus genotyping of *Giardia lamblia* in cattle in Jiangxi Province, China: novel assemblage E subtypes identified. *Korean Journal of Parasitology* 58: 681-687.
51. Liang XX, Zou Y, Li TS, Chen H, Wang SS, Cao FQ, Yang JF, Sun XL, Zhu XQ, Zou FC (2021) First report of the prevalence and genetic characterization of *Giardia duodenalis* and *Cryptosporidium* spp. in Yunling cattle in Yunnan Province, southwestern China. *Microbial* *Pathogenesis* 158: 105025.
52. Ma N, Wang HX, Tao WF, Xue NY, Bai JY, Zhao Q, Jiang J, Lyu C (2021) Detection of point prevalence and assemblages of *Giardia* spp. in dairy calves and sika deer, Northeast China. *Vector Borne Zoonotic Diseases* 21: 685-691.
53. Fu Y, Dong H, Bian X, Qin Z, Han H, Lang J, Zhang J, Zhao G, Li J, Zhang L (2022) Molecular characterizations of *Giardia duodenalis* based on multilocus genotyping in sheep, goats, and beef cattle in Southwest Inner Mongolia, China. *Parasite* 29: 33.
54. Heng ZJ, Yang JF, Xie XY, Xu CR, Chen JR, Ma J, He JJ, Mao HM (2022) Prevalence and multilocus genotyping of *Giardia duodenalis* in Holstein cattle in Yunnan, China. *Frontiers in Veterinary Science* 9: 949462.
55. Wu Y, Yao L, Chen H, Zhang W, Jiang Y, Yang F, Liu A, Shen Y (2022) *Giardia duodenalis* in patients with diarrhea and various animals in northeastern China: prevalence and multilocus genetic characterization. *Parasites & Vectors* 15: 165.
56. Gao H, Liang G, Su N, Li Q, Wang D, Wang J, Zhao L, Kang X, Guo K (2023) Prevalence and molecular characterization of *Cryptosporidium* spp., *Giardia duodenalis*, and *Enterocytozoon bieneusi* in diarrheic and non-diarrheic calves from Ningxia, Northwestern China. *Animals* 13: 1983.
57. Zhao L, Zhang ZS, Han WX, Yang B, Chai HL, Wang MY, Wang Y, Zhang S, Zhao WH, Ma YM, Zhan YJ, Wang LF, Ding YL, Wang JL, Liu YH (2023) Prevalence and molecular characterization of *Giardia duodenalis* in dairy cattle in central inner Mongolia, Northern China. *Scientific Reports* 13: 13960.
58. Sabry MA, Taher ES, Meabed EMH (2009) Prevalence and genotyping of zoonotic *Giardia* from Fayoum Governorate, Egypt. *Research Journal of Parasitology* 4: 105-114.
59. Amer SER (2013) Genotypic and phylogenetic characterization of *Giardia intestinalis* from human and dairy cattle in Kafr El Sheikh governorate, Egypt. *Journal of the Egyptian Society of Parasitology* 43: 133-146.
60. Helmy YA, Klotz C, Wilking H, Krücken J, Nöckler K, Von Samson-Himmelstjerna G, et al. (2014) Epidemiology of *Giardia duodenalis* infection in ruminant livestock and children in the Ismailia province of Egypt: insights by genetic characterization. *Parasites & Vectors* 7: 321.
61. Naguib D, El-Gohary AH, Mohamed AA, Roellig DM, Arafat N, Xiao L (2018) Age patterns of *Cryptosporidium* species and *Giardia duodenalis* in dairy calves in Egypt. *Parasitology International* 67: 736-741.
62. Wegayehu T, Karim MR, Erko B, Zhang L, Tilahun G (2016) Multilocus genotyping of *Giardia duodenalis* isolates from calves in Oromia Special Zone, Central Ethiopia. *Infection,* *Genetics and Evolution* 43: 281-288.
63. Kifleyohannes T, Nødtvedt A, Debenham JJ, Terefe G, Robertson LJ (2022) *Cryptosporidium* and *Giardia* in livestock in Tigray, Northern Ethiopia and associated risk factors for infection: A cross-sectional study. *Frontiers in Veterinary Science* 8: 825940.
64. Geurden T, Vanderstichel R, Pohle H, Ehsan A, von Samson-Himmelstjerna G, Morgan ER, Camuset P, Capelli G, Vercruysse J, Claerebout E (2012) A multicentre prevalence study in Europe on *Giardia duodenalis* in calves, with molecular identification and risk factor analysis. *Veterinary Parasitology* 190: 383-390.
65. Gillhuber J, Rügamer D, Pfister K, Scheuerle M (2014) Giardiosis and other enteropathogenic infections: a study on diarrhoeic calves in Southern Germany. *BMC Research Notes* 7: 112-120.
66. Khan SM, Debnath C, Pramanik AK, Xiao L, Nozaki T, Ganguly S (2011) Molecular evidence for zoonotic transmission of *Giardia duodenalis* among dairy farm workers in West Bengal, India. *Veterinary Parasitology* 178: 342-345.
67. Malekifard F, Ahmadpour M (2018) Molecular detection and identification of *Giardia duodenalis* in cattle of Urmia, northwest of Iran. *Veterinary Research Forum* 9: 81-85.
68. Al-Difaie RS (2016) Molecular study to detect genotyping of *Giardia lamblia* from human and cattle feces in Al-Qadisiya governorate, Iraq. *Ibn Al-Haitham Journal for Pure & Applied Sciences* 29: 1-13.
69. Madlol NAB, Ameer QJ, Al-Kaabawi NAM (2020) Molecular detection of *Giardia lamblia* isolated from cattle feces. *Indian Journal of Public Health Research & Development* 11: 1778-1783.
70. Alseady HH, Al-Dabbagh SMK, Marhash AD (2023) Prevalence and molecular characterization of *Giardia intestinalis* isolates from children and calves in Babylon province, Iraq. *Veterinary World* 16: 1781-1789.
71. Lalle M, Pozio E, Capelli G, Bruschi F, Crotti D, Caccio SM (2005) Genetic heterogeneity at the beta-giardin locus among human and animal isolates of *Giardia duodenalis* and identification of potentially zoonotic subgroups. *International Journal for Parasitology* 35: 207-213.
72. Berrilli F, Di Cave D, De Liberato C, Franco A, Scaramozzino P, Orecchia P (2004) Genotype characterisation of *Giardia duodenalis* isolates from domestic and farm animals by SSU-rRNA gene sequencing. *Veterinary Parasitology* 122: 193-199.
73. Otero-Negrete JJ, Ibarra-Velarde F, Martínez-Gordillo MN, Ponce-Macotela M (2011) Prevalence of *Giardia intestinalis* and zoonotic genotype predominance in small scale sheep and cattle farms in five states of the Mexican Republic. *Veterinaria México* 42: 219-226.
74. Learmonth JJ, Ionas G, Pita AB, Cowie RS (2003) Identification and genetic characterisation of *Giardia* and *Cryptosporidium* strains in humans and dairy cattle in the Waikato Region of New Zealand. *Water Science and Technology* 47: 21-26.
75. Winkworth CL, Matthaei CD, Townsend CR (2008) Prevalence of *Giardia* and *Cryptosporidium* spp. in calves from a region in New Zealand experiencing intensification of dairying. *New Zealand Veterinary Journal* 56: 15-20.
76. Abeywardena H, Jex AR, Nolan MJ, Haydon SR, Stevens MA, McAnulty RW, Gasser RB (2012) Genetic characterisation of *Cryptosporidium* and *Giardia* from dairy calves: Discovery of species/genotypes consistent with those found in humans. *Infection, Genetics and Evolution* 12: 1984-1993.
77. Stojecki K, Sroka J, Cencek T, Dutkiewicz J (2015) Epidemiological survey in Łęczyńsko-Włodawskie lake district of eastern Poland reveals new evidence of zoonotic potential of *Giardia intestinalis*. *Annals of Agricultural and Environmental Medicine* 22: 594-598.
78. Mendonca C, Almeida A, Castro A, Delgado ML, Soares S, Correia da Costa JM, Canada N (2007) Molecular characterization of *Cryptosporidium* and *Giardia* isolates from cattle from Portugal. *Veterinary Parasitology* 147: 47-50.
79. Figueiredo AN, Koster PC, Dashti A, Torres RT, Fonseca C, Mysterud A, Bailo B, Carvalho J, Ferreira E, Hipolito D, Fernandes J, Lino A, Palmeira JD, Sarmento P, Neves N, Carrapato C, Calero-Bernal R, Carmena D (2023) Molecular detection and distribution of *Giardia duodenalis* and *Cryptosporidium* spp. infections in wild and domestic animals in Portugal. *Transboundary and Emerging Diseases* Article ID:5849842.
80. Onac D, Jarca A, Kalmar Z, Cozma V (2015) *Giardia duodenalis* in calves from an isolated farm from northwestern Romania. *Scientica Parasitologica* 16: 133-137
81. Bartley PM, Roehe BK, Thomson S, Shaw HJ, Peto F, Innes EA, Katzer F (2019) Detection of potentially human infectious assemblages of *Giardia duodenalis* in fecal samples from beef and dairy cattle in Scotland. *Parasitology* 146: 1123-1130.
82. Lee SH, VanBik D, Kim HY, Cho A, Kim JW, Byun JW, Oem JK, Oh SI, D. Kwak D (2016) Prevalence and molecular characterisation of *Giardia duodenalis* in calves with diarrhoea. *Veterinary Record* 178: 633-633*.*
83. Lee YJ, Han DG, Ryu JH, Chae JB, Chae JS, Yu DH, Park J, Park BK, Kim HC, Choi KS (2018) Identification of zoonotic *Giardia duodenalis* in Korean native calves with normal feces. *Parasitology Research* 117: 1969-1973.
84. Lee YJ, Ryu JH, Shin SU, Choi KS (2019) Prevalence and molecular characterization of *Cryptosporidium* and *Giardia* in pre-weaned native calves in the Republic of Korea. *Parasitology Research*; 118: 3509-3517.
85. Oh SI, Jung SH, Lee HK, Choe C, Hur TY, So KM (2021) Multilocus genotyping of *Giardia duodenalis* occurring in Korean native calves. *Veterinary* *Sciences* 8: 118.
86. Park YJ, Cho HC, Jang DH, Park J, Choi KS (2023) Multilocus genotyping of *Giardia duodenalis* in pre-weaned calves with diarrhea in the Republic of Korea. *PLoS One* 18: e0279533.
87. Abeywardena H, Jex AR, Koehler AV, Rajapakse RJ, Udayawarna K, Haydon SR, et al. (2014) First molecular characterization of *Cryptosporidium* and *Giardia* from bovines (*Bos taurus* and *Bubalus bubalis*) in Sri Lanka: unexpected absence of *C. parvum* from pre-weaned calves. *Parasites & Vectors* 7:75.
88. Hsu BM, Wun HY, Hsu PC (2007) Prevalence and genotyping of *Giardia* in husbandry systems in Taiwan. *Parasitology Research* 101: 275-280.
89. Lam HYP, Chen TTW, Tseng YC, Chang KC, Yang TH, Peng S (2021) Detection and genotyping of *Giardia duodenalis* from cattle and pigs in Hualien country, Eastern Taiwan. *Journal of Microbiology, Immunology and Infection* 54: 718-727.
90. Inpankaew T, Jiyipong T, Thadtapong N, Kengradomkij C, Pinyopanuwat N, Chimnoi W, Jittapalapong S (2015) Prevalence and genotype of *Giardia duodenalis* in dairy cattle from northern and northeastern part of Thailand. *Acta Parasitology* 60: 459-461.
91. Gultekin M, Ural K, Aysul N, Ayan A, Balikci C, Toplu S, Akyildiz G (2017) Prevalence and molecular characterization of *Giardia duodenalis* in calves in Turkey. *Acta Scientiae Veterinariae* 45: 1450.
92. Ayan A, Ural DA, Erdogan H, Kilinc OO, Gultekin M, Ural K (2019) Prevalence and molecular characterization of *Giardia duodenalis* in livestock in Van, Turkey. *International Journal of Ecosystems and Ecology Science* 9: 289-296.
93. Onder Z, Simsek E, Duzlu O, Yetismis G, Ciloglu A, Okur M, Kokcu ND, Inci A, Yildirim A (2020) Molecular prevalence and genotyping of *Giardia duodenalis* in cattle in Central Anatolia Region of Turkey. *Parasitology Research* 119: 2927-2934.
94. Celik BA, Çelik OY, Ayan A, Akyildiz G, Kilinç OO, Ayan OO, Ercan K (2023) Molecular prevalence of *Giardia duodenalis* and subtype distribution (assemblage E and B) in calves in Siirt, Turkey. *Egyptian Journal of Veterinary Sciences* 54: 457-463.
95. Graczyk TK, Bosco-Nizeyi J, Ssebide B, Thompson RC, Read C, Cranfield MR (2002) Anthropozoonotic *Giardia* *duodenalis* genotype (assemblage) a infections in habitats of free-ranging human-habituated gorillas, Uganda. *Journal of Parasitology* 88: 905-909.
96. Nolan MJ, Unger M, Yeap YT, Rogers E, Millet I, Harman K, Fox M, Kalema-Zikusoka G, Blake DP (2017) Molecular characterisation of protest parasites in human habituated mountain gorillas (*Gorilla beringei beringei*), humans and livestock, from Bwindi Impenetrable National Park, Uganda. *Parasites & Vectors* 10: 340.
97. Cibot M, McLennan MR, Kváč M, Sak B, Asiimwe C, Petrželková K (2021) Sparse evidence for *Giardia intestinalis*, *Cryptosporidium* spp. and *Microsporidia* infections in humans, domesticated animals and wild nonhuman primates sharing a farm-forest mosaic landscape in Western Uganda. *Pathogens* 10: 933.
98. Minetti C, Taweenan W, R. Hogg R, Featherstone C, Randle N, Latham SM, Wastling JM (2014) Occurrence and diversity of *Giardia duodenalis* assemblages in livestock in the UK. *Transboundary and Emerging Diseases* 61: e60-e67.
99. Trout JM, Santin M, Ellis Greiner E, Fayer R (2004) Prevalence of *Giardia duodenalis* genotypes in pre-weaned dairy calves. *Veterinary Parasitology* 124: 179-186.
100. Trout JM, Santin M, Ellis Greiner E, Fayer R (2005) Prevalence and genotypes of *Giardia duodenalis* in post-weaned dairy calves. *Veterinary Parasitology* 130: 177-183.
101. Trout JM, Santin M, Ellis Greiner E, Fayer R (2006) Prevalence and genotypes of *Giardia duodenalis* in 1-2 year old dairy cattle. *Veterinary Parasitology* 140: 217-22.
102. Trout JM, Santin M., Fayer R (2007) Prevalence of *Giardia duodenalis* genotypes in adult dairy cows. *Veterinary Parasitology* 147: 205-209.
103. Barigye R, Dyer NW, Newell TK, Khaitsa ML, Trout JM, Santin M, Fayer R (2008) Molecular and immunohistochemical detection of assemblage E, *Giardia duodenalis* in scouring North Dakota calves. *Veterinary Parasitology* 157: 196-202.
104. Santin M, Trout JM, Fayer R (2009) A longitudinal study of *Giardia duodenalis* genotypes in dairy cows from birth to 2 years of age. *Veterinary Parasitology* 162: 40-45.
105. Fayer R, Santin M, Macarisin D (2012) Detection of concurrent infection of dairy cattle with *Blastocystis*, *Cryptosporidium*, *Giardia*, and *Enterocytozoon* by molecular and microscopic methods. *Parasitology Research* 111: 1349-1355.
106. Mark-Carew MP, Wade SE, Chang YF, Schaaf S, Mohammed HO (2012) Prevalence of *Giardia duodenalis* assemblages among dairy herds in the New York City Watershed. *Veterinary Parasitology* 185: 15-157.
107. Oates SC, Miller MA, Hardin D, Conrad PA, Melli A, Jessup DA, Dominik C, Roug A, Tinker MT, Miller WA (2012) Prevalence, environmental loading, and molecular Characterization of *Cryptosporidium* and *Giardia* Isolates from domestic and wild animals along the Central California Coast. *Applied Environmental Microbiology* 78: 8762-8772.
108. Santin M, Dargatz D, Fayer R (2012) Prevalence of *Giardia duodenalis* assemblages in weaned cattle on cow-calf operations in the United States. *Veterinary Parasitology* 183: 231-236.
109. Li X, Flores KA, Barry S, Becchetti TA, Doran M, Finzel JA, Larsen R, Lile D, McDougald N, Nguyen T, Xiao C, Atwill ER (2019) Statewide cross-sectional survey of *Cryptosporidium* and *Giardia* in California cow-calf herds. *Rangeland Ecology & Management* 72: 461-466.
110. Hublin JSY, Maloney JG, George NS, Molokin A, Lombard JE, Urie NJ, Shivley CB, Santin M (2022) Enhanced detection of *Giardia duodenalis* mixed assemblage infections in pre-weaned dairy calves using next generation sequencing. *Veterinary Parasitology* 304: 109702.
111. Geurden T, Somers R, Thanh NT, Vien LV, Nga VT, Giang HH, Dorny P, Giao HK, Vercruysse J (2008) Parasitic infections in dairy cattle around Hanoi, northern Vietnam. *Veterinary Parasitology* 153: 384-8.
112. Iwashita H, Sugamoto T, Takemura T, Tokizawa A, Vu TD, Nguyen TH, Pham TD, Tran NL, Doan HT, Pham AHQ, Yamashiro T (2020) Molecular epidemiology of *Giardia* spp. in northern Vietnam: potential transmission between animals and humans. *Parasite Epidemiology and Control* 12: e00193.
113. Debenham JJ, Tysnes K, Khunger S, Robertson LJ (2017) Occurrence of *Giardia*, *Cryptosporidium*, and *Entamoeba* in wild rhesus macaques (*Macaca mulatta*) living in urban and semi-rural North-West India. International Journal for Parasitology: *Parasites and Wildlife* 6: 29-34.
114. Di Cristanziano V, Santoro M, Parisi F, Albonico M, Shaali MA, Di Cave D, Berrilli F (2014) Genetic characterization of *Giardia duodenalis* by sequence analysis in humans and animals in Pemba Island, Tanzania. *Parasitology International* 63: 438-441.
115. Abeywardena H, Jex AR, von Samson-Himmelstjerna G, Haydon SR, Stevens MA, Gasser RB (2013) First molecular characterisation of *Cryptosporidium* and *Giardia* from *Bubalus bubalis* (water buffalo) in Victoria, Australia. *Infection, Genetics and Evolution* 20: 96-102.
116. Russell S, Power M, Ens E (2020) *Cryptosporidium* and *Giardia* in feral water buffalo (*Bubalus bubalis*) in the South East Arnhem Land Indigenous Protected Area, Australia. *Parasitology Research* 119: 2149-2157.
117. de Aquino MCC, Harvey TV, Inácio SV, Nagata WB, Ferrari ED, Oliveira BCM, Albuquerque GR, Widmer G, Meireles MV, Bresciani KDS (2019) First description of *Giardia duodenalis* in buffalo calves (*Bubalus* *bubalis*) in southwest region of São Paulo State, Brazil. *Food and Waterborne Parasitology* 16: e00062.
118. Utaaker KS, Chaudhary S, Bajwa RS, Robertson LJ (2018) Prevalence and zoonotic potential of intestinal protozoans in bovines in Northern India. *Veterinary Parasitology: Regional Studies and Reports* 13: 92-97.
119. Caccio SM, Rinaldi L, Cringoli G, Condoleo R, Pozio E (2007) Molecular identification of *Cryptosporidium parvum* and *Giardia duodenalis* in the Italian water buffalo (*Bubalus* *bubalis*). *Veterinary Parasitology* 150: 146-149.
120. Barburas DA, Cozma V, Ionică AM, Abbas I, Bărburaș R, Mircean V, D’Amico G, Dubey JP, Györke A (2022) Intestinal parasites of buffalo calves from Romania: molecular characterisation of *Cryptosporidium* spp. and *Giardia duodenalis*, and the first report of *Eimeria bareillyi. Folia* *Parasitologica* 69: 015.
121. Khurajog B, Masakul A, Inpankaew T, Kamyingkird K, Wongpanit K, Jittapalapong S (2014) Prevalence of *Giardia duodenalis* and factors associated with its infection in water buffaloes in Northeast Thailand. *Journal of Tropical Medicine and Parasitology* 37: 35-41.
122. Kilinc OO, Ayan A, Çelik BA, Çelik OY, Yüksek N, Akyıldız G, Oguz FE (2023) The investigation of giardiasis (foodborne and waterborne diseases) in buffaloes in Van region, Türkiye: first molecular report of *Giardia duodenalis* assemblage B from buffaloes. *Pathogens* 12: 106.
123. Zhao GH, Du SZ, Wang HB, Hu XF, Deng MJ, Yu SK, Zhang LX, Zhu XQ (2015) First report of zoonotic *Cryptosporidium* spp., *Giardia intestinalis* and *Enterocytozoon bieneusi* in golden takins (*Budorcas taxicolor bedfordi*). *Infection, Genetics and Evolution* 34: 394-401.
124. Sak B, Petrzelkova KJ, Kvetonova D, Mynarova A, Kathryn A, Shutt KA, Pomajbikova K, Kalousova B, Modry D, Benavides J, Todd A, Kvac M (2013) Long-term monitoring of *Microsporidia*, *Cryptosporidium* and *Giardia* infections in western lowland gorillas (*Gorilla gorilla gorilla*) at different stages of habituation in Dzanga Sangha protected areas, Central African Republic. *PLoS ONE* 8: e71840.
125. Hossain MA, Mina SA, Marzan LW, Emon MAIK, Das R, Siddiki AZ (2015) Molecular characterization of *Giardia intestinalis* assemblage E from goat kids in Bangladesh. *Asian Pacific Journal of Tropical Disease* 5: 374-379.
126. Geurden T, Thomas P, Casaert S, Vercruysse J, Claerebout E (2008) Prevalence and molecular characterization of *Cryptosporidium* and *Giardia* in lambs and goat kids in Belgium. *Veterinary Parasitology* 155: 142-145.
127. Sudré AP, Leles D, Lima MF, Bomfim TCB (2014) First molecular characterisation of *Giardia duodenalis* infection in dairy goats in Brazil. *Veterinarni Medicina* 59: 283-292.
128. Zhang W, Zhang X, Wang R, Liu A, Shen Y, Ling H, Cao J, Yang F, Zhang X, Zhang L (2012) Genetic characterizations of *Giardia duodenalis* in sheep and goats in Heilongjiang province, China and possibility of zoonotic transmission. *PLoS Neglected Tropical Diseases* 6: e1826.
129. Gu YF, Wang LK, Li Y, Li L, Chu XH, Xin DW, Ma CX, Xu WH, Wu SB, Wang HY, Li WC (2014) Prevalence and molecular characterization of *Giardia lamblia* isolates from goats in Anhui province. *Zhongguo Ji Sheng Chong Xue Yu Ji Sheng Chong Bing Za Zhi* 32: 401-403 (in Chinese).
130. Xie SC, Zou Y, Chen D, Jiang MM, Yuan XD, Li Z, Zou FC, Yang JF, Sheng JL, Zhu XQ (2018) Occurrence and multilocus genotyping of *Giardia duodenalis* in Yunnan black goats in China. *BioMed Research International* Article ID: 4601737.
131. Zhong Z, Tu R, Ou H, Yan G, Dan J, Xiao Q, Wang Y, Cao S, Shen L, Deng J, Zuo Z, Ma X, Zhou Z, Liu H, Yu S, Ren Z, Hu Y (2018) Occurrence and genetic characterization of *Giardia duodenalis* and *Cryptosporidium* spp. from adult goats in Sichuan Province, China. *PLoS ONE* 13: e0199325.
132. Chen D, Zou Y, Li Z, Wang SS, Xie SC, Shi LQ, Zou FC, Yang JF, Zhao GH, Zhu XQ (2019) Occurrence and multilocus genotyping of *Giardia duodenalis* in black‑boned sheep and goats in southwestern China. *Parasites & Vectors* 12: 102.
133. Wang P, Zheng L, Liu L, Yu F, Jian Y, Wang R, Zhang S, Zhang L, Ning C, Jian F (2022) Genotyping of *Cryptosporidium* spp., *Giardia duodenalis* and *Enterocytozoon bieneusi* from sheep and goats in China. *BMC Veterinary Research* 18: 361.
134. Yang X, Wang J, Huang S, Song J, Fan Y, Zhao G (2023) Molecular characterization of *Cryptosporidium* spp., *Giardia duodenalis*, *Enterocytozoon bieneusi* and *Escherichia coli* in dairy goat kids with diarrhea in partial regions of Shaanxi province, China. *Animals* 13: 2922.
135. Yu X, Wang H, Li Y, Mu X, Yuan K, Wu A, Guo J, Hong Y, Zhang H (2023) Occurrence and genotypic identification of Blastocystis spp., Enterocytozoon bieneusi, and Giardia duodenalis in Leizhou black goats in Zhanjiang city, Guangdong province, China. *Animals* 13: 2777.
136. Berrilli F, D’Alfonso R, Giangaspero A, Marangi M, Brandonisio O, Kabore Y, Gle C, Cianfanelli C, Lauro R, Di Cave D (2012) *Giardia duodenalis* genotypes and *Cryptosporidium* species in humans and domestic animals in Cote d’Ivoire: occurrence and evidence for environmental contamination. *Transactions of the Royal Society of Tropical Medicine and Hygiene* 106: 191-195.
137. Squire SA, Yang R, Robertson I, Ayi I, Ryan U (2017) Molecular characterization of *Cryptosporidium* and *Giardia* in farmers and their ruminant livestock from the Coastal Savannah zone of Ghana. *Infection Genetics and Evolution* 55: 236-243.
138. Utaaker KS, Myhr N, Bajwa RS, Joshi H, Kumar A, Robertson LJ (2017) Goats in the city: prevalence of *Giardia duodenalis* and *Cryptosporidium* spp. in extensively reared goats in northern India. *Acta Veterinaria Scandinavica* 59: 86.
139. Jafari H, Jalali MHR, Seyfi Abad Shapouri M, Haji Hajikolaii MR (2014) Determination of *Giardia duodenalis* genotypes in sheep and goat from Iran. *Journal of Parasitic Diseases* 38: 81-84.
140. Kiani-Salmi N, Fattahi-Bafghi A, Astani A, Sazmand A, Zahedi A, Firoozi Z, Ebrahimi B, Dehghani-Tafti A, Ryan U, Akrami-Mohajeri F (2019) Molecular typing of *Giardia* *duodenalis* in cattle, sheep and goats in an arid area of central Iran. *Infection, Genetics and Evolution* 75: 104021.
141. Lim YAL, Mahdy MAK, Tan TK, Goh XT, Jex AR, Nolan MJ, Sharma RSK, Gasser RB (2013) First molecular characterization of *Giardia duodenalis* from goats in Malaysia. *Molecular and Cellular Probes* 27: 28-31.
142. Akinkuotu OA, Takeet MI, Otesile EB, Olufemi F, Greenwood SJ, McClure JT (2019) Multilocus genotyping and phylogenetic analyses of *Giardia intestinalis* isolates from indigenous goats in Ogun State, Nigeria. *Acta Tropica* 195: 15-22.
143. Castro-Hermida JA, Almeida A, González-Warleta M, Correia da Costa JM, Rumbo-Lorenzo C, Mezo M (2007) Occurrence of *Cryptosporidium parvum* and *Giardia duodenalis* in healthy adult domestic ruminants. *Parasitology Research* 101: 1443-1448.
144. Ruiz A, Foronda P, Gonzalez JF, Guedes A, Abreu-Acosta N, Molina JM, Valladares B (2008) Occurrence and genotype characterization of *Giardia duodenalis* in goat kids from the Canary Islands, Spain. *Veterinary Parasitology* 154: 137-141.
145. Kutz S, Thompson RCA, Polley L, Kandola K, Nagy J, Wielinga CM, Elkin BT (2008) *Giardia* assemblage A: human genotype in muskoxen in the Canadian Arctic. *Parasites & Vectors* 1: 32.
146. Berg RPKD, Stensvold CR, Jokelainen P, Grønlund AK, Nielsen HV, Kutz S, Kapel CMO (2021) Zoonotic pathogens in wild muskoxen (*Ovibos* *moschatus*) and domestic sheep (*Ovis aries*) from Greenland. Veterinary Medicine and Science 7: 1-13.
147. Ryan UM, Bath C, Robertson I, Read C, Elliot A, Mcinnes L, Traub R, Besier B (2005) Sheep may not be an important zoonotic reservoir for *Cryptosporidium* and *Giardia* parasites. *Applied and Environmental Microbiology* 71: 4992-4997.
148. Yang R, Jacobson C, Cameron Gordon C, Ryan U (2009) Prevalence and molecular characterisation of *Cryptosporidium* and *Giardia* species in pre-weaned sheep in Australia. *Veterinary Parasitology* 161: 19-24.
149. Nolan MJ, Jex AR, Pangasa A, Young ND, Campbell AJ, Stevens M, Gasser RB (2010) Analysis of nucleotide variation within the triose-phosphate isomerase gene of *Giardia* *duodenalis* from sheep and its zoonotic implications. *Electrophoresis* 31: 287-298.
150. Paz e Silva FM, Lopes RS, Bresciani KDS, Amarante AFT, Araujo Jr JP (2014) High occurrence of *Cryptosporidium ubiquitum* and *Giardia duodenalis* genotype E in sheep from Brazil. *Acta Parasitologica* 59: 193-196.
151. Ye J, Xiao L, Wang Y, Guo Y, Roellig DM, Feng Y (2015) Dominance of *Giardia duodenalis* assemblage A and *Enterocytozoon bieneusi* genotype BEB6 in sheep in Inner Mongolia, China. *Veterinary Parasitology* 210: 235-239.
152. Wang H., Qi M., Zhang K., Li J., Huang J., Ning C., Zhang L (2016) Prevalence and genotyping of *Giardia duodenalis* isolated from sheep in Henan Province, central China. Infections, Genetics and Evolution 39: 330-335.
153. Wu Y, Chang Y, Chen Y, Zhang X, Li D, Zheng S, Wang L, Li J, Ning C, Zhang L (2018) Occurrence and molecular characterization of *Cryptosporidium* spp., *Giardia duodenalis*, and *Enterocytozoon bieneusi* from Tibetan sheep in Gansu, China. *Infections, Genetics and Evolution* 64: 46-51.
154. Qi M, Zhang Z, Zhao A, Jing B, Guan G, Luo J, Zhang L (2019) Distribution and molecular characterization of *Cryptosporidium* spp., *Giardia* *duodenalis*, and *Enterocytozoon bieneusi* amongst grazing adult sheep in Xinjiang, China. *Parasitology International* 71: 80-86.
155. Chang Y, Yange Wang Y, Wu Y, Niu Z, Li J, Zhang S, Wang R, Jian F, Ning C, Zhang L (2019) Molecular characterization of *Giardia duodenalis* and *Enterocytozoon bieneusi* isolated from tibetan sheep and tibetan goats under natural grazing conditions in Tibet. *Journal of Eukaryotic* *Microbiology* 67: 100-106.
156. Cao L, Han K, Wang L, Hasi S, Yu F, Cui Z, Hai Y, Zhai X, Zhang L (2020) Genetic characteristics of *Giardia duodenalis* from sheep in Inner Mongolia, China. *Parasite* 27: 60.
157. Peng JJ, Zou Y, Li ZX, Liang QL, Song HY, Li TS, Ma YY, Zhu XQ, Zhou DH (2020) Prevalence and multilocus genotyping of *Giardia duodenalis* in Tan sheep (*Ovis aries*) in northwestern China. *Parasitology International* 77: 102126.
158. Yang F, Ma L, Gou JM, Yao HZ, Ren M, Yang BK, Lin Q (2022) Seasonal distribution of *Cryptosporidium* spp., *Giardia duodenalis* and *Enterocytozoon bieneusi* in Tibetan sheep in Qinghai, China. *Parasites & Vectors* 15: 394.
159. Zhao Q, Lu C, Pei Z, Gong P, Li J, Jian F, Jing B, Qi M, Ning C (2023) *Giardia duodenalis* in Hu sheep: occurrence and environmental contamination on large-scale housing farms. *Parasite* 30: 2.
160. Wegayehu T, Karim MR, Li J, Adamu H, Erko B, Zhang L, Tilahun G (2017) Prevalence and genetic characterization of *Cryptosporidium* species and *Giardia duodenalis* in lambs in Oromia Special Zone, Central Ethiopia. *BMC Veterinary Research* 13: 22.
161. Gómez-Muñoz MT, Cámara-Badenes C, Martínez-Herrero MC, Dea-Ayuela MA, Pérez-Gracia MT, Fernández-Barredo S, Santín M, Fayer R (2012) Multilocus genotyping of *Giardia duodenalis* in lambs from Spain reveals a high heterogeneity. *Research in Veterinary Science* 93: 836-842.
162. Celik OY, Celik BA, Ayan A, Kilinc OO, Ercan K, Selcuk MA, Ayan OO (2023) A microscopic and molecular survey of *Giardia duodenalis* in lambs in Siirt, Turkiye. *Dicle Universitesi Veteriner Fakultesi Dergisi* 16: 71-74.
163. Dashti A, Koster PC, Bailo B, Sanchez de las Matas A, Habela MA, Rivero-Juarez A, Vicente J, Serrano E, Arnal MC, de Luco DF, Morrondo P, Armenteros JA, Balseiro A, Cardona GA, Martinez-Carrasco C, Ortiz JA, Carpio AJ, Calero-Bernal R, Gonzalez-Barrio D, David Carmena D (2023) Occurrence and limited zoonotic potential of *Cryptosporidium* spp., *Giardia duodenalis*, and *Balantioides coli* infections in free-ranging and farmed wild ungulates in Spain. *Research in Veterinary Science* 159: 189-197.
164. De Liberato C, Berrilli F, Marangi M, Santoro M, Trogu T, Putignani L, Lanfranchi P, Ferretti F, D’Amelio S, Giangaspero A (2015) *Giardia duodenalis* in Alpine (*Rupicapra rupicapra rupicapra*) and Apennine (*Rupicapra pyrenaica ornata*) chamois. *Parasites & Vectors* 8: 650.
165. Guadano Procesi IG, Di Filippo MM, De Liberato C, Lombardo A, Brocherel G, Perrucci S, Di Cave D, Berrilli F (2022) *Giardia duodenalis* in wildlife: exploring genotype diversity in Italy and across Europe. *Pathogens* 11: 105.
166. Zou Y, Li XD, Meng YM, Wang XL, Wang HN, Zhu XQ (2022) Prevalence and multilocus genotyping of *Giardia duodenalis* in zoo animals in three cities in China. *Parasitology Research* 121: 2359-2366.
167. Karim MR, Li J, Rume FI, Sumon SMR, Selim ASM, Hoda N, Zhang L (2021) Occurrence and molecular characterization of *Cryptosporidium* spp. and *Giardia duodenalis* among captive mammals in the Bangladesh National Zoo. *Parasitology International* 84: 102414.
168. Beck R, Sprong H, Lucinger S, Pozio E, Caccio SM (2011) A large survey of croatian wild mammals for *Giardia duodenalis* reveals a low prevalence and limited zoonotic potential. *Vector-Borne and Zoonotic Diseases* 11: 1049-1055.
169. Solarczyk P, Majewska AC, Moskwa B, Cabaj W, Dabert M, Nowosad P (2012) Multilocus genotyping of *Giardia duodenalis* isolates from red deer (*Cervus elaphus*) and roe deer (*Capreolus capreolus*) from Poland. *Folia Parasitologica* 59: 237-240.
170. Stojecki K, Sroka J, Caccio SM, Cencek T, Dutkiewicz J, Kusyk P (2015) Prevalence and molecular typing of *Giardia duodenalis* in wildlife from eastern Poland. *Folia Parasitologica* 62: 042.
171. Garcia-Presedo I, Pedraza-Díaz S, González-Warleta M, Mezo M, Gómez-Bautista M, Ortega-Mora LM, Castro-Hermida JA (2013) The first report of *Cryptosporidium bovis*, *C. ryanae* and *Giardia duodenalis* sub-assemblage A-II in roe deer (*Capreolus capreolus*) in Spain. *Veterinary* *Parasitology* 197: 658-664.
172. Ren G, Li J, Xiong J, Lai X, Wang Y, Lei S, Lu X, He T, Zhou Y, Zhang Y, Lv G (2023) Molecular detection and public health risk assessment of *Cryptosporidium* spp., *Giardia duodenalis*, *Enterocytozoon bieneusi*, and *Blastocystis* sp. of animals in a tropical wildlife park of Hainan Island, China. *One Health Bulletin* 3: 1-10.
173. Huang J, Zhang Z, Zhang Y, Yang Y, Zhao J, Wang R, Jian F, Ning C, Zhang W, Zhang L (2018) Prevalence and molecular characterization of *Cryptosporidium* spp. and *Giardia duodenalis* in deer in Henan and Jilin, China. *Parasites & Vectors* 11: 239.
174. Yamazaki A, Izumiyama S, Yagita K, Kishida N, Kubosaki A, Hara-Kudo Y, Kamata Y, Terajima J (2018) The Molecular Detection of *Cryptosporidium* and *Giardia* in Sika Deer (*Cervus Nippon Centralis*) in Japan. *Food Safety* 6: 88-95.
175. Lalle M, Frangipane di Regalbono A, Poppi L, Nobili G, Tonanzi D, Pozio E, Cacciò SM (2007) A novel *Giardia duodenalis* assemblage A subtype in fallow deer. *The Journal of Parasitology* 93: 426-428.
176. Song Y, Li W, Liu H, Zhong Z, Luo Y, Wei Y, Fu W, Ren Z, Zhou Z, Deng L, Cheng J, Peng G (2018) First report of *Giardia duodenalis* and *Enterocytozoon bieneusi* in forest musk deer (*Moschus berezovskii*) in China. *Parasites & Vectors* 11: 204.
177. Cui Z, Wang Q, Huang X, Bai J, Zhu B, Wang B, Guo X, Qi M, Li J (2022) Multilocus genotyping of *Giardia duodenalis* in Alpine musk deer (*Moschus* *chrysogaster*) in China. *Frontiers in Cellular and Infection Microbiology* 12: 856429.
178. Trout JM, Santin M, Fayer R (2003) Identification of assemblage A *Giardia* in white-tailed deer. Journal of Parasitology 89: 1254-1255.
179. Santin M, Fayer R (2015) *Enterocytozoon bieneusi*, *Giardia* and *Cryptosporidium* infecting white-tailed deer. *Journal of Eukaryotic Microbiology* 62: 34-43.
180. Koehler AV, Haydon SR, Jex AR, Gasser RB (2016) *Cryptosporidium* and *Giardia* taxa in faecal samples from animals in catchments supplying the city of Melbourne with drinking water (2011 to 2015). *Parasites & Vectors* 9: 315.
181. Zhang Y, Mi R, Yang L, Gong H, Xu C, Feng Y, Chen X, Huang Y, Han X, Chen Z (2021) Wildlife is a potential source of human infections of *Enterocytozoon bieneusi* and *Giardia duodenalis* in southeastern China. *Frontiers in Microbiology* 12: 692837.
182. Li W, Deng L, Wu K, Huang X, Song Y, Su H, Hu Y, Fu H, Zhong Z, Peng G (2017) Presence of zoonotic *Cryptosporidium* *scrofarum*, *Giardia duodenalis* assemblage A and *Enterocytozoon bieneusi* genotypes in captive Eurasian wild boars (*Sus scrofa*) in China: potential for zoonotic transmission. *Parasites & Vectors* 10: 10.
183. Ghebremichael ST, Meng X, Yang Y, Andegiorgish AK, Wu Z, Chen J, Wei J, Li T, Bao J, Zhou Z, Pan G (2023) First identification and coinfection detection of *Enterocytozoon bieneusi*, *Encephalitozoon* spp., *Cryptosporidium* spp. and *Giardia duodenalis* in diarrheic pigs in southwest China. *BMC* *Microbiology* 23: 334.
184. Lee H, Kwak D (2023) Molecular detection and assemblage analysis of the intestinal protozoan *Giardia duodenalis* in wild boars in Korea. *Frontiers in Veterinary Science* 10: 1139060.
185. Marti-Marco A, Moratal S, Torres-Blas I, Cardells J, Lizana V, Dea-Ayuela MA (2023) Molecular detection of epidemiology of potentially zoonotic *Cryptosporidium* spp. and *Giardia duodenalis* in wild boar (*Sus scrofa*) from Eastern Spain. *Animals* 13: 2501.
186. Rodriguez-Rivera LD, Cummings KJ, McNeely I, Suchodolski JS, Scorza AV, Lappin MR, Mesenbrink BT, Leland BR, Bodenchuk MJ (2016) Prevalence and diversity of *Cryptosporidium* and *Giardia* identified among feral pigs in Texas. *Vector-Borne and Zoonotic Diseases* 16: 765-768.
187. Armson A, Yang R, Thompson J, Johnson J, Reid S, Ryan UM (2009) *Giardia* genotypes in pigs in Western Australia: prevalence and association with diarrhea. *Experimental Parasitology* 121: 381-383.
188. Farzan A, Parrington L, Coklin T, Cook A, Pintar K, Pollari F, Friendship R, Farber J, Dixon B (2011) Detection and characterization of *Giardia duodenalis* and *Cryptosporidium* spp. on swine farms in Ontario, Canada. *Foodborne Pathogens and Disease* 8: 1207-1213.
189. Wang SS, Yuan YJ, Yin YL, Hu RS, Song JK, Zhao GH (2017) Prevalence and multilocus genotyping of *Giardia duodenalis* in pigs of Shaanxi Province, northwestern China. *Parasites & Vectors* 10: 490.
190. Jing B, Zhang Y, Xu C, Li D, Xing J, Tao D, Zhang L, Qi M, Wang H (2019) Detection and genetic characterization of *Giardia duodenalis* in pigs from large-scale farms in Xinjiang, China. *Parasite* 26: 53.
191. Liu H, Xu N, Yin J, Yuan Z, Shen Y, Cao J (2019) Prevalence and multilocus genotyping of potentially zoonotic *Giardia duodenalis* in pigs in Shanghai, China. *Parasitology* 146: 1199-1205.
192. Zhang HJ, Song JK, Wu XM, Li YH, Wang Y, Lin Q, Zhao GH (2019) First report of *Giardia duodenalis* genotypes in Zangxiang pigs from China. *Parasitology Research* 118: 2305-2310.
193. Zou Y, Yuan XD, Zhang SY, Zhang HY, Chen XQ (2021) Molecular detection and characterization of *Giardia duodenalis* in farmed pigs in three provinces of Southern China. *Pathogens* 10: 1481.
194. Li D, Deng H, Zheng Y, Zhang H, Wang S, He L, Zhao J (2022) First characterization and zoonotic potential of *Cryptosporidium* spp. and *Giardia duodenalis* in pigs in Hubei province of China. *Frontiers in Cellular and Infection Microbiology* 12:949773.
195. Zhao FR, Zhang N, Miao WY, Wu R, Cui LL, Huang CQ, Zhou DH (2022) Molecular detection and multilocus genotyping of *Giardia duodenalis* in pigs in Fujian province, Southeastern China. *Animals* 12: 3148.
196. Maddox-Hyttel C, Langkjaer RB, Enemark HL, Vigre H (2006) *Cryptosporidium* and *Giardia* in different age groups of Danish cattle and pigs - occurrence and management associated risk factors. *Veterinary Parasitology* 141: 48-59.
197. Petersen HH, Jianmin W, Katakam KK, Mejer H, Thamsborg SM, Dalsgaard A, Olsen A, Enemark HL (2015) *Cryptosporidium* and *Giardia* in Danish organic pig farms: Seasonal and age-related variation in prevalence, infection intensity and species/genotypes. *Veterinary* *Parasitology* 214: 29-39.
198. Akinkuotu OA, Takeet MI, Otesile EB, Olufemi F, Greenwood SJ, McClure JT (2019) Prevalence and multilocus genotypes of *Giardia duodenalis* infecting pigs in Ogun state, Nigeria. *Infection, Genetics and Evolution* 70: 53-60.
199. Lee H, Jung B, Lim JS, Seo MG, Lee SH, Choi KH, Hwang MH, Kim TH, Kwon OD, Kwak D (2020) Multilocus genotyping of *Giardia duodenalis* from pigs in Korea. *Parasitology International* 78: 102154.
200. Zhao SS, Li YH, Zhang Y, Zhou Q, Jing B, Xu CY, Zhang LX, Song JK, Qi M, Zhao GH (2020) Multilocus genotyping of *Giardia duodenalis* in Bactrian camels (*Camelus bactrianus*) in China. *Parasitology Research* 119: 3873-3880.
201. Zhang K, Zheng S, Wang Y, Wang K, Wang Y, Gazizova A, Han K, Yu F, Chen Y, Zhang L (2021) Occurrence and molecular characterization of *Cryptosporidium* spp., *Giardia duodenalis*, *Enterocytozoon bieneusi*, and *Blastocystis* sp. in captive wild animals in zoos in Henan, China. *BMC* *Veterinary Research* 17: 332.
202. Koehler AV, Rashid MH, Zhang Y, Vaughan JL, Gasser RB, Jabbar A (2018) First cross-sectional, molecular epidemiological survey of *Cryptosporidium*, *Giardia* and *Enterocytozoon* in alpaca (*Vicugna pacos*) in Australia. *Parasites & Vectors* 11: 498.
203. Gómez-Couso H, Ortega-Mora LM, Aguado-Martínez A, Rosadio-Alcántara R, Maturrano-Hernández L, Espinoza LL, Zanabria-Huisa V, Pedraza-Díaz S (2012) Presence and molecular characterisation of *Giardia* and *Cryptosporidium* in alpacas (*Vicugna pacos*) from Peru. *Veterinary* *Parasitology* 187: 414-420.
204. Gomez-Puerta LA, Lopez-Urbina MT, Alarcon V, Cama V, Gonzalez AE, Xiao L (2014) Occurrence of *Giardia duodenalis* assemblages in alpacas in the Andean region. *Veterinary Parasitology* 63: 31-34.
205. Trout JM, Santin M, Fayer F (2008) Detection of Assemblage A, *Giardia duodenalis* and *Eimeria* spp. in alpacas on two Maryland farms. *Veterinary* *Parasitology* 153: 203-208.
206. Reboredo-Fernández A, Ares-Mazás E, Martínez-Cedeira JA, Romero-Suances R, Cacciò SM, Gómez-Couso H (2014) *Giardia* and *Cryptosporidium* in cetaceans on the European Atlantic coast. *Parasitology Research* 114: 693-698.
207. Lasek-Nesselquist E, Welch DM, Sogin ML (2010) The identification of a new *Giardia duodenalis* assemblage in marine vertebrates and a preliminary analysis of *G. duodenalis* population biology in marine systems. *International Journal for Parasitology* 40: 1063-1074.
208. Reboredo-Fernández A, Gómez-Couso H, Martínez-Cedeira JA, Cacciò SM, Ares-Mazás E (2014) Detection and molecular characterization of *Giardia* and *Cryptosporidium* in common dolphins (*Delphinus delphis*) stranded along the Galician coast (Northwest Spain). *Veterinary* *Parasitology*. 202: 132-137.
209. Marangi M, Carlucci R, Carlino P, Fanizza C, Cirelli G, Maglietta R, Beneduce L (2022) Dolphins and sea turtles may host zoonotic parasites and pathogenic bacteria as indicators of anthropic pressure in the Gulf of Taranto (Northern Ionian Sea, Central-Eastern Mediterranean Sea). *Veterinary Research Communications* 46: 1157-1166.
210. Claerebout E, Casaert S, Dalemans AC, DeWilde N, Levecke B, Vercruysse J, Geurden T (2009) *Giardia* and other intestinal parasites in different dog populations in Northern Belgium. *Veterinary Parasitology* 161: 41-46.
211. Volotao AC, Costa-Macedo LM, Haddad FSM, Brandão A, Peralta JM, Fernandes O (2007) Genotyping of *Giardia duodenalis* from human and animal samples from Brazil using ꞵ-giardin gene: a phylogenetic analysis. *Acta Tropica* 102: 10-19.
212. Paz e Silva FM, Monobe MM, Lopes RS, Araujo Jr. JP (2012) Molecular characterization of *Giardia duodenalis* in dogs from Brazil. *Parasitology* *Research* 110: 325-33.
213. de Godoy EAM, Santos Junior JE, Belloto MVT, de Moraes MVP, Cassiano GC, Volotão ACC, Luvizotto MCR, Carareto CMA, de Moraes Silva MC, Machado RLD (2013) Molecular investigation of zoonotic genotypes of *Giardia intestinalis* isolates in humans, dogs and cats, sheep, goats and Cattle in Araçatuba (São Paulo State, Brazil) by the analysis of β-giardin gene fragments. *Microbiology Research* 4: e6.
214. Quadros RMd, Wiess PHE, Marques SMT, Miletti LC (2016) Potential cross-contamination of similar *Giardia* *duodenalis* assemblages in children and pet dogs in southern Brazil, as determined by PCR-RFLP. *Revista do Instituto de Medicina Tropical de Sao Paulo* 58: 66.
215. Trevisan YPA, de Almeida AdBPF, Nakazato L, Pacheco TdA, de Souza LI, Canei DH, Pereira ME, Maia MO, Pacheco RC, Sousa VRF (2020) Frequency of *Giardia duodenalis* infection and its genetic variability in dogs in Cuiabá, Midwest Brazil. *The Journal of Infection in Developing Countries* 14: 1431-1436.
216. Harvey TV, Carvalho JPS, Aquino MCC, Oliveira BCM, Barros LD, Fehlberg HF, Rocha CMBM, Albuquerque GR (2023) Giardiasis in children and dogs, and the first report of assemblage E in dogs from northeastern Brazil. *Brazilian Journal of Veterinary Parasitology* 32(1): e012222.
217. Himsworth CG, Skinner S, Chaban B, Jenkins E, Wagner BA, Harms NJ, Leighton FA, Thompson RC, Hill JE (2010) Multiple zoonotic pathogens identified in canine feces collected from a remote Canadian indigenous community. *American Journal of Tropical Medicine and Hygiene* 83: 338-341
218. Schurer JM, Hill JE, Fernando C, Jenkins EJ (2012) Sentinel surveillance for zoonotic parasites in companion animals in indigenous communities of Saskatchewan. *American Journal of Tropical Medicine and Hygiene* 87: 495-498.
219. Uehlinger FD, Greenwood SJ, McClure JT, Conboy G, O’Handley R, Barkema HW (2013) Zoonotic potential of *Giardia duodenalis* and *Cryptosporidium* spp. and prevalence of intestinal parasites in young dogs from different populations on Prince Edward Island, Canada. *Veterinary Parasitology* 196: 509-514.
220. Julien DA, Sargeant JM, Guy RA, Shapiro K, Imai RK, Bunce A, Sudlovenick E, Chen S, Li J, Harper SL (2019) Prevalence and genetic characterization of *Giardia* spp. and *Cryptosporidium* spp. in dogs in Iqaluit, Nunavut, Canada. *Zoonoses and Public Health* 66: 813-825.
221. Smith AF, Neumann N, Banting G, Claudia Klein C, Liccioli S, Massolo A (2020) Molecular characterization of *Giardia* spp. and *Cryptosporidium* spp. from dogs and coyotes in an urban landscape suggests infrequent occurrence of zoonotic genotypes. *Veterinary Parasitology* 281: 109115.
222. Li J, Zhang P, Wang P, Alsarakibi M, Zhu H, Liu Y, Meng X, Li J, Guo J, Li G (2012) Genotype identification and prevalence of *Giardia duodenalis* in pet dogs of Guangzhou, southern China. *Veterinary Parasitology* 188: 368-371.
223. Li W, Liu C, Yu Y, Li J, Gong P, Song M, Xiao L, Zhang X (2013) Molecular characterization of *Giardia* *duodenalis* isolates from police and farm dogs in China. *Experimental Parasitology* 135: 223-226.
224. Zheng G, Alsarakibi M, Liu Y, Hu W, Luo Q, Tan L, Li G (2014) Genotyping of *Giardia duodenalis* isolates from dogs in Guangdong, China based on multilocus sequence. *Korean Journal of Parasitology* 52: 299-304.
225. Gu YF, Wang K, Liu DY, Mei N, Chen C, Chen T, Han MM, Zhou L, Cao JT, Zhang H, Zhang XL, Fan ZL, Li WC (2015) Molecular detection of *Giardia lamblia* and *Cryptosporidium* species in pet dogs. *Zhongguo Ji Sheng Chong Xue Yu Ji Sheng Chong Bing Za Zhi* 33: 362-367. (In Chinese)
226. Li W, Li Y, Song M, Lu Y, Yang J, Tao W, Jiang Y, Wan Q, Zhang S, Xiao L (2015) Prevalence and genetic characteristics of *Cryptosporidium*, *Enterocytozoon bieneusi* and *Giardia duodenalis* in cats and dogs in Heilongjiang province, China. *Veterinary Parasitology* 208: 125-134.
227. Qi M, Dong H, Wang R, Li J, Zhao J, Zhang L, Luo J (2016) Infection rate and genetic diversity of *Giardia duodenalis* in pet and stray dogs in Henan Province, China. *Parasitology International* 65: 159-162.
228. Xu H, Jin Y, Wu W, Li P, Wang L, Li N, Feng Y, Xiao L (2016) Genotypes of *Cryptosporidium* spp., *Enterocytozoon bieneusi* and *Giardia duodenalis* in dogs and cats in Shanghai, China. *Parasites & Vectors* 9: 121.
229. Zhang Y, Zhong Z, Deng L, Wang M, Li W, Gong C, Fu H, Cao S, Shi X, Wu K, Peng G (2017) Detection and multilocus genotyping of *Giardia duodenalis* in dogs in Sichuan province, China. *Parasite* 24: 31.
230. Pan W, Wang M, Abdullahi AY, Fu Y, Yan X, Yang F, Shi X, Zhang P, Hang J, Li G (2018) Prevalence and genotypes of *Giardia lamblia* from stray dogs and cats in Guangdong, China. *Veterinary Parasitology: Regional Studies and Reports* 13: 30-34.
231. Yu Z, Ruan Y, Zhou M, Chen S, Zhang Y, Wang L, Zhu G, Yu Y (2018) Prevalence of intestinal parasites in companion dogs with diarrhea in Beijing, China, and genetic characteristics of *Giardia* and *Cryptosporidium* species. *Parasitology Research* 117: 35-43.
232. Li J, Dan X, Zhu K, Li N, Guo Y, Zheng Z, Feng Y, Xiao L (2019) Genetic characterization of *Cryptosporidium* spp. and *Giardia duodenalis* in dogs and cats in Guangdong, China. *Parasites & Vectors* 12: 571.
233. Liao S, Lin X, Sun Y, Qi N, Lv M, Wu C, Li J, Hu J, Yu L, Cai H, Xiao W, Sun M, Li G (2020) Occurrence and genotypes of *Cryptosporidium* spp., *Giardia* *duodenalis*, and *Blastocystis* sp. in household, shelter, breeding, and pet market dogs in Guangzhou, southern China. *Scientific Reports* 10: 17736.
234. Cao Y, Fang C, Deng J, Yu F, Ma D, Chuai L, Wang T, Qi M, Li J (2022) Molecular characterization of *Cryptosporidium* spp. and *Giardia duodenalis* in pet dogs in Xinjiang, China. *Parasitology Research* 121: 1429-1435.
235. Sui Y, Zhang X, Wang H, Yu F, Zheng L, Guo Y, Lu Y, Chen M, Wang B, Dai H, Liu F, Li J, Dong H, Tong C, Zhang L (2022) Prevalence and genetic diversity of *Giardia duodenalis* in pet dogs from Zhengzhou, central China and the association between gut microbiota and fecal characteristics during infection. *One Health* 14: 100401.
236. Zhang J, Qin Y, Shen Y, Wang Y, Cao J, Su Y, Liu H (2023) Prevalence and genotyping of *Cryptosporidium* spp. and *Giardia lamblia* in dogs and cats from a pet hospital in Shanghai Municipality. *Zhongguo Xue Xi Chong Bing Fang Zhi Za Zhi* 35: 258-262. (In Chinese)
237. Scorza AV, Duncan C, Miles L, Lappin MR (2011) Prevalence of selected zoonotic and vector-borne agents in dogs and cats in Costa Rica. *Veterinary Parasitology* 183: 178-183.
238. Puebla LEJ, Núñez FA., Rivero LR, Hernández YR, Millán IA, Müller N (2017) Prevalence of intestinal parasites and molecular characterization of *Giardia duodenalis* from dogs in La Habana, Cuba. *Veterinary Parasitology: Regional Studies and Reports* 8: 107-112.
239. Gingrich EN, Scorza AV, Clifford EL, Olea-Popelka FJ, Lappin MR (2010) Intestinal parasites of dogs on the Galapagos Islands. *Veterinary* *Parasitology* 169: 404-407.
240. Abdelaziz AR, Sorour SSG (2021) Prevalence and molecular characterization of *Giardia duodenalis* assemblage D of dogs in Egypt, and its zoonotic implication. *Microbes, Infection and Chemotherapy* 1: e1268.
241. Elmahallawy EK, Gareh A, Abu-Okail A, Köster PC, Dashti A, Asseri J, Gouda AA, Mubaraki MA, Mohamed SA-A, Mohamed YM, Hassan EA, Elgendy M, Hernández-Castro C, Bailo B, González-Barrio D, Xiao L, Carmena D (2023) Molecular characteristics and zoonotic potential of enteric protists in domestic dogs and cats in Egypt. Frontiers in Veterinary Science 10: 1229151.
242. Rimhanen-Finne R, Enemark HL, Kolehmainen J, Toropainen P, Han-ninen ML (2007) Evaluation of immunofluorescence microscopy and enzyme-linked immunosorbent assay in detection of *Cryptosporidium* and *Giardia* infections in asymptomatic dogs. *Veterinary Parasitology* 145: 345-348.
243. Leonhard S, Pfister K, Beelitz P, Wielinga C, Thompson RCA (2007) The molecular characterisation of *Giardia* from dogs in southern Germany. *Veterinary* *Parasitology* 150: 33-38.
244. Sommer MF, Rupp P, Pietsch M, Kaspar A, Beelitz P (2018) *Giardia* in a selected population of dogs and cats in Germany - diagnostics, coinfections and assemblages. *Veterinary Parasitology* 249: 49-56.
245. Murnik LC, Daugschies A, Delling C (2023) Gastrointestinal parasites in young dogs and risk factors associated with infection. *Parasitology* *Research* 122: 585-596.
246. Kostopoulou D, Claerebout E, Arvanitis D, Ligda P, Voutzourakis N, Casaert S, Sotiraki S (2017) Abundance, zoonotic potential and risk factors of intestinal parasitism amongst dog and cat populations: The scenario of Crete, Greece. *Parasites & Vectors* 10: 43.
247. Szenasi Z, Marton S, Kucsera I, Tánczos B, Horváth K, Orosz E, Lukács Z, Szeidemann Z (2007) Preliminary investigation of the prevalence and genotype distribution of *Giardia intestinalis* in dogs in Hungary. *Parasitology Research* 101: S145-S152.
248. Utaaker KS, Tysnes KR, Krosness MM, Robertson LJ (2018) Not just a walk in the park: Occurrence of intestinal parasites in dogs roaming recreational parks in Chandigarh, Northern India. *Veterinary Parasitology: Regional Studies and Reports* 14: 176-180.
249. Homayouni MM, Razavi SM, Shaddel M, Asadpour M (2019) Prevalence and molecular characterization of *Cryptosporidium* spp. and *Giardia intestinalis* in household dogs and cats from Shiraz, Southwestern Iran. *Veterinaria Italiana* 55: 311-318.
250. Esmailzadeh R, Malekifard F, Rakhshanpour A, Tavassoli M (2023) Frequency and genotyping of *Giardia duodenalis* in dogs of Urmia, northwest of Iran. *Veterinary Research Forum* 14: 335-340.
251. Salant H, Kuzi S, Navarro D, Baneth G (2020) Prevalence and molecular characterization of *Giardia duodenalis* in dogs in Israel. *Comparative Immunology, Microbiology and Infectious Diseases* 73: 101548.
252. Paoletti B, Iorio R, Capelli G, Sparagano OAE, Giangaspero A (2008) Epidemiological scenario of giardiosis in dogs from central Italy. *Animal* *Biodiversity and Emerging Diseases* 1149: 371-374.
253. Papini R, Marangi M, Mancianti F, Giangaspero A (2009) Occurrence and cyst burden of *Giardia* *duodenalis* in dog faecal deposits from urban green areas: implications for environmental contamination and related risks. *Preventive Veterinary Medicine* 92: 158-162.
254. Scaramozzino P, Di Cave D, Berrilli F, D’Orazi C, Spaziani A, Mazzanti S, Scholl F, De Liberato C (2009) A study of the prevalence and genotypes of *Giardia duodenalis* infecting kennelled dogs. *The Veterinary Journal* 182: 231-234.
255. Marangi M, Berrilli F, Otranto D, Giangaspero A (2010) Genotyping of *Giardia duodenalis* among children and dogs in a closed society deprived community from Italy. *Zoonoses and Public Health* 57: e54-8.
256. Riggio F, Mannella R, Ariti G, Perrucci S (2013) Intestinal and lung parasites in owned dogs and cats from central Italy. *Veterinary Parasitology* 193: 78-84.
257. Pipia AP, Varcasia A, Tamponi C, Sanna G, Soda M, Paoletti B, Traversa D, Scala A (2014) Canine giardiosis in Sardinia Island, Italy: prevalence, molecular characterization, and risk factors. *The Journal of Infection in Developing Countries* 8: 655-660.
258. Zanzani SA, Gazzonis AL, Scarpa P (2014) Intestinal Parasites of owned dogs and cats from metropolitan and micropolitan areas: prevalence, zoonotic risks, and pet owner awareness in Northern Italy. *BioMed Research International* Article ID: 696508.
259. Paoletti B, Traversa D, Iorio R, De Berardinis A, Bartolini R, Salini R, Di Cesare A (2015) Zoonotic parasites in feces and fur of stray and private dogs from Italy. *Parasitology Research* 114: 2135-41.
260. Simonato G, di Regalbono AF, Cassini R, Traversa D, Beraldo P, Tessarin C, Pietrobelli M (2015) Copromicroscopic and molecular investigations on intestinal parasites in kenneled dogs. *Parasitology Research* 114: 1963-1970.
261. De Liberato C, Berrilli F, Odorizi L, Scarcella R, Barni M, Amoruso C, Scarito A, Di Filippo MM, Carvelli A, Iacoponi F, Scaramozzino P (2018) Parasites in stray dogs from Italy: prevalence, risk factors and management concerns. *Acta Parasitologica* 63: 27-32.
262. Sauda F, Malandrucco L, Macrì G, Scarpulla M, De Liberato C, Terracciano G, Fichi G, Berrilli F, Perrucci S (2018) *Leishmania* *infantum*, *Dirofilaria* spp. and other endoparasite infections in kennel dogs in central Italy. *Parasite* 25: 2.
263. Perrucci S, Berrilli F, Procopio C, Di Filippo MM, Pierini A, Marchetti V (2020) *Giardia duodenalis* infection in dogs affected by primary chronic enteropathy. *Open Veterinary Journal* 10: 74-79.
264. Agresti A, Berrilli F, Maestrini M, Guadano Procesi I, Loretti E, Vonci N, Perrucci S (2022) Prevalence, risk factors and genotypes of *Giardia duodenalis* in sheltered dogs in Tuscany (central Italy). *Pathogens* 11: 12.
265. Lee MF, Cadogan P, Copeland S, Walochnik J, Lindo JF (2017) Molecular epidemiology and multilocus sequence analysis of potentially zoonotic *Giardia* spp. from humans and dogs in Jamaica. *Parasitology Research* 116: 406-414.
266. Yoshiuchi R, Matsubayashi M, Kimata I, Furuya M, Tani H, Sasai K (2010) Survey and molecular characterization of *Cryptosporidium* and *Giardia* spp. in owned companion animal, dogs and cats, in Japan. *Veterinary Parasitology* 174: 313-316.
267. Itoh N, Itagaki T, Kawabata T, Konaka T, Muraoka N, Saeki H, Kanai K, Chikazawa S, Hori Y, Hoshi F, Higuchi S (2011) Prevalence of intestinal parasites and genotyping of *Giardia intestinalis* in pet shop puppies in east Japan. *Veterinary* *Parasitology* 176: 74-78.
268. Eligio-Garcia L, Cortes-Campos A, Jimenez-Cardoso E (2005) Genotype of *Giardia intestinalis* isolates from children and dogs and its relationship to host origin. *Parasitology Research* 97: 1-6.
269. Jiménez-Cardoso E, Eligio-García L, Cortés-Campos A, Estrada AC, Pinto-Sagahón M, Noguera-Estrada C (2010) The frequency of intestinal parasites in puppies from Mexican kennels. *Health* 2: 1316-1319.
270. Godínez-Galaz EM, Veyna-Salazar NP, Olvera-Ramírez AM, Milián-Suazo F, Perea-Razo CA, Bernal-Reynaga R, Cantó-Alarcón GJ (2019) Prevalence and zoonotic potential of *Giardia intestinalis* in dogs of the central region of Mexico. *Animals* 9: 325.
271. Overgaauw PAM, van Zutphen L, Hoek D, Yaya FO, Roelfsema J, Pinelli E, van Knapen F, Kortbeek LM (2009) Zoonotic parasites in fecal samples and fur from dogs and cats in The Netherlands. *Veterinary Parasitology* 163: 115-122.
272. Zygner W, Jaros D, Skowrońska M, Bogdanowicz-Kamirska M, Wędrychowicz H (2006) Prevalence of *Giardia intestinalis* in domestic dogs in Warsaw. *Wiadomosci Parazytologiczne* 52: 311-315. (In Polish)
273. Solarczyk P, Majewska AC (2010) A survey of the prevalence and genotypes of *Giardia duodenalis* infecting household and sheltered dogs. *Parasitology Research* 106: 1015-1019.
274. Bajer A, Bednarska M, Rodo A (2011) Risk factors and control of intestinal parasite infections in sled dogs in Poland. *Veterinary Parasitology* 175: 343-350.
275. Piekarska J, Bajzert J, Gorczykowski M, Kantyka M, Podkowik M (2016) Molecular identification of *Giardia duodenalis* isolates from domestic dogs and cats in Wroclaw, Poland. *Annals of Agricultural Environmental Medicine* 23: 410-415.
276. Piekara-Stepinska A, Piekarska J, Gorczykowski M, Bania J (2021) Genotypes of *Giardia duodenalis* in household dogs and cats from Poland. *Acta* *Parasitologica* 66: 428-435.
277. Ferreira FS, Pereira-Baltasar P, Parreira R, Padre L, Vilhena M, Tavora Tavira L, Atouguia J, Centeno-Lima S (2011) Intestinal parasites in dogs and cats from the district of Evora, Portugal. *Veterinary Parasitology* 179: 242-245.
278. Pereira A, Teixeira J, Sousa S, Parreira R, Campino L, Meireles J, Maia C (2021) *Giardia duodenalis* infection in dogs from the metropolitan area of Lisbon, Portugal: prevalence, genotyping and associated risk factors. *Journal of Parasitic Diseases* 45: 372-379.
279. Shin JC, Wehdnesday Bernardo Reyes A, Kim SH, Kim S, Park HJ, Seo KW, Song KH (2015) Molecular detection of *Giardia intestinalis* from stray dogs in animal shelters of Gyeongsangbuk-do (Province) and Daejeon, Korea. *Korean Journal of Parasitology* 53: 477-481.
280. Kim HY, Lee H, Lee SH, Seo MG, Yi S, Kim JW, Kim CH, Lee YR, So BJ, Kwon OD, Kwak D (2019) Multilocus genotyping and risk factor analysis of *Giardia duodenalis* in dogs in Korea. *Acta Tropica* 199: 105113.
281. Dado D, Montoya A, Blanco MA, Miró G, Saugar JM, Bailo B, Fuentes I (2012) Prevalence and genotypes of *Giardia duodenalis* from dogs in Spain: possible zoonotic transmission and public health importance. *Parasitology Research* 111: 2419-2422.
282. Ortuno A, Scorza V, Castella J, Lappin M (2014) Prevalence of intestinal parasites in shelter and hunting dogs in Catalonia, Northeastern Spain. *The Veterinary Journal* 199: 465-467.
283. de Lucio A, Bailo B, Aguilera M, Cardona GA, Fernandez-Crespo JC, Carmena D (2017) No molecular epidemiological evidence supporting household transmission of zoonotic *Giardia* *duodenalis* and *Cryptosporidium* spp. from pet dogs and cats in the province of Álava, Northern Spain. *Acta* *Tropica* 170: 48-56.
284. Gil H, Cano L, de Lucio A, Bailo B, de Mingo MH, Cardona GA, Fernández-Basterra JA, Aramburu-Aguirre J, López-Molina N, Carmena D (2017) Detection and molecular diversity of *Giardia duodenalis* and *Cryptosporidium* spp. in sheltered dogs and cats in Northern Spain. *Infection, Genetics and Evolution* 50: 62-69.
285. Adell-Aledón M, Köster PC, de Lucio A, Puente P, Hernández-de-Mingo M, Sánchez-Thevenet P, Dea-Ayuela MA, Carmena D (2018) Occurrence and molecular epidemiology of *Giardia duodenalis* infection in dog populations in eastern Spain. *BMC Veterinary Research* 14: 26.
286. Mateo M, Montoya A, Bailo B, Köster PC, Dashti A, Hernández-Castro C, Saugar JM, Matas P, Xiao L, Carmena D (2023) Prevalence and public health relevance of enteric parasites in domestic dogs and cats in the region of Madrid (Spain) with an emphasis on *Giardia* *duodenalis* and *Cryptosporidium* sp.. *Veterinary Medicine and Science* 9: 2542-2558.
287. Tseng YC, Ho GD, Chen TT, Huang BF, Cheng PC, Chen JL, Peng SY (2014) Prevalence and genotype of *Giardia duodenalis* from faecal samples of stray dogs in Hualien city of eastern Taiwan. *Tropical Biomedicine* 31: 305-311.
288. Inpankaew T, Traub R, Thompson RC, Sukthana Y (2007) Canine parasitic zoonoses in Bangkok temples. *The Southeast Asian Journal of Tropical Medicine and Public Health* 38: 247-255.
289. Traub RJ, Inpankaew T, Reid SA, Sutthikornchai C, Sukthana Y, Robertson ID, Thompson RCA (2009) Transmission cycles of *Giardia duodenalis* in dogs and humans in temple communities in Bangkok - a critical evaluation of its prevalence using three diagnostic tests in the field in the absence of a gold standard. *Acta Tropica* 111: 125-132.
290. Tangtrongsup S, Scorza AV, Reif JS, Ballweber LR, Lappin MR, Salman MD (2017) Prevalence and multilocus genotyping analysis of *Cryptosporidium* and *Giardia* isolates from dogs in Chiang Mai, Thailand. *Veterinary Sciences* 4: 26.
291. Tangtrongsup S, Scorza AV, Reif JS, Ballweber LR, Lappin MR, Salman MD (2020) Seasonal distributions and other risk factors for *Giardia duodenalis* and *Cryptosporidium* spp. infections in dogs and cats in Chiang Mai, Thailand. *Preventive Veterinary Medicine* 174: 104820.
292. Mark-Carew MP, Adesiyun AA, Basu A, Georges KA, Pierre T, Tilitz S, Wade SE, Mohammed HO (2013) Characterization of *Giardia duodenalis* infections in dogs in Trinidad and Tobago. *Veterinary Parasitology* 196: 199-202.
293. Celik BA, Çelik OY, Koçhan A, Ayan A, Kilinc OO, Akyıldız G, İrak K, Ayan OO, Ercan K (2023) Prevalence and genotypes of *Giardia duodenalis* in shelter dogs of southeastern Türkiye. *Veterinary Research Forum* 14: 595-599.
294. Ipek DNS (2023) Molecular characterization and zoonotic significance of *Cryptosporidium spp.* and *Giardia duodenalis* in asymptomatic adult stray cats and dogs in Turkey. *Indian Journal of Animal Research* DOI: 10.18805/IJAR.BF-1707.
295. Upjohn M, Cobb C, Monger J, Geurden T, Claerebout E, Fox M (2010) Prevalence, molecular typing and risk factor analysis for *Giardia duodenalis* infections in dogs in a central London rescue shelter. *Veterinary Parasitology* 172: 341-346.
296. Wang A, Ruch-Gallie R, Scorza V, Lin P, Lappin MR (2012) Prevalence of *Giardia* and *Cryptosporidium* species in dog park attending dogs compared to non-dog park attending dogs in one region of Colorado. *Veterinary Parasitology* 184: 335-340.
297. Johansen KM, Castro NS, Lancaster KE, Madrid E, Havas A, Simms J, Sterling CR (2014) Characterization of *Giardia lamblia* genotypes in dogs from Tucson, Arizona using SSU-rRNA and beta-giardin sequences. *Parasitology Research* 113: 387-390.
298. Hascall KL, Kass PH, Saksen J, Ahlmann A, Scorza AV, Lappin MR, Marks SL (2016) Prevalence of enteropathogens in dogs attending 3 regional dog parks in Northern California. *Journal of Veterinary Internal Medicine* 30: 1838-1845.
299. Thompson RCA, Colwell DD, Shury T, Appelbee AJ, Read C, Njiru Z, Olson ME (2009) The molecular epidemiology of *Cryptosporidium* and *Giardia* infections in coyotes from Alberta, Canada, and observations on some cohabiting parasites. *Veterinary Parasitology* 159: 167-170.
300. Trout J, Santin M, Fayer R (2006) *Giardia* and *Cryptosporidium* species and genotypes in coyotes (*Canis latrans*). *Journal of Zoo and Wildlife Medicine* 37:141-144.
301. Zhang XX, Zheng WB, Ma JG, Yao QX, Zou Y, Bubu CJ, Zhao Q, Zhu XQ (2016) Occurrence and multilocus genotyping of *Giardia intestinalis* assemblage C and D in farmed raccoon dogs, *Nyctereutes procyonoides*, in China. *Parasites & Vectors* 9: 471.
302. Solarczyk P, Majewska AC, Jędrzejewski S, Górecki MT, Nowicki S, Przysiecki P (2016) First record of *Giardia* assemblage D infection in farmed raccoon dogs (*Nyctereutes procyonoides*). *Annals of Agricultural and Environmental Medicine* 23: 696-698
303. Hamnes IS, Gjerde BK, Forberg T, Robertson LJ (2007) Occurrence of *Giardia* and *Cryptosporidium* in Norwegian red foxes (*Vulpes vulpes*). *Veterinary Parasitology* 143: 347-353.
304. Onac D, Oltean M, Mircean V, Jarca A, Cozma V (2015) Occurrence of *Giardia duodenalis* zoonotic assemblages in red foxes from Romania. Science Parasitology 16: 177-180.
305. Debenham JJ, Landuyt H, Troell K, Tysnes K, Robertson LJ (2017) Occurrence of *Giardia* in Swedish red foxes (*Vulpes vulpes*). *Journal of Wildlife* *Diseases* 53: 649-652.
306. Maestrini M, Berrilli F, Di Rosso A, Coppola F, Guadano Procesi I, Mariacher A, Felicioli A, Perrucci S (2022) Zoonotic *Giardia duodenalis* genotypes and other gastrointestinal parasites in a badger population living in an anthropized area of central Italy. *Pathogens* 11: 906.
307. Delport TC, Asher AJ, Beaumont LJ, Webster KN, Harcourt RG, Power ML (2014) *Giardia duodenalis* and *Cryptosporidium* occurrence in Australian sea lions (*Neophoca cinerea*) exposed to varied levels of human interaction. *International Journal for Parasitology:* *Parasites and* *Wildlife* 3: 269-275.
308. Dixon BR, Parrington LJ, Parenteau M, Leclair D, Santin M, Fayer R (2008) *Giardia duodenalis* and *Cryptosporidium* spp. in the intestinal contents of ringed seals (*Phoca hispida*) and bearded seals (*Erignathus barbatus*) in Nunavik, Quebec, Canada. *Journal of Parasitology* 94: 1161-1163.
309. Gaydos JK, Miller WA, Johnson C, Zornetzer H, Melli A, Packham A, Jeffries SJ, Lance MM, Conrad PA (2008) Novel and canine genotypes of *Giardia duodenalis* in harbor seals (*Phoca vitulina richardsi*). *Journal of Parasitology* 94: 1264-1268.
310. Yang R, Ying JLJ, Monis P, Ryan U (2015) Molecular characterisation of *Cryptosporidium* and *Giardia* in cats (*Felis catus*) in Western Australia. *Experimental Parasitology* 155: 13-18.
311. Hinney B, Ederer C, Stengl C, Wilding K, Strkolcova G, Harl J, Flechl E, Fuehrer HP, Joachim A (2015) Enteric protozoa of cats and their zoonotic potential-a field study from Austria. *Parasitology Research* 114: 2003-2006.
312. Joachim A, Auersperg V, Drue J, Wiedermann S, Hinney B, Spergser J (2023) Parasites and zoonotic bacteria in the feces of cats and dogs from animal shelters in Carinthia, Austria. *Research in Veterinary Science* 164: 105022.
313. Zheng G, Hu W, Liu Y, Luo Q, Tan L, Li G (2015) Occurrence and molecular identification of *Giardia duodenalis* from stray cats in Guangzhou, Southern China. *Korean Journal of Parasitology* 53: 119-124.
314. Li W, Liu X, Gu Y, Liu J, Luo J (2019) Prevalence of *Cryptosporidium*, *Giardia*, *Blastocystis*, and trichomonads in domestic cats in East China. *The* *Journal of Veterinary Medical Sciences* 81: 890-896.
315. Zhang J, Qin Y, Shen Y, Wang Y, Cao J, Su Y, Liu H (2023) Prevalence and genotyping of *Cryptosporidium* spp. and *Giardia lamblia* in dogs and cats from a pet hospital in Shanghai Municipality. *Zhongguo Xue Xi Chong Bing Fang Zhi Za Zhi* 35: 258-262. (In Chinese)
316. Santin M, Trout JM, Vecino JAC, Dubey JP, Fayer R (2006) *Cryptosporidium*, *Giardia* and *Enterocytozoon* *bieneusi* in cats from Bogota (Colombia) and genotyping of isolates. *Veterinary Parasitology* 141: 334-339.
317. Enemark HL, Starostka TP, Larsen B, Takeuchi-Storm N, Thamsborg SM (2020) *Giardia* and *Cryptosporidium* infections in Danish cats: risk factors and zoonotic potential. *Parasitology Research* 119: 2275-2286.
318. Pezeshki A, Rezaeian M, Zarebavani M (2005) Study of the genetic resemblance of tpi gene in cat and human *Giardia* using PCR-RFLP method. *Journal of Ardabil University of Medical Sciences* 13: 164-173.
319. Karimi P, Shafaghi‑Sisi S, Meamar AR, Razmjou E (2023) Molecular identification of *Cryptosporidium*, *Giardia*, and *Blastocystis* from stray and household cats and cat owners in Tehran, Iran. *Scientific Reports* 13: 1554.
320. Paoletti B, Otranto D, Weigl S, Giangaspero A, Di Cesare A, Traversa D (2011) Prevalence and genetic characterization of *Giardia* and *Cryptosporidium* in cats from Italy. *Research in Veterinary Science* 91: 397-399.
321. Mancianti F, Nardoni S, Mugnaini L, Zambernardi L, Guerrini A, Gazzola V, Papini RA (2015) A retrospective molecular study of select intestinal protozoa in healthy pet cats from Italy. *Journal of Feline Medicine and Surgery* 17: 163-167.
322. Procesi IG, Carnio A, Berrilli F, Di Filippo MM, Scarito A, Amoruso C, Barni M, Ruffini M, Barlozzari G, Scarpulla M, De Liberato C (2022) *Giardia duodenalis* in colony stray cats from Italy. *Zoonoses and Public Health* 69: 46-54.
323. Suzuki J, Murata R, Kobayashi S, Sadamasu K, Kai A, Takeuchi T (2011) Risk of human infection with *Giardia duodenalis* from cats in Japan and genotyping of the isolates to assess the route of infection in cats. *Parasitology* 138: 493-500.
324. Ito Y, Iijima Y, Itoh N, Kimura Y (2017) Multilocus genotyping of *Giardia duodenalis* isolates from breeding cattery cats in Japan. *Journal of Feline Medicine and Surgery Open Reports* 3: 2055116917745237.
325. Jaros D, Zygner W, Jaros S, Wedrychowicz H (2011) Detection of *Giardia intestinalis* assemblages A, B and D in domestic cats from Warsaw, Poland. *Polish Journal of Microbiology* 60: 259-263.
326. Kwak D, Seo MG (2020) Genetic analysis of zoonotic gastrointestinal protozoa and microsporidia in shelter cats in South Korea. *Pathogens* 9: 894.
327. Yun CS, Moon BY, Lee K, Kang SM, Ku BK, Hwang MH (2023) The detection and phylogenetic characterization of *Cryptosporidium*, *Cystoisospora*, and *Giardia duodenalis* of cats in South Korea. *Frontiers in Cellular and Infection Microbiology* 13: 1296118.
328. Sursal N, Simsek E, Yildiz K (2020) Feline giardiasis in Turkey: prevalence and genetic and haplotype diversity of *Giardia duodenalis* based on the ꞵ-giardin gene sequence in symptomatic cats. *The Journal of Parasitology* 106: 699-706.
329. Celik BA, Celik OY, Ayan A, Akyildiz G, Kilinc OO, Ayan OO, Ercan K (2023) Preliminary investigation of the prevalence and genotype distribution of *Cryptosporidium* spp., and *Giardia duodenalis* in cats in Siirt, Turkey. *Acta Veterinaria* 73: 317-324.
330. Krumrie S, Capewell P, McDonald M, Dunbar D, Panarese R, Katzer F, El Sakka N, Mellor D, Alexander CL, Weir W (2022) Molecular characterisation of *Giardia duodenalis* from human and companion animal sources in the United Kingdom using an improved triosephosphate isomerase molecular marker. *Current Research in Parasitology & Vector-Borne Diseases* 2: 100105.
331. Vasilopulos RJ, Rickard LG, Mackin AJ, Pharr GT, Huston CL (2007) Genotypic analysis of *Giardia duodenalis* in domestic cats. *Journal of Veterinary Internal Medicine* 21: 352-355.
332. Solarczyk P, Osten-Sacken N, Frantz AC, Schneider S, Pir JB, Heddergott M (2019) First molecular detection of *Giardia duodenalis* assemblage B in a free-living European wildcat (*Felis s. silvestris*) from Luxembourg. *Acta Protozoologica* 58: 1-5.
333. Yu Z, Wen X, Huang X, Yang R, Guo Y, Feng Y, Xiao L, Li N (2020) Molecular characterization and zoonotic potential of *Enterocytozoon bieneusi*, *Giardia* *duodenalis* and *Cryptosporidium* sp. in farmed masked palm civets (*Paguma larvata*) in southern China. *Parasites & Vectors* 13: 403.
334. Thompson J, Yang R, Power M, Hufschmid J, Beveridge I, Reid S, Ng J, Armson A, Ryan U (2008) Identification of zoonotic *Giardia* genotypes in marsupials in Australia. *Experimental Parasitology* 120: 88-93
335. Thompson RCA, Smith A, Lymbery AJ, Averis S, Morris KD, Wayne AF (2010) *Giardia* in Western Australia wildlife. *Veterinary Parasitology* 170: 207-211.
336. Wait LF, Fox S, Peck S, Power ML (2017) Molecular characterization of *Cryptosporidium* and *Giardia* from the Tasmanian devil (*Sarcophilus* *harrisii*). *PLoS ONE* 12: e174994.
337. Fehlberg HF, Matos Ribeiro C, Brito Junior PdA, Miranda Oliveira BC, Albano dos Santos C, del Valle Alvarez MR, Harvey TV, Albuquerque GR (2021) Detection of *Cryptosporidium* spp. and *Giardia* *duodenalis* in small wild mammals in northeastern Brazil. *PLoS ONE* 16: e0256199.
338. McCarthy S, Ng J, Gordon C, Miller R, Wyber A, Ryan UM (2008) Prevalence of *Cryptosporidium* and *Giardia* species in animals in irrigation catchments in the southwest of Australia. *Experimental Parasitology* 118: 596-599.
339. Barbosa A, Reiss A, Jackson B, Warren K, Paparini A, Gillespie G, Stokeld D, Irwin P, Ryan U (2017) Prevalence, genetic diversity and potential clinical impact of blood-borne and enteric protozoan parasites in native mammals from northern Australia. *Veterinary Parasitology* 238: 94-105.
340. Krawczyk AI, Van Leeuwen AD, Jacobs-Reitsma W, Wijnands LM, Bouw E, Jahfari S, Van Hoek AHAM, Van der Giessen JWB, Roelfsema JH, Kroes M, Kleve J, Dullemont Y, Sprong H, De Bruin A (2015) Presence of zoonotic agents in engorged ticks and hedgehog faeces from Erinaceus europaeus in (sub) urban areas. *Parasites & Vectors* 8: 210.
341. Li TS, Zou Y, Peng JJ, Wang LQ, Zhang HS, Cong W, Zhu XQ, Sun XL (2020) Prevalence and genotype distribution of *Giardia duodenalis* in rabbits in Shandong province, eastern China. *BioMed Research International* Article ID: 4714735.
342. Baptista CB, Araujo MJ, Inacio SV, de Araujo Mendes BC, Costa de Aquino MC, Ferrari ED, Bresciani KDS, da Costa AJ (2023) First report of *Giardia* *duodenalis* in pet rabbits in Brazil. *Preventive Veterinary Medicine* 218: 105981.
343. Zhang W, Shen Y, Wang R, Liu A, Ling H, Li Y, Cao J, Zhang X, Shu J, Zhang L (2012) *Cryptosporidium cuniculus* and *Giardia duodenalis* in rabbits: genetic diversity and possible zoonotic transmission. *PLoS ONE* 7: e31262.
344. Qi M, Xi J, Li J, Wang H, Ning C, Zhang L (2015) Prevalence of zoonotic *Giardia duodenalis* assemblage B and first identification of assemblage E in rabbit fecal samples isolates from central China. *Journal of Eukaryotic Microbiology* 62: 810-814.
345. Jiang J, Ma JG, Zhang MZ, Xu P, Hou G, Zhao Q, Zhang XX (2018) Prevalence and risk factors of *Giardia duodenalis* in domestic rabbits (*Oryctolagus cuniculus*) in Jilin and Liaoning province, northeastern China. *Journal of Infection and Public Health* 11: 723-726.
346. Zhang X, Qi M, Jing B, Yu F, Wu Y, Chang Y, Zhao A, Wei Z, Dong H, Zhang L (2018) Molecular Characterization of *Cryptosporidium* spp., *Giardia duodenalis*, and *Enterocytozoon bieneusi* in Rabbits in Xinjiang, China. *Journal of Eukaryotic Microbiology* 65: 854-859.
347. Tang H, Ye Y, Kang R, Yu J, Cao Y (2021) Prevalence and multilocus genotyping of *Giardia duodenalis* in rabbits from Shaanxi province in northwestern China. *Parasite* 28: 54.
348. Pantchev N, Broglia A, Paoletti B, Vrhovec MG, Bertram A, Nöckler K, Cacciò SM (2014) Occurrence and molecular typing of *Giardia* isolates in pet rabbits, chinchillas, guinea pigs and ferrets collected in Europe during 2006-2012. *Veterinary Record* 175: 18.
349. Akinkuotu OA, Greenwood SJ, McClure JT, Takeet MI, Otesile EB, Olufemi F (2018) Multilocus genotyping of *Giardia duodenalis* infecting rabbits in Ogun State, Nigeria. *Veterinary Parasitology: Regional Studies and Reports* 13: 171-176.
350. Rego L, Castro-Scholten S, Cano C, Jimenez -Martin D, Koster PC, Caballero-Gomez J, Bailo B, Dashti A, Hernandez-Castro C, Cano-Terriza D, Vioque F, Maloney JG, Santin M, Garcia-Bocanegra I, Carmena D, Gonzalez-Barrio D (2023) Iberian wild leporidae as hosts of zoonotic enteroparasites in Mediterranean ecosystems of Southern Spain. *Zoonoses and Public Health* 70: 223-237.
351. Adams PJ, Monis PT, Elliot AD, Thompson RCA (2004) Cyst morphology and sequence analysis of the small subunit rDNA and *ef1*- identifies a novel *Giardia* genotype in a quenda (*Isoodon obesulus*) from Western Australia. *Infection, Genetics and Evolution* 4: 365-370.
352. Hillman A, Ash A, Elliot A, Lymbery A, Perez C, Thompson RCA (2016) Confirmation of a unique species of *Giardia*, parasitic in the quenda (*Isoodon obesulus*). International Journal for Parasitology: *Parasites and Wildlife* 5: 110-115.
353. Zhang XX, Zhang FK, Li FC, Hou JL, Zheng WB, Du SZ, Zhao Q, Zhu XQ (2017) The presence of *Giardia intestinalis* in donkeys, *Equus asinus*, in China. *Parasites & Vectors* 10: 3.
354. Li F, Wang R, Guo Y, Li N, Feng Y, Xiao L (2020) Zoonotic potential of *Enterocytozoon bieneusi* and *Giardia duodenalis* in horses and donkeys in northern China. *Parasitology Research* 119: 1101-1108.
355. Xu C, Tuo H, Wang W, Zhang Z, Yu F, Chuai L, Qi M, Jing B (2023) Occurrence and genetic characteristics of *Giardia duodenalis* in donkeys in Xinjiang, China. *Parasite* 30: 50.
356. Mukbel RM, Ghaith AO, Abu Halaweh M, Abo-Shehada MN (2017) Prevalence of *Giardia* assemblages among equines in Jordan. *Journal of* *Equine Veterinary Science* 57: 1-7.
357. Santin M, Cortes Vecino JA, Fayer R (2013) A large scale molecular study of *Giardia duodenalis* in horses from Colombia. *Veterinary Parasitology* 196: 31-36.
358. Qi M, Huan H, Wang H, Wang R, Xiao L, Arrowood MJ, Li J, Zhang L (2015) Molecular identification of *Cryptosporidium* spp. and *Giardia duodenalis* in grazing horses from Xinjiang, China. *Veterinary Parasitology* 209: 169-172.
359. Deng L, Li W, Zhong Z, Liu X, Chai Y, Luo X, Song Y, Wang W, Gong C, Huang X, Hu Y, Fu H, He M, Wang Y, Zhang Y, Wu K, Cao S, Peng G (2017) Prevalence and molecular characterization of *Giardia intestinalis* in racehorses from the Sichuan province of southwestern China. *PLoS ONE* 12: e0189728.
360. Qi M, Ji X, Zhang Y, Wei Z, Jing B, Zhang L, Lin X, Karim MdR, Wang H, Sun M (2020) Prevalence and multilocus analysis of *Giardia duodenalis* in racehorses in China. *Parasitology Research* 119: 483-490.
361. Jafari H, Razi Jalali MH, Seyfi Abad Shapouri M, Haji Hajikolaii MR (2016) Prevalence and genotyping of *Giardia duodenalis* among Arabian horses in Ahvaz, southwest of Iran. *Archives of Razi Institute* 71: 177-181.
362. Veronesi F, Passamonti F, Caccio S, Diaferia M, Piergili Fioretti D (2010) Epidemiological survey on equine *Cryptosporidium* and *Giardia* infections in Italy and molecular characterization of isolates. *Zoonoses and Public Health* 57: 510-517.
363. Traversa D, Otranto D, Milillo P, Latrofa MS, Giangaspero A, Di Cesare A, Paoletti B (2012) *Giardia duodenalis* sub-assemblage of animal and human origin in horses. *Infection, Genetics and Evolution* 12: 1642-1646.
364. Demircan K, Onder Z, Duzlu O, Yildirim A, Okur M, Ciloglu A, Yetismis G, Inci A (2019) First Molecular detection and phylogenetic analyses of zoonotic *Giardia intestinalis* in horses in Turkey. *Journal of Equine Veterinary Science* 80: 56-60.
365. Dos Reis LL, de Souza LSS, Braga FCO, Lima DCS, Lima NAS, Padinha JDS, Nava AFD, Vicente ACP (2023) Zoonotic *Giardia duodenalis* assemblage A in northern sloth from Brazilian Amazon. *Memorias do Instituo Oswaldo Cruz* 118: e230088.
366. David EB, Patti M, Coradi ST, Oliveira-Sequeira TCG, Ribolla PEM, Guimaraes S (2014) Molecular typing of *Giardia duodenalis* isolates from nonhuman primates housed in a Brazilian zoo. *Revista Instituto de Medicina Tropical de Sao Paulo* 56: 49-54.
367. Volotao ACC, Souza Junior JC, Grassini C, Peralta JM, Fernandes O (2008) Genotyping of *Giardia duodenalis* from southern brown howler monkeys (*Alouatta clamitans*) from Brazil. *Veterinary Parasitology* 158: 133-137.
368. Vitazkova SK, Wade SE (2006) Parasites of free-ranging black howler monkeys (*Alouatta pigra*) from Belize and Mexico. *American Journal of Primatology* 68: 1089-1097.
369. Köster PC, Martínez-Nevado E, González A, Abelló-Poveda MT, Fernández-Bellon H, de la Riva-Fraga M, Marquet B, Guéry JP, Knauf-Witzens T, Weigold A, Dashti A, Bailo B, Imaña E, Muadica AS, González-Barrio D, Ponce-Gordo F, Calero-Bernal R, Carmena D (2022) Intestinal protists in captive non-human primates and their handlers in six european zoological gardens. molecular evidence of zoonotic transmission. *Frontiers in* *Veterinary Science* 8: 819887.
370. Du SZ, Zhao GH, Shao JF, Fang YQ, Tian GR, Zhang LX, Wang RJ, Wang HY, Qi M, Yu SK (2015) *Cryptosporidium* spp., *Giardia intestinalis*, and *Enterocytozoon bieneusi* in captive non-human primates in Qinling mountains. Korean Journal of Parasitology 53: 395-402.
371. Karim MdR, Wang R, Yu F, Li T, Dong H, Li D, Zhang L, Li J, Jian F, Zhang S, Rume FI, Ning C, Xiao L (2015) Multilocus analysis of *Giardia duodenalis* from nonhuman primates kept in zoos in China: geographical segregation and host-adaptation of assemblage B isolates. *Infection,* *Genetics and Evolution* 30: 82-88.
372. Johnston AR, Gillespie TR, Rwego IB, Tranby McLachlan TL, Kent AD, Goldberg TL (2010) Molecular epidemiology of Cross-species *Giardia duodenalis* transmission in Western Uganda. *PLoS Neglected Tropical Diseases* 4: e638.
373. Martinez-Diaz RA, Sansano-Maestre J, Martínez-Herrero MdC, Ponce-Gordo F, Gómez-Muñoz MT (2011) Occurrence and genetic characterization of *Giardia duodenalis* from captive nonhuman primates by multilocus sequence analysis. *Parasitology Research* 109: 539-544.
374. Zhang X, Wang L, Lan X, Dan J, Ren Z, Cao S, Shen L, Deng J, Zuo Z, Yu S, Wang Y, Ma X, Liu H, Zhou Z, Hu Y, Fu H, He C, Geng Y, Gu X, Peng G, Wang Y, Zhong Z (2020) Occurrence and multilocus genotyping of *Giardia duodenalis* in captive nonhuman primates from 12 zoos in China. *PLoS* *ONE* 15: e0228673.
375. Karim MdR, Zhang S, Jian F, Li J, Zhou C, Zhang L, Sun M, Yang G, Zou F, Dong H, Li J, Rume FI, Qi M, Wang R, Ning C, Xiao L (2014) Multilocus typing of *Cryptosporidium* spp. and *Giardia duodenalis* from non-human primates in China. *International Journal for Parasitology* 44: 1039-1047.
376. Ye J, Xiao L, Li J, Huang W, Amer SE, Guo Y, Roellig D, Feng Y (2014) Occurrence of human-pathogenic *Enterocytozoon bieneusi*, *Giardia duodenalis* and *Cryptosporidium* genotypes in laboratory macaques in Guangxi, China. *Parasitology International* 63: 132-137.
377. Zhong Z, Tian Y, Li W, Huang X, Deng L, Cao S, Geng Y, Fu H, Shen L, Liu H, Peng G (2017) Multilocus genotyping of *Giardia duodenalis* in captive non-human primates in Sichuan and Guizhou provinces, Southwestern China. *PLoS ONE* 12: e0184913.
378. Chen L, Zhao J, Li N, Guo Y, Feng Y, Feng Y, Xiao L (2019) Genotypes and public health potential of *Enterocytozoon bieneusi* and *Giardia* *duodenalis* in crab‑eating macaques. *Parasites & Vectors* 12: 254.
379. Sricharern W, Inpankaew T, Keawmongkol S, Supanam J, Stich RW, Jittapalapong S (2016) Molecular detection and prevalence of *Giardia duodenalis* and *Cryptosporidium* spp. among long-tailed macaques (*Macaca fascicularis*) in Thailand. Infection, Genetics and Evolution 40: 310-314.
380. Ye J, Xiao L, Ma J, Guo M, Liu L, Feng Y (2012) Anthroponotic enteric parasites in monkeys in public Park, China. *Emerging Infectious Diseases*. 18: 1640-1643.
381. Shu F, Song S, Wei Y, Li F, Guo Y, Feng Y, Xiao L, Li N (2022) High zoonotic potential of *Cryptosporidium* spp., *Giardia duodenalis*, and *Enterocytozoon bieneusi* in wild nonhuman primates from Yunnan Province, China. *Parasites & Vectors* 15: 85.
382. Hogan JN, [Miller](https://pubmed.ncbi.nlm.nih.gov/?term=Miller+WA&cauthor_id=24171566) WA, [Cranfield](https://pubmed.ncbi.nlm.nih.gov/?term=Cranfield+MR&cauthor_id=24171566) MR, [Ramer](https://pubmed.ncbi.nlm.nih.gov/?term=Ramer+J&cauthor_id=24171566) J, [Hassell](https://pubmed.ncbi.nlm.nih.gov/?term=Hassell+J&cauthor_id=24171566) J, [Noheri](https://pubmed.ncbi.nlm.nih.gov/?term=Noheri+JB&cauthor_id=24171566) JB, [Conrad](https://pubmed.ncbi.nlm.nih.gov/?term=Conrad+PA&cauthor_id=24171566) PA,  [Gilardi](https://pubmed.ncbi.nlm.nih.gov/?term=Gilardi+KV&cauthor_id=24171566) KVK (2014) *Giardia* in mountain gorillas (*Gorilla beringei beringei*), forest buffalo (*Syncerus caffer*), and domestic cattle in Volcanoes National Park, Rwanda. *Journal of Wildlife Diseases* 50: 21-30.
383. Willenborg CM, Cervena B, Thompson P, Rosario E, Ruaux C, Vogelnest L, Slapeta J (2022) *Giardia duodenalis* in a clinically healthy population of captive zoo chimpanzees: Rapid antigen testing, diagnostic real-time PCR and faecal microbiota profiling. International Journal for Parasitology: *Parasites and Wildlife* 17: 308-318.
384. Tangtrongsup S, Sripakdee D, Malaivijitnond S, Angkuratipakorn R, Lappin M (2019) Intestinal parasites and the occurrence of zoonotic *Giardia duodenalis* genotype in captive gibbons at krabokkoo wildlife breeding center, Thailand. *Frontiers in Veterinary Science* 6: 110.
385. Berrilli F, Prisco C, Friedrich KG, Cerbo PD, Di Cave D, De Liberato C (2011) *Giardia duodenalis* assemblages and *Entamoeba* species infecting non-human primates in an Italian zoological garden: zoonotic potential and management traits. *Parasites & Vectors* 4: 199.
386. Fayer R, Santin M, Trout JM, DeStefano S, Koenen K, Kaur T (2006) Prevalence of *Microsporidia*, *Cryptosporidium* spp., and *Giardia* spp. in beavers (*Castor canadensis*) in Massachusetts. *Journal of Zoo and Wildlife Medicine* 37: 492-497.
387. Qi M, Yu F, Li S, Wang H, Luo N, Huang J, Zhang L (2015) Multilocus genotyping of potentially zoonotic *Giardia duodenalis* in pet chinchillas (*Chinchilla lanigera*) in China. *Veterinary Parasitology* 208: 113-117.
388. Veronesi F, Piergili Fioretti D, Morganti G, Bietta A, Moretta I, Moretti A, Traversa D (2012) Occurrence of *Giardia duodenalis* infection inchinchillas (*Chincilla lanigera*) from Italian breeding facilities. *Research in Veterinary Science* 93: 807-810.
389. Gherman CM, Kalmár Z, Györke A, Mircean V (2018) Occurrence of *Giardia duodenalis* assemblages in farmed long-tailed chinchillas *Chinchilla lanigera* (Rodentia) from Romania. *Parasites & Vectors* 11: 86.
390. Lux L, Ulrich RG, Santos-Silva S, Queirós J, Imholt C, Klotz C, Paupério J, Pita R, Vale-Gonçalves H, Alves PC, Mesquita JR (2023) Detection and molecular characterization of *Giardia* and *Cryptosporidium* spp. circulating in wild small mammals from Portugal. *Animals* 13: 515.
391. Helmy YA, Spierling NG, Schmidt S, Rosenfeld UM, Reil D, Imholt C, Jacob J, Ulrich RG, Aebischer T, Klotz C (2018) Occurrence and distribution of *Giardia* species in wild rodents in Germany. *Parasites & Vectors* 11: 213.
392. Perec-Matysiak A, Buńkowska-Gawlik K, Zaleśny G, Hildebrand J (2015) Small rodents as reservoirs of *Cryptosporidium* spp. and *Giardia* spp. in south-western Poland. *Annals of Agricultural and Environmental Medicine* 22: 1-5.
393. Vioque F, Dashti A, Santin M, Ruiz-Fons F, Koster PC, Hernandez-Castro C, Garcia JT, Bailo B, Ortega S, Olea PP, Arce F, Chicharro C, Nieto J, Gonzalez F, Vinuela J, Carmena D, Gonzalez-Barrio D (2022) Wild micromammal host spectrum of zoonotic eukaryotic parasites in Spain. Occurrence and genetic characterisation. *Transboundary and Emerging Diseases* 1-17.
394. Lyu Z, Shao J, Xue M, Ye Q, Chen B, Qin Y, Wen J (2018) A new species of *Giardia* Kunstler, 1882 (Sarcomastigophora: Hexamitidae) in hamsters. *Parasites & Vectors* 11: 202.
395. Mohebali M, Zarei Z, Khanaliha K, Kia EB, Motavalli-Haghi A, Davoodi J, Rezaeian T, Tarighi F, Rezaeian M (2017) Natural intestinal protozoa in rodents (Rodentia: Gerbillinae, Murinae, Cricetinae) in northwestern Iran. *Iranian Journal of Parasitology* 12: 382-388.
396. Kilonzo C, Li X, Vivas EJ, Jay-Russell MT, Fernandez KL, Atwill ER (2013) Fecal shedding of zoonotic food-borne pathogens by wild rodents in a major agricultural region of the central California Coast. *Applied and Environmental Microbiology* 79: 6337-6344.
397. Cui Z, Wang D, Wang W, Zhang Y, Jing B, Xu C, Chen Y, Qi M, Zhang L (2021) Occurrence and multilocus analysis of *Giardia* *duodenalis* in coypus (*Myocastor coypus*) in China. *Pathogens* 10: 179.
398. Coppola F, Maestrini M, Berrilli F, Procesi IG, Felicioli A, Perrucci S (2020) First report of *Giardia duodenalis* infection in the crested porcupine (*Hystrix* *cristata* L., 1758). *International Journal for Parasitology:* *Parasites and Wildlife* 11: 108-113.
399. Carriquiriborde M, Milocco S, Laborde JM, Gentil F, Maschi F, Principi G, Rogers E, Cagliada MdP, Ayala MA, Carbone C (2020) Microbiological contaminations of laboratory miceand rats in conventional facilities in Argentina. *Revista Argentina de Microbiologia* 52: 96-100.
400. Zhao Z, Wang R, Zhao W, Qi M, Zhao J, Zhang L, Li J, Liu A (2015) Genotyping and subtyping of *Giardia* and *Cryptosporidium* isolates from commensal rodents in China. *Parasitology* 142: 800-806.
401. Asghari A, Motazedian MH, Asgari Q, Shamsi L, Sarkari B, Shahabi S, Mohammadi-Ghalebin B (2022) Occurrence, genetic characterization, and zoonotic importance of *Giardia duodenalis* in various species of rodents (*Mus musculus*, *Rattus norvegicus*, and *Rattus rattus*). *Comparative Immunology, Microbiology and Infectious Disease* 85: 101812.
402. Fernandez-Alvarez A, Martin-Alonso A, Abreu-Acosta N, Feliu C, Hugot JP, Valladares B, Foronda P (2014) Identification of a novel assemblage G subgenotype and a zoonotic assemblage B in rodent isolates of *Giardia duodenalis* in the Canary Islands, Spain. *Parasitology* 141: 206-215.
403. Masakul A, Thaprathom N, Chaisiri K, Karnchanabanthoeng A, Inpankaew T, Desquesnes M, Morand S, Herder S, Binot A, Jittapalapong S (2016) Infection rates of *Giardia duodenalis* associated with diversity of rodents in Thailand. In: *Proceedings of 54^th^ Kasetsart University Annual Conference: Plants, Animals, Veterinary Medicine, Fisheries, Agricultural Extension and Home Economics*. *Bangkok, Thailand*. Available from: file:///C:/Users/Computer.icu/Downloads/KC5403003-4.pdf
404. Li J, Lang P, Huang M, Jing B, Karim MdR, Chao L, Wang Z, Lv Y, Li J, Qi M (2020) Molecular characterization of *Cryptosporidium* spp. and *Giardia duodenalis* in experimental rats in China. *Parasitology International* 77: 102127.
405. Galán-Puchades MT, Trelis M, Sáez-Durán S, Cifre S, Gosálvez C, Sanxis-Furió J, Pascual J, Bueno-Marí R, Franco S, Peracho V, Montalvo T, Fuentes MV (2021) One Health Approach to Zoonotic Parasites: Molecular detection of intestinal protozoans in an urban population of Norway rats, *Rattus norvegicus*, in Barcelona, Spain. *Pathogens* 10: 311.
406. Cervero-Arago S, Desvars-Larrive A, Lindner G, Sommer R, Hafeli I, Walochnik J (2021) Surface waters and urban brown rats as potential sources of human-infective *Cryptosporidium* and *Giardia* in Vienna, Austria. *Microrganisms* 9: 1596.
407. Tan TK, Low VL, Ng WH, Ibrahim J, Wang D, Tan CH, Chellappan S, Lim YAL (2019) Occurrence of zoonotic *Cryptosporidium* and *Giardia duodenalis* species/genotypes in urban rodents. *Parasitology International* 69: 110-113.
408. Deng L, Luo R, Liu H, Zhou Z, Li L, Chai Y, Yang L, Wang W, Fu H, Zhong Z, Cao S Peng G (2018) First identification and multilocus genotyping of *Giardia* *duodenalis* in pet chipmunks (*Eutamias asiaticus*) in Sichuan Province, southwestern China. *Parasites & Vectors* 11: 199.
409. Xu J, Liu H, Jiang Y, Jing H, Cao J, Yin J, Li T, Sun Y, Shen Y, Wang X (2022) Genotyping and subtyping of *Cryptosporidium* spp. and *Giardia* *duodenalis* isolates from two wild rodent species in Gansu Province, China. *Scientific Reports* 12: 12178.
410. Ma X, Wang Y, Zhang HJ, Wu HX, Zhao GH (2018) First report of *Giardia duodenalis* infection in bamboo rats. *Parasites & Vectors* 11: 520.
