# Supplementary material for: Global prevalence of Giardia infection in nonhuman mammalian hosts: A systematic review and meta-analysis of five million animals
Source: PLoS Negl Trop Dis. 2025 Apr 24;19(4):e0013021. doi: 10.1371/journal.pntd.0013021 (PMC12052165; doi:10.1371/journal.pntd.0013021)
Supplement: S12 Table — (DOC) [file pntd.0013021.s013.doc]

**S12 Table.** *Giardia duodenalis* subassemblages AI and AII in humans and animals.

| **Host** | **Country** | **Locus** | **Isolates (*n*)** | **Subassemblages (*n*)** | | **References** |
| --- | --- | --- | --- | --- | --- | --- |
|  |  |  |  | **AI** | **AII** |  |
| Human | Belgium | MLG | 18 |  | 18 | [1] |
| Human | France | *gdh*/*tpi* | 8 |  | 8 | [2] |
| Human | Germany | *bg* | 3 |  | 3 | [3] |
| Human | Italy | *bg* | 35 | 7 | 28 | [4–6] |
| Human | Portugal | *bg* | 27 | 25 | 2 | [7, 8] |
| Human | UK | *tpi* | 21 |  | 21 | [9, 10] |
| Human | Mexico | *vsp*/*bg* | 54 | 26 | 28 | [11–13] |
| Human | Argentina | *tpi* | 3 |  | 3 | [14] |
| Human | Brazil | *gdh/bg* | 91 | 60 | 31 | [15, 16] |
| Human | Nicaragua | *bg* | 16 |  | 16 | [17] |
| Human | Peru | *gdh/tpi* | 102 | 9 | 93 | [18–20] |
| Human | Bangladesh | *tpi* | 29 | 8 | 20 | [21] |
| Human | China | *tpi* | 12 | 8 | 4 | [22] |
| Human | Japan | *gdh* | 2 |  | 2 | [23] |
| Human | India | *tpi* | 8 | 5 | 3 | [24] |
| Human | Philippines | *tpi* | 50 | 3 | 47 | [25] |
| Human | Saudi Arabia | *igs* | 25 | 12 | 11 | [26] |
| Human | Thailand | *gdh/bg* | 23 | 3 | 20 | [27, 28] |
| Human | Australia | *gdh* | 31 | 1 | 30 | [29, 30] |
| Human | Ethiopia | *bg* | 23 | 1 | 22 | [31] |
| Cattle | Denmark | *gdh* | 8 | 8 |  | [32] |
| Cattle | Italy | MLG | 4 | 4 |  | [33] |
| Cattle | Portugal | *gdh/bg* | 2 |  | 2 | [34] |
| Cattle | USA | *tpi* | 10 | 4 | 6 | [35] |
| Cattle | Brazil | *gdh* | 1 | 1 |  | [15] |
| Buffalo | Italy | MLG | 2 | 2 |  | [36] |
| Sheep | Belgium | *bg* | 2 |  | 2 | [37] |
| Sheep | Italy | *gdh/bg* | 5 | 5 |  | [38] |
| Sheep | Spain | *bg* | 1 | 1 |  | [39] |
| Sheep | Sweden | MLG | 7 | 7 |  | [40] |
| Sheep | Australia | *tpi* | 30 | 29 | 1 | [41] |
| Goat | Belgium | *bg* | 6 | 6 |  | [38] |
| Pig | Denmark | *gdh* | 10 | 10 |  | [32] |
| Pig | Italy | MLG | 1 | 1 |  | [33] |
| Horse | USA | *tpi* | 4 | 3 | 1 | [42] |
| Dog | Belgium | *bg* | 38 |  | 38 | [43] |
| Dog | Germany | *gdh* | 14 | 14 |  | [44] |
| Dog | Italy | *bg* | 17 | 16 | 1 | [4, 5, 45] |
| Dog | Mexico | *vsp/bg* | 8 | 5 | 3 | [4, 12] |
| Cat | Italy | MLG | 3 | 2 | 1 | [33] |
| Cat | Sweden | MLG | 3 | 3 |  | [40] |
| Cat | Mexico | *vsp* | 1 |  | 1 | [12] |
| Cat | Brazil | *gdh/bg* | 9 | 9 |  | [15, 16] |

MLG: multilocus genotype analyses of the *gdh*, *bg*, and *tpi* genes; *gdh*: glutamate dehydrogenase; *tpi*: triosephosphate isomerase; *bg*: beta-giardin; *igs*: intergenic spacer of the rRNA gene; *vsp*: variant surface protein gene.

References

1. Geurden T, Levecke B, Cacció SM, Visser A, De Groote G, Casaert S, et al. Multilocus genotyping of *Cryptosporidium* and *Giardia* in non-outbreak related cases of diarrhoea in human patients in Belgium. Parasitology 2009;136(10):1161–8.
2. Bertrand I, Albertini L, Schwartzbrod J. Comparison of two target genes for detection and genotyping of *Giardia lamblia* in human feces by PCR and PCR-restriction fragment length polymorphism. J Clin Microbiol. 2005;43(12):5940–4.
3. Sagebiel D, Weitzel T, Stark K, Leitmeyer K. Giardiasis in kindergartens: prevalence study in Berlin, Germany, 2006. Parasitol Res. 2009;105(3):681–7.
4. Lalle M, Pozio E, Capelli G, Bruschi F, Crotti D, Cacciò SM. Genetic heterogeneity at the beta-giardin locus among human and animal isolates of *Giardia duodenalis* and identification of potentially zoonotic subgenotypes. Int J Parasitol. 2005;35(2):207–13.
5. Marangi M, Berrilli F, Otranto D, Giangaspero A. Genotyping of *Giardia duodenalis* among children and dogs in a closed socially deprived community from Italy. Zoonoses Public Health. 2010;57(7-8):e54–8.
6. Cacciò SM, De Giacomo M, Pozio E. Sequence analysis of the beta-giardin gene and development of a polymerase chain reaction-restriction fragment length polymorphism assay to genotype *Giardia duodenalis* cysts from human faecal samples. Int J Parasitol. 2002;32(8):1023–30.
7. Almeida AA, Delgado ML, Soares SC, Castro AO, Moreira MJ, Mendonça CM, et al. Genotype analysis of *Giardia* isolated from asymptomatic children in northern Portugal. J Eukaryot Microbiol. 2006;53 Suppl 1:S177–8.
8. Sousa MC, Morais JB, Machado JE, Poiares-da-Silva J. Genotyping of *Giardia lamblia* human isolates from Portugal by PCRRFLP and sequencing. J Eukaryot Microbiol. 2006;53 Suppl 1:S174–6.
9. Amar CF, Dear PH, Pedraza-Díaz S, Looker N, Linnane E, McLauchlin J. Sensitive PCR-restriction fragment length polymorphism assay for detection and genotyping of *Giardia duodenalis* in human feces. J Clin Microbiol. 2002;40(2):446–52.
10. Breathnach AS, McHugh TD, Butcher PD. Prevalence and clinical correlations of genetic subtypes of *Giardia lamblia* in an urban setting. Epidemiol Infect. 2010;138(10):1459–67.
11. Lalle M, Jimenez-Cardosa E, Cacciò SM, Pozio E. Genotyping of *Giardia duodenalis* from humans and dogs from Mexico using a beta-giardin nested polymerase chain reaction assay. J Parasitol. 2005;91(1):203–5.
12. Ponce-Macotela M, Martínez-Gordillo MN, Bermúdez-Cruz RM, Salazar-Schettino PM, Ortega-Pierres G, Ey PL. Unusual prevalence of the *Giardia intestinalis* A-II subtype amongst isolates from humans and domestic animals in Mexico. Int J Parasitol. 2002;32(9):1201–2.
13. Eligio-García L, Cortes-Campos A, Cota-Guajardo S, Gaxiola S, Jiménez-Cardoso E. Frequency of *Giardia intestinalis* assemblages isolated from dogs and humans in a community from Culiacan, Sinaloa, Mexico using beta-giardin restriction gene. Vet Parasitol. 2008;156(3–4):205–9.
14. Minvielle MC, Molina NB, Polverino D, Basualdo JA. First genotyping of *Giardia lamblia* from human and animal feces in Argentina, South America. Mem Inst Oswaldo Cruz. 2008;103(1):98–103.
15. Souza SL, Gennari SM, Richtzenhain LJ, Pena HF, Funada MR, Cortez A. Molecular identification of *Giardia duodenalis* isolates from humans, dogs, cats and cattle from the state of Sao Paulo, Brazil, by sequence analysis of fragments of glutamate dehydrogenase (*gdh*) coding gene. Vet Parasitol. 2007;149(3–4):258–64.
16. Volotão AC, Costa-Macedo LM, Haddad FS, Brandão A, Peralta JM, Fernandes O. Genotyping of *Giardia duodenalis* from human and animal samples from Brazil using beta-giardin gene: a phylogenetic analysis. Acta Trop. 2007 Apr;102(1):10–9.
17. Lebbad M, Ankarklev J, Tellez A, Leiva B, Andersson JO, Svärd S. Dominance of *Giardia* assemblage B in Leon, Nicaragua. Acta Trop. 2008;106(1):44–53.
18. Cooper MA, Sterling CR, Gilman RH, Cama V, Ortega Y, Adam RD. Molecular analysis of household transmission of *Giardia lamblia* in a region of high endemicity in Peru. J Infect Dis. 2010;202(11):1713–21.
19. Peréz Cordón G, Cordova Paz Soldan O, Vargas Vásquez F, Velasco Soto JR, Sempere Bordes L, Sánchez Moreno M, et al. Prevalence of enteroparasites and genotyping of *Giardia lamblia* in Peruvian children. Parasitol Res. 2008;103(2):459–65.
20. Sulaiman IM, Fayer R, Bern C, Gilman RH, Trout JM, Schantz PM, et al. Triosephosphate isomerase gene characterization and potential zoonotic transmission of *Giardia duodenalis*. Emerg Infect Dis. 2003;9(11):1444–52.
21. Haque R, Roy S, Kabir M, Stroup SE, Mondal D, Houpt ER. *Giardia* assemblage A infection and diarrhea in Bangladesh. J Infect Dis. 2005;192(12):2171–3.
22. Wang R, Zhang X, Zhu H, Zhang L, Feng Y, Jian F, et al. Genetic characterizations of *Cryptosporidium* spp. and *Giardia duodenalis* in humans in Henan, China. Exp Parasitol. 2011;127(1):42–5.
23. Abe N, Kimata I, Tokoro M. Genotyping of *Giardia* isolates from humans in Japan using the small subunit ribosomal RNA and glutamate dehydrogenase gene sequences. Jpn J Infect Dis. 2005;58(1):57–8.
24. Traub RJ, Monis PT, Robertson I, Irwin P, Mencke N, Thompson RC. Epidemiological and molecular evidence supports the zoonotic transmission of *Giardia* among humans and dogs living in the same community. Parasitology. 2004;128(Pt 3):253–62.
25. Yason JA, Rivera WL. Genotyping of *Giardia duodenalis* isolates among residents of slum area in Manila, Philippines. Parasitol Res. 2007;101(3):681–7.
26. Al-Mohammed HI. Genotypes of *Giardia intestinalis* clinical isolates of gastrointestinal symptomatic and asymptomatic Saudi children. Parasitol Res. 2011;108(6):1375–81.
27. Ratanapo S, Mungthin M, Soontrapa S, Faithed C, Siripattanapipong S, Rangsin R, et al. Multiple modes of transmission of giardiasis in primary schoolchildren of a rural community, Thailand. Am J Trop Med Hyg. 2008;78(4):611–5.
28. Tungtrongchitr A, Sookrung N, Indrawattana N, Kwangsi S, Ongrotchanakun J, Chaicumpa W. *Giardia intestinalis* in Thailand: identification of genotypes. J Health Popul Nutr. 2010;28(1):42–52.
29. Read CM, Monis PT, Thompson RC. Discrimination of all genotypes of *Giardia duodenalis* at the glutamate dehydrogenase locus using PCR-RFLP. Infect Genet Evol. 2004;4(2):125–30.
30. Yang R, Lee J, Ng J, Ryan U. High prevalence *Giardia duodenalis* assemblage B and potentially zoonotic subtypes in sporadic human cases in Western Australia. Int J Parasitol. 2010;40(3):293–7.
31. Gelanew T, Lalle M, Hailu A, Pozio E, Cacciò SM. Molecular characterization of human isolates of *Giardia duodenalis* from Ethiopia. Acta Trop. 2007;102(2):92–9.
32. Langkjaer RB, Vigre H, Enemark HL, Maddox-Hyttel C. Molecular and phylogenetic characterization of *Cryptosporidium* and *Giardia* from pigs and cattle in Denmark. Parasitology. 2007;134(Pt 3):339–50.
33. Cacciò SM, Beck R, Lalle M, Marinculic A, Pozio E. Multilocus genotyping of *Giardia duodenalis* reveals striking differences between assemblages A and B. Int J Parasitol. 2008;38(13):1523–31.
34. Mendonça C, Almeida A, Castro A, de Lurdes Delgado M, Soares S, da Costa JM, et al. Molecular characterization of *Cryptosporidium* and *Giardia* isolates from cattle from Portugal. Vet Parasitol. 2007;147(1–2):47–50.
35. Feng Y, Ortega Y, Cama V, Terrel J, Xiao L. High intragenotypic diversity of *Giardia duodenalis* in dairy cattle on three farms. Parasitol Res. 2008;103(1):87–92.
36. Cacciò SM, Rinaldi L, Cringoli G, Condoleo R, Pozio E. Molecular identification of *Cryptosporidium parvum* and *Giardia duodenalis* in the Italian water buffalo (*Bubalus bubalis*). Vet Parasitol. 2007;150(1–2):146–9.
37. Geurden T, Thomas P, Casaert S, Vercruysse J, Claerebout E. Prevalence and molecular characterisation of *Cryptosporidium* and *Giardia* in lambs and goat kids in Belgium. Vet Parasitol. 2008;155(1–2):142–5.
38. Giangaspero A, Paoletti B, Iorio R, Traversa D. Prevalence and molecular characterization of *Giardia duodenalis* from sheep in central Italy. Parasitol Res. 2005;96(1):32–7.
39. Gómez-Muñoz MT, Navarro C, Garijo-Toledo MM, Dea-Ayuela MA, Fernández-Barredo S, Pérez-Gracia MT, et al. Occurrence and genotypes of *Giardia* isolated from lambs in Spain. Parasitol Int. 2009;58(3):297–9.
40. Lebbad M, Mattsson JG, Christensson B, Ljungström B, Backhans A, Andersson JO, et al. From mouse to moose: multilocus genotyping of *Giardia* isolates from various animal species. Vet Parasitol. 2010;168(3–4):231–9.
41. Nolan MJ, Jex AR, Pangasa A, Young ND, Campbell AJ, Stevens M, et al. Analysis of nucleotide variation within the triose-phosphate isomerase gene of *Giardia duodenalis* from sheep and its zoonotic implications. Electrophoresis. 2010;31(2):287–98.
42. Traub R, Wade S, Read C, Thompson A, Mohammed H. Molecular characterization of potentially zoonotic isolates of *Giardia duodenalis* in horses. Vet Parasitol. 2005;130(3–4):317–21.
43. Claerebout E, Casaert S, Dalemans AC, De Wilde N, Levecke B, Vercruysse J, et al. *Giardia* and other intestinal parasites in different dog populations in Northern Belgium. Vet Parasitol. 2009;161(1–2):41–6.
44. Leonhard S, Pfister K, Beelitz P, Wielinga C, Thompson RC. The molecular characterisation of *Giardia* from dogs in southern Germany. Vet Parasitol. 2007;150(1–2):33–8.
45. Paoletti B, Iorio R, Capelli G, Sparagano OA, Giangaspero A. Epidemiological scenario of giardiosis in dogs from central Italy. Ann N Y Acad Sci. 2008;1149:371–4.
